# Bismuth‐Mediated α‐Arylation of Acidic Diketones with *ortho*‐Substituted Boronic Acids

**DOI:** 10.1002/anie.202210840

**Published:** 2022-08-29

**Authors:** Katie Ruffell, Stephen P. Argent, Kenneth B. Ling, Liam T. Ball

**Affiliations:** ^1^ School of Chemistry University of Nottingham Nottingham NG7 2RD UK; ^2^ Syngenta Jealott's Hill International Research Centre Bracknell RG42 6EY UK

**Keywords:** 1,3-Diketones, Bismacycle, Bismuth, Pyrazoles, α-Arylation

## Abstract

The α‐arylation of cyclic and fluoroalkyl 1,3‐diketones is made challenging by the highly stabilized nature of the corresponding enolates, and is especially difficult for sterically demanding aryl partners. As a general solution to this problem, we report the Bi‐mediated oxidative coupling of acidic diones and *ortho*‐substituted arylboronic acids. Starting from a bench‐stable bismacycle precursor, a sequence of B‐to‐Bi transmetallation, oxidation and C−C bond formation furnishes the arylated diones. Development of methodology that tolerates both sensitive functionality and steric demand is supported by interrogation of key reactive intermediates. Application of our strategy to cyclic diones enables the concise synthesis of important agrochemical intermediates which were previously prepared using toxic Pb reagents. This methodology also enables the first ever arylation of fluoroalkyl diones which, upon condensation with hydrazine, provides direct access to valuable fluoroalkyl pyrazoles.

## Graphical abstract

A modular, bismacycle‐mediated method is disclosed for the oxidative coupling of cyclic and fluoroalkyl diketones with *ortho*‐substituted arylboronic acids. A wide range of functionality is tolerated on both partners, providing direct access to agrochemically and pharmaceutically important motifs that are currently inaccessible by other approaches.

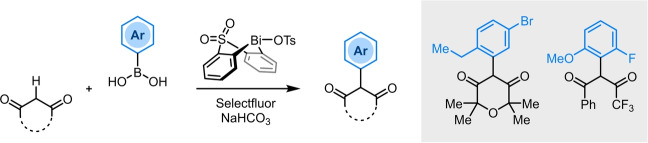

## Introduction

Metal‐catalyzed cross‐coupling of C‐, N‐, O‐ and S‐nucleophiles is an essential tool for the discovery, development and production of biologically active molecules.[^1^, ^2^, ^3^, ^4^] However, despite the highly sophisticated state of the art, the arylation of weak (stabilized) nucleophiles^[5]^ remains challenging due to the low rate of reductive elimination from highly polarized M−R bonds.^[6]^ This limitation is particularly apparent in the α‐arylation of carbonyl compounds: while couplings of simple ketones, esters, amides and malonates are facile,[^7^, ^8^, ^9^, ^10^] the analogous couplings of cyclic 1,3‐diketones are less well developed[^11^, ^12^, ^13^, ^14^, ^15^] and couplings of fluoroalkyl‐substituted 1,3‐diketones are entirely unknown.

**Scheme 1:**
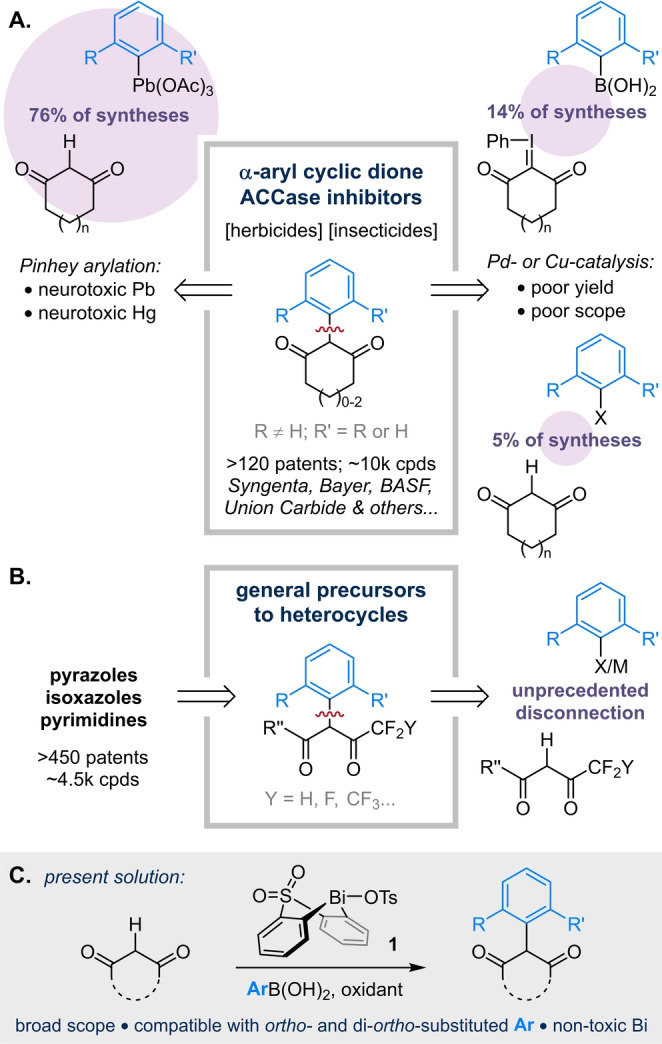
Strategies for the α‐arylation of acidic 1,3‐diketones. a) coupling of cyclic diones with *ortho*‐substituted aryl partners; b) couplings of fluoroalkyl diones with any aryl partner; c) a bismacycle‐based platform for the oxidative coupling of acidic diones with *ortho*‐substituted arylboronic acids.

Expedient α‐arylation of these acidic 1,3‐diketones[^16^, ^17^] is of direct importance to discovery chemistry. For example, α‐aryl cyclic diones have applications in human health[^18^, ^19^] and particularly in plant care, where they represent the core of an emerging class of herbicidal and insecticidal ACCase inhibitors.[^20^, ^21^] Notably, nearly 10 000 (60 %) of the compounds claimed in >120 agrochemical patents bear at least one *ortho* substituent on the aryl moiety (Scheme 1A), a feature which significantly hinders cross‐coupling approaches to this motif (see below). In contrast, α‐arylated fluoroalkyl diones are valued as key *precursors* to biologically relevant heterocycles, including pyrazoles, isoxazoles and pyrimidines (Scheme 1B).[^22^, ^23^, ^24^, ^25^] Together, these arylated fluoroalkyl heterocycles account for over 4 500 patented compounds with indications including obesity, inflammation, cancer and Huntington's disease.

Palladium‐catalyzed methods for the cross‐coupling of cyclic diones are based on two reports from Buchwald covering just four examples,[^11^, ^12^] all of which involve non‐*ortho*‐substituted aryl halides. Tellingly, Landais observes that this methodology does not tolerate *ortho*‐substitution,^[13]^ while Tanner notes that couplings of related tetramic acids are also inhibited for *ortho*‐substituted aryl halides.^[26]^ Cu‐catalyzed arylations of cyclic diketones are similarly limited by sterics, with low yields (<25 %) reported for couplings of mono‐*ortho*‐substituted aryl iodides, and no examples reported for di‐*ortho*‐substituted aryl iodides.[^14^, ^27^, ^28^] In addition to their acidity, the cross‐coupling of fluoroalkyl diones is made even more difficult by their propensity to form hydrates[^29^, ^30^] and their sensitivity to retro‐Claisen condensation under both neutral^[31]^ and basic[^32^, ^33^] conditions. As a result, the direct coupling of fluoroalkyl diones with any aryl partner has—to the best of our knowledge—never been reported.

Given the inadequacy of cross‐couplings between cyclic or fluoroalkyl diones and *ortho*‐substituted arylating agents, these motifs are routinely prepared by alternative strategies based on: (1) multi‐step procedures that lack modularity, (2) S_N_Ar of highly activated aryl halides, which is necessarily limited in scope, or (3) the use of highly toxic reagents.[^34^, ^35^, ^36^, ^37^, ^38^, ^39^] Indeed, the synthesis of α‐aryl cyclic diones is dominated by Pinhey arylation^[40]^ with stoichiometric aryllead triacetate reagents (Scheme 1A, left), which are prepared using catalytic Hg(OAc)_2_.^[41]^ This reliance on lead chemistry is clearly undesirable due to its major implications for operator and environmental safety.

A concise and efficient method for the α‐arylation of acidic diones would therefore not only address a fundamental limitation of contemporary organic synthesis, but would also represent a valuable tool for discovery chemistry. In this manuscript, we demonstrate that modular bismuth reagents enable direct, high‐yielding arylation of cyclic and—for the first time—fluoroalkyl diketones with *ortho*‐substituted arylboronic acids (Scheme 1C). Through detailed analysis of the reaction pathways, we deliver a robust and general process for these otherwise elusive transformations. The resulting protocols are user‐friendly, employing commercial starting materials, being performed under air and without exclusion of moisture, and avoiding the use of toxic reagents. The oxidative and effectively pH‐neutral nature of our strategy confers compatibility with aryl bromides and chlorides, acidic protons and potentially reactive electrophiles, rendering it complementary to both cross‐coupling and S_N_Ar.

## Results and Discussion

**Scheme 2:**
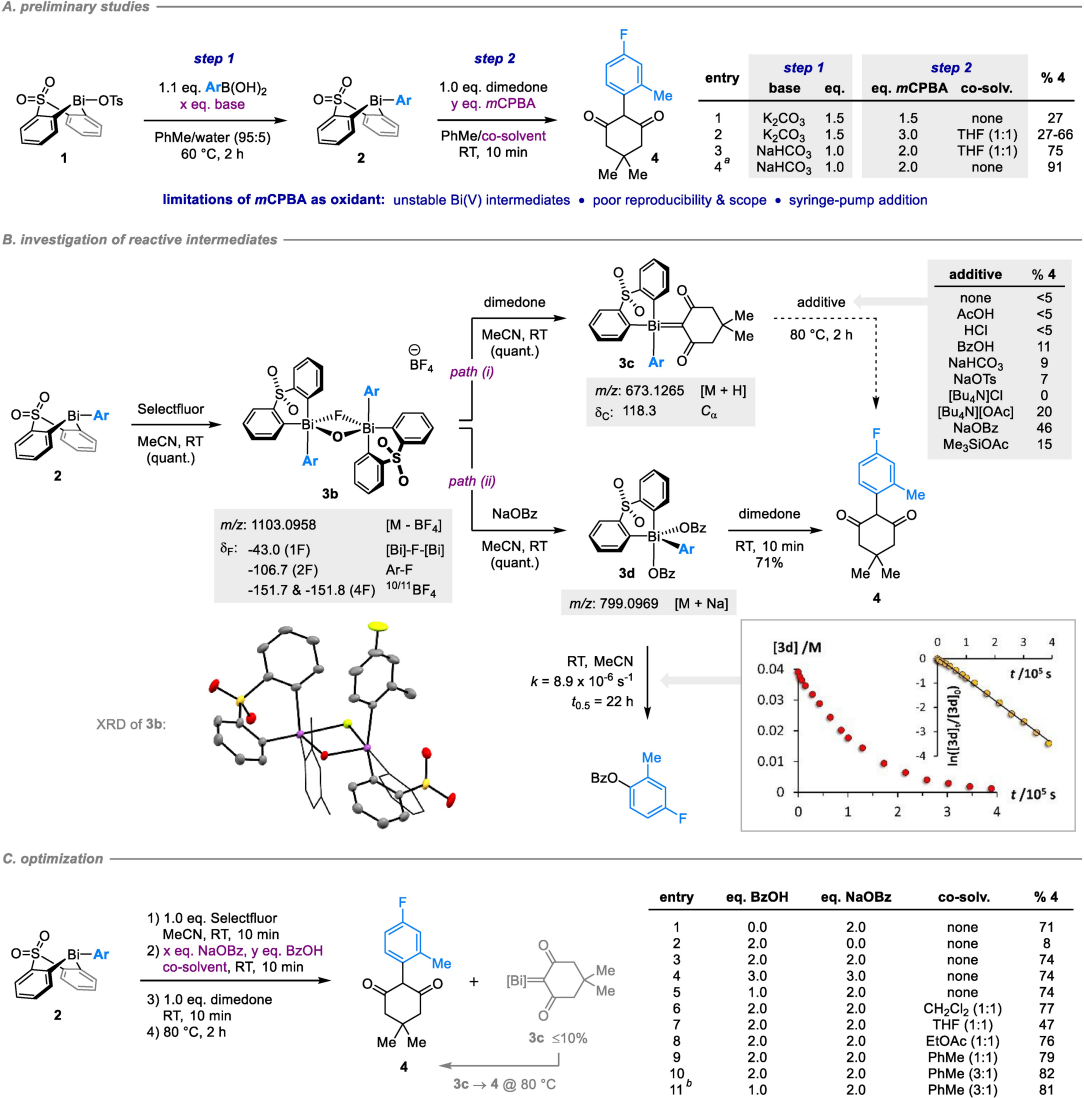
Development of the Bi^V^‐mediated α‐arylation of cyclic 1,3‐diones. a) initial studies highlight the limitations of *m*CPBA as oxidant; b) mechanistic interrogation of reaction pathways with Selectfluor as oxidant; c) optimization with Selectfluor as oxidant. Yields determined by ^19^F NMR spectroscopic analysis vs. internal standard. [a] Using 1.1 eq. bismacycle **1**, 1.1 eq. NaHCO_3_ and 1.2 eq. boronic acid; *m*CPBA added as a solution in PhMe over 20 mins. [b] BzOH and NaOBz were added together with Selectfluor in step (1). ORTEP image of **3 b**: H atoms and tetrafluoroborate omitted for clarity; thermal ellipsoids shown at 50 % probability.

We recently reported a convenient and step‐economic method for the Bi^V^‐mediated C−H arylation of phenols and naphthols with boronic acids.^[42]^ Based on the structural similarity of phenols and the enol tautomer of 1,3‐dicarbonyls, we hypothesized that a similar strategy could be developed as a general tool for the arylation of acidic diones. Our proposed procedure consists of telescoped B‐to‐Bi transmetallation from an arylboronic acid to a bench‐stable Bi^III^ precursor **1**, followed by oxidation of the resulting arylbismacycle **2** to a reactive Bi^V^ intermediate and subsequent dione arylation (cf. Scheme 2A). Crucially, the viability of the C−C bond forming step was suggested by previous reports on the α‐arylation of cyclic diones with homoleptic Bi^V^ reagents.^[43]^ In this precedent, however, only α,α‐diphenylation was observed; selective monoarylation of cyclic diones remains limited to just four examples from the patent literature (average 21 % yield).[^44^, ^45^] To the best of our knowledge, bismuth‐mediated arylation has never been applied to fluoroalkyl diones.

We began by studying the Bi^V^‐mediated arylation of cyclic diones. Initial investigations indicated that our previous conditions were unsuitable for the arylation of dimedone, furnishing the expected product in only 27 % yield (Scheme 2A, entry 1). It was subsequently determined that the oxidation/arylation process is inhibited by K_2_CO_3_ residual from the initial transmetallation, and that the Bi^V^ intermediate formed under the action of *m*CPBA was unstable towards decomposition. This instability was not encountered in our previous work^[42]^—presumably due to the higher relative rate of arylation of phenols vs. diones—and stands in contrast to the stability of analogous Bi^V^ species bearing *ortho*‐unsubstituted aryl moieties. While the use of excess *m*CPBA provided an inconsistent solution to the inhibitory effects of K_2_CO_3_ (entry 2), use of a milder base proved both more effective and more reproducible (entry 3). The inherent instability of *m*CPBA‐derived Bi^V^ species could not, however, be reliably addressed. As a consequence, the excellent yields ultimately achieved for dimedone (entry 4) could not be extended to other substrates, prompting investigation of alternative oxidants to *m*CPBA (see Supporting Information).

To this end, treatment of arylbismacycle **2** with Selectfluor in acetonitrile generated a single, stable Bi^V^ species within minutes at room temperature (Scheme 2B). The new intermediate was identified as the mixed oxo/fluoro‐bridged dimer **3 b** on the basis of in situ HRMS and NMR spectroscopy, and its assignment was confirmed crystallographically following work‐up and isolation. Isotopic labeling studies indicated that the bridging oxygen atom originates from water present in the technical‐grade solvent (see Supporting Information). While structurally related bis‐μ‐oxo‐bridged and bis‐μ‐fluoro‐bridged dimers of Bi^V^ have been reported previously,[^42^, ^46^] mixed dimers are unknown.

Addition of dimedone to dimer **3 b** affords isolable ylide **3 c** rapidly and in quantitative yield (*path (i)*, Scheme 2B), rather than the expected product of α‐arylation **4**. The few prior reports of Bi^V^ ylides indicate that these species are formed under basic conditions,[^47^, ^48^] and that although ylides derived from cyclic diones are stable and have been characterized crystallographically,^[49]^ those derived from acyclic diones undergo ligand coupling on silica gel.[^50^, ^51^] Based on this latter precedent, we hypothesised that ligand coupling may be promoted in **3 c** either by protonation of the ylidic carbon or by modification of the Bi^V^ centre through complexation. However, treatment of ylide **3 c** with various additives failed to afford high yields of the desired arylation product **4**, instead returning either arylbismacycle **2** or the products of formal protodebismuthation.

In contrast, addition of NaOBz directly to dimer **3 b** generates a new Bi^V^ species, dibenzoate **3 d** (*path (ii)*, Scheme 2B). While dibenzoate **3 d** decomposes over time in solution (*t*_0.5_=22 h at 25 °C in CD_3_CN),^[52]^ it reacts rapidly with dimedone to give product **4** in high yield. In this way less than *ca* 10 % of ylide **3 c** is formed, and this side‐product can—in part—be converted to product **4** by subsequent heating of the reaction mixture (cf. *path (i)*).

Further investigation indicated that, unlike NaOBz, the addition of BzOH alone does not promote formation of Bi^V^ dibenzoate **3 d** from dimer **3 b**, and hence does not promote productive arylation (Scheme 2C, entries 1–2). However, adding BzOH *alongside* NaOBz led to improved reaction rates and reproducibility across different dione substrates. While the yield of **4** was not affected by the relative or absolute stoichiometries of BzOH and NaOBz (entries 3–5), a small but significant increase in yield was obtained by performing the arylation step in the presence of toluene co‐solvent (entries 6–10). Finally, the oxidation/anion exchange/α‐arylation sequence could be achieved in a single telescoped operation simply by performing oxidation in the presence of BzOH and NaOBz (entry 11). Arylation of the dione at oxygen was not observed under any of the conditions investigated (see Supporting Information).

Our optimized protocol for cyclic diones thus consists of B‐to‐Bi transmetallation, solvent exchange, and combined oxidation/anion metathesis prior to α‐arylation. This overall sequence is economic in both the dione and the arylating agent, is performed under air in technical‐grade solvents, and uses readily available, non‐toxic reagents. Furthermore, the bismacycle can be recovered in >80 % yield via a simple aqueous work‐up (see Supporting Information). It therefore represents an efficient and convenient method for the coupling of cyclic diones with *ortho*‐substituted aryl partners, a transformation that is extremely difficult to achieve by existing strategies.

**Scheme 3:**
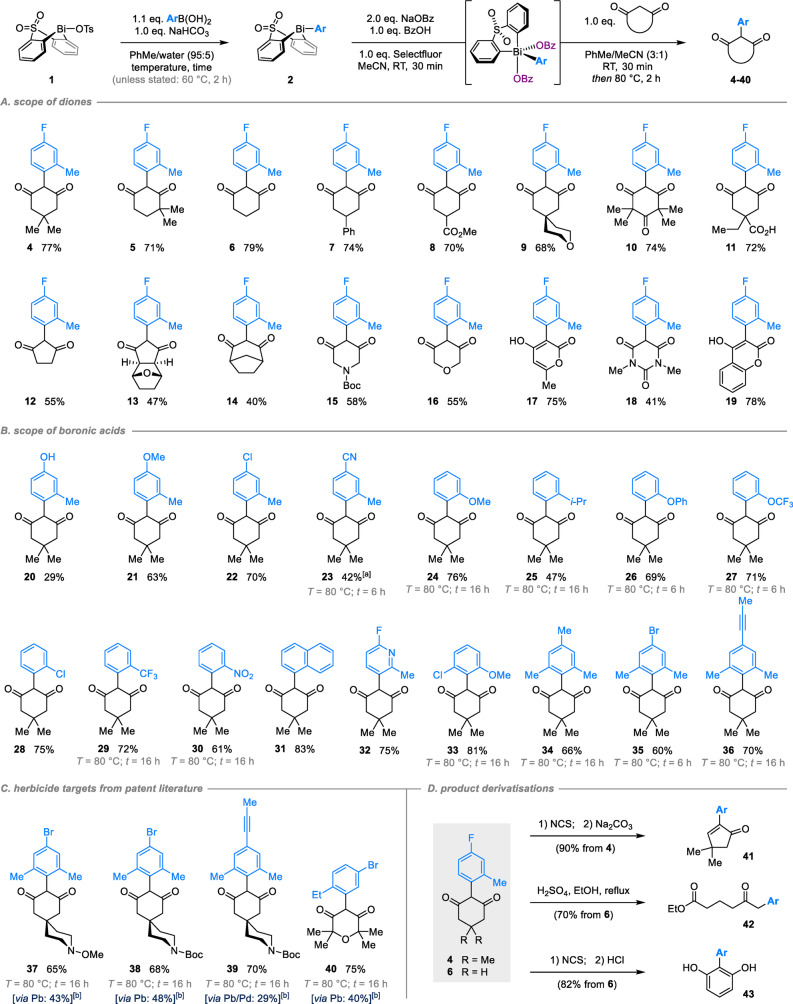
Scope and applications of Bi^V^‐mediated α‐arylation of cyclic 1,3‐diones. a) variation of the dione substrate; b) variation of the boronic acid partner; c) application to synthesis of industrially‐relevant targets; d) synthetic applications of arylated diones. Reactions performed using 0.50 mmol dione; yields refer to material isolated following purification. [a] NaOBz and BzOH added after dimedone. [b] Literature yields achieved via Pinhey arylation.[^53^, ^54^, ^55^, ^56^]

Subsequent studies of reaction scope indicated that our Bi^V^‐mediated arylation methodology can be applied to diverse cyclohexanedione derivatives, affording the expected products in good yield and with complete selectivity towards monoarylation (Scheme 3, **4**–**11**). The methodology can be extended to diones featuring different ring sizes (**12**–**14**) and heterocyclic substructures (**15**–**19**), including those based on pyrone (**17**), barbituric acid (**18**) and hydroxycoumarin (**19**). Its applicability to substrates of direct relevance to agrochemical discovery is clearly illustrated by the diones employed in the synthesis of **9**, **10**, **13** and **16**, which together feature in over 1820 patented compounds.

The reaction is similarly general with respect to the boronic acid component (Scheme 3B), with even very electron‐deficient and sterically‐hindered aryl moieties giving respectable yields if longer times and higher temperatures are employed for B‐to‐Bi transmetallation. Boronic acids featuring *ortho*‐methyl substituents are tolerated, irrespective of their electronic properties (**20**–**23**). Of particular note, the hydroxyarene motif in **20** is a precursor to more than 160 agrochemical candidates that feature an aryloxy substituent at the 4‐position of the aryl ring. Although the yield of **20** is modest, it surpasses that obtained via the multi‐step syntheses employed previously for related motifs (<18 % via Pinhey arylation/*O*‐debenzylation^[44]^). While instability of the Bi^V^ dibenzoate intermediate results in low yield for cyano‐substituted **23** under our standard conditions, an improved yield is achieved by instead adding dimedone *before* NaOBz/BzOH to purposefully form a Bi^V^ ylide prior to product‐forming thermolysis at 80 °C.

Good yields are also obtained for boronic acids featuring *ortho*‐substituents other than methyl (**24**–**31**), with very electron donating (**24**), very electron withdrawing (**29**, **30**) and very sterically demanding (**25**) groups all well tolerated. Notably, *ortho*‐chlorophenyl substituted **28** was previously prepared via a three‐step sequence consisting of S_*N*_Ar, nitro reduction and subsequent diazotisation/deamination.^[57]^ Its facile synthesis in a single telescoped operation from bismacycle tosylate **1** again illustrates the enabling nature of our Bi^V^‐mediated arylation methodology.

While aryl moieties featuring two *ortho* substituents are the most challenging to install by conventional methods, including cross‐coupling, our methodology furnishes the desired products in high yields (**33**–**36**). This scope is especially relevant to agrochemical discovery, given that the aryl groups featured in **33**, **35** and **36** are also present in over 760 herbicidal and pesticidal candidates from 35 patents. To further showcase the utility of our methodology we prepared **37**–**39**, each in a single telescoped operation from the corresponding dione and 2,6‐disubstituted boronic acid (Scheme 3C). These compounds are key herbicidal intermediates from the patent literature which have been prepared previously only by Pinhey arylation. Our strategy achieves higher yield, even without substrate‐specific optimization, and avoids the use of highly toxic lead reagents and mercury catalysts.^[41]^ In addition, mono‐*ortho*‐substituted **40**—a key intermediate required for a Syngenta research project^[34]^—is formed in high yield, significantly improved relative to the original Pinhey arylation approach.

The broader value of our methodology is illustrated by subsequent diversification of the aryl dione products (Scheme 3D). For example, decarboxylative ring contraction of **4** gives cyclopentadienone **41**, a substructure present in known Cox‐2 inhibitors,^[58]^ whereas subjecting **6** to retro‐Claisen condensation gives δ‐keto ester **42**. Finally, aromatization affords 2‐aryl resorcinol **43**, a motif that is common to polyketide natural products, but which is not otherwise directly accessible.^[59]^

**Scheme 4:**
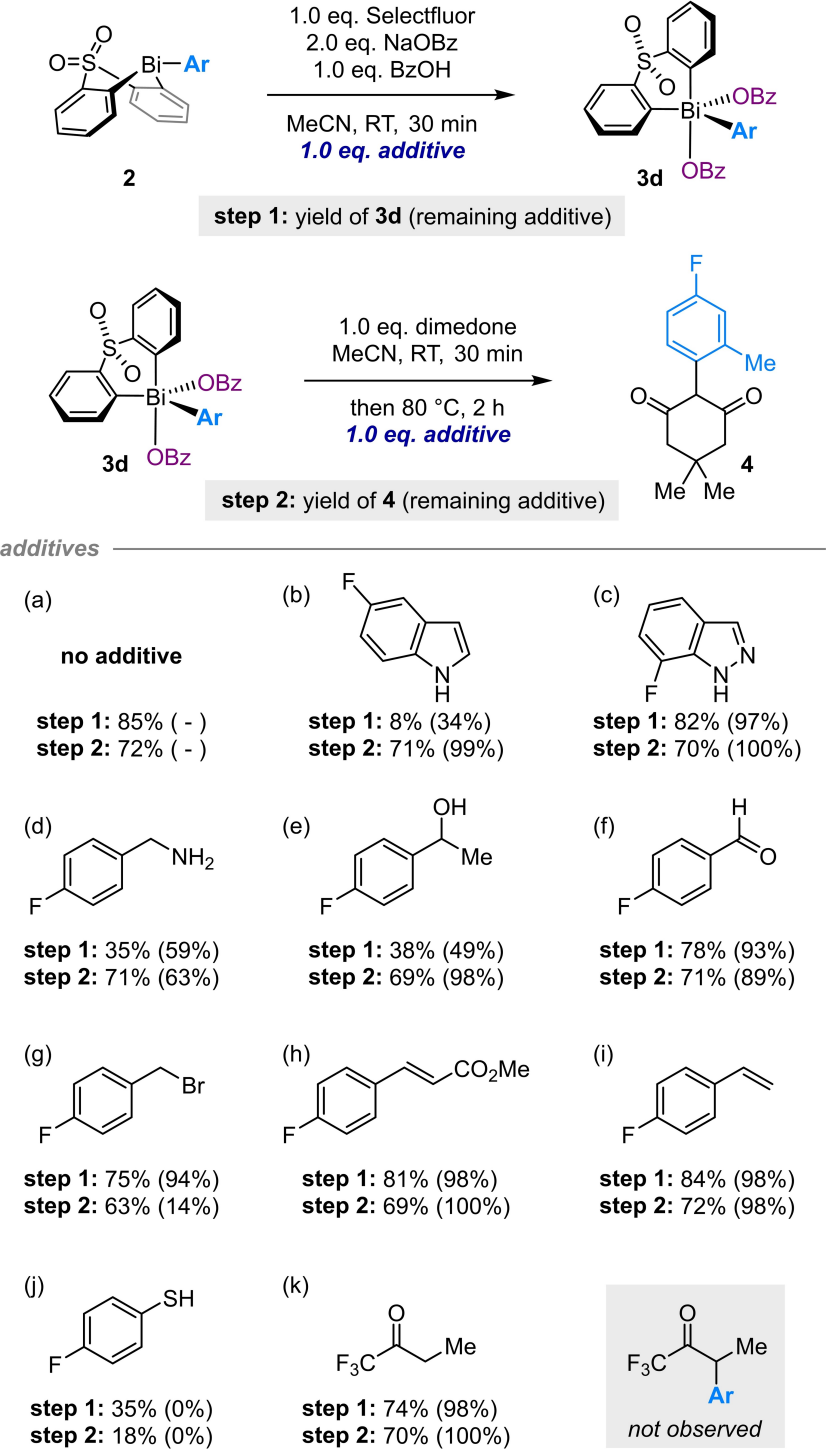
Robustness screen of the oxidation and arylation steps. Yields determined by ^19^F NMR spectroscopic analysis vs. internal standard.

The mild conditions employed in our methodology render it compatible with a broad range of synthetically valuable functionality, including esters (**8**), acids (**11**), nitriles (**23**), 2‐halopyridines (**32**), aryl chlorides (**28**), and aryl bromides (**35**, **37**, **38**, **40**). A further assessment of functional group tolerance was made using a Glorius‐type robustness screen (Scheme 4).[^60^, ^61^] The key oxidation and arylation steps were studied individually in order to probe compatibility with functionality introduced through: (1) the boronic acid, which is present during both oxidation and arylation, and (2) the dione, which is present during only arylation.

Consistent with known reactivity patterns, the addition of an unprotected indole,[^62^, ^63^] benzylamine,^[64]^ benzyl alcohol,^[65]^ or thiophenol[^47^, ^66^] led to a significant decrease in the yield of Bi^V^ dibenzoate **3 d** (step 1; compare entry a with entries b, d, e and j). In contrast, aldehydes, electrophilic alkyl halides, electron‐poor and electron‐rich alkenes, and acidic ketones are well tolerated in the same step (entries f–i, k). Given the propensity of styrenes towards electrophilic fluorination,[^67^, ^68^] the compatibility of this additive with Selectfluor (entry i) reflects the high relative rate with which bismacycle **2** is oxidized.

The subsequent arylation process (step 2) is largely unaffected by any of the additives other than a thiophenol (entry j), which is again consistent with known reactivity.^[47]^ Notably, dimedone is arylated selectively in the presence of an acidic ketone (entry k). These additional studies indicate that, while a broad range of functionality is tolerated in both steps, a greater degree of diversity can be incorporated as part of the dione partner.

We next sought to extend our methodology to fluoroalkyl diones, a class of substrates that has so far resisted arylation by any other method. However, application of the conditions developed for cyclic diones to a trifluoromethyl‐substituted dione afforded the arylation product **44** in only modest yield (Table 1, entry 1). While further optimization of the telescoped process did not significantly improve the reaction outcome (see Supporting Information), the use of isolated arylbismacycle **2** resulted in a significantly improved yield (entry 2). Subsequent investigations indicated that reaction of the trifluoromethyl dione with Bi^V^ dimer **3 b** does not form an observable ylide (cf. **3 c**, Scheme 2B), but instead gives the expected arylation product **44**. The reaction conditions could therefore be simplified (Table 1, entry 3), with reproducibly high yields of the arylated dione **44** being obtained in the absence of additives or co‐solvents. As for cyclic diones, O‐arylation was not observed under any of the conditions investigated.

**Table 1:** Optimization of Bi^V^‐mediated α‐arylation of fluoroalkyl 1,3‐diones.

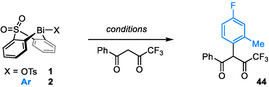		
Entry	conditions	Yield [%]^[a]^
1	as Scheme 3, from [Bi]‐OTs **1**	30
2	as Scheme 3, from [Bi]‐Ar **2**	75
3	from [Bi]‐Ar **2**: 1) 1.0 eq. Selectfluor, MeCN, RT, 15 min 2) 1.0 eq. dione, 80 °C, 2 h	76

[a] Yields determined by ^19^F NMR spectroscopic analysis vs. internal standard.

**Scheme 5:**
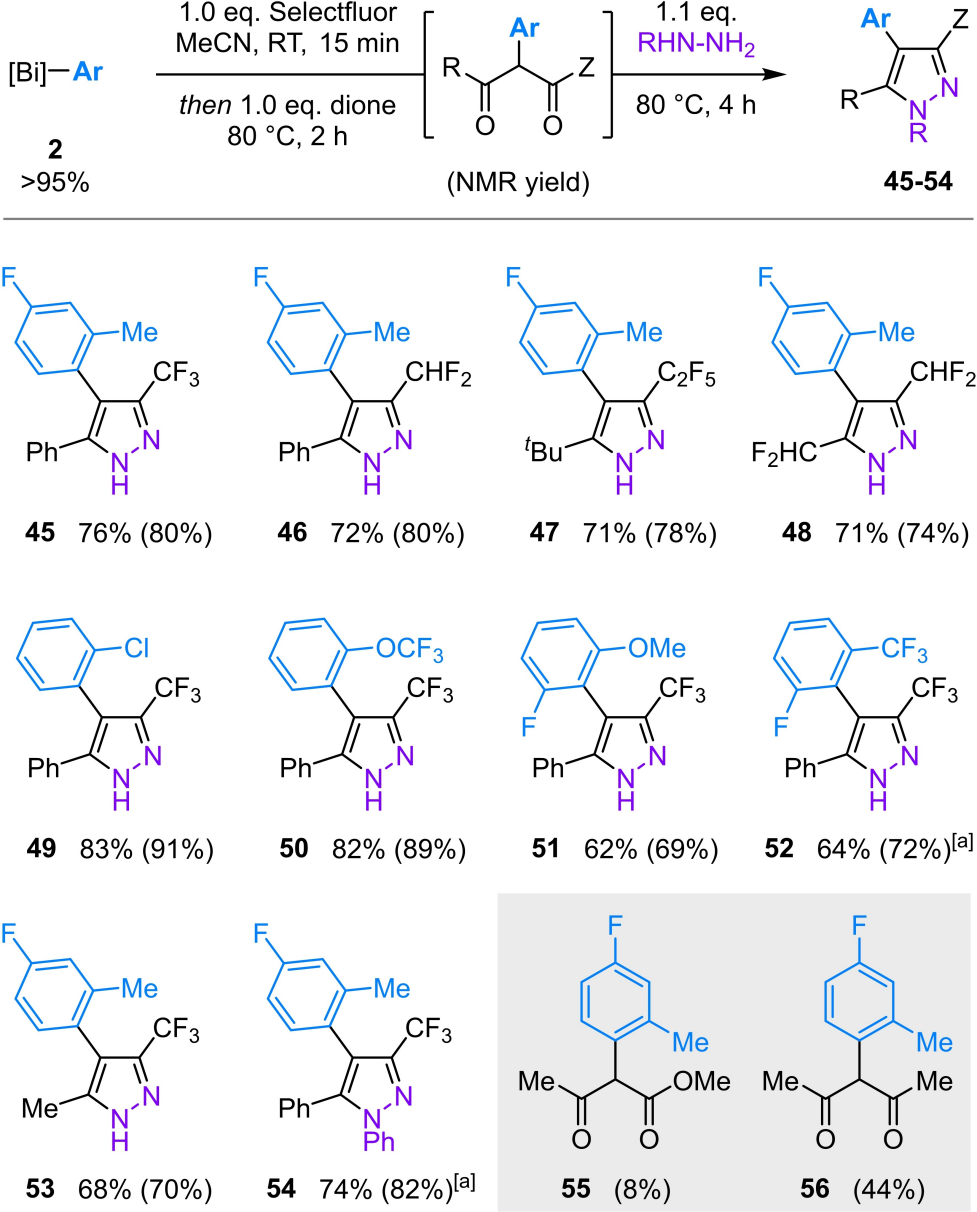
Scope of Bi^V^‐mediated α‐arylation of fluoroalkyl 1,3‐diones. Reactions performed using 0.50 mmol dione. Yields refer to pyrazoles isolated following purification; values in parentheses refer to yields of intermediate aryl diones determined by ^19^F NMR spectroscopic analysis vs. internal standard. [a] Pyrazole formation performed with 1.5 eq. hydrazine for 16 h at 80 °C.

Isolation of the arylation products proved challenging due to their propensity to undergo retro‐Claisen condensation. However, addition of hydrazine directly to the crude reaction mixture allowed near‐quantitative conversion to the corresponding pyrazole, which could then be isolated conveniently (Scheme 5). In this way, it is now possible to convert a fluoroalkyl dione to the corresponding, industrially valuable arylated pyrazole in a straightforward, one‐pot process.

The arylation is compatible with diones bearing both perfluoroalkyl (Scheme 5, **45**, **47**, **53**) and difluoromethyl (**46**, **48**) substituents, and is uniquely effective for acidic diones (cf. **55**, **56**). As anticipated, condensation of the intermediate arylated diones with phenyl hydrazine gives the corresponding *N*‐substituted pyrazole as a single regioisomer (**54**).[^69^, ^70^] The fluoroalkyl pyrazoles that are accessible using this methodology feature in at least 17 commercial agrochemicals and 7 pharmaceuticals,^[23]^ where the difluoromethyl substituent is of particular importance as a lipophilic bioisostere of hydroxyl, thiol and amine moieties.^[71]^ Similarly to cyclic diones, both electron‐withdrawing (**49**, **50**, **52**) and electron‐donating (**51**) *ortho* substituents are well‐tolerated on the arylating agent.

Finally, application of leading Pd‐ and Cu‐catalyzed cross‐coupling methods[^11^, ^12^, ^14^] to the synthesis of **4**, **34** and **44** allowed for a head‐to‐head comparison with our Bi^V^‐mediated arylation strategy (see Supporting Information). The universally poor performance of the catalytic approaches (Cu: 0–13 %; Pd: 0 %; vs. Bi: 60–82 %) underlines the challenging nature of couplings between fluoroalkyl or cyclic diones and *ortho*‐substituted aryl partners, and further illustrates the utility of our methodology.

## Conclusion

In summary, by exploiting insight into the reactivity of key intermediates, we have developed a concise and general method for the Bi^V^‐mediated coupling of cyclic and fluoroalkyl 1,3‐diketones with *ortho*‐substituted aryl partners. Our methodology employs a bench‐stable Bi^III^ precursor and readily available arylboronic acids, and is tolerant of both synthetically‐versatile and sterically‐demanding functionality. Its application to cyclic diones enables the straightforward synthesis of a motif that is privileged in ACCase herbicides and pesticides, and which was previously only directly accessible via stoichiometric chemistries based on neurotoxic Pb and Hg. In contrast, application of our protocol to fluoroalkyl diones represents the first method for arylation of this compound class; subsequent condensation with hydrazine provides facile access to pharmaceutically and agrochemically valuable fluorinated pyrazoles. More broadly, our methodology provides a clear demonstration of how relatively underexplored reactivity manifolds—such as ligand coupling at high‐valent main group elements—can be used to address the limitations inherent to conventional synthesis strategies.

## Conflict of interest

The authors declare no conflict of interest.

1

## Supporting information

As a service to our authors and readers, this journal provides supporting information supplied by the authors. Such materials are peer reviewed and may be re‐organized for online delivery, but are not copy‐edited or typeset. Technical support issues arising from supporting information (other than missing files) should be addressed to the authors.

Supporting Information

Supporting Information

## Data Availability

The data that support the findings of this study are available in the supplementary material of this article.
