# Supplementary material for: Bismuth‐Mediated α‐Arylation of Acidic Diketones with ortho‐Substituted Boronic Acids
Source: Angew Chem Int Ed Engl. 2022 Aug 29;61(40):e202210840. doi: 10.1002/anie.202210840 (PMC9805042; doi:10.1002/anie.202210840)

Angewandte Chemie
Eine Zeitschrift der Gesellschaft Deutscher Chemiker

Supporting Information

Bismuth-Mediated α-Arylation of Acidic Diketones with *ortho*-Substituted Boronic Acids

*K. Ruffell, S. P. Argent, K. B. Ling, L. T. Ball**

Supporting Information

Bismuth-Mediated α-Arylation of Acidic Diketones with *ortho*-Substituted Boronic Acids

Katie Ruffell,[†] Stephen P. Argent,[†] Kenneth B. Ling[‡] and Liam T. Ball*[,†]

[†] School of Chemistry, University of Nottingham, Nottingham NG7 2RD, U.K.

[‡] Syngenta, Jealott's Hill International Research Centre, Bracknell, Berkshire RG42 6EY, U.K.

liam.ball@nottingham.ac.uk ● orcid.org/0000-0003-3849-9006

Table of Contents

1. General Information 2

2. Starting Material Synthesis 4

i. Organobismuth(III) Compounds 4

ii. Substrates 13

3. Characterisation of Bi(V) Species 16

4. Optimisation 21

i. Arylation of Cyclic 1,3-Diketones: mCPBA as Oxidant 21

ii. Arylation of Cyclic 1,3-Diketones: Selectfluor as Oxidant 27

a. Transmetallation and Arylation in MeCN 34

b. C- vs O-Selectivity of Arylation 35

iii. Arylation of Fluoroalkyl 1,3-Diketones 39

5. General Procedures 41

6. Characterisation Data for Arylation Products 44

7. Derivatisations 93

8. Robustness Screen 96

9. Comparison to Pd- and Cu-Catalysis 100

i. Procedure for Pd-Catalysed Arylation 102

ii. Procedure for Cu-Catalysed Arylation 103

iii. Synthesis of 2-(ortho-Tolyl)-1,3-cyclohexanedione (S6) 104

10. X-Ray Diffraction Data Tables 106

11. References 107

12. NMR Spectra 108

1. General Information

Procedures employing oxygen- and/or moisture-sensitive materials were performed with anhydrous solvents (*vide infra*) using standard inert-atmosphere techniques (atmosphere of anhydrous dinitrogen). Analytical thin-layer chromatography was performed on precoated aluminium-backed plates (Silica Gel 60 F254; Merck) and visualized using a combination of UV light (254 nm) and aqueous basic potassium permanganate stains. Manual flash column chromatography was performed using Scharlab 60 silica gel (35-70 mesh); automated flash column chromatography was performed on disposable columns pre-packed with 50 μm spherical silica gel using a Büchi C-850 equipped with a UV-vis DAD (200-800 nm) and an ELSD. Preparative HPLC was performed on a C18 column (100 Å, 10 μm, 250 × 30 mm) using a Büchi C-850 equipped with a UV-vis DAD (200-800 nm) and an ELSD.

NMR spectra were recorded at 25 °C on a Bruker Avance 500 or 400 spectrometer (^{1}H, 500 / 400 MHz; $^{13}C\{^{1}H\}$, 125 / 100 MHz; ^{19}F NMR, 471 / 376 MHz). Chemical shifts are reported in ppm; coupling constants, *J*, are reported in Hz and are uncorrected for digitization. The following abbreviations (and their combinations) are used to label the multiplicities: s (singlet), d (doublet), t (triplet), q (quartet), quint (quintet), sept (septet), m (multiplet), br (broad) and app. (apparent). ^{1}H and $^{13}C\{1H\}$ chemical shifts are reported relative to tetramethylsilane, and are referenced to the appropriate residual solvent peaks:

- $CDCl_3$: δ_H = 7.26 ppm, δ_C = 77.16 ppm
- CD_3OD: δ_H = 3.31 ppm, δ_C = 49.00 ppm
- CD_3CN: δ_H = 1.94 ppm, δ_C = 118.26 ppm
- DMSO-d_6: δ_H = 2.50 ppm, δ_C = 39.52 ppm

Infrared spectra of neat compounds were recorded over the range 4000-600 cm^{-1} using either a PerkinElmerSpectrum 1000 Series FTIR spectrometer with an ATR diamond cell, or a Bruker Alpha FTIR spectrometer fitted with a Bruker Platinum ATR Quicksnap™ diamond cell. Melting points were measured using Stuart SMP10 or Gallenkamp melting point apparatus in open capillaries.

High resolution electrospray ionization mass spectra (HRMS) were recorded using a Bruker ESITOF MicroTOF II spectrometer.

X-ray measurements were made on crystals coated in vacuum grease and mounted on a glass needle using Mo-Kα (λ = 0.71073 Å) radiation on SuperNova Atlas diffractometer at 120(2) K.

Using Olex2,[1] the structure was solved with the SHELXT[2] structure solution program using Intrinsic Phasing and refined with the SHELXL[2] refinement package using Least Squares minimisation.

Reagent grade solvents (Fisher Technical) were employed. THF was dried using an Inert PureSolv Grubbs-type system (alumina columns, argon atmosphere). CH_2Cl_2 was distilled from 4Å molecular sieved under an atmosphere of anhydrous dinitrogen. Unless stated otherwise, all reagents were used as received from commercial sources.

2. Starting Material Synthesis

i. Organobismuth(III) Compounds

Tri(4-fluorophenyl)bismuthine (S1)

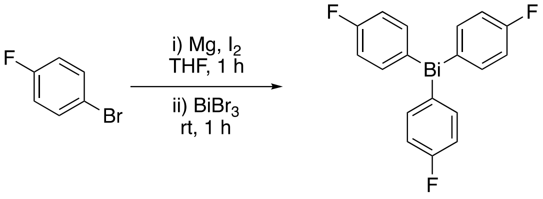

S1

In a flame dried flask under an atmosphere of N_2, magnesium turnings (4.01 g, 165 mmol) and ~3 iodine crystals were heated until a purple vapour developed and stirred for 20 mins. Anhydrous THF (60 mL) was added followed by dropwise addition of 1-bromo-4-fluorobenzene (17.0 mL, 155 mmol) at a rate that maintained reflux. The mixture was then stirred for 1 h, allowing time to cool to room temperature. In a separate flame dried flask under an atmosphere of N_2, a solution of $BiBr_3$ (22.4 g, 50.0 mmol) in anhydrous THF (75 mL) was prepared to which the Grignard reagent was added dropwise. The mixture was stirred for 1 h before quenching with water (100 mL), filtering through a pad of silica gel and extracting with Et_2O (3 × 100 mL). The combined organic portions were dried over $MgSO_4$ and concentrated to dryness. Recrystallisation from hot ethanol afforded the title compound (22.2 g, 44.8 mmol, 90%) as a colourless solid. Characterisation data were consistent with literature values.[3]

^{1}H NMR (500 MHz, $CDCl_3$): δ 7.66 (dd, *J* = 8.4, 6.2 Hz, 6H), 7.09 (app. t, *J* = 9.0 Hz, 6H).
^{13}C{^{1}H} NMR (126 MHz, $CDCl_3$): δ 163.0 (d, *J* = 247.3 Hz), 149.6 (br), 139.3 (d, *J* = 7.0 Hz), 118.1 (d, *J* = 19.7 Hz).
^{19}F NMR (471 MHz, $CDCl_3$): δ -112.67 (tt, *J* = 9.4, 6.1 Hz).
ν_{max} (ATR)/cm^{-1}: 3058, 1571, 1482, 1384, 1212, 1159, 1016, 816, 506.
HRMS calcd. for $C_{18}H_{12}BiF_3$+Na^+: 517.0587 [M+Na]$^+$; found (ESI$^+$) 517.0583.
m.p./°C: 96–97.

5,5-Dioxido-10*H*-dibenzo[*b*,*e*][1,4]thiabismin-10-yl 4-methylbenzenesulfonate (1)

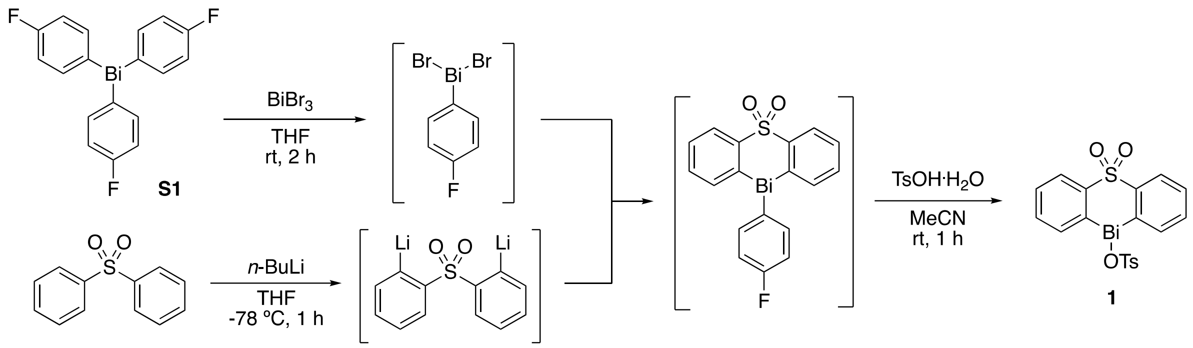

In a flame dried flask under an atmosphere of N_2, a solution of tri(4-fluorophenyl)bismuth (9.49 g, 19.2 mmol) and $BiBr_3$ (17.2 g, 38.4 mmol) in anhydrous THF (100 mL) was stirred at room temperature for 2 h.

In a flame dried flask under an atmosphere of N_2, *n*-butyllithium (2.5 M in hexanes; 48 mL, 120 mmol) was added dropwise to a solution of diphenylsulfone (13.1 g, 60.0 mmol) in anhydrous THF (120 mL) at -78 °C. The resulting solution was stirred at -78 °C for 1 h.

The bromodiarylbismuth solution was added dropwise *via* cannula to the dilithiodiphenylsulfone mixture and the resulting suspension was allowed to warm to room temperature overnight. The mixture was quenched with MeOH (10 mL) and concentrated to dryness then dissolved in CH_2Cl_2 (100 mL) and filtered through a pad of silica gel. The filtrate was stirred with sat. aq. $NaHCO_3$ solution (100 mL) and water (100 mL) for 1 h. The phases were separated and the aqueous portion extracted with CH_2Cl_2 (3 × 100 mL). The combined organic portions were dried over $MgSO_4$, filtered through a pad of silica gel and concentrated to dryness affording a crude pale yellow solid.

p-Toluenesulfonic acid monohydrate (12.1 g, 63.4 mmol) was added to a suspension of the crude aryl bismacycle (29.6 g, 57.6 mmol) in MeCN (80 mL). The resulting suspension was stirred at room temperature for 1 h then cooled to 0 °C. The product was collected by filtration and washed with ice cold MeCN and Et_2O and dried under a flow of air to afford the title compound (22.7 g, 38.1 mmol, 66% over 2 steps) as a colourless solid. Characterisation data were consistent with literature values.[4]

^{1}H NMR (400 MHz, $CDCl_3$): δ 8.93 (dd, *J* = 7.4, 1.1 Hz, 2H), 8.40 (dd, *J* = 7.7, 1.2 Hz, 2H), 7.84 (d, *J* = 8.0 Hz, 2H), 7.79 (app. td, *J* = 7.5, 1.2 Hz, 2H), 7.49 (app. td, *J* = 7.6, 1.1 Hz, 2H), 7.29 (d, *J* = 8.0 Hz, 2H), 2.42 (s, 3H).

$^{13}C\{^{1}H\}$ **NMR (126 MHz, $CDCl_3$):** δ 189.4, 142.6, 139.8, 138.5, 136.4, 135.7, 129.6 (2C), 129.1, 126.6, 21.6.

ν_{max} (ATR)/cm^{-1}: 3051, 1565, 1302, 1223, 1151, 1123, 739, 588, 567.

HRMS calcd. for $C_{19}H_{15}BiO_5S_2$+Na^+: 619.0057 $[M+Na]^+$; found (ESI^+): 619.0020.

m.p./°C: 224–225.

5,5-Dioxido-10H-dibenzo[b,e][1,4]thiabismin-10-yl benzoate

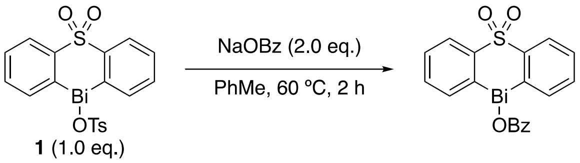

A suspension of bismacycle tosylate **1** (298 mg, 0.500 mmol) and sodium benzoate (144 mg, 1.00 mmol) in toluene (5 mL) was heated at 60 °C for 2 h then cooled to room temperature. Water (30 mL) was added and the mixture extracted with CH_2Cl_2 (3 × 20 mL). The combined organic portions were dried over $MgSO_4$ and concentrated to dryness affording the title compound (264 mg, 0.483 mmol, 97%) as a colourless solid.

^{1}H NMR (400 MHz, $CDCl_3$): δ 8.80 (dd, *J* = 7.4, 1.1 Hz, 2H), 8.37 (dd, *J* = 7.7, 1.2 Hz, 2H), 8.17 – 8.05 (m, 2H), 7.69 (app. td, *J* = 7.4, 1.3 Hz, 2H), 7.61 – 7.51 (m, 1H), 7.50 – 7.41 (m, 4H).

^{13}C NMR (101 MHz, $CDCl_3$): δ 184.8, 174.0, 141.1, 136.2, 135.3, 132.7, 132.3, 130.4, 128.8, 128.7, 128.4.

ν_{max} (ATR)/cm^{-1}: 3053, 1603, 1567, 1449, 1440, 1342, 1302, 1290, 1254, 1175, 1143, 1132, 1111, 1101, 1087, 1069, 1025, 1011, 909, 850, 763, 739, 716, 685, 588, 565, 510, 466.

HRMS calcd. for $C_{19}H_{13}BiO_4S_{+}H^+$: 569.0231 $[M+H]^+$; found (ESI^+) 569.0245.

m.p./°C: 182-183.

General Procedure for Isolation of Arylbismacycles (2)

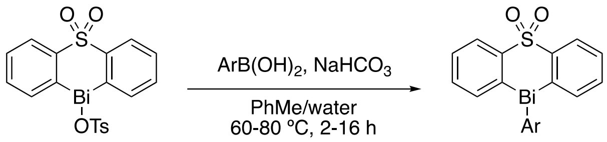

1

A suspension of bismacycle tosylate **1** (1.0 eq.), aryl boronic acid (1.1 eq.) and sodium bicarbonate (1.0 eq.) in toluene (0.1 M) and water (5 vol%) was heated at **X** °C for **Y** h. The mixture was cooled to room temperature before diluting with EtOAc (20 mL) and washing with 2M NaOH (3 × 10 mL). The organic portion was dried over $MgSO_4$ and concentrated to dryness, afforcing the aryl bismacycle **2** as a colourless solid.

10-(4-Fluoro-2-methylphenyl)-10*H*-dibenzo[*b,e*][1,4]thiabismine 5,5-dioxide (2a)

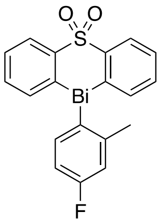

Synthesised according to the above procedure (**X** = 60; **Y** = 2). Using 4-fluoro-2-methylphenylboronic acid (508 mg, 3.30 mmol) afforded the title compound (1.59 g, 2.97 mmol, 99%) as a colourless solid.

^{1}H NMR (500 MHz, $CDCl_3$): δ 8.38 (dd, *J* = 7.8, 1.4 Hz, 2H), 7.85 (dd, *J* = 7.3, 1.3 Hz, 2H), 7.48 (dd, *J* = 8.3, 6.6 Hz, 1H), 7.41 (app. td, *J* = 7.6, 1.3 Hz, 2H), 7.35 (app. td, *J* = 7.3, 1.4 Hz, 2H), 7.09 (dd, *J* = 10.3, 2.7 Hz, 1H), 6.72 (app. td, *J* = 8.5, 2.6 Hz, 1H), 2.54 (s, 3H).

^{13}C{^{1}H} NMR (126 MHz, $CDCl_3$): δ 163.2 (d, *J* = 247.9 Hz), 162.2 (br), 157.9 (br), 147.4 (d, *J* = 6.9 Hz), 143.4 (d, *J* = 7.4 Hz), 141.9, 137.8, 133.6, 128.5, 127.3, 117.6 (d, *J* = 19.9 Hz), 116.8 (d, *J* = 19.7 Hz), 26.6.

^{19}F NMR (471 MHz, $CDCl_3$): δ -112.08 (ddd, *J* = 10.5, 8.8, 6.6 Hz).

ν_{max} (ATR)/cm^{-1}: 3050, 2973, 1566, 1303, 1223, 1151, 1088, 763, 740, 588, 567.

HRMS calcd. For $C_{19}H_{14}BiFO_2S+Na^+$: 557.0395 [M+Na]$^+$; found (ESI$^+$) 557.0392.

m.p./°C: 219–220.

10-(2-Chlorophenyl)-10*H*-dibenzo[*b*,*e*][1,4]thiabismine 5,5-dioxide (2b)

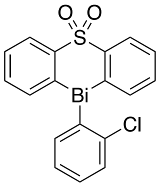

Synthesised according to the above procedure (**X** = 80; **Y** = 2). Using 2-chlorophenylboronic acid (138 mg, 0.880 mmol) afforded the title compound (427 mg, 0.795 mmol, 99%) as a colourless solid.

^{1}H NMR (400 MHz, $CDCl_3$): δ 8.46 – 8.27 (m, 2H), 8.01 – 7.87 (m, 2H), 7.59 (dd, *J* = 8.0, 1.2 Hz, 1H), 7.54 (dd, *J* = 7.5, 1.7 Hz, 1H), 7.46 – 7.34 (m, 4H), 7.30 (ddd, *J* = 8.0, 7.3, 1.7 Hz, 1H), 7.09 (app. td, *J* = 7.4, 1.2 Hz, 1H).

^{13}C{^{1}H} NMR (101 MHz, $CDCl_3$): δ 165.4 (br), 160.8 (br), 141.9, 141.5, 140.9, 137.7, 133.8, 130.4, 130.3, 129.0, 128.4, 127.4.

ν_{max} (ATR)/cm^{-1}: 3050, 1561, 1440, 1422, 1304, 1287, 1252, 1151, 1120, 1087, 1075, 1029, 1010, 909, 740, 718, 705, 588, 567, 514, 462.

HRMS calcd. For $C_{18}H_{12}BiClO_2S+Na^+$: 558.9943 [M+Na]$^+$; found (ESI$^+$) 558.9934.

m.p./°C: 229–230.

10-(2-(Trifluoromethoxy)phenyl)-10*H*-dibenzo[*b*,*e*][1,4]thiabismine 5,5-dioxide (2c)

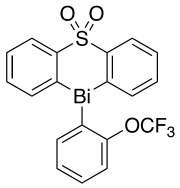

Synthesised according to the above procedure (**X** = 80; **Y** = 16). Using 2-(trifluoromethoxy)phenylboronic acid (181 mg, 0.880 mmol) afforded the title compound (466 mg, 0.795 mmol, 99%) as a colourless solid.

^{1}H NMR (400 MHz, CDCl$_3$): δ 8.38 (dd, *J* = 7.5, 1.5 Hz, 2H), 7.87 (dd, *J* = 7.1, 1.4 Hz, 2H), 7.57 – 7.49 (m, 2H), 7.48 – 7.31 (m, 5H), 7.15 (app. td, *J* = 7.3, 1.1 Hz, 1H).

^{13}C{^{1}H} NMR (101 MHz, CDCl$_3$): δ 159.6 (br), 157.5 (br), 153.7 (q, *J* = 1.6 Hz), 142.0, 141.4, 137.8, 133.8, 130.7, 130.6, 128.5, 127.4, 120.8 (q, *J* = 258.0 Hz), 120.2 (q, *J* = 1.7 Hz).

^{19}F NMR (377 MHz, CDCl$_3$): δ -56.62 (d, *J* = 1.7 Hz).

ν_{max} (ATR)/cm^{-1}: 1564, 1436, 1306, 1286, 1249, 1217, 1184, 1151, 1120, 1108, 1089, 1075, 1013, 920, 763, 740, 718, 704, 588, 567, 514, 462.

HRMS calcd. For $C_{19}H_{12}BiF_3O_3S+Na^+$: 609.0155 [M+Na]$^+$; found (ESI$^+$) 609.0131.

m.p./°C: 179-180.

10-(2-Fluoro-6-methoxyphenyl)-10*H*-dibenzo[*b*,*e*][1,4]thiabismine 5,5-dioxide (2d)

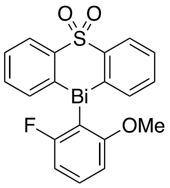

Synthesised according to the above procedure (**X** = 80; **Y** = 16). Using 2-fluoro-6-methoxyphenylboronic acid (150 mg, 0.880 mmol) afforded the title compound (431 mg, 0.783 mmol, 98%) as a colourless solid.

^{1}H NMR (400 MHz, $CDCl_3$): δ 8.38 (dd, *J* = 7.6, 1.5 Hz, 2H), 8.09 (dd, *J* = 7.2, 1.3 Hz, 2H), 7.37 (app. td, *J* = 7.5, 1.3 Hz, 2H), 7.34 – 7.27 (m, 3H), 6.84 (dd, *J* = 8.2, 0.8 Hz, 1H), 6.76 (ddd, *J* = 8.2, 6.3, 0.8 Hz, 1H), 3.70 (s, 3H).

^{13}C{^{1}H} NMR (126 MHz, $CDCl_3$): δ 166.5 (d, *J* = 241.1 Hz), 164.1 (d, *J* = 15.8 Hz), 157.4 (br), 141.4, 138.5 (br d, *J* = 40.2 Hz), 137.9, 133.0, 132.5 (d, *J* = 9.5 Hz), 128.0, 127.1, 109.2 (d, *J* = 27.9 Hz), 106.1 (d, *J* = 2.7 Hz), 55.9.

^{19}F NMR (376 MHz, $CDCl_3$): δ -86.47 (app. t, *J* = 6.6 Hz).

ν_{max} (ATR)/cm^{-1}: 1584, 1573, 1457, 1433, 1302, 1287, 1252, 1224, 1150, 1124, 1109, 1087, 1072, 1013, 910, 763, 740, 718, 702, 588, 567, 512, 463.

HRMS calcd. For $C_{19}H_{14}BiFO_3S+Na^+$: 573.0344 $[M+Na]^+$; found (ESI^+) 573.0352.

m.p./°C: 198-199.

10-(2-Fluoro-6-(trifluoromethyl)phenyl)-10*H*-dibenzo[*b*,*e*][1,4]thiabismine 5,5-dioxide (2e)

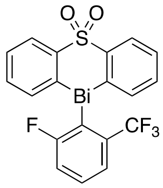

Synthesised according to the above procedure (**X** = 80; **Y** = 16). Using 2-fluoro-6-(trifluoromethyl)phenylboronic acid (183 mg, 0.880 mmol) afforded the title compound (453 mg, 0.770 mmol, 96%) as a colourless solid.

^{1}H NMR (500 MHz, $CDCl_3$): δ 8.43 (dd, *J* = 7.7, 1.4 Hz, 2H), 8.06 (dd, *J* = 7.2, 1.3 Hz, 2H), 7.68 (dd, *J* = 7.8, 1.0 Hz, 1H), 7.48 – 7.40 (m, 3H), 7.37 (app. td, *J* = 7.3, 1.5 Hz, 2H), 7.17 (app. t, *J* = 7.5 Hz, 1H).

^{13}C{^{1}H} NMR (101 MHz, $CDCl_3$): δ 166.4 (d, *J* = 243.8 Hz), 160.7 (br), 149.0 (br d, *J* = 41.6 Hz), 141.0, 138.1 (qd, *J* = 30.3, 11.5 Hz), 137.3 (d, *J* = 1.3 Hz), 133.7, 131.6 (d, *J* = 8.4 Hz), 128.5, 127.5, 124.8 (qd, *J* = 274.8, 1.8 Hz), 122.2 (qd, *J* = 5.2, 2.9 Hz), 119.7 (d, *J* = 29.1 Hz).

^{19}F NMR (377 MHz, $CDCl_3$): δ -56.66 (s, 3F), -77.08 (app. t, *J* = 6.3 Hz, 1F).

ν_{max} (ATR)/cm^{-1}: 1563, 1426, 1315, 1303, 1287, 1232, 1184, 1164, 1150, 1113, 1087, 1073, 892, 801, 764, 739, 717, 586, 566.

HRMS calcd. For $C_{19}H_{11}BiF_4O_2S+Na^+$: 611.0112 $[M+Na]^+$; found (ESI^+) 611.0091.

m.p./°C: 217-218.

ii. Substrates

1-Ethyl-3,5-dioxocyclohexane-1-carboxylic acid (S2)

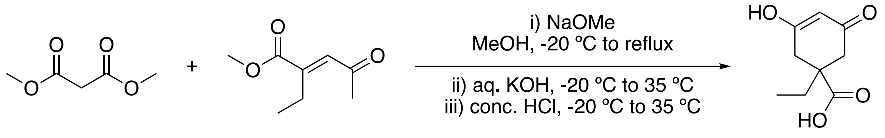

Dimethyl malonate (4.49 g, 33.3 mmol) was added to a solution of sodium methoxide (6.85 g, 31.7 mmol) in MeOH (35 mL) at -20 °C. After warming to room temperature, methyl (*E*)-2-ethyl-4-oxopent-2-enoate (4.95 g, 31.7 mmol) was added and the reaction mixture was heated at reflux for 1.5 h before cooling to -20 °C. A solution of potassium hydroxide (7.39 g, 112 mmol) in water (5 mL) was added dropwise before heating at 35 °C for 72 h and then concentrating to dryness. Water (10 mL) was added and the mixture cooled to -20 °C before conc. HCl (19.5 mL) was added dropwise. The reaction mixture was stirred at room temperature for 2 h before heating at 35 °C for a further 2 h. Water was removed by freeze drying and then MeOH (25 mL) was added and the mixture filtered and washed with MeOH (3 × 10 mL). The filtrate was concentrated to dryness and purified by reverse phase column chromatography to afford the title compound (2.54 g, 13.8 mmol, 44%) as a beige solid.

^{1}H NMR (400 MHz, DMSO-*d*$_6$): δ 12.53 (br s, 1H), 11.06 (br s, 1H)*, 5.13 (s, 1H), 2.59 (d, *J* = 16.5 Hz, 2H), 2.26 (d, *J* = 16.5 Hz, 2H), 1.60 (q, *J* = 7.5 Hz, 2H), 0.80 (t, *J* = 7.5 Hz, 3H).
^{13}C{^{1}H} NMR (126 MHz, DMSO-*d*$_6$): δ 185.7 (br)*, 176.2, 103.3, 47.1, 40.4 (br)*, 31.1, 8.6.
ν_{max} (ATR)/cm^{-1}: 2971, 2937, 1702, 1568, 1399, 1345, 1293, 1230, 1205, 1150, 969, 922, 857, 840, 779, 598, 523.
HRMS calcd. for $C_9H_{12}O_4$-H^+: 183.0663 [M-H]$^-$; found (ESI$^-$) 183.0062.
m.p./°C: 182-183.
* Signal very broad and just visible in the baseline.

(3a*R*,4*S*,7*R*,7a*S*)-Hexahydro-1*H*-4,7-epoxyindene-1,3(2*H*)-dione (S3)

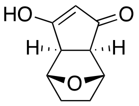

Prepared according to literature procedure.[5]

^{1}H NMR (400 MHz, DMSO-*d*$_6$): δ 12.14 (br s, 1H), 5.10 (s, 1H), 4.48 – 4.35 (m, 2H), 2.61 (s, 2H), 1.69 – 1.43 (m, 4H).

^{13}C{^{1}H} NMR (126 MHz, DMSO-*d*$_6$): δ 196.1 (br)*, 107.9, 76.3, 52.4 (br), 28.4.

ν_{max} (ATR)/cm^{-1}: 2989, 2955, 1658, 1568, 1416, 1301, 1276, 1233, 1178, 999, 926, 816, 605, 496, 464.

HRMS calcd. for $C_9H_{10}O_3$-H^+: 165.0557 [M-H]$^-$; found (ESI$^-$) 165.0559.

m.p./°C: 215-216.

* Signal very broad and just visible in the baseline.

3-Methoxy-3-azaspiro[5.5]undecane-8,10-dione (S4)

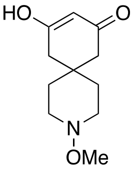

Prepared according to literature procedure.[6]

^{1}H NMR (500 MHz, DMSO-*d*$_6$): δ 11.02 (s, 1H), 5.17 (s, 1H), 3.38 (s, 3H), 3.05 (br s, 2H), 2.44 (br s, 2H), 2.30 (br s, 2H), 2.13 (br s, 2H), 1.64 (br s, 2H), 1.39 (br s, 2H).

^{13}C{^{1}H} NMR (126 MHz, DMSO-*d*$_6$): δ 196.6 (br)*, 175.8 (br)*, 102.6, 58.4, 50.4, 49.4 (br)*, 43.6 (br)*, 42.1 (br)*, 36.4 (br)*, 34.2, 32.5.

ν_{max} (ATR)/cm^{-1}: 2931, 2843, 1596, 1482, 1464, 1433, 1409, 1354, 1316, 1295, 1266, 1224, 1182, 1144, 1090, 1051, 1028, 993, 832, 460.

HRMS calcd. for $C_{11}H_{17}NO_3$-H^+: 210.1136 [M-H]$^-$; found (ESI$^-$) 210.1135.

m.p./°C: 165-166.

* Signal very broad and just visible in the baseline.

(2,6-Dimethyl-4-(prop-1-yn-1-yl)phenyl)boronic acid (S5)

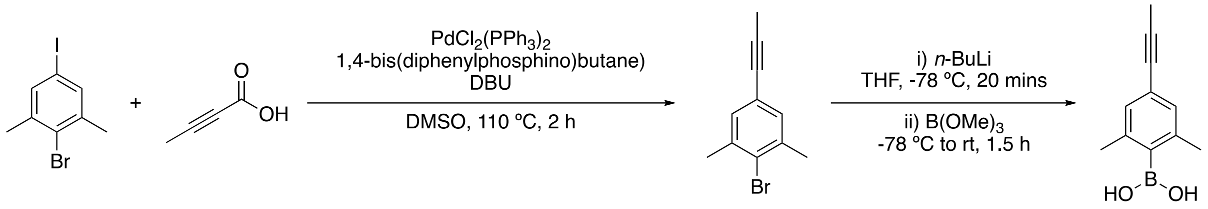

S5

DBU (1.44 mL, 9.65 mmol) was added to a mixture of $PdCl_2(PPh_3)_2$ (114 mg, 0.161 mmol) and 1,4-bis(diphenylphosphino)butane (137 mg, 0.322 mmol) in DMSO (30 mL). The reaction mixture was heated at 110 ºC for 2 h, before cooling to room temperature, diluting with 2M HCl (100 mL) and extracting with EtOAc (2 × 50 mL). The combined organic portions were washed with water, dried over $MgSO_4$ and concentrated to dryness. Purification by silica gel column chromatography (100% isohexane) afforded the 2-bromo-1,3-dimethyl-5-(prop-1-yn-1-yl)benzene (467 mg, 2.09 mmol, 65%) as a colourless oil.

In a flask under an atmosphere of N_2, *n*-butyllithium (2.5 M in hexanes; 19.7 mL, 49.3 mmol) was added dropwise to a solution of 2-bromo-1,3-dimethyl-5-(prop-1-yn-1-yl)benzene (10.0 g, 44.8 mmol) in anhydrous THF (100 mL) at -78 °C. After stirring at -78 °C for 20 mins, trimethyl borate (10 mL, 89.6 mmol) was added dropwise and stirred at -78 °C for 40 mins. The reaction mixture was allowed to warm to room temperature over 1.5 h before quenching with 2M HCl (50 mL) and stirring for 1 h. The mixture was extracted with EtOAc (2 × 100 mL) and the combined organic portions were washed with water, dried over Na_2SO_4 and concentrated to dryness. Isohexane (50 mL) was added to the crude and stirred for 30 mins. The precipitate was collected by filtration, washed with isohexane and dried under a flow of air to afford the title compound (6.29 g, 33.4 mmol, 75%) as a colourless solid.

^{1}H NMR (400 MHz, DMSO-*d*$_6$): δ 8.17 (s, 2H), 6.95 (s, 2H), 2.22 (s, 6H), 2.00 (s, 3H).
^{13}C{^{1}H} NMR (126 MHz, DMSO-*d*$_6$): δ 139.3 (br), 138.8, 128.3, 122.2, 85.4, 80.2, 21.7, 3.9.
ν_{max} (ATR)/cm^{-1}: 3313 (br), 2917, 1602, 1541, 1436, 1329, 1232, 1185, 1098, 1029, 870, 738, 679.
HRMS calcd. for $C_{11}H_{13}BO_2+Na^+$: 211.0901 [M+Na]$^+$; found (ESI$^+$) 211.0900.
m.p./°C: 99-100.

3. Characterisation of Bi(V) Species

4',10-Bis(4-fluoro-2-methylphenyl)-4'*H*,10*H*-4'λ^5,10λ^5-dispiro[dibenzo[*b,e*][1,4]thiabismine-10,2'-[1,3,2,4]fluoraoxadibismetane-4',10''-dibenzo[*b,e*][1,4]thiabismin]-1'-ium 5,5,5'',5''-tetraoxide tetrafluoroborate (3b)

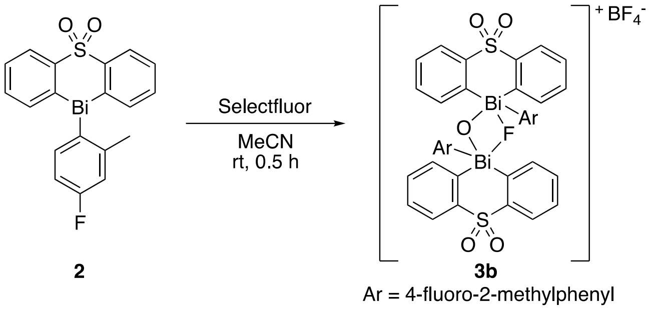

Selectfluor (177 mg, 0.500 mmol) was added to a solution of aryl bismacycle **2** (267 mg, 0.500 mmol) in MeCN (2.5 mL) and stirred at room temperature for 0.5 h. Water (20 mL) was added and the reaction mixture extracted with CH_2Cl_2 (3 × 20 mL). The combined organic portions were dried over $MgSO_4$ and concentrated to dryness affording the title compound (297 mg, 0.249 mmol, quant.) as a yellow solid. Crystals suitable for X-ray diffractometry were grown by slow evaporation of a CH_2Cl_2/toluene solution of **3b**. See Section S10 for XRD data tables.

^{1}H NMR (500 MHz, CD_3CN): δ 8.32 (dd, *J* = 7.7, 1.5 Hz, 2H), 8.19 (br app. s, 2H), 7.91 – 7.76 (m, 3H), 7.73 (app. td, *J* = 7.6, 1.1 Hz, 2H), 7.43 (ddd, *J* = 9.8, 2.6, 1.0 Hz, 1H), 7.19 (app. td, *J* = 8.5, 3.0 Hz, 1H), 2.65 (s, 3H).

^{13}C{^{1}H} NMR (126 MHz, CD_3CN): δ 165.6 (d, *J* = 252.5 Hz), 164.7 (br), 156.2, 146.2, 145.6 (d, *J* = 8.9 Hz), 137.1, 136.5, 136.3 (d, *J* = 9.4 Hz), 133.2, 130.1, 121.3 (d, *J* = 23.0 Hz), 117.5 (d, *J* = 22.9 Hz), 24.1 (d, *J* = 1.4 Hz).

^{19}F NMR (376 MHz, CD_3CN): δ -43.04 (1F), -106.74 (app. td, *J* = 8.8, 5.3 Hz, 2F), -151.73 and -151.78 (4F; $^{10}BF_4$ and $^{11}BF_4$).

ν_{max} (ATR)/cm^{-1}: 3086, 1565, 1468, 1438, 1321, 1233, 1149, 1052, 997, 740, 711, 643, 625, 583, 562, 506, 460.

HRMS calcd. For $C_{38}H_{28}Bi_2F_3O_5S_2^+$: 1103.0933 [M]$^+$; found (ESI$^+$) 1103.0958.

m.p./°C: 136-137.

Fragments observed by mass spectrometry:

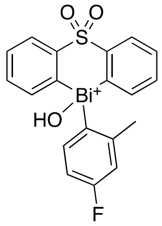

HRMS calcd. For $C_{19}H_{15}BiFO_3S^+$: 551.0524 $[M]^+$; found (ESI^+) 551.0545.

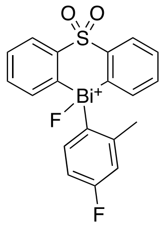

HRMS calcd. For $C_{19}H_{14}BiF_2O_2S^+$: 554.0559 $[M]^+$; found (ESI^+) 554.0528.

Isotopic labelling studies to identify the origin of the oxo bridge in Bi(V) dimer (3b)

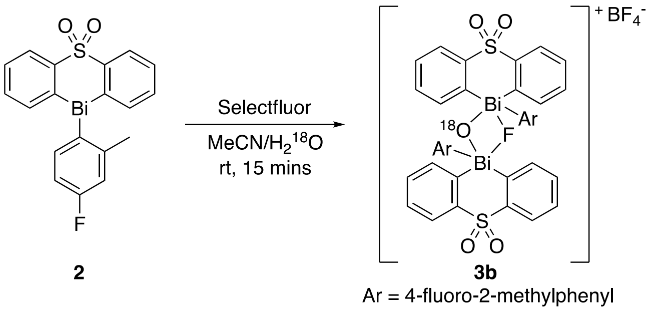

Aryl bismacycle **2** (10.7 mg, 0.020 mmol) and Selectfluor (7.09 mg, 0.020 mmol) were charged to an oven dried vial followed by $H_2{}^{18}O$ (5 drops) and anhydrous MeCN (0.2 mL). The solution was stirred for 15 mins before sampling for direct injection mass spectrometry (see Supplementary Figure 1; note: rapid exchange of the isotopic label with residual $H_2{}^{16}O$ leads to incomplete ^{18}O incorporation).

HRMS calcd. For $C_{38}H_{28}Bi_2F_3O_4{}^{18}OS_2{}^+$: 1105.0975 $[M]^+$; found (ESI^+) 1105.0933.

Ionisation Mode ESI Positive Instrument Bruker MicroTOF

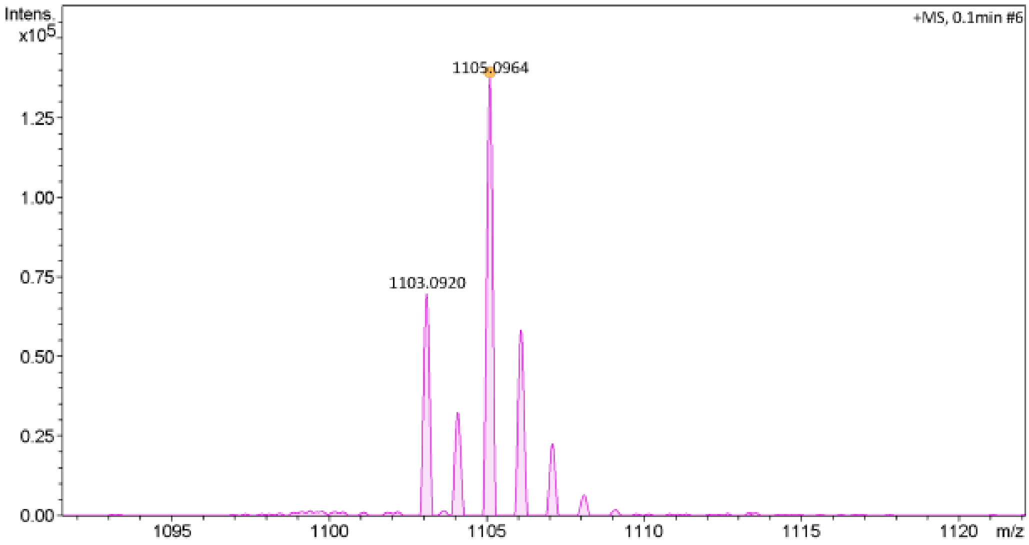

Meas. m/z	#	Ion Formula	Score	m/z	err [mDa]	err [ppm]	mSigma
1105.0964	1	C38H28Bi2F3O4S2^18O	100.00	1105.0975	1.1	1.0	11.7

Note: Sigma fits < 0.05 indicates high probability of correct MF

Ionisation Mode ESI Positive Instrument Bruker MicroTOF

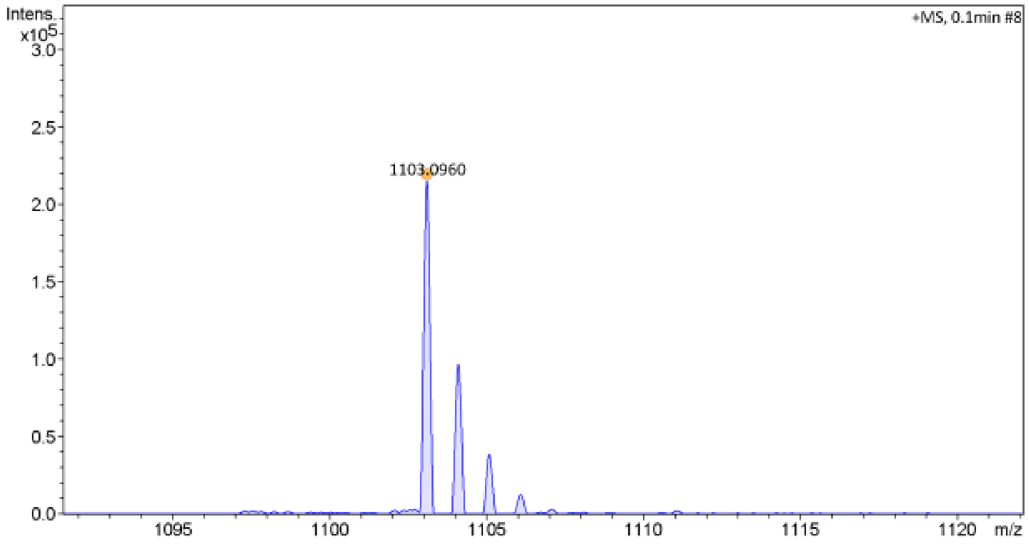

Meas. m/z	#	Ion Formula	Score	m/z	err [mDa]	err [ppm]	mSigma
1103.0960	1	C38H28Bi2F3O5S2	100.00	1103.0933	-2.7	-2.5	11.3

Note: Sigma fits < 0.05 indicates high probability of correct MF

Supplementary Figure 1. ESI mass spectrometry data from oxidation of aryl bismacycle **2** in the presence of $H_2{}^{18}O$ (top) and $H_2{}^{16}O$ (bottom).

1-(10-(4-Fluoro-2-methylphenyl)-5,5-dioxido-10*H*-dibenzo[*b*,*e*][1,4]thiabismin-10-ium-10-yl)-4,4-dimethyl-2,6-dioxocyclohexan-1-ide (3c)

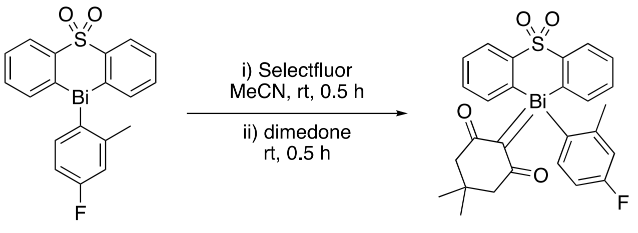

Selectfluor (106 mg, 0.300 mmol) was added to a solution of aryl bismacycle **2** (160 mg, 0.300 mmol) in MeCN (3 mL) and stirred at room temperature for 0.5 h. Dimedone (42.1 mg, 0.300 mmol) was added and the mixture was stirred for 0.5 h before diluting with water (20 mL) and extracting with CH_2Cl_2 (3 × 20 mL). The combined organic portions were dried over $MgSO_4$ and concentrated to dryness affording the title compound (202 mg, 0.300 mmol, quant.) as a yellow solid.

^{1}H NMR (500 MHz, CD_3CN): δ 8.47 (br d, *J* = 7.5 Hz, 2H), 8.36 (dd, *J* = 7.7, 1.5 Hz, 2H), 7.75 (app. td, *J* = 7.5, 1.5 Hz, 2H), 7.68 (app. td, *J* = 7.7, 1.2 Hz, 2H), 7.61 (dd, *J* = 8.6, 5.9 Hz, 1H), 7.27 – 7.19 (m, 1H), 7.08 (app. td, *J* = 8.6, 2.8 Hz, 1H), 2.42 (s, 4H), 2.23 (s, 3H), 1.10 (s, 6H).

^{1}H NMR (500 MHz, $CDCl_3$): δ 8.86 (d, *J* = 7.7 Hz, 2H), 8.38 (dd, *J* = 7.7, 1.5 Hz, 2H), 7.72 (td, *J* = 7.5, 1.4 Hz, 2H), 7.68 (dd, *J* = 8.6, 5.7 Hz, 1H), 7.62 (td, *J* = 7.7, 1.2 Hz, 2H), 7.07 (dd, *J* = 9.6, 2.8 Hz, 1H), 7.00 (td, *J* = 8.4, 2.8 Hz, 1H), 2.50 (s, 4H), 2.42 (s, 3H), 1.13 (s, 6H).

^{13}C{^{1}H} NMR (126 MHz, CD_3CN): δ 190.4, 165.2 (d, *J* = 249.2 Hz), 147.3 (d, *J* = 8.4 Hz), 145.6, 145.3, 142.9, 138.7, 138.6 (d, *J* = 9.2 Hz), 136.1, 132.5, 128.4, 119.6 (d, *J* = 21.8 Hz), 118.2*, 116.2 (d, *J* = 21.8 Hz), 52.6, 33.9, 28.6, 24.3 (d, *J* = 1.7 Hz).

^{13}C{^{1}H} NMR (126 MHz, $CDCl_3$): δ 191.1, 164.2 (d, *J* = 251.3 Hz), 144.9 (d, *J* = 8.1 Hz), 144.7 (2C), 144.4, 138.7, 137.0 (d, *J* = 8.7 Hz), 135.2, 131.4, 128.0, 119.1 (d, *J* = 21.8 Hz), 118.3, 115.9 (d, *J* = 21.5 Hz), 52.3, 33.5, 28.7, 24.7.

^{19}F NMR (376 MHz, CD_3CN): δ -110.27 – -110.81 (m).

^{19}F NMR (376 MHz, $CDCl_3$): δ -107.93 – -109.46 (m).

ν_{max} (ATR)/cm^{-1}: 3056, 2953, 2928, 2867, 1592, 1516, 1469, 1432, 1320, 1229, 1156, 1134, 1082, 1009, 765, 740, 714, 584, 564, 511, 455, 423.

HRMS calcd. For $C_{27}H_{24}BiFO_4S+H^+$: 673.1256 $[M+H]^+$; found (ESI^+) 673.1265.

m.p./°C: 129-131.

* Signal obscured by CD_3CN signal, observed by HMBC.

10-(4-Fluoro-2-methylphenyl)-5,5-dioxido-10*H*-10λ[5]-dibenzo[*b*,*e*][1,4]thiabismine-10,10-diyl dibenzoate (3d)

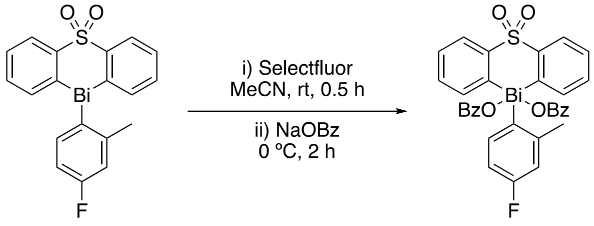

2 **3d**

Selectfluor (42.5 mg, 0.120 mmol) was added to a solution of aryl bismacycle **2** (64.1 mg, 0.120 mmol) in CD_3CN (2.4 mL) and stirred at room temperature for 0.5 h. The solution was cooled to 0 °C and sodium benzoate (34.6 mg, 0.240 mmol) was added. After stirring at 0 °C for 2 h the title compound was characterised *in situ* (due to the instability of the compound, isolation and full characterisation was not possible).

^{1}H NMR (500 MHz, CD_3CN): δ 8.76 – 8.62 (m, 2H), 8.57 – 8.42 (m, 2H), 8.30 – 8.17 (m, 1H), 7.96 (app. t, *J* = 7.8 Hz, 2H), 7.90 – 7.78 (m, 4H), 7.73 (app. t, *J* = 7.7 Hz, 2H), 7.56 – 7.46 (m, 2H), 7.45 – 7.34 (m, 4H), 7.34 – 7.30 (m, 1H), 7.30 – 7.20 (m, 1H), 2.93 (br s, 3H).
^{19}F NMR (376 MHz, CD_3CN): δ -109.7 – -109.9 (m).
HRMS calcd. For $C_{33}H_{24}BiFO_6S+Na^+$: 799.0974 $[M+Na]^+$; found (ESI^+) 799.0969.

4. Optimisation

i. Arylation of Cyclic 1,3-Diketones: *m*CPBA as Oxidant

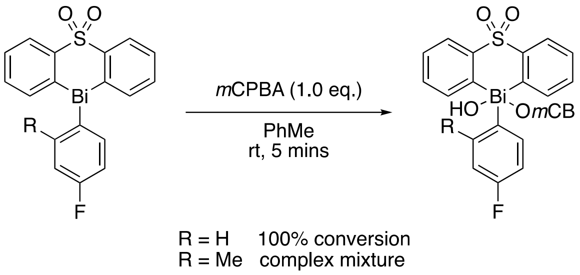

Supplementary Figure 2. Impact of *ortho*-substituent on the stability of the Bi(V) species resulting from oxidation with *m*CPBA.

Procedure: A solution of the aryl bismacycle (0.020 mmol) in toluene (0.5 mL) was prepared in an NMR tube. *m*CPBA (89% purity; 3.88 mg, 0.020 mmol) was added and the solution mixed for 5 mins before analysing by ^{19}F NMR spectroscopy.

Commentary: When no *ortho*-substituent is present on the aryl group (R = H), 100% conversion to a Bi(V) species is observed[4] but, in the presence of an *ortho*-substituted aryl group (R = Me), a Bi(V) species could not be observed and instead afforded a complex mixture of >20 compounds resulting from rapid decomposition.

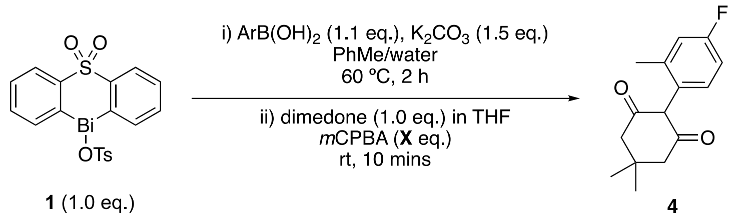

Entry	X	4 (%)
1*	1.5	27
2	1.5	23
3	3.0	27

Supplementary Table 1. Initial optimisation of telescoped reaction with K_2CO_3. * Dimedone added as a solid.

Procedure: A suspension of bismacycle tosylate **1** (59.6 mg, 0.100 mmol), 4-fluoro-2-methylphenylboronic acid (16.9 mg, 0.110 mmol) and K_2CO_3 (20.7 mg, 0.150 mmol) in toluene (1 mL) and water (50 µL) was heated at 60 °C for 2 h. After cooling to room temperature, 1-bromo-4-(trifluoromethyl)benzene (internal standard for ^{19}F NMR spectroscopy) was added before analysis by quantitative ^{19}F NMR spectroscopy to confirm successful transmetallation to form intermediate **2**.

Entry 1: Dimedone (14.0 mg, 0.100 mmol) was added, followed by *m*CPBA (89% purity; 29.1 mg, 0.150 mmol) and the reaction mixture was stirred at room temperature for 10 mins before analysis by quantitative ^{19}F NMR spectroscopy.

Entries 2 & 3: A solution of dimedone (14.0 mg, 0.100 mmol, 1 eq.) in THF (1 mL) was added, followed by *m*CPBA (89% purity; 1.5 or 3.0 eq.) and the reaction mixture was stirred at room temperature for 10 mins before analysis by quantitative ^{19}F NMR spectroscopy.

Commentary: Application of our laboratory's previously reported procedure[4] for the arylation of phenols to the arylation of dimedone afforded a poor yield with a significant amount of unoxidized aryl bismacycle observed (entry 1). Due to its poor solubility in toluene, dimedone was added as a solution in THF (entry 2), though this made a negligible difference to the yield. Increasing the stoichiometry of *m*CPBA to 3.0 equivalents (entry 3), did not improve the yield and unoxidized aryl bismacycle was still observed. We therefore hypothesized that a component from the transmetallation was inhibiting the oxidation (see Supplementary Table 2).

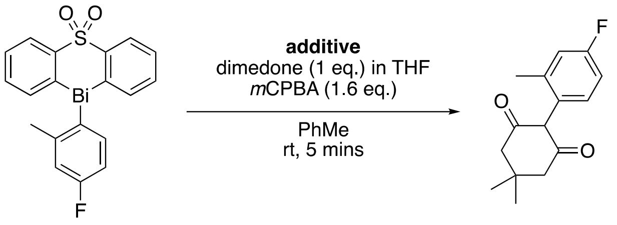

2 4

Entry	Additive	4 (%)
1	-	83
2	Water (1 vol%)	81
3	1.0 eq. $B(OH)_3$ (1.0 eq.)	80
4	4-Fluoro-2-methylphenylboronic acid (0.1 eq.)	77
5	K_2CO_3 (1.5 eq.)	12
6	$NaHCO_3$ (1.0 eq)	81

Supplementary Table 2. Impact of transmetallation components on the bismuth mediated arylation step. Yields determined by ^{19}F NMR spectroscopy against an internal standard.

Procedure: Aryl bismacycle **2** (10.7 mg, 0.020 mmol) and 1-bromo-4-(trifluoromethyl)benzene (internal standard for ^{19}F NMR spectroscopy) were added to an NMR tube and dissolved in toluene (0.5 mL) and C_6D_6 (10 drops) before analysing by quantitative ^{19}F NMR spectroscopy. The **additive** was added, followed by a solution of dimedone (2.80 mg, 0.020 mmol) in THF (0.2 mL) and then *m*CPBA (89% purity; 6.20 mg, 0.032 mmol). The solution was mixed at room temperature for 5 mins before analysis by quantitative ^{19}F NMR spectroscopy.

Commentary: The presence of water and boric acid during the arylation step has no impact on the yield (entries 2 and 3). Residual boronic acid (entry 4) results in a minor reduction in yield. The presence of K_2CO_3 during the arylation step was problematic (entry 5), with incomplete oxidation of the aryl bismacycle observed. As an alternative to K_2CO_3, $NaHCO_3$ proved equally competent in the transmetallation while showing no compatibility issues with subsequent steps (entry 6).

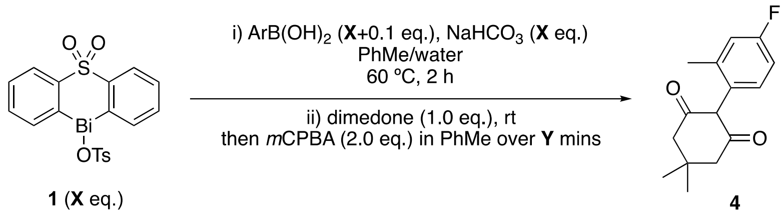

Entry	X	Y	4 (%)
1*	1.0	Solid addition	75
2	1.0	10	83
3	1.1	10	89
4	1.2	10	91
5	1.1	5	87
6	1.1	20	91

Supplementary Table 3. Optimisation of *m*CPBA system. Yield determined by ^{19}F NMR spectroscopy against an internal standard. * *m*CPBA added in a single portion as a solid and dimedone added as a solution in THF.

Procedure: A suspension of bismacycle tosylate **1** (**X** eq.), 4-fluoro-2-methylphenylboronic acid (**X**+0.1 eq.) and $NaHCO_3$ (**X** eq.) in toluene (1 mL) and water (50 µL) was heated at 60 °C for 2 h. After cooling to room temperature, 1-bromo-4-(trifluoromethyl)benzene (internal standard for ^{19}F NMR spectroscopy) was added before analysis by quantitative ^{19}F NMR spectroscopy.

Entry 1 – A solution of dimedone (14.0 mg, 0.100 mmol, 1 eq.) in THF (1 mL) was then added followed by *m*CPBA (89% purity; 38.8 mg, 0.200 mmol) and the reaction mixture was stirred at room temperature for 5 mins before analysis by quantitative ^{19}F NMR spectroscopy.

Entries 2-6 – Dimedone (14.0 mg, 0.100 mmol, 1 eq.) was then added followed by dropwise addition of *m*CPBA (89% purity; 38.8 mg, 0.200 mmol) as a solution in toluene (1 mL) over **Y** mins. The reaction mixture was stirred at room temperature for 5 mins before analysis by quantitative ^{19}F NMR spectroscopy.

Commentary: To offset the instability of the Bi(V) species, *m*CPBA was added slowly as a solution in toluene. Highest yields were achieved using 1.1 eq. of the bismacycle tosylate with *m*CPBA added over 20 minutes (entry 6).

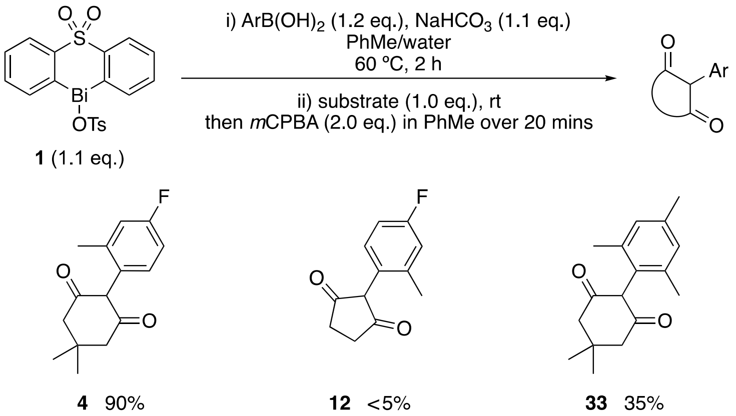

Supplementary Figure 3. Evaluation of robustness of *m*CPBA system. Yield determined by ^{19}F or ^{1}H NMR spectroscopy against an internal standard.

Procedure for compounds 4 & 12: A suspension of bismacycle tosylate **1** (328 mg, 0.550 mmol), 4-fluoro-2-methylphenylboronic acid (92.4 mg, 0.600 mmol), $NaHCO_3$ (46.2 mg, 0.550 mmol) and bromo-4-(trifluoromethyl)benzene (internal standard for ^{19}F NMR spectroscopy) in toluene (5 mL) and water (250 μL) was heated at 60 °C for 2 h. After cooling to room temperature, an aliquot was taken and analysed by quantitative ^{19}F NMR spectroscopy. The substrate (0.500 mmol) was then added followed by dropwise addition of a solution of *m*CPBA (89% purity; 194 mg, 1.00 mmol) in toluene (5 mL) over 20 mins. After stirring at room temperature for 5 mins, the reaction mixture was analysed by quantitative ^{19}F NMR spectroscopy.

Procedure for compound 33: A suspension of bismacycle tosylate **1** (328 mg, 0.550 mmol), 2,4,6-trimethylphenylboronic acid (98.4 mg, 0.600 mmol), $NaHCO_3$ (46.2 mg, 0.550 mmol) in toluene (5 mL) and water (250 μL) was heated at 80 °C for 16 h. After cooling to room temperature, dimedone (70.1 mg, 0.500 mmol) was added followed by dropwise addition of a solution of *m*CPBA (89% purity; 194 mg, 1.00 mmol) in toluene (5 mL) over 20 mins. After stirring at room temperature for 5 mins, the reaction was diluted with EtOAc (20 mL) and extracted with 1M NaOH (3 × 10 mL). The combined aqueous portions were acidified to *p*H 1 with 2 M HCl then extracted with EtOAc (3 × 20 mL). The combined organic portions were dried over $MgSO_4$ and concentrated to dryness. 1,3,5-Trimethoxybenzene (internal standard for ^{1}H NMR spectroscopy) was added and the crude material was dissolved in $CDCl_3$ and analysed by ^{1}H NMR spectroscopy.

Commentary: While the optimised procedure utilising *m*CPBA afforded excellent yields for the coupling of dimedone and 4-fluoro-2-methylphenylboronic acid, the procedure could not be reliably applied to other substrates. For example, coupling of 1,3-cyclopentanedione and 4-fluoro-2-methylphenylboronic acid afforded only trace amounts of product, while the coupling of dimedone and 2,4,6-trimethylphenylboronic acid also afforded a poor yield. Due to the unreliable nature of the procedure, alternative oxidants were investigated.

ii. Arylation of Cyclic 1,3-Diketones: Selectfluor as Oxidant

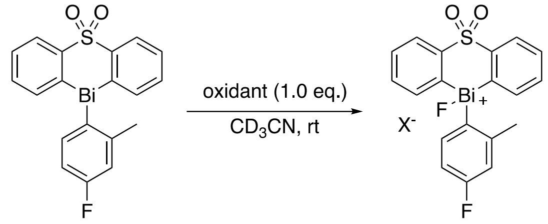

2 (1.0 eq.)

Entry	Oxidant	Yield at 10 mins (%)	Yield at 1 h (%)
1	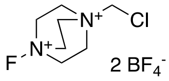	100	100
2	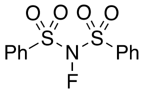	16*	35*
3	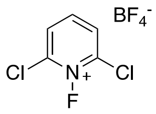	69*	72*
4	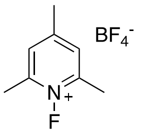	0	0

Supplementary Table 4. Evaluation of fluoronium based oxidants. Yield determined by ^{19}F NMR spectroscopy against an internal standard. * Degradation of oxidised species observed.

Procedure: A mixture of aryl bismacycle **2** (10.7 mg, 0.020 mmol) and 4,4'-bis(trifluoromethyl)-1,1'-biphenyl (internal standard for ^{19}F NMR spectroscopy) in CD_3CN (0.5 mL) was prepared in an NMR tube. The oxidant (0.020 mmol) was added and mixed for 5 mins before analysing by quantitative ^{19}F NMR spectroscopy at 10 mins and 1 h.

Commentary: Oxidation with Selectfluor afforded a stable Bi(V) species within 10 mins and in a quantitative yield (entry 1). NFSI and 2,6-dichloro-1-fluoropyridinium tetrafluoroborate were slower to oxidise **2** and degradation products were observed indicating instability of the Bi(V) species (entries 2 & 3). 1-Fluoro-2,4,6-trimethylpyridinium tetrafluoroborate proved incapable of oxidising the aryl bismacycle (entry 4).

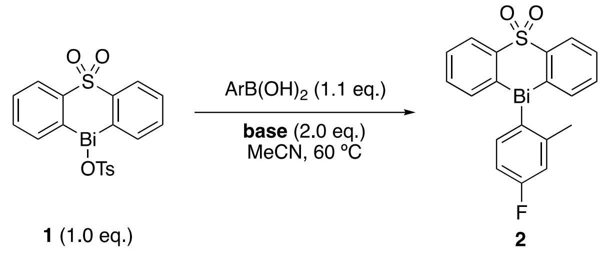

Entry	Base	Yield at 2 h (%)	Yield at 4 h (%)
1	$NaHCO_3$	53	75
2	K_2CO_3	66	71
3	NaOH	64	64
4	K_3PO_4	79	90
5	KF	28	28

Supplementary Table 5. Evaluation of the transmetalation in MeCN. Yield determined by ^{1}H NMR spectroscopy against an internal standard.

Procedure: A mixture of bismacycle tosylate **1** (59.6 mg, 0.100 mmol), 4-fluoro-2-methylphenylboronic acid (16.9 mg, 0.110 mmol), **base** (0.200 mmol) and 1,3,5-trimethoxybenzene (internal standard for ^{1}H NMR spectroscopy) in MeCN (1 mL) was heated at 60 °C and analysed by ^{1}H NMR spectroscopy at 2 and 4 h.

Commentary: Due to poor solubility of Selectfluor, oxidation of the aryl bismacycle **2** requires acetonitrile as solvent. However, transmetallation in MeCN is consistently slower, lower yielding and less reliable than transmetallation in toluene, irrespective of which base is used.

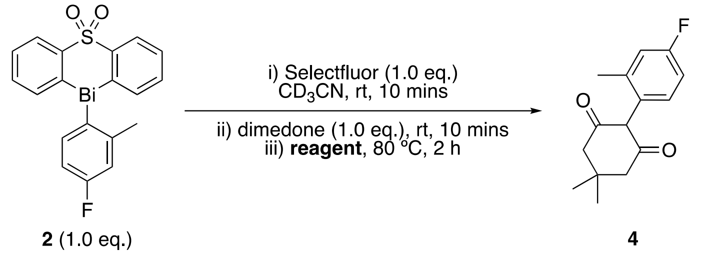

Entry	Reagent	4 (%)
1	None	<5
2	AcOH (2 eq.)	6
3	conc. HCl (2 eq.)	11
4	BzOH (2 eq.)	11
5	$NaHCO_3$ (2 eq.)	9
6	NaOTs (2 eq.)	7
7	[Bu_4N][OAc] (2 eq.)	20
8	TMSOAc (2 eq.)	15
9	[Bu_4N]Cl (2 eq.)	<5
10	NaOBz (2 eq.)	46
11	NaOBz (1 eq.)	41
12	NaOBz (1 eq.) & BzOH (1 eq.)	42
13	NaOBz (2 eq.) & BzOH (2 eq.)	56

Supplementary Table 6. Evaluation of reagents in promoting ligand coupling from ylide. Yield determined by ^{19}F NMR spectroscopy against an internal standard.

Procedure: A stock solution of aryl bismacycle **2** (1 eq.), Selectfluor (1 eq.) and 4,4’-bis(trifluoromethyl)-1,1’-biphenyl (internal standard for ^{19}F NMR spectroscopy) in CD_3CN (0.04 M) was stirred for 10 mins. An aliquot was taken and analysed by quantitative ^{19}F NMR spectroscopy. Dimedone (2.80 mg, 0.020 mmol) was then added to an aliquot of the stock solution (0.5 mL, 0.020 mmol scale) in an NMR tube and mixed for 10 mins. The **reagent(s)** was then added and the mixture heated at 80 °C for 2 h before analysing by quantitative ^{19}F NMR spectroscopy.

Commentary: Product-forming thermolysis of ylide **3c** was most successful in the presence of carboxylate salts and mixtures of carboxylate salts and their carboxylic acids. High yields could not, however, be achieved.

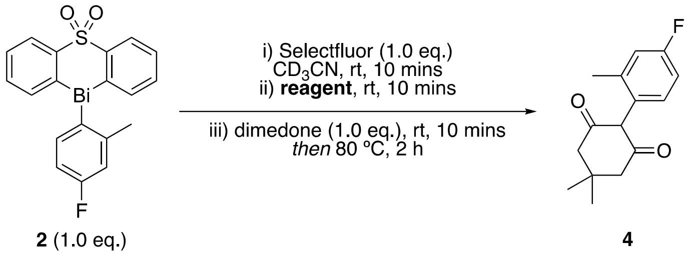

Entry	Reagent	4 (%)
1	None	trace
2*	NaOBz (1 eq.)	55
3*	NaOBz (2 eq.)	71
4*	NaOBz (3 eq.)	72
5	BzOH (2 eq.)	8
6	NaOBz (1 eq.) & BzOH (1 eq.)	63
7	NaOBz (2 eq.) & BzOH (2 eq.)	74
8	NaOBz (3 eq.) & BzOH (3 eq.)	74
9	NaOBz (2 eq.) & BzOH (1 eq.)	74
10	NaOBz (2 eq.) & BzOH (3 eq.)	76
11	Sodium *p*-methoxybenzoate (2 eq.) & *p*-methoxybenzoic acid (2 eq.)	73
12	Sodium *p*-trifluoromethylbenzoate (2 eq.) & *p*-trifluoromethylbenzoic acid (2 eq.)	72
13*	$NaHCO_3$ (2 eq.) and BzOH (3 eq.)	64
14*	$NaHCO_3$ (2.5 eq.) and BzOH (3 eq.)	64

Supplementary Table 7. Optimisation of the bismuth mediated arylation. Yield determined by ^{19}F NMR spectroscopy against an internal standard. * Stirred for 1 h in step (ii).

Procedure: A stock solution of aryl bismacycle **2** (1 eq.), Selectfluor (1 eq.) and 4,4'-bis(trifluoromethyl)-1,1'-biphenyl (internal standard for ^{19}F NMR spectroscopy) in CD_3CN (0.04 M) was stirred for 10 mins. An aliquot was taken and analysed by quantitative ^{19}F NMR spectroscopy. The **reagent(s)** was added to an aliquot of the stock solution (0.5 mL, 0.020 mmol scale) in an NMR tube and mixed for 10 mins. Dimedone (2.80 mg, 0.020 mmol) was

subsequently added and mixed for 10 mins before heating at 80 °C for 2 h then analysing by quantitative ^{19}F NMR spectroscopy.

Commentary: BzOH did promote formation of the dibenzoate Bi(V) species **3d**, resulting in a poor yield of **4** (entry 5), but it was noted that addition of BzOH alongside NaOBz reduced the time it took to form **3d** from 1 h to just 10 mins (entry 7). Given the instability of the dibenzoate Bi(V) species **3d** this was advantageous and warranted its use alongside NaOBz. Changing the relative or absolute stoichiometries of BzOH and NaOBz (entries 6-10), or changing the electronic properties of the reagents (entries 11 & 12), had little to no effect on reaction yield. *In situ* formation of NaOBz from $NaHCO_3$ (already utilised in the transmetalation) and BzOH proved inferior (entries 13 & 14).

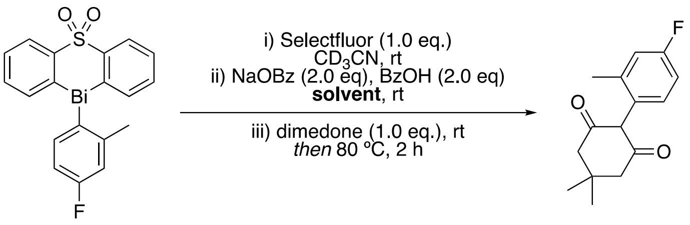

2 (1.0 eq) **4**

Entry	Solvent	4 (%)
1	CD_3CN	74
2	1:1 CH_2Cl_2/CD_3CN	77
3	1:1 THF/CD_3CN	47
4	1:1 EtOAc/CD_3CN	76
5	1:1 $CHCl_3$/CD_3CN	73
6	1:1 PhMe/CD_3CN	79
7	1:3 PhMe/CD_3CN	78
8	3:1 PhMe/CD_3CN	82
9*	3:1 PhMe/CD_3CN	81

Supplementary Table 8. Evaluation of co-solvents. Yield determined by ^{19}F NMR spectroscopy against an internal standard. * Aryl bismacycle telescoped from transmetalation, NaOBz and BzOH were added together with Selectfluor in step (i) and only 1 eq. of BzOH was used.

Procedure for entries 1-6: A solution of aryl bismacycle **2** (1 eq.), Selectfluor (1 eq.) and 4,4’-bis(trifluoromethyl)-1,1’-biphenyl (internal standard for ^{19}F NMR spectroscopy) in CD_3CN (0.08 M) was stirred for 10 mins. An aliquot was taken and analysed by quantitative ^{19}F NMR spectroscopy.

An aliquot of the stock solution (0.25 mL, 0.020 mmol scale) was added to an NMR tube and diluted with the appropriate solvent (0.25 mL). Sodium benzoate (5.76 mg, 0.040 mmol) and benzoic acid (4.88 mg, 0.040 mmol) were added and mixed for 10 mins before dimedone (2.80 mg, 0.020 mmol) was added and mixed for 10 mins. After heating at 80 °C for 2 h the mixture was analysed by quantitative ^{19}F NMR spectroscopy.

Procedure for entries 7 & 8: A mixture of aryl bismacycle **2** (10.7 mg, 0.020 mmol), Selectfluor (7.09 mg, 0.020 mmol) and 4,4'-bis(trifluoromethyl)-1,1'-biphenyl (internal

standard for ^{19}F NMR spectroscopy) in CD_3CN (entry 7, 0.375 mL; entry 8, 0.125 mL) was prepared in an NMR tube and mixed 10 mins. The solution was diluted with toluene (entry 7, 0.125 mL; entry 8, 0.375 mL) then analysed by quantitative ^{19}F NMR spectroscopy. Sodium benzoate (5.76 mg, 0.040 mmol) and benzoic acid (4.88 mg, 0.040 mmol) were added and mixed for 10 mins before dimedone (2.80 mg, 0.020 mmol) was added and mixed for 10 mins. After heating at 80 °C for 2 h the mixture was analysed by quantitative ^{19}F NMR spectroscopy.

Procedure for entry 9: A suspension of bismacycle tosylate **1** (298 mg, 0.500 mmol), 4-fluoro-2-methylphenylboronic acid (84.7 mg, 0.550 mmol) and $NaHCO_3$ (42.0 mg, 0.500 mmol) in toluene (5 mL) and water (250 μL) was heated at 60 °C for 2 h before concentrating to dryness. Selectfluor (177 mg, 0.500 mmol), sodium benzoate (144 mg, 1.00 mmol) and benzoic acid (61.1 mg, 0.500 mmol) were added, followed by MeCN (2.5 mL) and the reaction mixture stirred at room temperature for 0.5 h before diluting with toluene (7.5 mL). Dimedone (70.1 mg, 0.500 mmol) was added and the reaction mixture was stirred at room temperature for 0.5 h then heated at 80 °C for 2 h. After cooling to room temperature, 4,4'-bis(trifluoromethyl)-1,1'-biphenyl (internal standard for ^{19}F NMR spectroscopy) was added and the mixture was analysed by quantitative ^{19}F NMR spectroscopy.

Commentary: Use of CH_2Cl_2 and ethyl acetate as co-solvents (entries 2 & 4) afforded small increases in the yield while chloroform offered no change (entry 5). THF was detrimental to the reaction (entry 3), while toluene proved the most promising co-solvent (entry 6). Further increasing the ratio of toluene to 3:1 toluene/acetonitrile increased the yield to >80% (entry 8). Finally, by carrying out a solvent swap, the entire process could be telescoped from the transmetallation step to combined oxidation/anion exchange and finally arylation (entry 9); this was identified as the optimised procedure.

a. Transmetallation and Arylation in MeCN

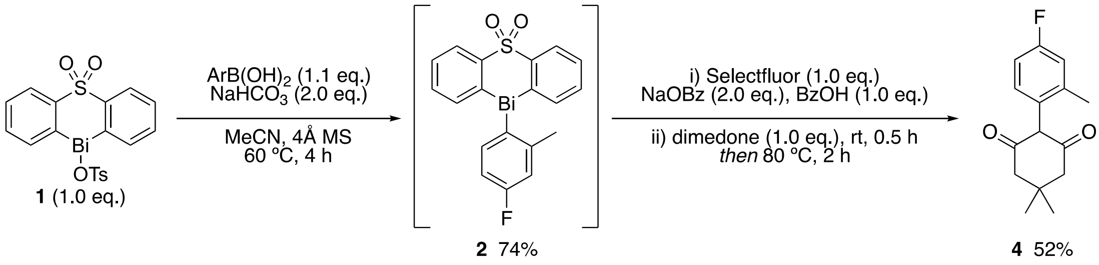

Procedure: A suspension of bismacycle tosylate **1** (59.6 mg, 0.100 mmol), 4-fluoro-2-methylphenylboronic acid (16.9 mg, 0.110 mmol), $NaHCO_3$ (16.8 mg, 0.200 mmol), 4,4'-bis(trifluoromethyl)-1,1'-biphenyl (internal standard for ^{19}F NMR spectroscopy) and 4Å molecular sieves (50 mg) in anhydrous MeCN (1 mL) was heated at 60 °C for 4 h under an atmosphere of N_2. The reaction mixture was analysed by quantitative ^{19}F NMR spectroscopy then cooled to room temperature before Selectfluor (35.4 mg, 0.100 mmol), sodium benzoate (28.8 mg, 0.020 mmol) and benzoic acid (12.2 mg, 0.100 mmol) were charged and stirred for 30 min. Dimedone (14.0 mg, 0.100 mmol) was added and the reaction mixture was stirred at room temperature for 30 min then heated at 80 °C for 2 h. After cooling to room temperature, the reaction mixture was analysed by quantitative ^{19}F NMR spectroscopy.

Commentary: While the transmetallation / oxidation / arylation process can be performed in a single solvent, the yield is reduced relative to the optimised procedure involving solvent exchange prior to oxidation. This reflects the lower efficiency of B-to-Bi transmetallation in MeCN, which – as observed previously[7] – results from competing protodeboronation.

b. C- vs O-Selectivity of Arylation

O-Arylation was not observed by NMR spectroscopic analysis of crude reaction mixtures at any point during this study, irrespective of the substrate combination or reaction conditions employed. To demonstrate the ability of both ^{1}H and ^{19}F NMR spectroscopies to discriminate between the C_{α}-aryl and (putative) O-aryl isomers, we prepared and characterised an authentic sample of O-aryl isomer **4′**. Spectroscopic comparisons of C_{α}-aryl **4** and O-aryl **4′** are presented in Supplementary Figures 4 and 5. We also demonstrate that **4′** does not convert to **4** under the reaction conditions (*infra*), indicating that O-aryl products are not viable intermediates.

3-(4-Fluoro-2-methylphenoxy)-5,5-dimethylcyclohex-2-en-1-one (4′)

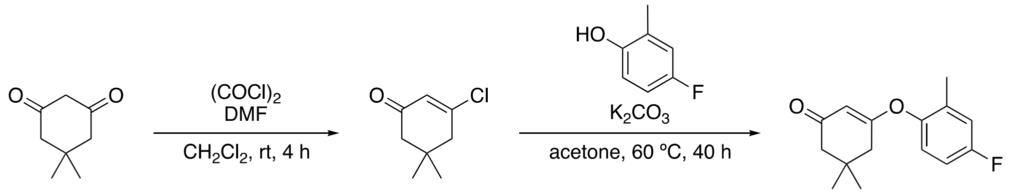

Oxalyl chloride (186 µL, 2.20 mmol) was added dropwise to a solution of dimedone (280 mg, 2.00 mmol) in CH_2Cl_2 (10 mL) and DMF (3 drops). The solution was stirred at r.t. for 2 h before concentrating to dryness. 4-Fluoro-2-methylphenol (277 mg, 2.20 mmol) and potassium carbonate (553 mg, 4.00 mmol) were added, followed by acetone (10 mL), and the suspension was heated at reflux for 40 h before cooling to r.t. The mixture was diluted with EtOAc (30 mL), washed with 2 M HCl (15 mL) and water (2 × 10 mL), dried over $MgSO_4$ and concentrated to dryness. Purification by silica gel chromatography (0-10% EtOAc in cyclohexane) afforded the title compound (323 mg, 1.30 mmol, 65%) as a yellow oil.

^{1}H NMR (400 MHz, $CDCl_3$): δ 6.98 – 6.92 (m, 1H), 6.92 – 6.82 (m, 2H), 4.96 (s, 1H), 2.52 (s, 2H), 2.24 (s, 2H), 2.13 (s, 3H), 1.15 (s, 6H).

^{13}C{^{1}H} NMR (101 MHz, $CDCl_3$): δ 199.4, 176.1, 160.4 (d, *J* = 244.8 Hz), 147.1 (d, *J* = 2.8 Hz), 132.3 (d, *J* = 8.3 Hz), 122.9 (d, *J* = 9.1 Hz), 118.2 (d, *J* = 23.0 Hz), 114.2 (d, *J* = 23.4 Hz), 104.4, 50.8, 42.3, 32.9, 28.4, 16.1 (d, *J* = 1.5 Hz).

^{19}F NMR (376 MHz, $CDCl_3$): δ -116.50 (ddd, *J* = 8.8, 7.0, 5.7 Hz).

ν_{max} (ATR)/cm^{-1}: 2959, 1658, 1614, 1590, 1491, 1469, 1368, 1317, 1195, 1181, 1135, 893, 866, 823, 717, 563, 448.

HRMS calcd. for $C_{15}H_{17}FO_2+H^+$: 249.1285 $[M+H]^+$; found (ESI$^+$) 249.1293.

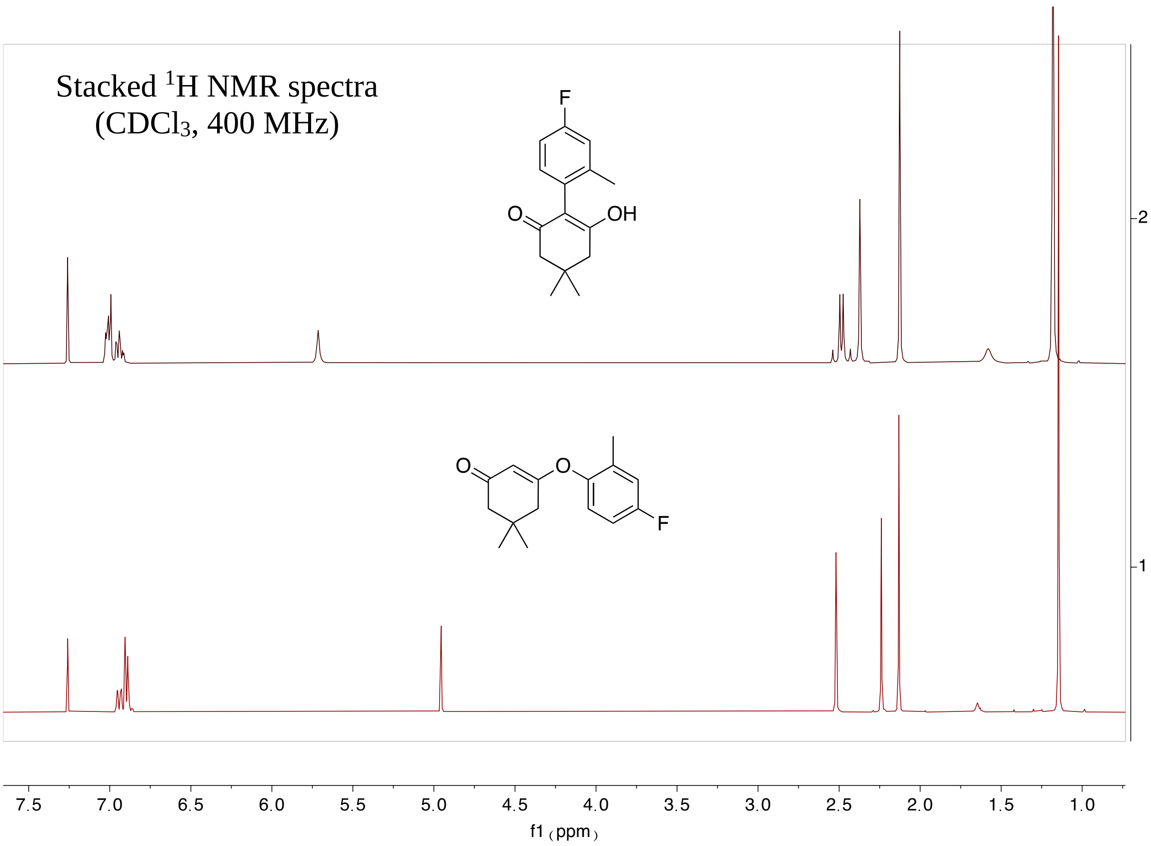

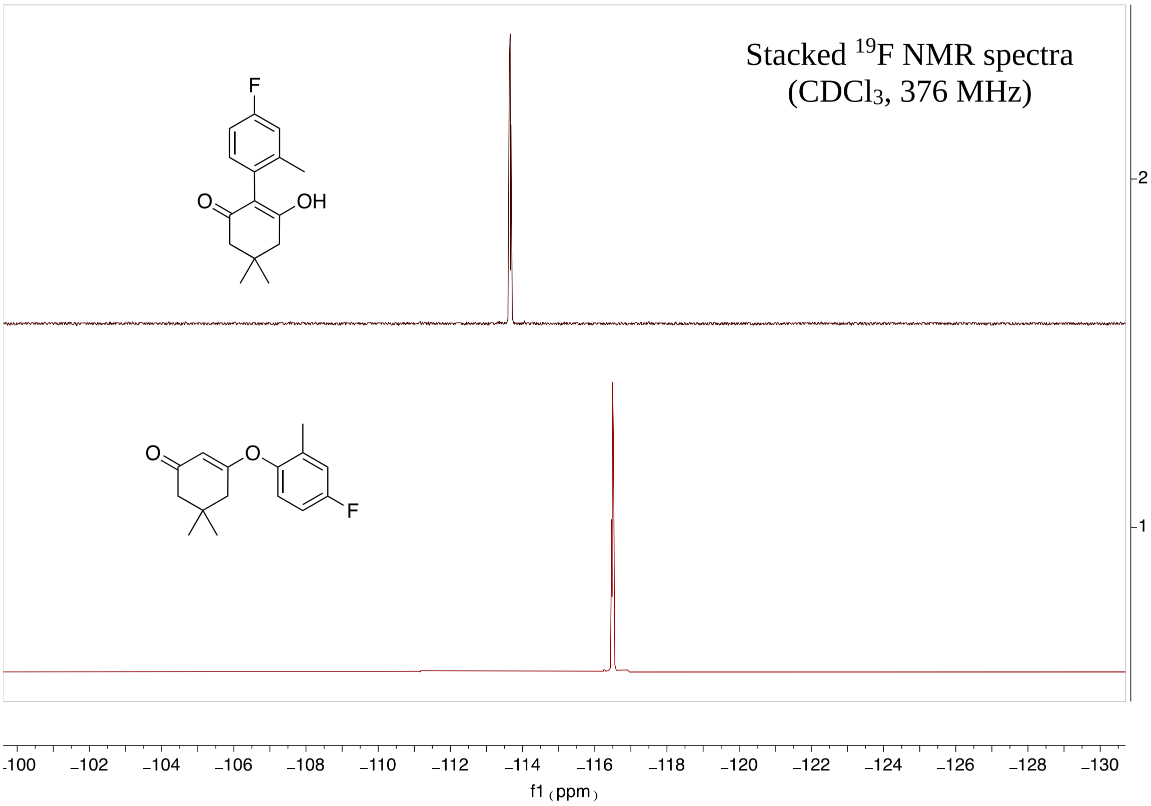

Supplementary Figure 4. Stacked ^{1}H (top) and ^{19}F (bottom) spectra of C-aryl **4** and O-aryl **4′**.

The C-aryl and O-aryl isomers are equally well resolved in crude reaction mixtures. Representative spectra presented in Supplementary Figure 5 illustrate that only C-aryl **4** is observed after both the initial addition of dimedone to the Bi(V) dibenzoate species at room temperature (*i.e.*, at the end of step ii), and after subsequent heating to 80 ° C (*i.e.*, at the end of step iii). O-Aryl isomer **4′** is not observed at either stage, as confirmed by addition of authentic material to the NMR sample.

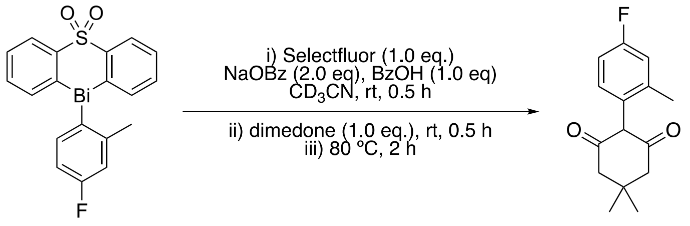

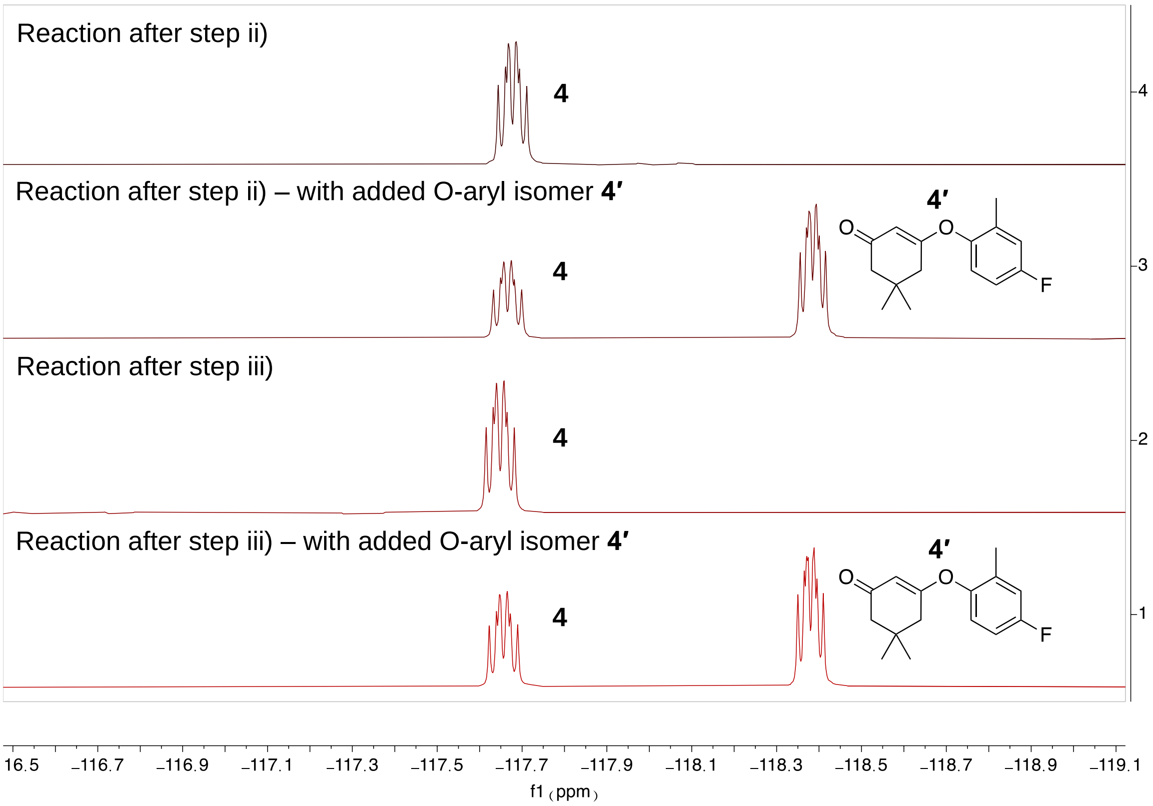

Supplementary Figure 5. ^{19}F NMR spectra of the crude reaction mixture for the synthesis of C-aryl **4**, before and after heating to 80 °C. Spiking with independently-prepared isomer **4′** indicates that O-arylation is not observed, and hence that the reaction is selective for C-arylation.

Procedure: A mixture of aryl bismacycle **2** (10.7 mg, 0.0200 mmol), Selectfluor (7.09 mg, 0.0200 mmol), sodium benzoate (5.76 mg, 0.0400 mmol) and benzoic acid (2.44 mg, 0.0200

mmol) in CD_3CN (0.5 mL) was mixed for 0.5 h. Dimedone (2.80 mg, 0.0200 mmol) was added and mixed for 0.5 h. The reaction up to step ii) was analysed before and after addition of O-aryl **4′**. For the reaction up to step iii), the process was repeated with heating to 80 °C for 2 h after the addition of dimedone. The mixture was then cooled to room temperature before analysing by ^{19}F NMR spectroscopy before and after addition of O-aryl **4′**.

It was proposed by a reviewer that O-aryl isomers may rearrange to C-aryl products under the conditions employed in our Bi(V)-mediated arylation methodology. However, subjecting a sample of O-aryl **4′** to the reaction conditions (80 °C for 2 hours), or more forcing conditions (120 °C for 16 hours), did not result in any observable formation of C-aryl **4**.

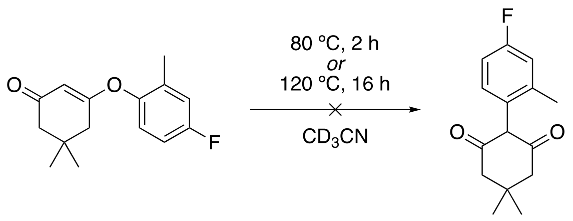

Procedure: A solution of 3-(4-fluoro-2-methylphenoxy)-5,5-dimethylcyclohex-2-en-1-one (4.97 mg, 0.0200 mmol) and 4,4’-bis(trifluoromethyl)-1,1’-biphenyl (internal standard for ^{19}F NMR spectroscopy) in CD_3CN (0.5 mL) was heated at 80 °C for 2 h or 120 °C for 16 h then cooled to room temperature and analysed by quantitative ^{19}F NMR spectroscopy.

iii. Arylation of Fluoroalkyl 1,3-Diketones

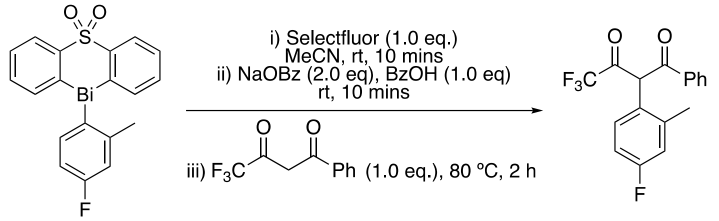

Entry	Deviation from above	44 (%)
1	None	75
2	No NaOBz	68
3	No NaOBz & BzOH	81

Supplementary Table 9. Optimisation of bismuth mediated arylation of fluoroalkyl diones. Yield determined by ^{19}F NMR spectroscopy against an internal standard.

Procedure: A stock solution of aryl bismacycle **2** (1 eq.), Selectfluor (1 eq.) and 4,4'-bis(trifluoromethyl)-1,1'-biphenyl (internal standard for ^{19}F NMR spectroscopy) in MeCN (0.04 M) was stirred for 10 mins. An aliquot was taken and analysed by quantitative ^{19}F NMR spectroscopy. Where applicable, sodium benzoate (5.76 mg, 0.040 mmol) and/or benzoic acid (2.44 mg, 0.020 mmol) were added to an aliquot of the stock solution (0.5 mL, 0.020 mmol scale) in an NMR tube and mixed for 10 mins. 4,4,4-Trifluoro-1-phenylbutane-1,3-dione (4.32 mg, 0.020 mmol) was subsequently added and the mixture heated at 80 °C for 2 h then analysed by quantitative ^{19}F NMR spectroscopy.

Commentary: Applying our procedure for the arylation of cyclic diones afforded a good yield (entry 1), but no ylide formation was observed. As the fluoroalkyl 1,3-diones do not appear to form the ylide under our reaction conditions, we subsequently found that simplified reaction conditions could be used (entry 3).

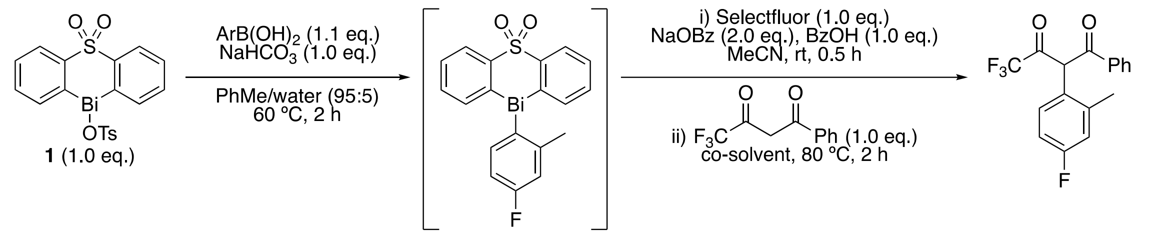

Entry	Deviation from above	Co-solvent	44 (%)
1	None	None	28
2	None	PhMe	33
3	No NaOBz & BzOH	None	34

Supplementary Table 10. Assessment of telescoped aryl bismacycle in the bismuth mediated arylation of fluoroalkyl diones. Yield determined by ^{19}F NMR spectroscopy against an internal standard.

Procedure: A suspension of bismacycle tosylate **1** (298 mg, 0.500 mmol), 4-fluoro-2-methylphenylboronic acid (84.7 mg, 0.550 mmol) and $NaHCO_3$ (42.0 mg, 0.500 mmol) in toluene (5 mL) and water (250 μL) was heated at 60 °C for 2 h before concentrating to dryness. Selectfluor (177 mg, 0.500 mmol) was added alongside sodium benzoate (144 mg, 1.00 mmol) and benzoic acid (61.1 mg, 0.500 mmol) where applicable. MeCN (entries 1 & 3: 5 mL; entry 2: 2.5 mL) was charged and the reaction mixture stirred at room temperature for 0.5 h [entry 2: then diluted with toluene (7.5 mL)] before 4,4,4-trifluoro-1-phenylbutane-1,3-dione (108 mg, 0.500 mmol) was added and the reaction mixture heated at 80 °C for 2 h. After cooling to room temperature, benzotrifluoride (internal standard for ^{19}F NMR spectroscopy) was added and the mixture was analysed by quantitative ^{19}F NMR spectroscopy.

Commentary: Telescoping the aryl bismacycle into the arylation step led to significantly degraded yields under all reaction conditions tested. As a result, we decided to use isolated aryl bismacycles for the arylation of fluoroalkyl 1,3-diones.

5. General Procedures

General procedure 1: Arylation of Cyclic 1,3-Diketones

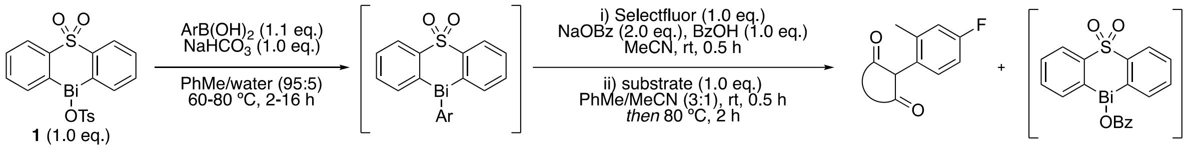

A suspension of bismacycle tosylate **1** (1.0 eq.), aryl boronic acid (1.1 eq.) and $NaHCO_3$ (1.0 eq.) in toluene (0.1 M) and water (5 vol%) was heated at **X** °C for **Y** h (determined complete by ^{1}H NMR spectroscopy) before concentrating to dryness. Selectfluor (1.0 eq.), sodium benzoate (2.0 eq.) and benzoic acid (1.0 eq.) were charged, followed by MeCN (0.2 M) and the reaction mixture stirred at room temperature for 0.5 h before diluting with toluene to a ratio of 3:1 toluene/MeCN. The 1,3-dicarbonyl substrate (1.0 eq.) was added and the reaction mixture was stirred at room temperature for 0.5 h then heated at 80 °C for 2 h. After cooling to room temperature, the reaction mixture was diluted with EtOAc (20 mL) and extracted with sat. aq. $NaHCO_3$ solution (3 × 10 mL).* The combined aqueous portions were acidified to *p*H 1 with 2 M HCl then extracted with EtOAc (3 × 20 mL). The combined organic portions were dried over $MgSO_4$ and concentrated to dryness, purification was achieved as described for individual entries.

* The bismacycle benzoate remains in the organic portion during this step, see below for recovery procedure.

General procedure 2: Arylation of Fluoroalkyl 1,3-Diketones

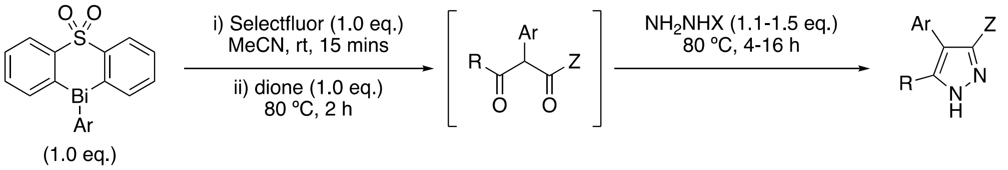

A mixture of the aryl bismacycle (1.0 eq.) and Selectfluor (1.0 eq.) in MeCN (0.1 M) was stirred at room temperature for 15 mins. The dione substrate (1.0 eq.) was charged and the reaction mixture heated at 80 °C for 2 h before cooling to room temperature. 4,4'-Bis(trifluoromethyl)-1,1'-biphenyl (internal standard for ^{19}F NMR spectroscopy) was added and the mixture was analysed by quantitative ^{19}F NMR spectroscopy to determine the NMR

yield for the arylation step. Hydrazine hydrate (1.1 eq.) or phenylhydrazine (1.5 eq.) was charged and the reaction mixture heated at 80 °C for 4 or 16 h, respectively, before cooling to room temperature. *p*-Toluenesulfonic acid monohydrate (2.0 eq.) was added and stirred for 1 h at room temperature before cooling to 0 °C. The mixture was filtered into a separatory funnel and the solid washed with ice-cold MeCN (5 mL) and Et_2O (5 mL) to afford the recovered bismacycle tosylate **1**.* The filtrate was diluted with EtOAc (50 mL), washed with water (10 mL) and brine (2 × 10 mL), dried over $MgSO_4$ and concentrated to dryness, purification was achieved as described for individual entries.

* Reaction affording **45**, carried out on a 0.500 mmol scale, gave the recovered bismacycle tosylate **1** (231 mg, 0.387 mmol, 77%) as a colourless solid in >95% purity.

Bismacycle recovery

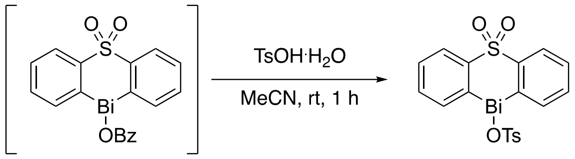

The organic portion was dried over $MgSO_4$ and concentrated to dryness. *p*-Toluenesulfonic acid monohydrate (105 mg, 0.55 mmol, 1.1 eq.) was added to a suspension of the crude bismacycle benzoate in MeCN (2 mL, 0.25 M) and the resulting mixture was stirred at room temperature for 1 h then cooled to 0 °C. The product **1** was collected by filtration and washed with ice cold MeCN and Et_2O, then dried under a flow of air to afford 5,5-dioxido-10H-dibenzo[b,e][1,4]thiabismin-10-yl 4-methylbenzenesulfonate (243 mg, 0.408 mmol, 82%) as a colourless solid.

Transmetallation to Bismacycle Benzoate:

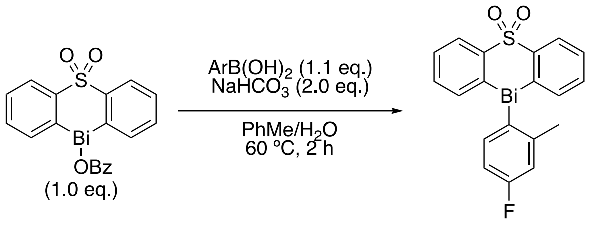

2 93%

A suspension of the bismacycle benzoate (54.6 mg, 0.100 mmol), 4-fluoro-2-methylphenylboronic acid (16.9 mg, 0.110 mmol), $NaHCO_3$ (16.8 mg, 0.200 mmol) and 4,4'-bis(trifluoromethyl)-1,1'-biphenyl (internal standard for ^{19}F NMR spectroscopy) in toluene (1mL) and water (50 μL) was heated at 60 °C for 2 h before analysing by quantitative ^{19}F NMR spectroscopy.

6. Characterisation Data for Arylation Products

2-(4-Fluoro-2-methylphenyl)-5,5-dimethylcyclohexane-1,3-dione (4)

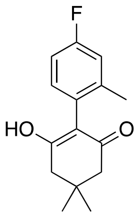

Synthesised according to **general procedure 1** (**X** = 60; **Y** = 2). Using 4-fluoro-2-methylphenylboronic acid (84.7 mg, 0.55 mmol) and dimedone (70.1 mg, 0.50 mmol) afforded, after purification by silica gel column chromatography (20-50% Et_2O in cyclohexane with 1% AcOH), the title compound (95.8 mg, 0.386 mmol, 77%) as a colourless solid.

^{1}H NMR (400 MHz, $CDCl_3$): δ 7.07 – 6.98 (m, 2H), 6.94 (td, *J* = 8.5, 2.7 Hz, 1H), 5.71 (s, 1H), 2.52 (d, *J* = 17.6 Hz, 1H), 2.45 (d, *J* = 17.6 Hz, 1H), 2.37 (s, 2H), 2.13 (s, 3H), 1.184 (s, 3H), 1.177 (s, 3H).

^{13}C{^{1}H} NMR (101 MHz, $CDCl_3$): δ 196.7, 169.6, 163.1 (d, *J* = 247.3 Hz), 141.7 (d, *J* = 8.1 Hz), 132.9 (d, *J* = 8.5 Hz), 125.8 (d, *J* = 3.0 Hz), 117.9 (d, *J* = 21.2 Hz), 115.7, 113.8 (d, *J* = 21.2 Hz), 50.9, 41.7, 32.1, 28.9, 28.3, 20.0.

^{19}F NMR (377 MHz, $CDCl_3$): δ -113.6 (ddd, *J* = 9.7, 8.3, 6.0 Hz).

ν_{max} (ATR)/cm^{-1}: 3037 (br), 2960, 2871, 2638 (br), 1568, 1497, 1335, 1256, 1235, 1149, 1027, 859, 618, 476.

HRMS calcd. for $C_{15}H_{17}FO_2$-H^+: 247.1140 [M-H]$^-$; found (ESI$^-$) 247.1163.

m.p./°C: 202-203.

2-(4-Fluoro-2-methylphenyl)-4,4-dimethylcyclohexane-1,3-dione (5)

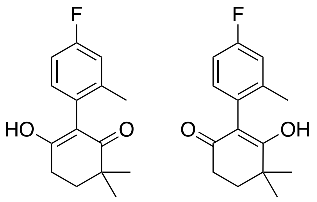

Synthesised according to **general procedure 1** (**X** = 60; **Y** = 2). Using 4-fluoro-2-methylphenylboronic acid (84.7 mg, 0.550 mmol) and 4,4-dimethylcyclohexane-1,3-dione (70.1 mg, 0.500 mmol) afforded, after purification by silica gel column chromatography (0-50% Et_2O in pentane with 1% AcOH), the title compound (88.6 mg, 0.357 mmol, 71%) as a colourless solid.

Observed as a 1:0.5 mixture of tautomeric regioisomers by NMR spectroscopy. [a] Denotes major isomer. [b] Denotes minor isomer.

^{1}H NMR (500 MHz, $CDCl_3$): δ 7.06 – 6.97 (m, 3H)[a,b], 6.98 – 6.89 (m, 1.5H)[a,b], 5.51 (s, 1H)[a], 5.46 (s, 0.5H)[b], 2.71 – 2.52 (m, 3H)[a,b], 2.09 (s, 1.5H)[b], 2.07 (s, 3H)[a], 2.01 – 1.86 (m, 3H)[a,b], 1.343 (s, 1.5H)[b], 1.340 (s, 1.5H)[b], 1.20 (s, 3H)[a], 1.18 (s, 3H)[a].

^{13}C{^{1}H} NMR (126 MHz, $CDCl_3$): δ 201.4[a], 196.5[b], 176.7[b], 168.8[a], 163.1 (d, *J* = 247.4 Hz)[b], 163.0 (d, *J* = 247.0 Hz)[a], 141.6 (d, *J* = 8.1 Hz)[a,b], 133.2 (d, *J* = 8.6 Hz)[b], 133.0 (d, *J* = 8.4 Hz)[a], 126.4 (d, *J* = 3.2 Hz)[a], 126.0 (d, *J* = 3.1 Hz)[b], 118.0 (d, *J* = 21.8 Hz)[b], 117.8 (d, *J* = 21.4 Hz)[a], 115.2[b], 114.9[a], 113.9 (d, *J* = 21.0 Hz)[b], 113.6 (d, *J* = 21.1 Hz)[a], 40.3[a], 35.7[b], 35.1[b], 34.3[a], 34.2[b], 25.8[b], 24.90[a], 24.89[a], 24.7[a], 19.7 (d, *J* = 1.5 Hz)[a], 19.6 (d, *J* = 1.7 Hz)[b].

^{19}F NMR (471 MHz, $CDCl_3$): δ -113.50 (ddd, *J* = 9.7, 8.3, 6.0 Hz, 0.5F)[b], -113.97 (ddd, *J* = 9.7, 8.3, 5.9 Hz, 1F)[a].

ν_{max} (ATR)/cm^{-1}: 3118 (br), 2963, 2928, 2870, 2676 (br), 1584, 1496, 1373, 1329, 1269, 1229, 986, 955.

HRMS calcd. for $C_{15}H_{17}FO_2$-H^+: 247.1140 $[M-H]^-$; found (ESI$^-$) 247.1145.

m.p./°C: 128-129.

2-(4-Fluoro-2-methylphenyl)cyclohexane-1,3-dione (6)

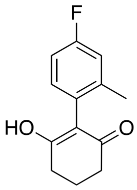

Synthesised according to **general procedure 1** (**X** = 60; **Y** = 2). Using 4-fluoro-2-methylphenylboronic acid (84.7 mg, 0.550 mmol) and 1,3-cyclohexanedione (56.1 mg, 0.500 mmol) afforded, after purification by silica gel column chromatography (50-80% Et_2O in cyclohexane), the title compound (86.9 mg, 0.395 mmol, 79%) as a colourless solid.

^{1}H NMR (500 MHz, $CDCl_3$): δ 7.09 – 6.99 (m, 2H), 6.94 (app. td, *J* = 8.3, 2.7 Hz, 1H), 5.64 (s, 1H), 2.63 (t, *J* = 6.3 Hz, 2H), 2.55 – 2.46 (m, 2H), 2.16 – 2.05 (m, 5H).

^{13}C{^{1}H} NMR (126 MHz, $CDCl_3$): δ 196.8, 171.3, 163.1 (d, *J* = 247.4 Hz), 141.6 (d, *J* = 8.0 Hz), 132.9 (d, *J* = 8.6 Hz), 125.7 (d, *J* = 3.2 Hz), 117.9 (d, *J* = 21.2 Hz), 116.8, 113.8 (d, *J* = 21.1 Hz), 37.0, 27.9, 20.8, 19.8 (d, *J* = 1.5 Hz).

^{19}F NMR (471 MHz, $CDCl_3$): δ -113.63 (ddd, *J* = 9.6, 8.3, 5.9 Hz).

ν_{max} (ATR)/cm^{-1}: 3057 (br), 2952, 2678 (br), 1582, 1497, 1359, 1335, 1235, 1200, 1149, 1069, 988, 860, 593.

HRMS calcd. for $C_{13}H_{13}FO_2$-H^+: 219.0827 [M-H]$^-$; found (ESI$^-$) 219.0833.

m.p./°C: 127-129.

2-(4-Fluoro-2-methylphenyl)-5-phenylcyclohexane-1,3-dione (7)

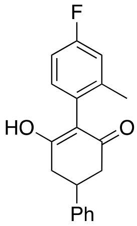

Synthesised according to **general procedure 1** (**X** = 60; **Y** = 2). Using 4-fluoro-2-methylphenylboronic acid (84.7 mg, 0.550 mmol) and 5-phenylcyclohexane-1,3-dione (94.1 mg, 0.500 mmol) afforded, after purification by silica gel column chromatography (20-50% Et_2O in cyclohexane with 1% AcOH), the title compound (109 mg, 0.370 mmol, 74%) as a colourless solid.

Observed as a 1:1 mixture of rotamers by NMR spectroscopy.

^{1}H NMR (500 MHz, $CDCl_3$): δ 7.46 – 7.28 (m, 10H), 7.14 – 6.85 (m, 6H), 5.72 (s, 1H), 5.67 (s, 1H), 3.68 – 3.38 (m, 2H), 3.01 – 2.63 (m, 8H), 2.20 (s, 3H), 2.09 (s, 3H).
^{13}C{^{1}H} NMR (126 MHz, $CDCl_3$): δ 195.9, 195.8, 170.3, 170.2, 163.2 (d, *J* = 247.6 Hz), 163.1 (d, *J* = 247.8 Hz), 142.6, 142.5, 141.8 (d, *J* = 8.2 Hz), 141.6 (d, *J* = 8.1 Hz), 133.0 (d, *J* = 8.6 Hz), 132.8 (d, *J* = 8.6 Hz), 129.04, 129.00, 127.34, 127.32, 126.9, 126.8, 125.5 (d, *J* = 3.1 Hz, 2C), 118.0 (d, *J* = 21.3 Hz, 2C), 116.9, 116.5, 113.94 (d, *J* = 21.2 Hz), 113.88 (d, *J* = 21.2 Hz), 44.1, 43.9, 38.9, 38.6, 35.5, 35.3, 19.9, 19.8.
^{19}F NMR (471 MHz, $CDCl_3$): δ -113.24 – -113.51 (m).
ν_{max} (ATR)/cm^{-1}: 3031 (br), 2958 (br), 2653 (br), 1578, 1496, 1353, 1235, 1148, 1020, 965, 909, 764, 730, 699.
HRMS calcd. for $C_{19}H_{17}FO_2$-H^+: 295.1140 [M-H]$^-$; found (ESI$^-$) 295.1152.
m.p./°C: >150 (decomp).

Methyl 4-(4-fluoro-2-methylphenyl)-3,5-dioxocyclohexane-1-carboxylate (8)

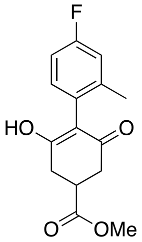

Synthesised according to **general procedure 1** (**X** = 60; **Y** = 2). Using 4-fluoro-2-methylphenylboronic acid (84.7 mg, 0.550 mmol) and methyl 3,5-dioxocyclohexane-1-carboxylate (85.1 mg, 0.500 mmol) afforded, after purification by silica gel column chromatography (30-60% EtOAc in cyclohexane), the title compound (96.9 mg, 0.348 mmol, 70%) as a colourless solid.

Observed as a 1:0.79 mixture by NMR spectroscopy in $CDCl_3$. The spectroscopic data are consistent with the existence of different rotameric forms of the enol tautomer, but – due to signal broadening – we cannot rule out the presence of diketo species. [a] Denotes major form. [b] Denotes minor form.

^{1}H NMR (400 MHz, $CDCl_3$): δ 7.00 – 6.90 (m, 3.6H)[a,b], 6.94 – 6.84 (m, 1.8H)[a,b], 6.02 (br s, 1.8)[a,b], 3.75 (s, 2.4H)[b], 3.73 (s, 3.0H)[a], 3.21 – 3.09 (m, 1.8H)[a,b], 2.87 – 2.65 (m, 7.2H)[a,b], 2.06 (s, 2.4H)[b], 2.01 (s, 3.0H)[a].

^{13}C{^{1}H} NMR (101 MHz, $CDCl_3$): δ 194.5 (br)[a,b]*, 173.3[a], 173.1[b], 170.1 (br)[a,b]*, 163.0 (d, *J* = 247.3 Hz)[a], 162.9 (d, *J* = 247.2 Hz)[b], 141.5 (d, *J* = 8.1 Hz)[a], 141.2 (d, *J* = 8.1 Hz)[b], 132.9 (d, *J* = 8.3 Hz)[b], 132.8 (d, *J* = 8.4 Hz)[a], 125.5 (d, *J* = 3.3 Hz)[a], 125.5 (d, *J* = 3.2 Hz)[b], 117.7 (d, *J* = 21.2 Hz)[a], 117.6 (d, *J* = 21.1 Hz)[b], 116.7[a], 116.4[b], 113.6 (d, *J* = 21.2 Hz)[b], 113.5 (d, *J* = 21.2 Hz)[a], 52.5[a], 52.5[b], 38.6 (br)[a,b]*, 38.0[b], 37.8[a], 30.3 (br)[a,b]*, 19.8 (d, *J* = 1.6 Hz)[b], 19.6 (d, *J* = 1.6 Hz)[a].

^{19}F NMR (376 MHz, $CDCl_3$): δ -113.60 – -113.69 (m)[a,b].

ν_{max} (ATR)/cm^{-1}: 3032 (br), 2956, 2923, 2682 (br), 1732, 1582, 1497, 1438, 1383, 1851, 1236, 1176, 1149, 1098, 1040, 1022, 1000, 969, 866, 756.

HRMS calcd. for $C_{15}H_{15}FO_4$-H^+: 277.0882 [M-H]$^-$; found (ESI$^-$) 277.0893.

m.p./°C: 102-104.

* Signal very broad and just visible in the baseline.

9-(4-Fluoro-2-methylphenyl)-3-oxaspiro[5.5]undecane-8,10-dione (9)

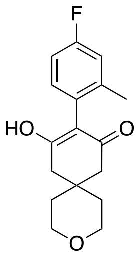

Synthesised according to **general procedure 1** (**X** = 60; **Y** = 2). Using 4-fluoro-2-methylphenylboronic acid (84.7 mg, 0.550 mmol) and 3-oxaspiro[5.5]undecane-8,10-dione (91.1 mg, 0.500 mmol) afforded, after purification by silica gel column chromatography (50-80% EtOAc in cyclohexane), the title compound (98.0 mg, 0.338 mmol, 68%) as a colourless solid.

^{1}H NMR (400 MHz, $CDCl_3$): δ 7.11 – 6.97 (m, 2H), 6.97 – 6.90 (m, 1H), 6.02 – 5.59 (m, 1H), 3.93 – 3.56 (m, 4H), 2.63 (s, 2H), 2.58 (d, *J* = 16.3 Hz, 1H), 2.51 (d, *J* = 16.3 Hz, 1H), 2.10 (s, 3H), 1.69 (app. q, *J* = 5.7 Hz, 4H).

^{13}C{^{1}H} NMR (101 MHz, $CDCl_3$): δ 196.1 (br)*, 170.2 (br)*, 162.8 (d, *J* = 246.9 Hz), 141.2 (d, *J* = 8.1 Hz), 132.8 (d, *J* = 8.5 Hz), 126.1 (d, *J* = 3.0 Hz), 117.5 (d, *J* = 21.1 Hz), 115.8, 113.4 (d, *J* = 21.2 Hz), 63.5, 63.4, 47.4 (br)*, 39.7 (br)*, 36.7, 36.2, 32.5, 20.0 (d, *J* = 1.6 Hz).

^{19}F NMR (376 MHz, $CDCl_3$): δ -113.10 – -113.51 (m).

ν_{max} (ATR)/cm^{-1}: 3064 (br), 3010, 2958, 2926, 2857, 1697 (br), 1715, 1583, 1496, 1376, 1265, 1234, 1149, 1129, 1099, 1031, 960, 843, 750.

HRMS calcd. for $C_{17}H_{19}FO_3$-H^+: 289.1245 [M-H]$^-$; found (ESI$^-$) 289.1248.

m.p./°C: 104-106.

* Signal very broad and just visible in the baseline.

6-(4-Fluoro-2-methylphenyl)-2,2,4,4-tetramethylcyclohexane-1,3,5-trione (10)

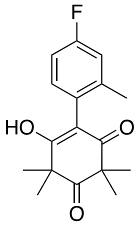

Synthesised according to **general procedure 1** (**X** = 60; **Y** = 2). Using 4-fluoro-2-methylphenylboronic acid (84.7 mg, 0.550 mmol) and 2,2,4,4-tetramethylcyclohexane-1,3,5-trione (91.1 mg, 0.500 mmol) afforded, after purification by silica gel column chromatography (0-20% Et_2O in cyclohexane with 1% AcOH), the title compound (107 mg, 0.369 mmol, 74%) as a colourless solid.

^{1}H NMR (400 MHz, $CDCl_3$): δ 7.10 – 7.02 (m, 2H), 6.99 (app. td, *J* = 8.3, 2.7 Hz, 1H), 5.74 (s, 1H), 2.14 (s, 3H), 1.54 (s, 3H), 1.53 (s, 3H), 1.44 (s, 3H), 1.42 (s, 3H).
^{13}C{^{1}H} NMR (101 MHz, $CDCl_3$): δ 212.5, 196.8, 171.5, 163.3 (d, *J* = 248.3 Hz), 141.6 (d, *J* = 8.1 Hz), 133.1 (d, *J* = 8.6 Hz), 125.5 (d, *J* = 3.2 Hz), 118.2 (d, *J* = 21.3 Hz), 114.1 (d, *J* = 21.3 Hz), 113.6, 55.9, 47.5, 25.3, 25.2, 25.0, 24.0, 19.7 (d, *J* = 1.7 Hz).
^{19}F NMR (376 MHz, $CDCl_3$): δ -112.70 (ddd, *J* = 9.6, 8.2, 5.9 Hz).
ν_{max} (ATR)/cm^{-1}: 3256 (br), 2983, 2940, 2877, 1716, 1601, 1586, 1496, 1471, 1382, 1254, 1228, 1178, 1120, 1088, 962.
HRMS calcd. for $C_{17}H_{19}FO_3$-H^+: 289.1245 [M-H]$^-$; found (ESI$^-$) 289.1257.
m.p./°C: 136-138.

1-Ethyl-4-(4-fluoro-2-methylphenyl)-3,5-dioxocyclohexane-1-carboxylic acid (11)

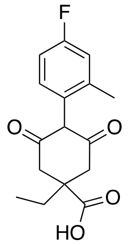

Synthesised according to **general procedure 1** (**X** = 60; **Y** = 2). Using 4-fluoro-2-methylphenylboronic acid (84.7 mg, 0.550 mmol) and **S2** (92.1 mg, 0.500 mmol) afforded, after purification by silica gel column chromatography (60-90% Et_2O in cyclohexane with 1% AcOH), the title compound (106 mg, 0.362 mmol, 72%) as a colourless solid.

Observed as a 1:0.55 mixture by NMR spectroscopy in CD_3OD. The spectroscopic data are consistent with the existence of different forms of the keto tautomer, but – due to signal broadening – we cannot determine whether these are related as rotamers or diastereomers. [a] Denotes major form. [b] Denotes minor form.

^{1}H NMR (400 MHz, CD_3OD): δ 7.08 – 6.69 (m, 4.7H)[a,b], 3.01 (d, *J* = 16.6 Hz, 2H)[a], 2.98 (d, *J* = 16.7 Hz, 1.1H)[b], 2.58 (d, *J* = 16.7 Hz, 1.2H)[b], 2.56 (d, *J* = 16.6 Hz, 2H)[a], 2.06 (s, 1.6H)[b], 2.03 (s, 3.0H)[a], 1.86 – 1.70 (m, 3.2H)[a,b], 1.03 – 0.91 (m, 4.7H)[a,b].

^{13}C{^{1}H} NMR (126 MHz, CD_3OD): δ 178.2[a], 178.1[b], 163.6 (d, *J* = 243.2 Hz)[b], 163.5 (d, *J* = 243.0 Hz)[a], 141.89 (d, *J* = 7.8 Hz)[a], 141.88 (d, *J* = 7.8 Hz)[b], 133.9 (d, *J* = 8.3 Hz)[a], 133.6 (d, *J* = 8.3 Hz)[b], 130.0 (d, *J* = 3.1 Hz)[b], 129.8 (d, *J* = 3.1 Hz)[a], 117.1[a,b], 117.0 (d, *J* = 21.0 Hz)[a,b], 113.0 (d, *J* = 21.3 Hz)[b], 113.0 (d, *J* = 21.3 Hz)[a], 48.3[a,b]*, 42.0 (br)[a,b†], 32.8[a], 32.5[b], 19.90[a], 19.89[b], 8.93[b], 8.86[a].[‡]

^{19}F NMR (376 MHz, CD_3OD): δ -118.49 (app. dt, *J* = 10.1, 7.5 Hz, 0.55F)[b], -118.57 (ddd, *J* = 9.8, 8.6, 6.1 Hz, 1.0F)[a].

ν_{max} (ATR)/cm^{-1}: 3056 (br), 2969, 2922, 2851, 2654 (br), 1791, 1584, 1497, 1377, 1352, 1262, 1236, 1150, 1012, 730.

HRMS calcd. for $C_{16}H_{17}FO_4$-H^+: 291.1038 [M-H]$^-$; found (ESI$^-$) 291.1041.

m.p./°C: 172-174.

* Signal obscured by CD_3OD peak, observed by HMBC.

[†] Signal very broad and just visible in the baseline.

[‡] Carbonyl quaternary centre could not be observed.

2-(4-Fluoro-2-methylphenyl)cyclopentane-1,3-dione (12)

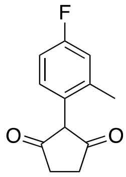

Synthesised according to **general procedure 1** (**X** = 60; **Y** = 2). Using 4-fluoro-2-methylphenylboronic acid (84.7 mg, 0.550 mmol) and 1,3-cyclopentanedione (49.1 mg, 0.500 mmol) afforded, after purification by silica gel column chromatography (0-10% MeOH in Et_2O), the title compound (56.8 mg, 0.275 mmol, 55%) as a colourless solid.

^{1}H NMR (500 MHz, CD_3OD): δ 7.05 (dd, *J* = 8.5, 6.1 Hz, 1H), 6.96 (dd, *J* = 10.0, 2.7 Hz, 1H), 6.89 (app. td, *J* = 8.5, 2.7 Hz, 1H), 2.66 (s, 4H), 2.16 (s, 3H).

^{13}C{^{1}H} NMR (126 MHz, CD_3OD): δ 163.7 (d, *J* = 244.0 Hz), 141.6 (d, *J* = 7.9 Hz), 133.3 (d, *J* = 8.6 Hz), 127.7 (d, *J* = 3.1 Hz), 118.8, 117.2 (d, *J* = 21.3 Hz), 113.1 (d, *J* = 21.3 Hz), 31.6 (br), 20.1 (d, *J* = 1.7 Hz).*

^{19}F NMR (471 MHz, CD_3OD): δ -117.85 (ddd, *J* = 10.0, 8.5, 6.0 Hz).

ν_{max} (ATR)/cm^{-1}: 2998 (br), 2921, 2850, 2627 (br), 1575, 1499, 1376, 1310, 1232, 1150, 960, 672.

HRMS calcd. for $C_{12}H_{11}FO_2$-H^+: 205.0670 [M-H]$^-$; found (ESI$^-$) 205.0677.

m.p./°C: 186-188.

* Carbonyl quaternary centre could not be observed

2-(4-Fluoro-2-methylphenyl)hexahydro-1*H*-4,7-epoxyindene-1,3(2*H*)-dione (13)

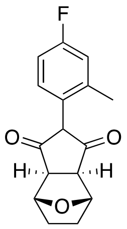

Synthesised according to **general procedure 1** (**X** = 60; **Y** = 2). Using 4-fluoro-2-methylphenylboronic acid (84.7 mg, 0.550 mmol) and **S3** (83.1 mg, 0.500 mmol) afforded, after purification by silica gel column chromatography (50-80% EtOAc in cyclohexane), the title compound (64.7 mg, 0.236 mmol, 47%) as a colourless solid.

^{1}H NMR (400 MHz, CD_3OD): δ 7.03 (dd, *J* = 8.5, 6.1 Hz, 1H), 6.96 (dd, *J* = 10.0, 2.7 Hz, 1H), 6.88 (td, *J* = 8.5, 2.7 Hz, 1H), 4.68 – 4.45 (m, 2H), 2.83 (s, 2H), 2.14 (s, 3H), 1.92 – 1.75 (m, 2H), 1.71 – 1.58 (m, 2H).

^{13}C{^{1}H} NMR (101 MHz, CD_3OD): δ 196.6 (br)*, 163.7 (d, *J* = 244.3 Hz), 141.7 (d, *J* = 7.9 Hz), 133.2 (d, *J* = 8.5 Hz), 127.4 (d, *J* = 3.0 Hz), 121.9, 117.2 (d, *J* = 21.4 Hz), 113.1 (d, *J* = 21.3 Hz), 78.5, 53.3, 29.8, 20.1 (d, *J* = 1.5 Hz).

^{19}F NMR (376 MHz, CD_3OD): δ -117.62 (ddd, *J* = 10.0, 8.6, 6.1 Hz).

ν_{max} (ATR)/cm^{-1}: 3055 (br), 2986, 2954, 2926, 2878, 2662 (br), 1579, 1495, 1373, 1321, 1269, 1233, 1150, 997, 971, 878, 820, 788.

HRMS calcd. for $C_{16}H_{15}FO_3$-H^+: 273.0932 [M-H]$^-$; found (ESI$^-$) 273.0972.

m.p./°C: 217-219.

* Signal very broad and just visible in the baseline.

3-(4-Fluoro-2-methylphenyl)bicyclo[3.2.1]octane-2,4-dione (14)

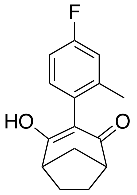

Synthesised according to **general procedure 1** (**X** = 60; **Y** = 2). Using 4-fluoro-2-methylphenylboronic acid (84.7 mg, 0.550 mmol) and bicyclo[3.2.1]octane-2,4-dione (69.1 mg, 0.500 mmol) afforded, after purification by silica gel column chromatography (40-70% Et_2O in cyclohexane with 1% AcOH), the title compound (49.1 mg, 0.199 mmol, 40%) as a colourless solid.

Observed as an indistinguishable mixture of rotamers by NMR spectroscopy.

^{1}H NMR (400 MHz, $CDCl_3$): δ 7.09 – 6.71 (m, 3H), 6.22 (br s, 1H), 3.10 – 2.89 (m, 2H), 2.29 – 2.08 (m, 4H), 2.01 (s, 2H), 1.94 – 1.71 (m, 2H), 1.72 – 1.55 (m, 1H).

^{13}C{^{1}H} NMR (101 MHz, $CDCl_3$): δ 200.9*, 177.7*, 163.0 (d, *J* = 246.9 Hz), 162.9 (d, *J* = 246.9 Hz), 141.6 (d, *J* = 8.1 Hz), 141.3 (d, *J* = 8.1 Hz), 132.9 (d, *J* = 8.5 Hz), 132.7 (d, *J* = 8.5 Hz), 125.6 (d, *J* = 3.1 Hz), 125.5 (d, *J* = 3.0 Hz), 117.74 (d, *J* = 21.2 Hz), 117.67 (d, *J* = 21.2 Hz), 113.6 (d, *J* = 21.2 Hz), 113.5 (d, *J* = 21.2 Hz), 112.9, 112.3, 49.4 (br)†, 41.6 (br)†, 38.3, 38.2, 29.3 (br)†, 26.4 (br)†, 19.8 (d, *J* = 1.6 Hz), 19.3 (d, *J* = 1.6 Hz).

^{19}F NMR (376 MHz, $CDCl_3$): δ -114.0 – -114.4 (m).

ν_{max} (ATR)/cm^{-1}: 3062 (br), 2958, 2875, 2678 (br), 1577, 1497, 1384, 1347, 1291, 1267, 1235, 1169, 1149, 1125, 951, 755.

HRMS calcd. for $C_{15}H_{15}FO_2$-H^+: 245.0983 [M-H]$^-$; found (ESI$^-$) 245.0981.

m.p./°C: 174-176.

* Observed by HMBC.

† Signal very broad and just visible in the baseline.

tert-Butyl 4-(4-fluoro-2-methylphenyl)-3,5-dioxopiperidine-1-carboxylate (15)

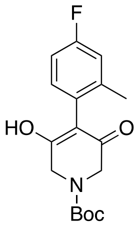

Synthesised according to **general procedure 1** (**X** = 60; **Y** = 2). Using 4-fluoro-2-methylphenylboronic acid (84.7 mg, 0.550 mmol) and _tert_-butyl 3,5-dioxopiperidine-1-carboxylate (106 mg, 0.500 mmol) afforded, after purification by silica gel column chromatography (20-50% Et_2O in cyclohexane with 1% AcOH), the title compound (93.5 mg, 0.291 mmol, 58%) as a colourless solid.

^{1}H NMR (500 MHz, $CDCl_3$): δ 7.07 – 6.96 (m, 2H), 6.96 – 6.84 (m, 1H), 4.54 – 3.99 (m, 4H), 2.10 (s, 3H), 1.49 (s, 9H).
^{13}C{^{1}H} NMR (126 MHz, $CDCl_3$): δ 191.4 (br)*, 168.8 (br)*, 163.1 (d, J = 247.7 Hz), 154.3, 141.4 (d, J = 8.2 Hz), 132.8 (d, J = 8.6 Hz), 124.3 (d, J = 2.5 Hz), 117.8 (d, J = 21.2 Hz), 115.3, 113.6 (d, J = 21.3 Hz), 81.8, 50.7 (br)*, 44.3, 28.4 (br)*, 19.8 (d, J = 1.5 Hz).
^{19}F NMR (471 MHz, $CDCl_3$): δ -113.22 (br s).
ν_{max} (ATR)/cm^{-1}: 3069 (br), 2978, 2930, 2673 (br), 1702, 1586, 1497, 1369, 1235, 1161, 992, 859, 762.
HRMS calcd. for $C_{17}H_{20}FNO_4$-H^+: 320.1304 [M-H]$^-$; found (ESI$^-$) 320.1319.
m.p./°C: 59-60.
* Signal very broad and just visible in the baseline.

4-(4-Fluoro-2-methylphenyl)-2*H*-pyran-3,5(4*H*,6*H*)-dione (16)

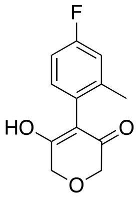

Synthesised according to **general procedure 1** (**X** = 60; **Y** = 2). Using 4-fluoro-2-methylphenylboronic acid (84.7 mg, 0.550 mmol) and 2*H*-pyran-3,5(4*H*,6*H*)-dione (57.1 mg, 0.500 mmol) afforded, after purification by silica gel column chromatography (0-10% MeOH in Et_2O), the title compound (61.0 mg, 0.275 mmol, 55%) as a colourless solid.

^{1}H NMR (500 MHz, $CDCl_3$): δ 7.10 – 7.00 (m, 2H), 6.96 (app. td, *J* = 8.3, 2.7 Hz, 1H), 6.18 (br s, 1H), 4.35 (br s, 4H), 2.15 (s, 3H).

^{13}C{^{1}H} NMR (126 MHz, $CDCl_3$): δ 192.6 (br)*, 169.2 (br)*, 163.2 (d, *J* = 248.3 Hz), 141.6 (d, *J* = 8.1 Hz), 132.8 (d, *J* = 8.6 Hz), 123.3 (d, *J* = 3.2 Hz), 118.0 (d, *J* = 21.3 Hz), 114.4, 113.8 (d, *J* = 21.3 Hz), 71.1 (br)*, 65.6 (br)*, 19.8 (d, *J* = 1.7 Hz).

^{19}F NMR (471 MHz, $CDCl_3$): δ -112.89 (ddd, *J* = 9.6, 8.3, 5.8 Hz).

ν_{max} (ATR)/cm^{-1}: 2980 (br), 2661 (br), 1584, 1498, 1395, 1348, 1235, 1141, 966, 599.

HRMS calcd. for $C_{12}H_{11}FO_3$-H^+: 221.0619 $[M-H]^-$; found (ESI^-) 221.0630.

m.p./°C: 123-124.

* Signal very broad and just visible in the baseline.

3-(4-Fluoro-2-methylphenyl)-4-hydroxy-6-methyl-2H-pyran-2-one (17)

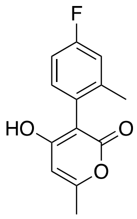

Synthesised according to **general procedure 1** (**X** = 60; **Y** = 2). Using 4-fluoro-2-methylphenylboronic acid (84.7 mg, 0.550 mmol) and 4-hydroxy-6-methyl-2H-pyran-2-one (63.1 mg, 0.500 mmol) afforded, after purification by silica gel column chromatography (50-80% Et_2O in cyclohexane), the title compound (87.4 mg, 0.373 mmol, 75%) as a colourless solid.

^{1}H NMR (500 MHz, $CDCl_3$): δ 7.17 (dd, *J* = 8.3, 5.9 Hz, 1H), 7.06 (dd, *J* = 9.7, 2.7 Hz, 1H), 6.98 (app. td, *J* = 8.3, 2.7 Hz, 1H), 6.00 (q, *J* = 0.9 Hz, 1H), 5.92 (br s, 1H), 2.29 (d, *J* = 0.9 Hz, 3H), 2.21 (s, 3H).
^{13}C{^{1}H} NMR (126 MHz, $CDCl_3$): δ 164.7, 163.5, 163.4 (d, *J* = 248.7 Hz), 163.0, 142.2 (d, *J* = 8.3 Hz), 133.0 (d, *J* = 8.8 Hz), 124.0 (d, *J* = 3.2 Hz), 118.3 (d, *J* = 21.3 Hz), 114.1 (d, *J* = 21.4 Hz), 102.5, 99.5, 20.2, 19.8 (d, *J* = 1.8 Hz).
^{19}F NMR (471 MHz, $CDCl_3$): δ -112.41 (ddd, *J* = 9.7, 8.3, 5.9 Hz).
ν_{max} (ATR)/cm^{-1}: 3067 (br), 2924, 2853, 2682 (br), 1662, 1577, 1498, 1449, 1402, 1361, 1232, 1151, 998, 961, 527.
HRMS calcd. for $C_{13}H_{11}FO_3$-H^+: 233.0619 [M-H]$^-$; found (ESI$^-$) 233.0623.
m.p./°C: 197-198.

5-(4-Fluoro-2-methylphenyl)-1,3-dimethylpyrimidine-2,4,6(1*H*,3*H*,5*H*)-trione (18)

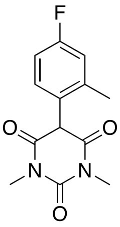

Synthesised according to **general procedure 1** (**X** = 60; **Y** = 2). Using 4-fluoro-2-methylphenylboronic acid (84.7 mg, 0.550 mmol) and 1,3-dimethylbarbituric acid (78.1 mg, 0.500 mmol) afforded, after purification by silica gel column chromatography (50-80% Et_2O in cyclohexane with 1% AcOH), the title compound (53.6 mg, 0.203 mmol, 41%) as a colourless solid.

^{1}H NMR (500 MHz, $CDCl_3$): δ 7.01 – 6.94 (m, 2H), 6.90 (app. td, *J* = 8.4, 2.7 Hz, 1H), 4.81 (s, 1H), 3.38 (s, 6H), 2.35 (s, 3H).

^{13}C{^{1}H} NMR (126 MHz, $CDCl_3$): δ 167.1, 162.7 (d, *J* = 247.9 Hz), 151.6, 139.7 (d, *J* = 8.0 Hz), 130.4 (d, *J* = 8.8 Hz), 128.7 (d, *J* = 3.3 Hz), 118.4 (d, *J* = 21.6 Hz), 113.8 (d, *J* = 21.7 Hz), 52.7, 29.1, 20.2.

^{19}F NMR (471 MHz, $CDCl_3$): δ -113.32 (ddd, *J* = 9.5, 7.9, 5.6 Hz).

ν_{max} (ATR)/cm^{-1}: 2960, 2928, 1679, 1500, 1443, 1376, 1256, 1120, 756.

HRMS calcd. for $C_{13}H_{13}FN_2O_3$-H^+: 263.0837 $[M-H]^-$; found (ESI^-) 263.0849.

m.p./°C: 151-152.

3-(4-Fluoro-2-methylphenyl)chromane-2,4-dione (19)

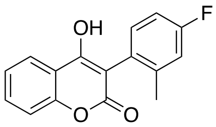

Synthesised according to **general procedure 1** (**X** = 60; **Y** = 2). Using 4-fluoro-2-methylphenylboronic acid (84.7 mg, 0.550 mmol) and 4-hydroxycoumarin (81.1 mg, 0.500 mmol) afforded, after purification by silica gel column chromatography (0-30% Et_2O in cyclohexane with 1% AcOH), the title compound (106 mg, 0.391 mmol, 78%) as a colourless solid.

^{1}H NMR (400 MHz, $CDCl_3$): δ 7.91 (dd, *J* = 7.9, 1.6 Hz, 1H), 7.62 (ddd, *J* = 8.8, 7.4, 1.7 Hz, 1H), 7.40 (dd, *J* = 8.3, 1.1 Hz, 1H), 7.35 (ddd, *J* = 8.3, 7.4, 1.1 Hz, 1H), 7.30 – 7.22 (m, 1H), 7.11 (dd, *J* = 9.6, 2.7 Hz, 1H), 7.04 (app. td, *J* = 8.3, 2.5 Hz, 1H), 6.12 (br s, 1H), 2.25 (s, 3H).
^{13}C{^{1}H} NMR (101 MHz, $CDCl_3$): δ 163.6 (d, *J* = 249.4 Hz), 161.6, 160.1, 153.4, 142.2 (d, *J* = 8.4 Hz), 133.0 (d, *J* = 8.8 Hz), 132.9, 124.3, 124.1 (d, *J* = 3.0 Hz), 123.8, 118.5 (d, *J* = 21.5 Hz), 116.9, 114.8, 114.3 (d, *J* = 21.4 Hz), 105.1, 19.9 (d, *J* = 1.6 Hz).
^{19}F NMR (376 MHz, $CDCl_3$): δ -111.74 (ddd, *J* = 9.6, 8.2, 5.8 Hz).
ν_{max} (ATR)/cm^{-1}: 3083 (br), 2926, 1679, 1608, 1566, 1497, 1271, 1229, 1185, 1153, 1091, 990, 758.
HRMS calcd. for $C_{16}H_{11}FO_3$-H^+: 269.0619 [M-H]$^-$; found (ESI$^-$) 269.0625.
m.p./°C: >210 (decomp).

2-(4-Hydroxy-2-methylphenyl)-5,5-dimethylcyclohexane-1,3-dione (20)

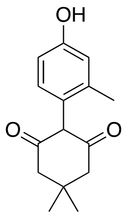

Synthesised according to **general procedure 1** (**X** = 60; **Y** = 2). Using 4-hydroxy-2-methylphenylboronic acid (83.6 mg, 0.550 mmol) and dimedone (70.1 mg, 0.500 mmol) afforded, after purification by silica gel column chromatography (50-80% Et_2O in cyclohexane), the title compound (35.8 mg, 0.145 mmol, 29%) as a colourless solid.

^{1}H NMR (400 MHz, CD_3OD): δ 6.76 (d, *J* = 8.2 Hz, 1H), 6.67 (d, *J* = 2.6 Hz, 1H), 6.60 (dd, *J* = 8.2, 2.6 Hz, 1H), 2.45 (d, *J* = 16.8 Hz, 2H), 2.39 (d, *J* = 16.8 Hz, 2H), 2.02 (s, 3H), 1.17 (s, 3H), 1.16 (s, 3H).

^{13}C{^{1}H} NMR (101 MHz, CD_3OD): δ 157.8, 140.5, 133.3, 124.6, 117.6, 117.1, 113.7, 47.8 (br), 32.9, 28.8, 28.4, 20.2.*

ν_{max} (ATR)/cm^{-1}: 3182 (br), 3022, 2959, 2871, 2674 (br), 1577, 1501, 1457, 1370, 1293, 1243, 1160, 1138, 1106, 1093, 1033, 957, 754.

HRMS calcd. for $C_{15}H_{18}O_3$-H^+: 245.1183 [M-H]$^-$; found (ESI$^-$) 245.1178.

m.p./°C: 186-188.

* Carbonyl quaternary centre could not be observed.

2-(4-Methoxy-2-methylphenyl)-5,5-dimethylcyclohexane-1,3-dione (21)

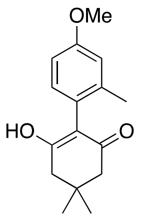

Synthesised according to **general procedure 1** (**X** = 60; **Y** = 2). Using 4-methoxy-2-methylphenylboronic acid (91.3 mg, 0.550 mmol) and dimedone (70.1 mg, 0.500 mmol) afforded, after purification by silica gel column chromatography (20-50% Et_2O in cyclohexane with 1% AcOH), the title compound (82.2 mg, 0.316 mmol, 63%) as a colourless solid.

^{1}H NMR (400 MHz, $CDCl_3$): δ 6.96 (d, *J* = 8.3 Hz, 1H), 6.85 (d, *J* = 2.7 Hz, 1H), 6.79 (dd, *J* = 8.3, 2.7 Hz, 1H), 5.71 (s, 1H), 3.80 (s, 3H), 2.51 (d, *J* = 17.6 Hz, 1H), 2.45 (d, *J* = 17.6 Hz, 1H), 2.37 (s, 2H), 2.11 (s, 3H), 1.18 (s, 3H), 1.17 (s, 3H).

^{13}C{^{1}H} NMR (101 MHz, $CDCl_3$): δ 196.9, 169.5, 160.1, 140.6, 132.4, 121.7, 116.8, 116.2, 112.3, 55.4, 50.9, 41.6, 32.1, 29.0, 28.3, 20.2.

ν_{max} (ATR)/cm^{-1}: 3081 (br), 2958, 2870, 2837, 2666 (br), 1725, 1602, 1574, 1502, 1370, 1341, 1293, 1243, 1160, 1037, 621.

HRMS calcd. for $C_{16}H_{20}O_3$-H^+: 259.1340 [M-H]$^-$; found (ESI$^-$) 259.1332.

m.p./°C: 167-169.

2-(4-Chloro-2-methylphenyl)-5,5-dimethylcyclohexane-1,3-dione (22)

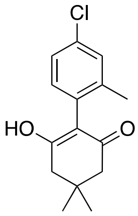

Synthesised according to **general procedure 1** (**X** = 60; **Y** = 2). Using 4-chloro-2-methylphenylboronic acid (93.7 mg, 0.550 mmol) and dimedone (70.1 mg, 0.500 mmol) afforded, after purification by silica gel column chromatography (20-50% Et_2O in cyclohexane with 1% AcOH), the title compound (92.1 mg, 0.348 mmol, 70%) as a colourless solid.

^{1}H NMR (400 MHz, $CDCl_3$): δ 7.30 (d, *J* = 2.3 Hz, 1H), 7.23 (dd, *J* = 8.1, 2.3 Hz, 1H), 6.99 (d, *J* = 8.1 Hz, 1H), 5.57 (s, 1H), 2.52 (d, *J* = 17.6 Hz, 1H), 2.46 (d, *J* = 17.6 Hz, 1H), 2.37 (s, 2H), 2.12 (s, 3H), 1.184 (s, 3H), 1.179 (s, 3H).

^{13}C{^{1}H} NMR (101 MHz, $CDCl_3$): δ 196.5, 169.4, 141.1, 134.9, 132.6, 131.2, 128.5, 127.1, 115.7, 50.8, 41.7, 32.1, 28.9, 28.3, 19.8.

ν_{max} (ATR)/cm^{-1}: 3063 (br), 2961, 2929, 2872, 2670 (br), 1704, 1589, 1484, 1373, 1256, 1205, 1141, 1033, 937, 866, 618.

HRMS calcd. for $C_{15}H_{17}ClO_2$-H^+: 263.0844 [M-H]$^-$; found (ESI$^-$) 263.0842.

m.p./°C: 186-188.

4-(4,4-Dimethyl-2,6-dioxocyclohexyl)-3-methylbenzonitrile (23)

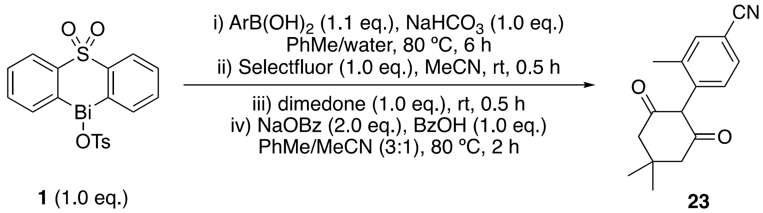

A suspension of bismacycle tosylate **1** (298 mg, 0.500 mmol), 4-cyanophenylboronic acid (88.5 mg, 0.550 mmol) and $NaHCO_3$ (42.0 mg, 0.500 mmol) in toluene (5 mL) and water (250 µL) was heated at 80 °C for 6 h before concentrating to dryness. Selectfluor (177 mg, 0.500 mmol) was charged followed by MeCN (2.5 mL) and the reaction mixture stirred at room temperature for 0.5 h before dimedone (70.1 mg, 0.500 mmol) was added. After stirring at room temperature for 0.5 h, the reaction was diluted with toluene (7.5 mL) before sodium benzoate (144 mg, 1.00 mmol) and benzoic acid (61.1 mg, 0.500 mmol) were added. The reaction was heated at 80 °C for 2 h then cooled to room temperature, diluted with EtOAc (20 mL) and extracted with sat. aq. $NaHCO_3$ solution (3 × 10 mL). The combined aqueous portions were acidified to *p*H 1 with 2 M HCl then extracted with EtOAc (3 × 20 mL). The combined organic portions were dried over $MgSO_4$ and concentrated to dryness. Purification of the crude material by silica gel column chromatography (50-80% Et_2O in cyclohexane with 1% acetic acid) afforded the title compound (54.1 mg, 0.212 mmol, 42%) as a colourless solid.

^{1}H NMR (400 MHz, CD_3OD): δ 7.61 – 7.54 (m, 1H), 7.48 (dd, *J* = 7.8, 1.8 Hz, 1H), 7.13 (d, *J* = 7.8 Hz, 1H), 2.49 (d, *J* = 16.7 Hz, 2H), 2.44 (d, *J* = 16.7 Hz, 2H), 2.15 (s, 3H), 1.19 (s, 3H), 1.18 (s, 3H).

^{13}C{^{1}H} NMR (101 MHz, CD_3OD): δ 141.1, 140.6, 134.0, 133.5, 130.1, 120.2, 116.1, 111.8, 47.7 (br), 33.1, 28.7, 28.4, 19.8.*

ν_{max} (ATR)/cm^{-1}: 3076 (br), 2959, 2871, 2663 (br), 2228, 1610, 1584, 1495, 1372, 1283, 1256, 1145, 1030, 623.

HRMS calcd. for $C_{16}H_{17}NO_2$-H^+: 254.1187 $[M-H]^-$; found (ESI^-) 254.1187.

m.p./°C: 245-246.

* Carbonyl quaternary centre could not be observed.

2-(2-Methoxyphenyl)-5,5-dimethylcyclohexane-1,3-dione (24)

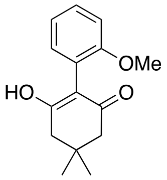

Synthesised according to **general procedure 1** (**X** = 80; **Y** = 16). Using 2-methoxyphenylboronic acid (83.6 mg, 0.550 mmol) and dimedone (70.1 mg, 0.500 mmol) afforded, after purification by silica gel column chromatography (40-70% Et_2O in cyclohexane with 1% AcOH), the title compound (93.4 mg, 0.379 mmol, 76%) as a colourless solid.

^{1}H NMR (400 MHz, $CDCl_3$): δ 7.35 (ddd, *J* = 8.3, 7.4, 1.8 Hz, 1H), 7.14 (dd, *J* = 7.5, 1.8 Hz, 1H), 7.03 (app. td, *J* = 7.4, 1.1 Hz, 1H), 6.98 (dd, *J* = 8.3, 1.1 Hz, 1H), 6.17 (s, 1H), 3.80 (s, 3H), 2.48 (s, 2H), 2.37 (s, 2H), 1.17 (s, 6H).

^{13}C{^{1}H} NMR (101 MHz, $CDCl_3$): δ 196.9, 169.5, 157.2, 133.0, 130.1, 121.5, 119.6, 113.6, 111.9, 56.0, 51.0, 42.3, 32.0, 28.4.

ν_{max} (ATR)/cm^{-1}: 3080 (br), 2963, 2879, 2844, 1707, 1600, 1486, 1468, 1440, 1369, 1300, 1253, 1181, 1164, 1019, 758.

HRMS calcd. for $C_{15}H_{18}O_3$-H^+: 245.1183 [M-H]$^-$; found (ESI$^-$) 245.1175.

m.p./°C: >100 (decomp).

2-(2-Isopropylphenyl)-5,5-dimethylcyclohexane-1,3-dione (25)

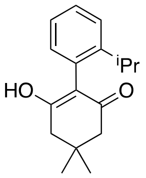

Synthesised according to **GP1** (**X** = 80; **Y** = 16). Using 2-isopropylphenylboronic acid (90.2 mg, 0.550 mmol) and dimedone (70.1 mg, 0.500 mmol) afforded, after purification by silica gel column chromatography (0-20% EtOAc in cyclohexane), the title compound (60.8 mg, 0.235 mmol, 47%) as a colourless solid.

^{1}H NMR (400 MHz, $CDCl_3$): δ 7.43 – 7.33 (m, 2H), 7.23 (app. td, *J* = 7.3, 1.6 Hz, 1H), 7.00 (dd, *J* = 7.6, 1.4 Hz, 1H), 5.73 (br s, 1H), 2.74 (p, *J* = 6.9 Hz, 1H), 2.43 (br app. s, 4H), 1.18 (s, 6H), 1.15 (d, *J* = 6.9 Hz, 6H).

^{13}C{^{1}H} NMR (101 MHz, $CDCl_3$): δ 197.0 (br)*, 169.4 (br)*, 149.7, 131.4, 129.4, 128.8, 126.8, 126.3, 116.7, 50.9 (br), 41.7 (br), 32.1, 30.7, 28.9, 28.3, 24.1 (br).

ν_{max} (ATR)/cm^{-1}: 3062 (br), 2959, 2869, 2662 (br), 1606, 1578, 1487, 1335, 1253, 1206, 1142, 1028, 1012, 908, 889, 755, 727, 616, 495, 466.

HRMS calcd. for $C_{17}H_{22}O_2$-H^+: 257.1547 [M-H]$^-$; found (ESI$^-$) 257.1538.

m.p./°C: 165-166.

* Signal very broad and just visible in the baseline.

5,5-Dimethyl-2-(2-phenoxyphenyl)cyclohexane-1,3-dione (26)

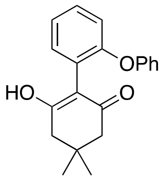

Synthesised according to **general procedure 1** (**X** = 80; **Y** = 6). Using 2-phenoxyphenylboronic acid (118 mg, 0.550 mmol) and dimedone (70.1 mg, 0.500 mmol) afforded, after purification by silica gel column chromatography (20-50% Et_2O in cyclohexane with 1% AcOH), the title compound (107 mg, 0.346 mmol, 69%) as a colourless solid.

^{1}H NMR (400 MHz, $CDCl_3$): δ 7.35 (ddd, *J* = 8.2, 6.6, 2.6 Hz, 1H), 7.30 – 7.16 (m, 4H), 7.10 – 6.96 (m, 2H), 6.92 – 6.80 (m, 2H), 6.14 (s, 1H), 2.37 (s, 2H), 2.20 (br s, 2H), 0.99 (br s, 6H).
^{13}C{^{1}H} NMR (101 MHz, $CDCl_3$): δ 196.4, 169.3, 157.4, 154.5, 133.7, 130.2, 129.8, 124.7, 123.1, 123.0, 120.7, 117.6, 113.1, 50.9, 42.1, 31.7, 28.2 (br).
ν_{max} (ATR)/cm^{-1}: 3067 (br), 2958, 2870, 2672 (br), 1611, 1579, 1486, 1447, 1372, 1235, 1028, 869, 753.
HRMS calcd. for $C_{20}H_{20}O_3$-H^+: 307.1340 [M-H]$^-$; found (ESI$^-$) 307.1347.
m.p./°C: 168-169.

5,5-Dimethyl-2-(2-(trifluoromethoxy)phenyl)cyclohexane-1,3-dione (27)

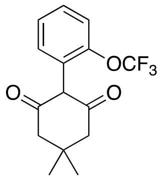

Synthesised according to **general procedure 1** (**X** = 80; **Y** = 6). Using 2-(trifluoromethoxy)phenylboronic acid (113 mg, 0.550 mmol) and dimedone (70.1 mg, 0.500 mmol) afforded, after purification by silica gel column chromatography (30-60% Et_2O in cyclohexane with 1% AcOH), the title compound (107 mg, 0.356 mmol, 71%) as a colourless solid.

^{1}H NMR (400 MHz, CD_3OD): δ 7.36 (app. td, *J* = 7.7, 1.9 Hz, 1H), 7.33 – 7.24 (m, 2H), 7.17 (dd, *J* = 7.5, 1.9 Hz, 1H), 2.49 (d, *J* = 16.8 Hz, 2H), 2.38 (d, *J* = 16.8 Hz, 2H), 1.173 (s, 3H), 1.170 (s, 3H).

^{13}C{^{1}H} NMR (126 MHz, CD_3OD): δ 149.2 (q, *J* = 2.0 Hz), 134.5, 129.8, 128.5, 127.6, 122.0 (q, *J* = 255.3 Hz), 121.3 (q, *J* = 2.0 Hz), 113.0, 48.0 (br)*, 33.0, 29.2, 27.6.†

^{19}F NMR (376 MHz, CD_3OD): δ -58.39.

ν_{max} (ATR)/cm^{-1}: 3068 (br), 2961, 2874, 2658 (br), 1614, 1573, 1492, 1372, 1249, 1213, 1154, 1105, 1030, 764, 617.

HRMS calcd. for $C_{15}H_{15}F_3O_3$-H^+: 299.0901 [M-H]$^-$; found (ESI$^-$) 299.0903.

m.p./°C: 128-130.

* Signal very broad and just visible in the baseline.

† Carbonyl quaternary centre could not be observed.

2-(2-Chlorophenyl)-5,5-dimethylcyclohexane-1,3-dione (28)

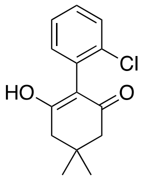

Synthesised according to **general procedure 1** (**X** = 60; **Y** = 2). Using 2-chlorophenylboronic acid (86.0 mg, 0.550 mmol) and dimedone (70.1 mg, 0.500 mmol) afforded, after purification by silica gel column chromatography (20-50% Et_2O in cyclohexane with 1% AcOH), the title compound (93.6 mg, 0.373 mmol, 75%) as a colourless solid.

^{1}H NMR (400 MHz, $CDCl_3$): δ 7.54 – 7.44 (m, 1H), 7.37 – 7.30 (m, 2H), 7.20 – 7.13 (m, 1H), 5.68 (s, 1H), 2.53 (d, *J* = 17.6 Hz, 1H), 2.47 (d, *J* = 17.6 Hz, 1H), 2.42 (d, *J* = 16.1 Hz, 1H), 2.36 (d, *J* = 16.1 Hz, 1H), 1.22 (s, 3H), 1.18 (s, 3H).

^{13}C{^{1}H} NMR (101 MHz, $CDCl_3$): δ 196.1, 169.6, 135.8, 133.1, 130.5, 130.3, 129.9, 127.6, 115.3, 50.7, 41.9, 32.2, 29.0, 28.1.

ν_{max} (ATR)/cm^{-1}: 3070 (br), 2960, 2923, 2870, 2657 (br), 1706, 1604, 1579, 1474, 1377, 1256, 1060, 1032, 1014, 753, 617.

HRMS calcd. for $C_{14}H_{15}ClO_2$-H^+: 249.0688 [M-H]$^-$; found (ESI$^-$) 249.0679.

m.p./°C: 182-184.

5,5-Dimethyl-2-(2-(trifluoromethyl)phenyl)cyclohexane-1,3-dione (29)

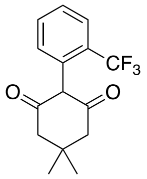

Synthesised according to **general procedure 1** (**X** = 80; **Y** = 16). Using 2-(trifluoromethyl)phenylboronic acid (104 mg, 0.550 mmol) and dimedone (70.1 mg, 0.500 mmol) afforded, after purification by silica gel column chromatography (30-60% Et_2O in cyclohexane with 1% AcOH), the title compound (102 mg, 0.360 mmol, 72%) as a colourless solid.

^{1}H NMR (400 MHz, CD_3OD): δ 7.70 (dd, *J* = 8.0, 1.4 Hz, 1H), 7.57 (app. t, *J* = 7.5 Hz, 1H), 7.45 (app. t, *J* = 7.7 Hz, 1H), 7.13 (d, *J* = 7.6 Hz, 1H), 2.46 (d, *J* = 16.8 Hz, 2H), 2.39 (d, *J* = 16.8 Hz, 2H), 1.185 (s, 3H), 1.178 (s, 3H).
^{13}C{^{1}H} NMR (101 MHz, CD_3OD): δ 134.6, 134.4 (q, *J* = 2.2 Hz), 132.9, 131.4 (q, *J* = 29.5 Hz), 128.7, 126.9 (q, *J* = 5.4 Hz), 125.7 (q, *J* = 272.9 Hz), 115.8, 47.7 (br), 32.8, 29.1, 28.0.*
^{19}F NMR (376 MHz, CD_3OD): δ -62.63.
ν_{max} (ATR)/cm^{-1}: 3070 (br), 2960, 2874, 2661 (br), 1610, 1574, 1374, 1314, 1260, 1162, 1122, 1107, 1062, 1035, 767, 619.
HRMS calcd. for $C_{15}H_{15}F_3O_2$-H^+: 283.0951 [M-H]$^-$; found (ESI$^-$) 283.0955.
m.p./°C: 211-212.
* Carbonyl quaternary centre could not be observed.

5,5-Dimethyl-2-(2-nitrophenyl)cyclohexane-1,3-dione (30)

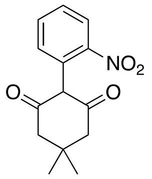

Synthesised according to **general procedure 1** (**X** = 80; **Y** = 16). Using 2-nitrophenylboronic acid (91.8 mg, 0.550 mmol) and dimedone (70.1 mg, 0.500 mmol) afforded, after purification by silica gel column chromatography (40-80% Et_2O in cyclohexane), the title compound (80.2 mg, 0.307 mmol, 61%) as a yellow solid.

^{1}H NMR (400 MHz, CD_3OD): δ 7.94 (dd, *J* = 8.2, 1.3 Hz, 1H), 7.61 (app. td, *J* = 7.6, 1.3 Hz, 1H), 7.46 (ddd, *J* = 8.2, 7.4, 1.5 Hz, 1H), 7.32 (dd, *J* = 7.7, 1.5 Hz, 1H), 2.48 (d, *J* = 16.8 Hz, 2H), 2.39 (d, *J* = 16.8 Hz, 2H), 1.20 (s, 3H), 1.16 (s, 3H).

^{13}C{^{1}H} NMR (101 MHz, CD_3OD): δ 151.3, 134.9, 133.4, 129.8, 129.0, 124.9, 114.4, 48.4 (br), 33.0, 29.1, 28.1.*

ν_{max} (ATR)/cm^{-1}: 3068 (br), 2958, 2872, 2660 (br), 1615, 1578, 1521, 1476, 1361, 1254, 1092 1030, 853, 786, 749, 708, 653.

HRMS calcd. for $C_{14}H_{15}NO_4$-H^+: 260.0928 [M-H]$^-$; found (ESI$^-$) 260.0927.

m.p./°C: 227-229.

* Carbonyl quaternary centre could not be observed.

5,5-Dimethyl-2-(naphthalen-1-yl)cyclohexane-1,3-dione (31)

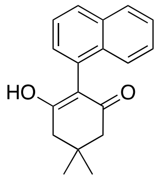

Synthesised according to **general procedure 1** (**X** = 60; **Y** = 2). Using naphthalen-1-ylboronic acid (94.6 mg, 0.550 mmol) and dimedone (70.1 mg, 0.500 mmol) afforded, after purification by silica gel column chromatography (20-50% Et_2O in cyclohexane with 1% AcOH), the title compound (111 mg, 0.415 mmol, 83%) as a colourless solid.

^{1}H NMR (400 MHz, $CDCl_3$): δ 7.94 – 7.81 (m, 2H), 7.64 – 7.57 (m, 1H), 7.56 – 7.43 (m, 3H), 7.30 (dd, *J* = 7.0, 1.3 Hz, 1H), 5.80 (s, 1H), 2.59 (d, *J* = 17.7 Hz, 1H), 2.54 (d, *J* = 17.7 Hz, 1H), 2.50 (d, *J* = 16.1 Hz, 1H), 2.44 (d, *J* = 16.1 Hz, 1H), 1.30 (s, 3H), 1.23 (s, 3H).
^{13}C{^{1}H} NMR (101 MHz, $CDCl_3$): δ 197.0, 170.1, 134.4, 132.6, 129.6, 129.4, 128.8, 128.3, 126.8, 126.4, 126.0, 125.2, 115.3, 51.0, 41.9, 32.1, 28.8, 28.7.
ν_{max} (ATR)/cm^{-1}: 3059 (br), 2960, 2672 (br), 1710, 1596, 1572, 1400, 1369, 1330, 1241, 1213, 1143, 1029, 798, 777, 755.
HRMS calcd. for $C_{18}H_{18}O_2$-H^+: 265.1234 [M-H]$^-$; found (ESI$^-$) 265.1232.
m.p./°C: 183-185.

2-(6-Fluoro-2-methylpyridin-3-yl)-5,5-dimethylcyclohexane-1,3-dione (32)

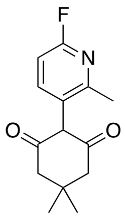

Synthesised according to **general procedure 1** (**X** = 60; **Y** = 2). Using 6-fluoro-2-methylpyridin-3-ylboronic acid (85.2 mg, 0.550 mmol) and dimedone (70.1 mg, 0.500 mmol) afforded, after purification by silica gel column chromatography (60-80% Et_2O in cyclohexane), the title compound (93.6 mg, 0.375 mmol, 75%) as a colourless solid.

^{1}H NMR (400 MHz, CD_3OD):) δ 7.51 (app. t, *J* = 8.2 Hz, 1H), 6.86 (dd, *J* = 8.3, 2.7 Hz, 1H), 2.49 (d, *J* = 16.9 Hz, 2H), 2.45 (d, *J* = 16.9 Hz, 2H), 2.24 (s, 3H), 1.19 (s, 3H), 1.18 (s, 3H).
^{13}C{^{1}H} NMR (101 MHz, CD_3OD): δ 163.4 (d, *J* = 237.4 Hz), 157.8 (d, *J* = 12.7 Hz), 146.5 (d, *J* = 8.1 Hz), 127.7 (d, *J* = 4.7 Hz), 114.2, 107.1 (d, *J* = 36.6 Hz), 47.7 (br), 33.0, 28.7, 28.4, 22.1.*
^{19}F NMR (376 MHz, CD_3OD): -74.32 (dd, *J* = 8.2, 2.7 Hz).
ν_{max} (ATR)/cm^{-1}: 3076 (br), 2959, 2872, 2688 (br), 1577, 1463, 1372, 1273, 1254, 1144, 1036, 1010, 959, 824, 753, 634.
HRMS calcd. for $C_{14}H_{16}FNO_2$-H^+: 248.1092 [M-H]$^-$; found (ESI$^-$) 248.1097.
m.p./°C: 218-220.
* Carbonyl quaternary centre could not be observed.

2-(2-Chloro-6-methoxyphenyl)-5,5-dimethylcyclohexane-1,3-dione (33)

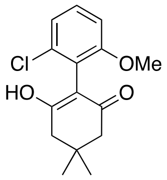

Synthesised according to **general procedure 1** (**X** = 80; **Y** = 16). Using 2-chloro-6-methoxyphenylboronic acid (103 mg, 0.550 mmol) and dimedone (70.1 mg, 0.500 mmol) afforded, after purification by silica gel column chromatography (50-80% Et_2O in cyclohexane with 1% AcOH), the title compound (114 mg, 0.406 mmol, 81%) as a colourless solid.

^{1}H NMR (400 MHz, $CDCl_3$): δ 7.32 – 7.23 (m, 1H), 7.09 (dd, *J* = 8.2, 1.0 Hz, 1H), 6.84 (dd, *J* = 8.4, 1.0 Hz, 1H), 5.68 (s, 1H), 3.74 (s, 3H), 2.51 (d, *J* = 17.5 Hz, 1H), 2.46 (d, *J* = 17.5 Hz, 1H), 2.38 (s, 2H), 1.21 (s, 3H), 1.18 (s, 3H).

^{13}C{^{1}H} NMR (101 MHz, $CDCl_3$): δ 196.0, 169.7, 159.2, 136.9, 130.7, 122.3, 118.8, 111.5, 109.8, 56.3, 50.8, 41.9, 32.3, 28.7, 28.3.

ν_{max} (ATR)/cm^{-1}: 3078 (br), 3007, 2959, 2871, 2837, 1572, 1463, 1432, 1371, 1258, 1043, 1031, 1014, 852, 775, 755.

HRMS calcd. for $C_{15}H_{17}ClO_3$-H^+: 279.0793 $[M-H]^-$; found (ESI^-) 279.0795.

m.p./°C: 186-188.

2-Mesityl-5,5-dimethylcyclohexane-1,3-dione (34)

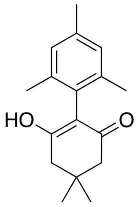

Synthesised according to **general procedure 1** (**X** = 80; **Y** = 16). Using 2,4,6-trimethylphenylboronic acid (98.4 mg, 0.550 mmol) and dimedone (70.1 mg, 0.500 mmol) afforded, after purification by silica gel column chromatography (10-30% Et_2O in cyclohexane with 1% AcOH), the title compound (85.3 mg, 0.330 mmol, 66%) as a colourless solid.

^{1}H NMR (400 MHz, $CDCl_3$): δ 6.94 (s, 2H), 5.43 (s, 1H), 2.49 (s, 2H), 2.40 (s, 2H), 2.28 (s, 3H), 2.06 (s, 6H), 1.20 (s, 6H).

^{13}C{^{1}H} NMR (126 MHz, $CDCl_3$): δ 196.6, 168.7, 138.72, 138.69, 129.2, 125.7, 115.2, 51.0, 41.4, 32.2, 28.8, 21.2, 20.1.

ν_{max} (ATR)/cm^{-1}: 3028 (br), 2961, 2917, 2676 (br), 1585, 1346, 1255, 1039, 621.

HRMS calcd. for $C_{17}H_{22}O_2$-H^+: 257.1547 [M-H]$^-$; found (ESI$^-$) 257.1554.

m.p./°C: 204-205.

2-(4-Bromo-2,6-dimethylphenyl)-5,5-dimethylcyclohexane-1,3-dione (35)

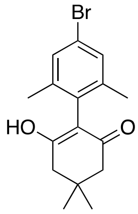

Synthesised according to **general procedure 1** (**X** = 80; **Y** = 16). Using (4-bromo-2,6-dimethylphenyl)boronic acid (126 mg, 0.550 mmol) and dimedone (70.1 mg, 0.500 mmol) afforded, after purification by silica gel column chromatography (0-50% Et_2O in cyclohexane with 1% AcOH), the title compound (97.5 mg, 0.302 mmol, 60%) as a colourless solid.

^{1}H NMR (400 MHz, $CDCl_3$): δ 7.28 (s, 2H), 5.37 (s, 1H), 2.50 (s, 2H), 2.40 (s, 2H), 2.08 (s, 6H), 1.20 (s, 6H).

^{13}C{^{1}H} NMR (126 MHz, $CDCl_3$): δ 196.3, 168.9, 141.1, 131.2, 128.2, 123.1, 114.4, 50.9, 41.5, 32.2, 28.8, 20.0.

ν_{max} (ATR)/cm^{-1}: 2957, 2923, 2868, 2638 (br), 1580, 1572, 1469, 1359, 1331, 1310, 1256, 1149, 1039, 589.

HRMS calcd. for $C_{16}H_{19}BrO_2$-H^+: 321.0496 [M-H]$^-$; found (ESI$^-$) 321.0495.

m.p./°C: >200 (decomp).

2-(2,6-Dimethyl-4-(prop-1-yn-1-yl)phenyl)-5,5-dimethylcyclohexane-1,3-dione (36)

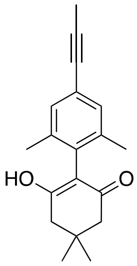

Synthesised according to **general procedure 1** (**X** = 80; **Y** = 16). Using **S5** (103 mg, 0.550 mmol) and dimedone (70.1 mg, 0.500 mmol) afforded, after purification by silica gel column chromatography (10-40% Et_2O in cyclohexane with 1% AcOH), the title compound (99.1 mg, 0.351 mmol, 70%) as a colourless solid.

^{1}H NMR (400 MHz, $CDCl_3$): δ 7.15 (s, 2H), 5.42 (s, 1H), 2.49 (s, 2H), 2.39 (s, 2H), 2.06 (s, 6H), 2.04 (s, 3H), 1.20 (s, 6H).

^{13}C{^{1}H} NMR (101 MHz, $CDCl_3$): δ 196.5, 168.8, 138.9, 131.3, 128.7, 124.6, 114.9, 86.1, 79.6, 51.0, 41.5, 32.2, 28.8, 20.0, 4.5.

ν_{max} (ATR)/cm^{-1}: 3046 (br), 2959, 2920, 2869, 2665 (br), 2360, 1583, 1435, 1367, 1254, 1136, 1040, 1011, 871, 757, 630.

HRMS calcd. for $C_{19}H_{22}O_2$-H^+: 281.1547 [M-H]$^-$; found (ESI$^-$) 281.1546.

m.p./°C: 224-226.

9-(4-Bromo-2,6-dimethylphenyl)-3-methoxy-3-azaspiro[5.5]undecane-8,10-dione (37)

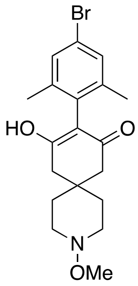

Synthesised according to **general procedure 1** (**X** = 80; **Y** = 16). Using (4-bromo-2,6-dimethylphenyl)boronic acid (126 mg, 0.550 mmol) and **S4** (106 mg, 0.500 mmol) afforded, after purification by silica gel column chromatography (20-70% EtOAc in cyclohexane), the title compound (129 mg, 0.327 mmol, 65%) as a colourless solid.

^{1}H NMR (400 MHz, $CDCl_3$): δ 7.27 (s, 2H), 5.70 (br s, 1H), 3.59 (s, 3H), 3.25 (br s, 2H), 2.90 – 2.28 (m, 6H), 2.05 (s, 6H), 1.99 – 1.50 (m, 4H).

^{13}C{^{1}H} NMR (126 MHz, $CDCl_3$): δ 194.9 (br)*, 168.3 (br)*, 140.9, 131.2, 128.1, 123.1, 114.6, 59.5, 50.7, 50.0 (br)*, 44.9 (br)*, 41.0 (br)*, 36.4 (br)*, 34.6 (br), 32.6, 20.0.

ν_{max} (ATR)/cm^{-1}: 3058 (br), 2931, 2846, 2658 (br), 1599, 1573, 1467, 1379, 1347, 1254,1142, 1044, 933, 900, 732.

HRMS calcd. for $C_{19}H_{24}BrNO_3$-H^+: 392.0867 [M-H]$^-$; found (ESI$^-$) 392.0876.

m.p./°C: >100 (decomp).

* Signal very broad and just visible in the baseline.

***tert*-Butyl 9-(4-bromo-2,6-dimethylphenyl)-8,10-dioxo-3-azaspiro[5.5]undecane-3-carboxylate (38)**

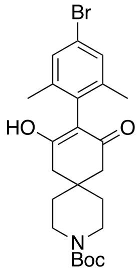

Synthesised according to **general procedure 1** (**X** = 80; **Y** = 16). Using (4-bromo-2,6-dimethylphenyl)boronic acid (126 mg, 0.550 mmol) and *tert*-butyl 8,10-dioxo-3-azaspiro[5.5]undecane-3-carboxylate (141 mg, 0.500 mmol) afforded, after purification by silica gel column chromatography (0-50% EtOAc in cyclohexane), the title compound (158 mg, 0.340 mmol, 68%) as a colourless solid.

^{1}H NMR (500 MHz, $CDCl_3$): δ 7.26 (s, 2H), 5.89 (br s, 1H), 3.66 – 3.23 (m, 4H), 2.53 (s, 4H), 2.05 (s, 6H), 1.63 (t, *J* = 5.8 Hz, 4H), 1.46 (s, 9H).

^{13}C{^{1}H} NMR (126 MHz, $CDCl_3$): δ 195.1 (br)*, 168.2 (br)*, 154.9, 140.9, 131.1, 128.2, 123.0, 114.7, 79.9, 47.2 (br)*, 39.6 (br), 38.9 (br)*, 36.0, 33.5, 28.6, 20.1.

ν_{max} (ATR)/cm^{-1}: 3097 (br), 2975, 2927, 2877, 2671 (br), 1680, 1601, 1573, 1429, 1366, 1278, 1250, 1165, 1040, 924, 906, 731.

HRMS calcd. for $C_{23}H_{30}BrNO_4$-H^+: 462.1285 [M-H]$^-$; found (ESI$^-$) 462.1272.

m.p./°C: 167-168.

* Signal very broad and just visible in the baseline.

tert-Butyl 9-(2,6-dimethyl-4-(prop-1-yn-1-yl)phenyl)-8,10-dioxo-3-azaspiro[5.5]undecane-3-carboxylate (39)

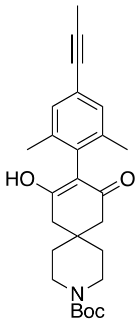

Synthesised according to **general procedure 1** (**X** = 80; **Y** = 16). Using **S5** (103 mg, 0.550 mmol) and *tert*-butyl 8,10-dioxo-3-azaspiro[5.5]undecane-3-carboxylate (141 mg, 0.500 mmol) afforded, after purification by recrystallisation from hot methanol, the title compound (148 mg, 0.349 mmol, 70%) as a colourless solid.

^{1}H NMR (400 MHz, $CDCl_3$): δ 7.15 (s, 2H), 5.50 (s, 1H), 3.65 – 3.28 (m, 4H), 2.58 (s, 2H), 2.52 (s, 2H), 2.043 (s, 6H), 2.036 (s, 3H), 1.64 (t, *J* = 5.9 Hz, 4H), 1.46 (s, 9H).

^{13}C{^{1}H} NMR (126 MHz, $CDCl_3$): δ 195.1, 167.8, 154.9, 138.8, 131.3, 128.4, 124.8, 115.2, 86.2, 79.9, 79.6, 47.4, 39.6 (br), 38.7, 36.0, 33.5, 28.6, 20.1, 4.5.

ν_{max} (ATR)/cm^{-1}: 3115 (br), 2974, 2920, 2873, 2683 (br), 1685, 1595, 1478, 1430, 1366, 1278, 1249, 1164, 1041, 925, 869, 731.

HRMS calcd. for $C_{26}H_{33}NO_4$-H^+: 422.2337 [M-H]$^-$; found (ESI$^-$) 422.2334.

m.p./°C: >250.

4-(5-Bromo-2-ethylphenyl)-2,2,6,6-tetramethyl-2*H*-pyran-3,5(4*H*,6*H*)-dione (40)

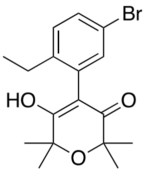

Synthesised according to **general procedure 1** (**X** = 60; **Y** = 2). Using (5-bromo-2-ethylphenyl)boronic acid (126 mg, 0.550 mmol) and 2,2,6,6-tetramethyl-2*H*-pyran-3,5(4*H*,6*H*)-dione (85.1 mg, 0.500 mmol) afforded, after purification by silica gel column chromatography (0-10% EtOAc in cyclohexane), the title compound (133 mg, 0.375 mmol, 75%) as a colourless solid.

^{1}H NMR (400 MHz, $CDCl_3$): δ 7.46 (dd, *J* = 8.3, 2.2 Hz, 1H), 7.24 – 7.16 (m, 2H), 5.74 (s, 1H), 2.55 – 2.19 (m, 2H), 1.57 (s, 6H), 1.45 (s, 6H), 1.08 (t, *J* = 7.6 Hz, 3H).

^{13}C{^{1}H} NMR (101 MHz, $CDCl_3$): δ 197.1, 172.4, 144.2, 134.3, 132.5, 131.1, 130.6, 120.2, 110.0, 78.4, 72.7, 28.8, 28.7, 27.8, 27.6, 25.9, 14.9.

ν_{max} (ATR)/cm^{-1}: 3179 (br), 2979, 2936, 2874 (br), 1753, 1724, 1600, 1583, 1561, 1478, 1399, 1375, 1361, 1326, 1224, 1169, 1145, 1106, 1032, 966, 823.

HRMS calcd. for $C_{17}H_{21}BrO_3$-H^+: 351.0601 [M-H]$^-$; found (ESI$^-$) 351.0605.

m.p./°C: 160-162.

4-(4-Fluoro-2-methylphenyl)-3-phenyl-5-(trifluoromethyl)-1*H*-pyrazole (45)

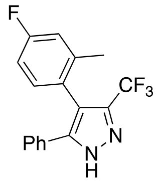

Synthesised according to **general procedure 2**. Using aryl bismacycle **2a** (267 mg, 0.500 mmol) and 4,4,4-trifluoro-1-phenylbutane-1,3-dione (108 mg, 0.500 mmol) afforded, after purification by preparative HPLC (50-80% MeCN in water), the title compound (121 mg, 0.378 mmol, 76%) as a colourless solid.

^{1}H NMR (400 MHz, CDCl$_3$): δ 10.80 (br s, 1H), 7.39 – 7.28 (m, 3H), 7.24 – 7.17 (m, 3H), 7.02 – 6.88 (m, 2H), 1.98 (s, 3H).

^{13}C{^{1}H} NMR (101 MHz, CDCl$_3$): δ 162.9 (d, *J* = 246.9 Hz), 142.4, 142.1 (q, *J* = 36.0 Hz), 140.4 (d, *J* = 8.0 Hz), 132.8 (d, *J* = 8.5 Hz), 129.4, 129.3, 128.3, 126.4, 125.9 (d, *J* = 3.2 Hz), 121.4 (q, *J* = 270.1 Hz), 117.1 (d, *J* = 21.3 Hz), 116.3 (d, *J* = 1.8 Hz), 113.1 (d, *J* = 21.3 Hz), 20.2 (d, *J* = 1.5 Hz).

^{19}F NMR (377 MHz, CDCl$_3$): δ -60.95 (s, 3F), -114.03 (app. td, *J* = 8.9, 5.8 Hz, 1F).

ν_{max} (ATR)/cm^{-1}: 3163 (br), 2931 (br), 1530, 1479, 1265, 1179, 1131, 1108, 1084, 989, 972, 908, 865, 820, 751, 730, 691.

HRMS calcd. for $C_{17}H_{12}F_4N_2$-H^+: 319.0864 [M-H]$^-$; found (ESI$^-$) 319.0861.

m.p./°C: 153-154.

5-(Difluoromethyl)-4-(4-fluoro-2-methylphenyl)-3-phenyl-1*H*-pyrazole (46)

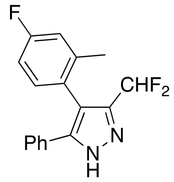

Synthesised according to **general procedure 2**. Using aryl bismacycle **2a** (267 mg, 0.500 mmol) and 4,4-difluoro-1-phenylbutane-1,3-dione (99.1 mg, 0.500 mmol) afforded, after purification by preparative HPLC (50-80% MeCN in water), the title compound (109 mg, 0.362 mmol, 72%) as a yellow solid.

^{1}H NMR (400 MHz, CDCl$_3$): δ 11.65 (br s, 1H), 7.37 – 7.15 (m, 6H), 7.02 – 6.85 (m, 2H), 6.54 (app. t, *J* = 53.9 Hz, 1H), 1.96 (s, 3H).

^{13}C{^{1}H} NMR (101 MHz, CDCl$_3$): δ 162.8 (d, *J* = 246.7 Hz), 144.8 (t, *J* = 25.9 Hz), 142.6, 140.4 (d, *J* = 8.0 Hz), 132.9 (d, *J* = 8.4 Hz), 129.2, 129.1, 126.4, 126.4 (d, *J* = 3.3 Hz), 117.1 (d, *J* = 21.1 Hz), 116.6 (t, *J* = 2.2 Hz), 113.1 (d, *J* = 21.1 Hz), 111.0 (t, *J* = 235.1 Hz), 20.3 (d, *J* = 1.6 Hz).*

^{19}F NMR (377 MHz, CDCl$_3$): δ -111.50 (dd, *J* = 311.3, 54.0 Hz, 1F), -113.21 (dd, *J* = 311.3, 53.9 Hz, 1F), -114.35 (app. td, *J* = 9.1, 5.9 Hz, 1F).

ν_{max} (ATR)/cm^{-1}: 3159 (br), 2945 (br), 1529, 1479, 1444, 1270, 1227, 1153, 1129, 1104, 1079, 1036, 1009, 976, 943, 909, 866, 840, 823, 776, 733, 694.

HRMS calcd. for $C_{17}H_{12}F_3N_2$-H^+: 301.0958 [M-H]$^-$; found (ESI$^-$) 301.0957.

m.p./°C: 116-117.

* One carbon signal was not observed.

3-(*tert*-Butyl)-4-(4-fluoro-2-methylphenyl)-5-(perfluoroethyl)-1*H*-pyrazole (47)

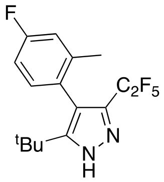

Synthesised according to **general procedure 2**. Using aryl bismacycle **2a** (267 mg, 0.500 mmol) and 1,1,1,2,2-pentafluoro-6,6-dimethylheptane-3,5-dione (123 mg, 0.500 mmol) afforded, after purification by preparative HPLC (70-95% MeCN in water), the title compound (124 mg, 0.354 mmol, 71%) as an off-white solid.

^{1}H NMR (400 MHz, $CDCl_3$): δ 11.46 (br s, 1H), 7.10 (dd, *J* = 8.5, 5.9 Hz, 1H), 6.96 (dd, *J* = 9.7, 2.7 Hz, 1H), 6.89 (app. td, *J* = 8.4, 2.8 Hz, 1H), 2.11 (s, 3H), 1.22 (s, 9H).

^{13}C{^{1}H} NMR (101 MHz, $CDCl_3$): δ 162.8 (d, *J* = 246.5 Hz), 150.3, 140.9 (d, *J* = 8.0 Hz), 140.4 (t, *J* = 27.0 Hz), 133.5 (d, *J* = 8.3 Hz), 127.2 (d, *J* = 3.2 Hz), 119.0 (qt, *J* = 286.1, 37.9 Hz), 116.9 (d, *J* = 1.4 Hz), 116.5 (d, *J* = 21.2 Hz), 112.2 (d, *J* = 21.1 Hz), 111.7 (tq, *J* = 251.9, 39.2 Hz), 32.6, 29.5, 20.7.

^{19}F NMR (377 MHz, $CDCl_3$): δ -83.59 (app. t, *J* = 2.4 Hz, 3F), -110.33 (dq, *J* = 286.5, 2.5 Hz, 1F), -112.12 (dq, *J* = 286.5, 2.4 Hz, 1F), -114.66 (ddd, *J* = 9.7, 8.3, 5.9 Hz, 1F).

ν_{max} (ATR)/cm^{-1}: 3233 (br), 2969, 1518, 1336, 1197, 1125, 1100, 1032, 1007, 953, 936, 868, 823, 752, 735.

HRMS calcd. for $C_{16}H_{16}F_6N_2$-H^+: 349.1145 [M-H]$^-$; found (ESI$^-$) 349.1136.

m.p./°C: 168-169.

3,5-Bis(difluoromethyl)-4-(4-fluoro-2-methylphenyl)-1*H*-pyrazole (48)

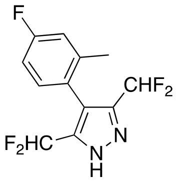

Synthesised according to **general procedure 2**. Using aryl bismacycle **2a** (267 mg, 0.500 mmol) and 1,1,5,5-tetrafluoropentane-2,4-dione (86.0 mg, 0.500 mmol) afforded, after purification by preparative HPLC (50-70% MeCN in water), the title compound (98.4 mg, 0.356 mmol, 71%) as an off-white solid.

^{1}H NMR (400 MHz, CDCl$_3$): δ 11.18 (br s, 1H), 7.16 (dd, *J* = 8.4, 5.8 Hz, 1H), 7.01 (dd, *J* = 9.6, 2.7 Hz, 1H), 6.94 (app. td, *J* = 8.3, 2.7 Hz, 1H), 6.53 (t, *J* = 53.7 Hz, 2H), 2.13 (s, 3H).

^{13}C{^{1}H} NMR (101 MHz, CDCl$_3$): δ 163.2 (d, *J* = 247.8 Hz), 140.6 (d, *J* = 8.2 Hz), 140.4 (br), 132.7 (d, *J* = 8.7 Hz), 123.4 (d, *J* = 3.1 Hz), 119.5, 117.1 (d, *J* = 21.5 Hz), 113.0 (d, *J* = 21.5 Hz), 109.3 (t, *J* = 236.1 Hz), 20.3.

^{19}F NMR (377 MHz, CDCl$_3$): δ -112.36 (dd, *J* = 313.2, 53.7 Hz, 2F), -113.14 – -113.25 (m, 1F), -113.67 (dd, *J* = 313.2, 53.5 Hz, 2F).

ν_{max} (ATR)/cm^{-1}: 3098 (br), 2956, 1530, 1488, 1351, 1295, 1272, 1228, 1154, 1194, 1091, 1041, 1009, 946, 868, 846.

HRMS calcd. for $C_{12}H_9F_5N_2$-H^+: 275.0613 [M-H]$^-$; found (ESI$^-$) 275.0617.

m.p./°C: 128-129.

4-(2-Chlorophenyl)-3-phenyl-5-(trifluoromethyl)-1*H*-pyrazole (49)

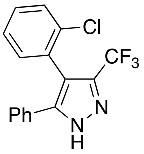

Synthesised according to **general procedure 2**. Using aryl bismacycle **2b** (268 mg, 0.500 mmol) and 4,4,4-trifluoro-1-phenylbutane-1,3-dione (108 mg, 0.500 mmol) afforded, after purification by preparative HPLC (50-70% MeCN in water), the title compound (134 mg, 0.415 mmol, 83%) as an oil which solidified on standing to a colourless solid.

^{1}H NMR (400 MHz, CDCl$_3$): δ 11.52 (br s, 1H), 7.46 (dd, *J* = 7.7, 1.3 Hz, 1H), 7.43 – 7.20 (m, 8H).

^{13}C{^{1}H} NMR (101 MHz, CDCl$_3$): δ 142.9, 142.1 (q, *J* = 36.1 Hz), 135.3, 132.8, 130.0, 129.9, 129.8, 129.4, 129.2, 128.2, 126.9, 126.8, 121.3 (q, *J* = 270.1 Hz), 115.4.

^{19}F NMR (377 MHz, CDCl$_3$): δ -60.98.

ν_{max} (ATR)/cm^{-1}: 3161 (br), 2942 (br), 1480, 1443, 1315, 1258, 1214, 1180, 1135, 1104, 1061, 989, 976, 909, 771, 750, 736, 692.

HRMS calcd. for $C_{16}H_{10}ClF_3N_2$-H^+: 321.0412 [M-H]$^-$; found (ESI$^-$) 321.0412.

m.p./°C: 138-139.

3-Phenyl-4-(2-(trifluoromethoxy)phenyl)-5-(trifluoromethyl)-1*H*-pyrazole (50)

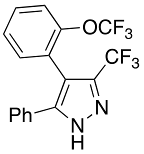

Synthesised according to **general procedure 2**. Using aryl bismacycle **2c** (293 mg, 0.500 mmol) and 4,4,4-trifluoro-1-phenylbutane-1,3-dione (108 mg, 0.500 mmol) afforded, after purification by preparative HPLC (50-70% MeCN in water), the title compound (153 mg, 0.411 mmol, 82%) as an oil which solidified on standing to an off-white solid.

^{1}H NMR (400 MHz, $CDCl_3$): δ 11.47 (br s, 1H), 7.49 – 7.39 (m, 1H), 7.40 – 7.28 (m, 6H), 7.27 – 7.22 (m, 2H).

^{13}C{^{1}H} NMR (101 MHz, $CDCl_3$): δ 147.6 (d, *J* = 1.7 Hz), 143.3, 142.1 (q, *J* = 34.9 Hz), 133.1, 130.1, 129.5, 129.2, 128.2, 127.2, 126.7, 124.0, 121.3 (q, *J* = 270.0 Hz), 120.5 (d, *J* = 1.9 Hz), 120.4 (q, *J* = 258.3 Hz), 112.5.

^{19}F NMR (377 MHz, $CDCl_3$): δ -57.40 (s, 3F), -60.87 (s, 3F).

ν_{max} (ATR)/cm^{-1}: 3164 (br), 2943, 1531, 1480, 1450, 1317, 1252, 1220, 1172, 1150, 1136, 1089, 990, 974, 768, 752, 694, 628.

HRMS calcd. for $C_{17}H_{10}F_6N_2O-H^+$: 371.0625 $[M-H]^-$; found (ESI^-) 371.0630.

m.p./°C: 132-133.

4-(2-Fluoro-6-methoxyphenyl)-3-phenyl-5-(trifluoromethyl)-1*H*-pyrazole (51)

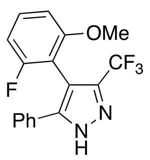

Synthesised according to **general procedure 2**. Using aryl bismacycle **2d** (275 mg, 0.500 mmol) and 4,4,4-trifluoro-1-phenylbutane-1,3-dione (108 mg, 0.500 mmol) afforded, after purification by preparative HPLC (50-70% MeCN in water), the title compound (104 mg, 0.309 mmol, 62%) as an oil which solidified on standing to a colourless solid.

^{1}H NMR (400 MHz, $CDCl_3$): δ 11.48 (br s, 1H), 7.33 (d, *J* = 2.0 Hz, 6H), 6.80 – 6.66 (m, 2H), 3.66 (s, 3H).

^{13}C{^{1}H} NMR (101 MHz, $CDCl_3$): δ 161.3 (d, *J* = 246.0 Hz), 159.3 (d, *J* = 6.3 Hz), 143.4, 142.7 (q, *J* = 36.2 Hz), 130.5 (d, *J* = 10.5 Hz), 129.2, 129.1, 128.7, 126.8, 121.4 (q, *J* = 270.1 Hz), 108.1 (d, *J* = 22.5 Hz), 107.9 (d, *J* = 18.4 Hz), 107.4 – 107.3 (m), 106.7 (d, *J* = 3.0 Hz), 56.0.

^{19}F NMR (377 MHz, $CDCl_3$): δ -62.30 (d, *J* = 2.6 Hz, 3F), -111.28 – -111.47 (m, 1F).

ν_{max} (ATR)/cm^{-1}: 3166 (br), 2939, 1623, 1580, 1531, 1469, 1438, 1420, 1293, 1277, 1243, 1212, 1175, 1131, 1099, 1082, 988, 971, 942, 909, 790, 776, 760, 750, 731, 693, 626.

HRMS calcd. for $C_{17}H_{12}F_4N_2O$-H^+: 335.0813 [M-H]$^-$; found (ESI$^-$) 335.0807.

m.p./°C: 71-72.

4-(2-Fluoro-6-(trifluoromethyl)phenyl)-3-phenyl-5-(trifluoromethyl)-1*H*-pyrazole (52)

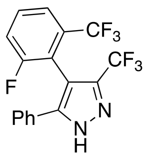

Synthesised according to **general procedure 2***. Using aryl bismacycle **2e** (294 mg, 0.500 mmol) and 4,4,4-trifluoro-1-phenylbutane-1,3-dione (108 mg, 0.500 mmol) afforded, after purification by preparative HPLC (50-70% MeCN in water), the title compound (120 mg, 0.321 mmol, 64%) as a yellow solid.

* In the pyrazole forming step 1.5 eq. of hydrazine hydrate was used and the reaction heated at 80 °C for 16 h.

^{1}H NMR (400 MHz, CDCl$_3$): δ 11.36 (br s, 1H), 7.59 – 7.49 (m, 2H), 7.43 – 7.29 (m, 4H), 7.25 (dd, *J* = 8.2, 2.0 Hz, 2H).

^{13}C{^{1}H} NMR (101 MHz, CDCl$_3$): δ 161.5 (d, *J* = 247.7 Hz), 143.7, 142.9 (q, *J* = 35.9 Hz), 132.5 (qd, *J* = 30.4, 1.9 Hz), 130.9 (d, *J* = 8.7 Hz), 129.6, 129.2, 127.8, 127.0, 122.9 (qd, *J* = 274.2, 3.5 Hz), 122.3 (qd, *J* = 4.8, 3.1 Hz), 121.1 (q, *J* = 270.2 Hz), 119.3 (d, *J* = 23.0 Hz), 117.7 (dq, *J* = 20.0, 1.8 Hz), 107.0.

^{19}F NMR (377 MHz, CDCl$_3$): δ -59.95 – -60.09 (m, 3F), -61.61 – -61.87 (m, 3F), -108.55 – -108.70 (m, 1F).

ν_{max} (ATR)/cm^{-1}: 3169 (br), 2944, 1476, 1462, 1319, 1291, 1256, 1215, 1146, 1099, 1070, 990, 973, 906, 808, 769, 752, 694.

HRMS calcd. for $C_{17}H_9F_7N_2$-H^+: 373.0581 [M-H]$^-$; found (ESI$^-$) 373.0586.

m.p./°C: 143-144.

4-(4-Fluoro-2-methylphenyl)-3-methyl-5-(trifluoromethyl)-1*H*-pyrazole (53)

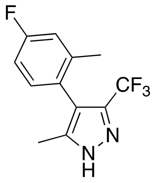

Synthesised according to **general procedure 2**. Using aryl bismacycle **2a** (267 mg, 0.500 mmol) and 1,1,1-trifluoropentane-2,4-dione (77.1 mg, 0.500 mmol) afforded, after purification by preparative HPLC (50-80% MeCN in water), the title compound (88.1 mg, 0.341 mmol, 68%) as an off-white solid.

^{1}H NMR (400 MHz, CDCl$_3$): δ 11.89 (br s, 1H), 7.10 (dd, *J* = 8.4, 5.9 Hz, 1H), 7.00 (dd, *J* = 9.7, 2.7 Hz, 1H), 6.92 (app. td, *J* = 8.4, 2.7 Hz, 1H), 2.14 (s, 3H), 2.09 (s, 3H).

^{13}C{^{1}H} NMR (101 MHz, CDCl$_3$): δ 162.8 (d, *J* = 246.5 Hz), 141.0 (q, *J* = 35.7 Hz), 140.4 (d, *J* = 8.1 Hz), 139.9, 132.7 (d, *J* = 8.5 Hz), 125.8 (d, *J* = 3.2 Hz), 121.7 (q, *J* = 269.7 Hz), 117.3, 116.8 (d, *J* = 21.3 Hz), 112.8 (d, *J* = 21.3 Hz), 20.1 (d, *J* = 1.5 Hz), 9.5.

^{19}F NMR (377 MHz, CDCl$_3$): δ -60.87 (s, 3F), -114.48 (ddd, *J* = 9.7, 8.5, 5.9 Hz, 1F).

ν_{max} (ATR)/cm^{-1}: 3191 (br), 2951 (br), 1584, 1533, 1483, 1301, 1271, 1235, 1208, 1166, 1129, 1034, 991, 865, 821, 609.

HRMS calcd. for $C_{12}H_{10}F_4N_2$-H^+: 257.0707 [M-H]$^-$; found (ESI$^-$) 257.0702.

m.p./°C: 119-120.

4-(4-Fluoro-2-methylphenyl)-1,5-diphenyl-3-(trifluoromethyl)-1*H*-pyrazole (54)

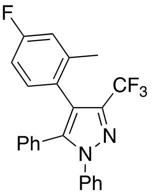

Synthesised according to **general procedure 2**. Using aryl bismacycle **2a** (267 mg, 0.500 mmol) and 4,4,4-trifluoro-1-phenylbutane-1,3-dione (108 mg, 0.500 mmol) afforded, after purification by preparative HPLC (50-100% MeCN in water), the title compound (146 mg, 0.368 mmol, 74%) as a yellow solid.

^{1}H NMR (400 MHz, $CDCl_3$): δ 7.41 – 7.29 (m, 5H), 7.25 – 7.21 (m, 2H), 7.21 – 7.09 (m, 2H), 7.01 – 6.75 (m, 4H), 1.96 (s, 3H).
^{13}C{^{1}H} NMR (101 MHz, $CDCl_3$): δ 162.7 (d, *J* = 246.5 Hz), 142.6, 141.5 (q, *J* = 36.0 Hz), 140.3 (d, *J* = 8.2 Hz), 139.4, 133.1 (d, *J* = 8.6 Hz), 129.6, 129.2, 128.9, 128.8, 128.6, 128.4, 125.9 (d, *J* = 3.2 Hz), 125.4, 121.6 (q, *J* = 270.2 Hz), 119.4, 116.7 (d, *J* = 21.2 Hz), 112.7 (d, *J* = 21.4 Hz), 20.3 (d, *J* = 1.6 Hz).
^{19}F NMR (377 MHz, $CDCl_3$): δ -60.80 (s, 3F), -114.40 (ddd, *J* = 9.5, 8.3, 5.9 Hz, 1F).
ν_{max} (ATR)/cm^{-1}: 1597, 1508, 1490, 1478, 1446, 1373, 1323, 1255, 1232, 1195, 1168, 1147, 1126, 1078, 988, 977, 828, 789, 767, 730, 697.
HRMS calcd. for $C_{23}H_{16}F_4N_2+H^+$: 397.1322 $[M+H]^+$; found (ESI^+) 397.1321.
m.p./°C: 114-115.

Methyl 2-(4-fluoro-2-methylphenyl)-3-oxobutanoate (55)

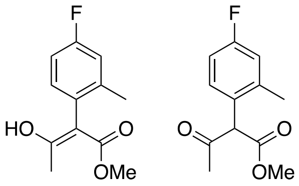

Synthesised according to **general procedure 1** (**X** = 60; **Y** = 2). Using 4-fluoro-2-methylphenylboronic acid (84.7, 0.550 mmol) and methyl acetoacetate (58.1 mg, 0.500 mmol) and the following workup: after cooling to room temperature, the reaction mixture was diluted with EtOAc (20 mL) and washed with sat. aq. $NaHCO_3$ solution (3 × 10 mL). The organic portion was dried over $MgSO_4$ and concentrated to dryness. Purification by silica gel column chromatography (0-2% EtOAc in cyclohexane) afforded the title compound (56.1 mg, 0.250 mmol, 50%) as a colourless oil.

Preparation according to ***general procedure 2*** *afforded the title compound in 8% yield, as determined by ^{19}F NMR spectroscopy (vs 4,4'-bis(trifluoromethyl)biphenyl).*

Observed as a 1:0.17 mixture of the enol and keto tautomers by NMR spectroscopy in $CDCl_3$. ^{a} Denotes the enol tautomer. ^{b} Denotes the keto tautomer.

^{1}H NMR (500 MHz, $CDCl_3$): δ 12.96 (s, 1.0H)^{a}, 7.24 (s, 0.17H)^{b}, 7.01 (dd, *J* = 8.4, 6.0 Hz, 1.0H)^{a}, 6.94 (dd, *J* = 9.7, 2.8 Hz, 1.3H)^{a,b}, 6.87 (app. td, *J* = 8.4, 2.8 Hz, 1.0H)^{a}, 4.87 (s, 0.16H)^{b}, 3.76 (s, 0.49H)^{b}, 3.68 (s, 3.0H)^{a}, 2.33 (s, 0.51H)^{b}, 2.17 (s, 0.51H)^{b}, 2.14 (s, 3.0H)^{a}, 1.75 (s, 3.0H)^{a}.

^{13}C{^{1}H} NMR (126 MHz, $CDCl_3$): δ 201.7^{b}, 174.2^{a}, 172.9^{a}, 169.3^{b}, 162.5 (d, *J* = 247.1 Hz)^{b}, 162.3 (d, *J* = 245.7 Hz)^{a}, 140.6 (d, *J* = 7.9 Hz)^{a}, 139.3 (d, *J* = 8.0 Hz)^{b}, 133.0 (d, *J* = 8.2 Hz)^{a}, 130.8 (d, *J* = 8.6 Hz)^{b}, 130.4 (d, *J* = 3.2 Hz)^{a}, 127.2 (d, *J* = 3.2 Hz)^{b}, 117.7 (d, *J* = 21.4 Hz)^{b}, 116.7 (d, *J* = 21.1 Hz)^{a}, 113.7 (d, *J* = 21.3 Hz)^{b}, 112.8 (d, *J* = 21.0 Hz)^{a}, 101.8^{a}, 61.4^{b}, 52.8^{b}, 52.0^{a}, 29.0^{b}, 20.1^{b}, 20.0 (d, *J* = 1.7 Hz)^{a}, 19.6^{a}.

^{19}F NMR (377 MHz, $CDCl_3$): δ -114.21 (app. td, *J* = 8.8, 5.6 Hz, 0.17F)^{b}, -115.37 (app. td, *J* = 9.1, 5.9 Hz, 1.0F)^{a}.

ν_{max} (ATR)/cm^{-1}: 2955, 2924, 2855, 1722, 1648, 1613, 1499, 1441, 1340, 1267, 1234, 1218, 1151, 1065, 982, 861, 822, 599.

HRMS calcd. for $C_{12}H_{13}FO_3$+Na^+: 247.0741 $[M+Na]^+$; found (ESI^+) 247.0751.

3-(4-Fluoro-2-methylphenyl)pentane-2,4-dione (56)

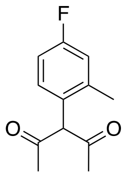

Synthesised according to **general procedure 1** (**X** = 60; **Y** = 2). Using 4-fluoro-2-methylphenylboronic acid (84.7, 0.550 mmol) and acetylacetone (50.1 mg, 0.500 mmol) and the following workup: after cooling to room temperature, the reaction mixture was diluted with EtOAc (20 mL) and washed with sat. aq. $NaHCO_3$ solution (3 × 10 mL). The organic portion was dried over $MgSO_4$ and concentrated to dryness. Purification by silica gel column chromatography (0-10% EtOAc in cyclohexane) afforded the title compound (63.0 mg, 0.303 mmol, 61%) as a colourless liquid.

*Preparation according to **general procedure 2** afforded the title compound in 44% yield, as determined by ^{19}F NMR spectroscopy (vs 4,4'-bis(trifluoromethyl)biphenyl).*

^{1}H NMR (400 MHz, $CDCl_3$): δ 7.06 (dd, *J* = 8.4, 6.0 Hz, 1H), 6.99 (dd, *J* = 9.5, 2.8 Hz, 1H), 6.91 (app. td, *J* = 8.4, 2.8 Hz, 1H), 2.17 (s, 3H), 1.80 (s, 6H).*
^{13}C{^{1}H} NMR (101 MHz, $CDCl_3$): δ 191.1, 162.5 (d, *J* = 246.6 Hz), 140.3 (d, *J* = 7.9 Hz), 133.1 (d, *J* = 8.4 Hz), 132.1 (d, *J* = 3.3 Hz), 117.2 (d, *J* = 21.1 Hz), 113.5 (d, *J* = 21.0 Hz), 112.7, 23.8, 20.2 (d, *J* = 1.7 Hz).
^{19}F NMR (377 MHz, $CDCl_3$): δ -114.73 (ddd, *J* = 9.5, 8.4, 6.0 Hz).
ν_{max} (ATR)/cm^{-1}: 2958, 2924, 1609, 1586, 1498, 1400, 1326, 1268, 1233, 1207, 1151, 994, 864, 820, 588.
HRMS calcd. for $C_{12}H_{13}FO_2+Na^+$: 231.0792 $[M+Na]^+$; found (ESI$^+$) 231.0799.
* α-proton could not be observed.

7. Derivatisations

2-(4-Fluoro-2-methylphenyl)-4,4-dimethylcyclopent-2-en-1-one (41)

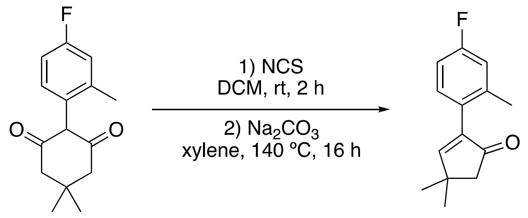

N-Chlorosuccinimide (108 mg, 0.800 mmol) was added to a solution of **4** (199 mg, 0.800 mmol) in anhydrous CH_2Cl_2 (8 mL). The mixture was stirred at room temperature for 2 h then water (10 mL) was added and the mixture extracted with CH_2Cl_2 (3 × 20 mL). The combined organic portions were dried over $MgSO_4$ and concentrated to dryness. The crude material was dissolved in xylene (10 mL) before Na_2CO_3 (93.3 mg, 0.880 mmol) was added and the reaction mixture was heated at reflux for 16 h. After cooling to room temperature, the mixture was washed with 1M HCl (3 × 5 mL), dried over $MgSO_4$ and concentrated to dryness. Purification by silica gel column chromatography (0-10% EtOAc in cyclohexane) afforded the title compound (157 mg, 0.719 mmol, 90%) as a colourless oil.

^{1}H NMR (400 MHz, $CDCl_3$): δ 7.33 (s, 1H), 7.12 (dd, *J* = 8.5, 6.0 Hz, 1H), 6.97 – 6.83 (m, 2H), 2.45 (s, 2H), 2.22 (s, 3H), 1.32 (s, 6H).

^{13}C{^{1}H} NMR (101 MHz, $CDCl_3$): δ 207.4, 170.8, 162.6 (d, *J* = 246.7 Hz), 142.1, 139.0 (d, *J* = 8.0 Hz), 131.1 (d, *J* = 8.4 Hz), 127.5 (d, *J* = 3.2 Hz), 117.1 (d, *J* = 21.2 Hz), 112.7 (d, *J* = 21.1 Hz), 50.6, 39.2, 28.5, 20.5 (d, *J* = 1.7 Hz).

^{19}F NMR (377 MHz, $CDCl_3$): δ -114.54 (ddd, *J* = 9.6, 8.4, 6.0 Hz).

ν_{max} (ATR)/cm^{-1}: 2959, 2926, 2868, 1709, 1608, 1585, 1495, 1410, 1310, 1273, 1249, 1235, 1153, 1125, 1094, 965, 862, 814, 613.

HRMS calcd. for $C_{14}H_{15}FO+H^+$: 219.1180 $[M+H]^+$; found (ESI$^+$) 219.1191.

Ethyl 6-(4-fluoro-2-methylphenyl)-5-oxohexanoate (42)

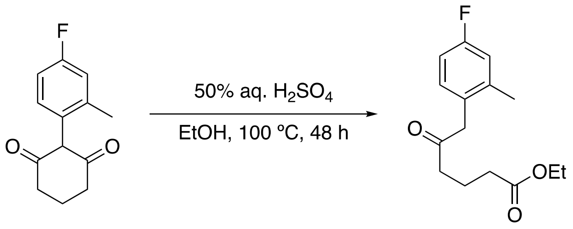

A solution of **6** (132 mg, 0.600 mmol) in EtOH (5 mL) and 50% aq. H_2SO_4 (1 mL) was heated at reflux for 48 h then cooled to room temperature. The reaction mixture was diluted with EtOAc (20 mL), washed with water (3 × 10 mL), dried over $MgSO_4$ and concentrated to dryness. Purification by silica gel column chromatography (0-10% EtOAc in cyclohexane) afforded the title compound (112 mg, 0.421 mmol, 70%) as a colourless oil.

^{1}H NMR (400 MHz, CDCl$_3$): δ 7.06 (dd, *J* = 8.4, 5.8 Hz, 1H), 6.98 – 6.78 (m, 2H), 4.10 (q, *J* = 7.1 Hz, 2H), 3.66 (s, 2H), 2.50 (t, *J* = 7.1 Hz, 2H), 2.29 (t, *J* = 7.2 Hz, 2H), 2.21 (s, 3H), 1.88 (app. p, *J* = 7.2 Hz, 2H), 1.23 (t, *J* = 7.1 Hz, 3H).

^{13}C{^{1}H} NMR (101 MHz, CDCl$_3$): δ 207.4, 173.2, 162.0 (d, *J* = 245.1 Hz), 139.3 (d, *J* = 7.7 Hz), 131.8 (d, *J* = 8.3 Hz), 128.9 (d, *J* = 3.1 Hz), 117.3 (d, *J* = 21.1 Hz), 113.0 (d, *J* = 21.1 Hz), 60.5, 47.5, 40.9, 33.3, 20.0 (d, *J* = 1.6 Hz), 19.0, 14.4.

^{19}F NMR (377 MHz, CDCl$_3$): δ -116.11 (app. td, *J* = 9.0, 5.8 Hz).

ν_{max} (ATR)/cm^{-1}: 2981, 2937, 2908, 2876, 1728, 1591, 1498, 1447, 1423, 1375, 1315, 1252, 1179, 1152, 1094, 1033, 957, 863, 814.

HRMS calcd. for $C_{15}H_{19}FO_3$+Na$^+$: 289.1210 [M+Na]$^+$; found (ESI$^+$) 289.1214.

4'-Fluoro-2'-methyl-[1,1'-biphenyl]-2,6-diol (43)

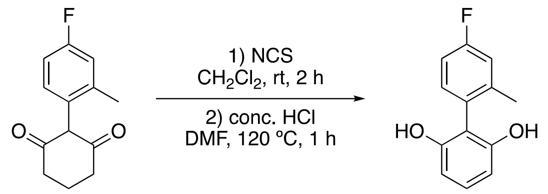

N-Chlorosuccinimide (108 mg, 0.800 mmol) was added to a solution of **6** (176 mg, 0.800 mmol) in anhydrous CH_2Cl_2 (8 mL). The mixture was stirred at room temperature for 2 h and then concentrated to dryness. The crude material was dissolved in DMF (8 mL) before conc. HCl (2.9 mL) was added and the reaction mixture heated at reflux for 1 h. After cooling to room temperature, the mixture was diluted with EtOAc (20 mL), washed with water (3 × 10 mL), dried over $MgSO_4$ and concentrated to dryness. Purification by silica gel column chromatography (0-20% EtOAc in cyclohexane) afforded the title compound (143 mg, 0.656 mmol, 82%) as a colourless oil. Characterisation data were consistent with literature values.[8]

^{1}H NMR (400 MHz, $CDCl_3$): δ 7.32 – 7.20 (m, 1H), 7.22 – 7.11 (m, 2H), 7.07 (app. td, J = 8.3, 2.8 Hz, 1H), 6.58 (d, J = 8.2 Hz, 2H), 4.55 (br s, 2H), 2.16 (s, 3H).

^{13}C{^{1}H} NMR (101 MHz, $CDCl_3$): δ 163.5 (d, J = 249.0 Hz), 153.6, 142.4 (d, J = 8.1 Hz), 133.4 (d, J = 8.7 Hz), 129.9, 125.4 (d, J = 3.0 Hz), 118.4 (d, J = 21.1 Hz), 114.5 (d, J = 21.4 Hz), 113.7, 107.7, 19.8 (d, J = 1.7 Hz).

^{19}F NMR (377 MHz, $CDCl_3$): δ -112.08 (ddd, J = 9.7, 8.3, 5.9 Hz).

ν_{max} (ATR)/cm^{-1}: 3515, 3405 (br), 2958, 2924, 2856, 1611, 1584, 1489, 1461, 1309, 1271, 1229, 1174, 1150, 1004, 866, 820, 788, 738.

HRMS calcd. for $C_{13}H_{11}FO_2$-H^+: 217.0670 [M-H]$^-$; found (ESI$^-$) 217.0665.

8. Robustness Screen

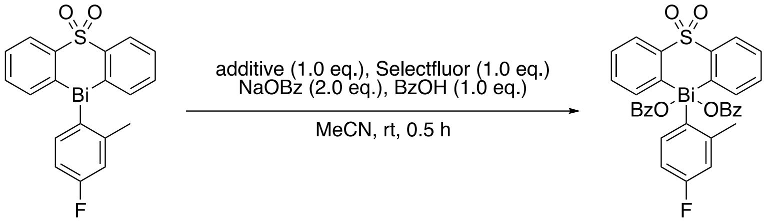

2 (1.0 eq.) → **3d**

Entry	Additive	3d (%)	Additive remaining (%)
1	none	85	-
2	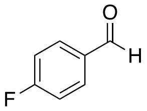	78	93
3	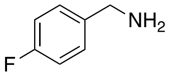	35	59
4	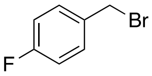	75	94
5	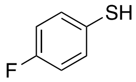	35	0
6	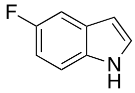	8	34
7	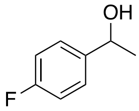	38	49
8	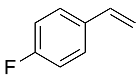	84	98
9	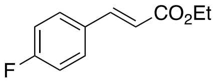	81	98
10	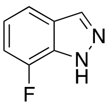	82	97
11	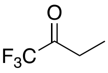	74	98

Supplementary Table 11. Robustness screen of Bi(III) → Bi(V) oxidation. Yields of **3d** and remaining additive determined by ^{19}F NMR spectroscopy against an internal standard.

Bismuth stock solution: A solution of aryl bismacycle **2** (128 mg, 0.240 mmol) and 4,4'-bis(trifluoromethyl)-1,1'-biphenyl (internal standard for ^{19}F NMR spectroscopy) in CH_2Cl_2 (3 mL) was prepared. An aliquot was analysed by quantitative ^{19}F NMR spectroscopy to confirm the initial bismacycle : internal standard ratio.

Additive stock solution: A solution of the additive (0.080 mmol) and 1,3-bis(trifluoromethyl)-5-bromobenzene (internal standard for ^{19}F NMR spectroscopy) in MeCN (1 mL) was prepared. An aliquot was analysed by quantitative ^{19}F NMR spectroscopy to confirm the initial additive : internal standard ratio.

Procedure: An aliquot of the bismuth stock solution (0.25 mL of a 0.08 M solution, 0.020 mmol) was added to an NMR tube and concentrated to dryness. Selectfluor (7.09 mg, 0.020 mmol), sodium benzoate (5.76 mg, 0.040 mmol) and benzoic acid (2.44 mg, 0.020 mmol) were charged to the NMR tube followed by an aliquot of the additive stock solution (0.25 mL of a 0.08 M solution, 0.020 mmol) and CD_3CN (0.25 mL). The mixture was mixed for 0.5 h then analysed by quantitative ^{19}F NMR spectroscopy.

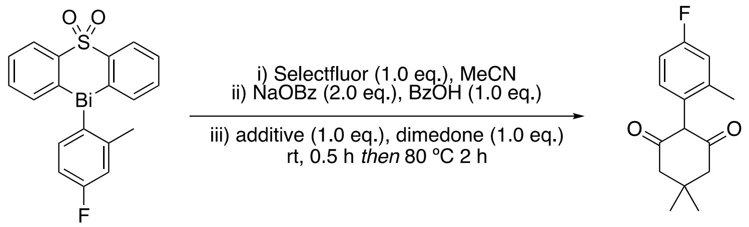

2 (1.0 eq.) → **4**

Entry	Additive	4 (%)	Additive remaining (%)
1	none	72	-
2	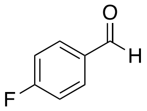	71	89
3	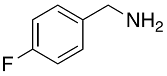	71	63
4	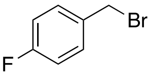	63	14
5	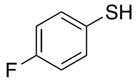	18	0
6	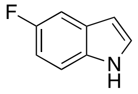	71	99
7	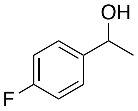	69	98
8	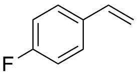	72	98
9	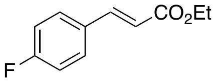	69	100
10	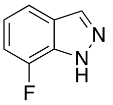	70	100
11	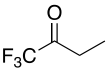	70	100

Supplementary Table 12. Robustness screen of Bi(V)-mediated arylation of dimedone. Yields of **4** and remaining additive determined by ^{19}F NMR spectroscopy against an internal standard.

Bismuth stock solution: A solution of aryl bismacycle **2** (150 mg, 0.280 mmol), Selectfluor (99.2 mg, 0.280 mmol) and 4,4'-bis(trifluoromethyl)-1,1'-biphenyl (internal standard for ^{19}F NMR spectroscopy) in MeCN (3.5 mL) was mixed for 15 mins. An aliquot was analysed by quantitative ^{19}F NMR spectroscopy to confirm the initial bismacycle : internal standard ratio.

Additive stock solution: A solution of the additive (0.080 mmol) and 1,3-bis(trifluoromethyl)-5-bromobenzene (internal standard for ^{19}F NMR spectroscopy) in MeCN (1 mL) was prepared. An aliquot was taken and analysed by quantitative ^{19}F NMR spectroscopy to confirm the initial additive : internal standard ratio.

Procedure: An aliquot of the bismuth stock solution (0.25 mL of a 0.08 M solution, 0.020 mmol) was added to an NMR tube followed by sodium benzoate (5.76 mg, 0.040 mmol) and benzoic acid (2.44 mg, 0.020 mmol). The mixture was mixed for 15 mins before an aliquot of the additive stock solution (0.25 mL of a 0.08 M solution, 0.020 mmol) was added followed by dimedone (2.80 mg, 0.020 mmol). After mixing at room temperature for 0.5 h, the mixture was heated at 80 °C for 2 h then analysed by quantitative ^{19}F NMR spectroscopy.

9. Comparison to Pd- and Cu-Catalysis

To illustrate the challenges associated with Pd- and Cu-catalysed strategies for couplings of diones with *ortho*-substituted aryl partners, we have applied leading methods from Buchwald[9] and Jiang[10] to representative compounds from our substrate scope (Supplementary Table 13, entries 1-3; SI Sections 9-*i* and 9-*ii*).

In addition, we have repeated as a benchmark the most common Cu-catalysed coupling between an *ortho*-substituted aryl iodide and a cyclic dione (*i.e.*, between 2-iodotoluene and 1,3-cyclohexanedione) following Dong's[13] / Jiang's[10] method. The yield we obtain is comparable to literature results (Supplementary Table 13, entry 4): 22%, *vs* 20%,[11] 24%[12] or 10% (when telescoped with an additional allylation step).[13] In contrast, the same compound **S6** is prepared in 81% isolated yield using our bismuth methodology (SI Section 9-*iii*). This further highlights the utility of our methodology, and the limitations associated with extant dione arylation protocols.

Entry	Product		% Yield	
		Pd-catalysed	Cu-catalysed	Bi-mediated
	Conditions:	1.0 eq. ArBr, 1.3 eq. dione, 1.0 mol% $Pd(OAc)_2$, 2.2 mol% ^{t}Bu-MePhos, 2.3 eq. K_3PO_4, THF, 80 °C, 24 h	1.0 eq. ArI, 3 eq. dione, 10 mol% CuI, 20 mol% proline, 4.0 eq. K_2CO_3, DMSO, 90 °C, 24 h	As per manuscript Schemes 3 (**4**, **34** and **S6**) and 5 (**44**)
1	**4** 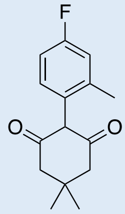	not observed	13	81
2	**34** 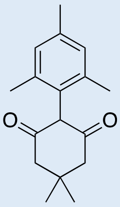	not observed	< 5	66 *
3	**44** 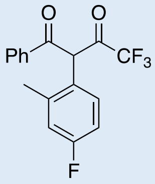	not observed	< 5	80
4	**S6** 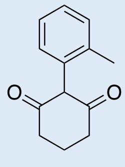	-	22 §<br>20[11] *<br>24[12] *,§	81 *

Supplementary Table 13. Comparison of Pd-, Cu- and Bi-mediated arylation methods. Yields determined by NMR spectroscopy against an internal standard. * Yield of isolated material following purification. § Using 5 mol% CuI and 10 mol% *L*-proline for 48 h.

i. Procedure for Pd-Catalysed Arylation[9]

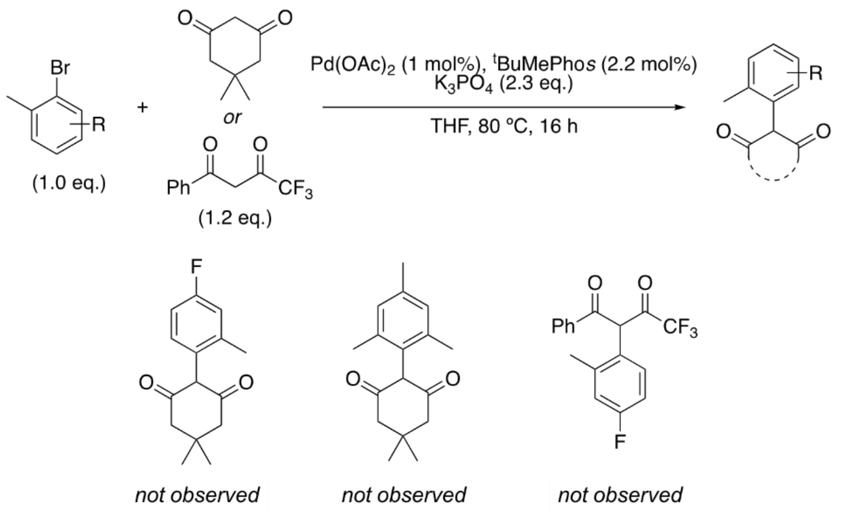

Procedure: The aryl bromide (1.00 mmol) was added to a mixture of $Pd(OAc)_2$ (2.25 mg, 0.010 mmol), ^{t}BuMePhos (6.87 mg, 0.022 mmol), K_3PO_4 (488 mg, 2.30 mmol) and 1,3-dione substrate (1.20 mmol) in anhydrous THF (3 mL) under an atmosphere of N_2. The reaction mixture was stirred at 80 ˚C for 16 h and then cooled to room temperature, diluted with MeOH and filtered through celite.

Arylation with 5-fluoro-2-bromotoluene: 4,4'-Bis(trifluoromethyl)-1,1'-biphenyl (internal standard for ^{19}F NMR spectroscopy) was added and the organic portion was analysed by quantitative ^{19}F NMR spectroscopy.

Arylation with 2-bromomesitylene: The filtrate was concentrated to dryness. 1,3,5-Trimethoxybenzene (internal standard) was added, and the crude material was dissolved in $CDCl_3$ and analysed by ^{1}H NMR spectroscopy.

ii. Procedure for Cu-Catalysed Arylation[10]

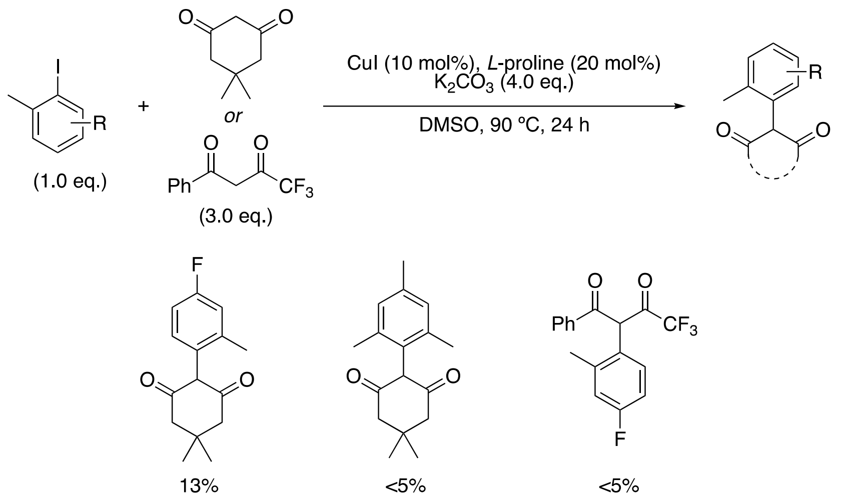

Procedure: The aryl iodide (0.500 mmol) was added to a solution of CuI (9.52 mg, 0.050 mmol), *L*-proline (11.5 mg, 0.100 mmol), K_2CO_3 (276 mg, 2.00 mmol) and 1,3-dione substrate (1.50 mmol) in anhydrous DMSO (2 ml) under an atmosphere of N_2. The reaction mixture was stirred at 90 ˚C for 24 h and then cooled to room temperature.

Arylation with 5-fluoro-2-iodotoluene: 4,4'-Bis(trifluoromethyl)-1,1'-biphenyl (internal standard for ^{19}F NMR spectroscopy) was added followed by 2 M HCl (3 mL) and EtOAc (4 mL) and the reaction mixture was stirred vigorously for 5 mins. The organic portion was analysed by quantitative ^{19}F NMR spectroscopy.

Arylation with 2-iodomesitylene: The reaction mixture was poured onto 2 M HCl (10 mL) and then extracted with EtOAc (3 × 10 mL). The combined organic portions were washed with brine, dried over $MgSO_4$ and concentrated to dryness. 1,3,5-Trimethoxybenzene (internal standard) was added, and the crude material was dissolved in $CDCl_3$ and analysed by ^{1}H NMR spectroscopy.

iii. Synthesis of 2-(*ortho*-Tolyl)-1,3-cyclohexanedione (S6)

Cu-Catalysed Arylation – Synthesis of (S6)

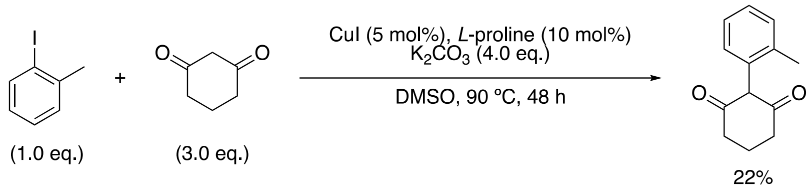

Procedure: 2-Iodotoluene (127 μL, 1.00 mmol) was added to a solution of CuI (9.52 mg, 0.05 mmol), *L*-proline (11.5 mg, 0.100 mmol), K_2CO_3 (553 mg, 4.00 mmol) and 1,3-cyclohexanedione (336 mg, 3.00 mmol) in anhydrous DMSO (4 ml) under an atmosphere of N_2. The reaction mixture was stirred at 90 ˚C for 48 h and then cooled to room temperature before pouring onto 1 M HCl. The organic layer was extracted with EtOAc (3 × 20 mL) and the combined organic portions were washed with brine, dried over $MgSO_4$ and concentrated to dryness. 1,3,5-Trimethoxybenzene (internal standard) was added and the crude material was dissolved in $CDCl_3$ and analysed by ^{1}H NMR spectroscopy.

Bi-Mediated Arylation – Synthesis of (S6)

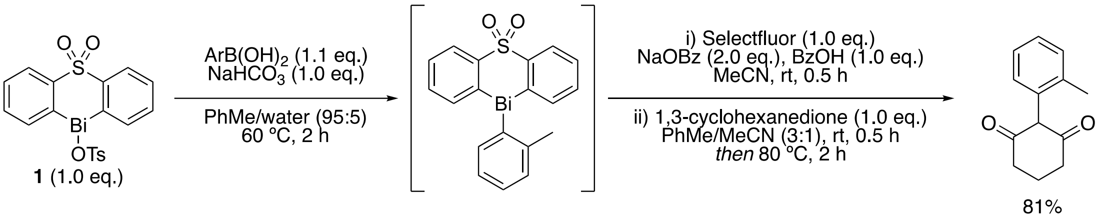

Procedure: According to *General Procedure 1*, a suspension of bismacycle tosylate **1** (296 mg, 1.00 mmol), *o*-tolylphenylboronic acid (150 mg, 1.10 mmol) and $NaHCO_3$ (84.0 mg, 1.00 mmol) in toluene (10 mL) and water (0.2 mL) was heated at 60 °C for 2 h before concentrating to dryness. Selectfluor (354 mg, 1.00 mmol), sodium benzoate (288 mg, 2.00 mmol) and benzoic acid (122 mg, 1.00 mmol) were added, followed by MeCN (5 mL) and the reaction mixture was stirred at room temperature for 0.5 h before diluting with toluene (15 mL). 1,3-Cyclohexanedione (112 mg, 1.00 mmol) was added and the reaction mixture was stirred at room temperature for 0.5 h then heated at 80 °C for 2 h. After cooling to room temperature, the reaction mixture was diluted with EtOAc (20 mL) and extracted with sat. aq. $NaHCO_3$ solution (3 × 10 mL). The combined aqueous portions were acidified to *p*H 1 with 2 M HCl then extracted with EtOAc (3 × 20 mL). The combined organic portions were dried over $MgSO_4$

and concentrated to dryness. Purification by silica gel column chromatography (15-40% EtOAc in cyclohexane) afforded the title compound (164 mg, 0.811 mmol, 81%) as a colourless solid.

^{1}H NMR (400 MHz, $CDCl_3$): δ 7.35 – 7.18 (m, 3H), 7.10 – 7.01 (m, 1H), 5,76 (s, 1H), 2.55 (t, *J* = 6.5 Hz, 4H), 2.18 – 2.05 (m, 5H).

^{13}C{^{1}H} NMR (101 MHz, $CDCl_3$): δ 197.0, 171.1, 138.9, 131.2, 131.1, 130.0, 129.1, 126.8, 117.8, 37.0, 27.9, 20.8, 19.6.

ν_{max} (ATR)/cm^{-1}: 3059, 2949, 2674, 1714, 1607, 1580, 1488, 1454, 1424, 1355, 1333, 1275, 1233, 1212, 1196, 1163, 1137, 1113, 1069, 985, 912, 756, 726, 592, 455.

HRMS calcd. for $C_{13}H_{14}O_2$-H^+: 201.0921 [M-H]$^-$; found (ESI$^-$) 201.0929.

m.p./°C: 140-141.

10. X-Ray Diffraction Data Tables

Supplementary Table 14. Crystal data and structure refinement for **3b** (CCDC 2171727).

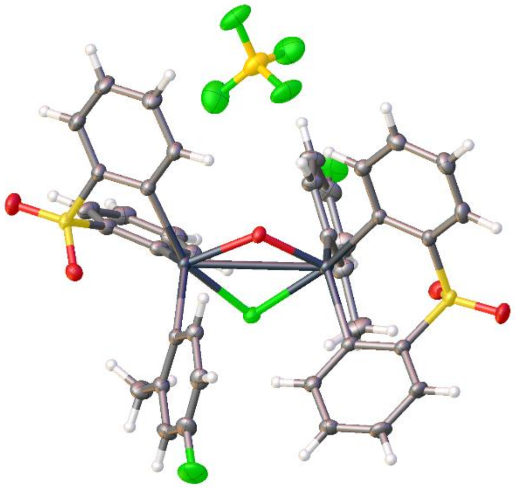

Empirical formula	$C_{38}H_{28}BBi_2F_7O_5S_2$
Formula weight	1190.49
Temperature/K	120(2)
Crystal system	monoclinic
Space group	$P2_1/n$
a/Å	12.34644(19)
b/Å	13.8717(2)
c/Å	21.8775(4)
α/°	90
β/°	90.9321(14)
γ/°	90
Volume/Å^3	3746.38(11)
Z	4
ρ_{calc}g/cm^3	2.111
μ/mm^{-1}	9.572
F(000)	2248.0
Crystal size/mm^3	0.219 × 0.186 × 0.096
Radiation	Mo Kα (λ = 0.71073)
2Θ range for data collection/°	5.744 to 70.418
Index ranges	$-19 \le h \le 19$, $-21 \le k \le 21$, $-35 \le l \le 35$
Reflections collected	113370
Independent reflections	16093 [R_{int} = 0.0401, R_{sigma} = 0.0260]
Data/restraints/parameters	16093/0/498
Goodness-of-fit on F^2	1.092
Final R indexes [I>=2σ (I)]	R_1 = 0.0242, wR_2 = 0.0471
Final R indexes [all data]	R_1 = 0.0377, wR_2 = 0.0519
Largest diff. peak/hole / e Å^{-3}	2.17/-1.31

11. References

1 O. V Dolomanov, L. J. Bourhis, R. J. Gildea, J. A. K. Howard and H. Puschmann, *J. Appl. Crystallogr.*, 2009, **42**, 339–341.

2 G. M. Sheldrick, *Acta Crystallogr. Sect. C*, 2015, **C71**, 3–8.

3 P. Petiot and A. Gagnon, *Eur. J. Org. Chem.*, 2013, 5282–5289.

4 M. Jurrat, L. Maggi, W. Lewis and L. T. Ball, *Nature Chem.*, 2020, **12**, 260–269.

5 SYNGENTA LTD, C. J. Mathews, J. Finney, J. N. Scutt, L. Robinson, J. S. Delaney, CYCLOPENTADIONE DERIVED HERBICIDES, WO2010102848 (A1), 2010.

6 WO2019/175117, 2019.

7 O. Planas, F. Wang, M. Leutzsch and J. Cornella, *Science*, 2020, **367**, 313–317.

8 E. S. Munday, M. A. Grove, T. Feoktistova, A. C. Brueckner, D. M. Walden, C. M. Young, A. M. Z. Slawin, A. D. Campbell, P. H. Cheong and A. D. Smith, *Angew. Chem. Int. Ed.*, 2020, **59**, 7897–7905.

9 J. M. Fox, X. Huang, A. Chieffi and S. L. Buchwald, *J. Am. Chem. Soc.*, 2000, **122**, 1360–1370.

10 Y. Jiang, N. Wu, H. Wu and M. He, *Synlett*, 2005, 2731–2734.

11 S. Reddy Chidipudi, M. D. Wieczysty, I. Khan and H. W. Lam, *Org. Lett.*, 2013, **15**, 570–573.

12 Y. Wada, R. Murata, Y. Fujii, K. Asano and S. Matsubara, *Org. Lett.*, 2020, **22**, 4710–4715.

13 X. Wu, Z. Chen, Y.-B. Bai and V. M. Dong, *J. Am. Chem. Soc.*, 2016, **138**, 12013–12016.

12. NMR Spectra

S1 - ^{1}H NMR (500 MHz, $CDCl_3$):

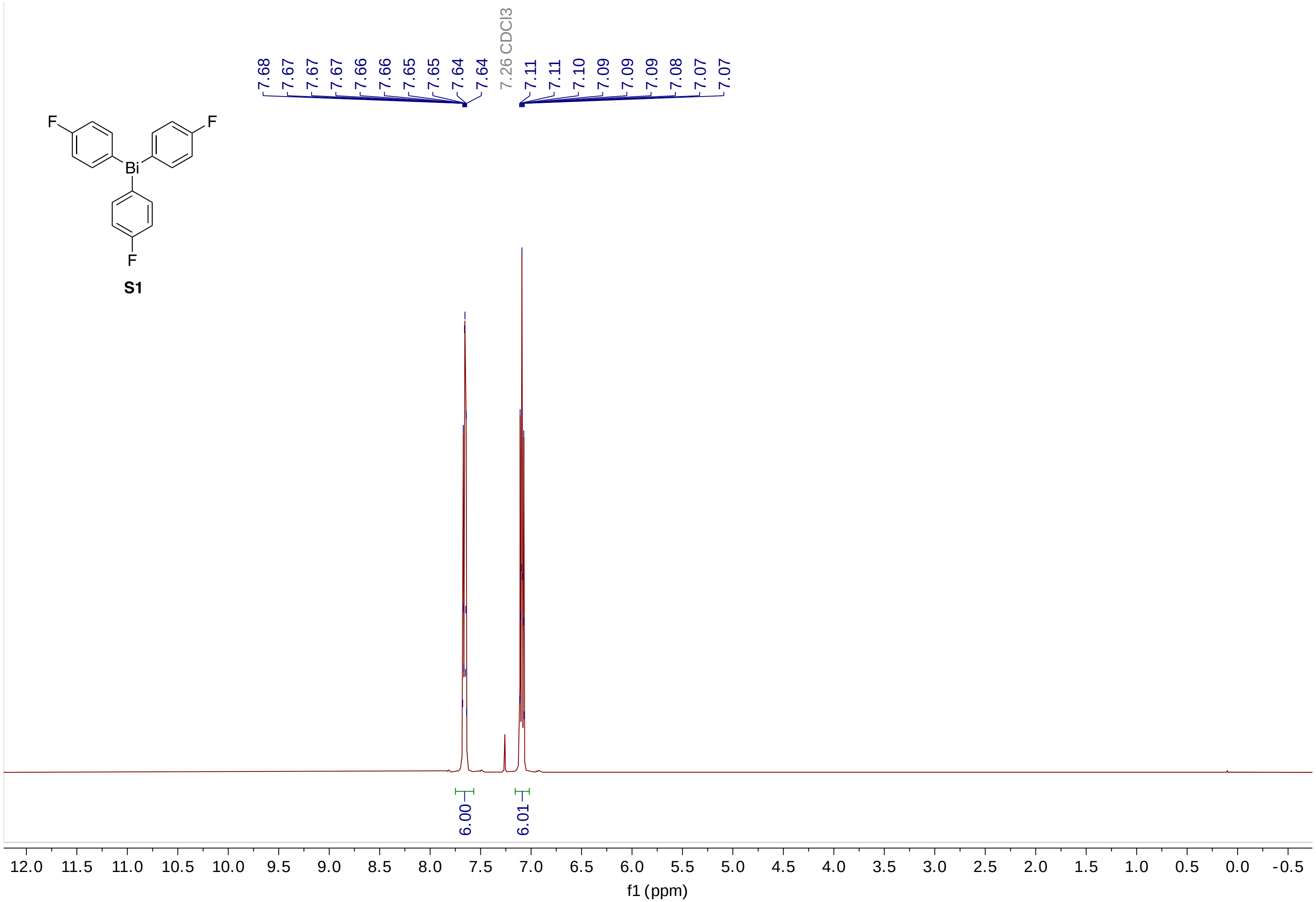

S1 - $^{13}C\{^{1}H\}$ NMR (126 MHz, CDCl$_3$):

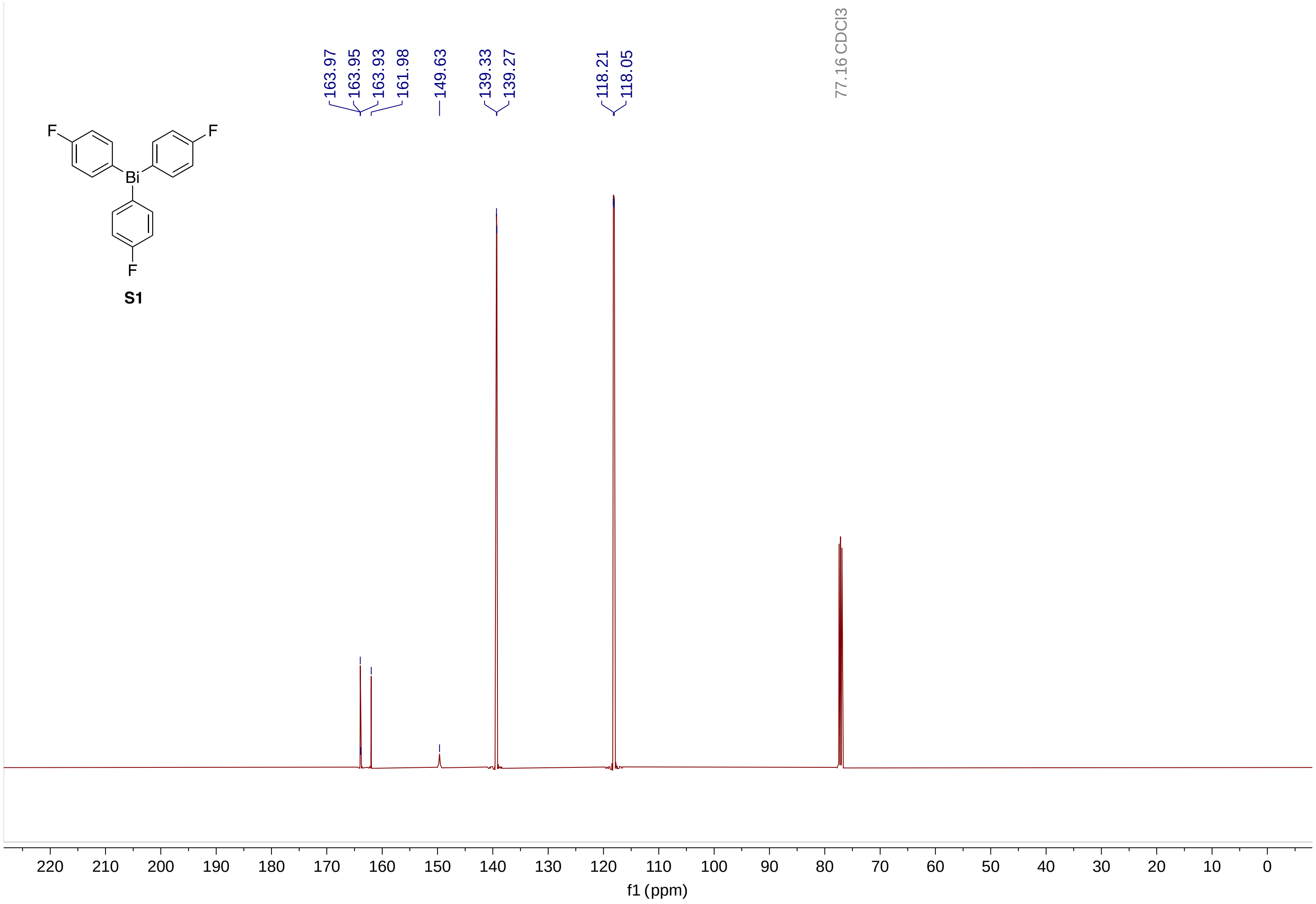

S1 - ^{19}F NMR (471 MHz, $CDCl_3$):

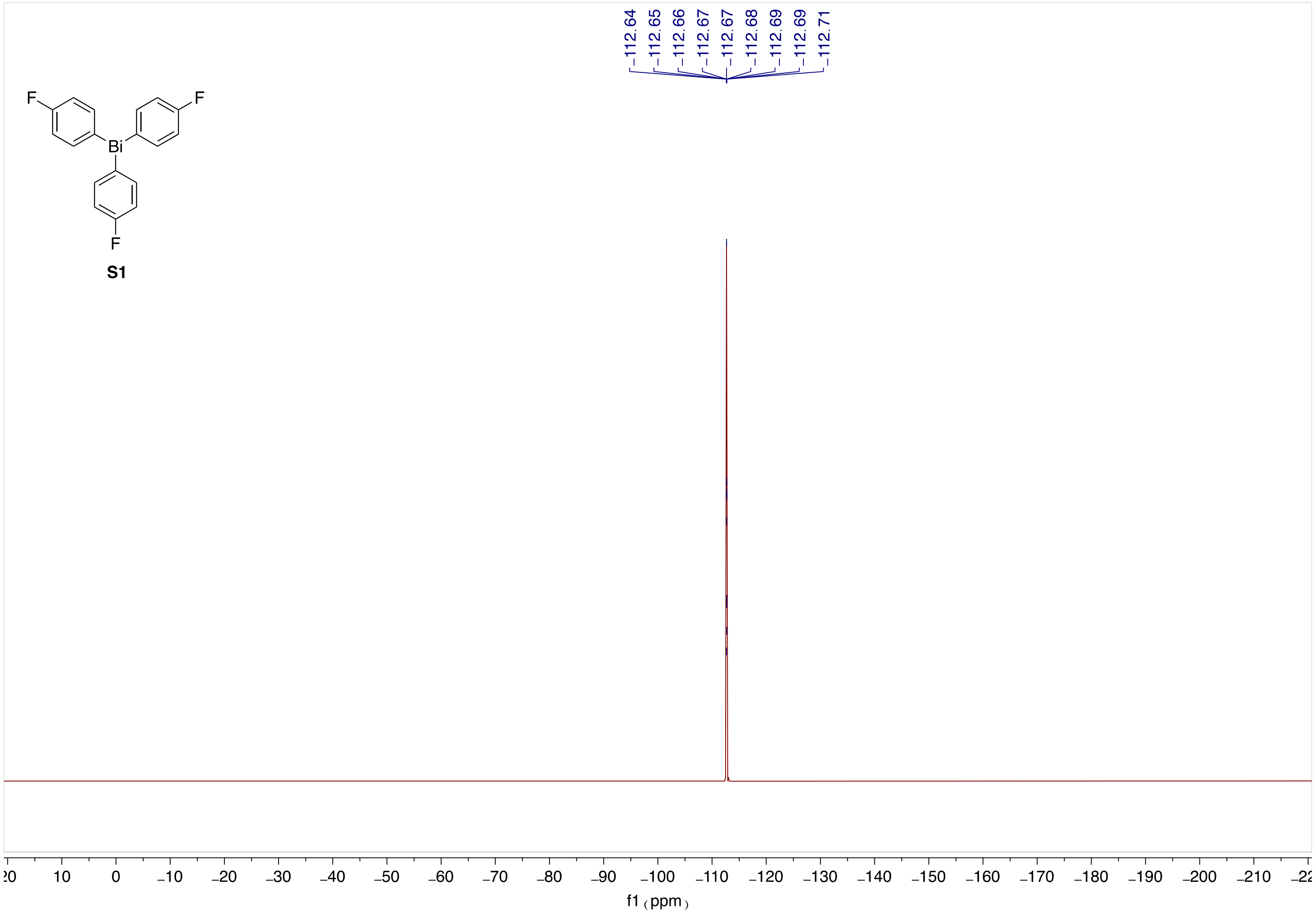

1 - ^{1}H NMR (400 MHz, $CDCl_3$):

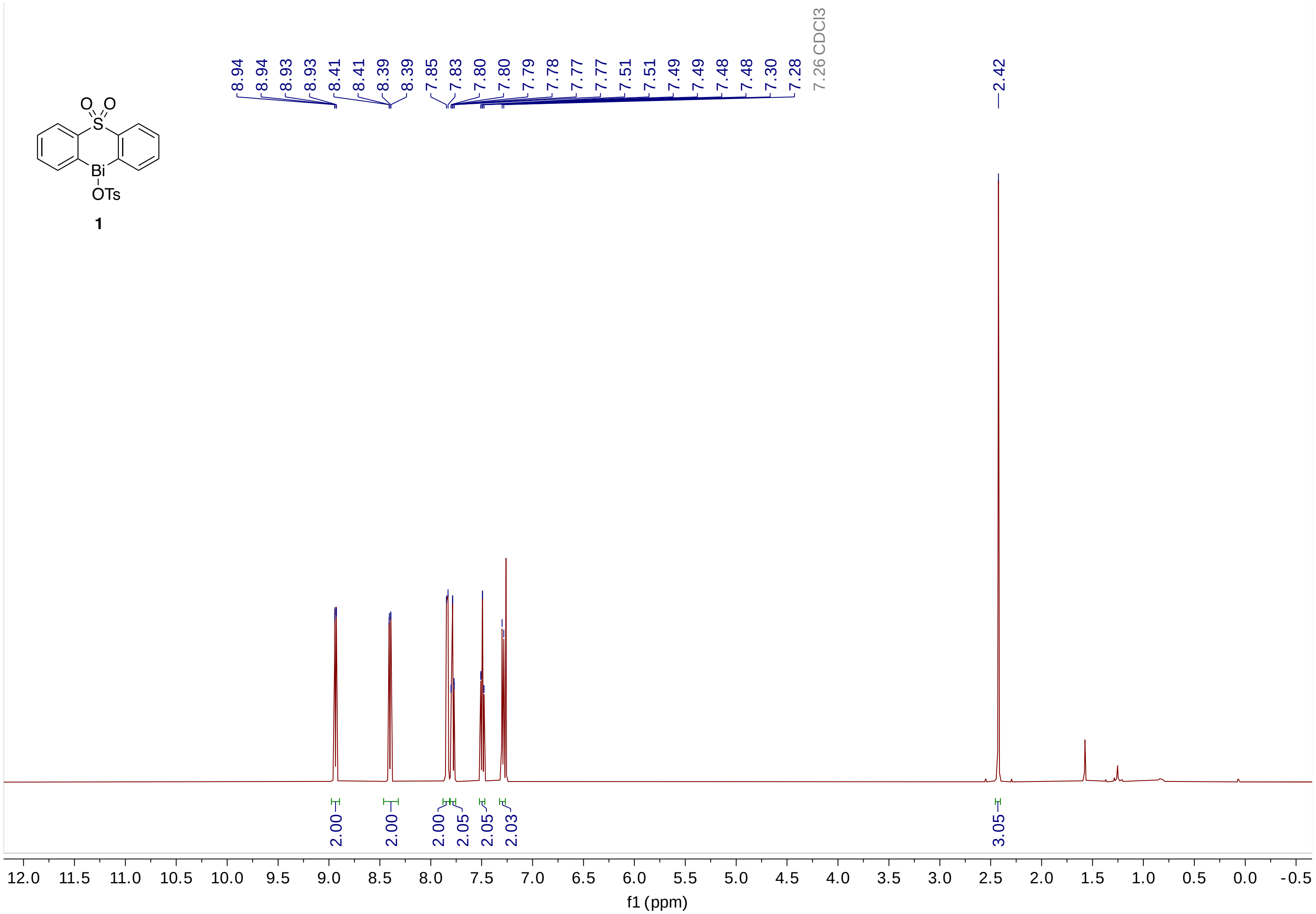

1 - $^{13}C\{^{1}H\}$ NMR (126 MHz, CDCl$_3$):

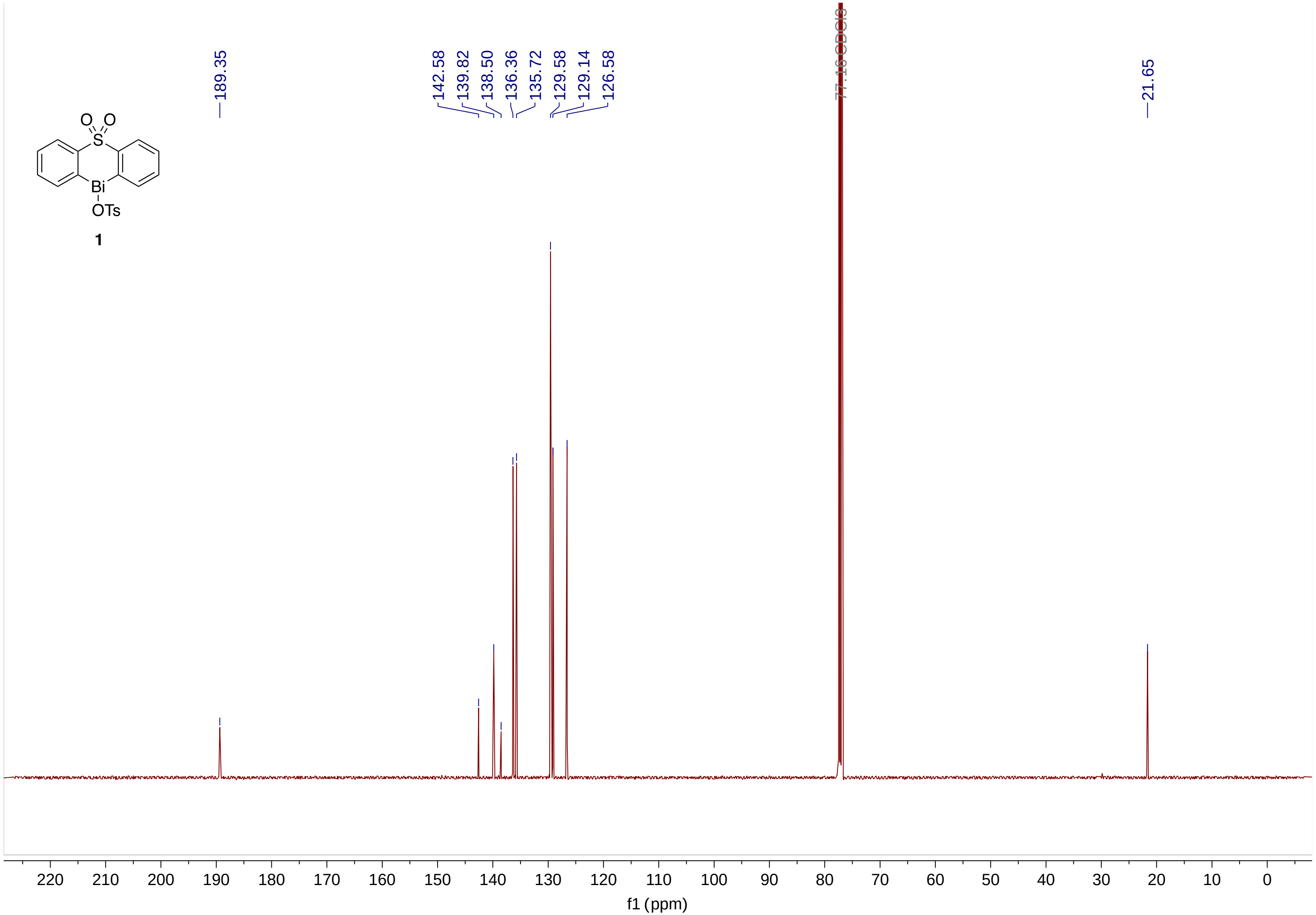

5,5-Dioxido-10H-dibenzo[b,e][1,4]thiabismin-10-yl benzoate - ^{1}H NMR (400 MHz, $CDCl_3$):

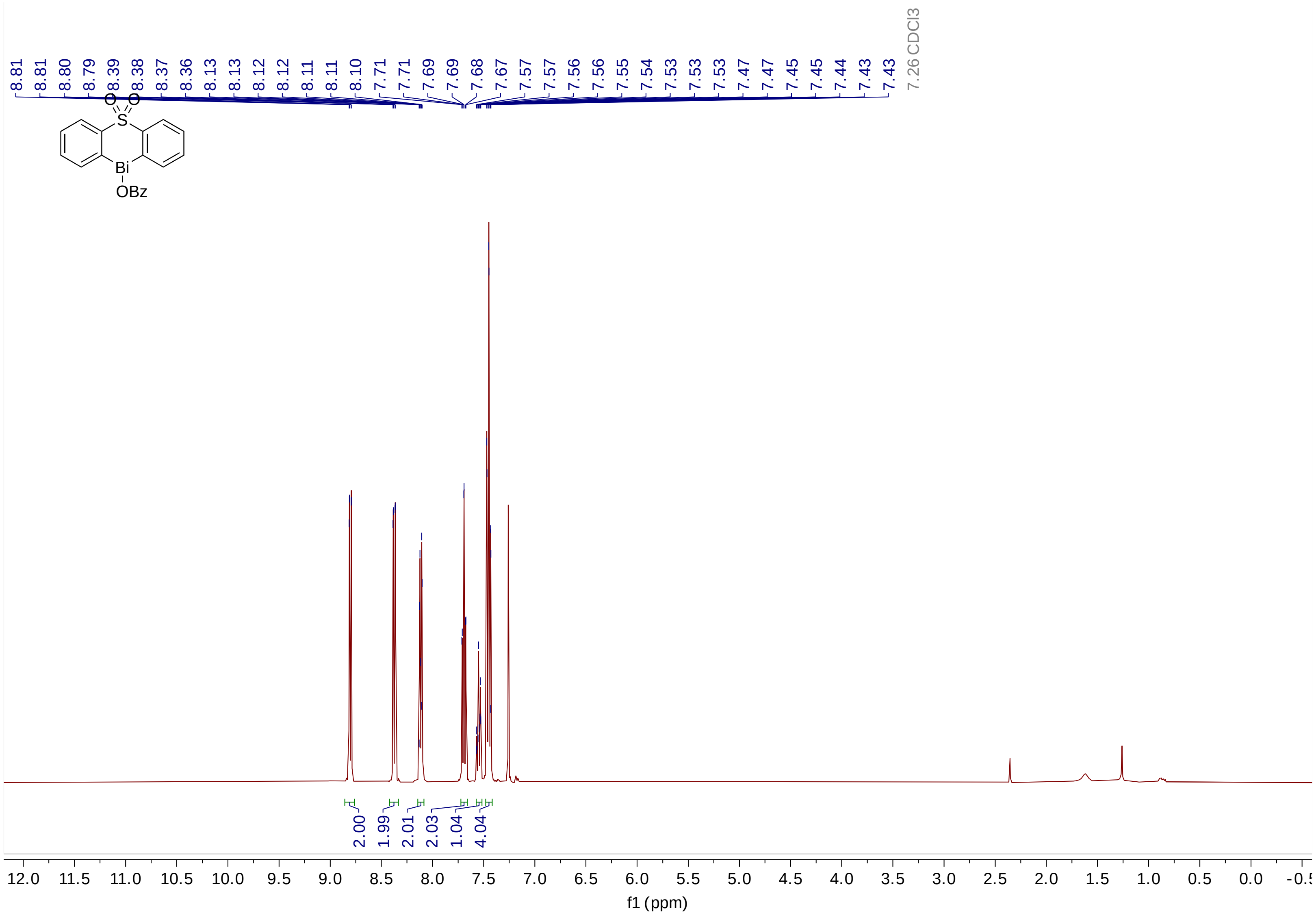

5,5-Dioxido-10H-dibenzo[b,e][1,4]thiabismin-10-yl benzoate - ^{13}C NMR (101 MHz, $CDCl_3$):

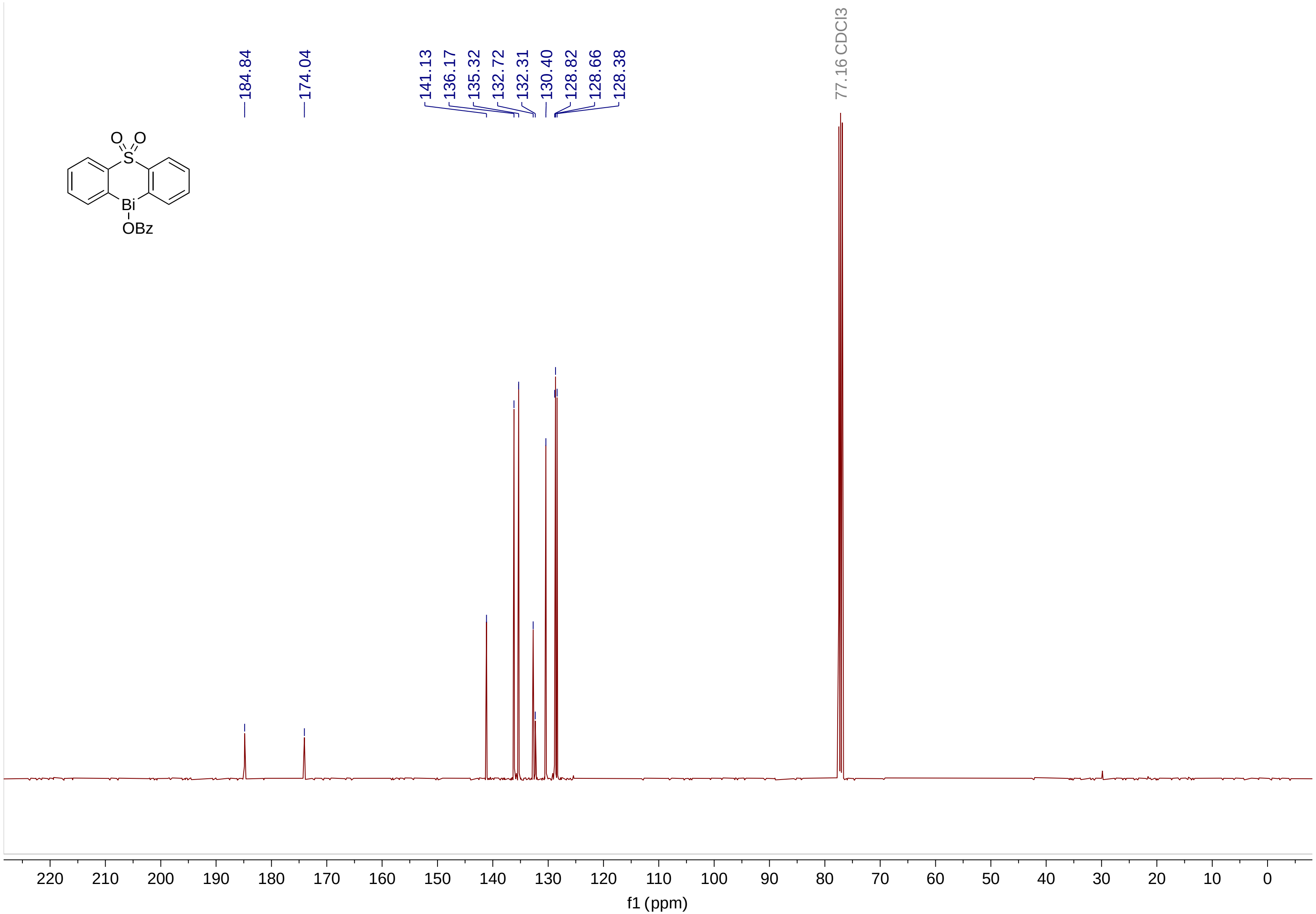

2a - ^{1}H NMR (500 MHz, $CDCl_3$):

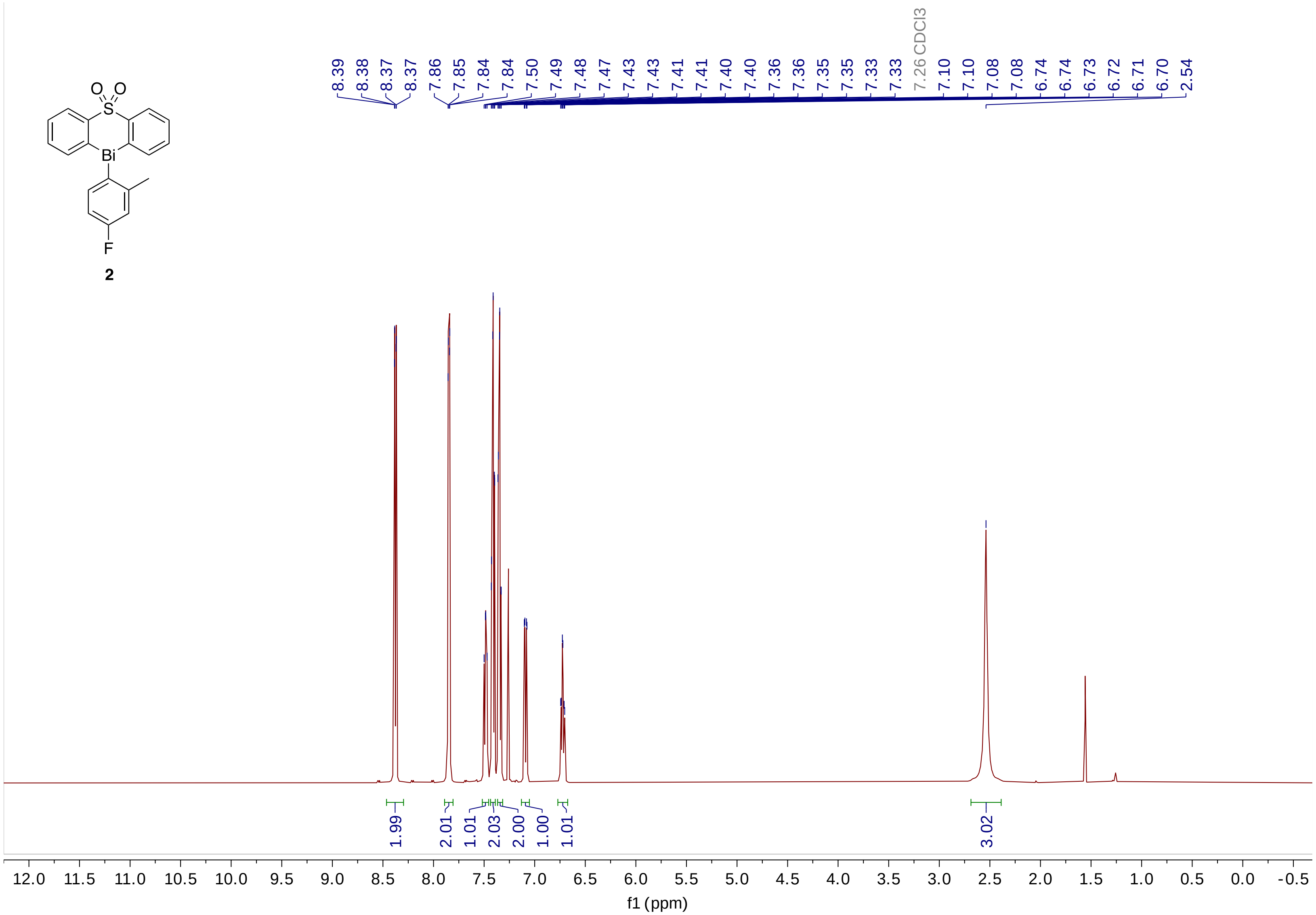

2a - $^{13}C\{^{1}H\}$ NMR (126 MHz, $CDCl_3$):

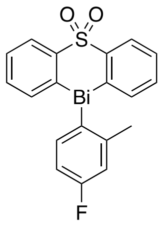

2

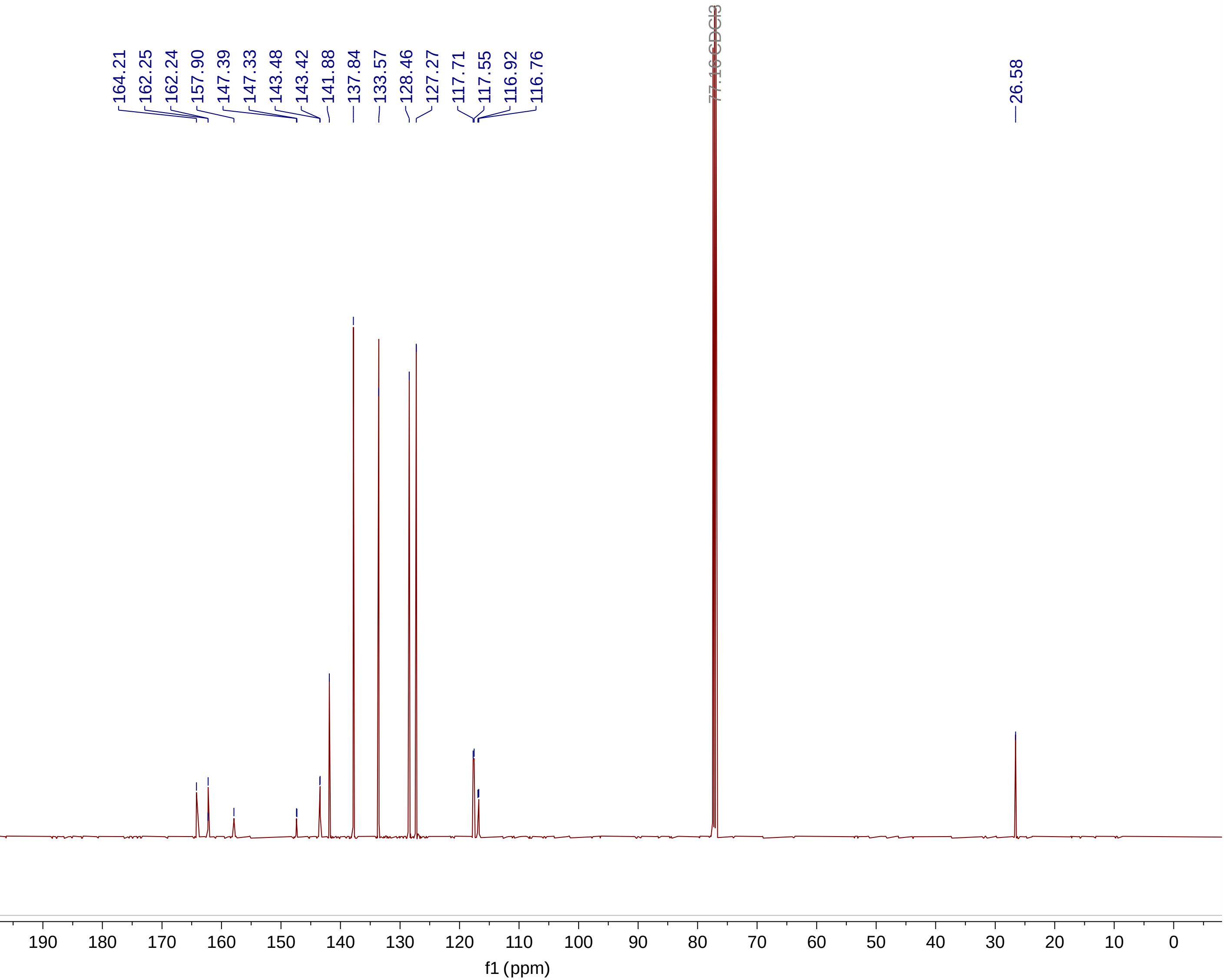

2a - ^{19}F NMR (471 MHz, CDCl$_3$):

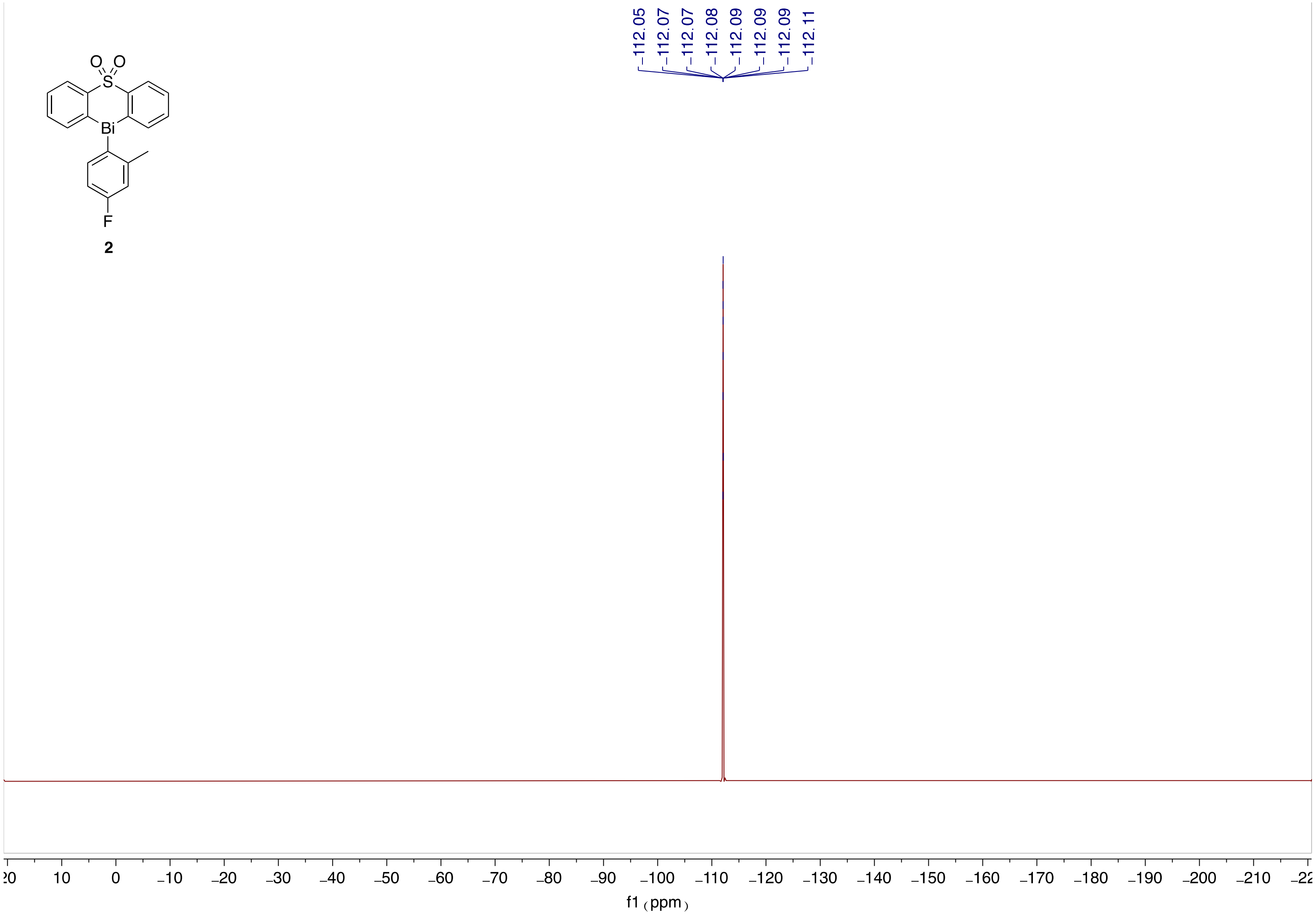

2b - ^{1}H NMR (400 MHz, $CDCl_3$):

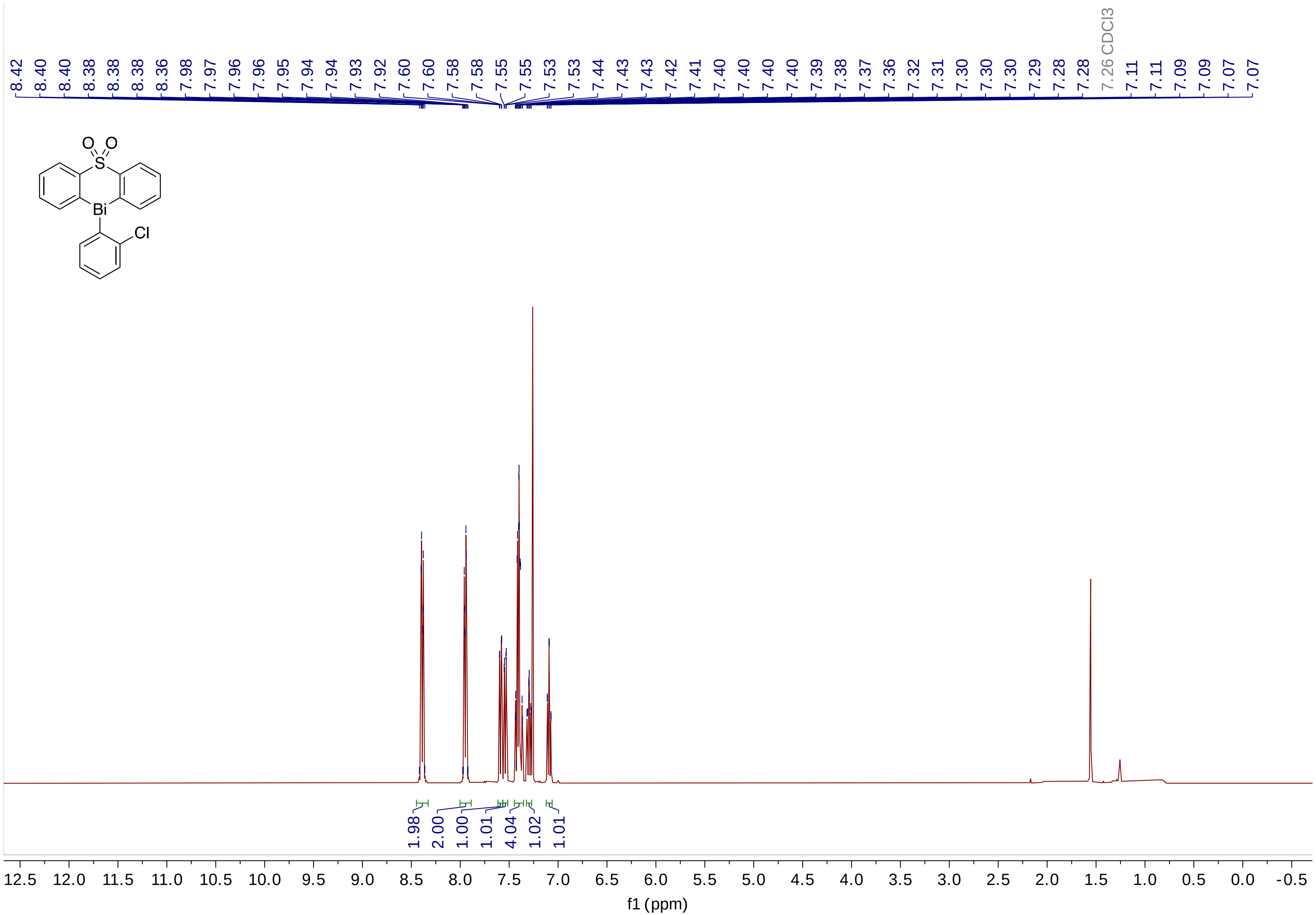

2b - $^{13}C\{^{1}H\}$ NMR (101 MHz, $CDCl_3$):

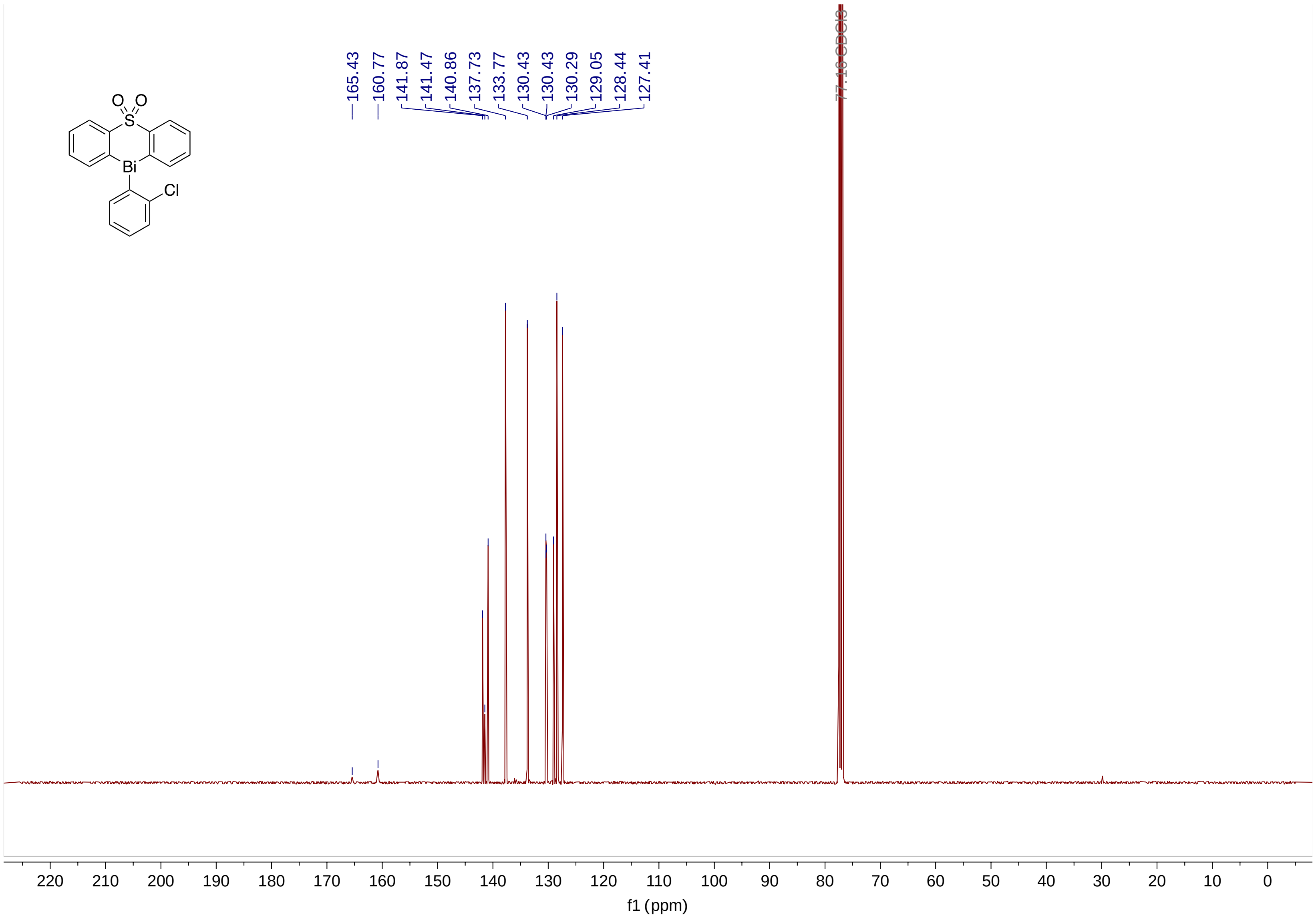

2c - ^{1}H NMR (400 MHz, $CDCl_3$):

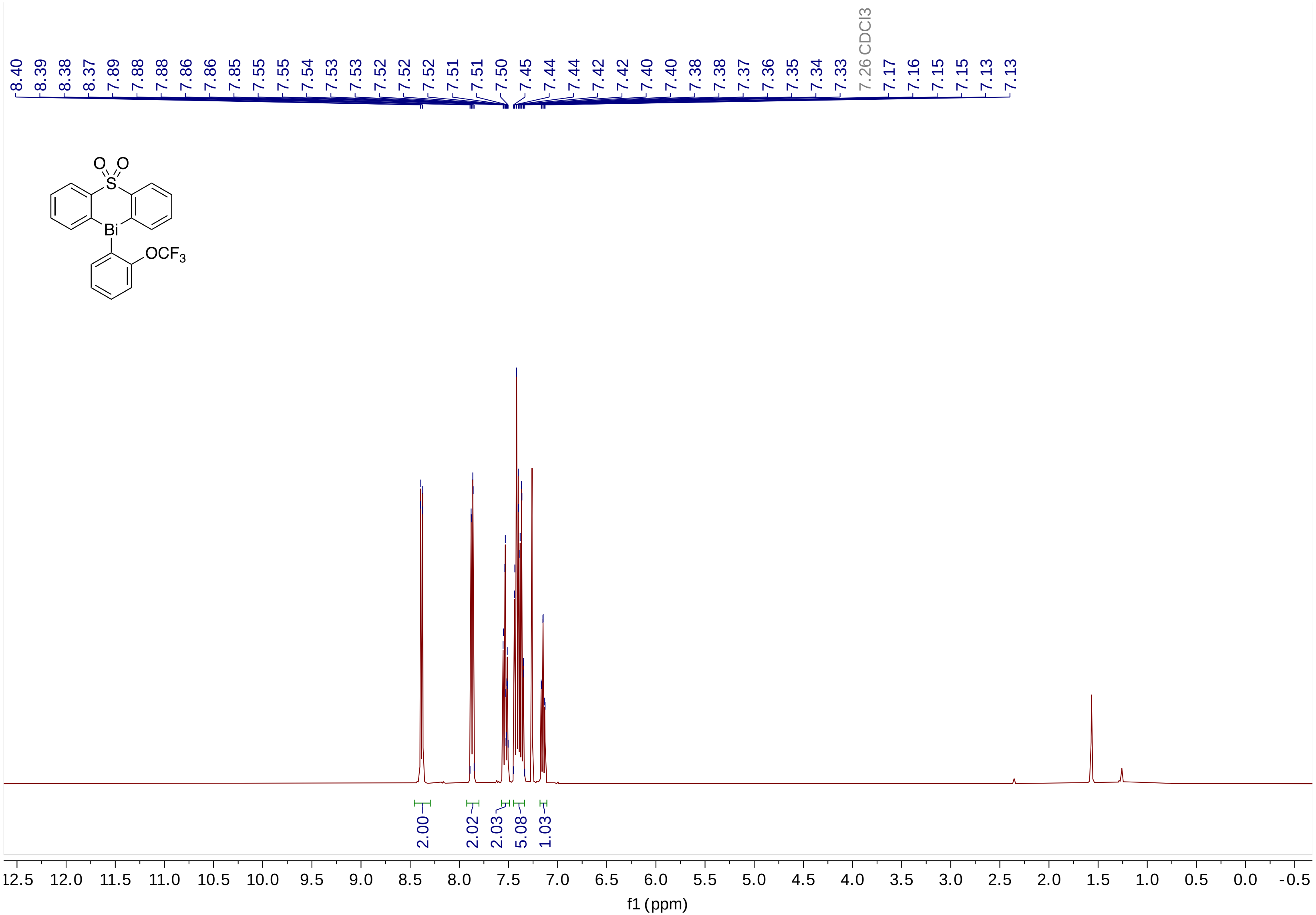

2c - $^{13}C\{^1H\}$ NMR (101 MHz, $CDCl_3$):

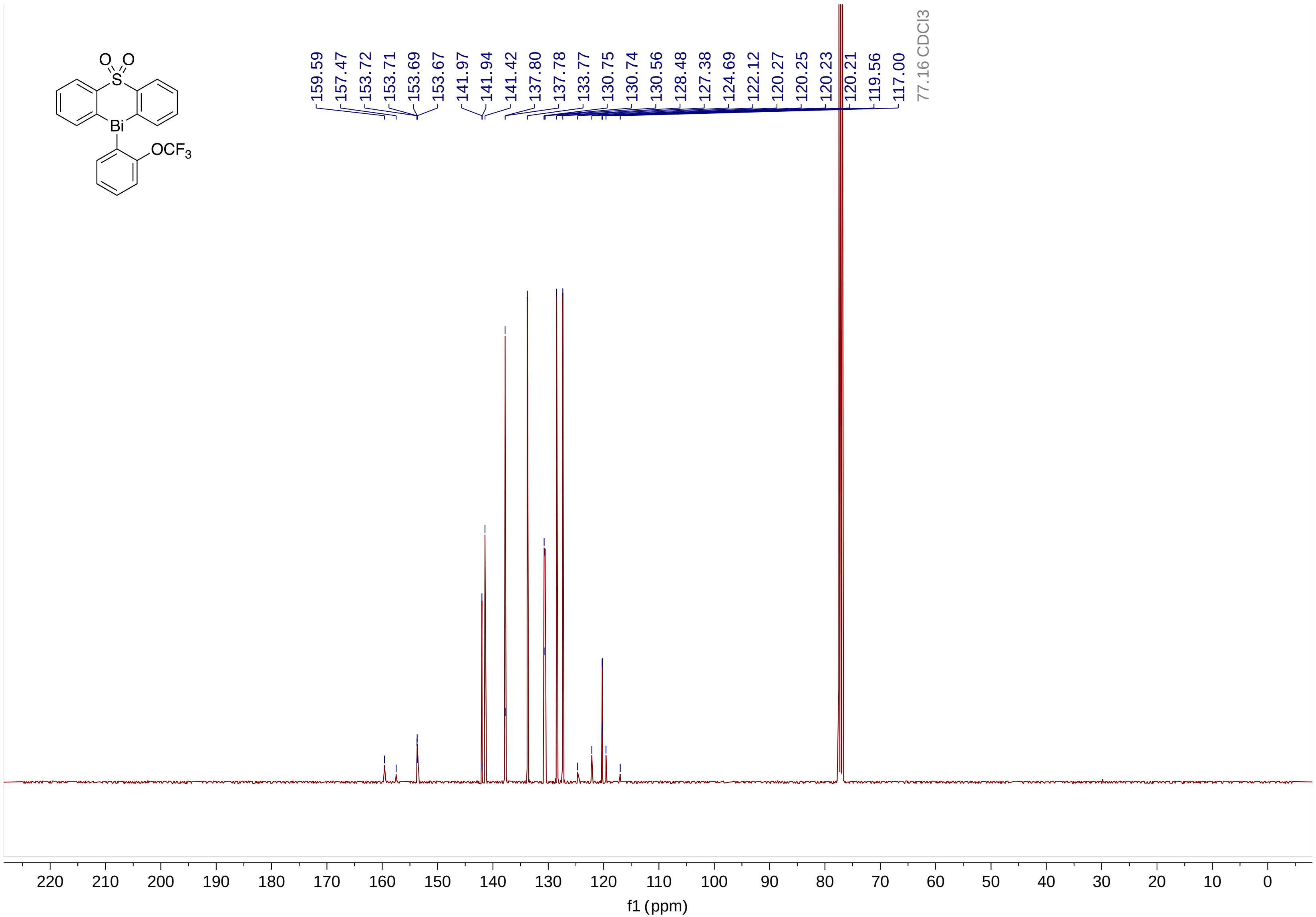

2c - ^{19}F NMR (377 MHz, CDCl$_3$):

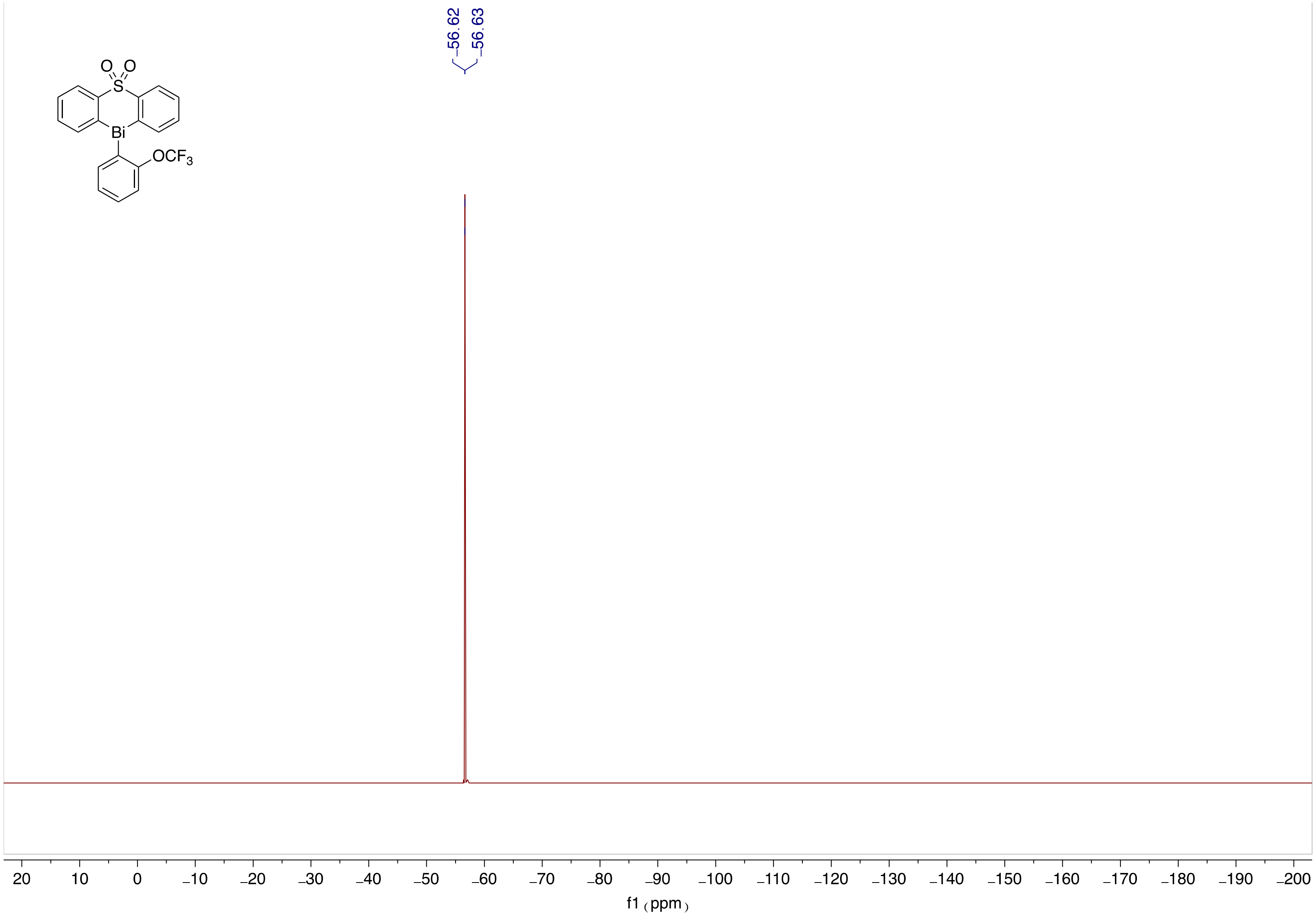

2d - ^{1}H NMR (400 MHz, $CDCl_3$):

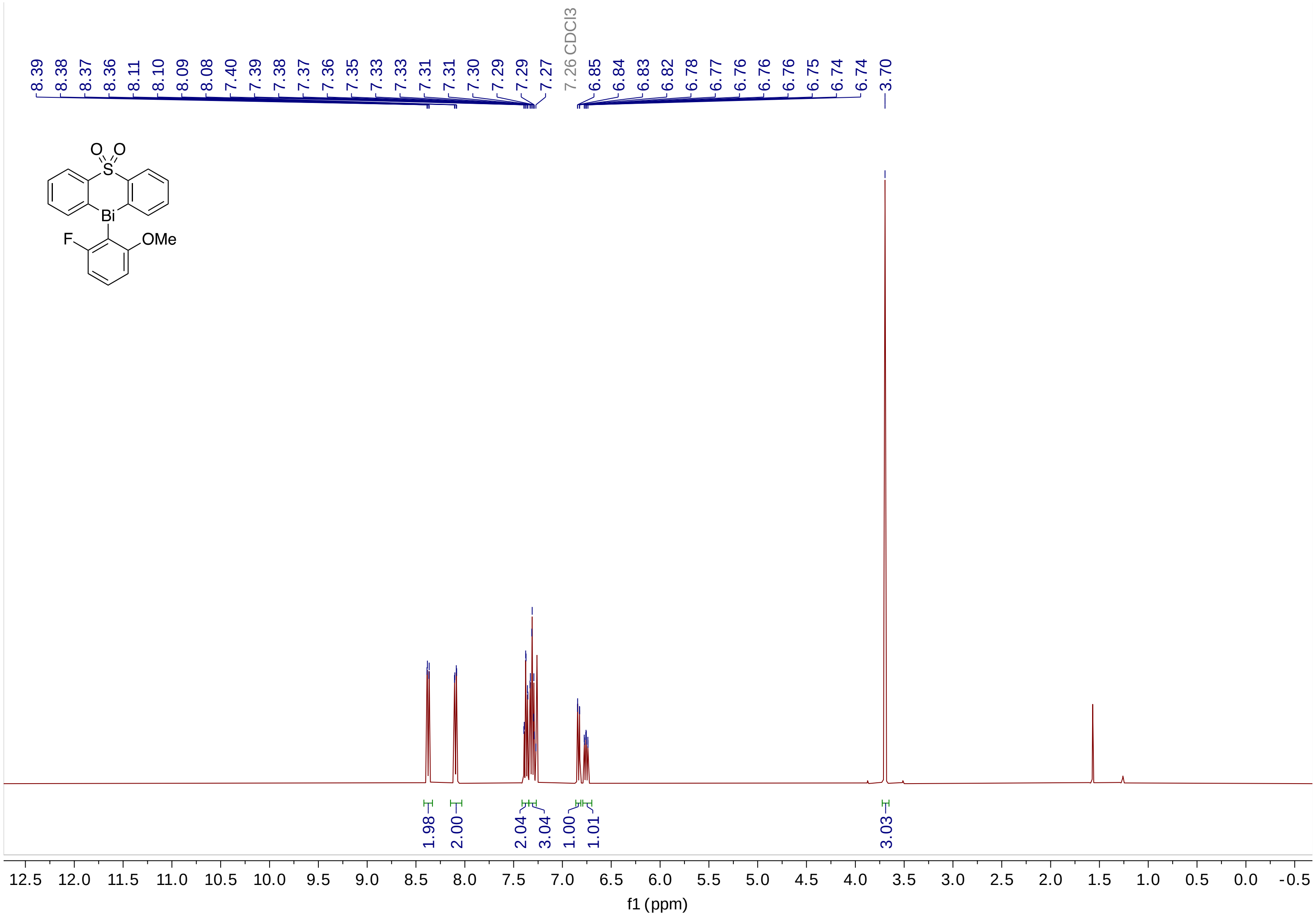

2d - $^{13}C\{^{1}H\}$ NMR (126 MHz, $CDCl_3$):

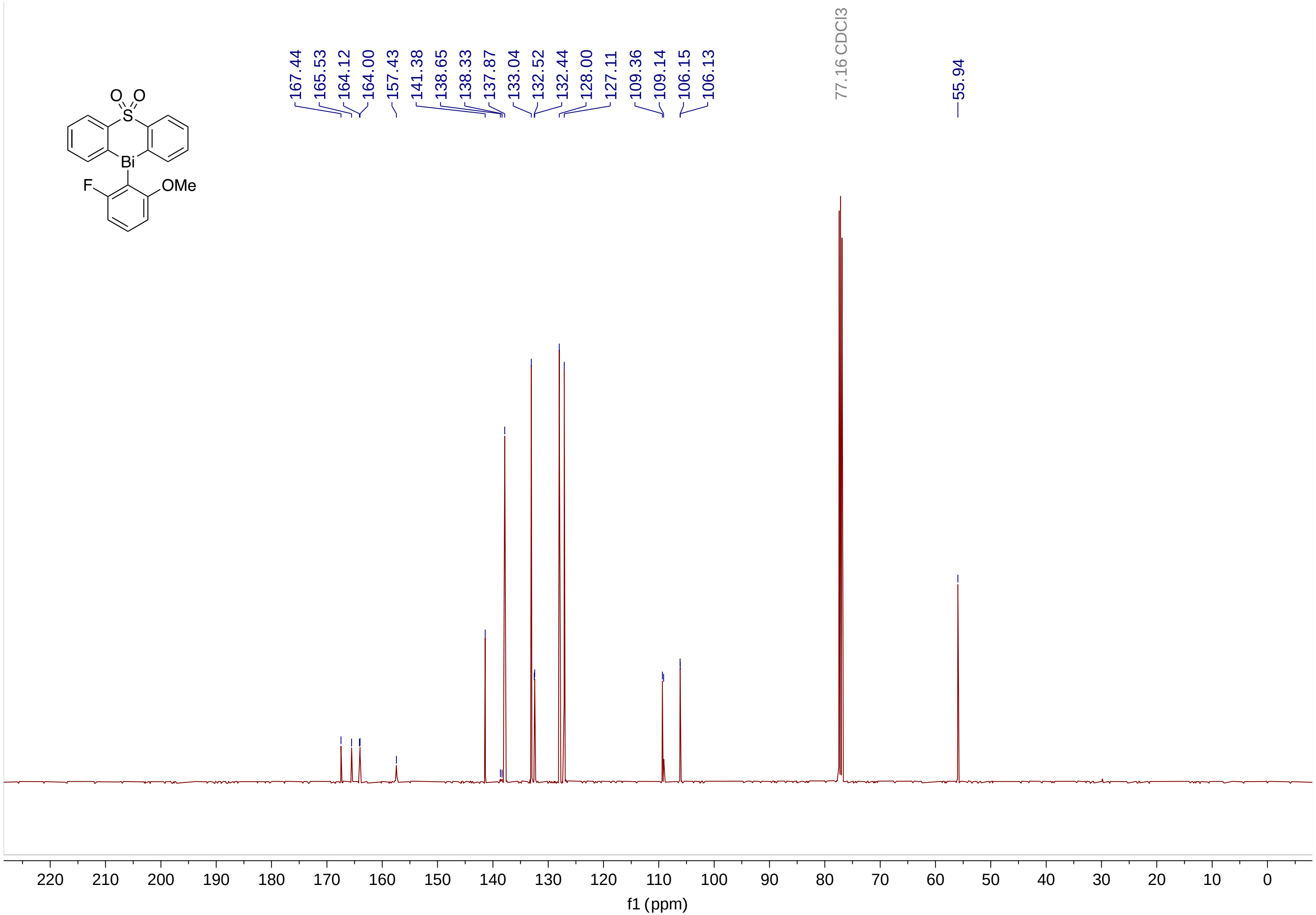

2d - ^{19}F NMR (376 MHz, $CDCl_3$):

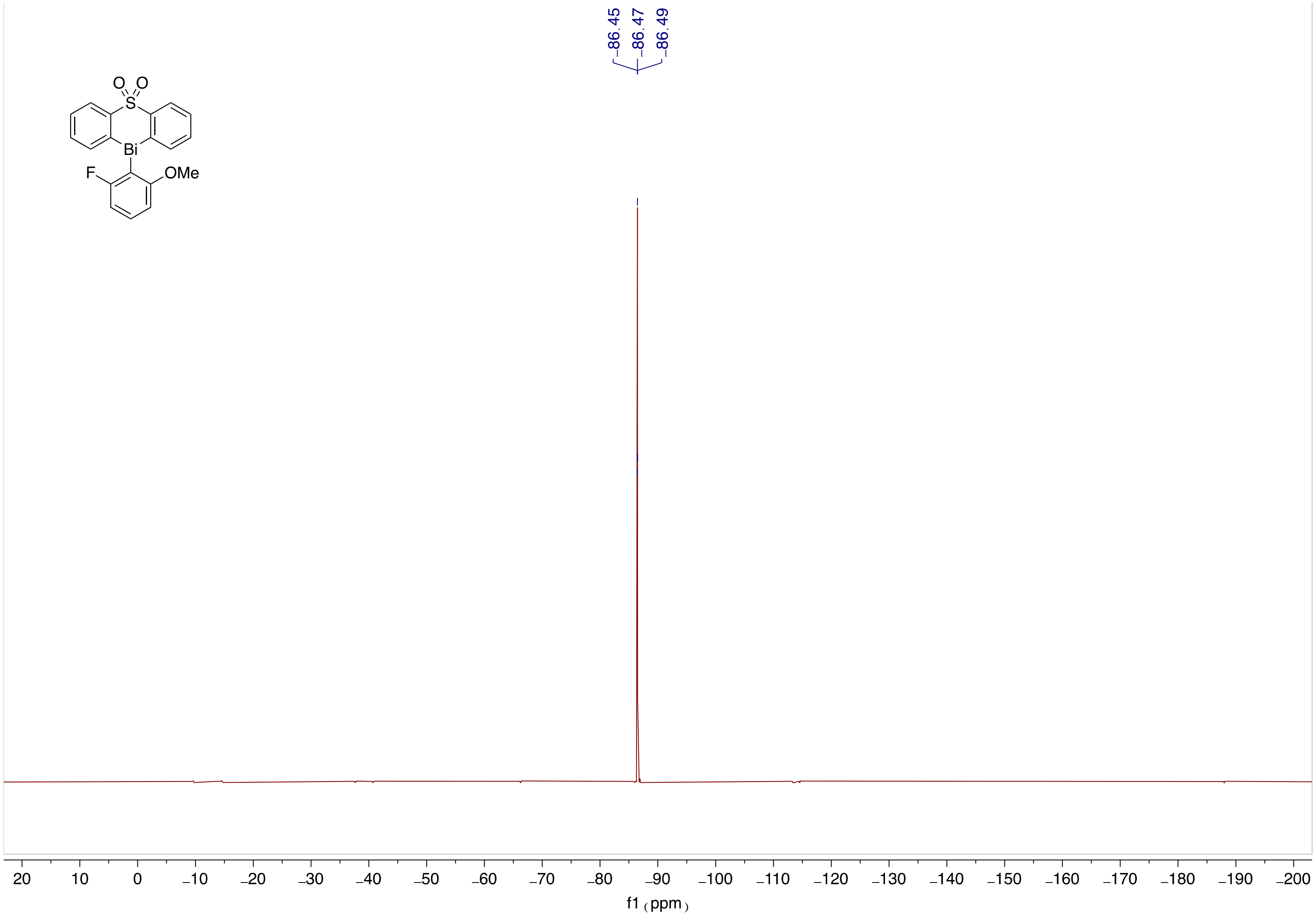

2e - ^{1}H NMR (500 MHz, CDCl$_3$):

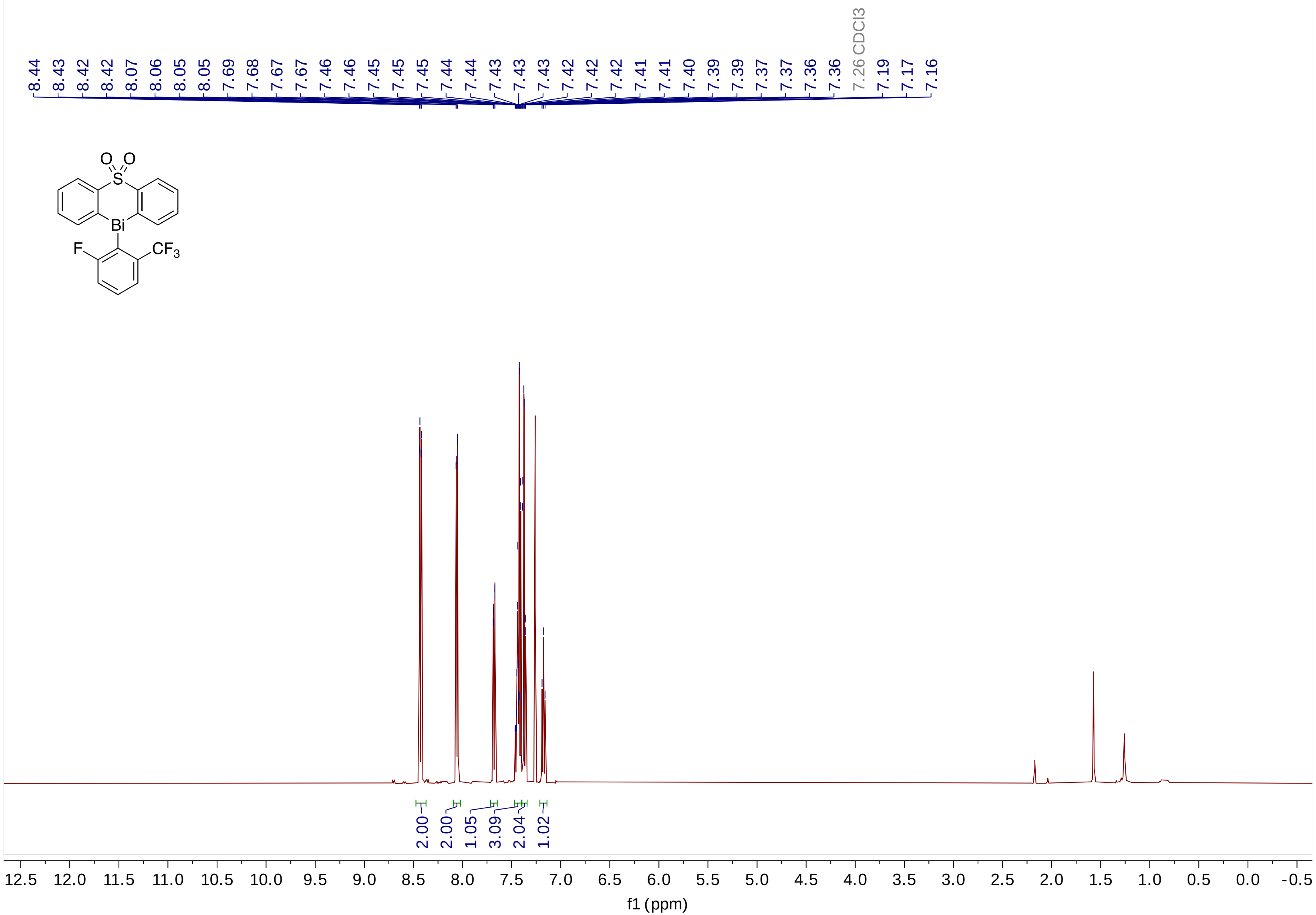

2e - $^{13}C\{^{1}H\}$ NMR (101 MHz, $CDCl_3$):

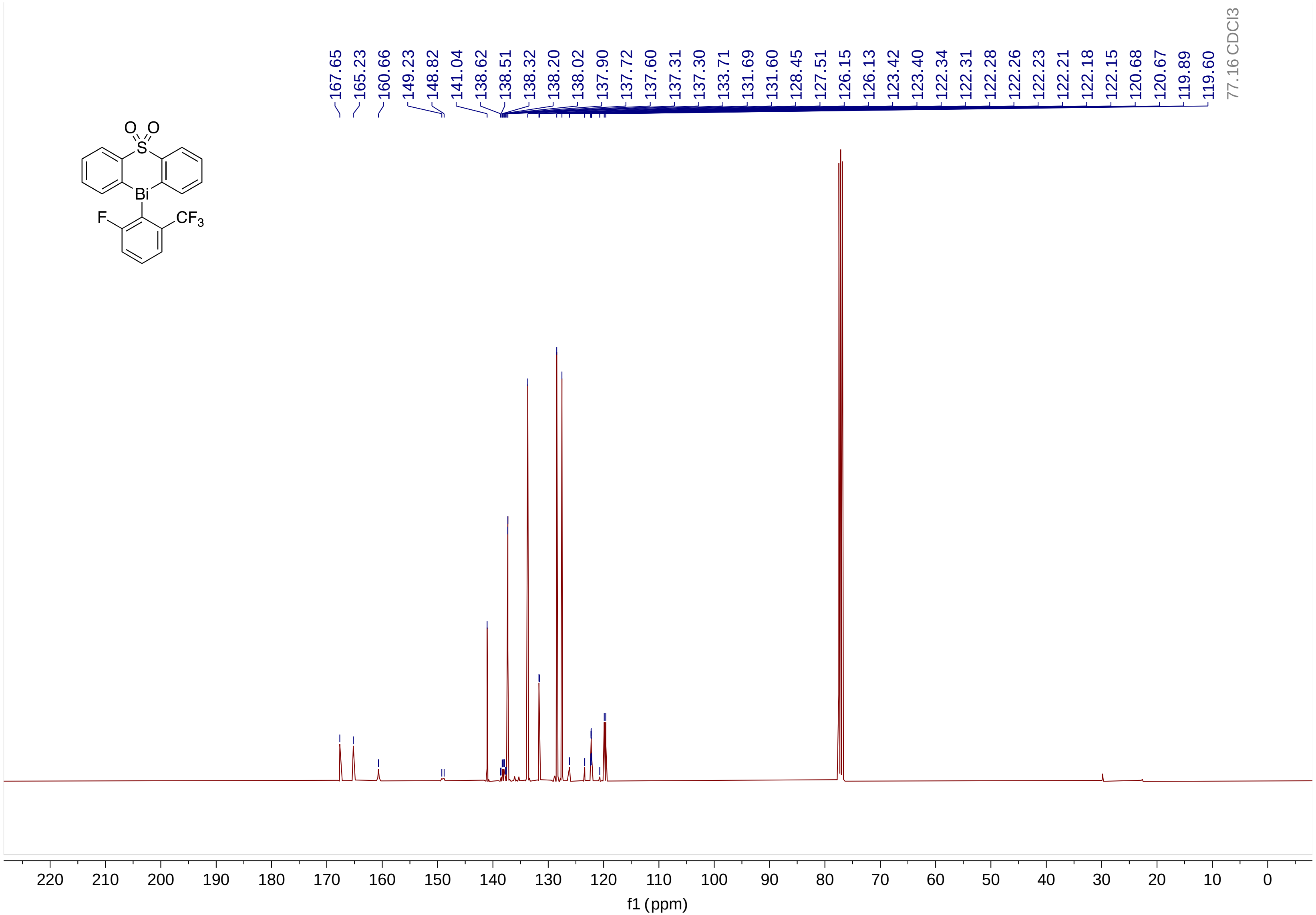

2e - ^{19}F NMR (377 MHz, CDCl$_3$):

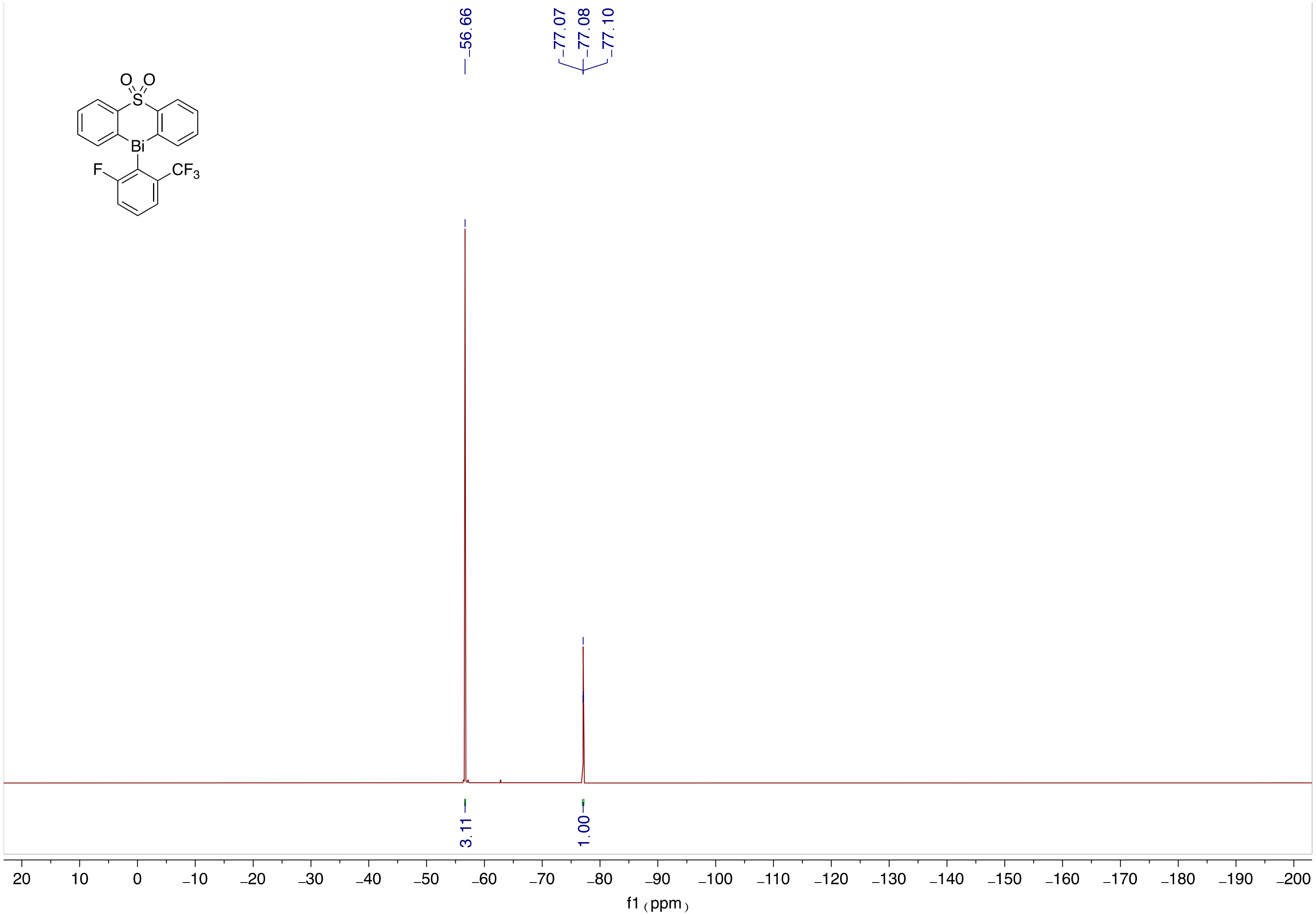

S2 - ^{1}H NMR (400 MHz, DMSO-d_6):

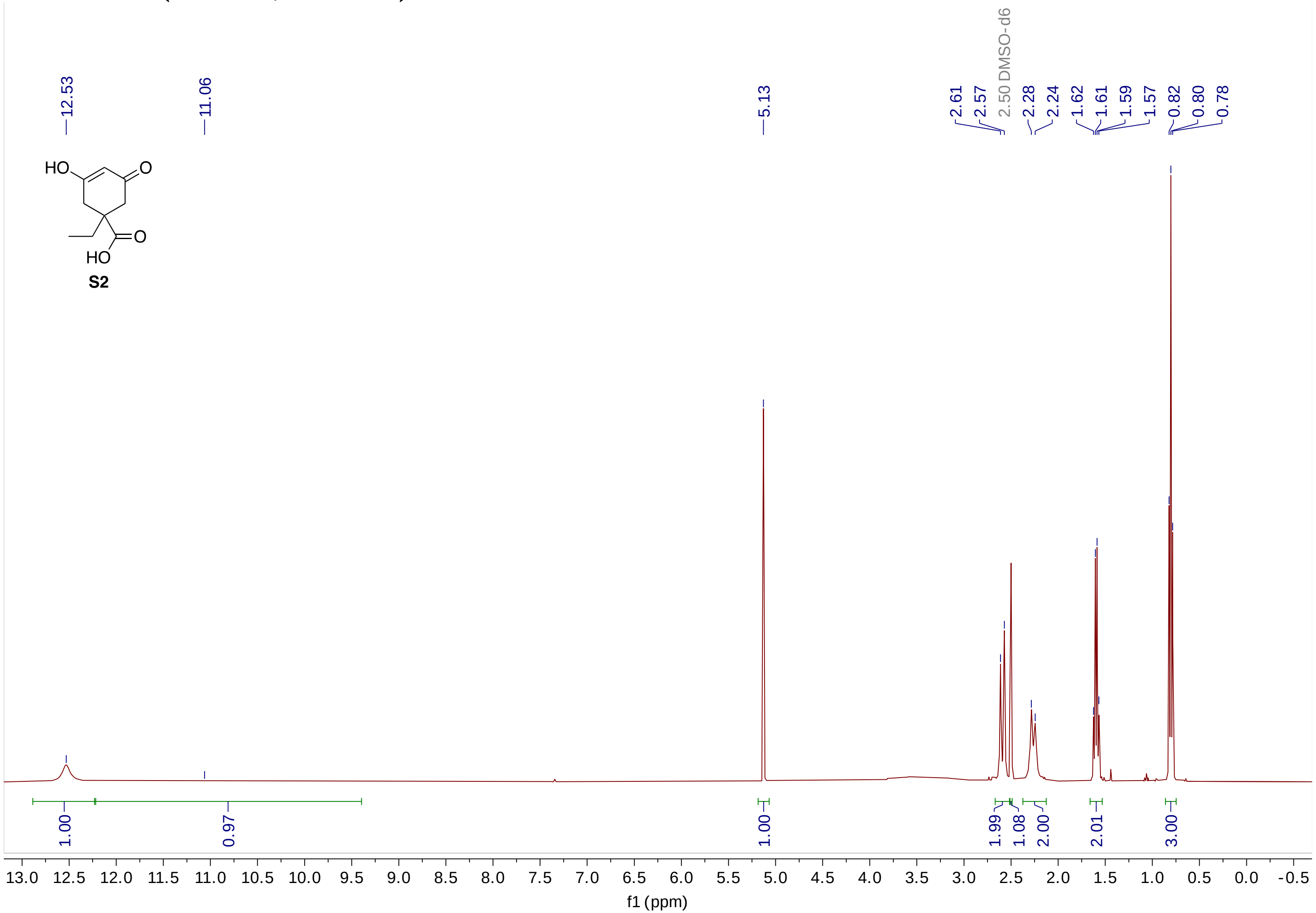

S2 - $^{13}C\{^1H\}$ NMR (126 MHz, DMSO-d_6):

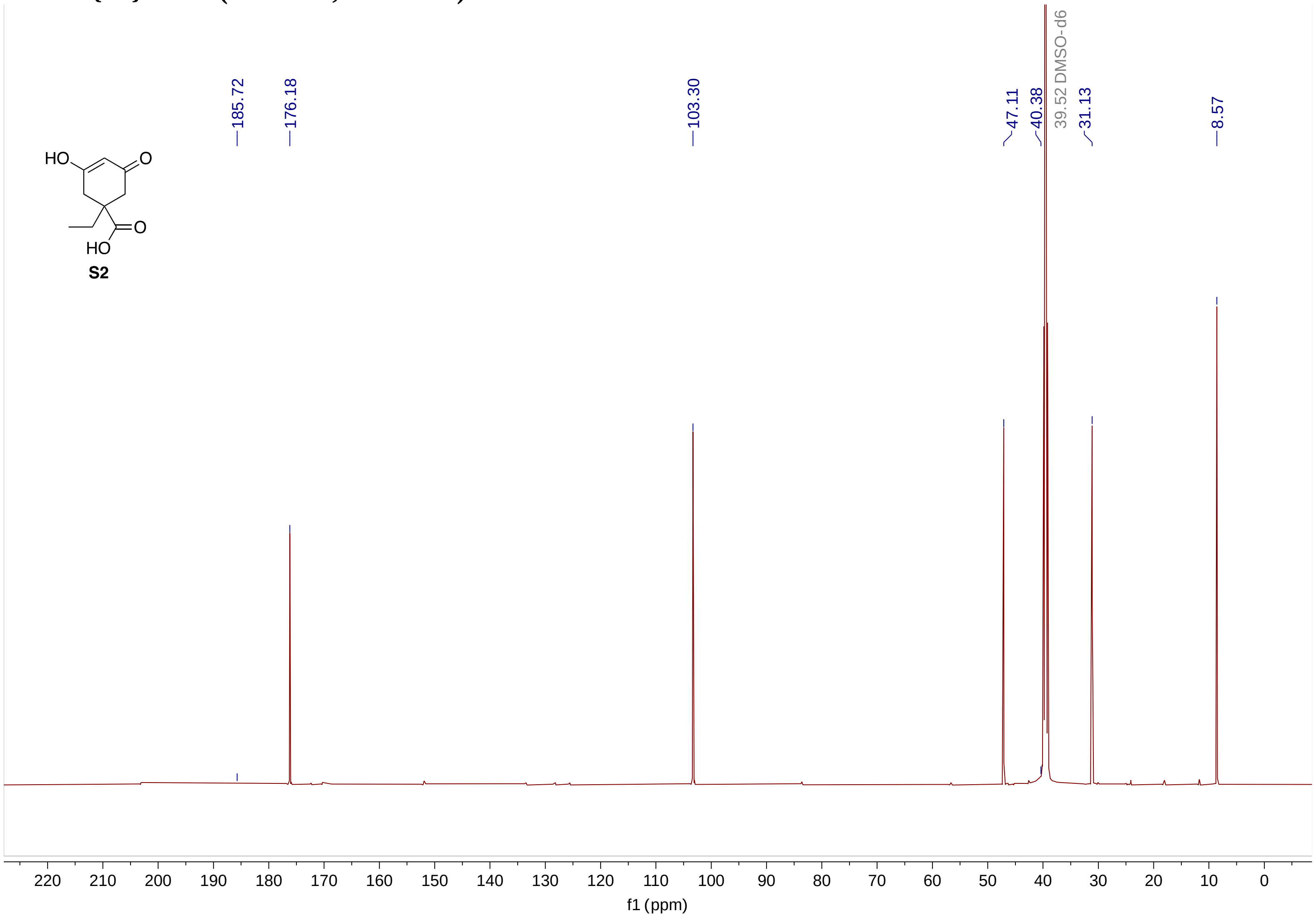

S3 - ^{1}H NMR (400 MHz, DMSO-d_6):

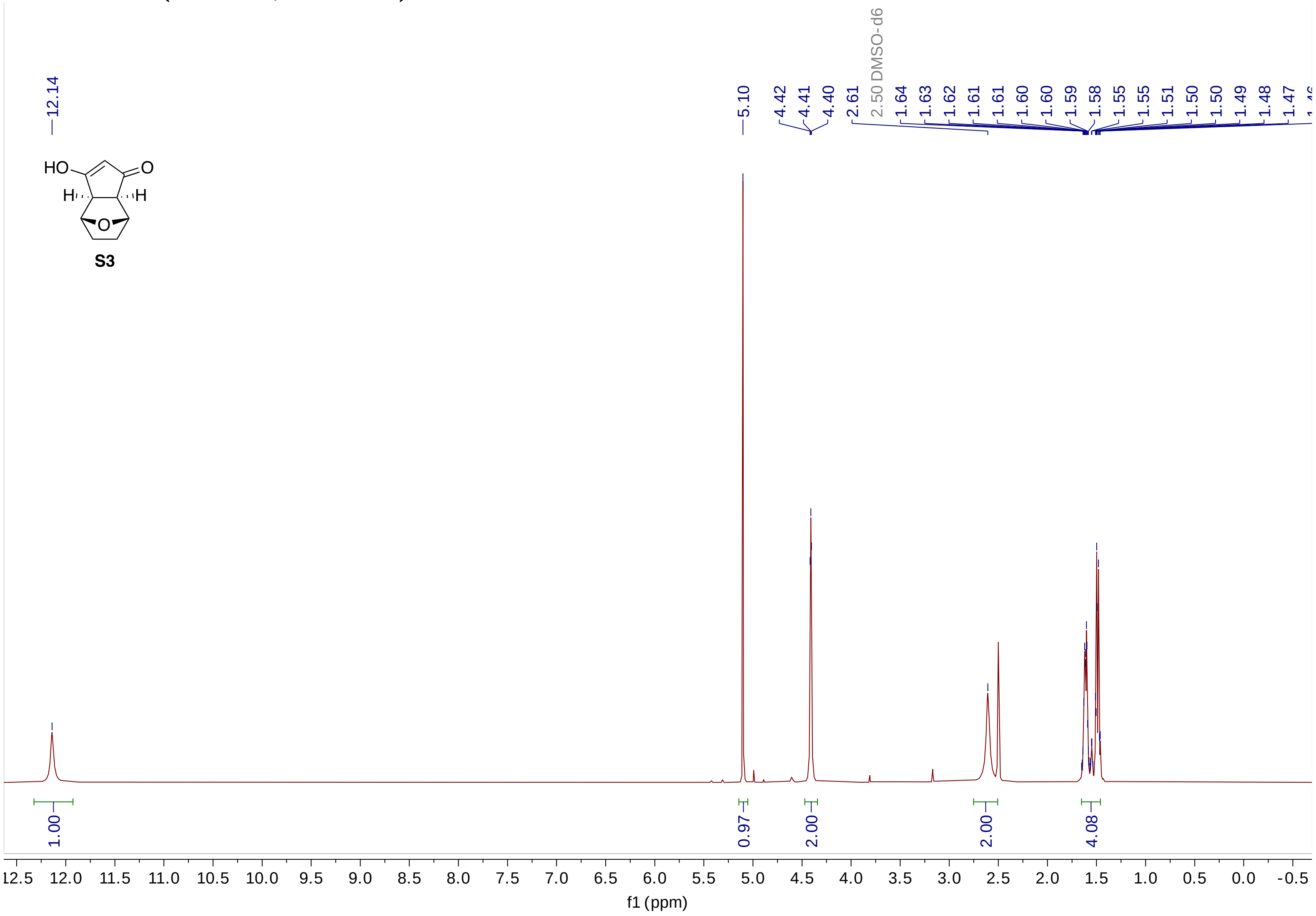

S3 - $^{13}C\{^1H\}$ NMR (126 MHz, DMSO-d_6):

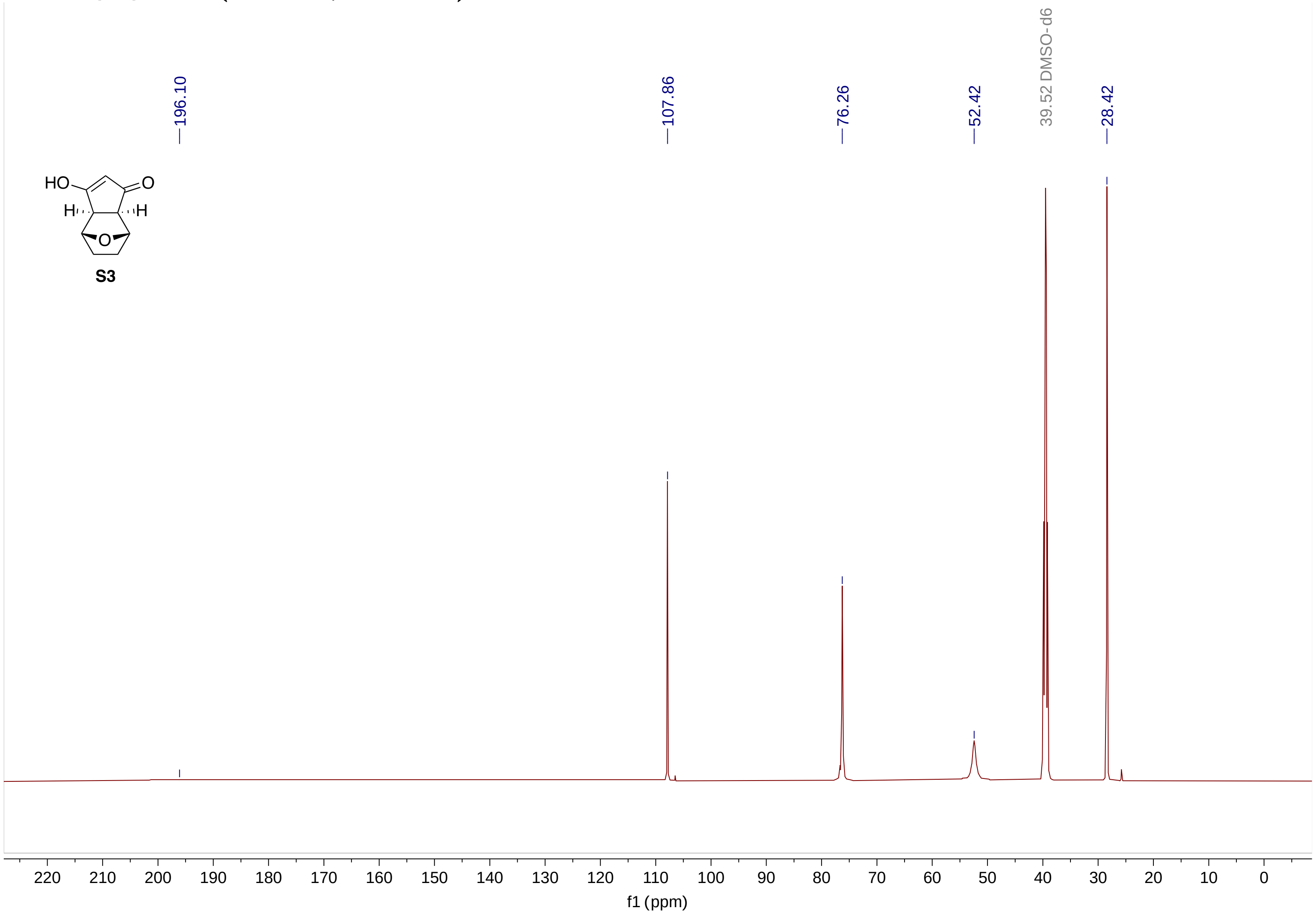

S4 - ^{1}H NMR (500 MHz, DMSO-d_6):

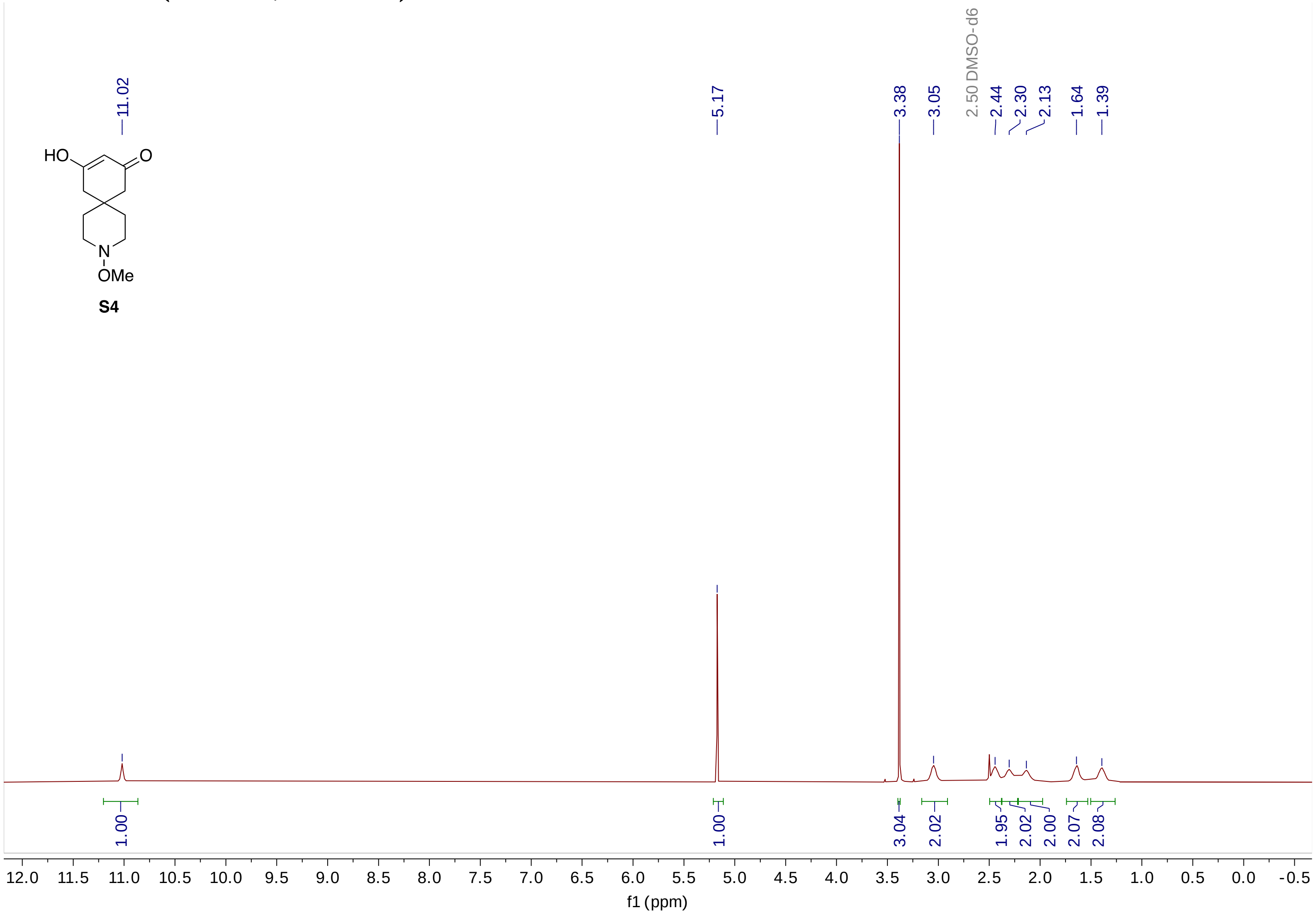

S4 - $^{13}C\{^{1}H\}$ NMR (126 MHz, DMSO-d_6):

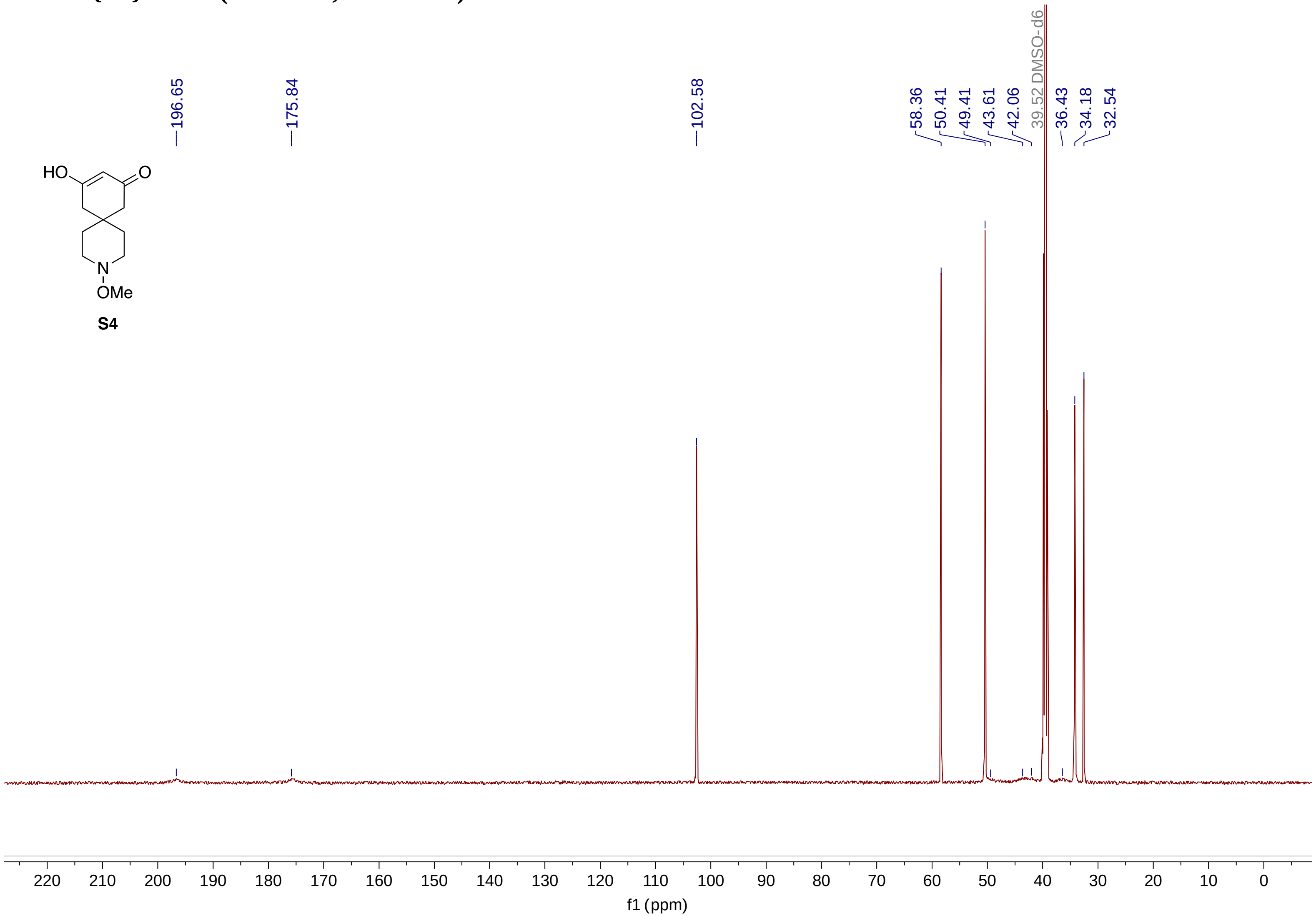

S5 - ¹H NMR (400 MHz, DMSO-*d*₆):

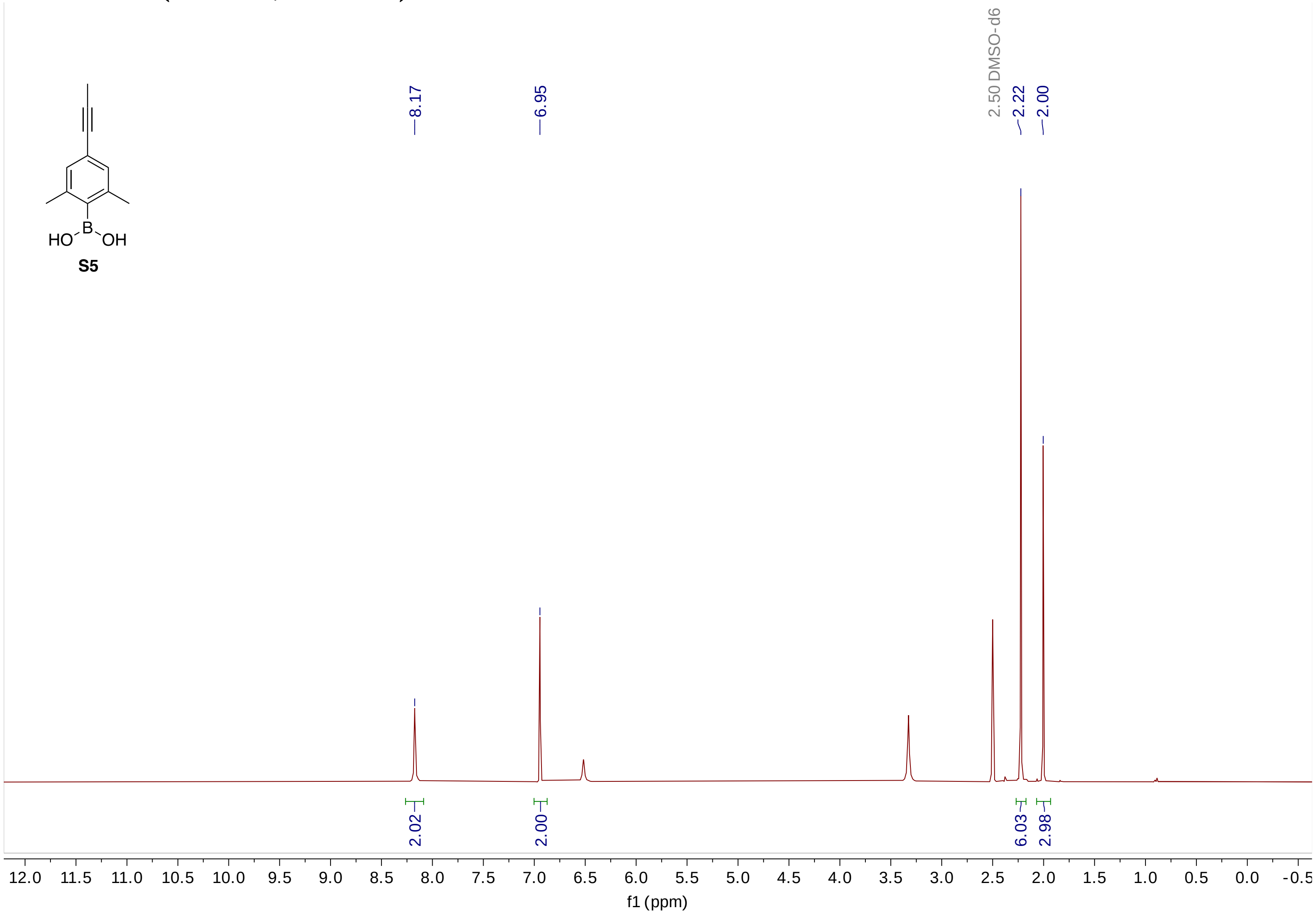

S5 - $^{13}C\{^{1}H\}$ NMR (126 MHz, DMSO-d_6):

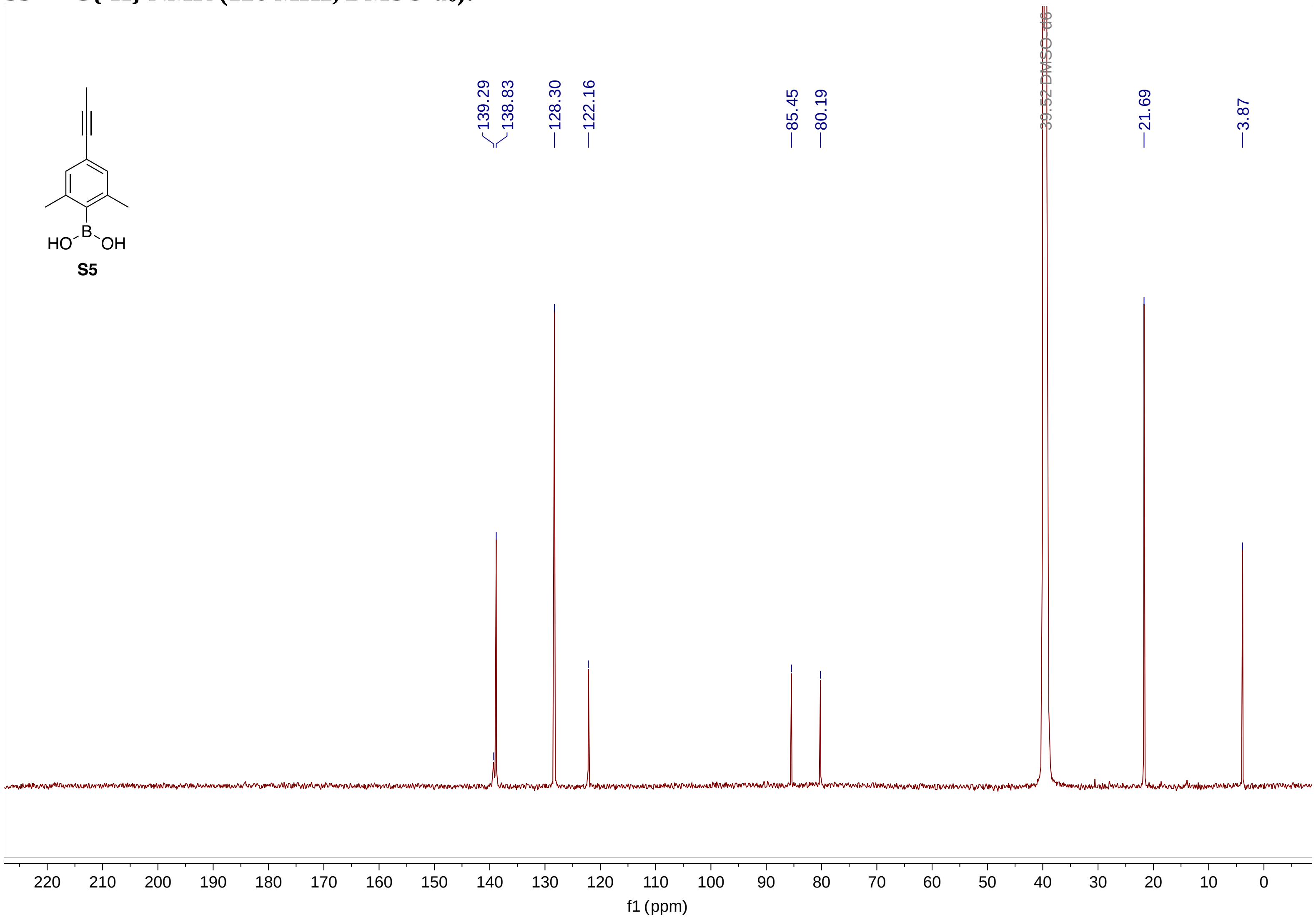

3b - ^{1}H NMR (500 MHz, CD3CN):

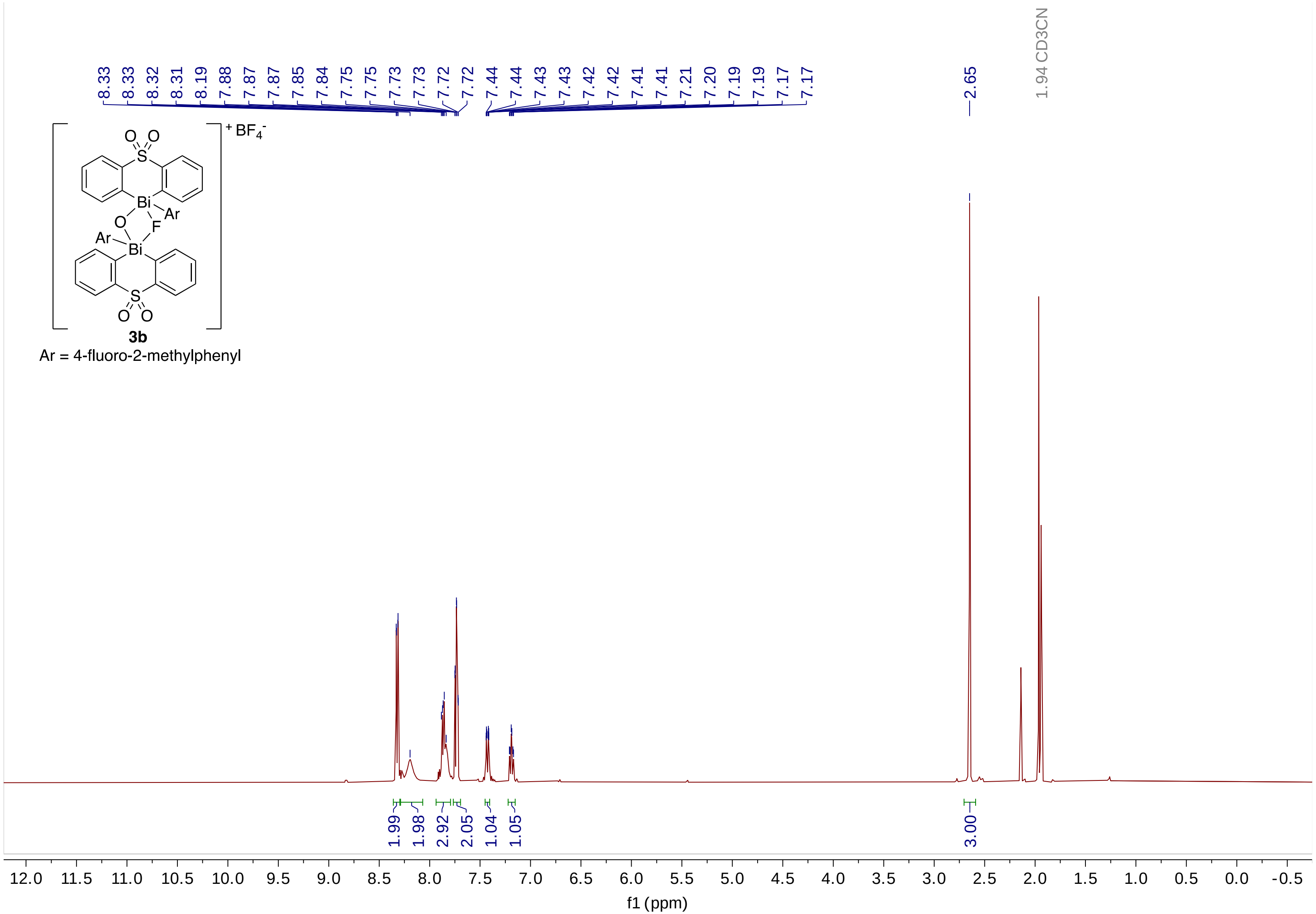

3b - $^{13}C\{^1H\}$ NMR (126 MHz, CD_3CN):

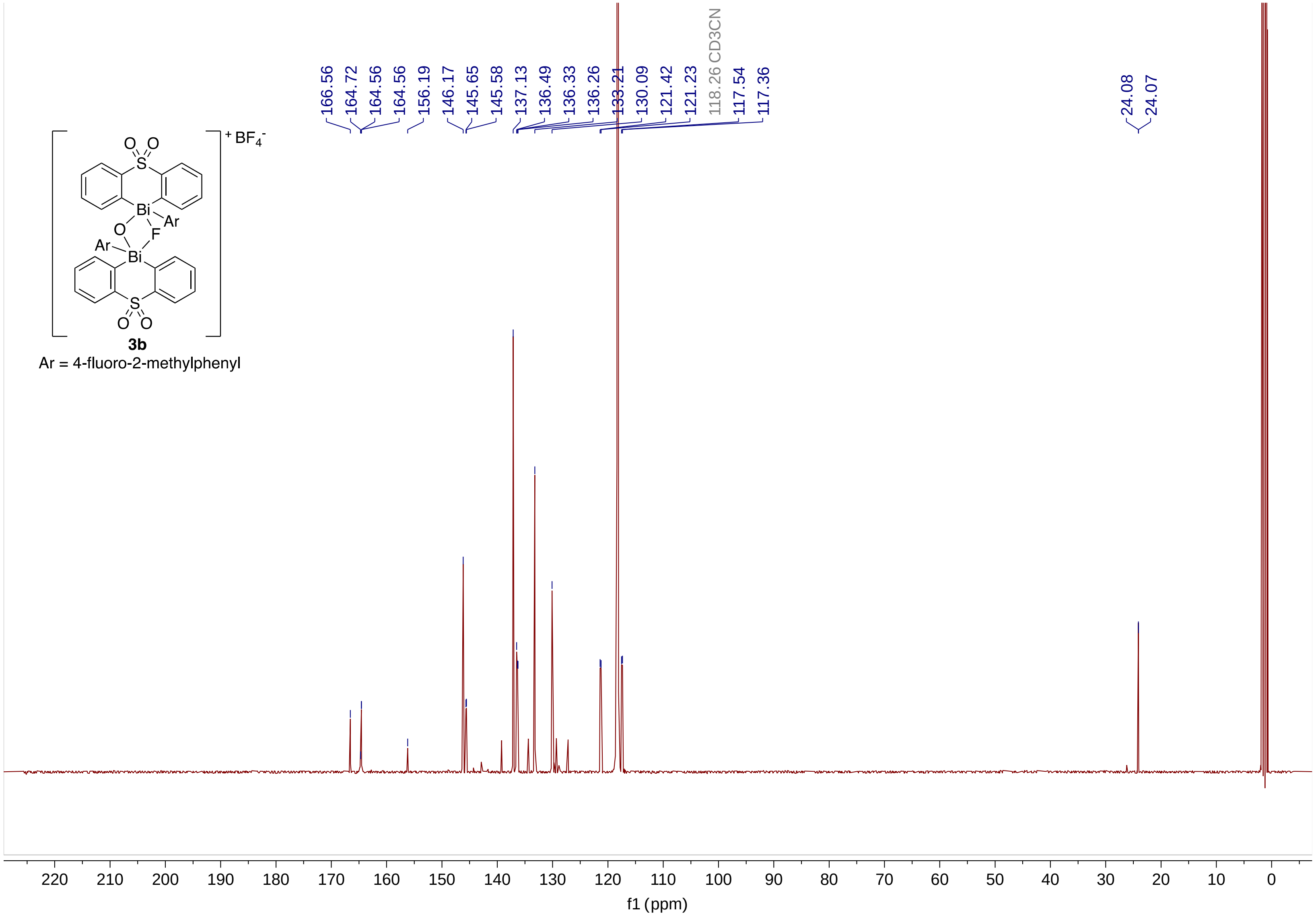

3b - ^{19}F NMR (376 MHz, CD_3CN):

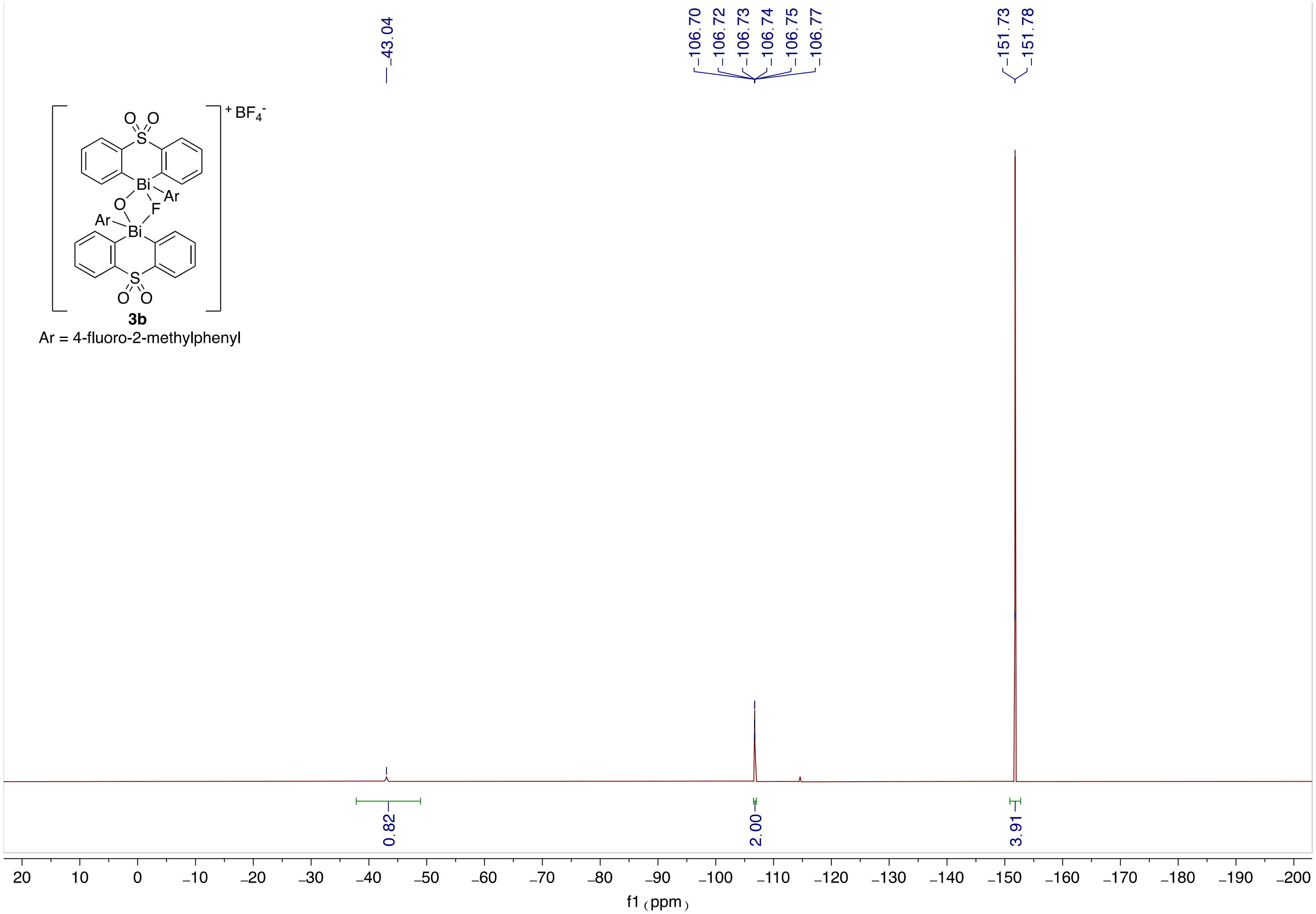

3c - ^{1}H NMR (500 MHz, CD_3CN):

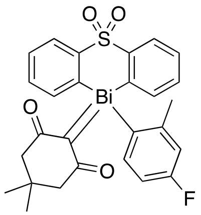

3c

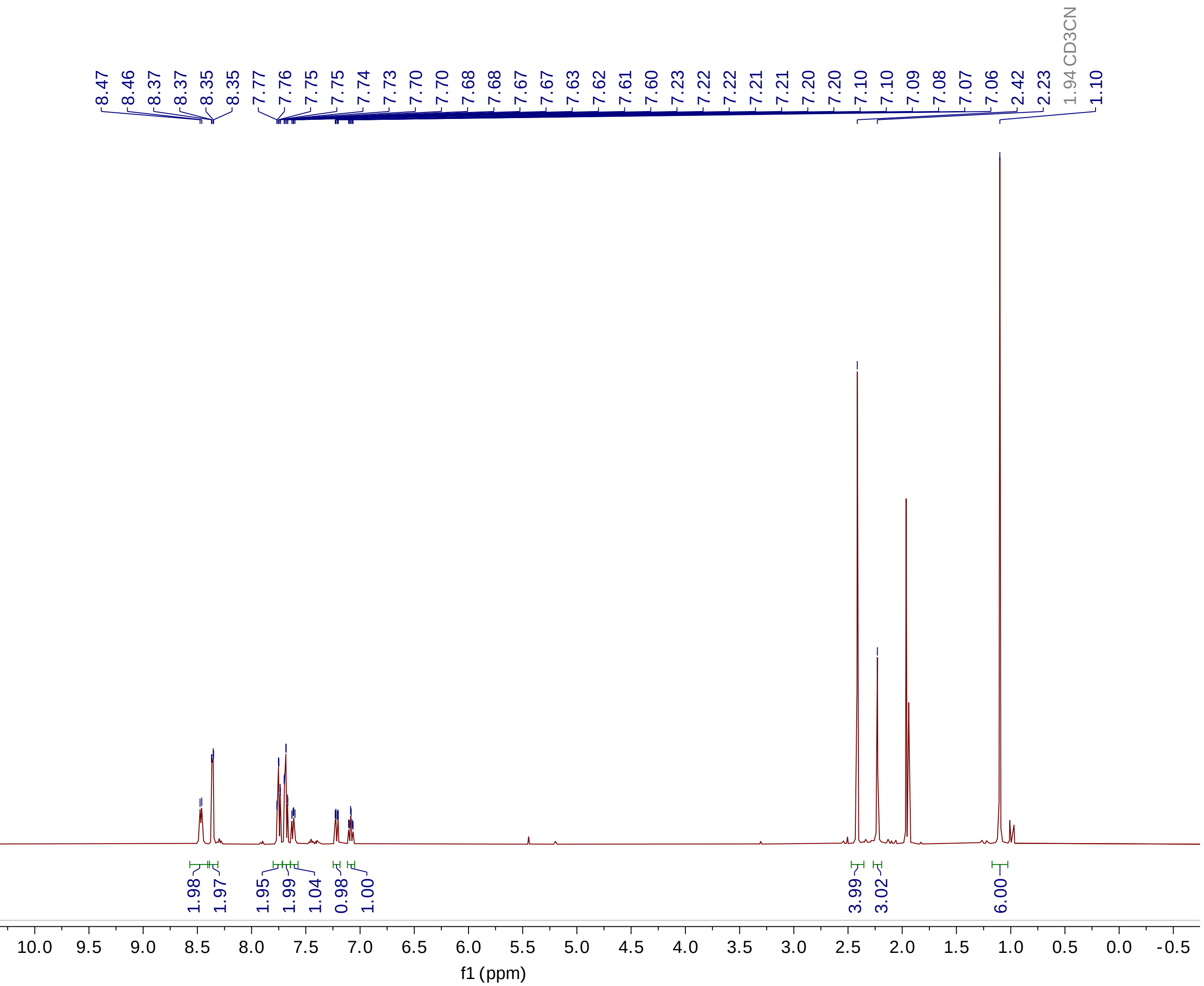

3c - ^{1}H NMR (500 MHz, CDCl$_3$):

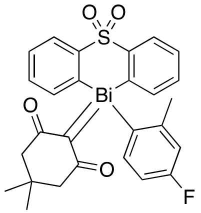

3c

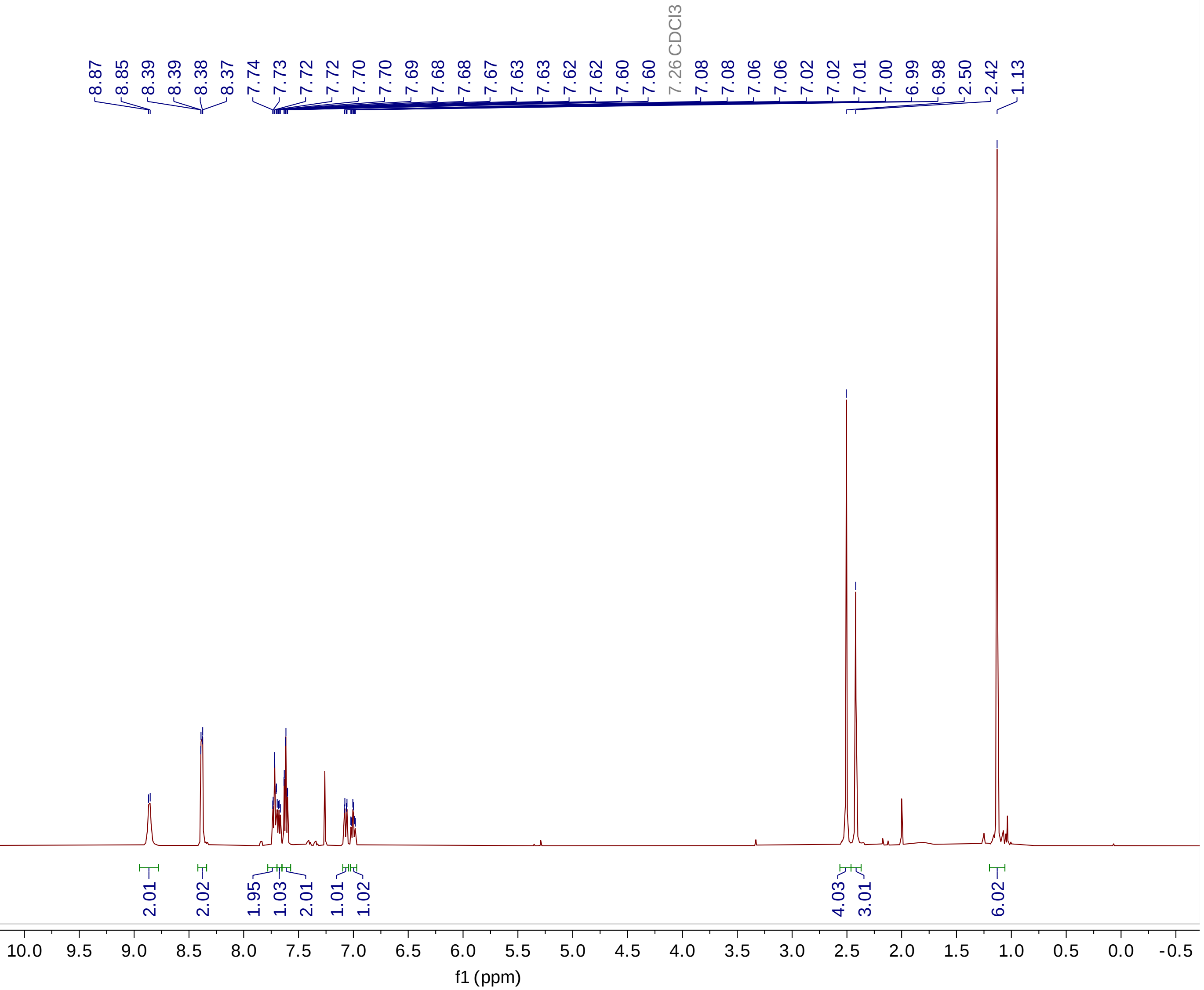

3c - $^{13}C\{^1H\}$ NMR (126 MHz, CD_3CN):

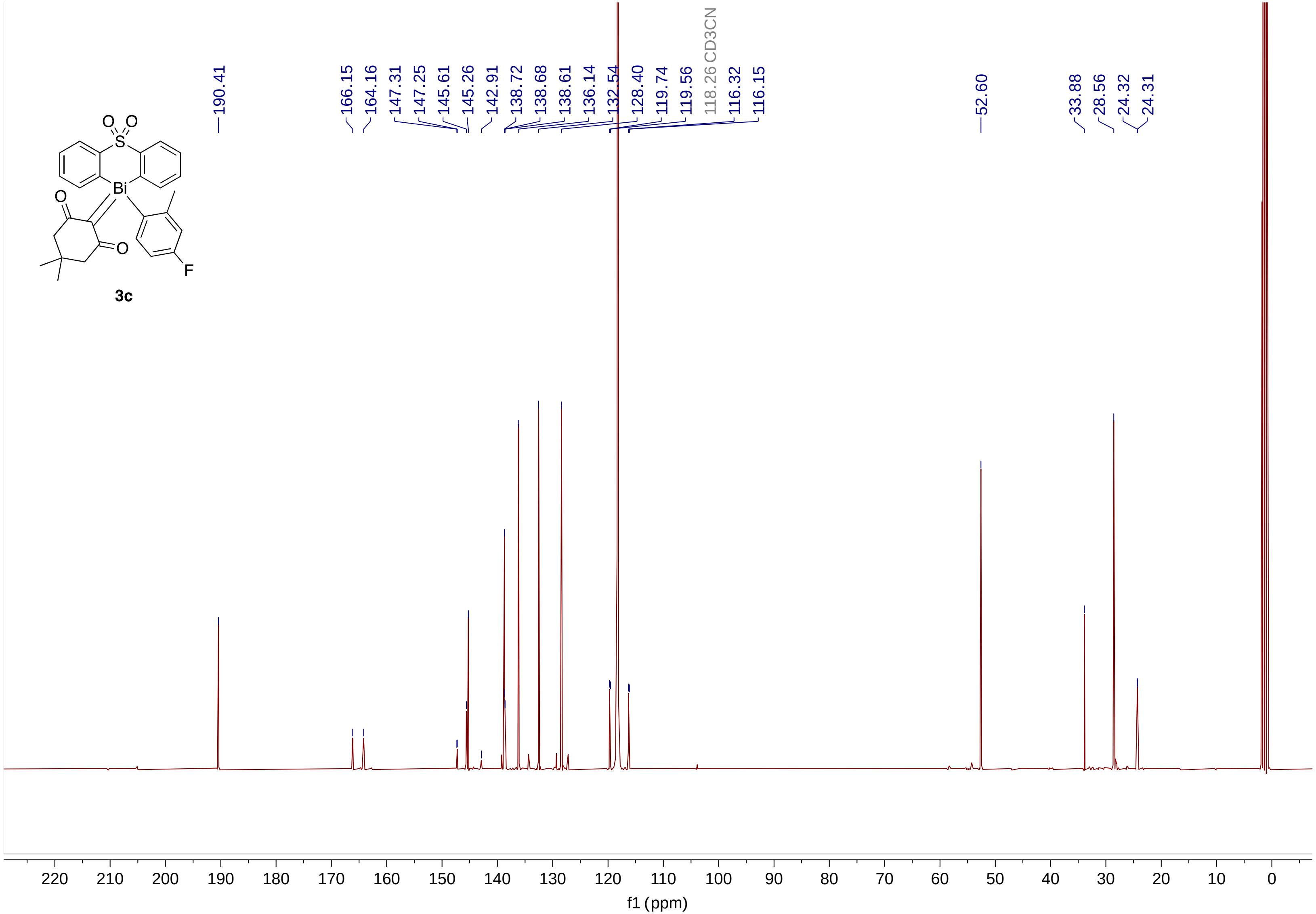

3c - $^{13}C\{^{1}H\}$ NMR (126 MHz, $CDCl_3$):

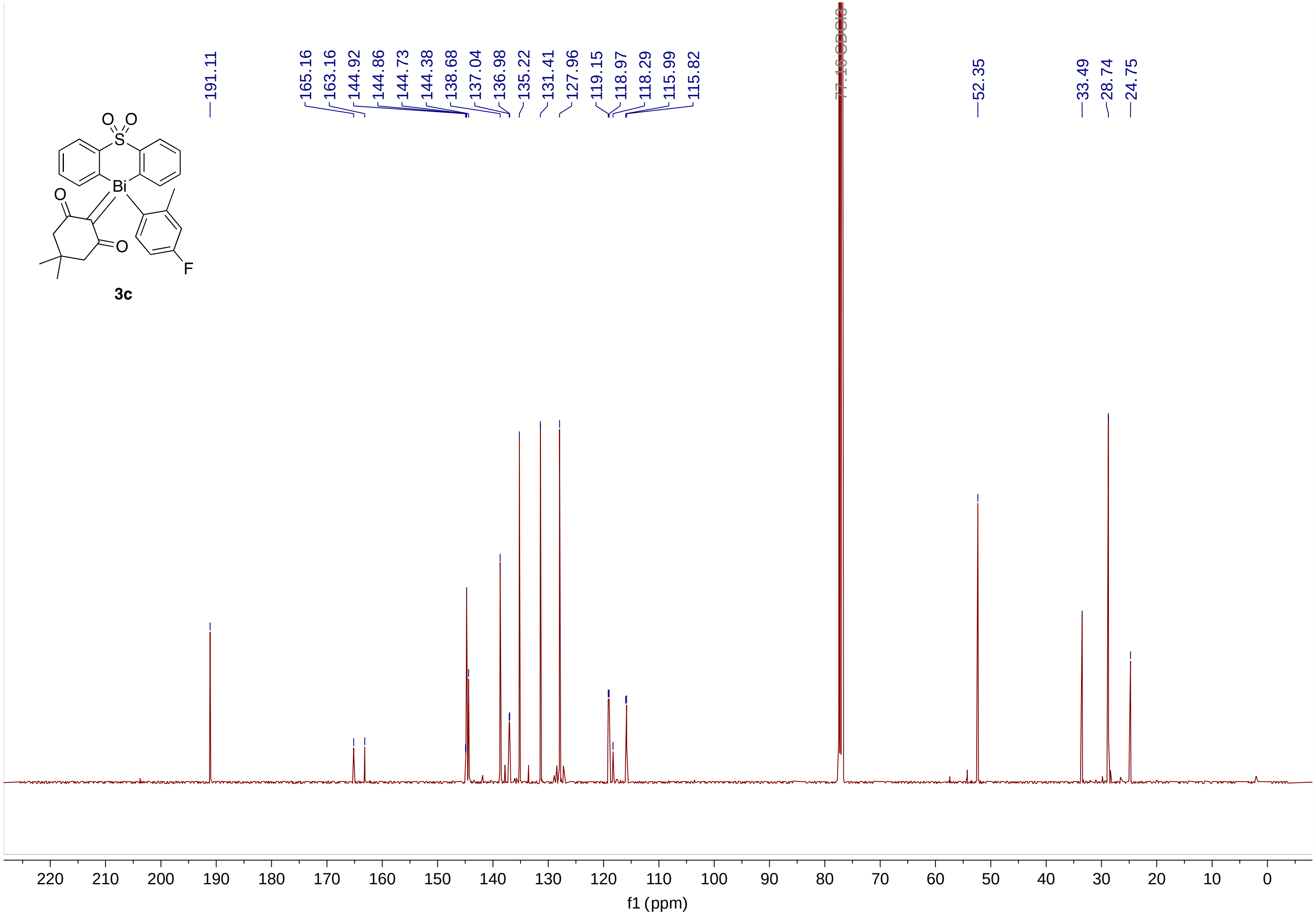

3c – COSY (500 MHz, CD_3CN):

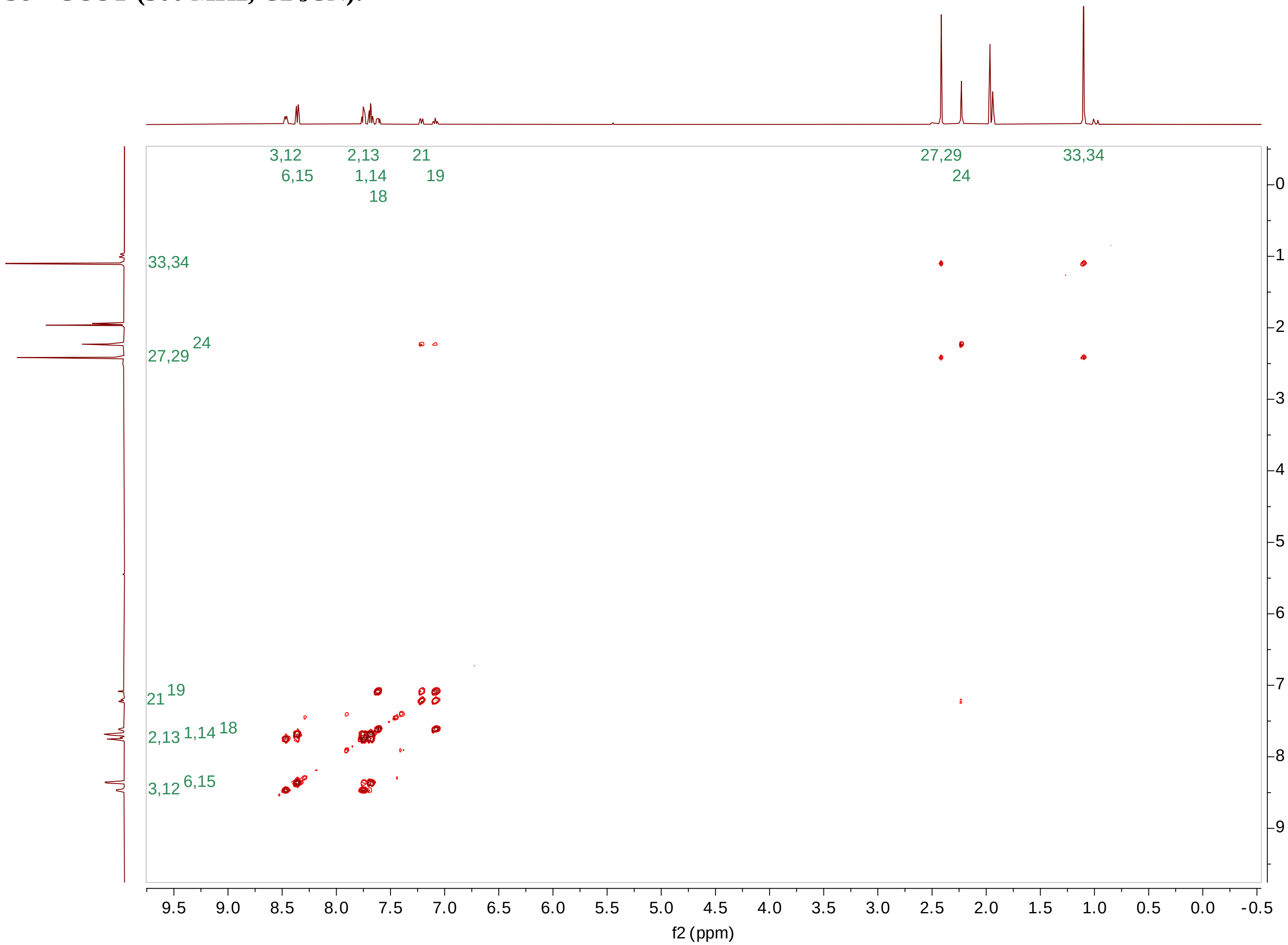

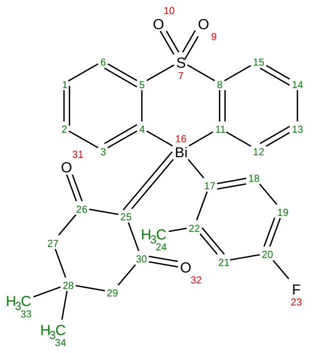

3c – HSQC (500 MHz, CD_3CN):

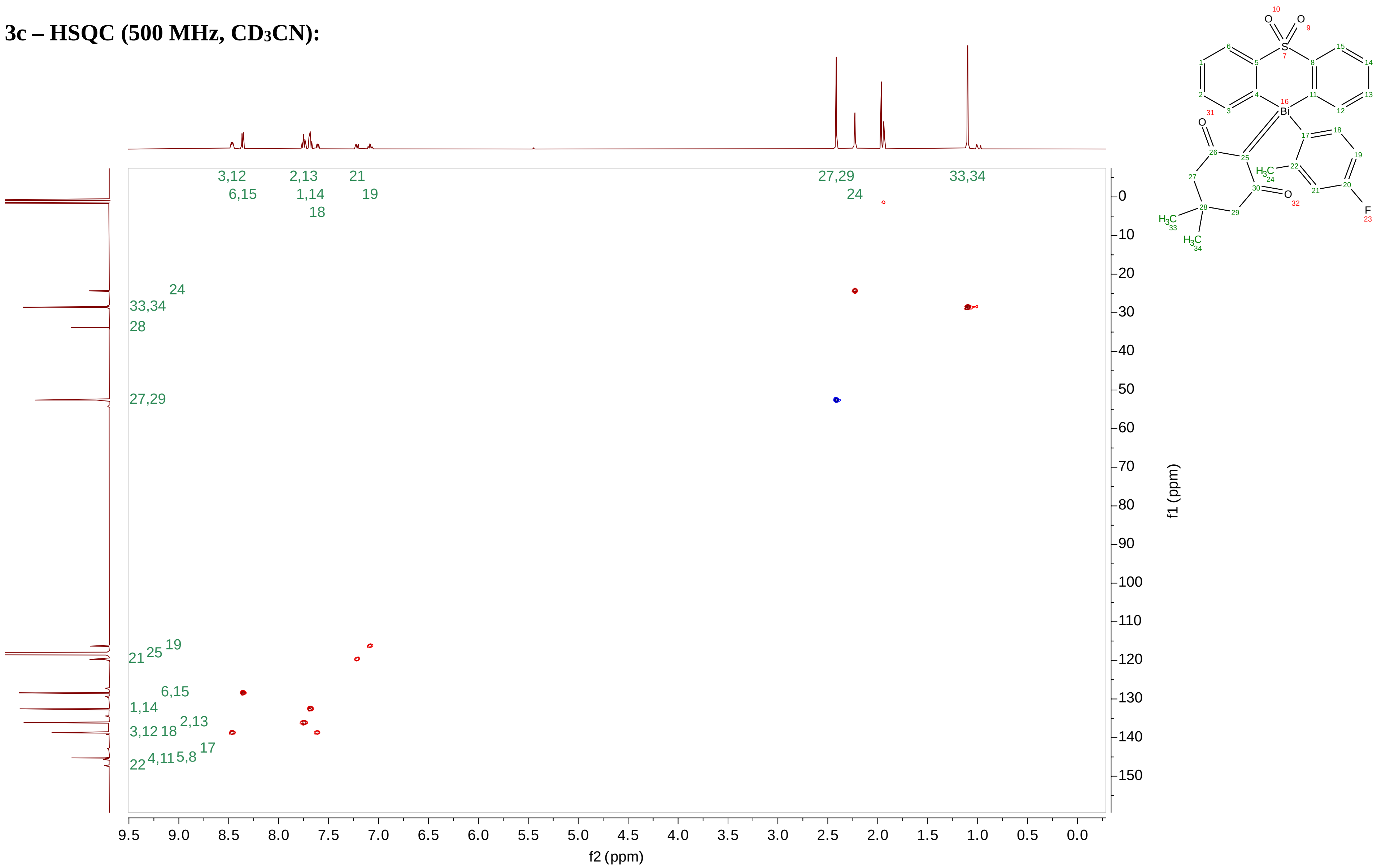

3c – HMBC (500 MHz, CD_3CN):

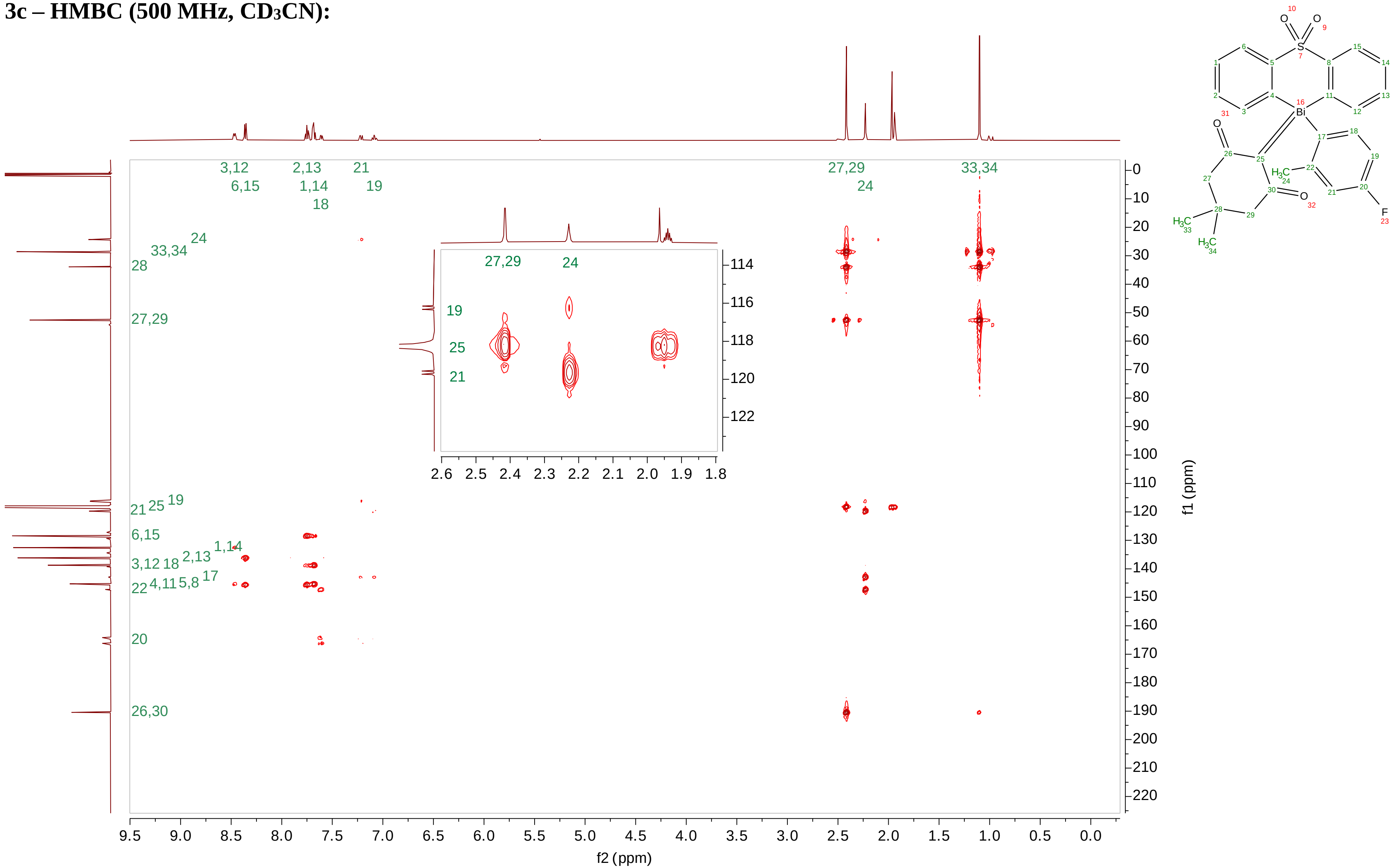

3c - ^{19}F NMR (376 MHz, CD_3CN):

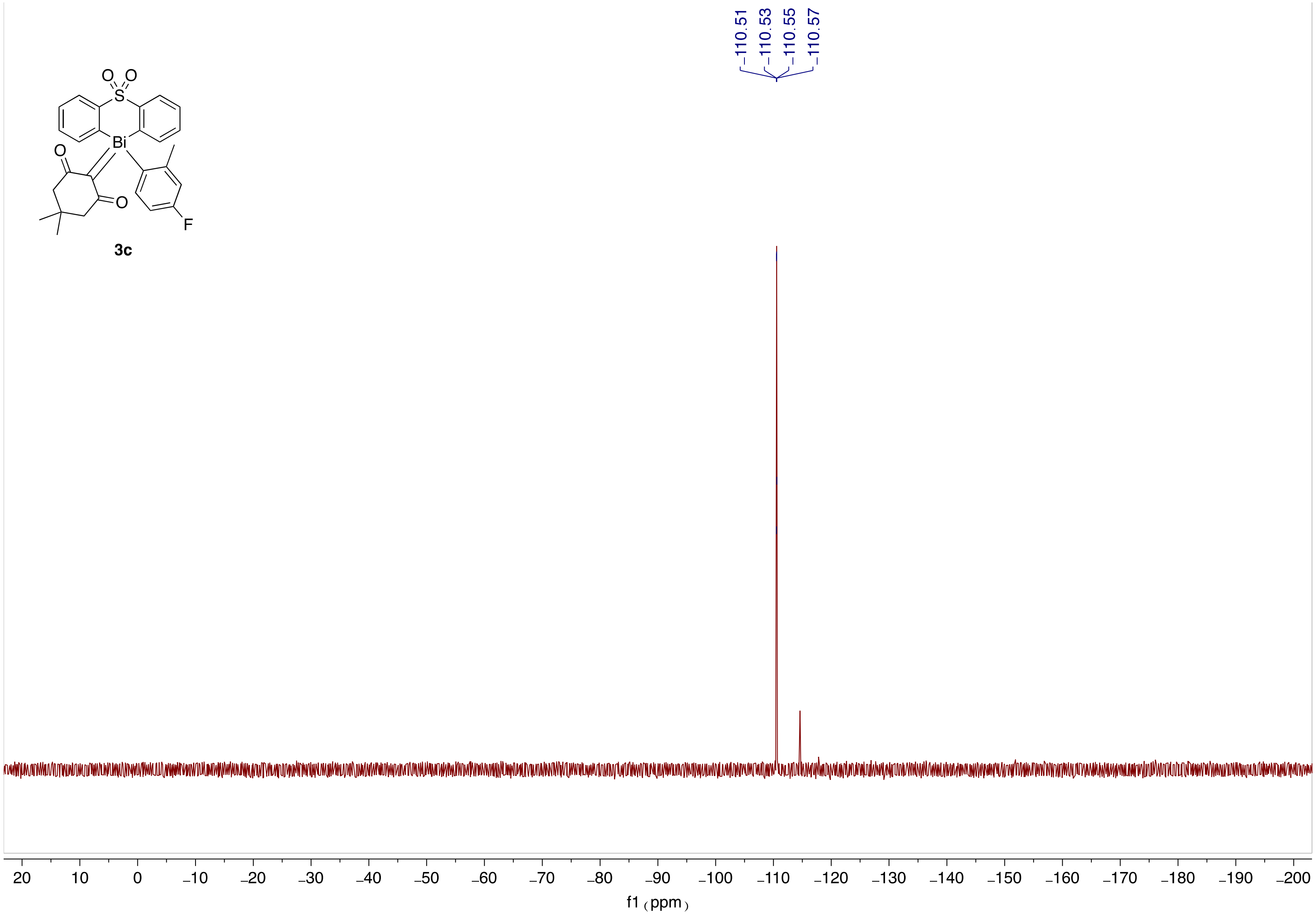

3c - ^{19}F NMR (376 MHz, $CDCl_3$):

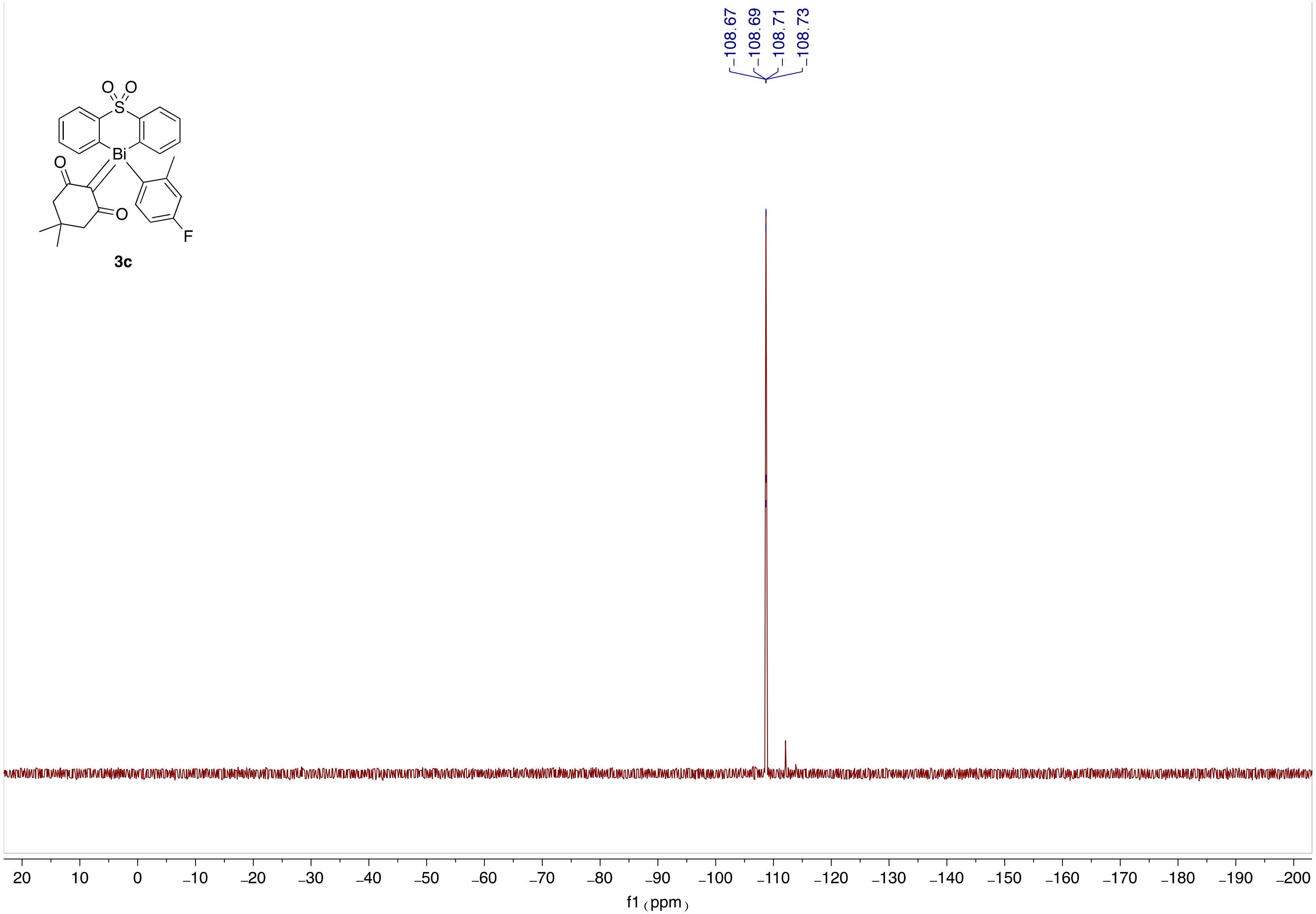

3d - ^{1}H NMR (500 MHz, CD$_3$CN):

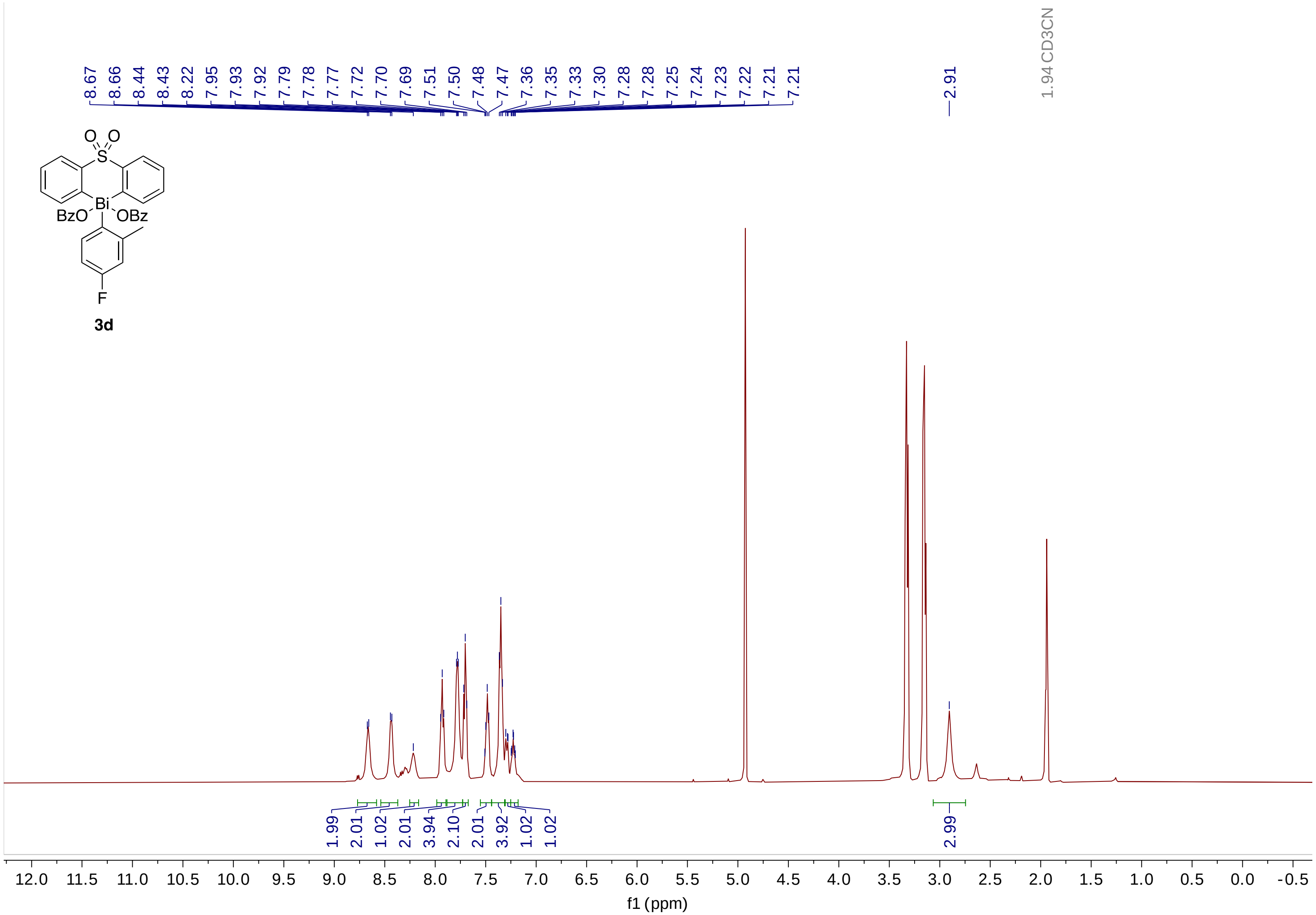

3d - ^{19}F NMR (376 MHz, CD_3CN):

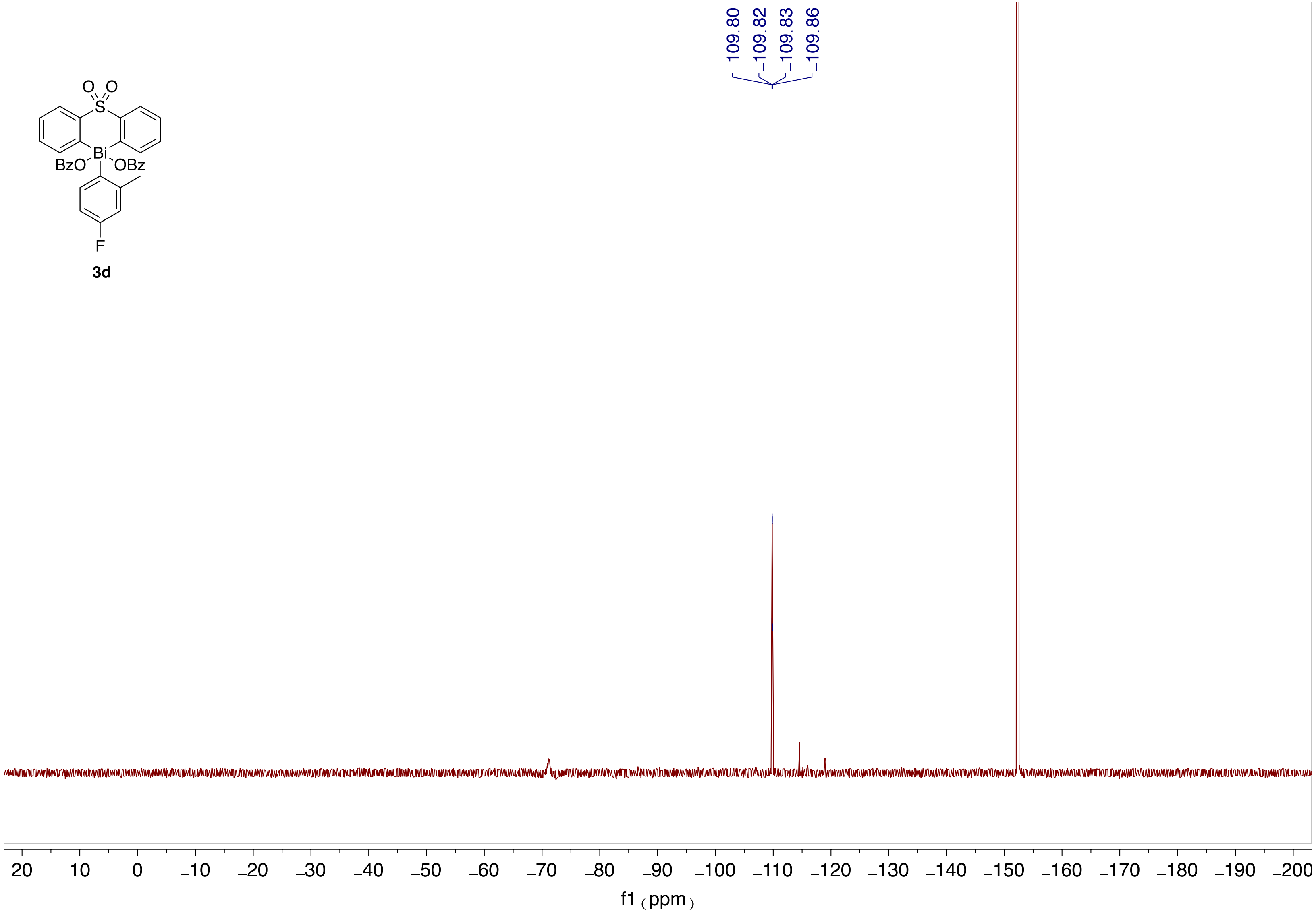

4 - ^{1}H NMR (400 MHz, CDCl$_3$):

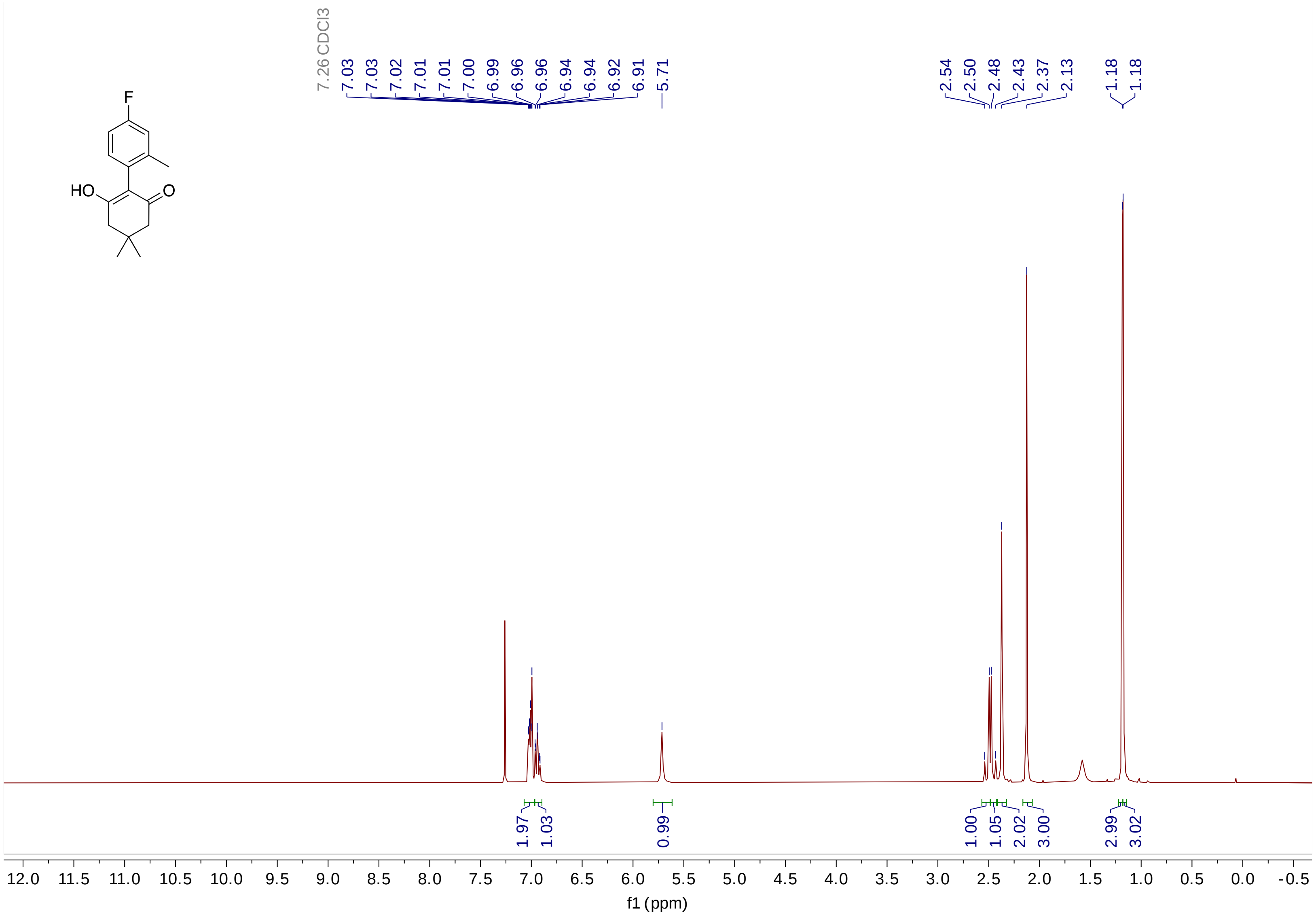

4 - $^{13}C\{^1H\}$ NMR (101 MHz, CDCl$_3$):

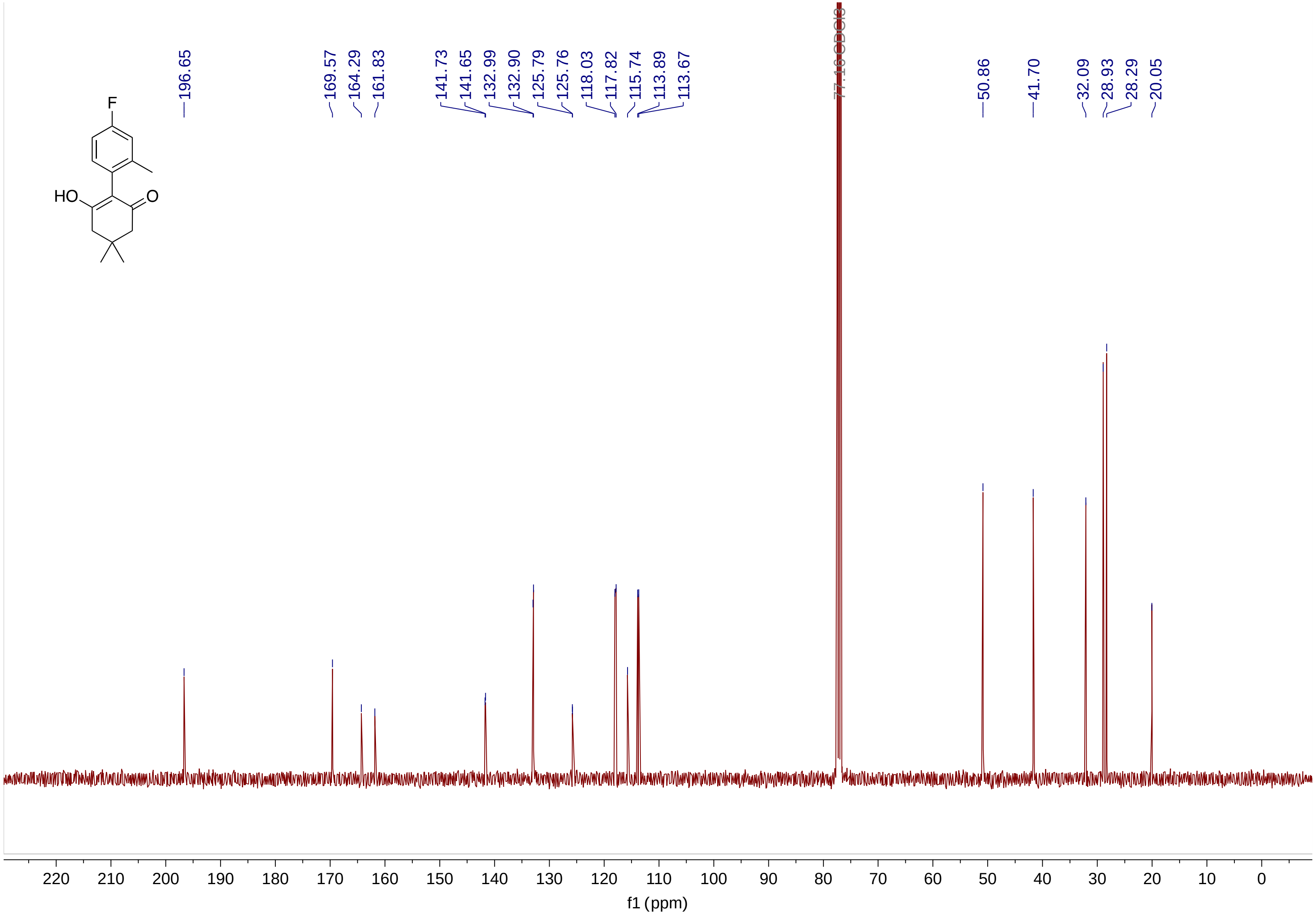

4 - ^{19}F NMR (377 MHz, $CDCl_3$):

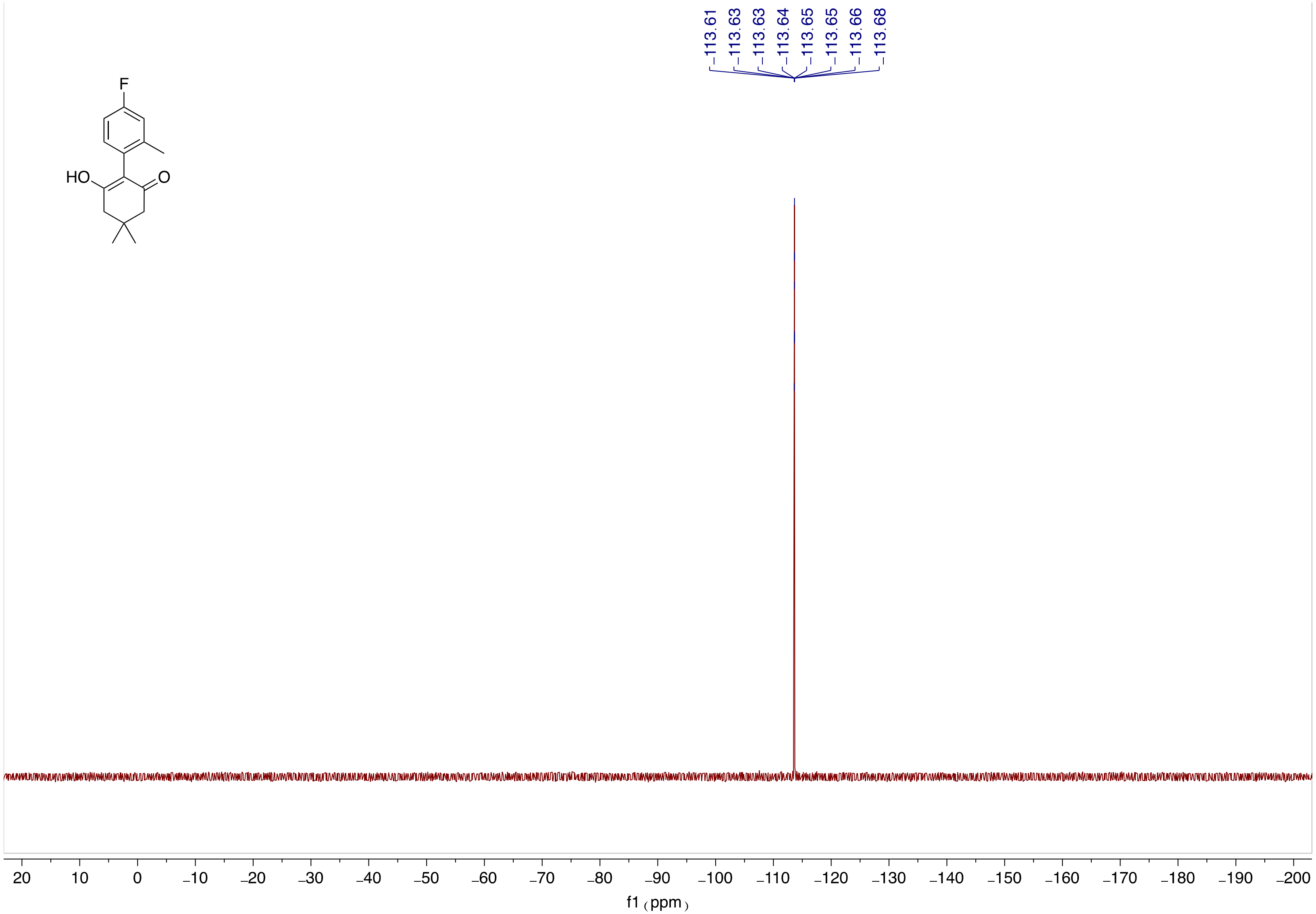

4′ (authentic sample) - ^{1}H NMR (400 MHz, $CDCl_3$):

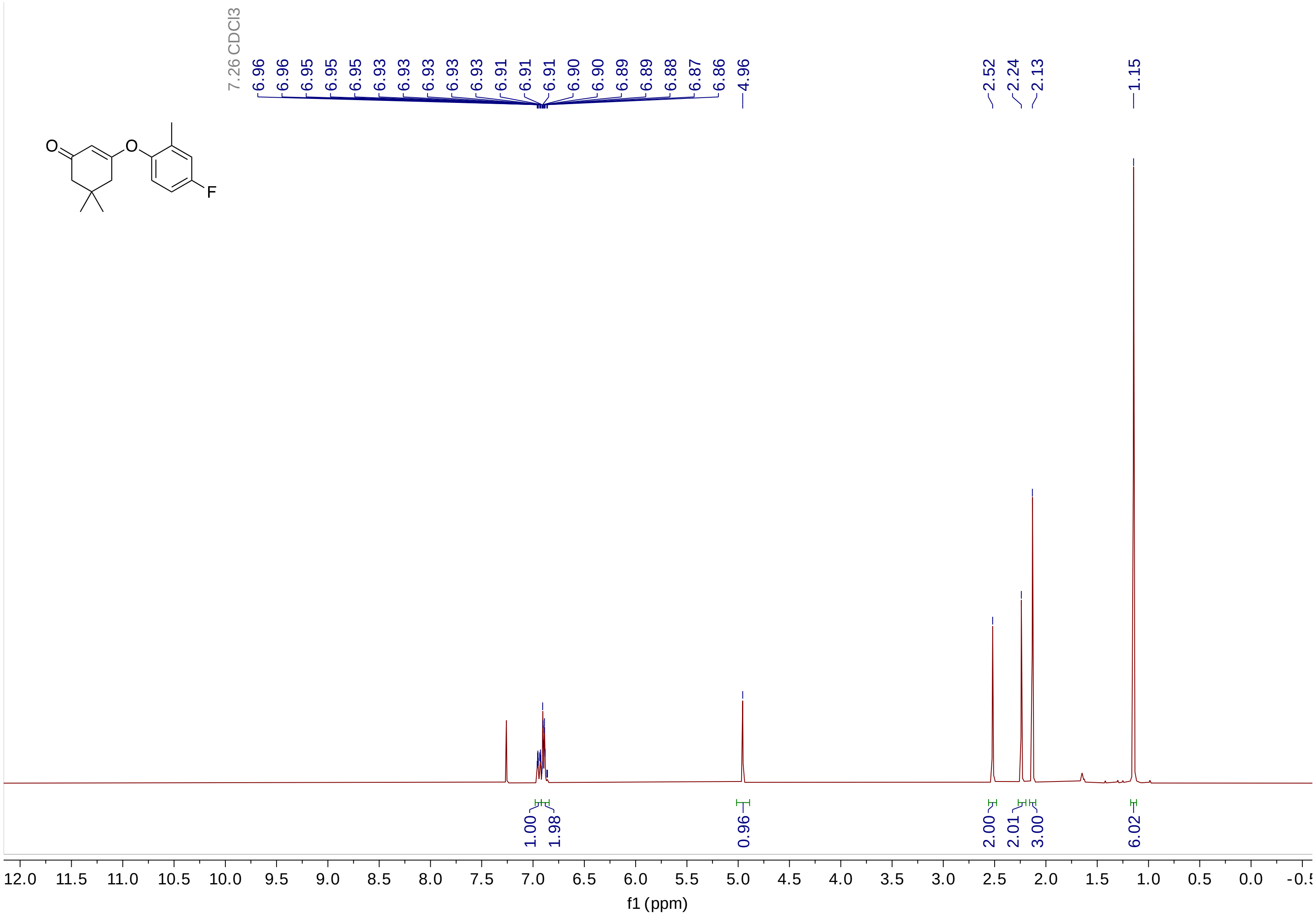

4′ (authentic sample) - $^{13}C\{^{1}H\}$ NMR (101 MHz, $CDCl_3$):

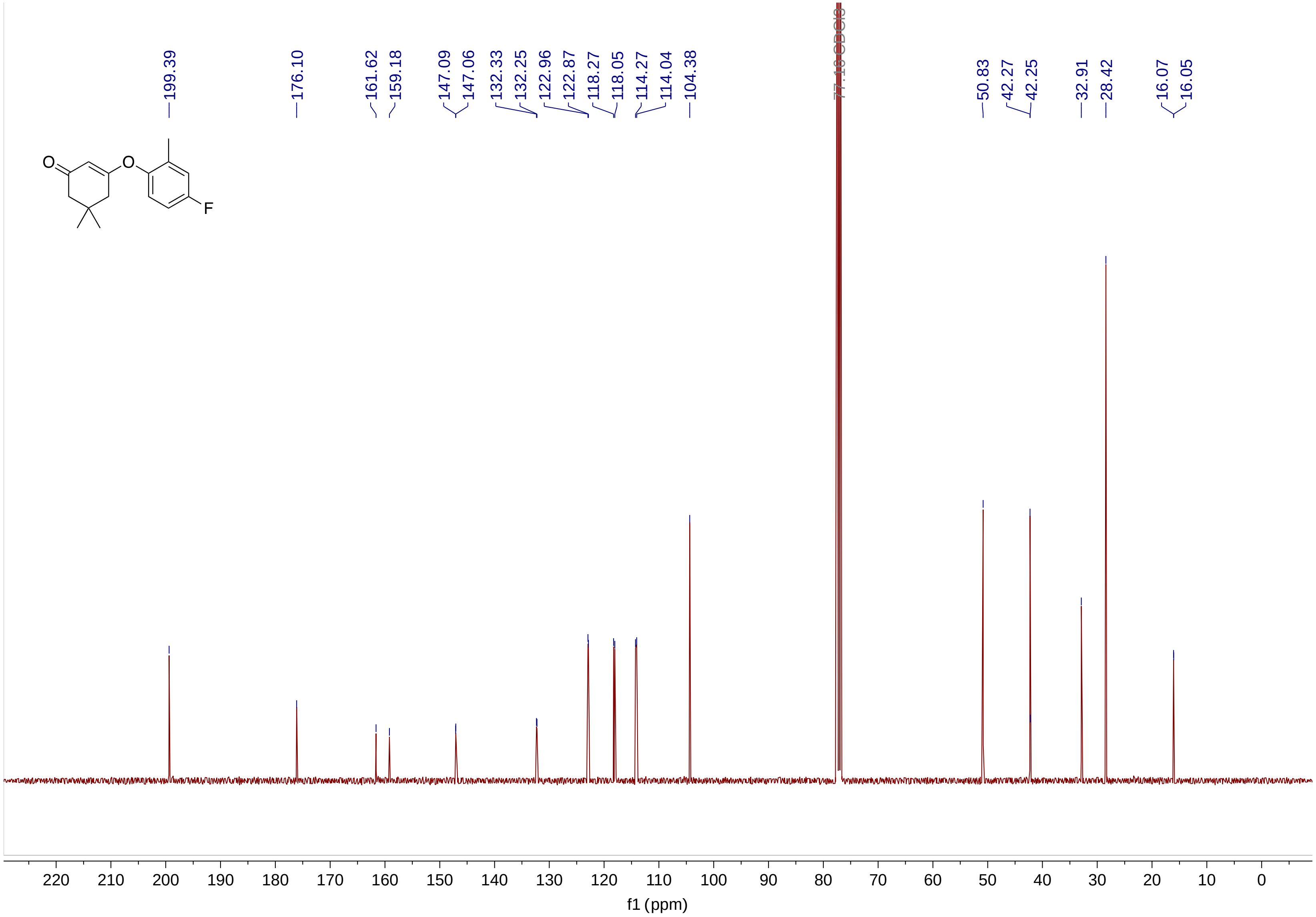

4′ (authentic sample) - ^{19}F NMR (376 MHz, CDCl$_3$):

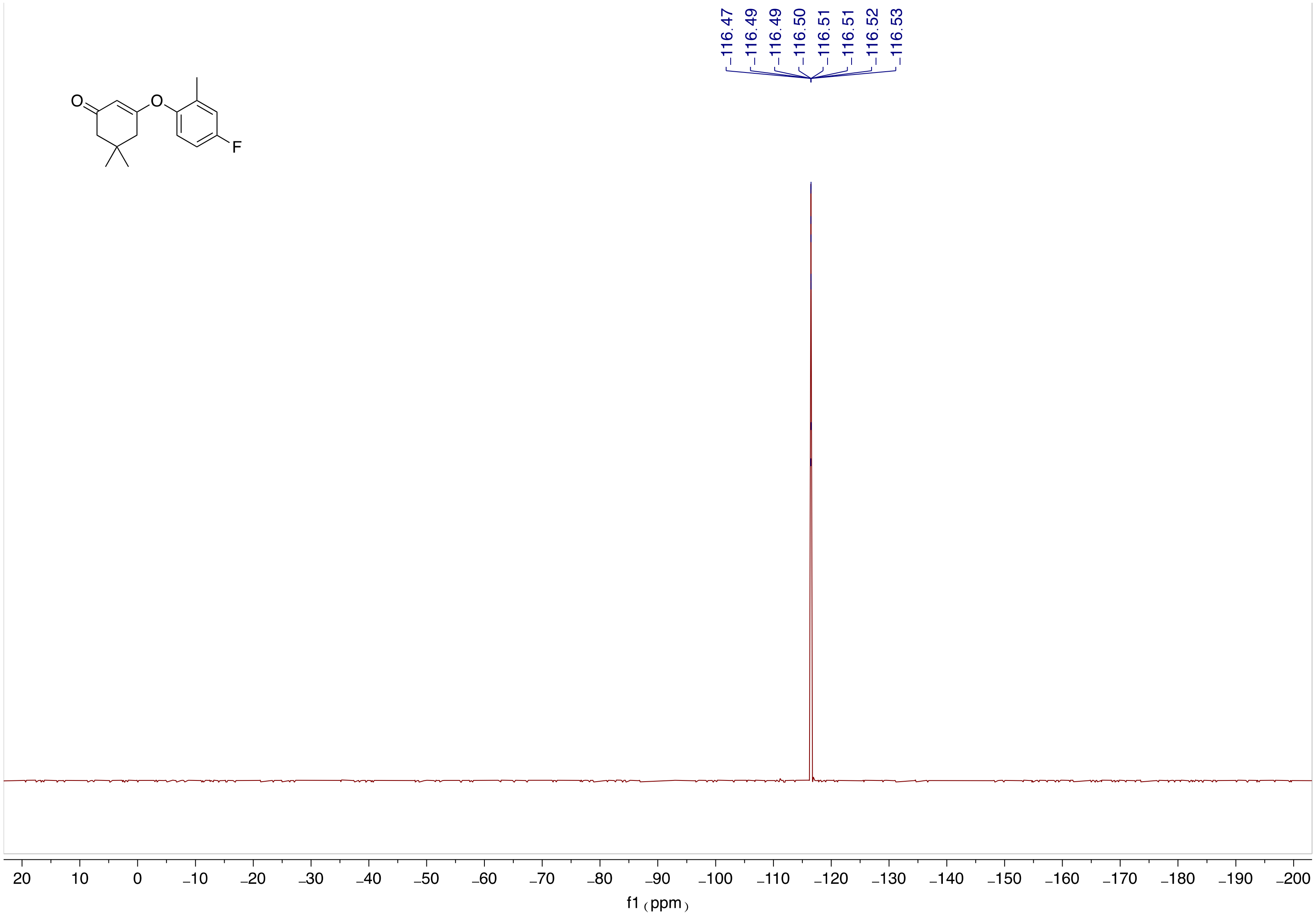

5 - ¹H NMR (500 MHz, CDCl₃):

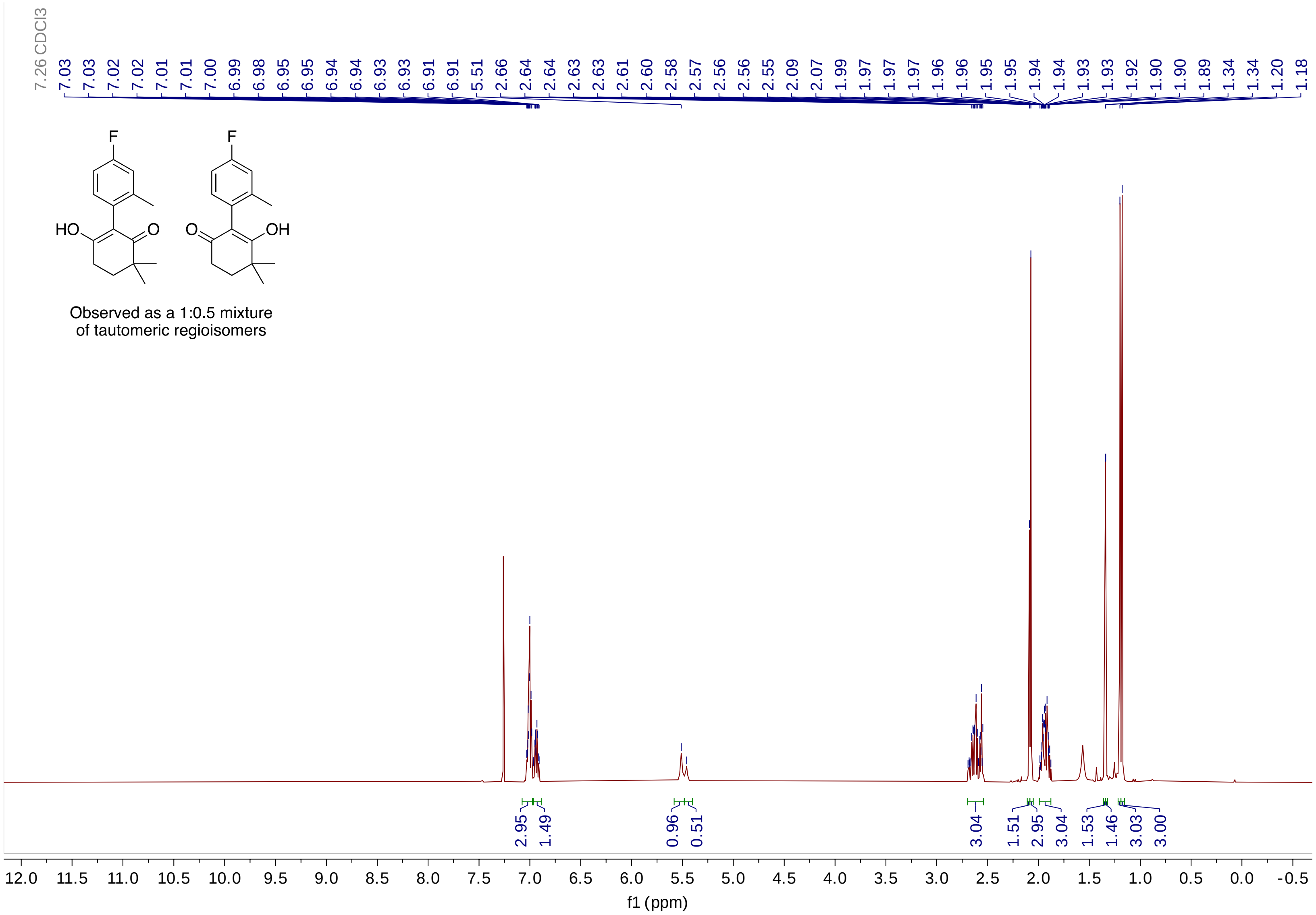

5 - $^{13}C\{^1H\}$ NMR (126 MHz, $CDCl_3$):

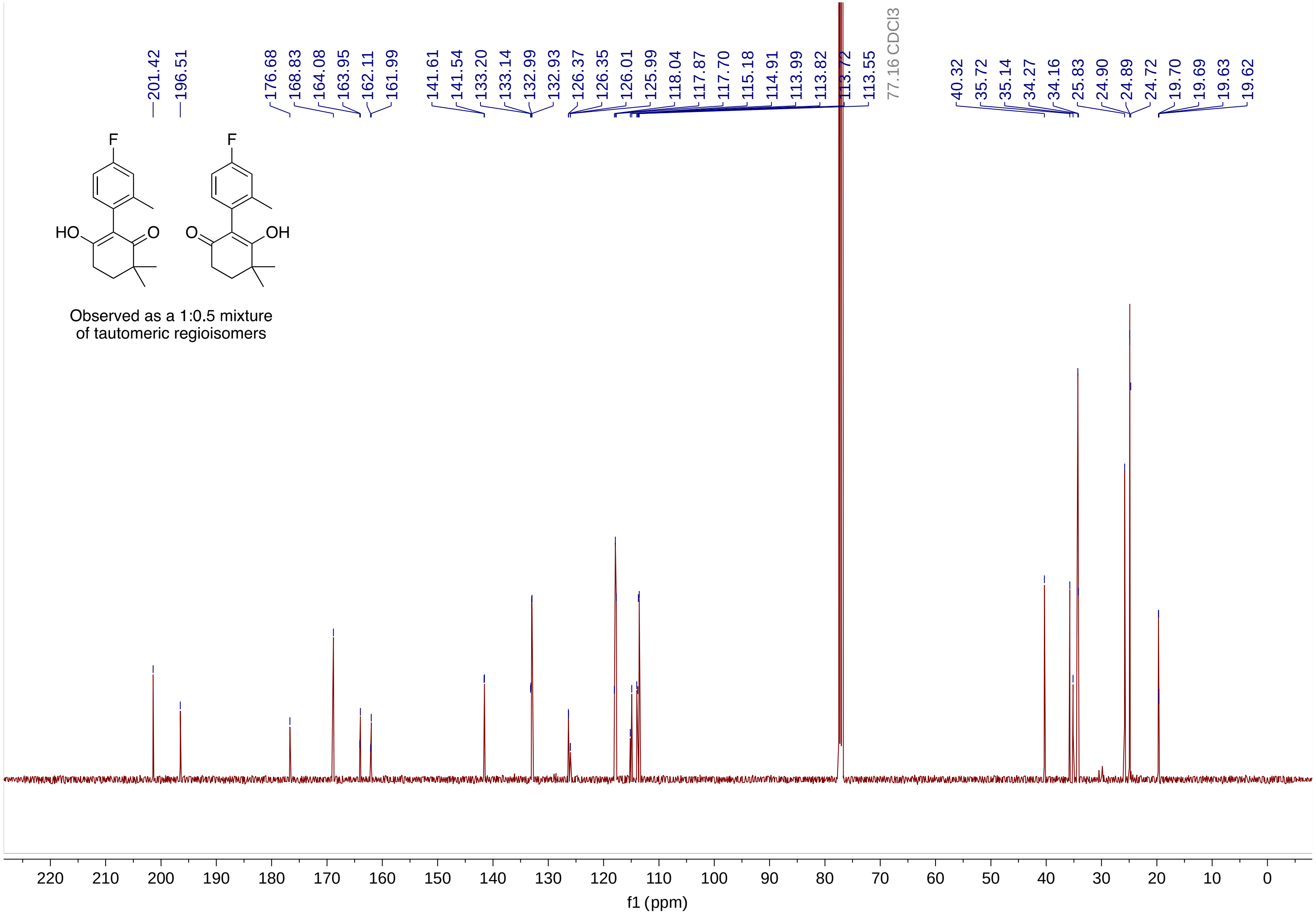

5 - ^{19}F NMR (471 MHz, $CDCl_3$):

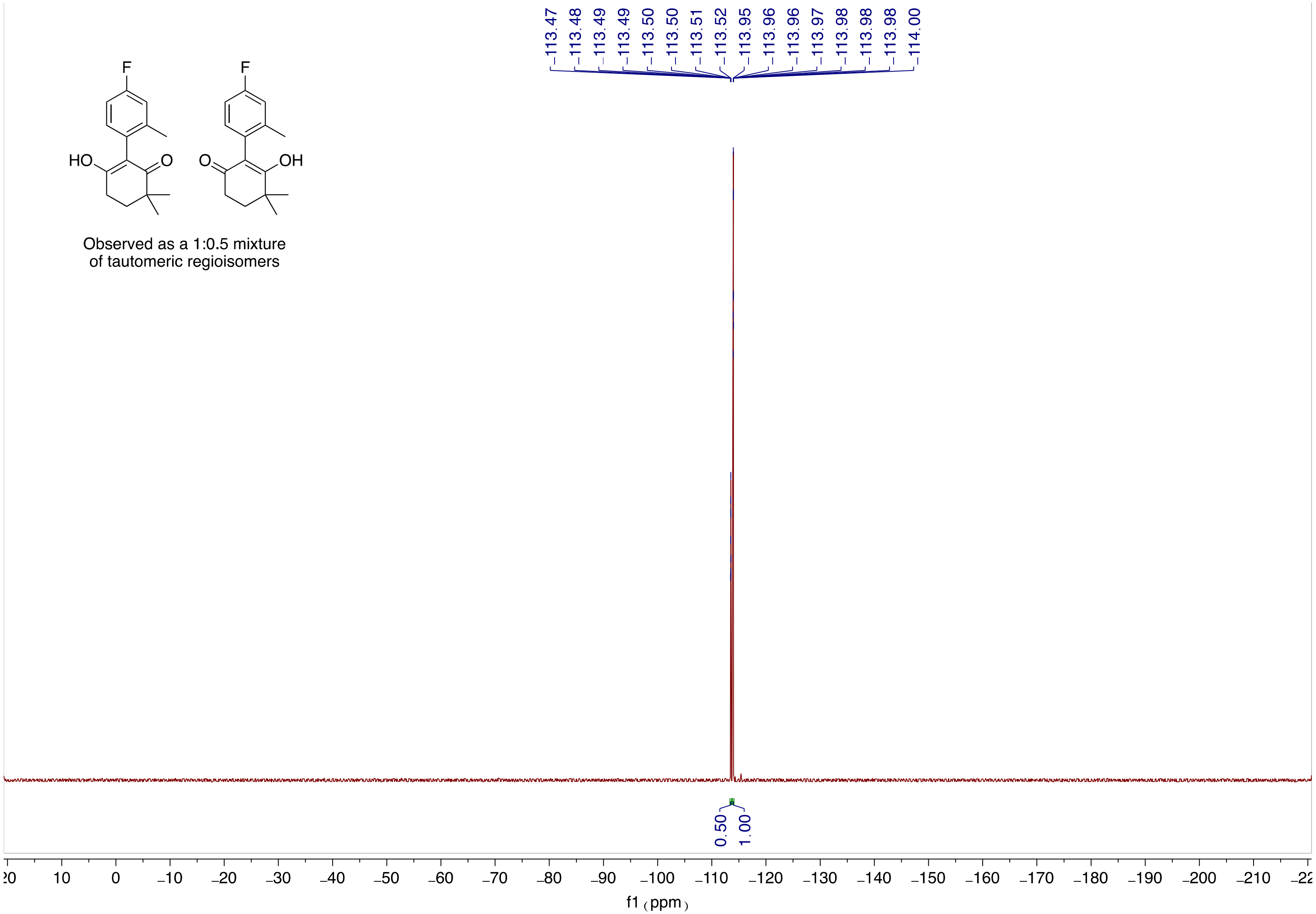

6 - ^{1}H NMR (500 MHz, $CDCl_3$):

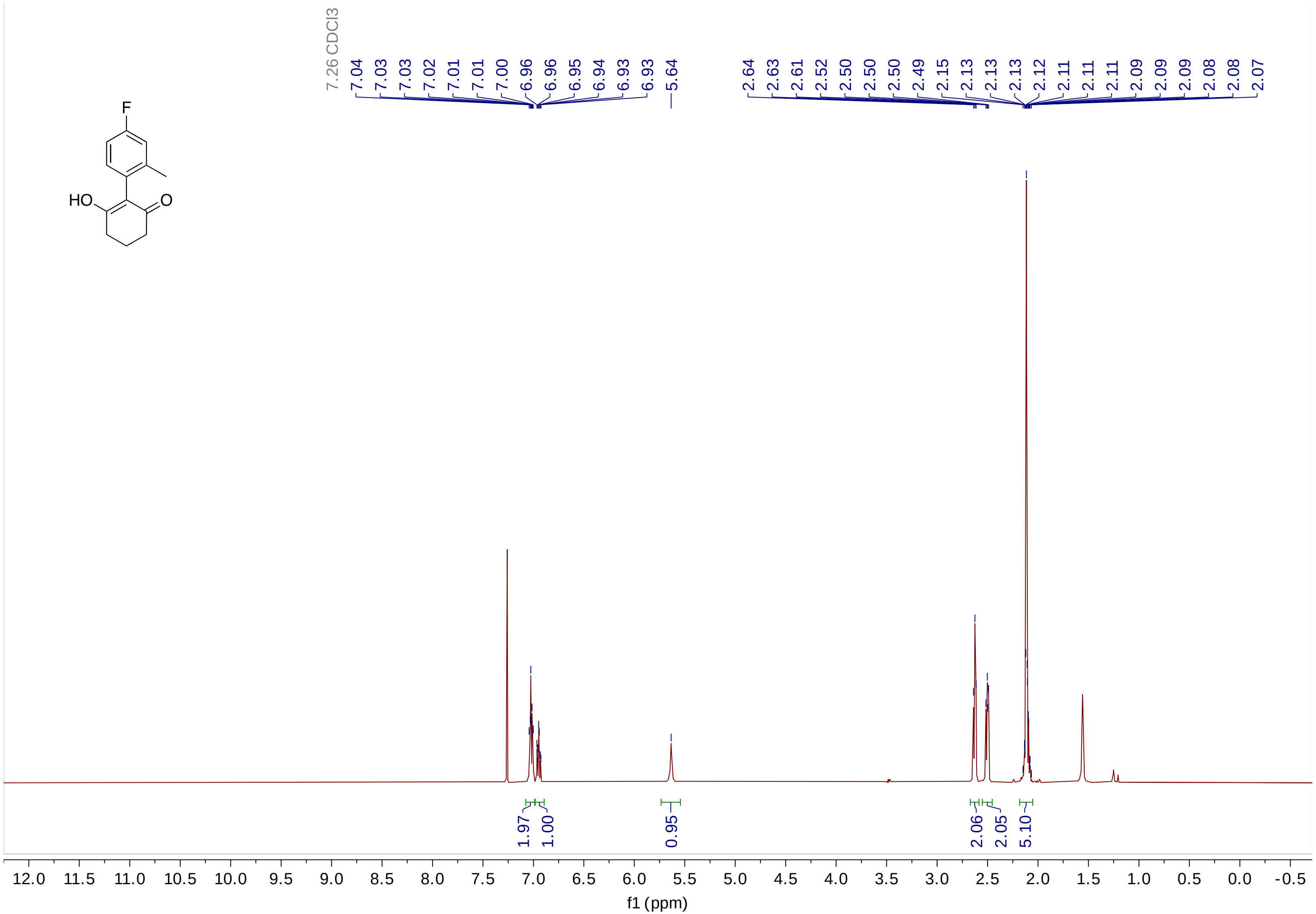

6 - $^{13}C\{^1H\}$ NMR (126 MHz, CDCl$_3$):

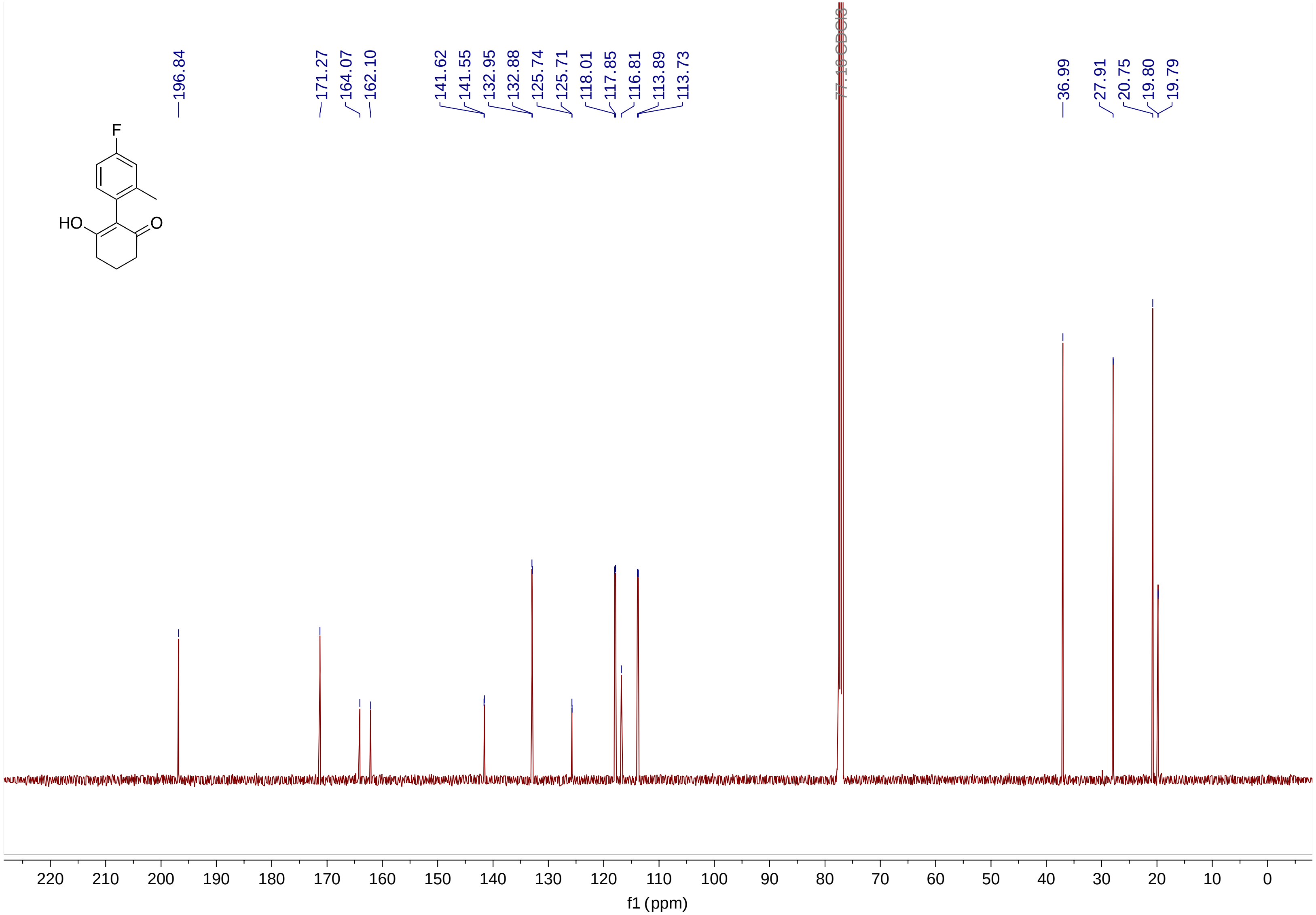

6 - ^{19}F NMR (471 MHz, CDCl$_3$):

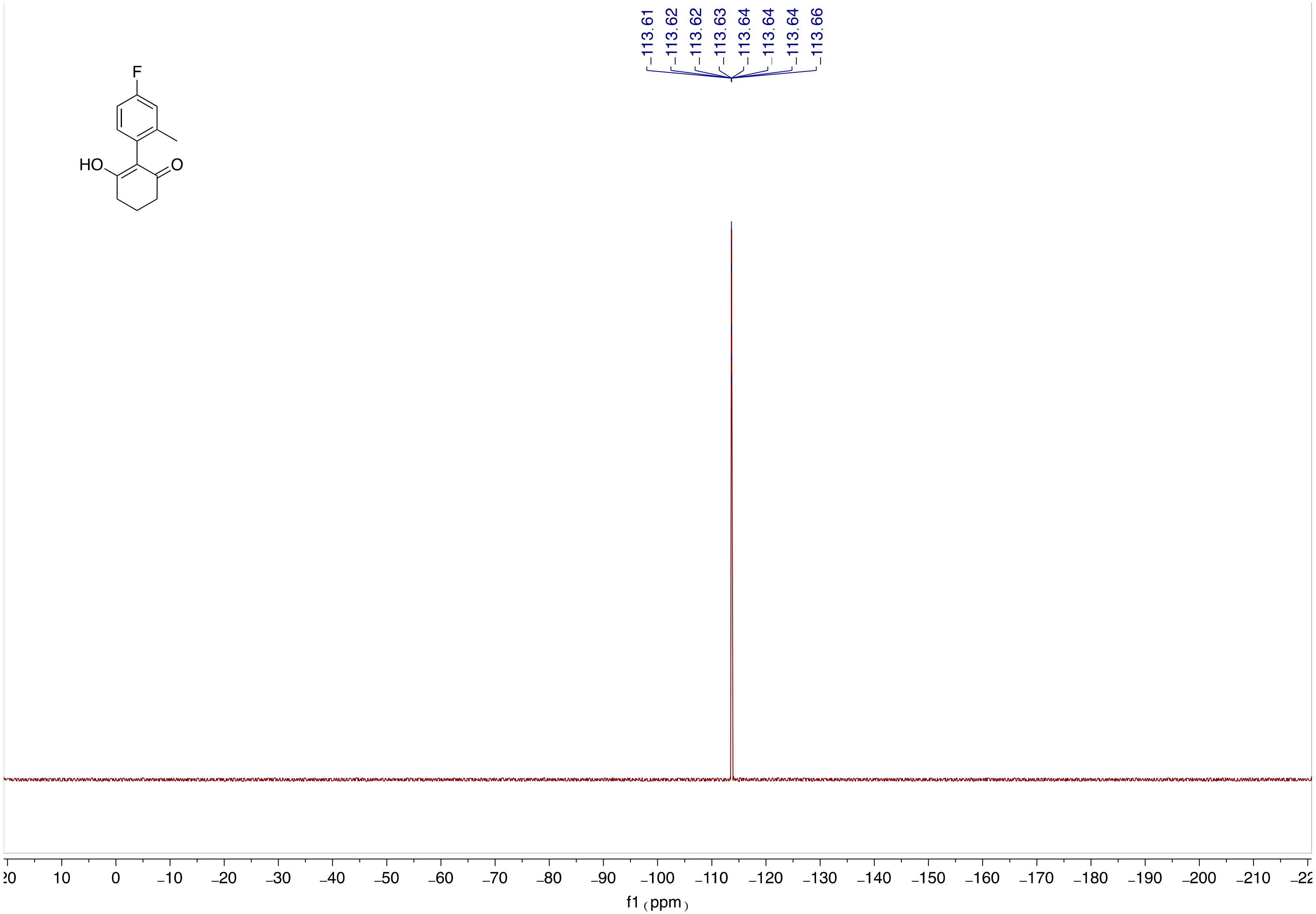

7 - ^{1}H NMR (500 MHz, CDCl₃):

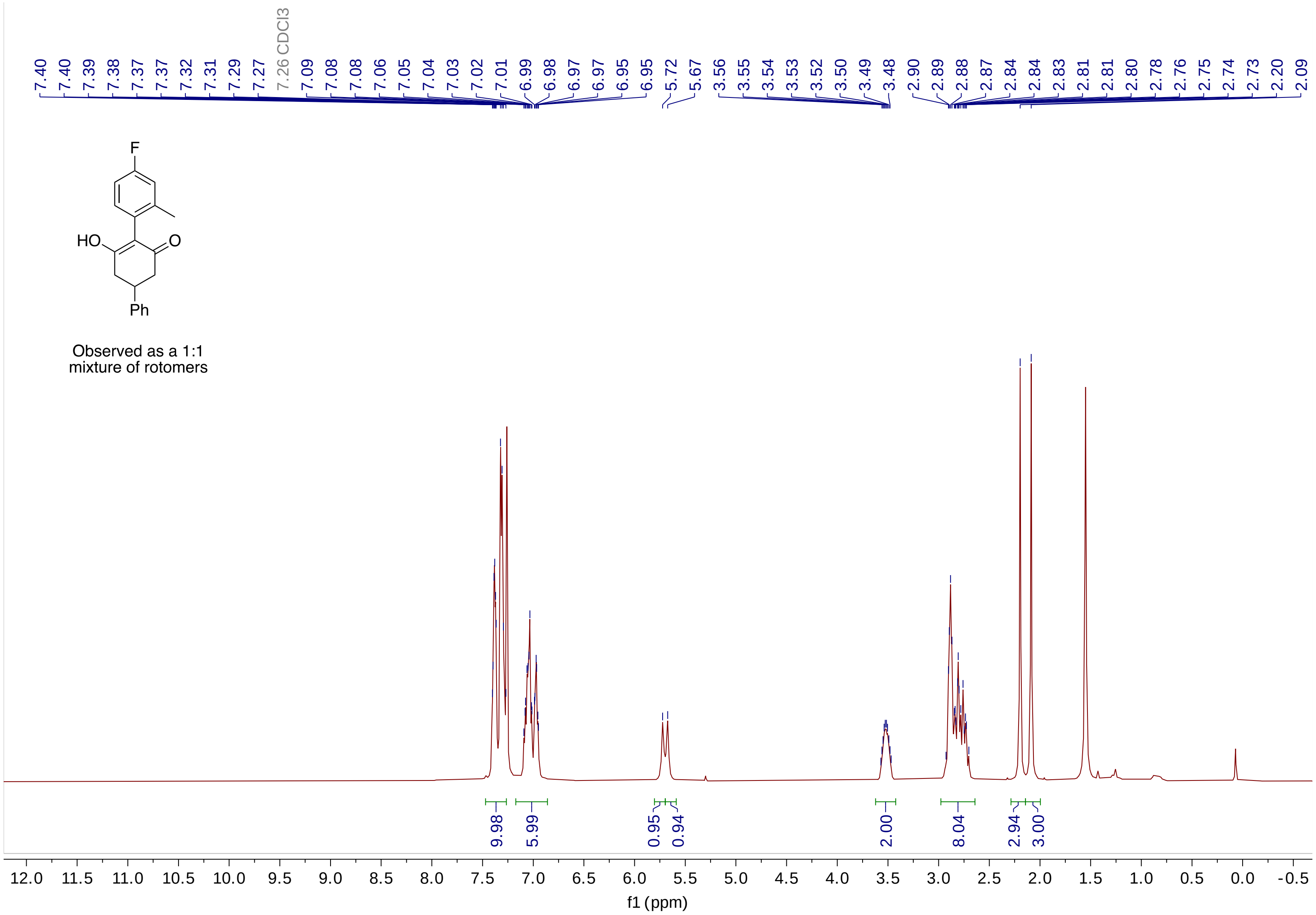

7 - $^{13}C\{^{1}H\}$ NMR (126 MHz, $CDCl_3$):

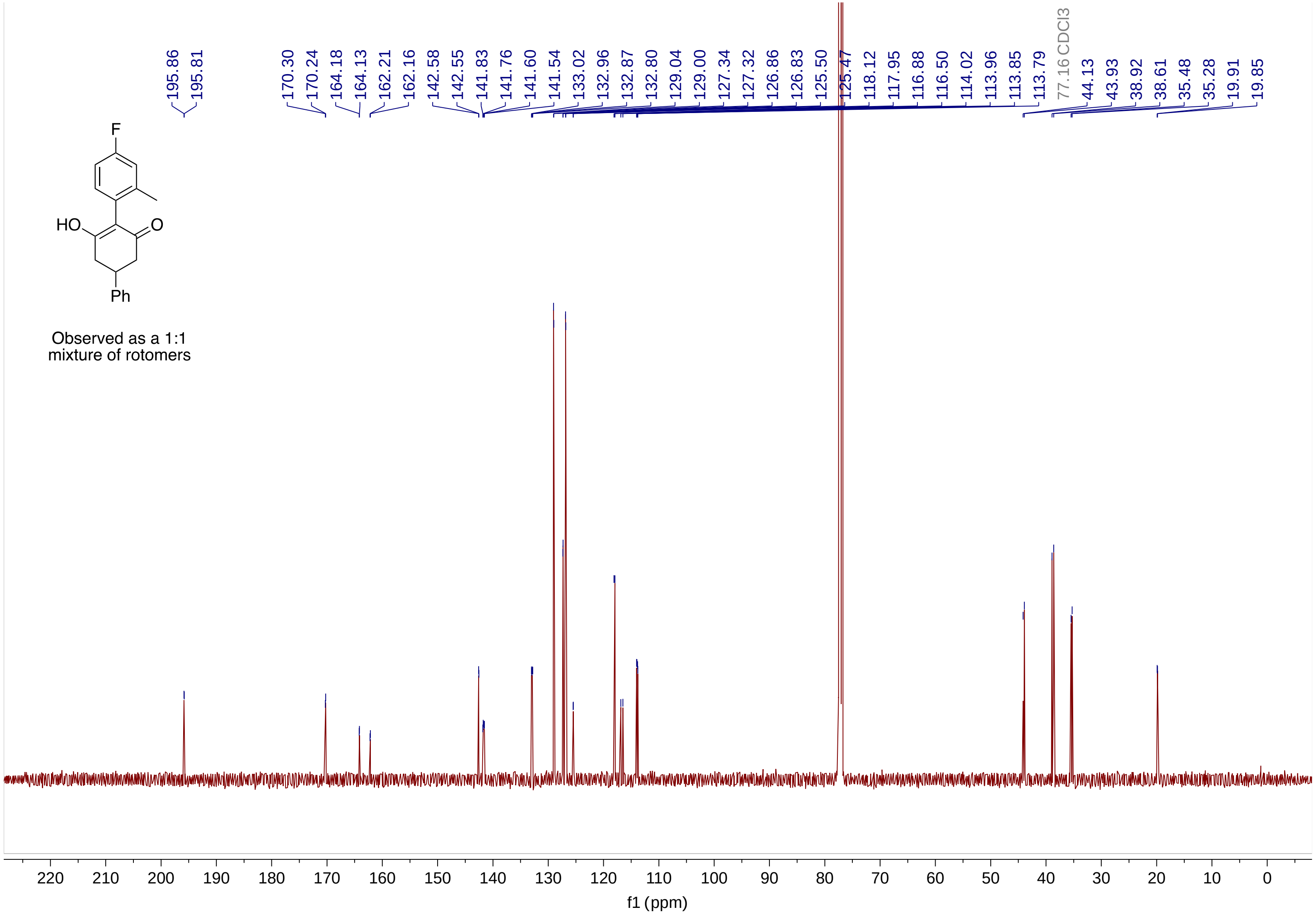

7 - ^{19}F NMR (471 MHz, $CDCl_3$):

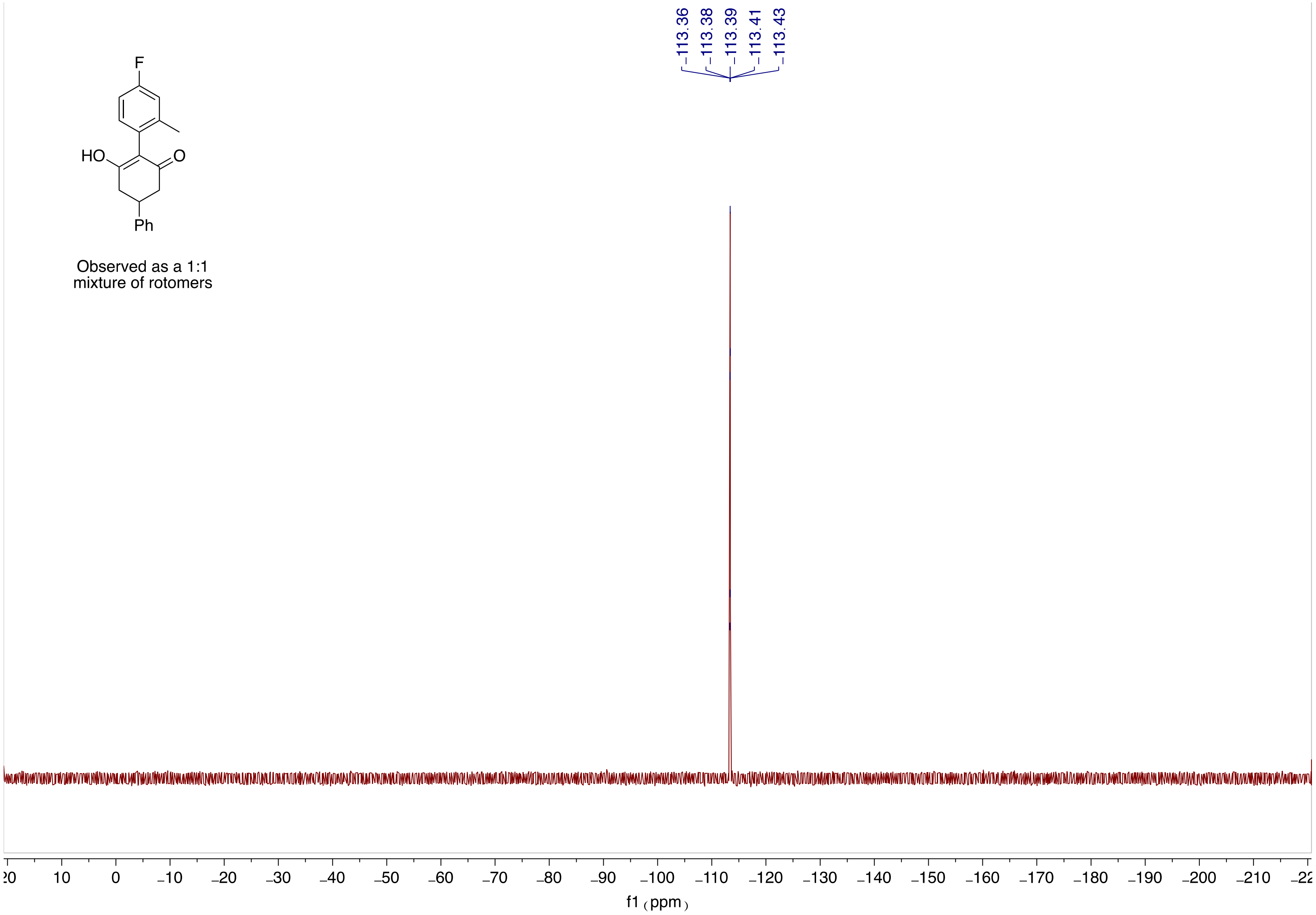

8 - ^{1}H NMR (400 MHz, $CDCl_3$):

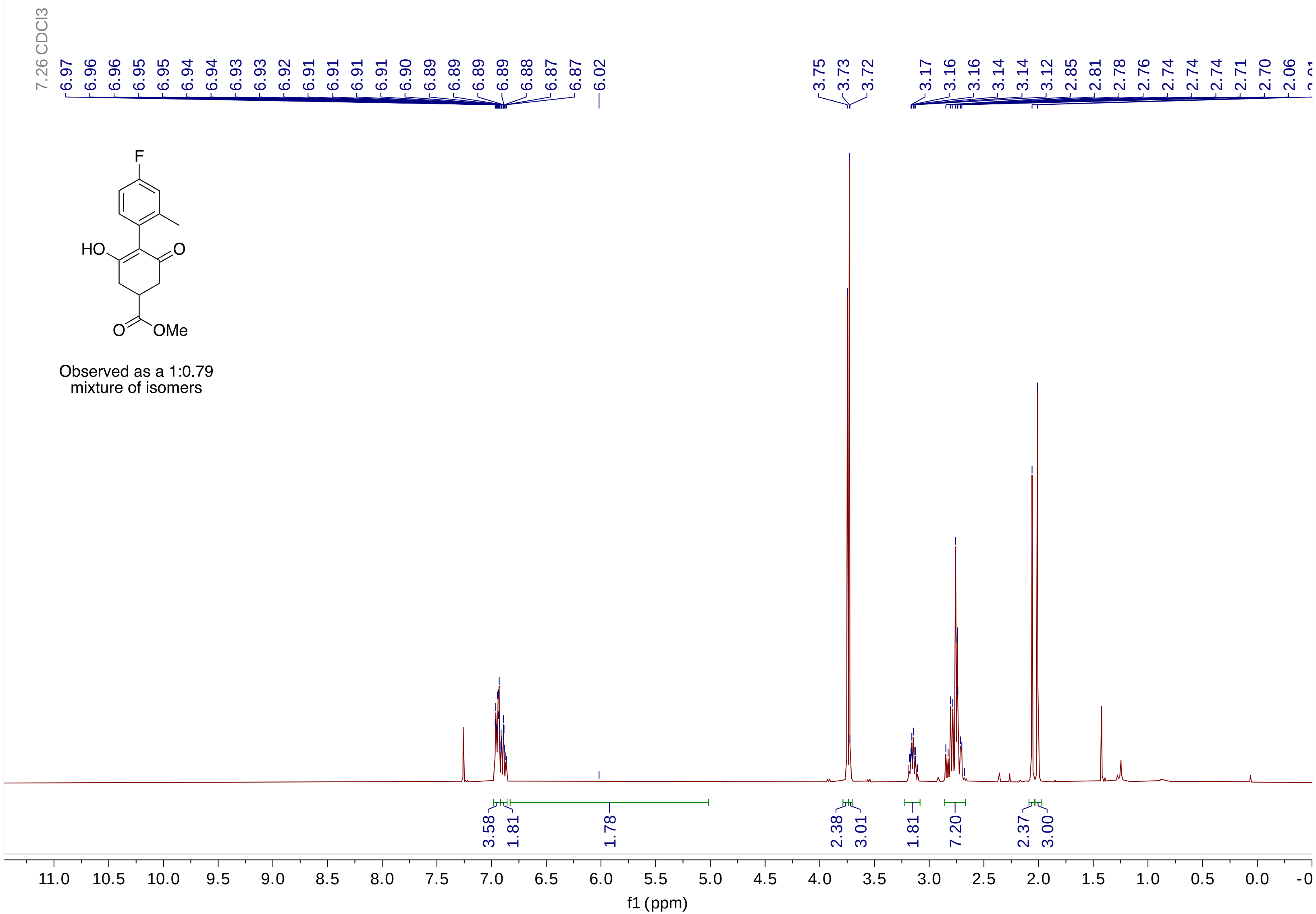

8 - $^{13}C\{^1H\}$ NMR (101 MHz, $CDCl_3$):

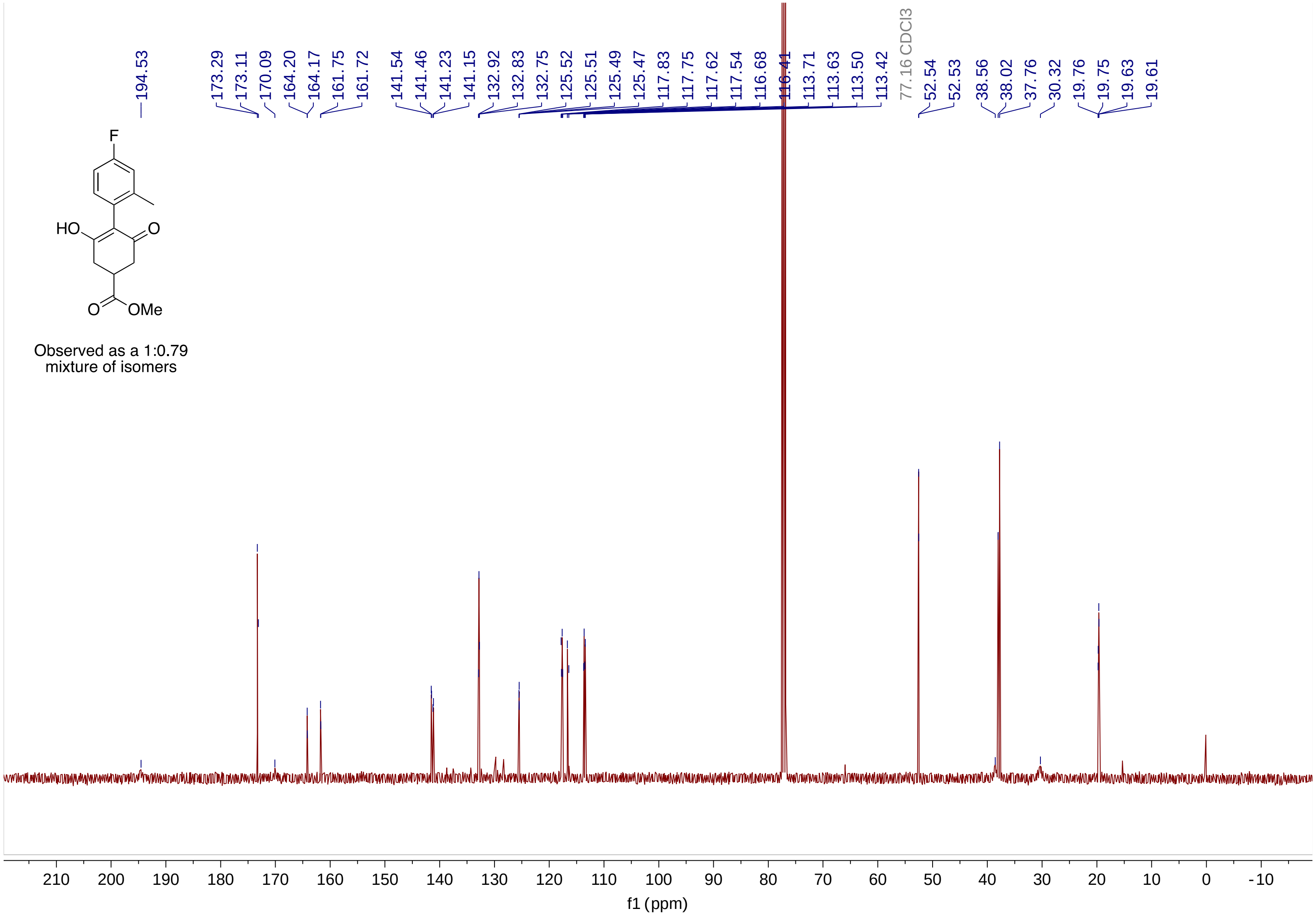

8 - ^{19}F NMR (376 MHz, $CDCl_3$):

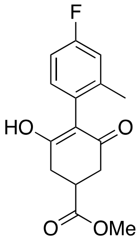

Observed as a 1:0.79 mixture of isomers

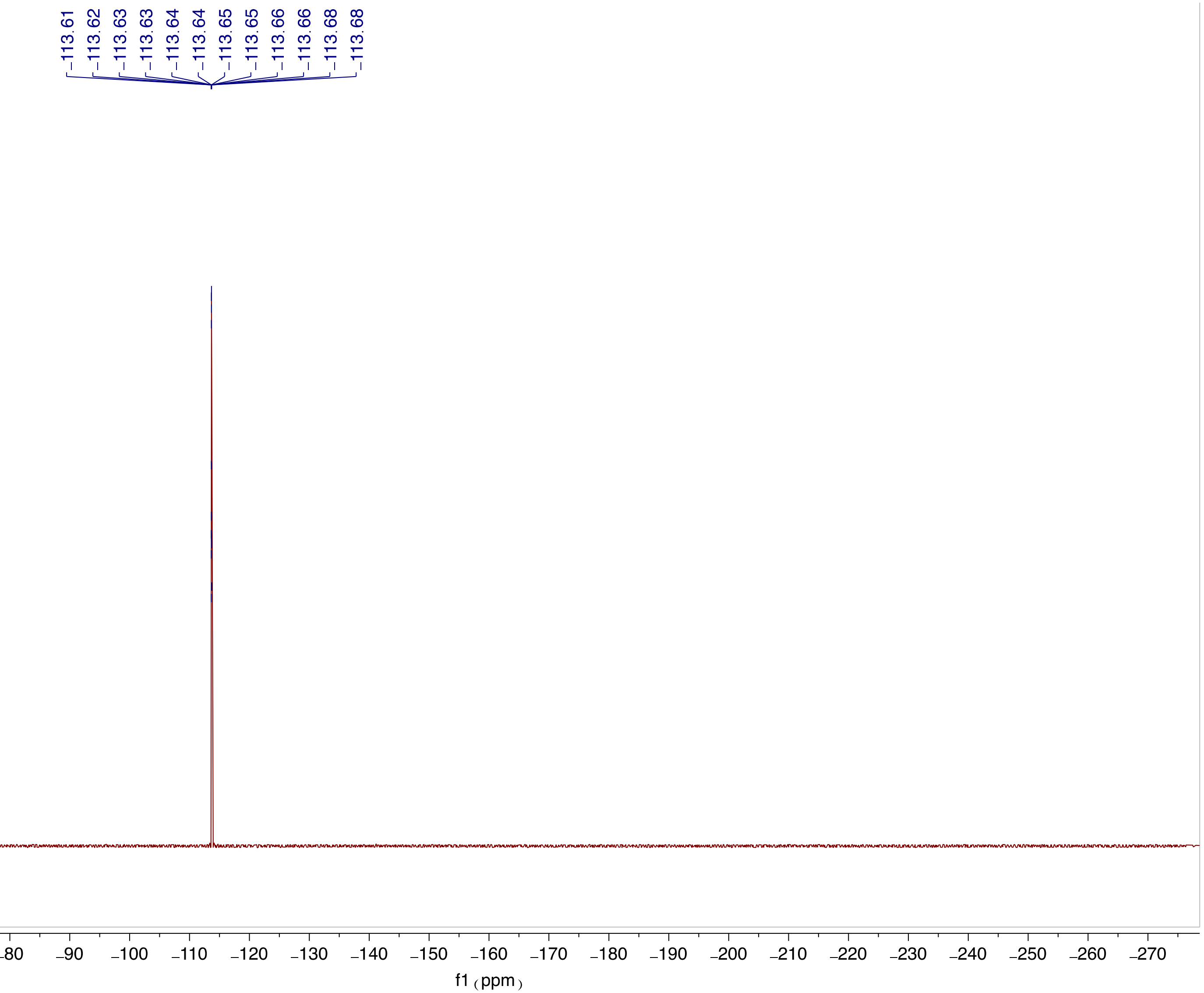

9 - ^{1}H NMR (400 MHz, $CDCl_3$):

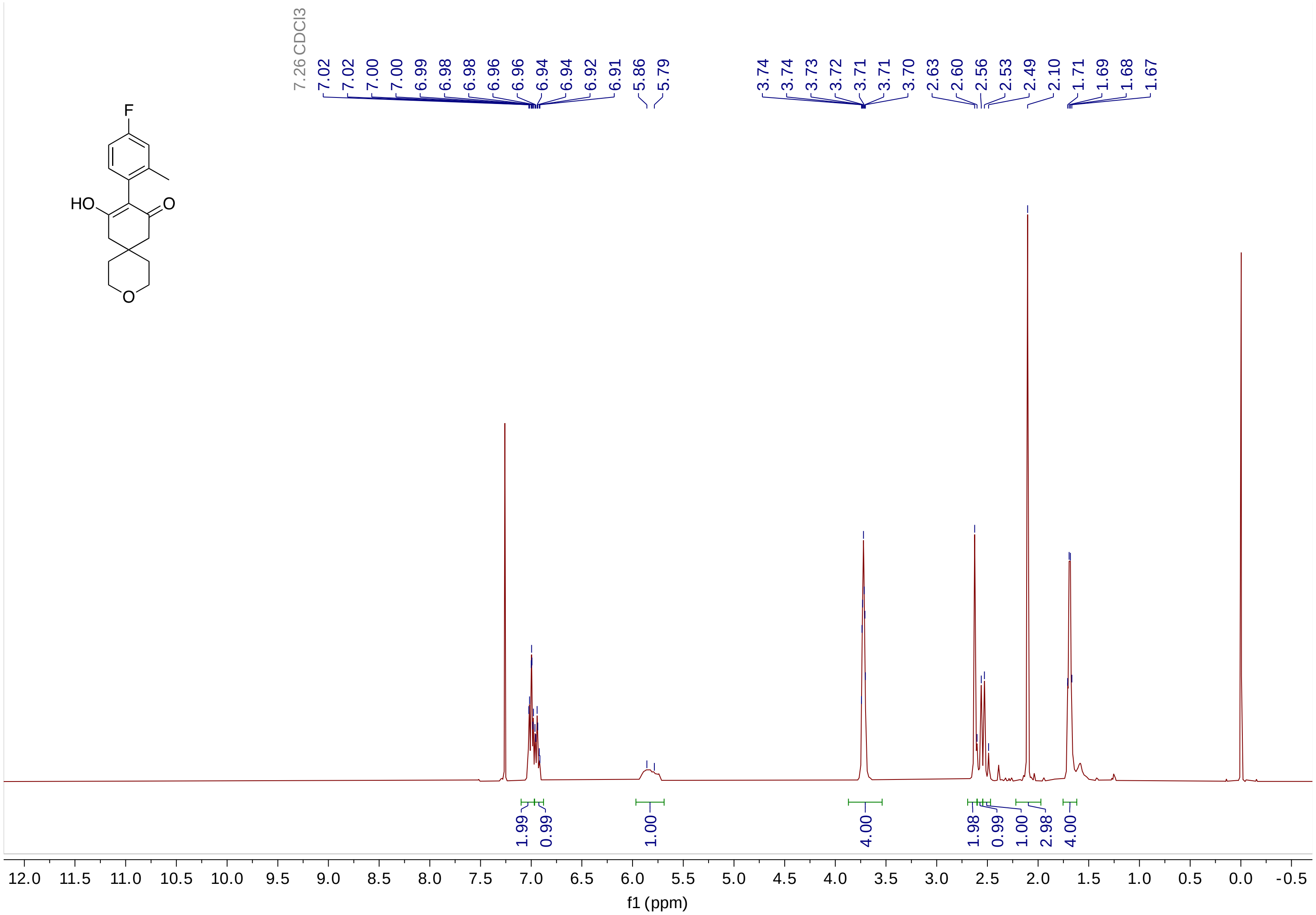

9 - $^{13}C\{^{1}H\}$ NMR (101 MHz, $CDCl_3$):

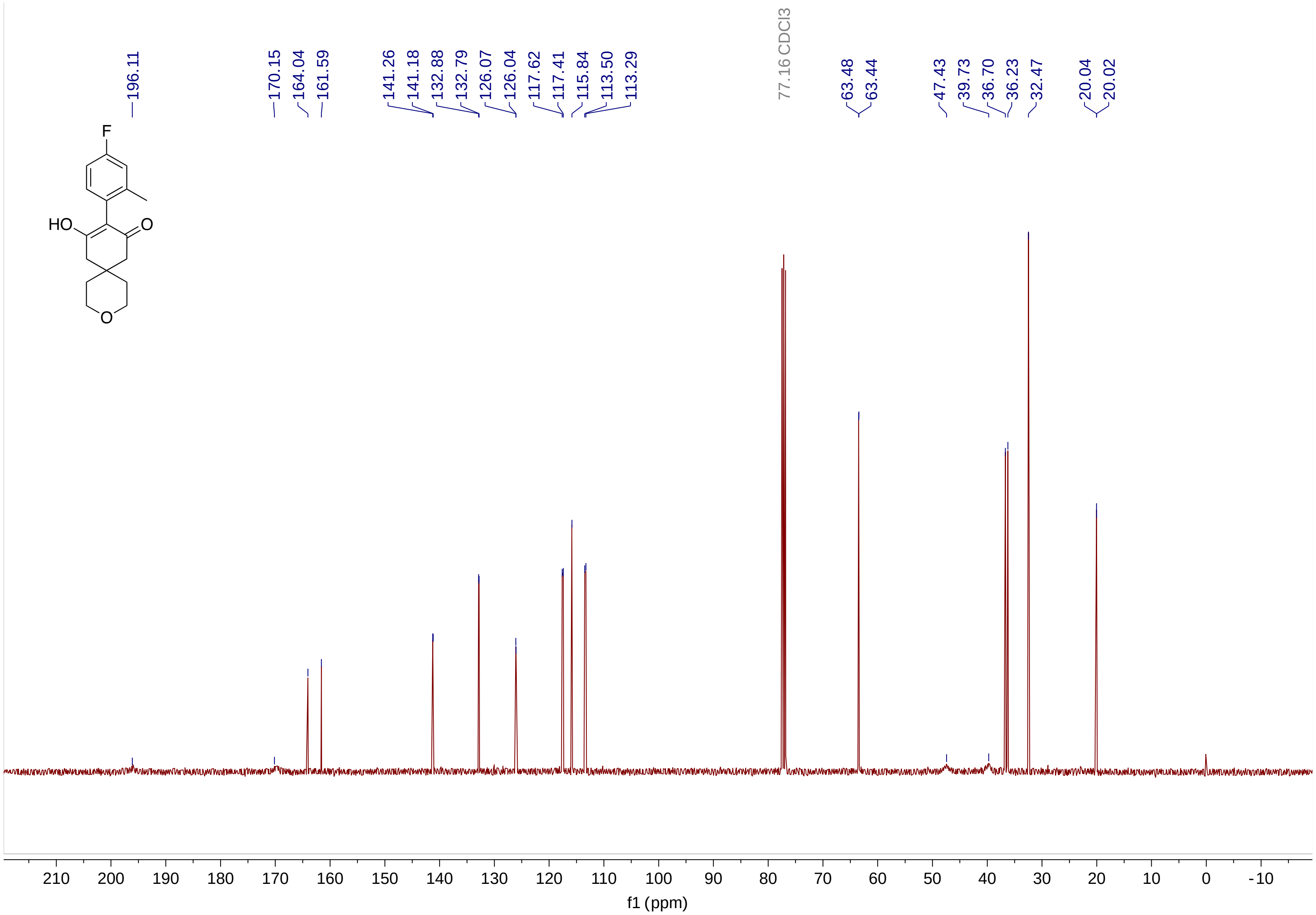

9 - ^{19}F NMR (376 MHz, CDCl$_3$):

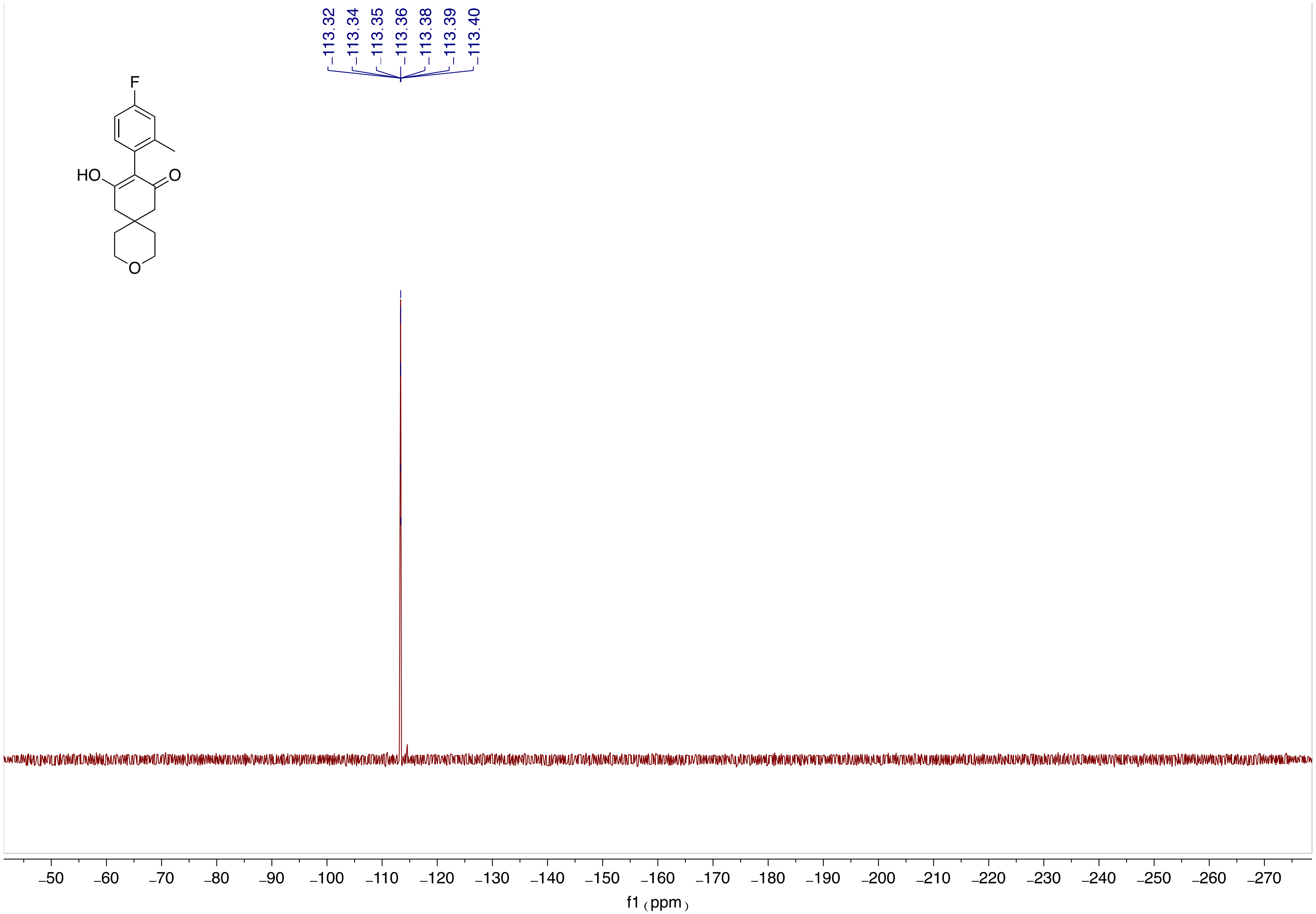

10 - ^{1}H NMR (400 MHz, $CDCl_3$):

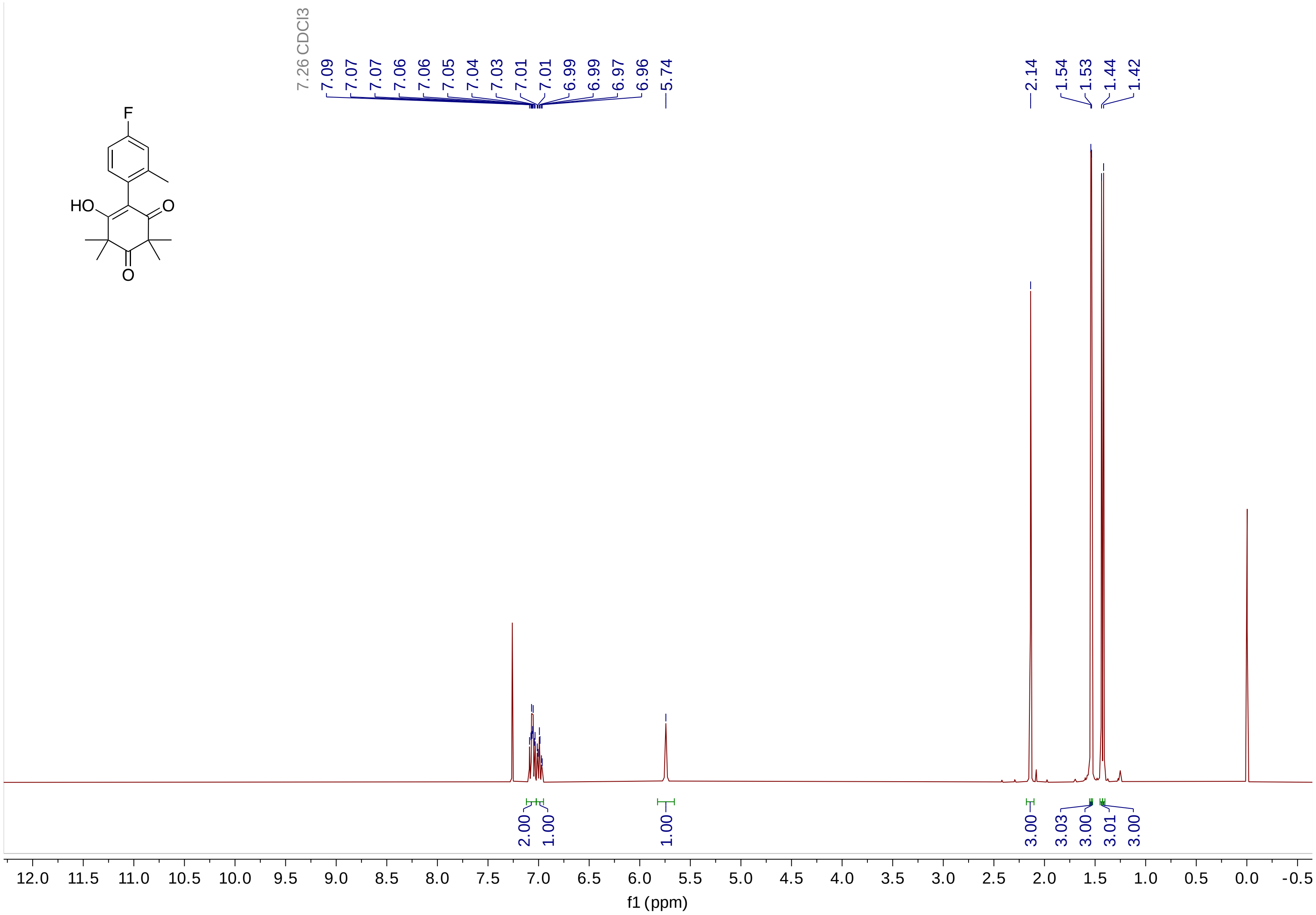

10 - $^{13}C\{^{1}H\}$ NMR (101 MHz, $CDCl_3$):

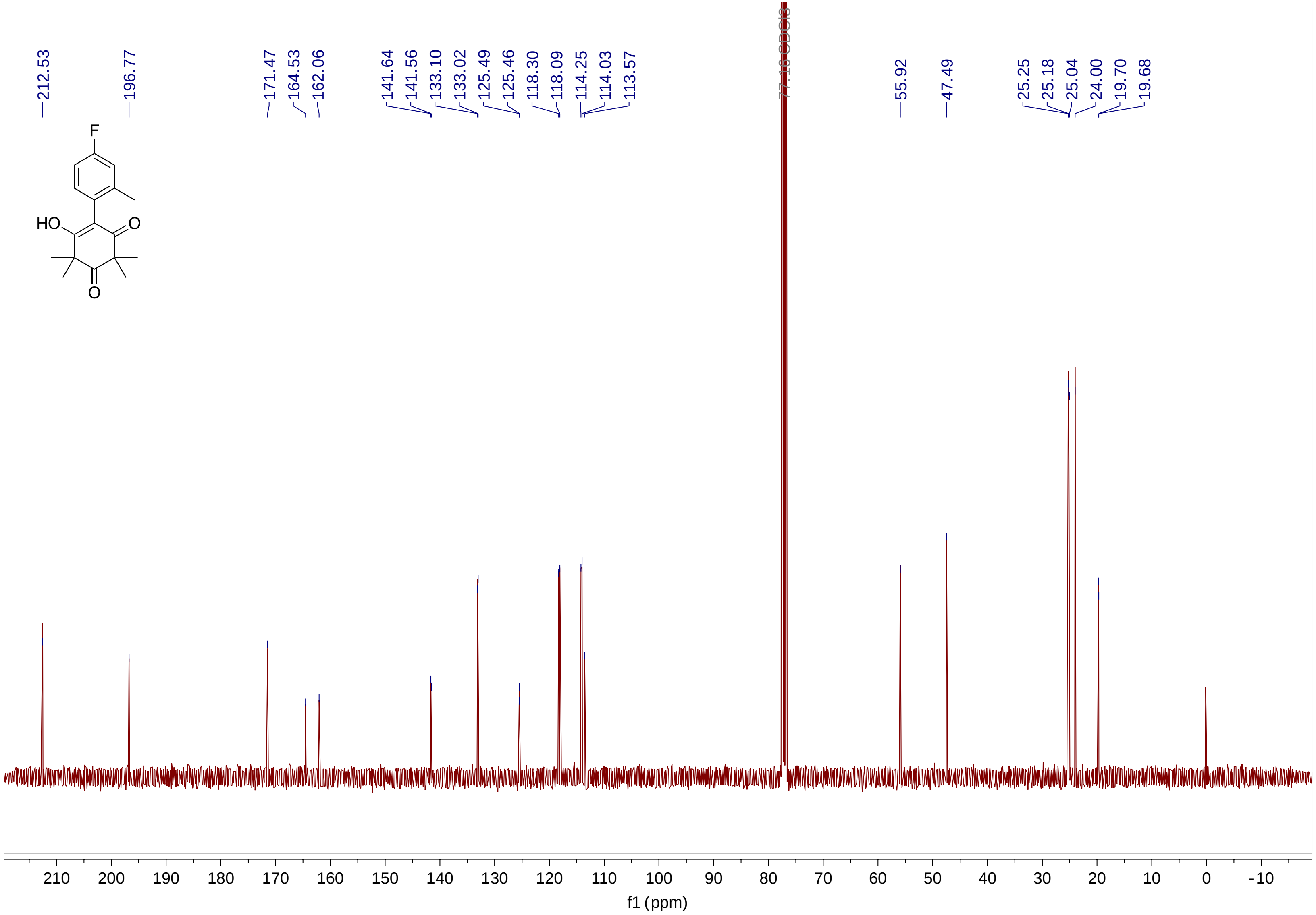

10 - ^{19}F NMR (376 MHz, $CDCl_3$):

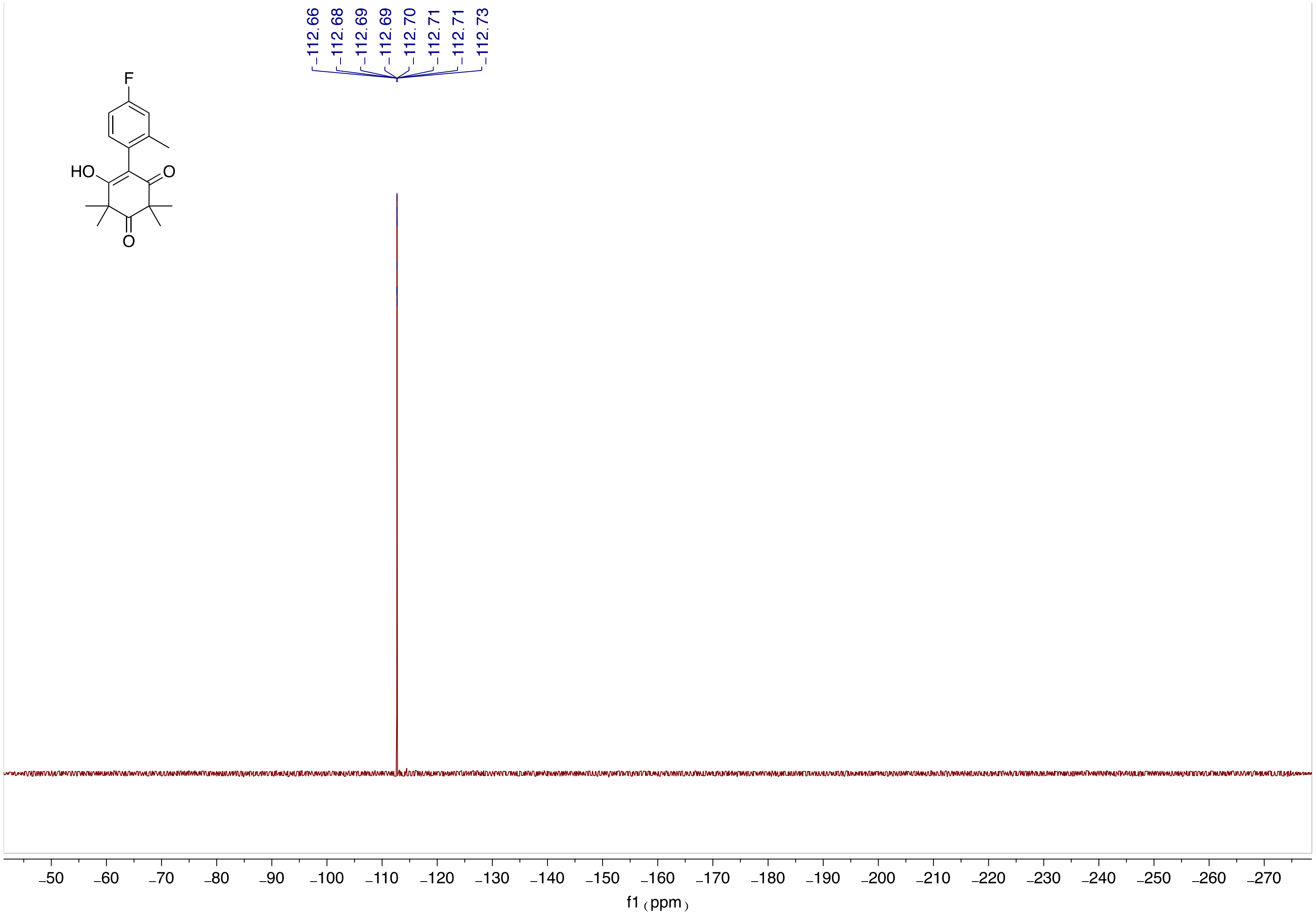

11 - ^{1}H NMR (400 MHz, CD_3OD):

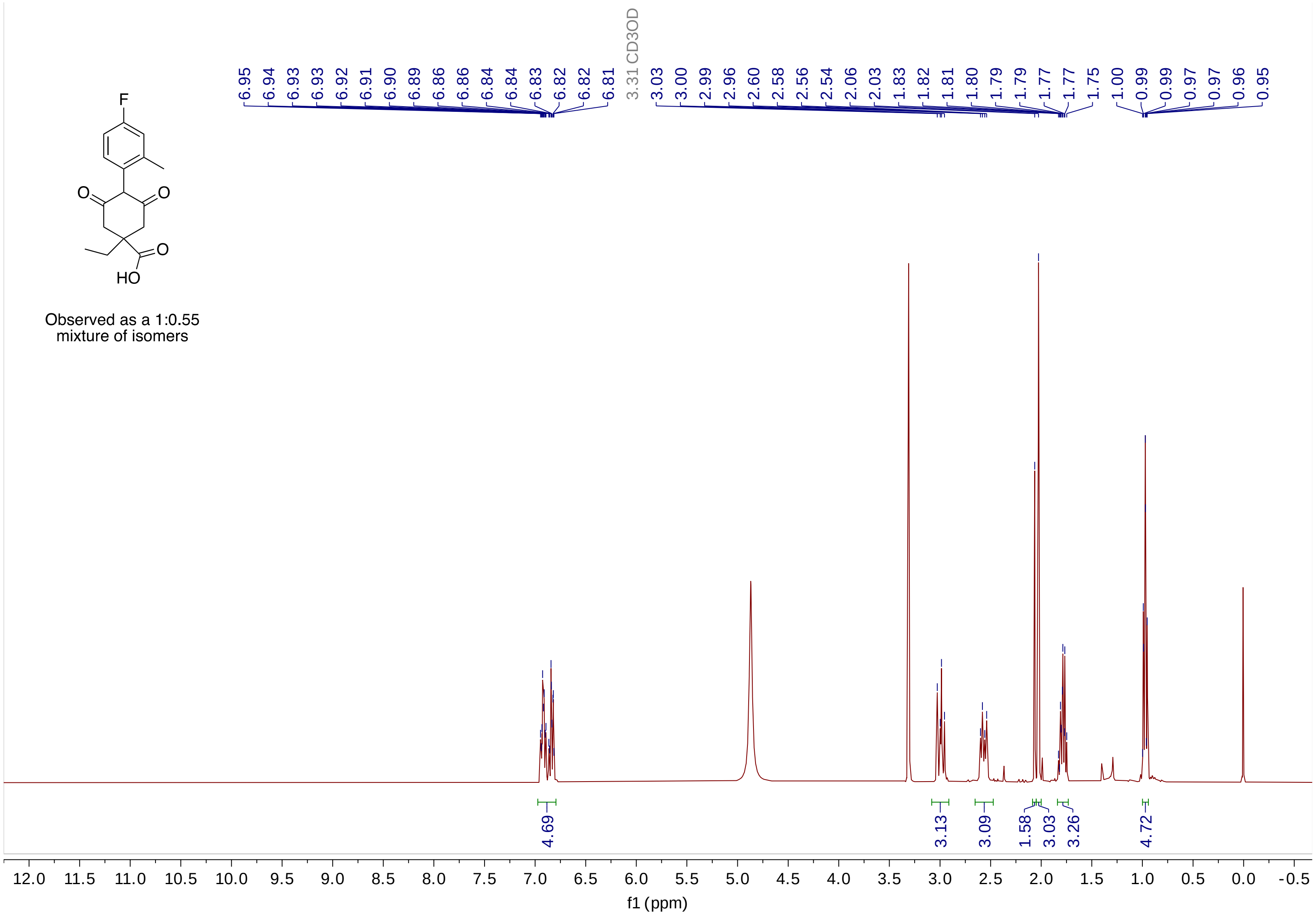

11 - $^{13}C\{^{1}H\}$ NMR (126 MHz, CD_3OD):

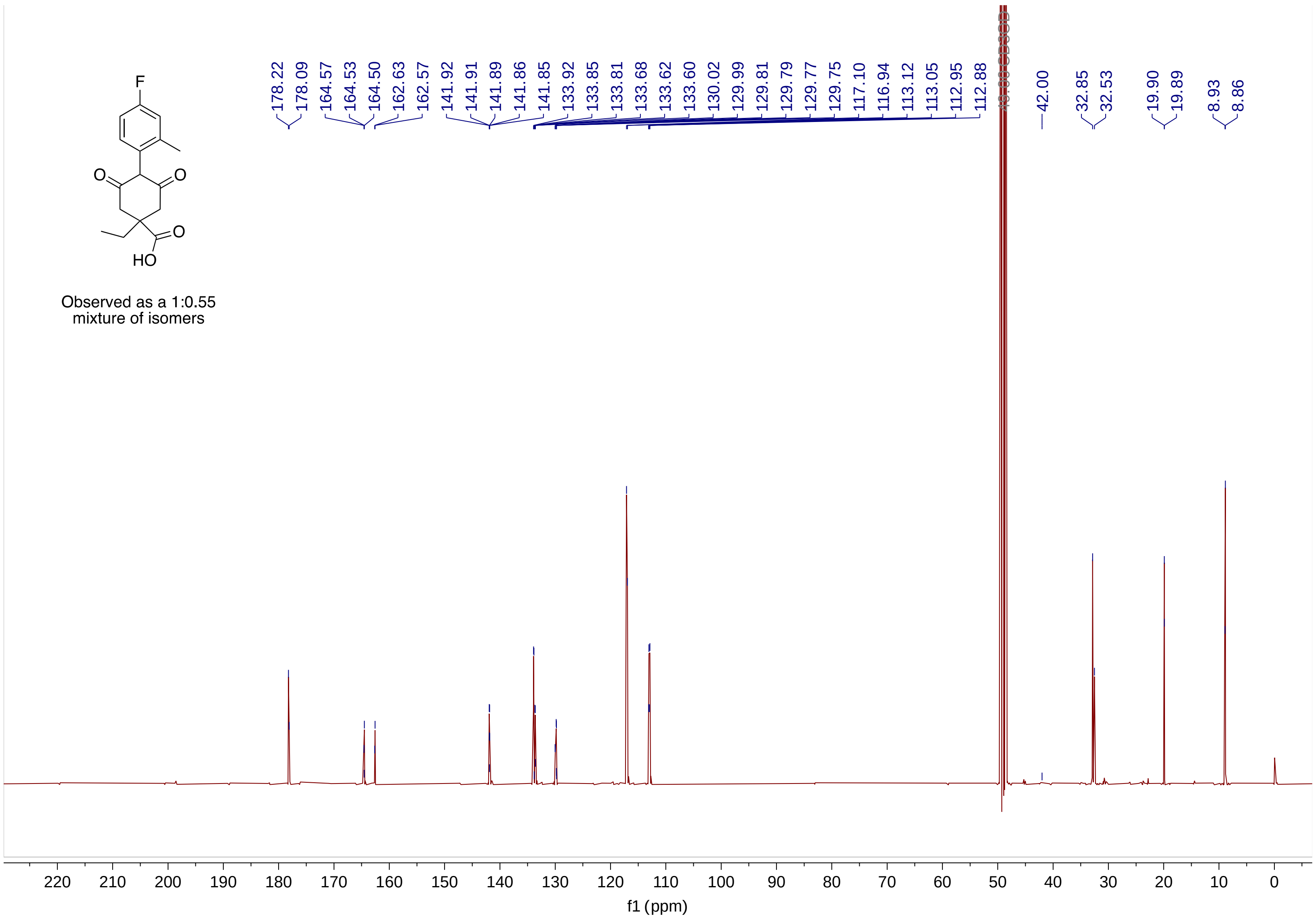

11 - ^{19}F NMR (376 MHz, CD_3OD):

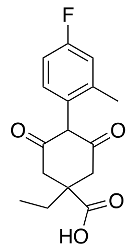

Observed as a 1:0.55 mixture of isomers

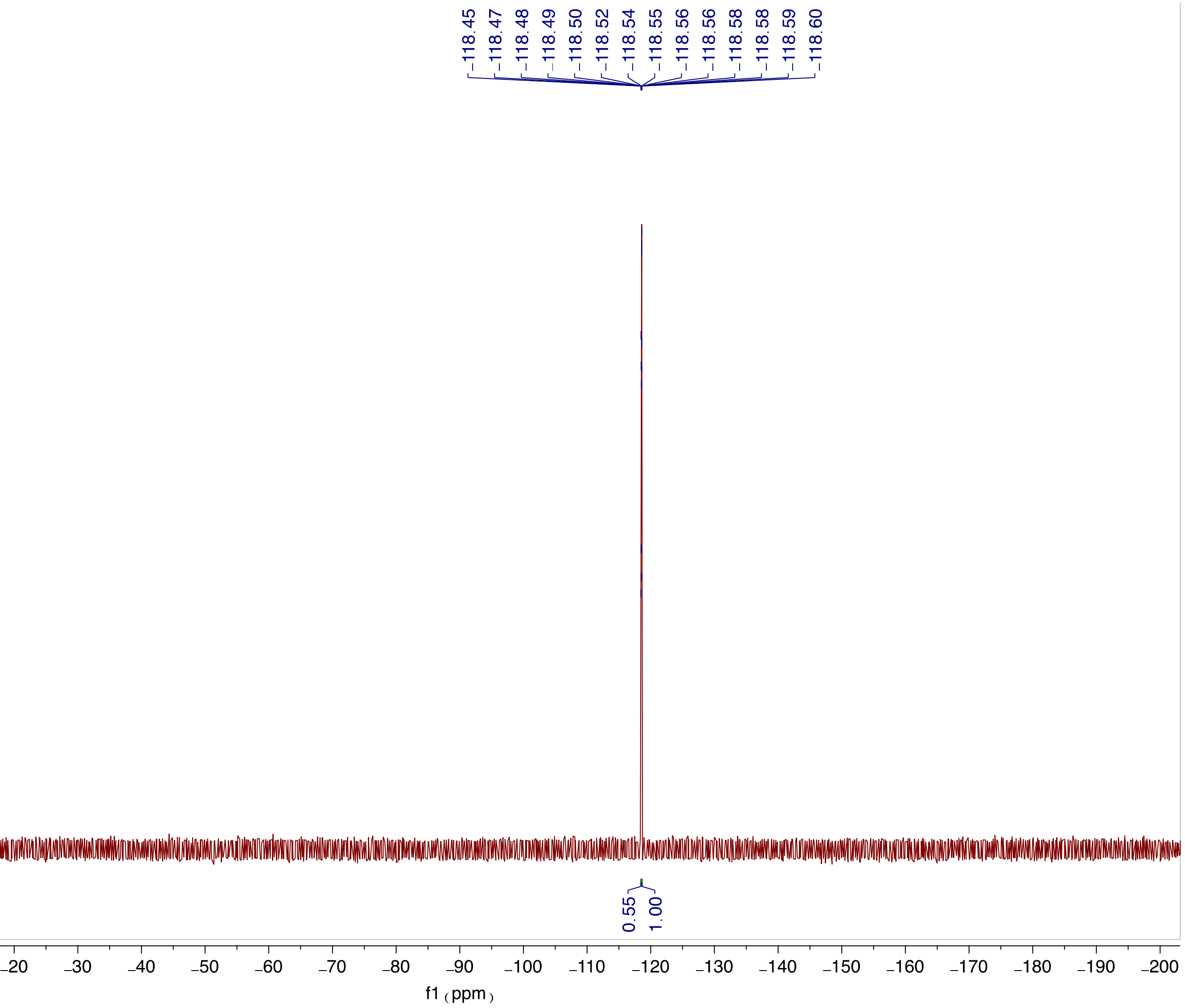

12 - ^{1}H NMR (500 MHz, CD_3OD):

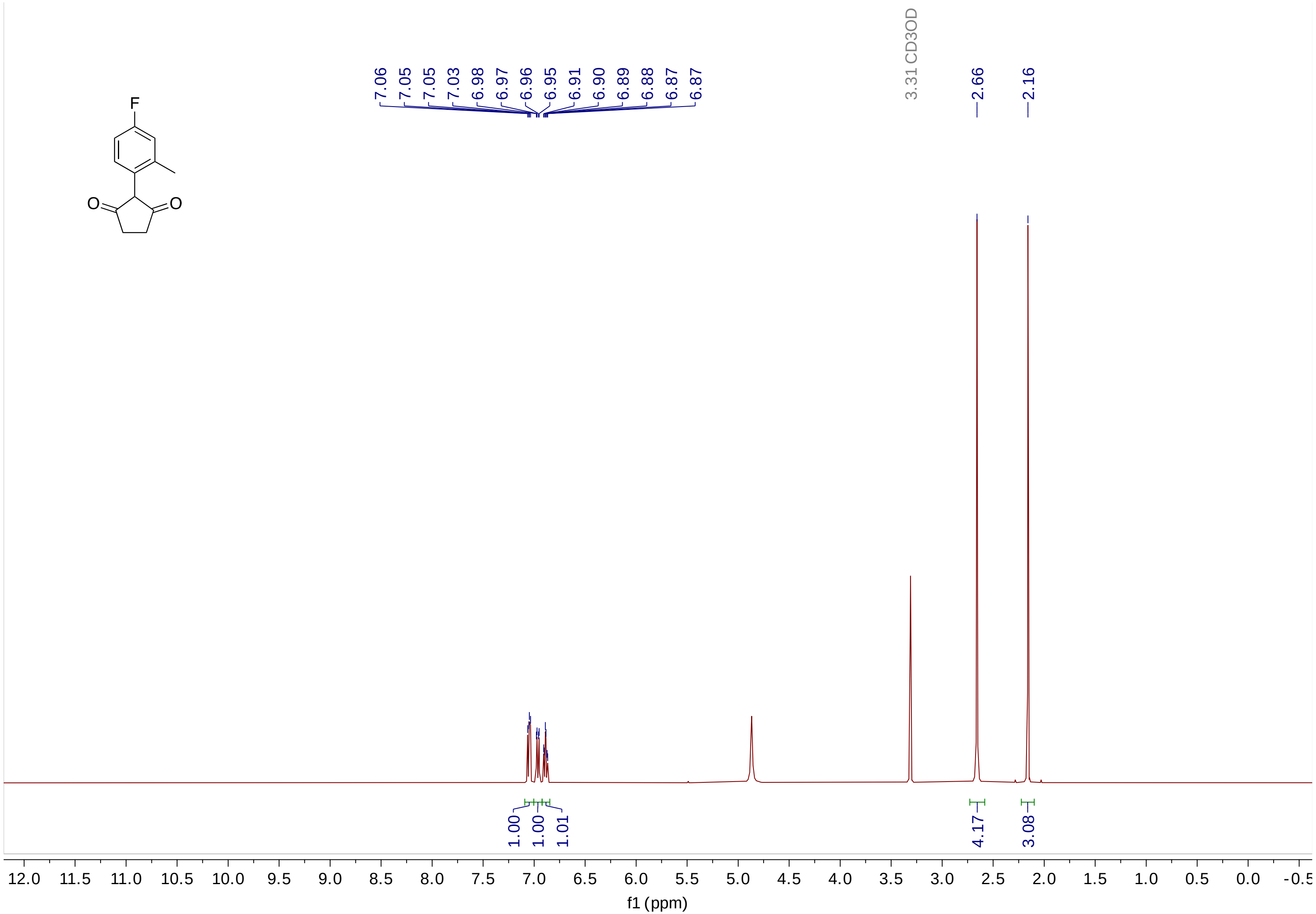

12 - $^{13}C\{^1H\}$ NMR (126 MHz, CD_3OD):

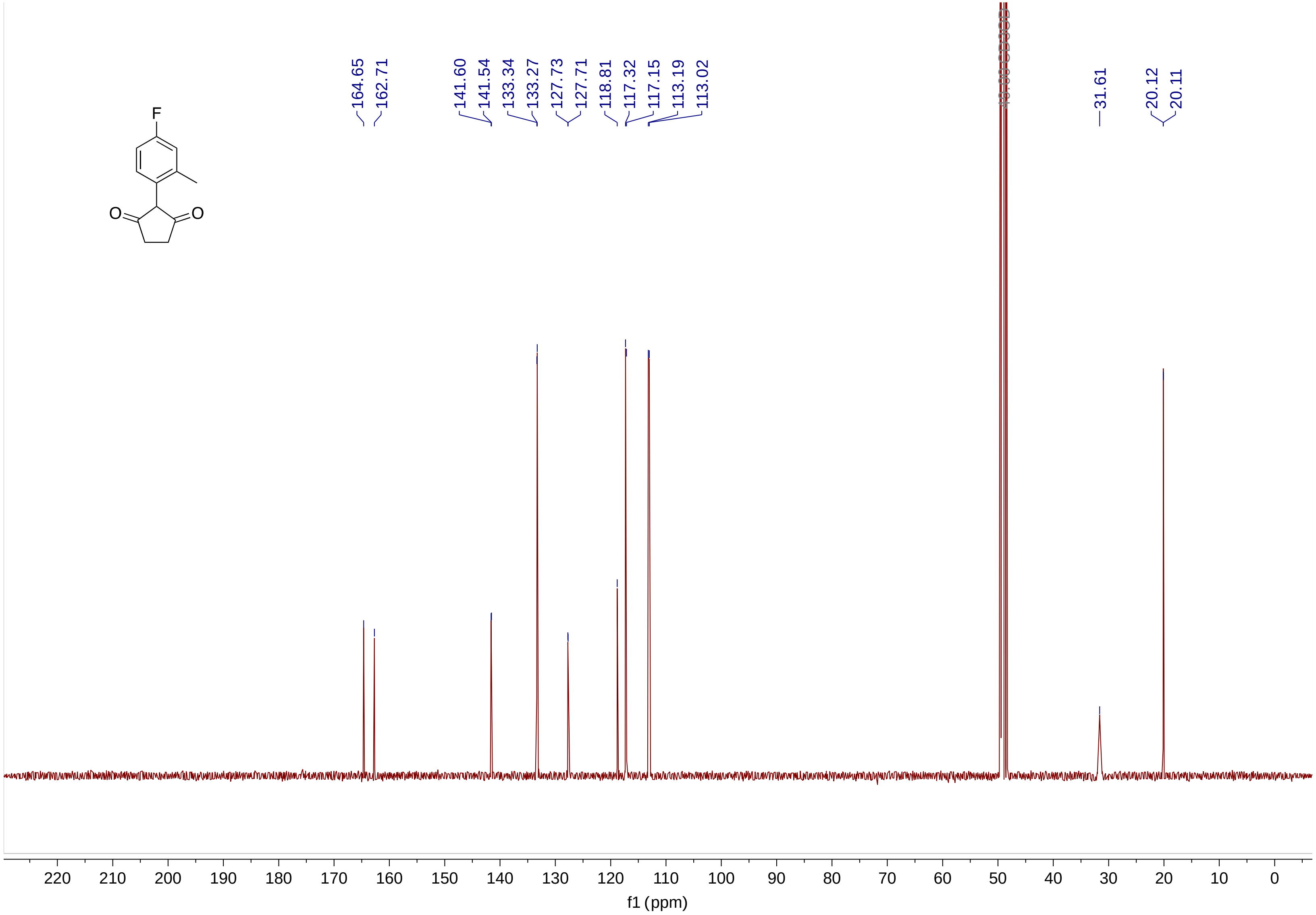

12 - ^{19}F NMR (471 MHz, CD_3OD):

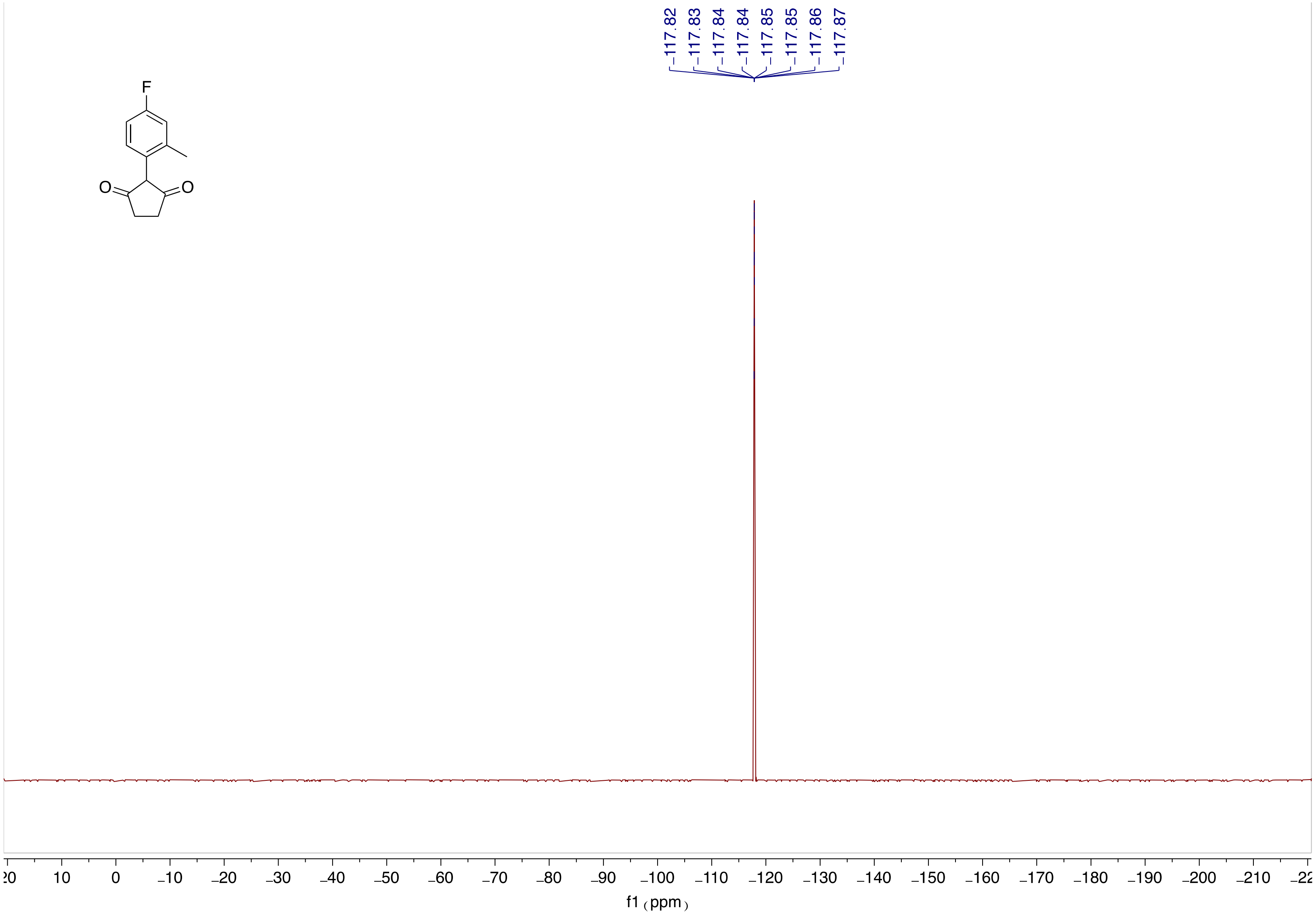

13 - ^{1}H NMR (400 MHz, CD_3OD):

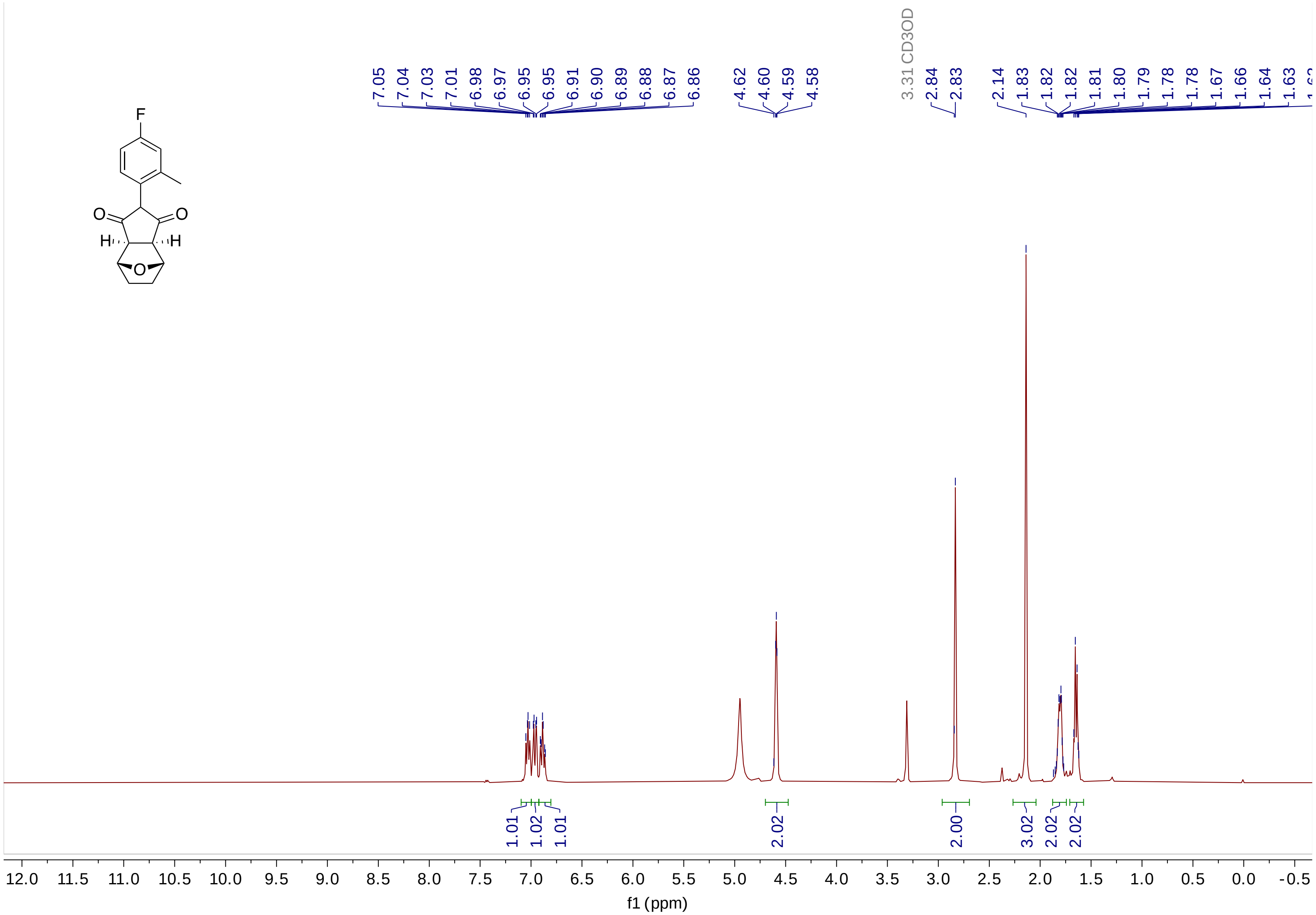

13 - $^{13}C\{^1H\}$ NMR (101 MHz, CD_3OD):

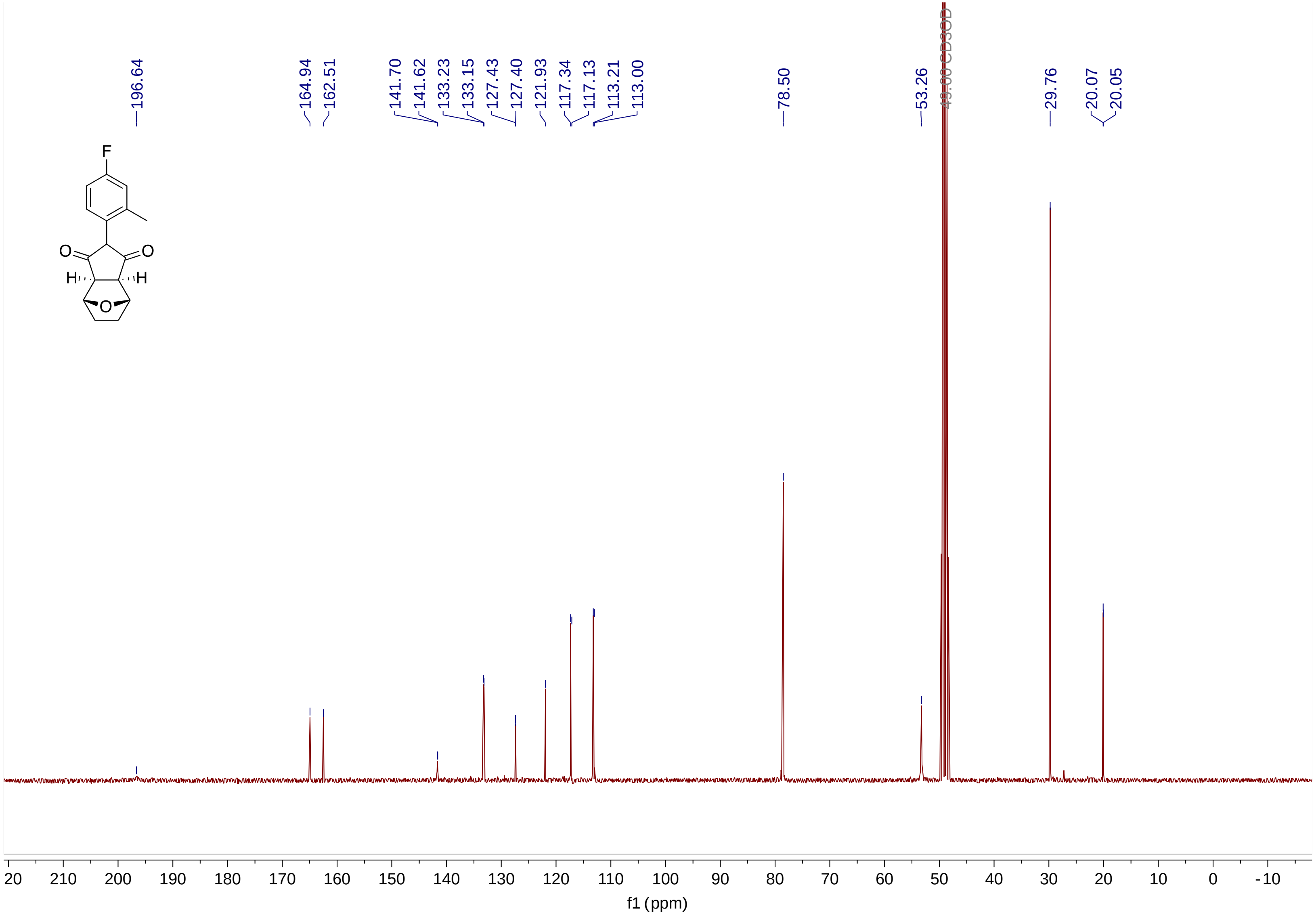

13 - ^{19}F NMR (376 MHz, CD_3OD):

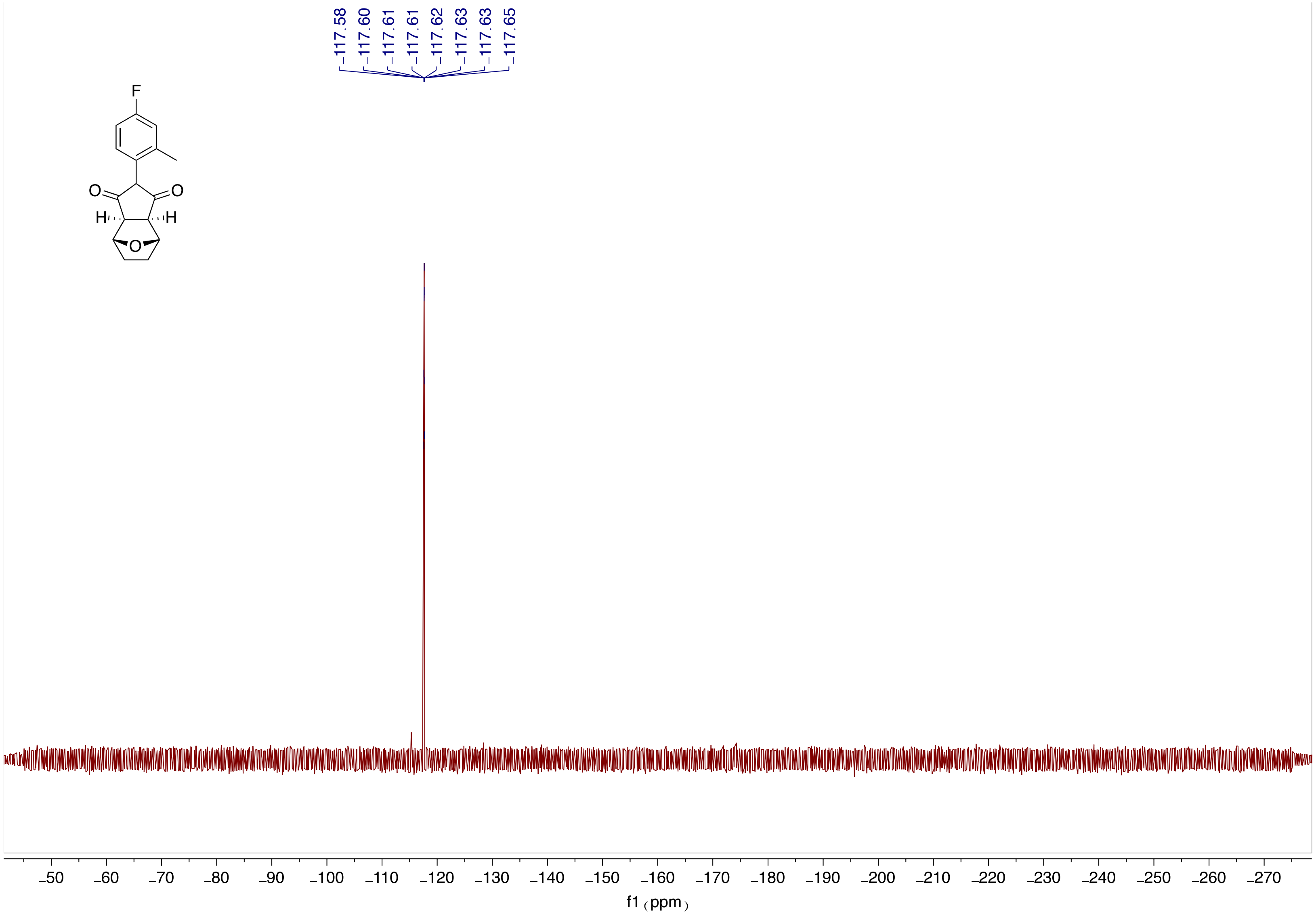

14 - ^{1}H NMR (400 MHz, $CDCl_3$):

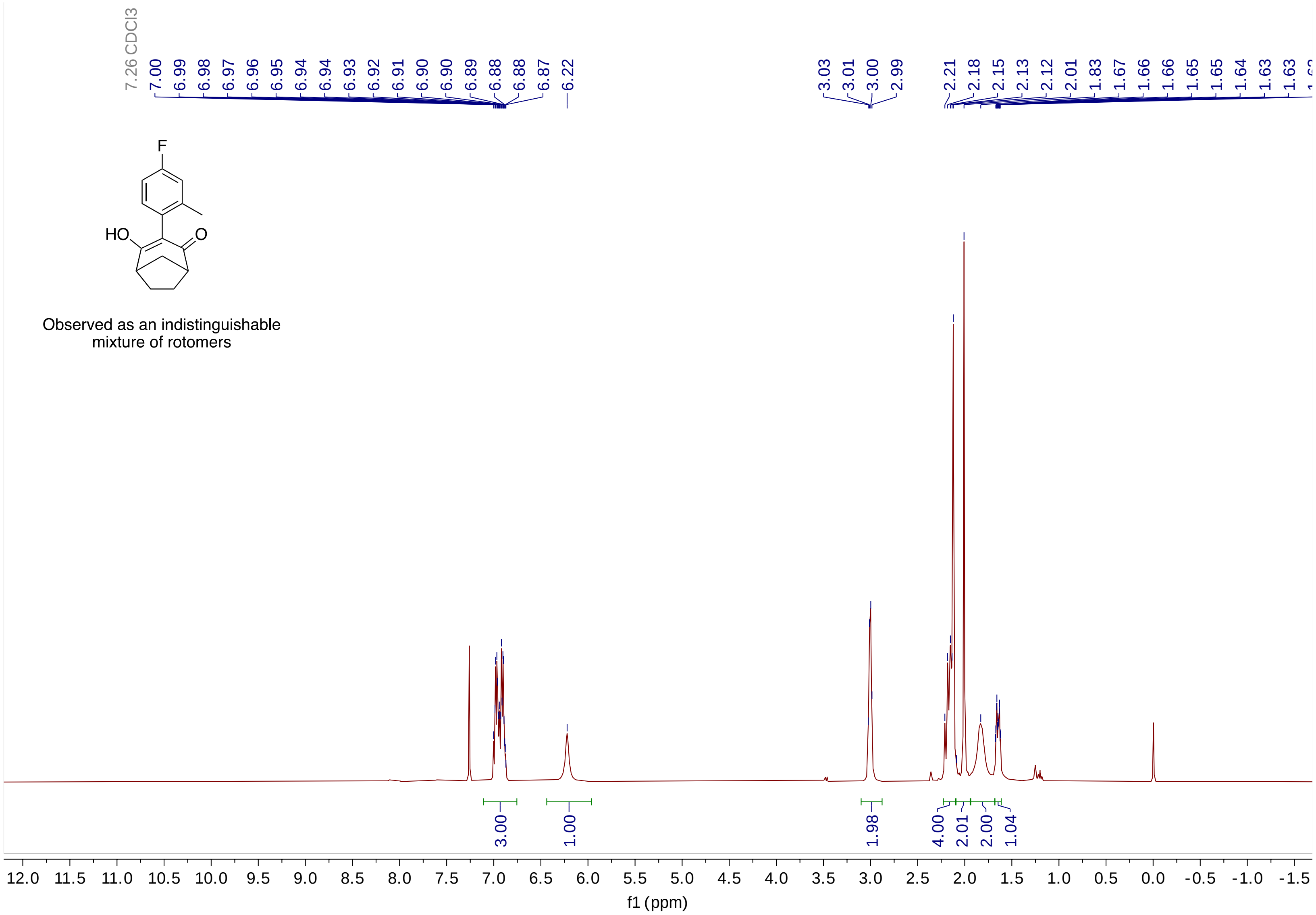

14 - $^{13}C\{^1H\}$ NMR (101 MHz, $CDCl_3$):

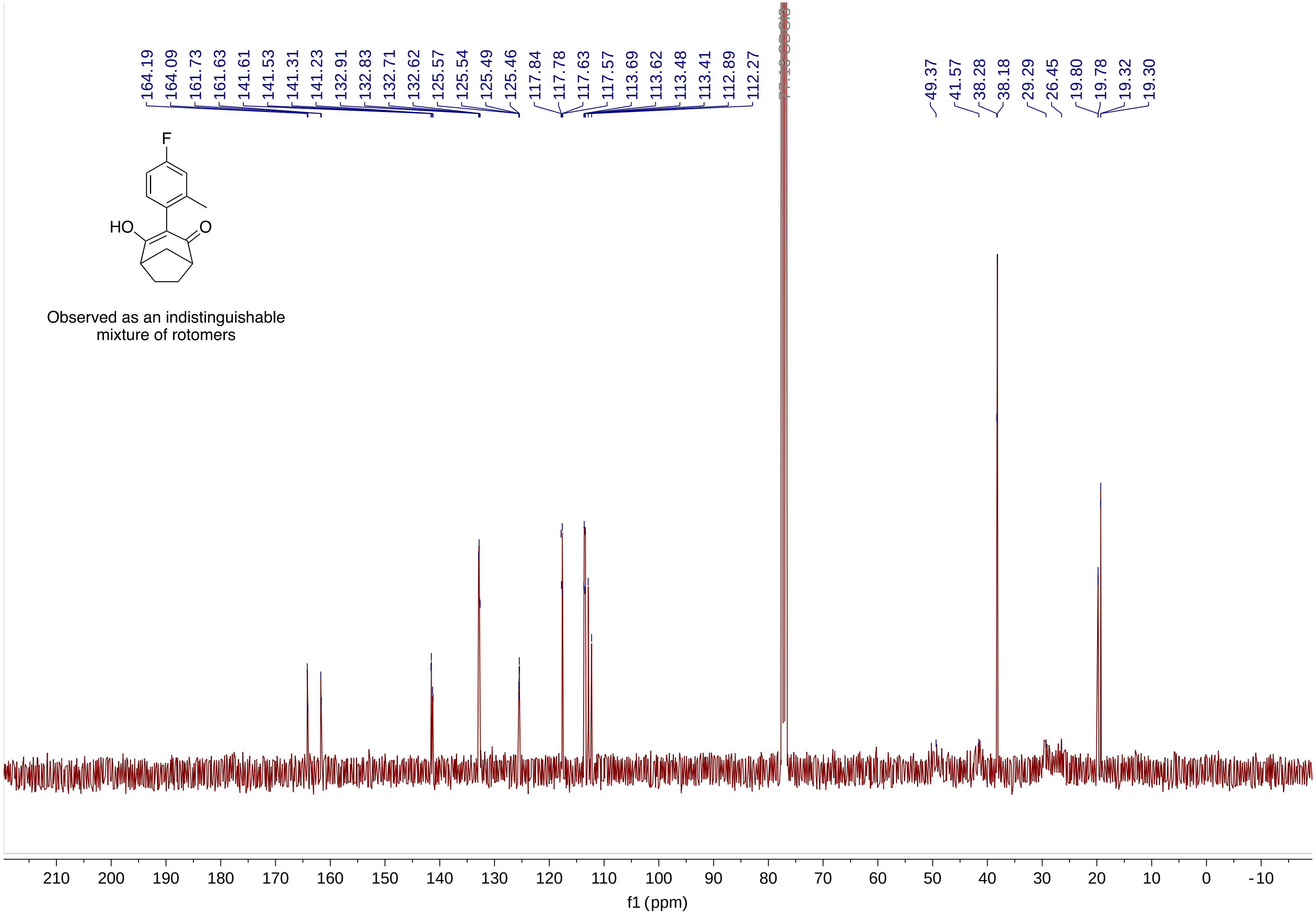

14 - ^{19}F NMR (376 MHz, $CDCl_3$):

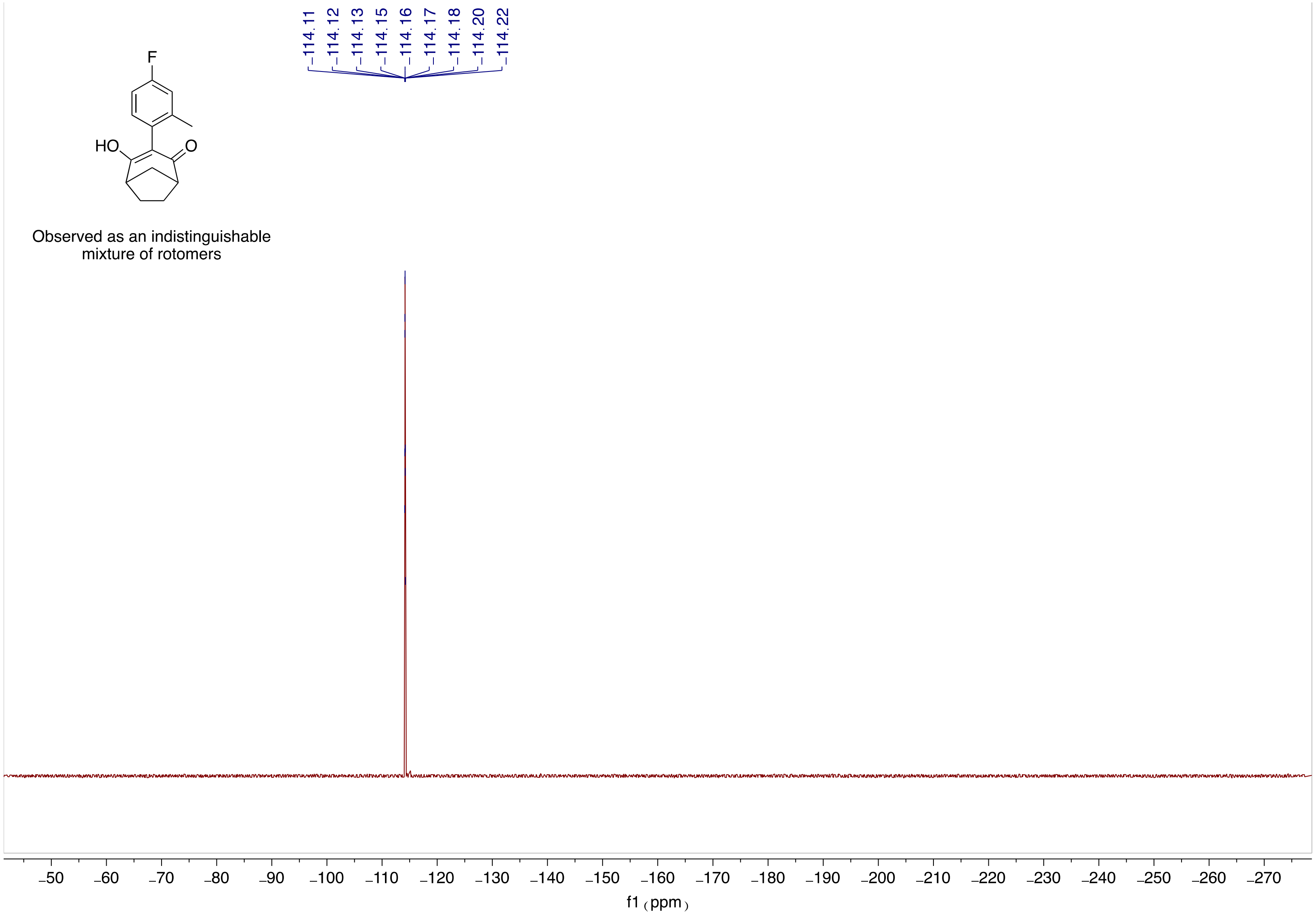

15 - ¹H NMR (500 MHz, CDCl₃):

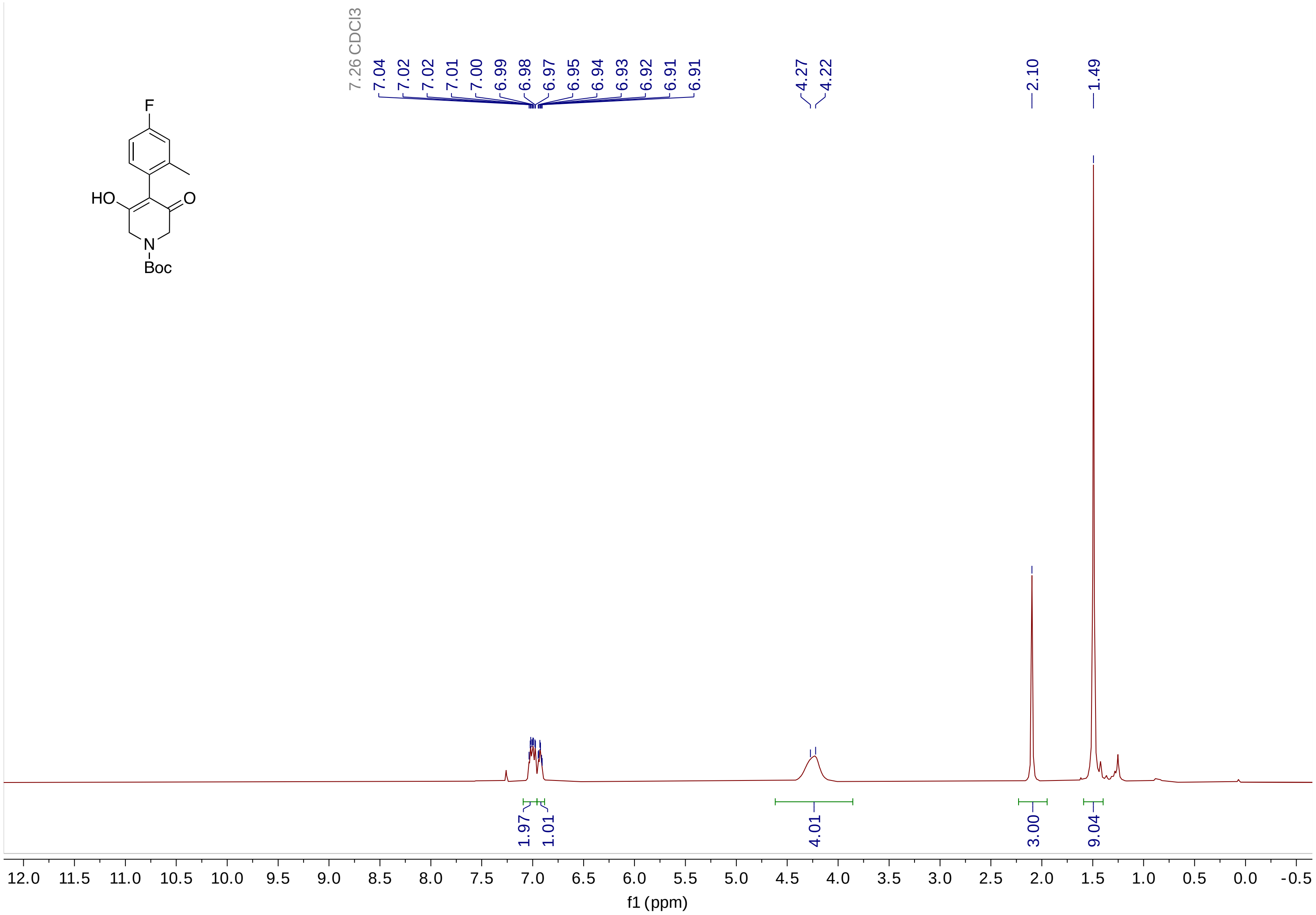

15 - $^{13}C\{^{1}H\}$ NMR (126 MHz, $CDCl_3$):

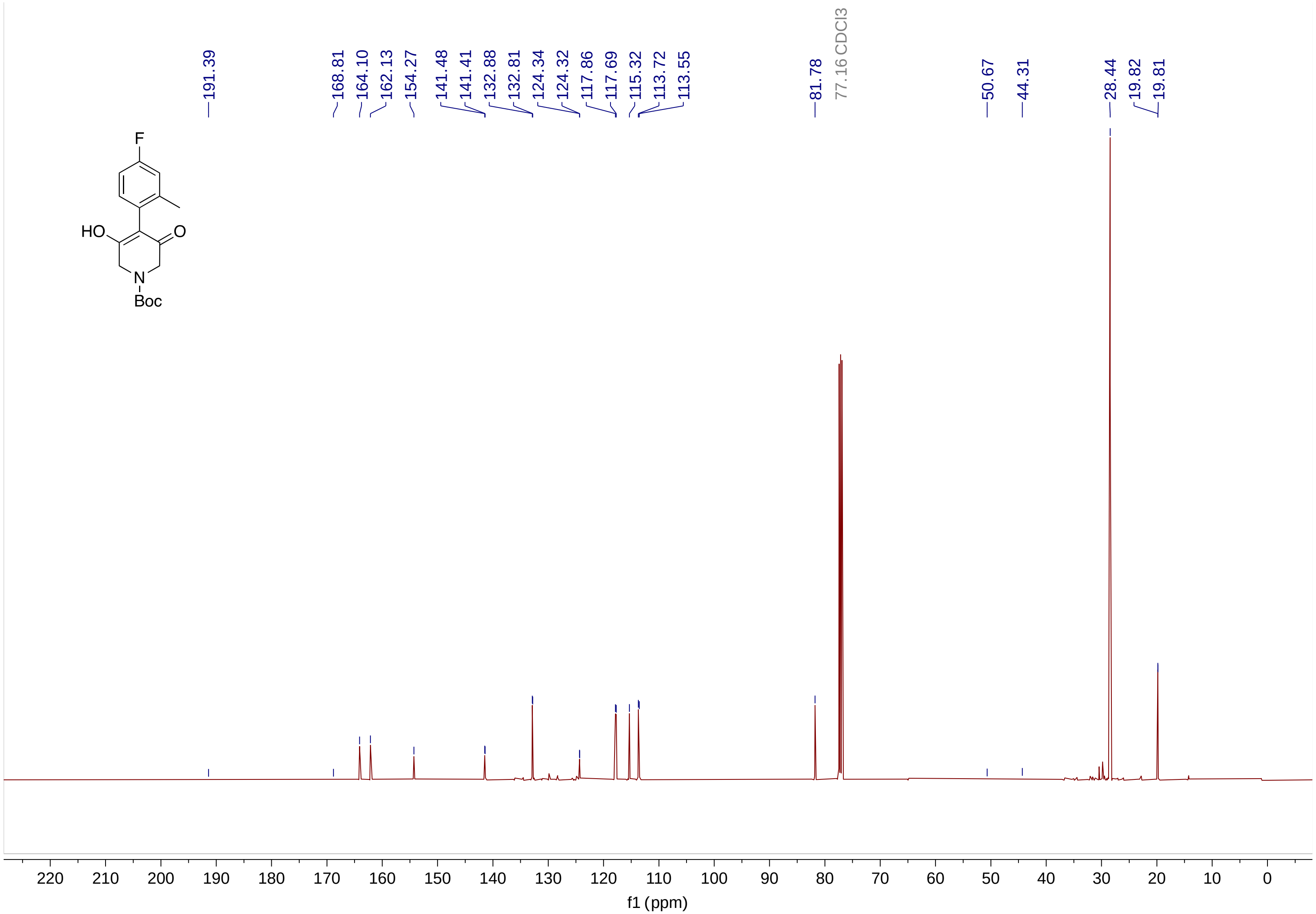

15 - ^{19}F NMR (471 MHz, $CDCl_3$):

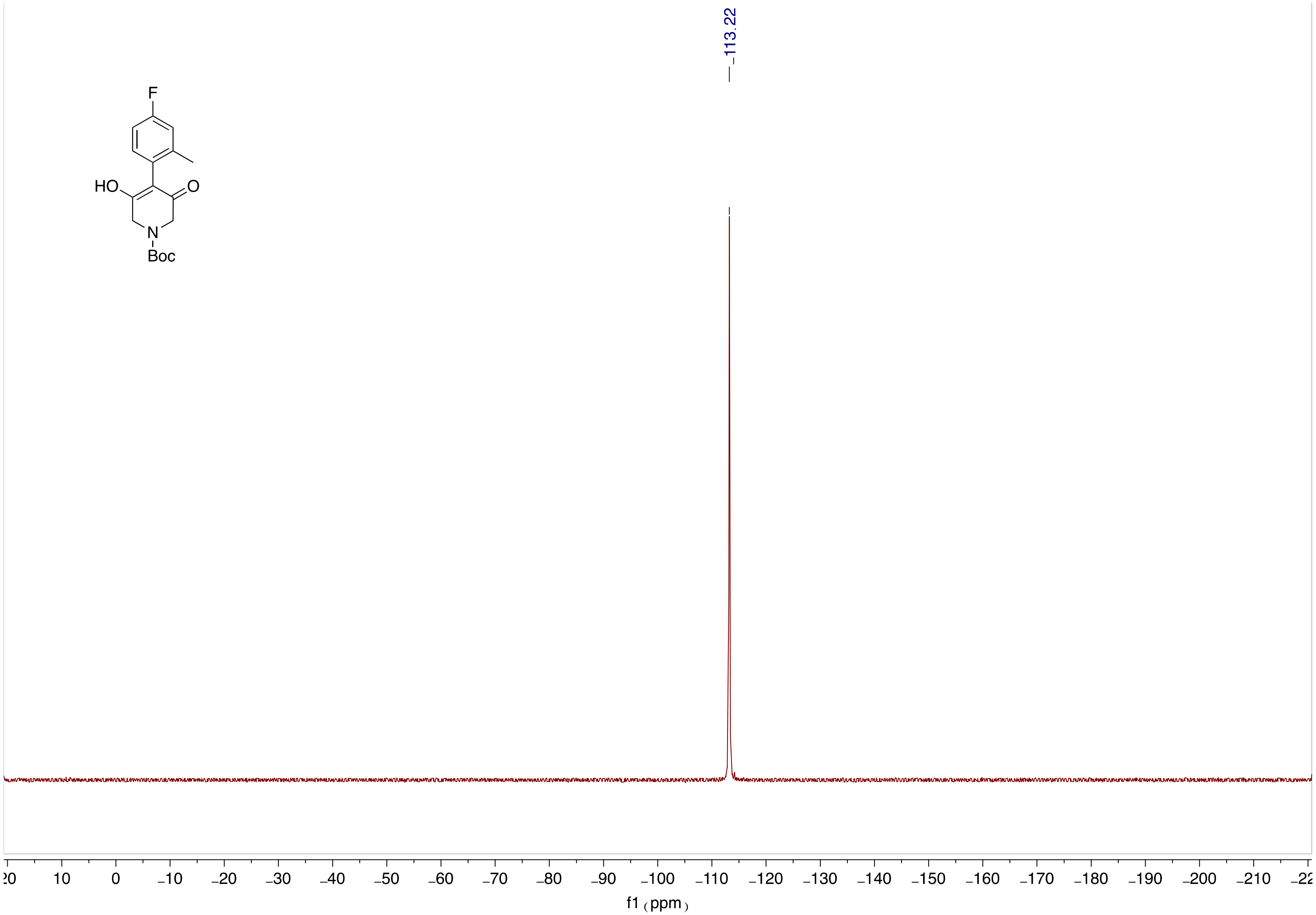

16 - ^{1}H NMR (500 MHz, $CDCl_3$):

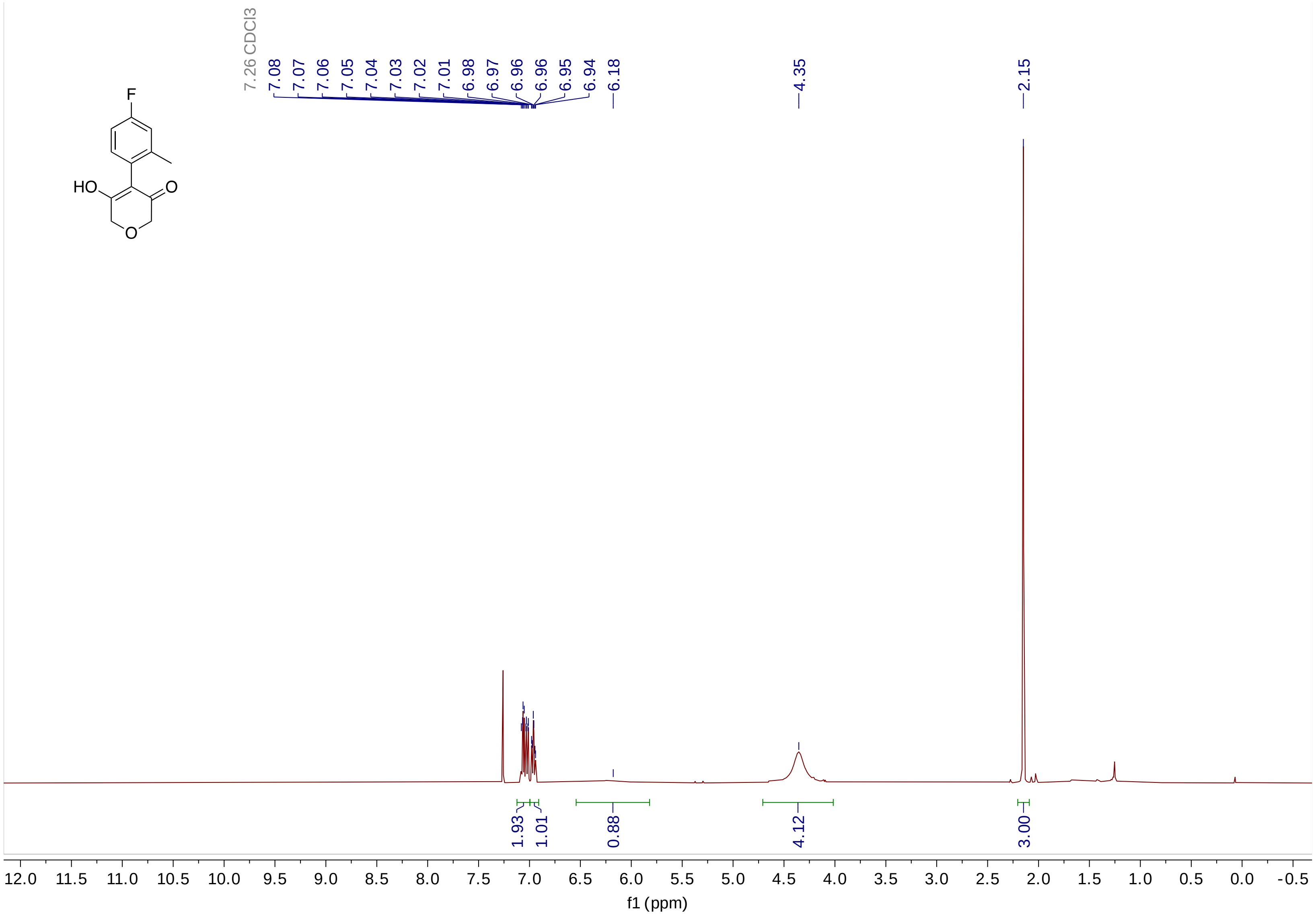

16 - ^{13}C{^{1}H} NMR (126 MHz, CDCl$_3$):

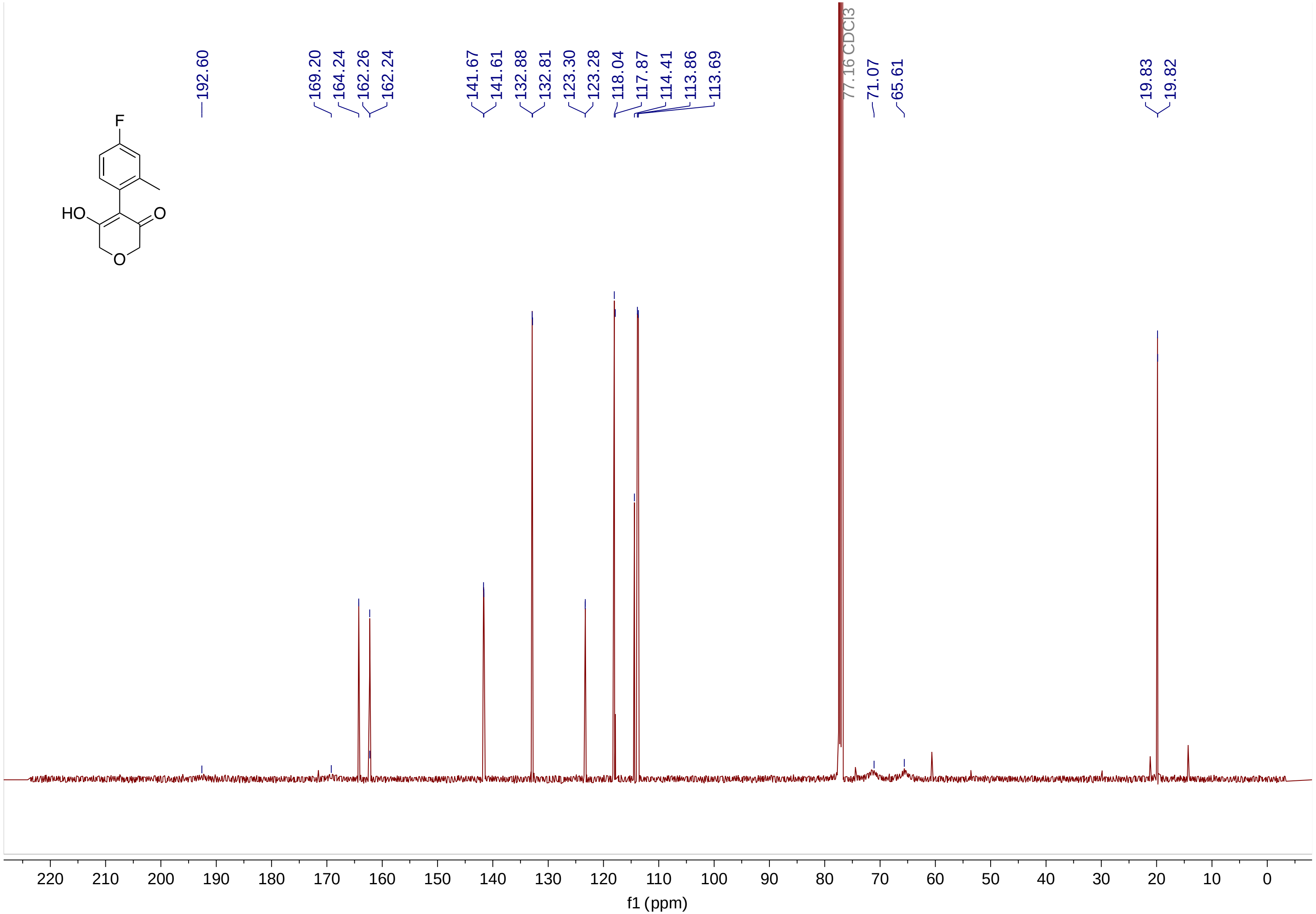

16 - ^{19}F NMR (471 MHz, $CDCl_3$):

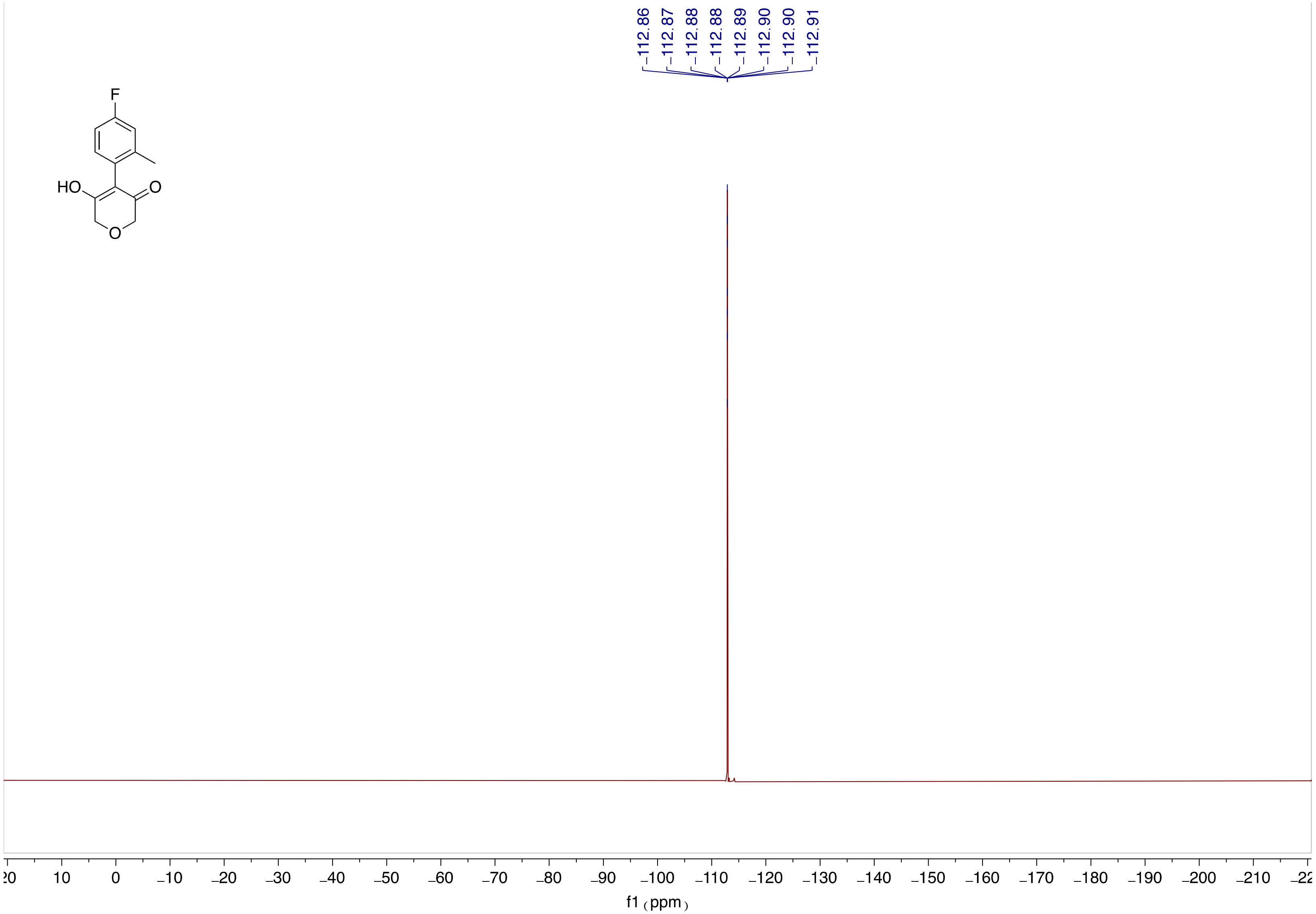

17 - ^{1}H NMR (500 MHz, $CDCl_3$):

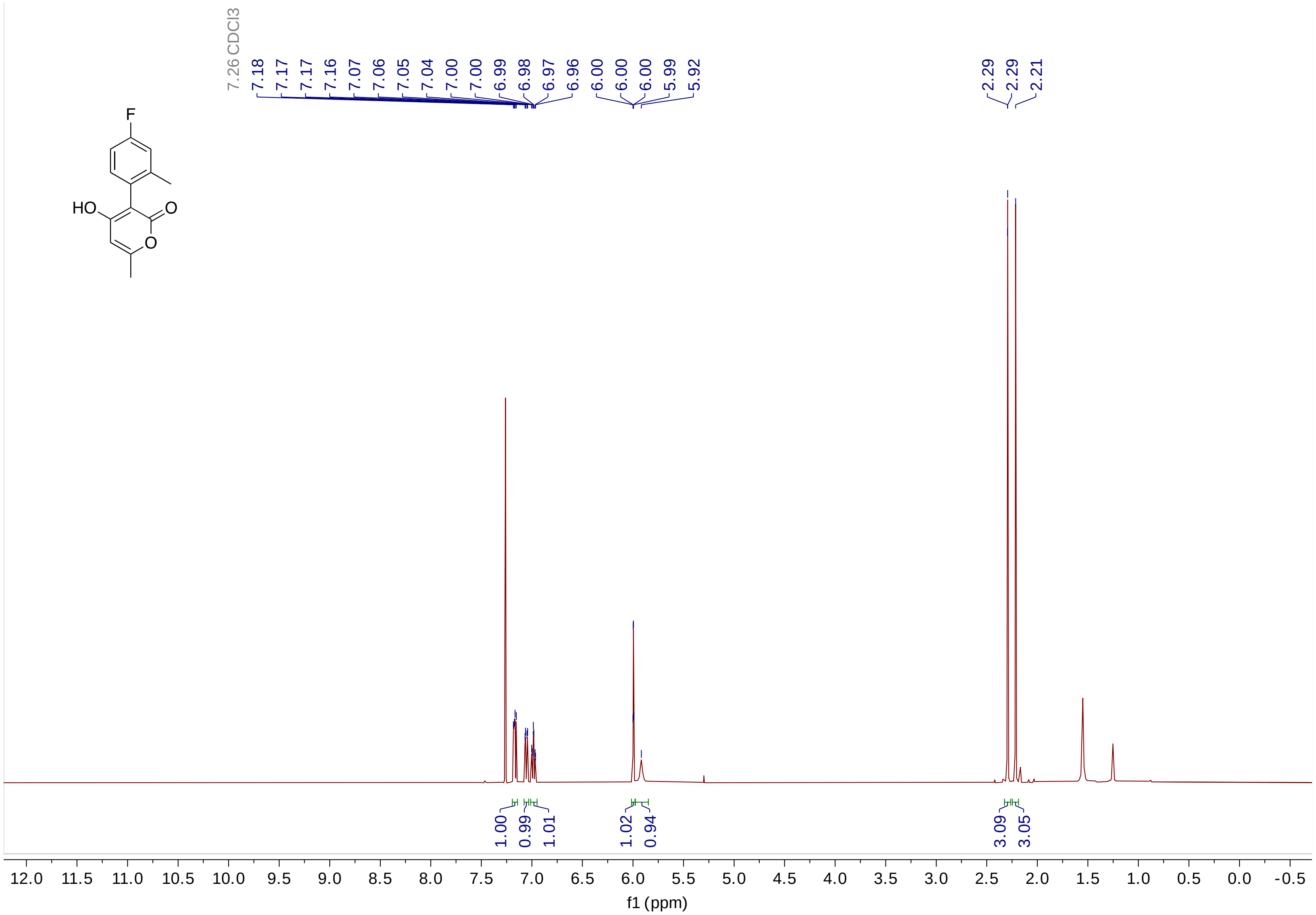

17 - $^{13}C\{^1H\}$ NMR (126 MHz, $CDCl_3$):

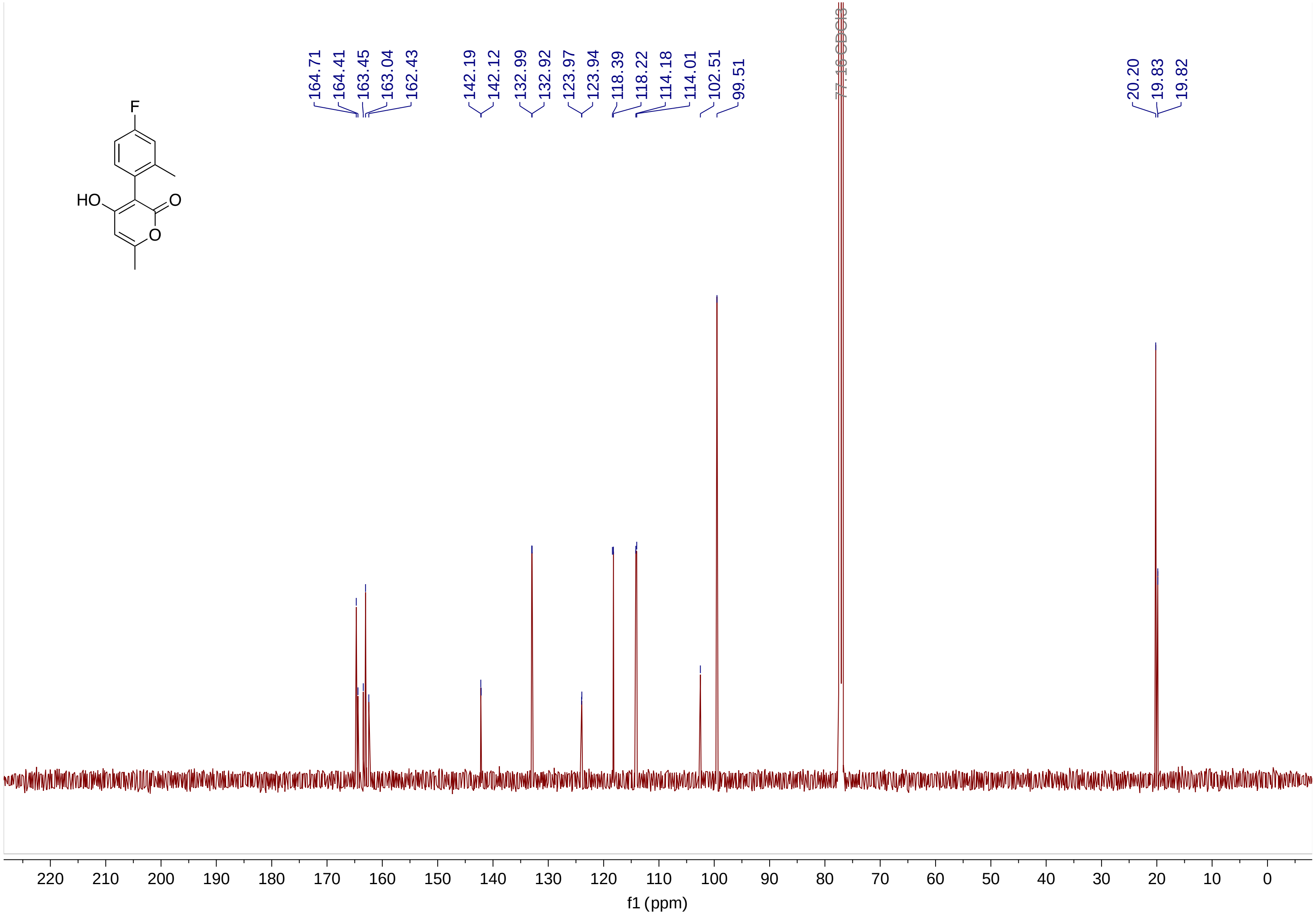

17 - ^{19}F NMR (471 MHz, $CDCl_3$):

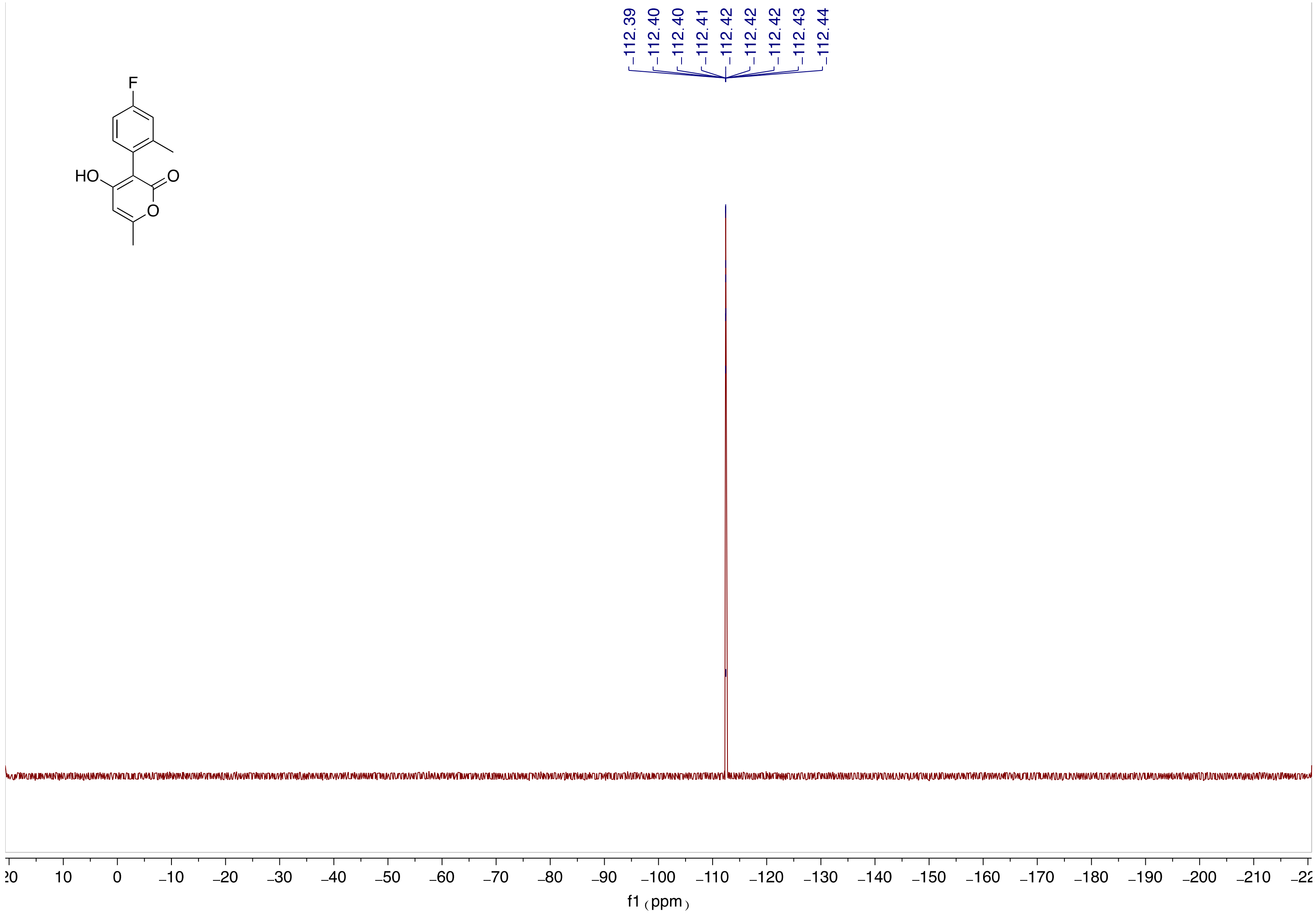

18 - ^{1}H NMR (500 MHz, CDCl3):

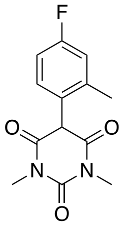

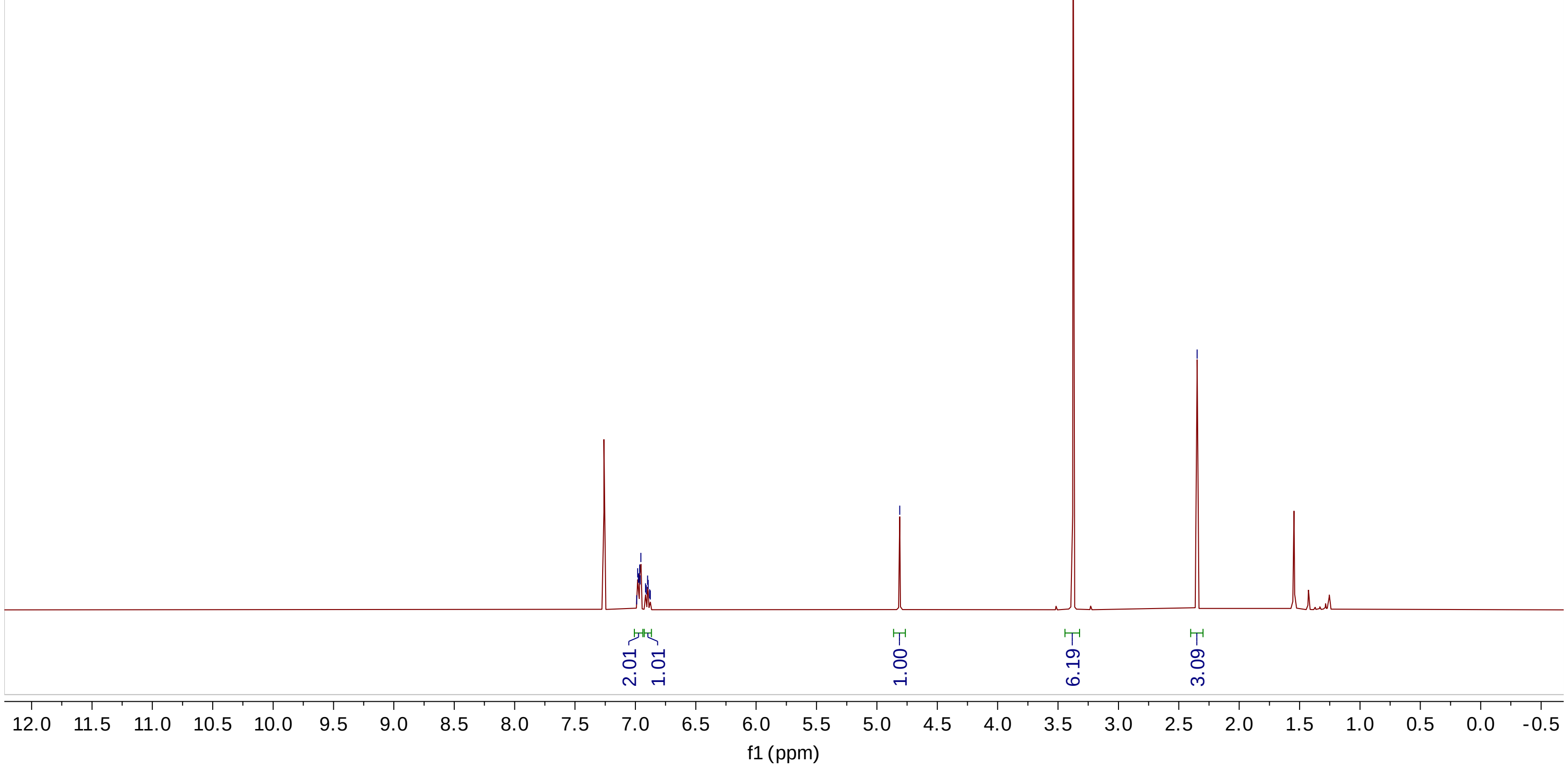

18 - $^{13}C\{^1H\}$ NMR (126 MHz, $CDCl_3$):

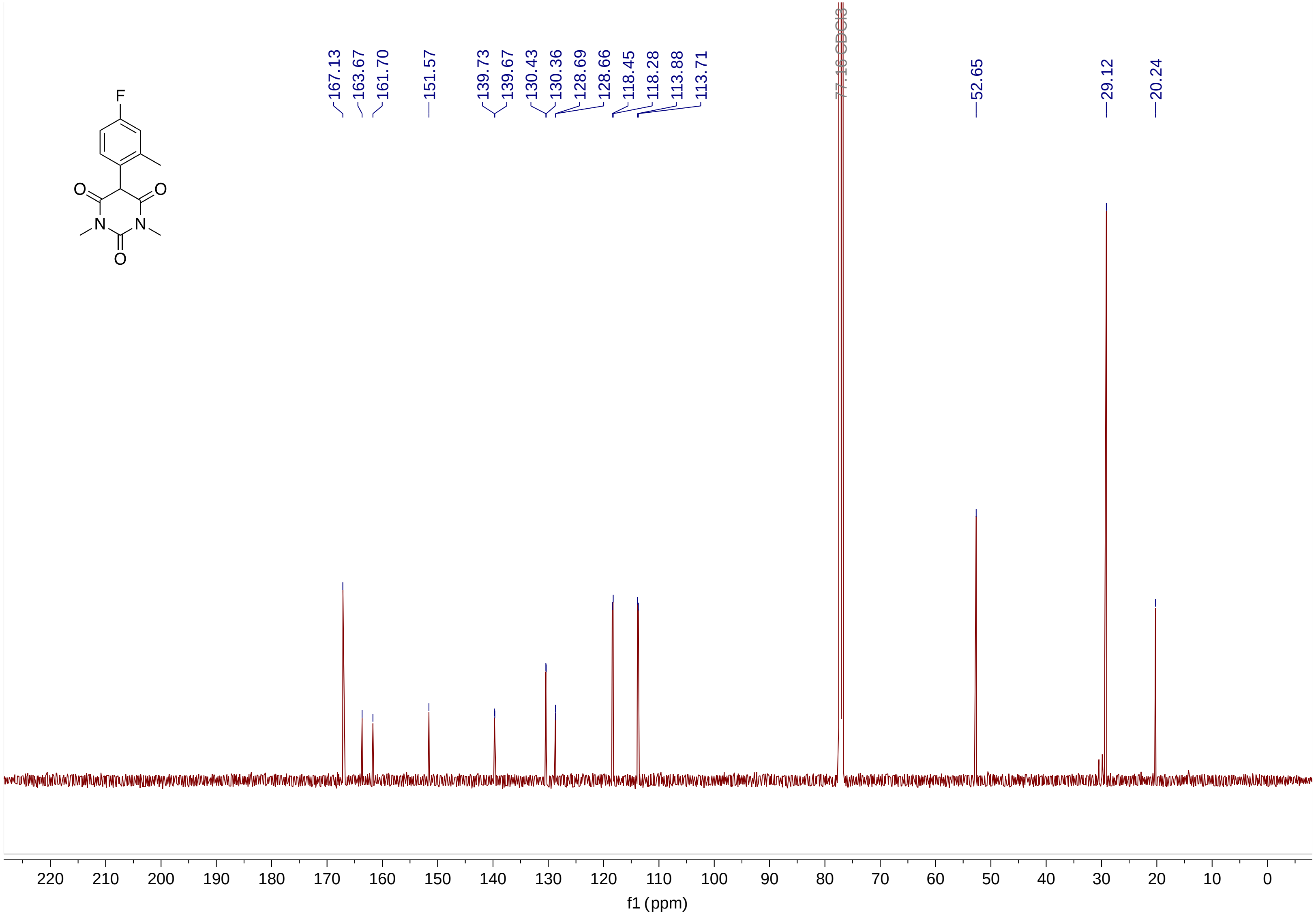

18 - ^{19}F NMR (471 MHz, $CDCl_3$):

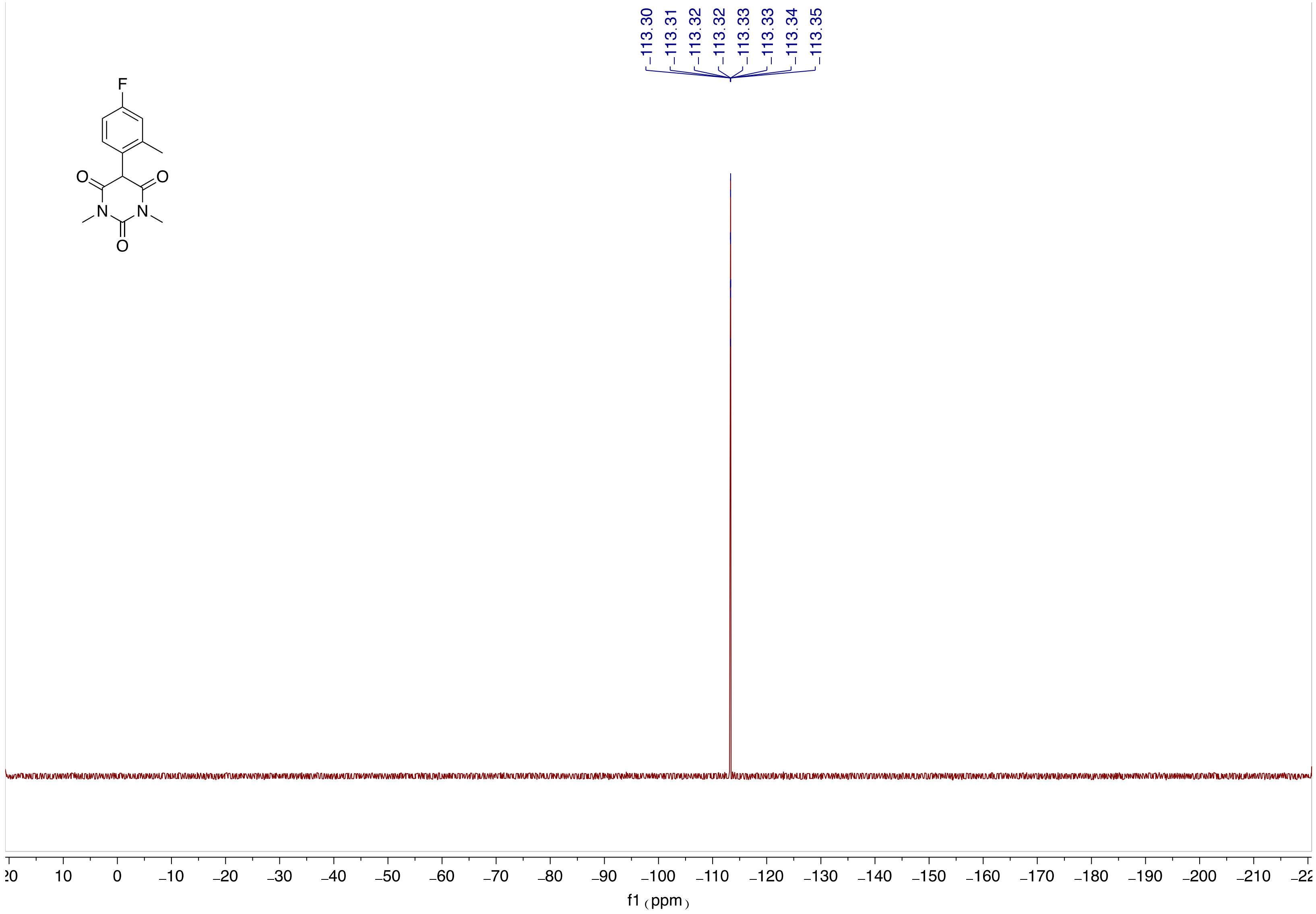

19 - ^{1}H NMR (400 MHz, $CDCl_3$):

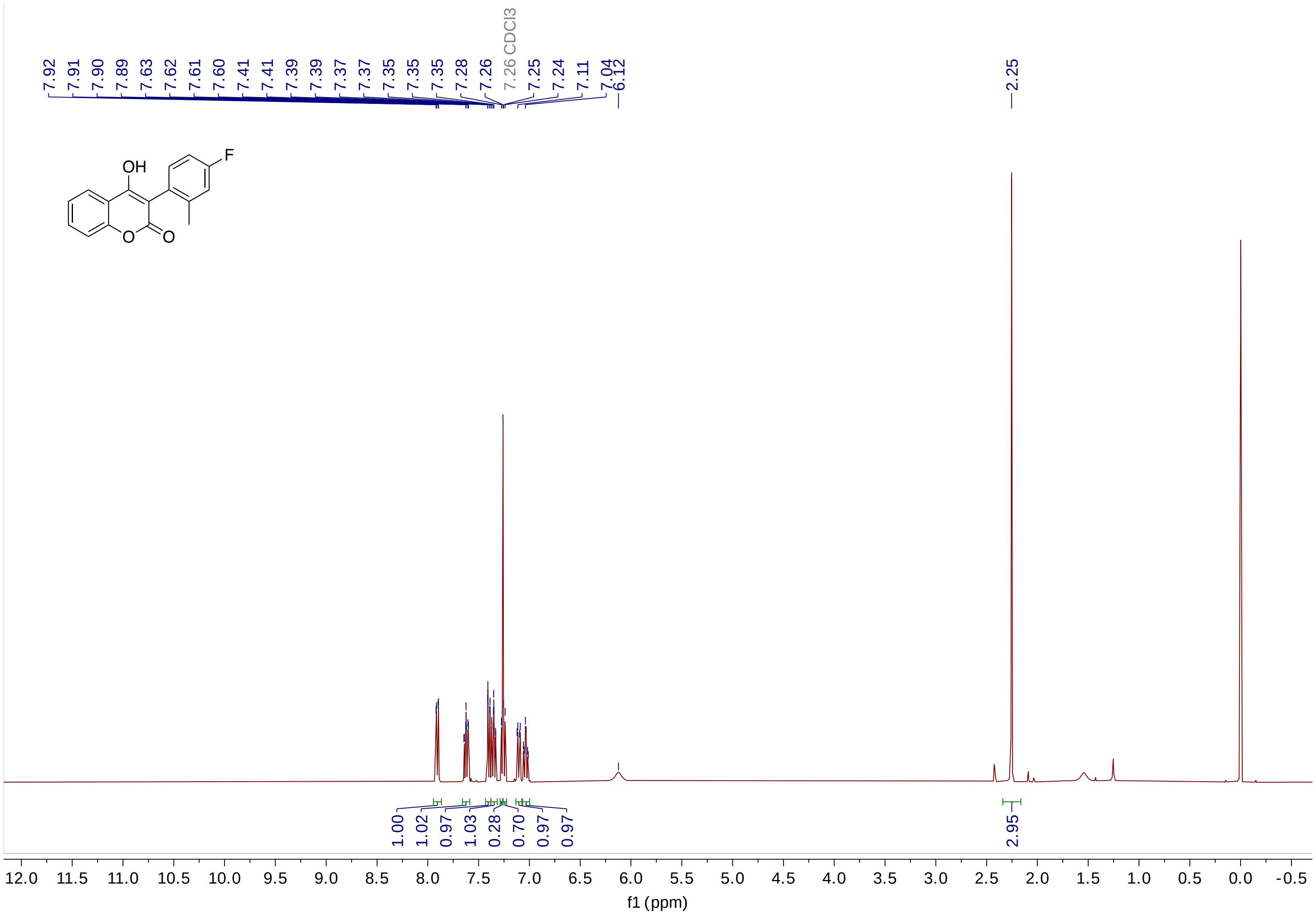

19 - $^{13}C\{^{1}H\}$ NMR (101 MHz, $CDCl_3$):

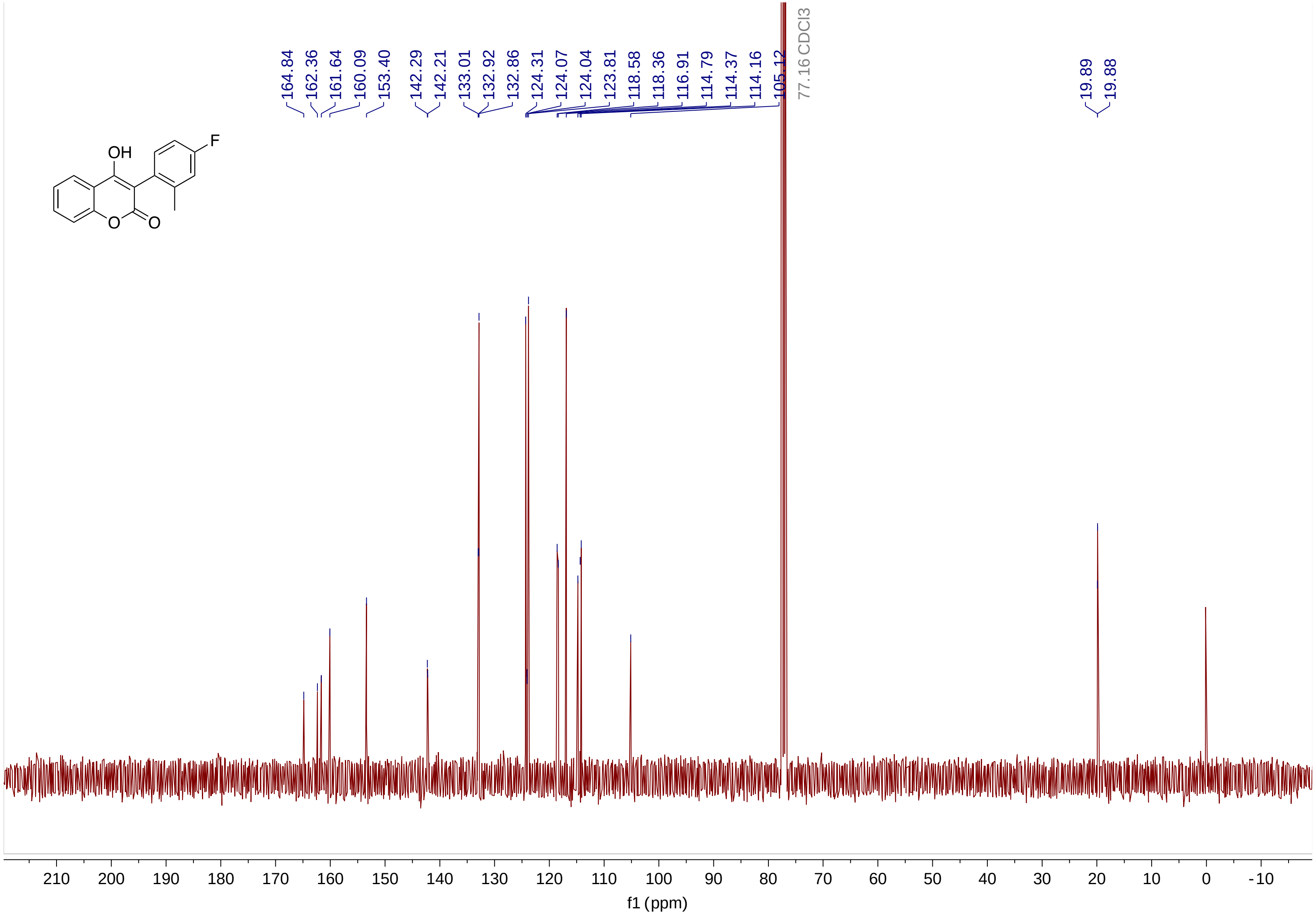

19 - ^{19}F NMR (376 MHz, CDCl$_3$):

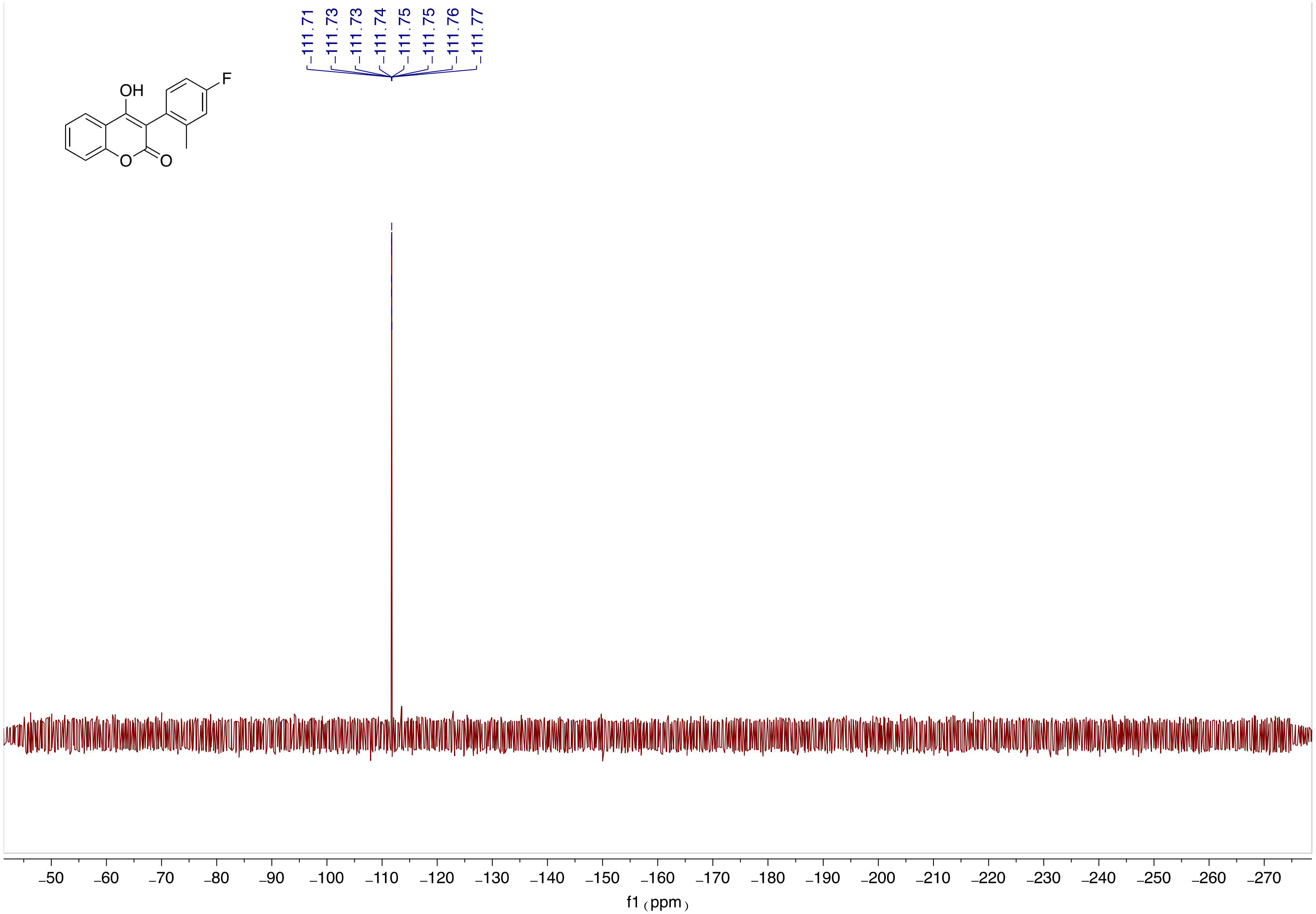

20 - ^{1}H NMR (400 MHz, CD_3OD):

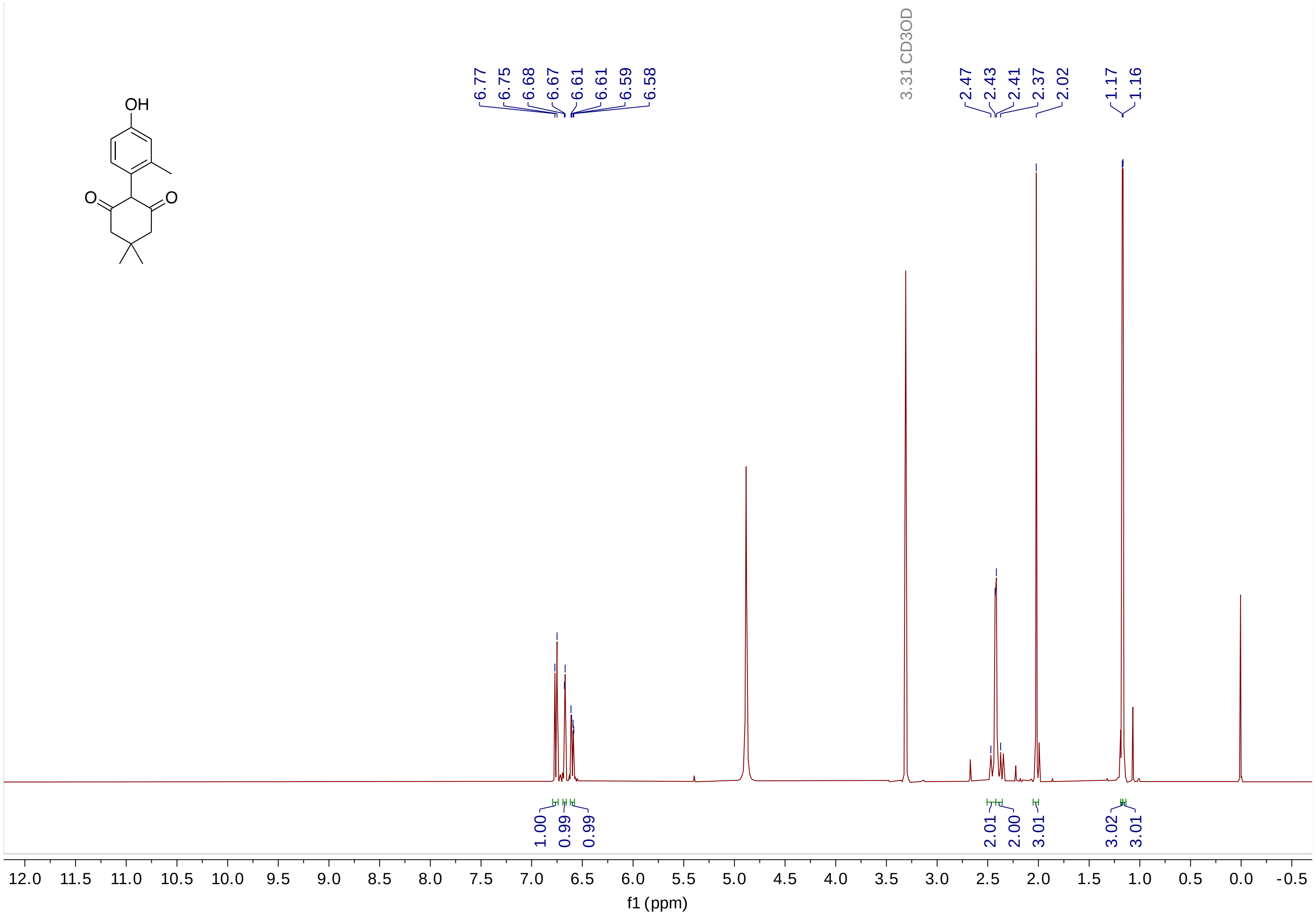

20 - $^{13}C\{^{1}H\}$ NMR (101 MHz, CD_3OD):

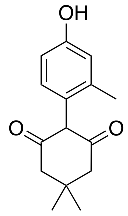

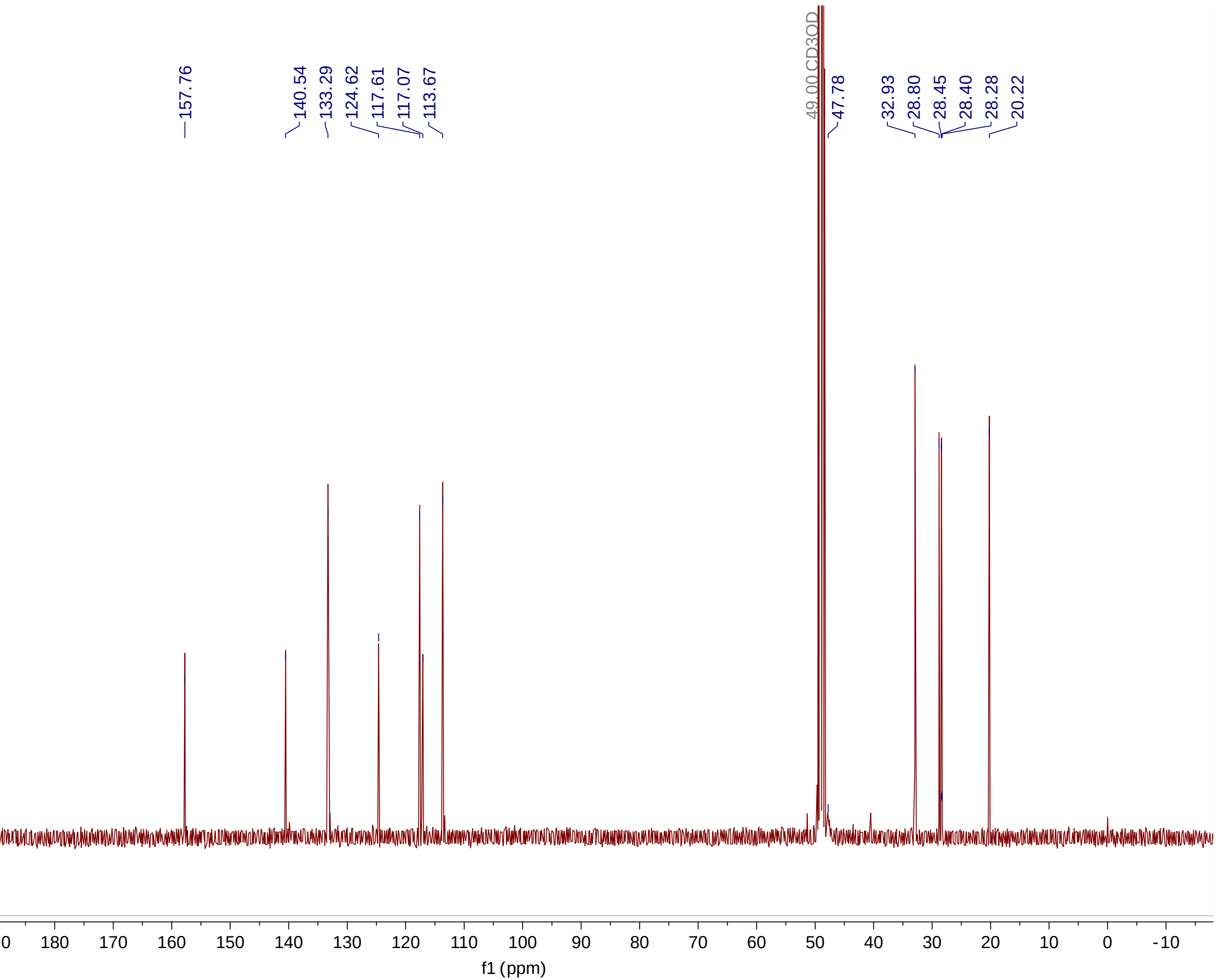

21 - ^{1}H NMR (400 MHz, $CDCl_3$):

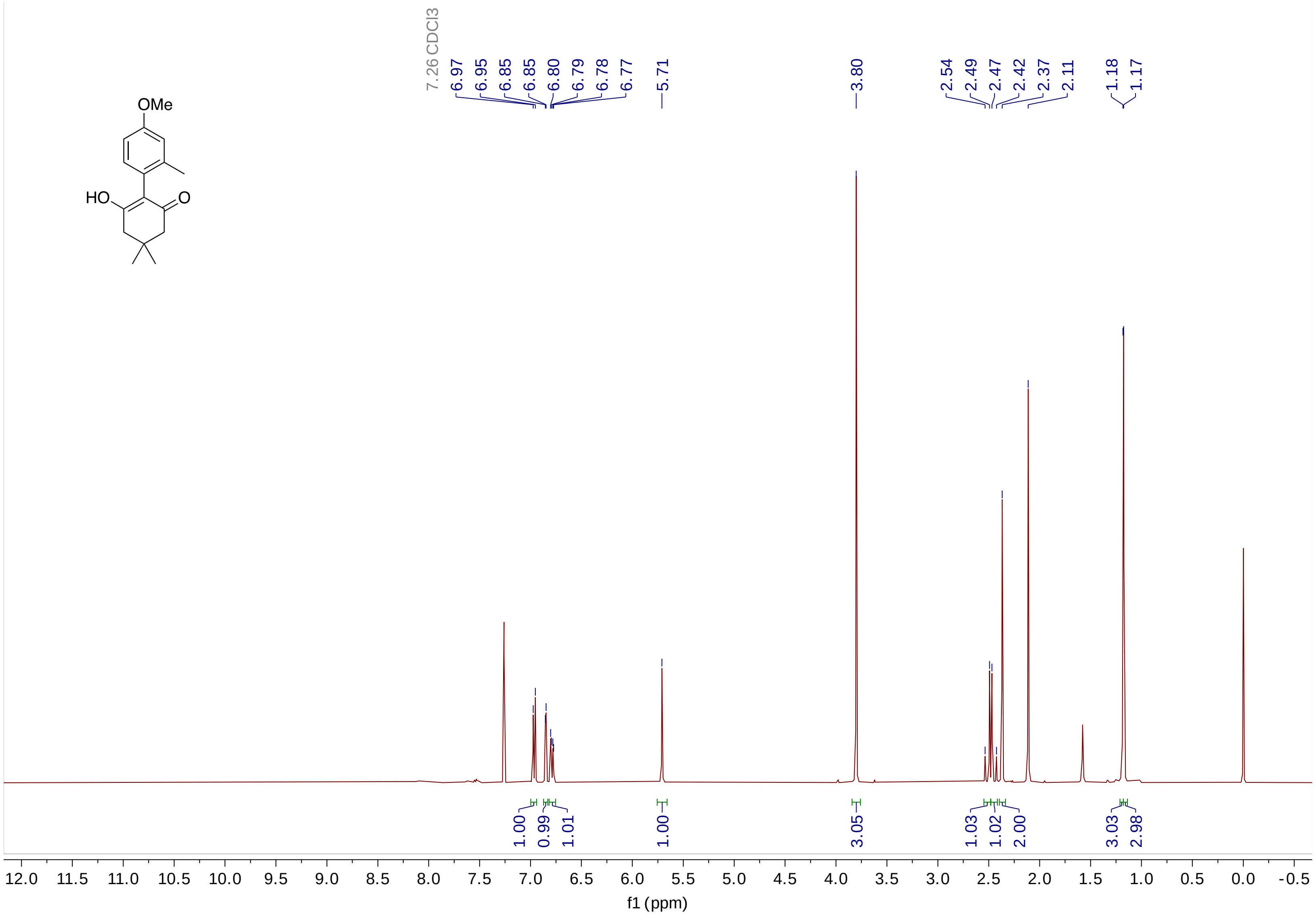

21 - $^{13}C\{^{1}H\}$ NMR (101 MHz, $CDCl_3$):

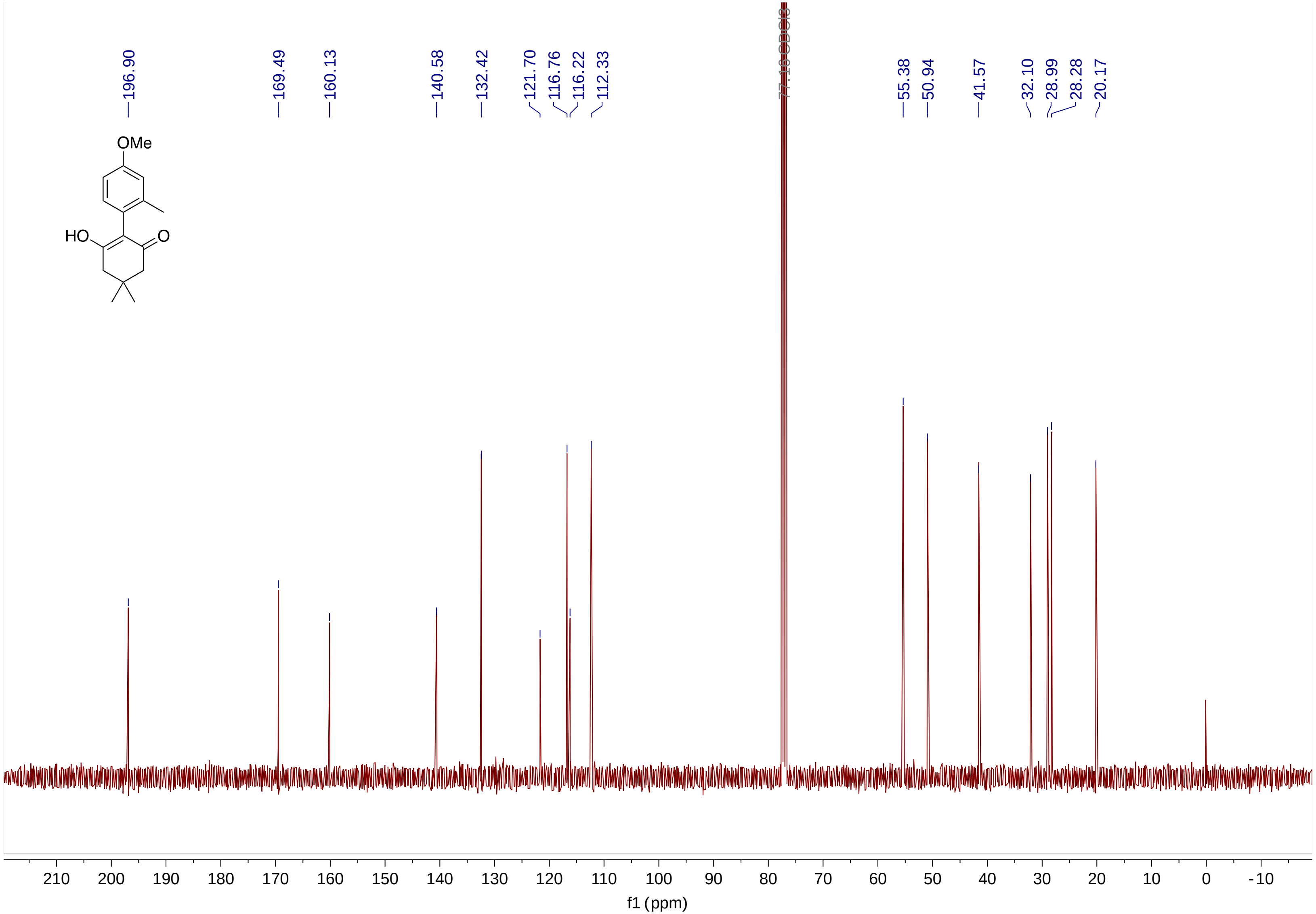

22 - ^{1}H NMR (400 MHz, CDCl₃):

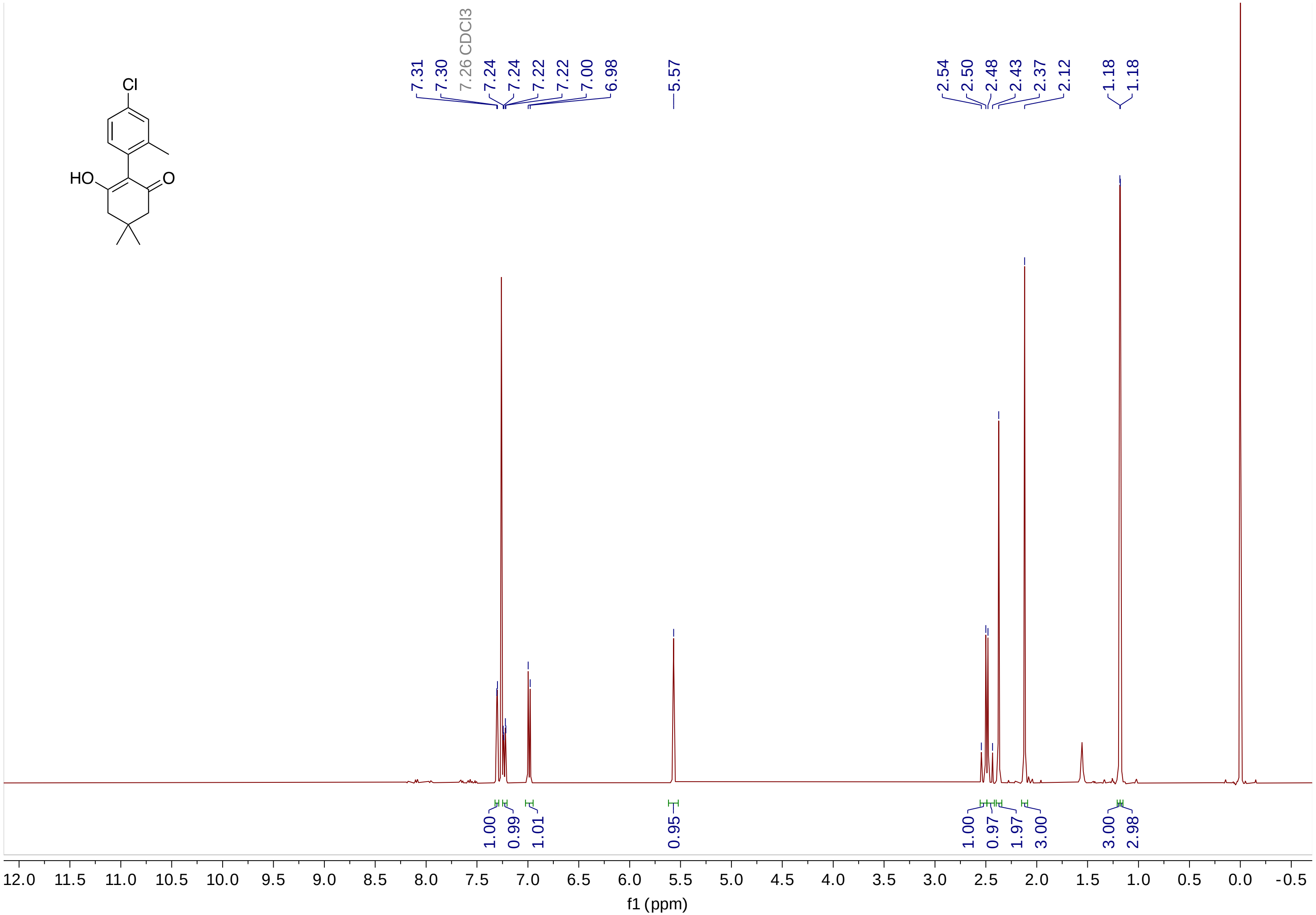

22 - $^{13}C\{^{1}H\}$ NMR (101 MHz, $CDCl_3$):

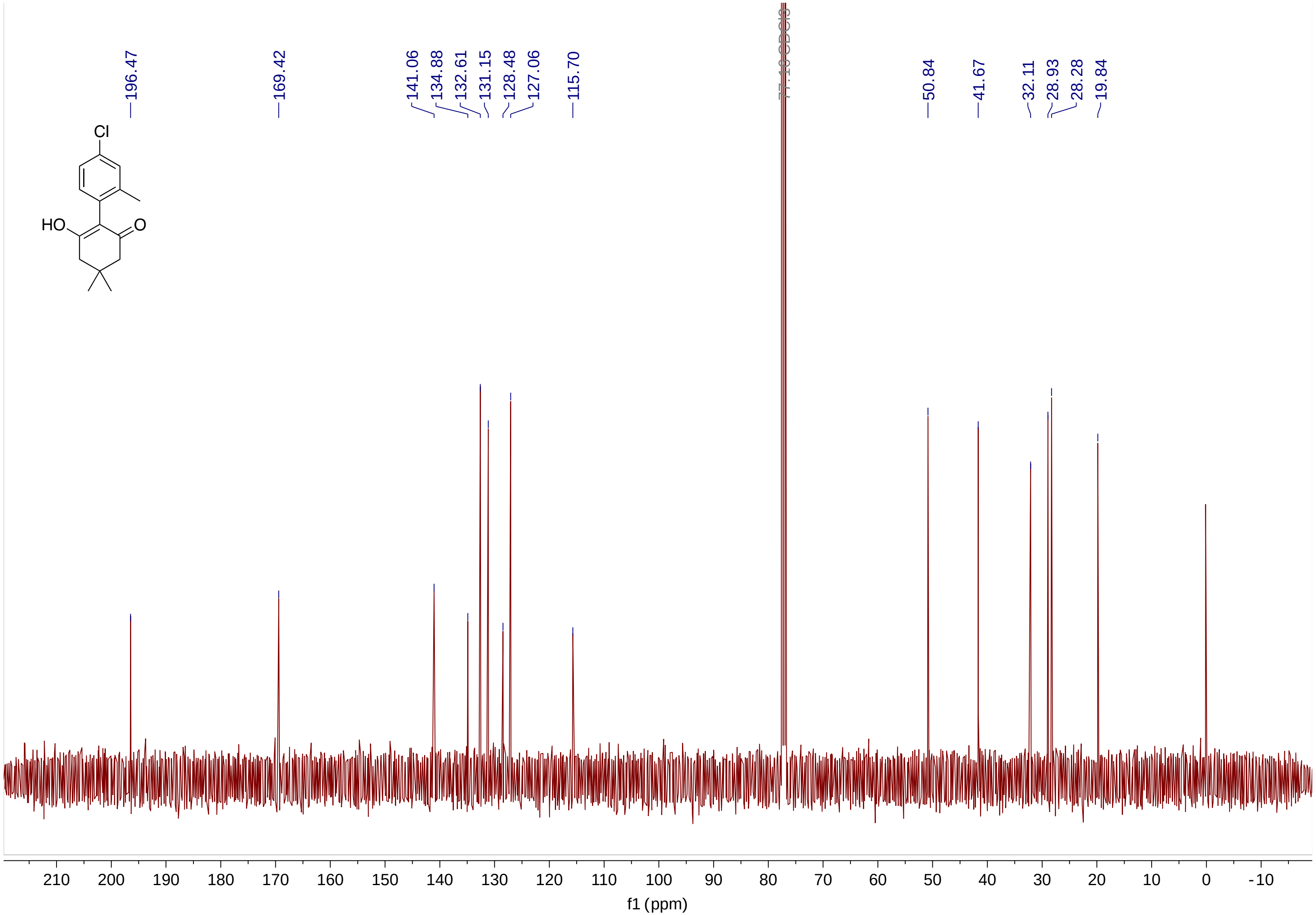

23 - ^{1}H NMR (400 MHz, CD_3OD):

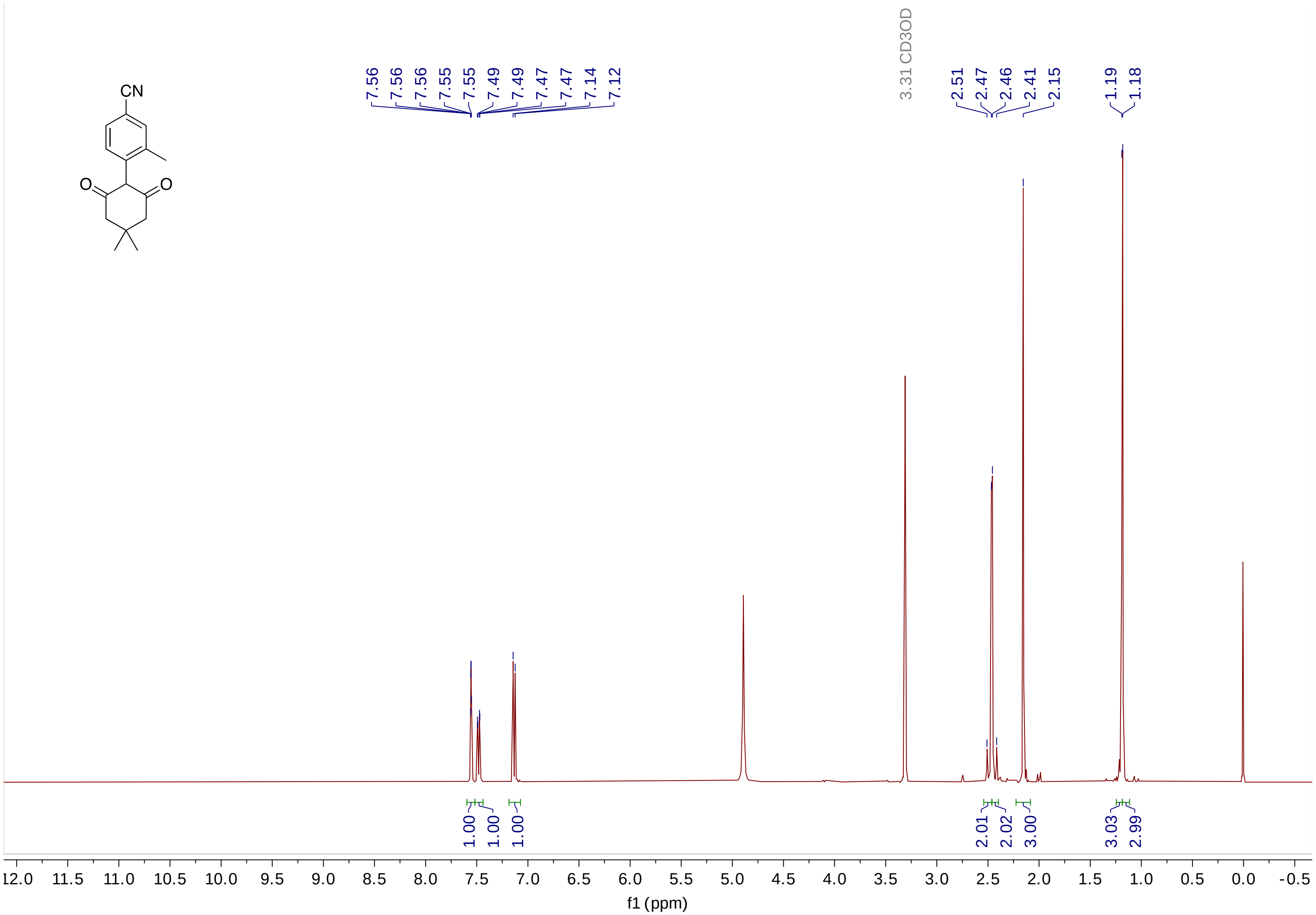

23 - $^{13}C\{^1H\}$ NMR (101 MHz, CD_3OD):

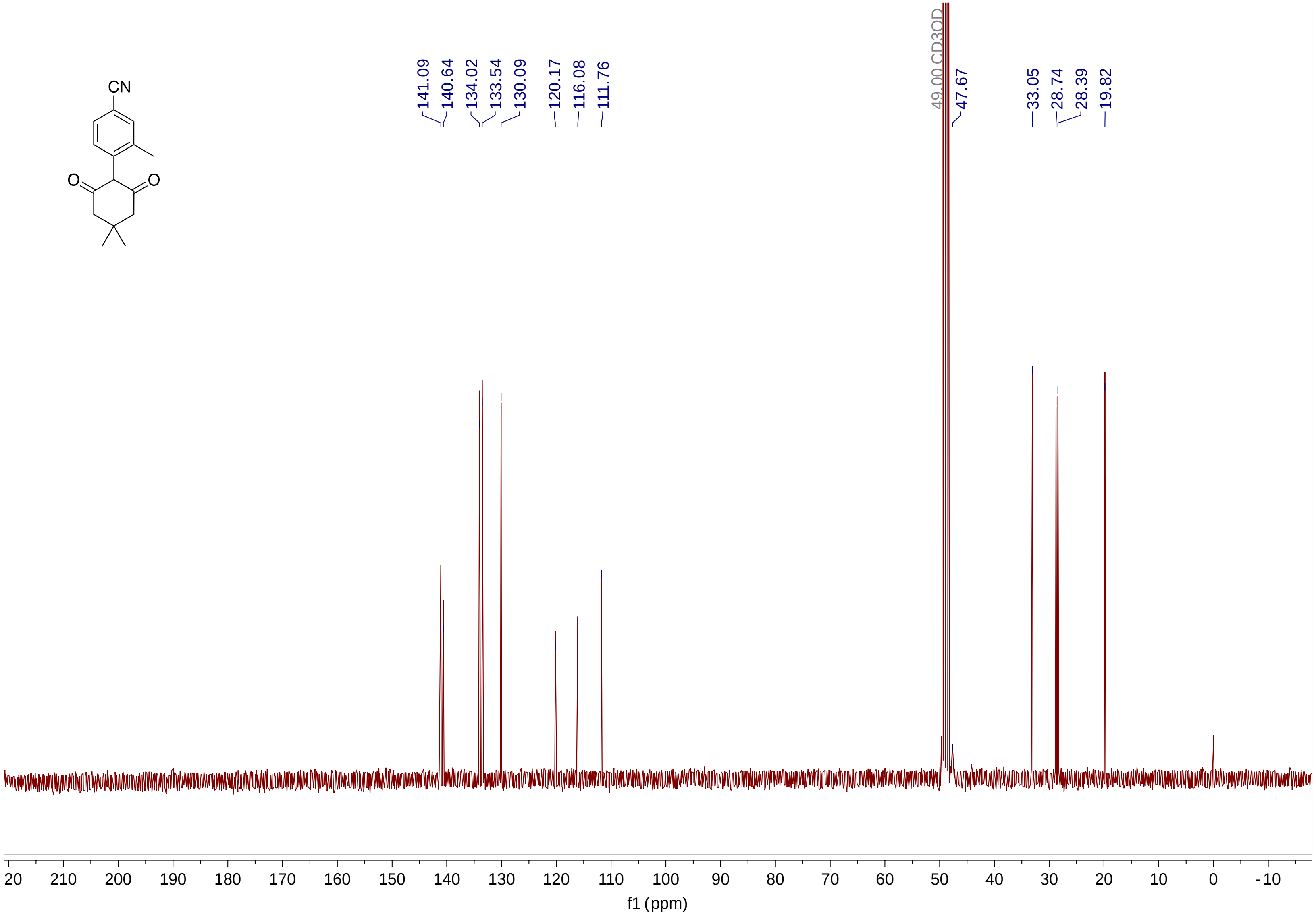

24 - ^{1}H NMR (400 MHz, $CDCl_3$):

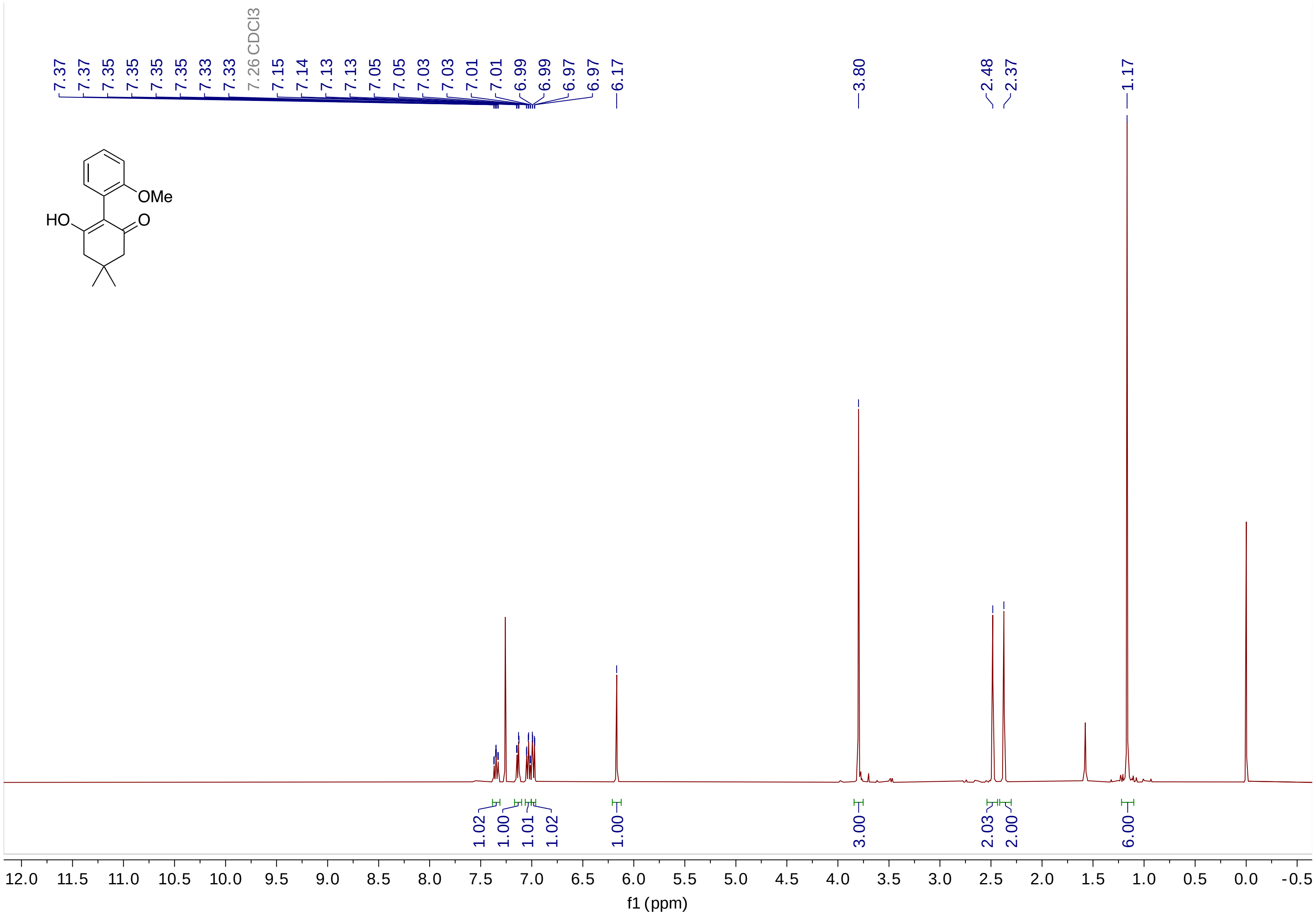

24 - $^{13}C\{^1H\}$ NMR (101 MHz, $CDCl_3$):

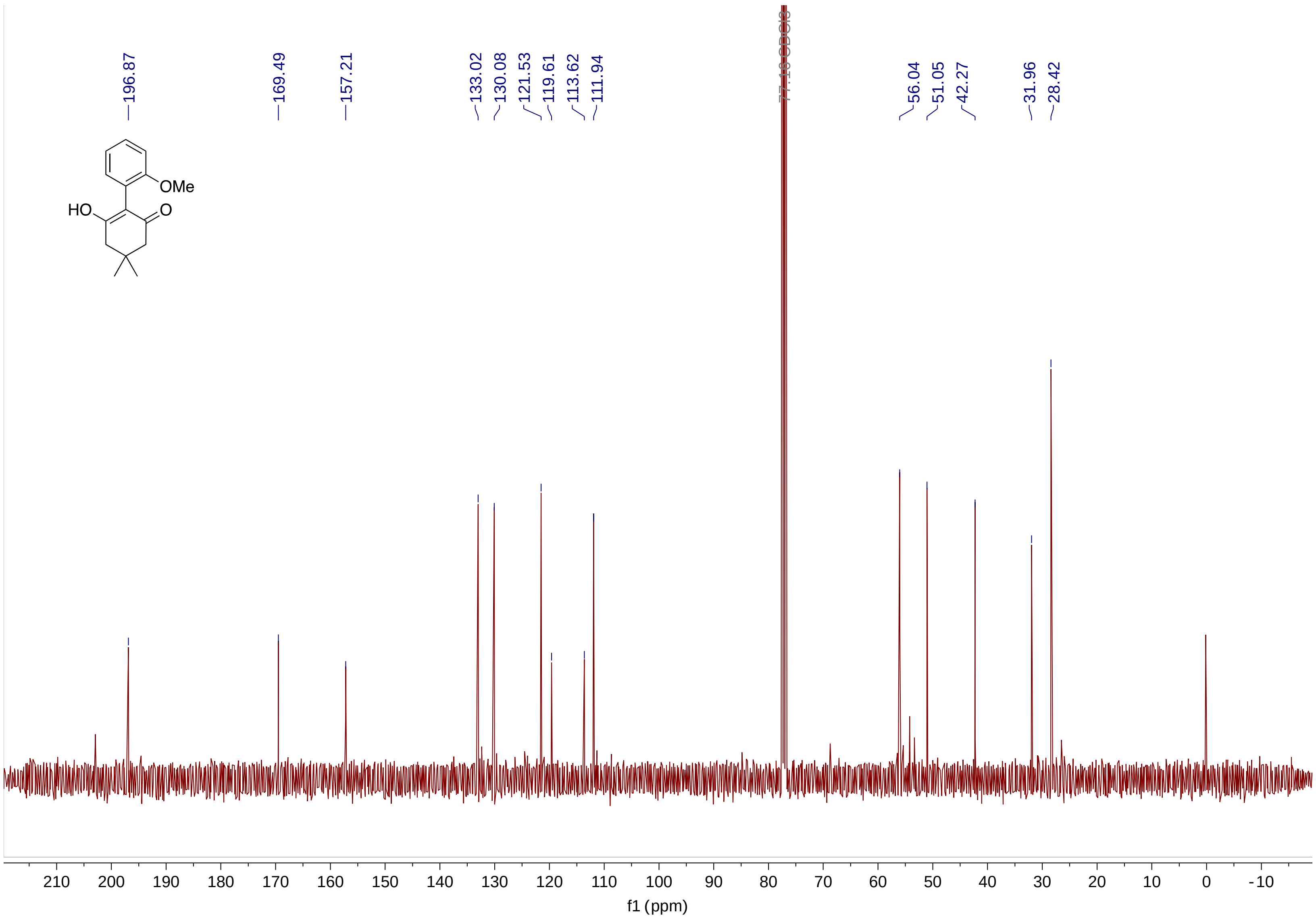

25 - ^{1}H NMR (400 MHz, $CDCl_3$):

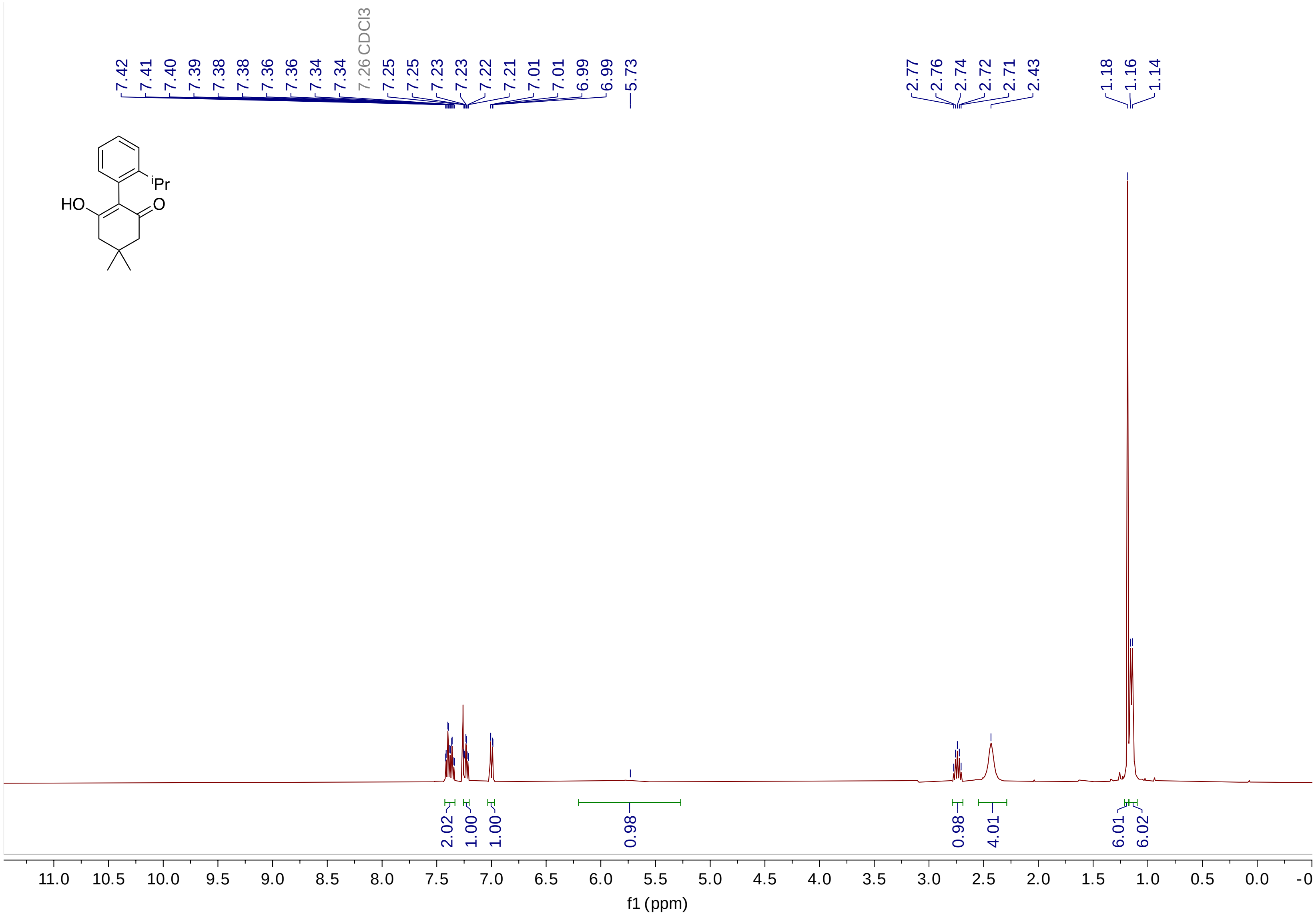

25 - $^{13}C\{^{1}H\}$ NMR (101 MHz, $CDCl_3$):

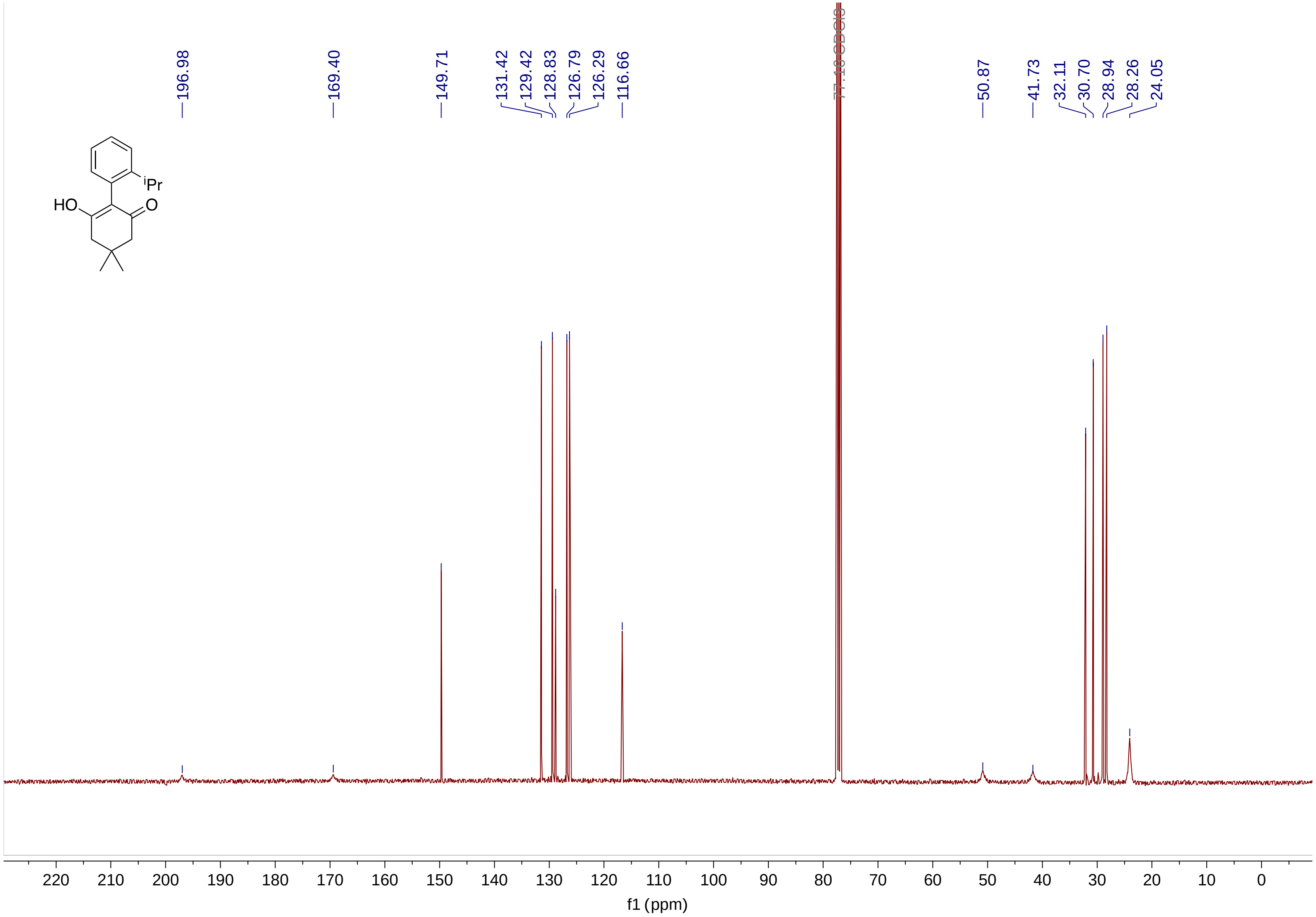

26 - ^{1}H NMR (400 MHz, $CDCl_3$):

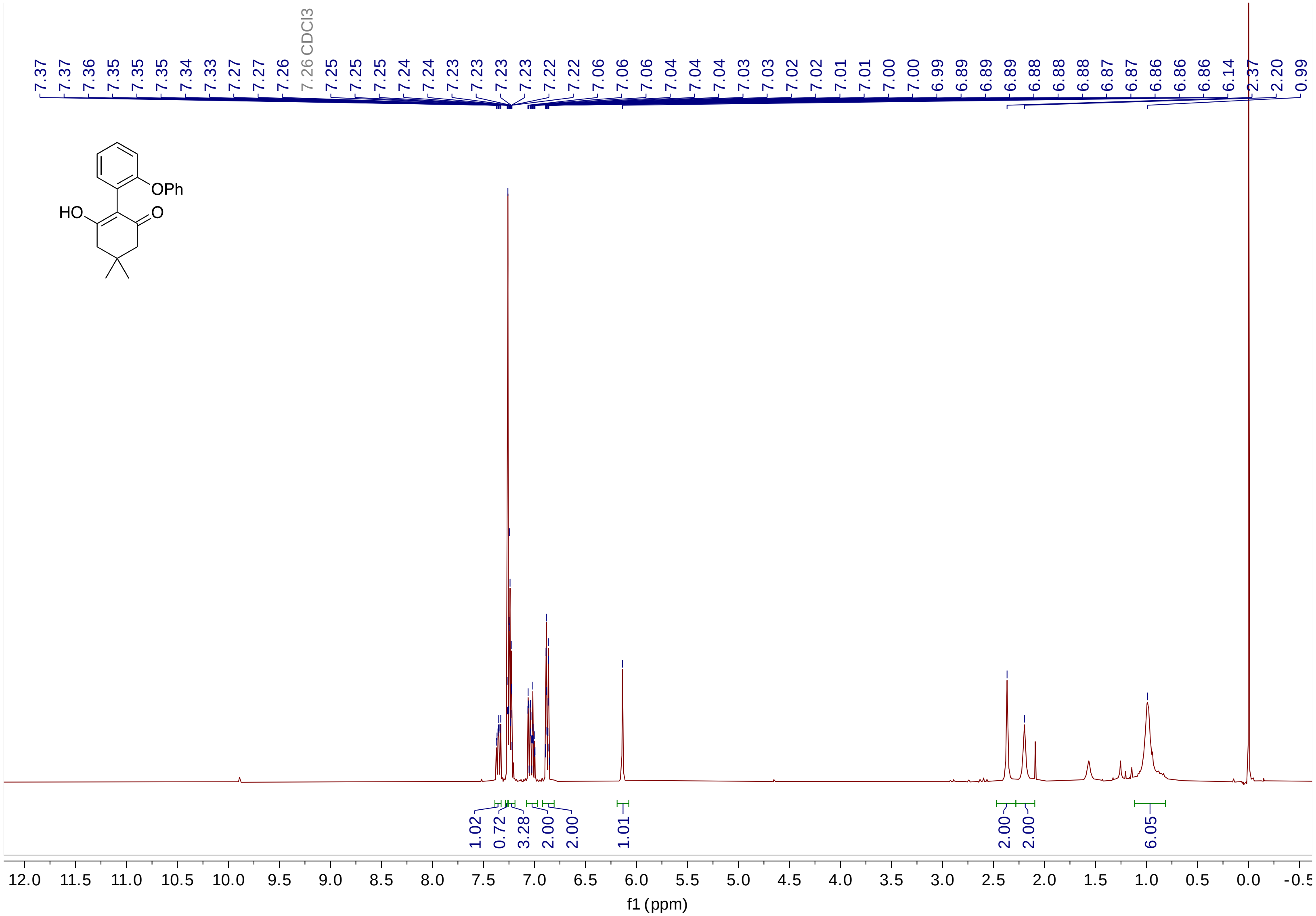

26 - $^{13}C\{^1H\}$ NMR (101 MHz, $CDCl_3$):

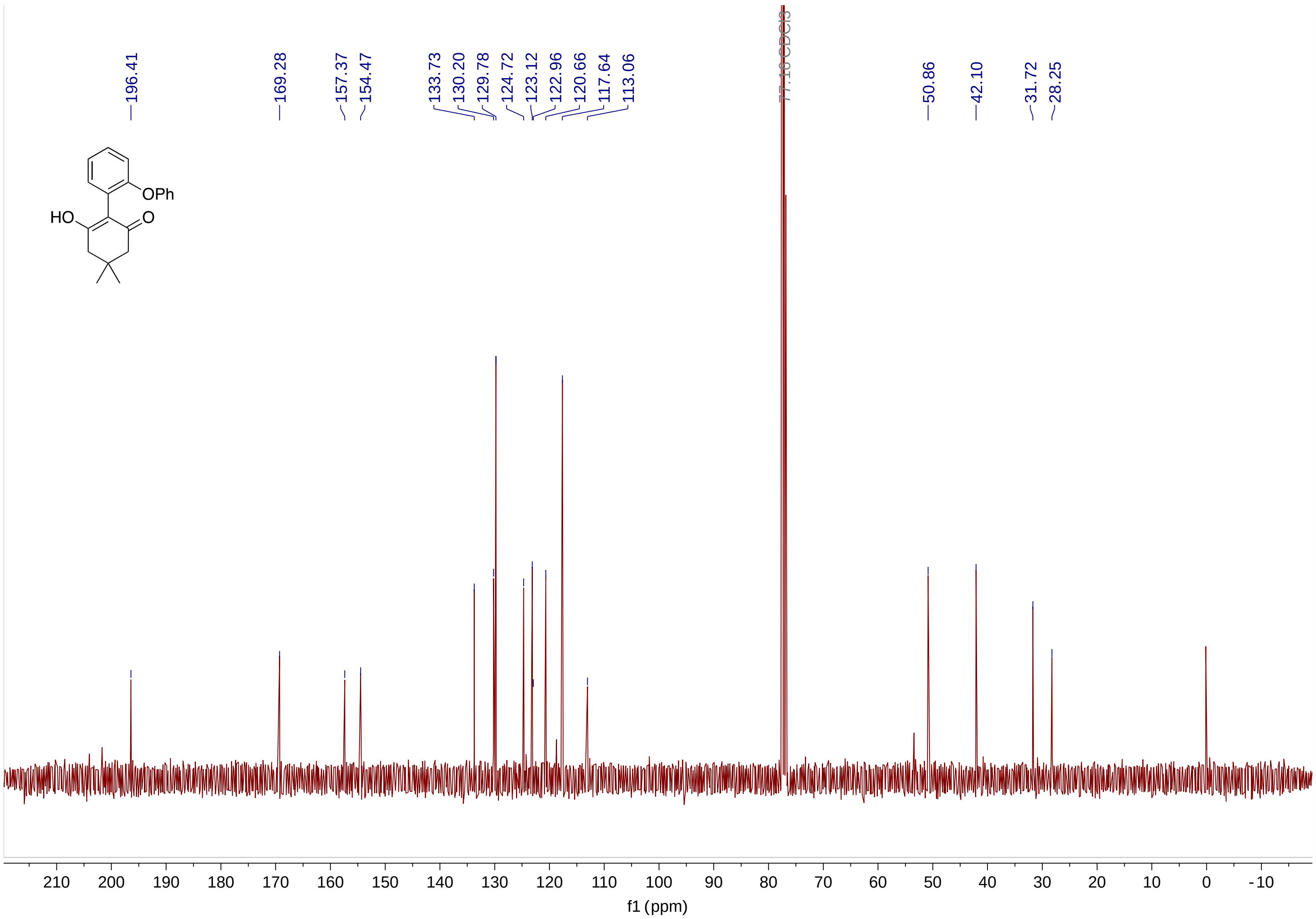

27 - ^{1}H NMR (400 MHz, CD$_3$OD):

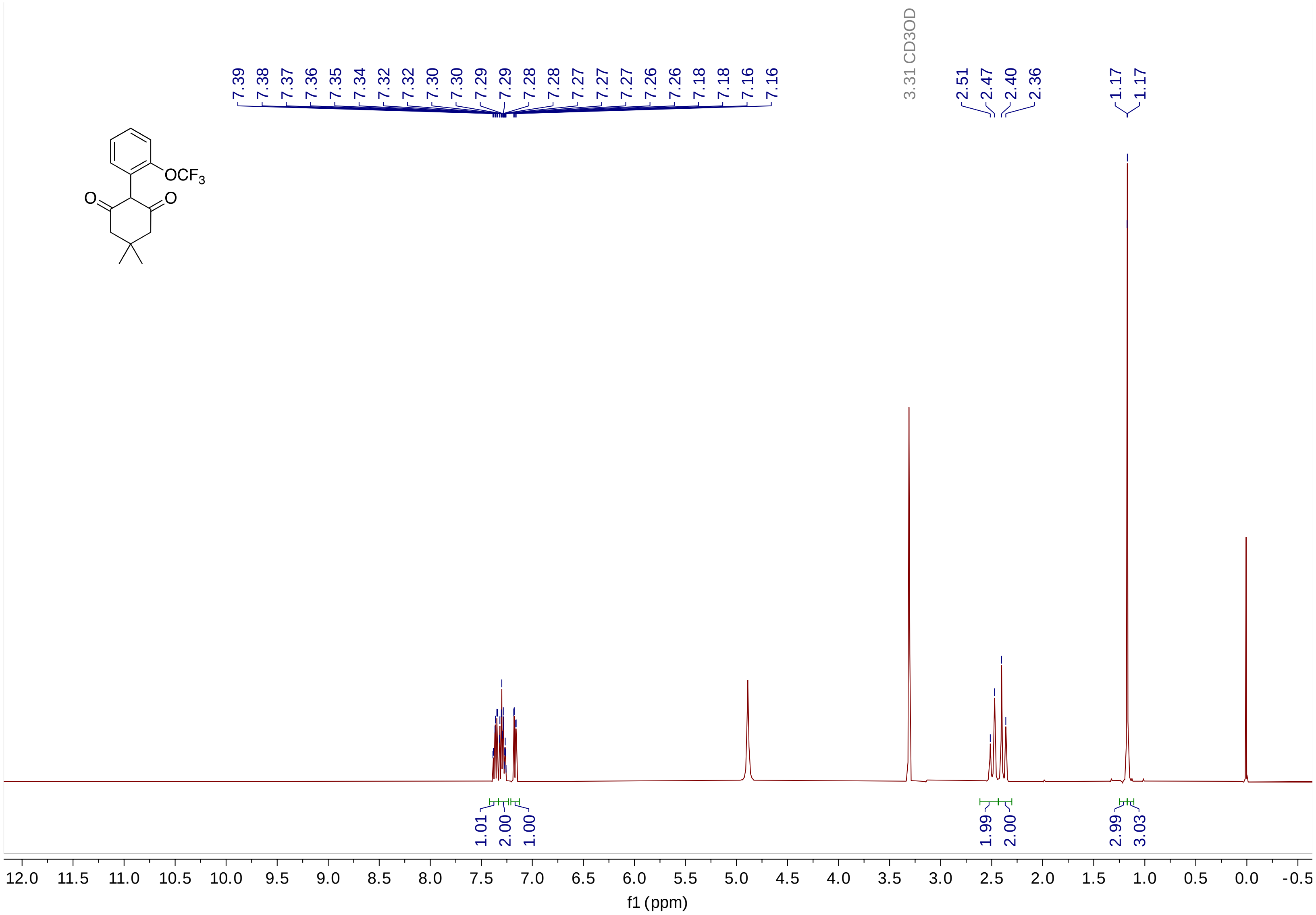

27 - $^{13}C\{^{1}H\}$ NMR (126 MHz, CD_3OD):

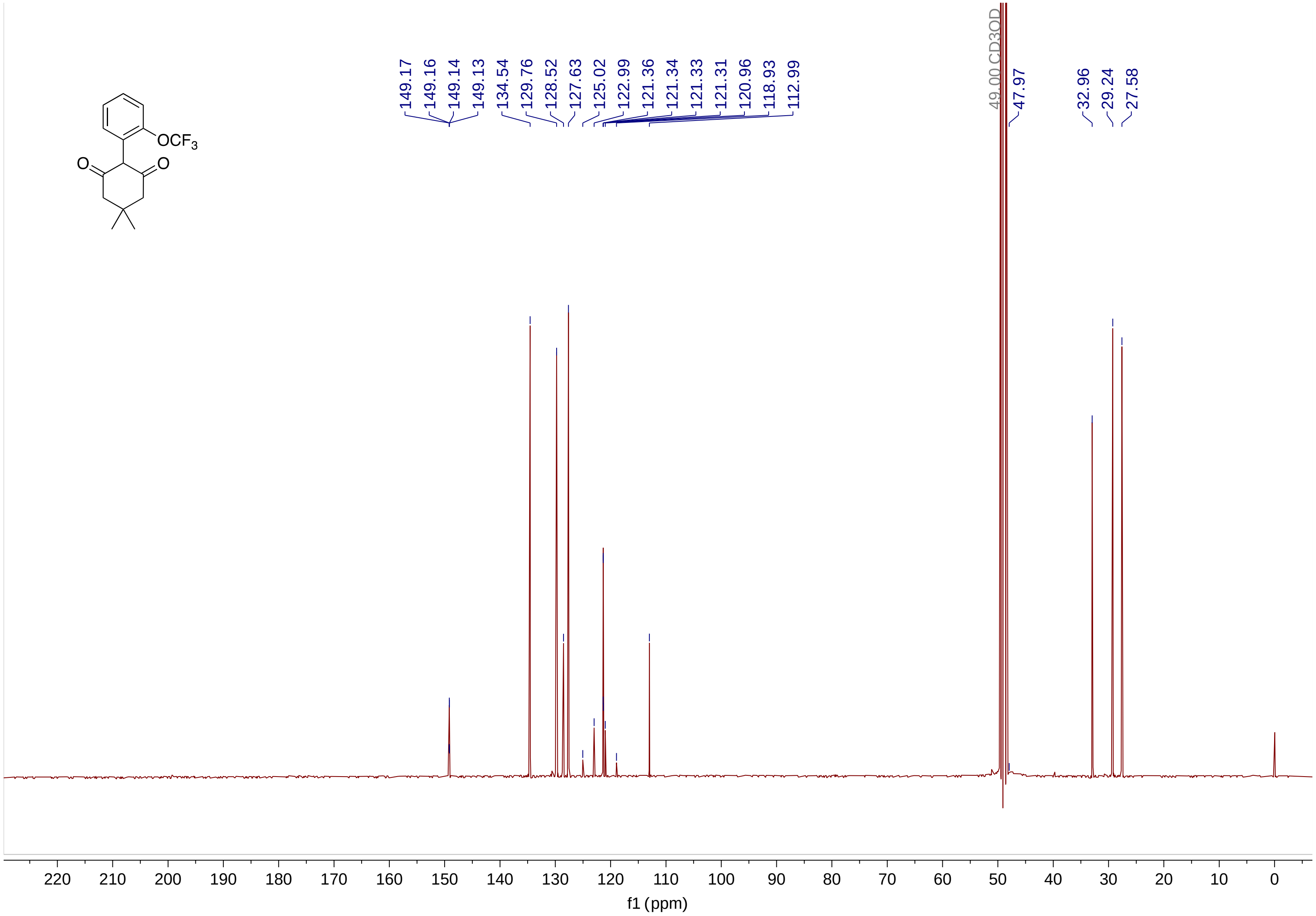

27 - ^{19}F NMR (376 MHz, CD_3OD):

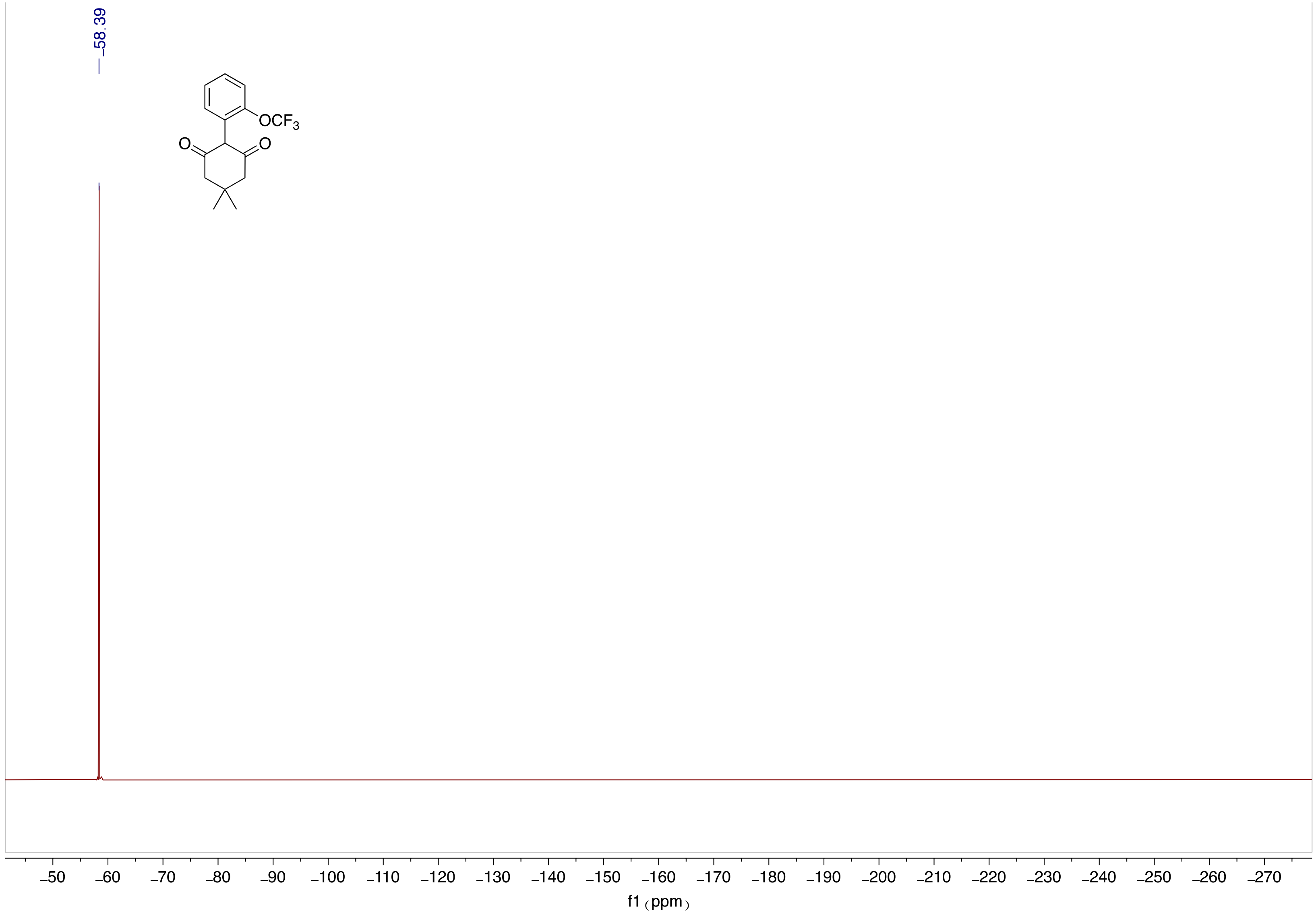

28 - ^{1}H NMR (400 MHz, CDCl$_3$):

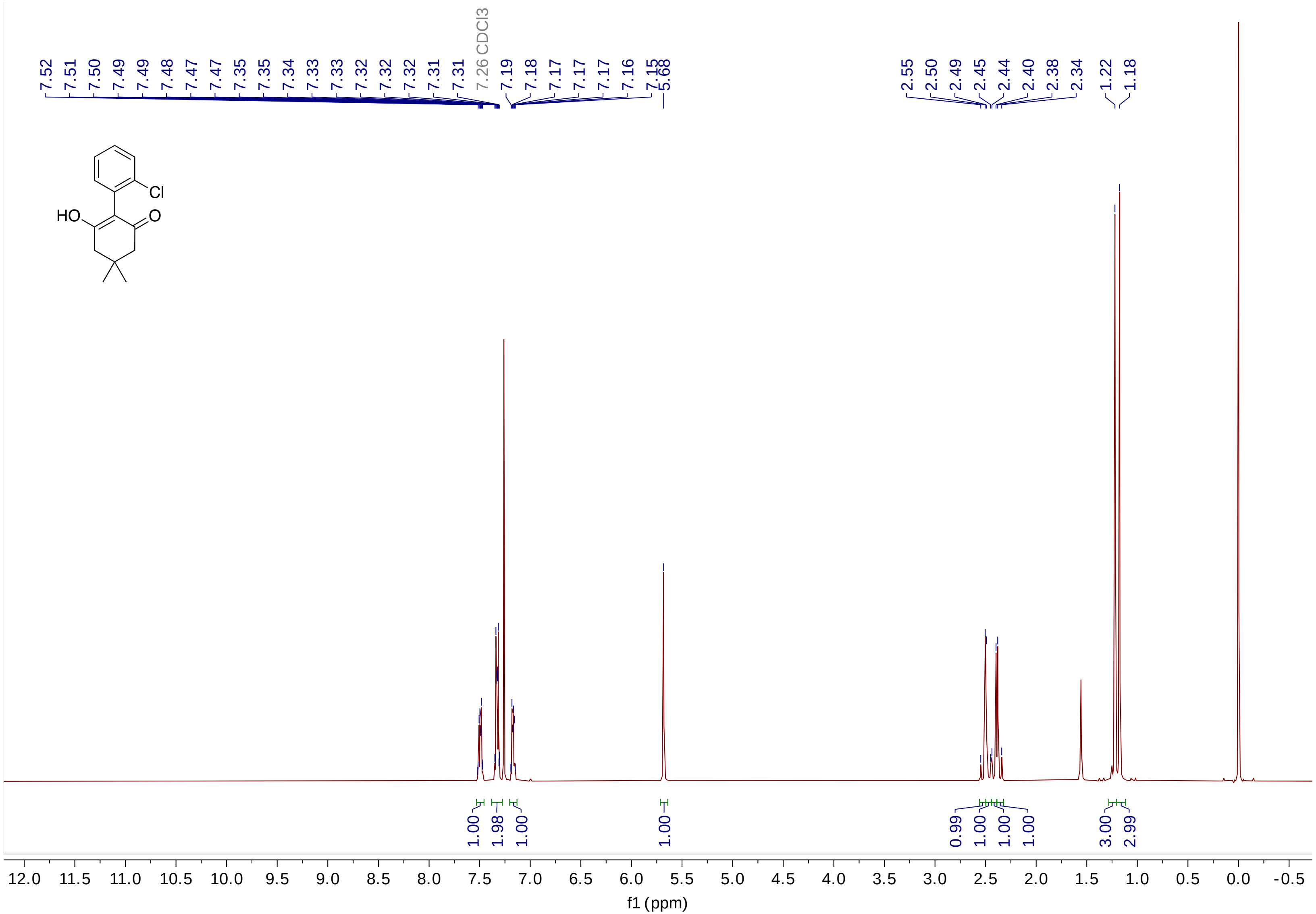

28 - $^{13}C\{^1H\}$ NMR (101 MHz, $CDCl_3$):

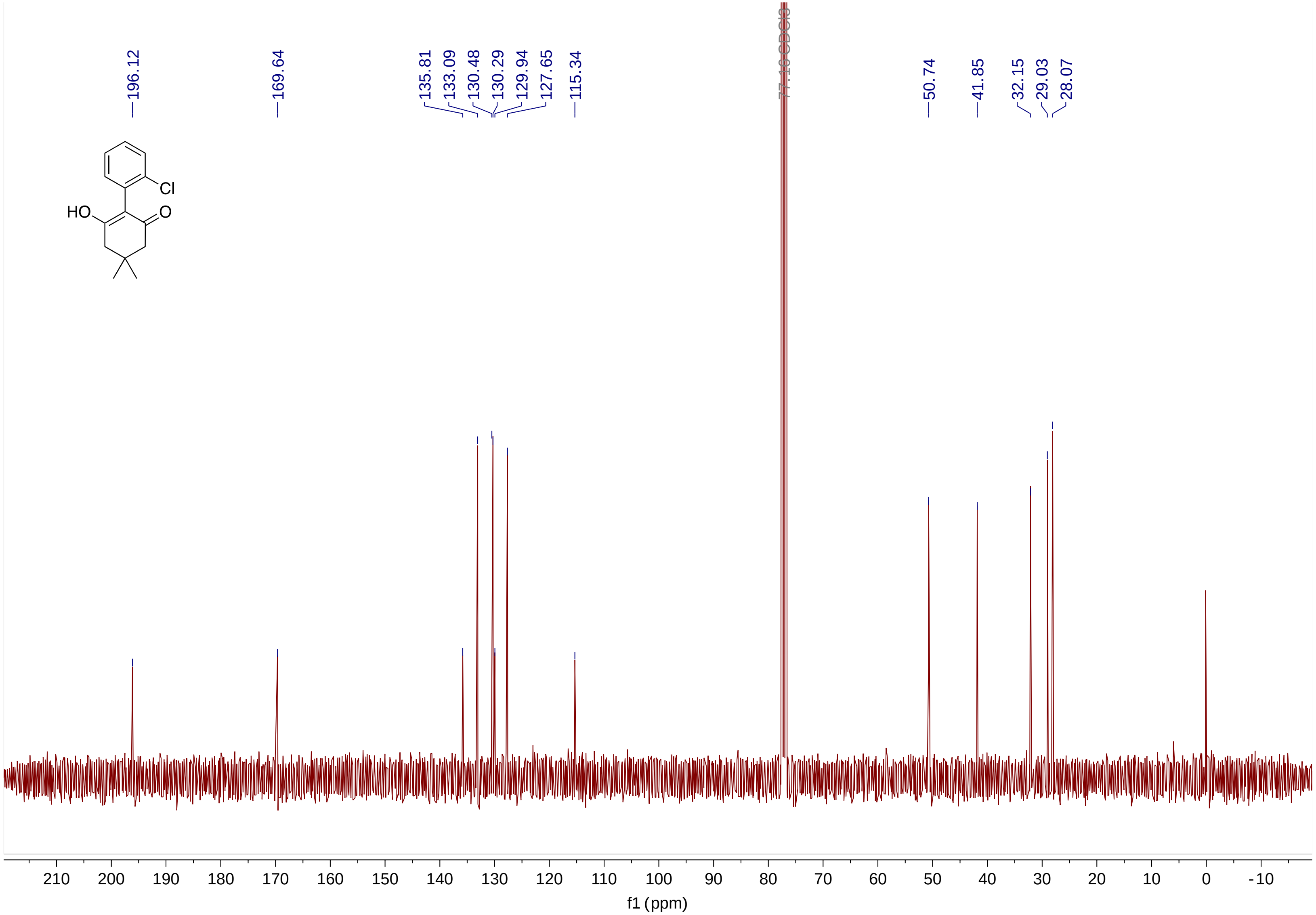

29 - ^{1}H NMR (400 MHz, CD_3OD):

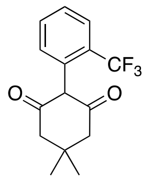

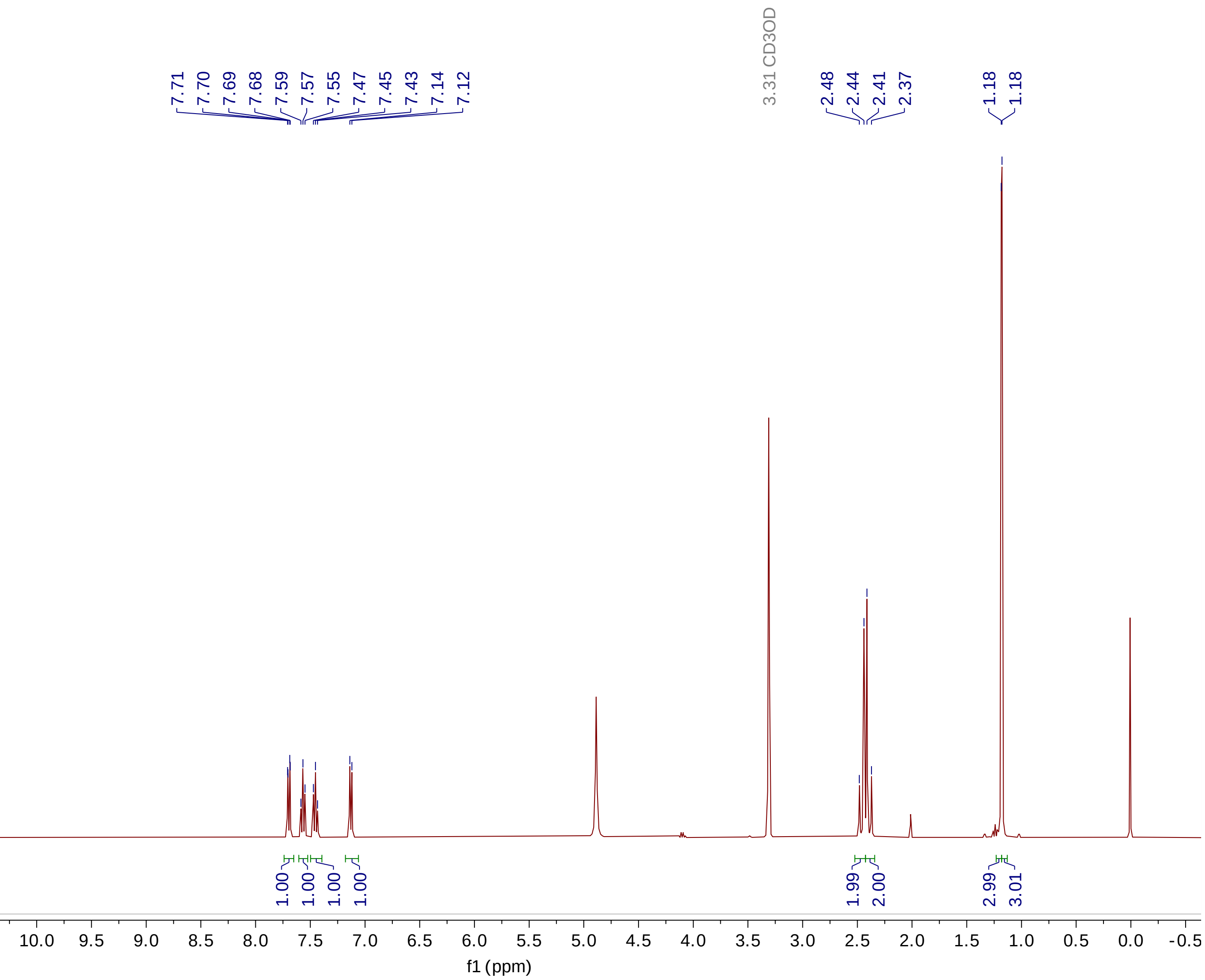

29 - $^{13}C\{^1H\}$ NMR (101 MHz, CD_3OD):

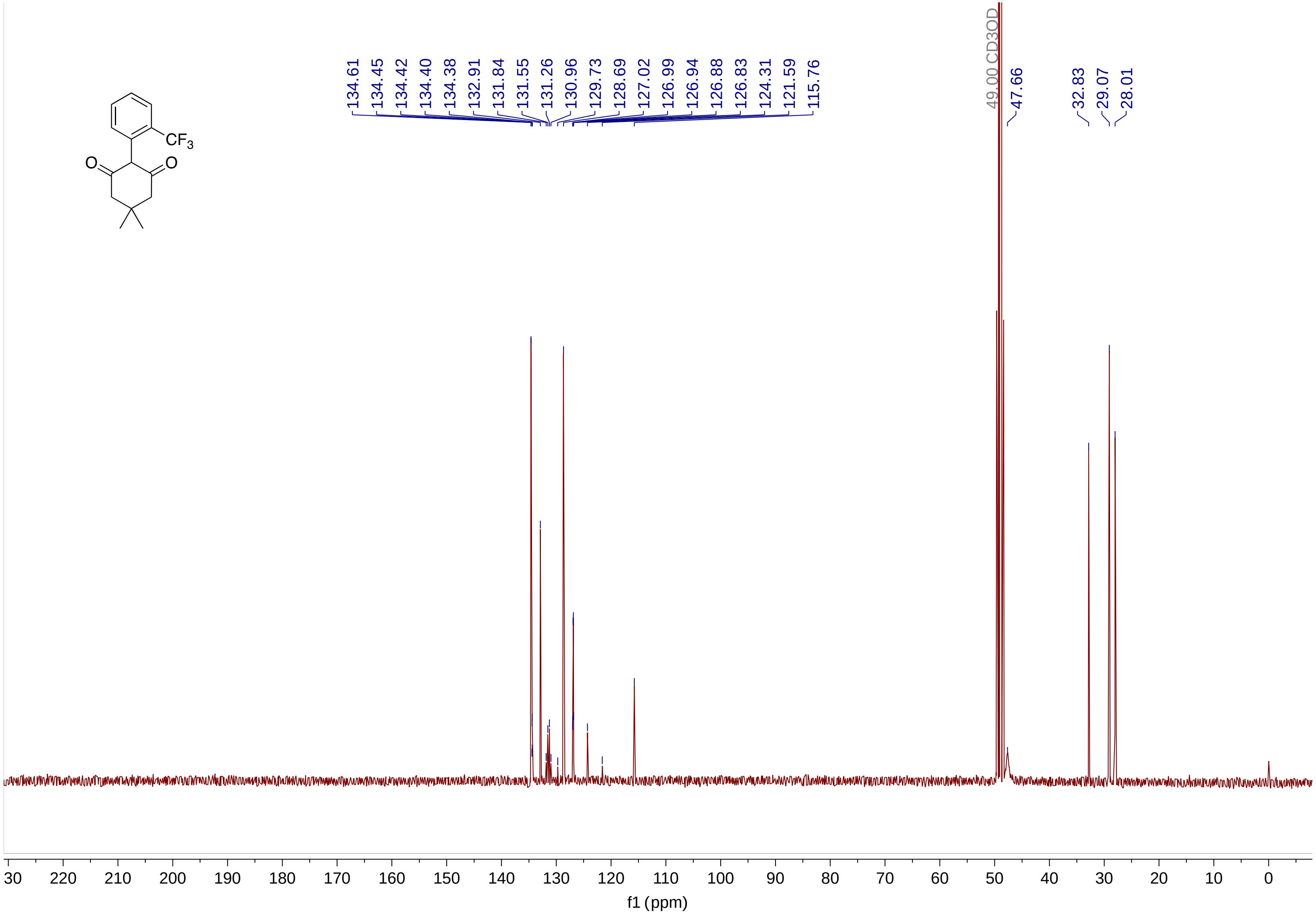

29 - ^{19}F NMR (376 MHz, CD_3OD):

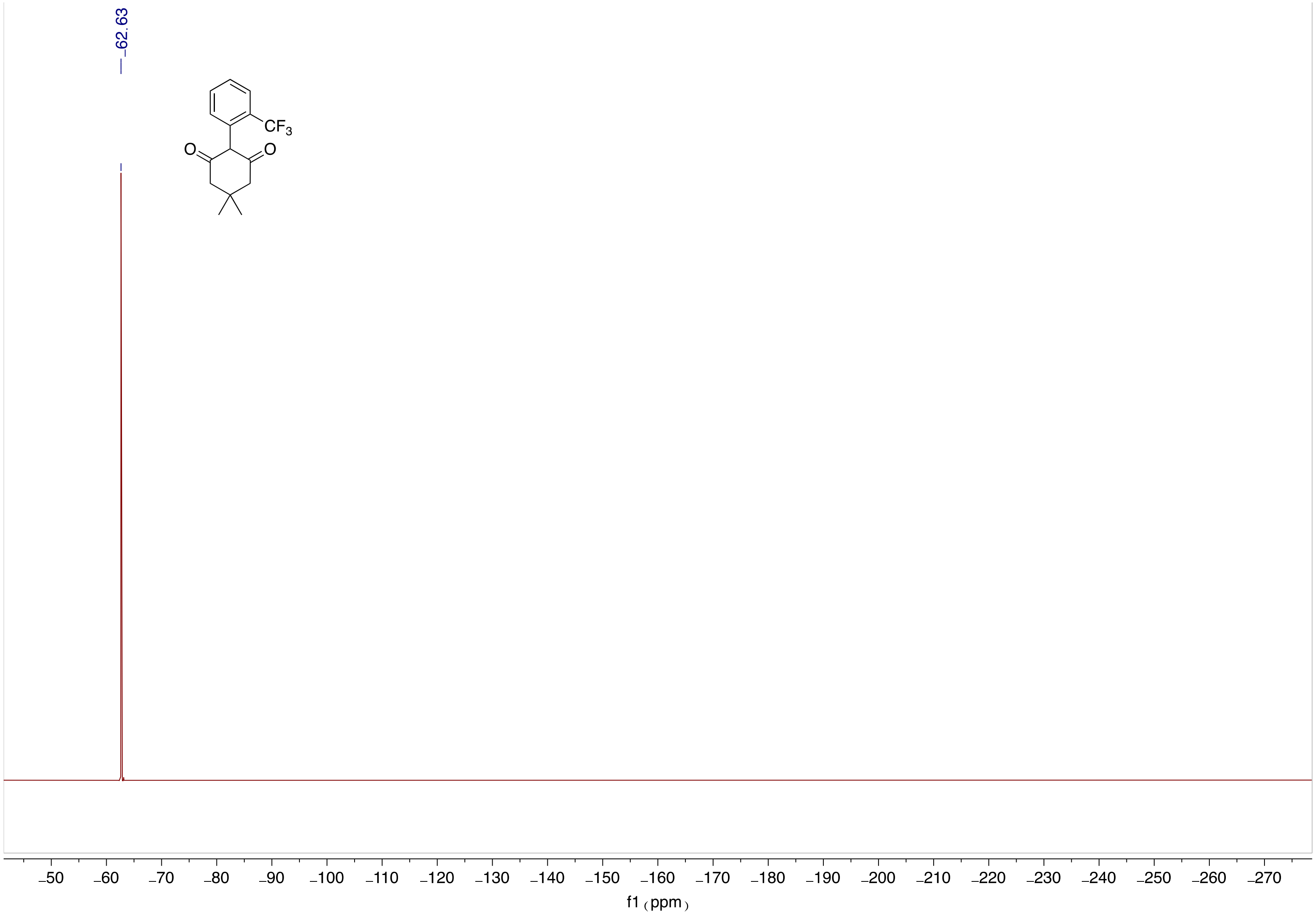

30 - ^{1}H NMR (400 MHz, CD_3OD):

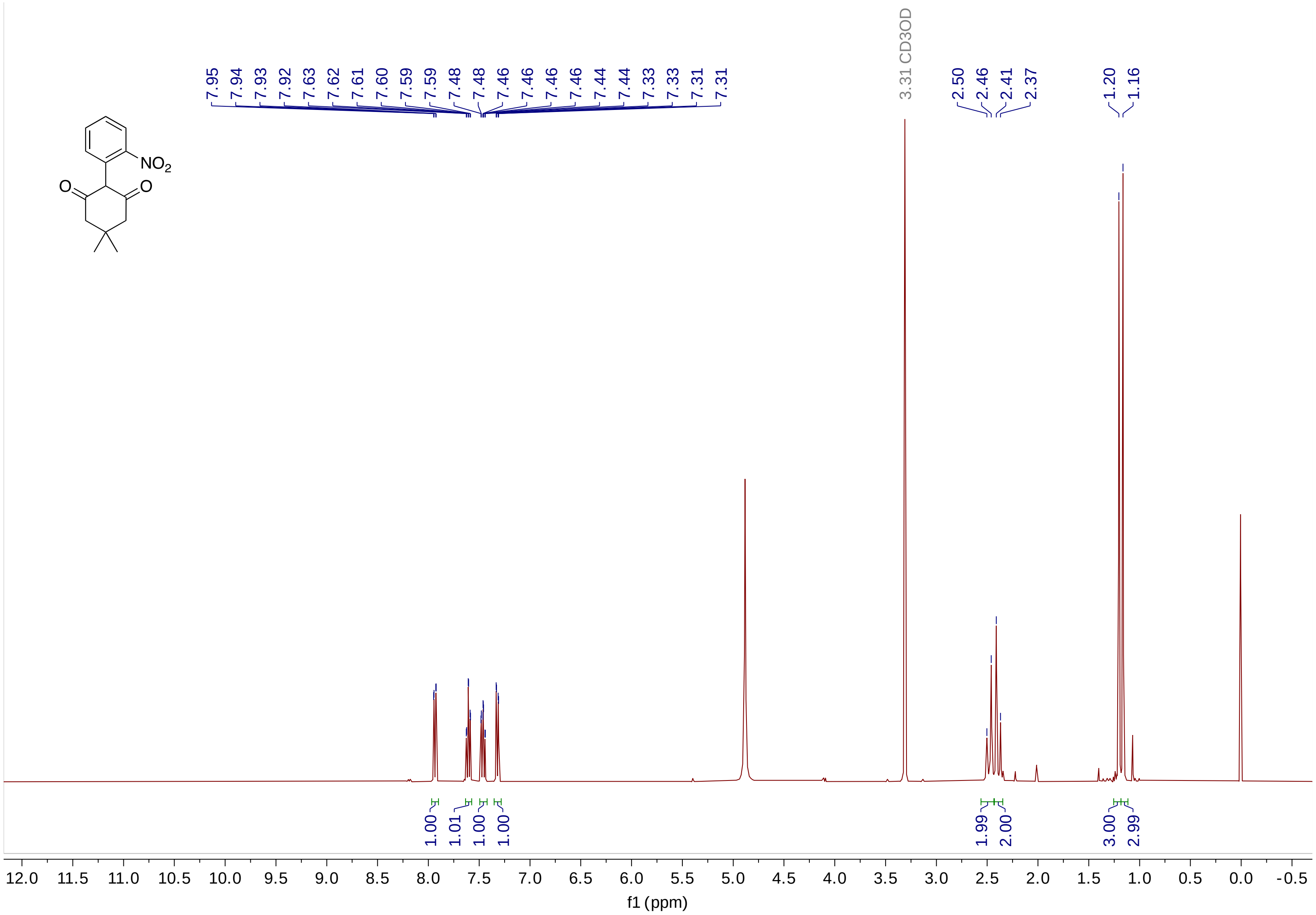

30 - $^{13}C\{^1H\}$ NMR (101 MHz, CD_3OD):

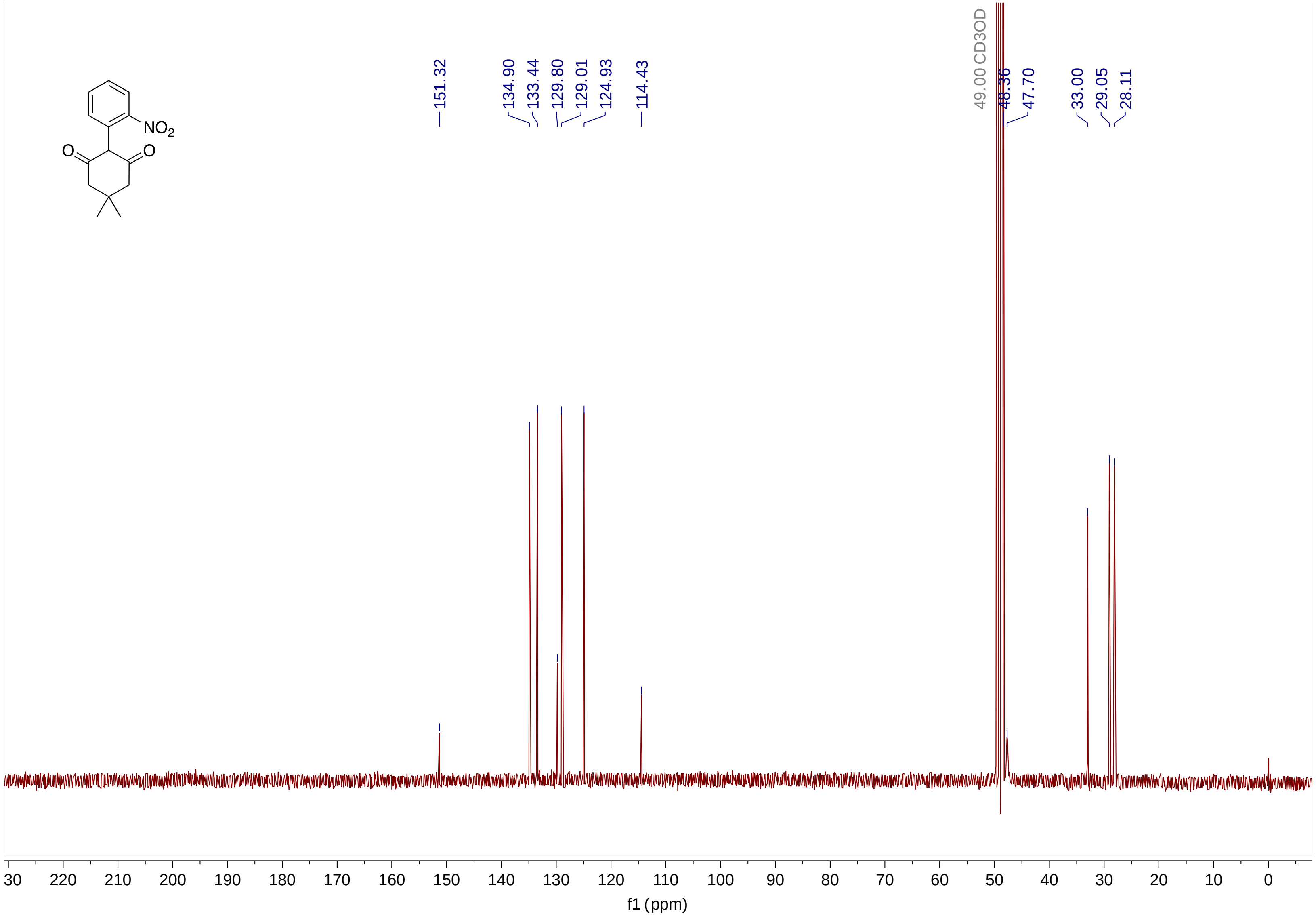

31 - ^{1}H NMR (400 MHz, $CDCl_3$):

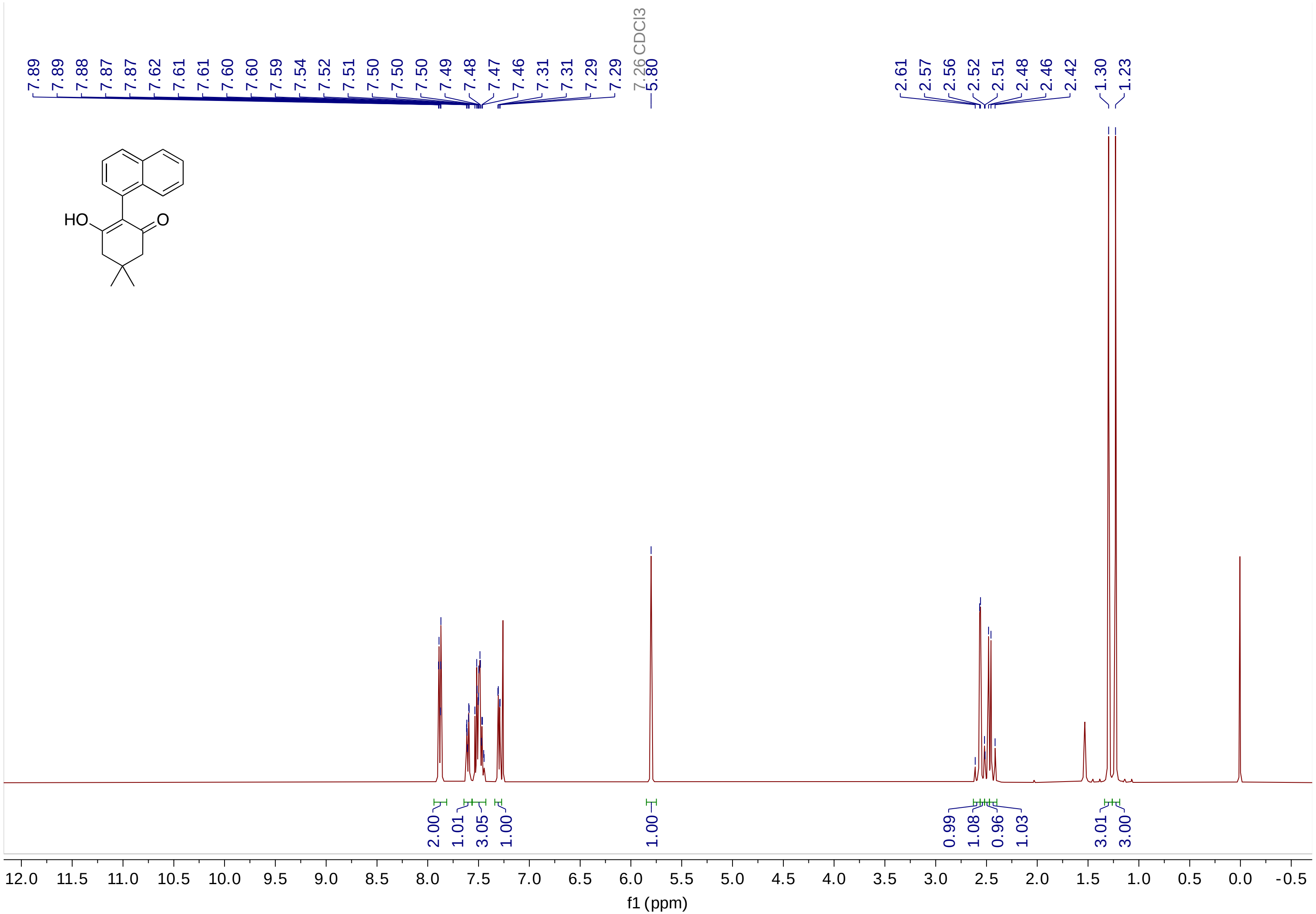

31 - $^{13}C\{^1H\}$ NMR (101 MHz, CDCl$_3$):

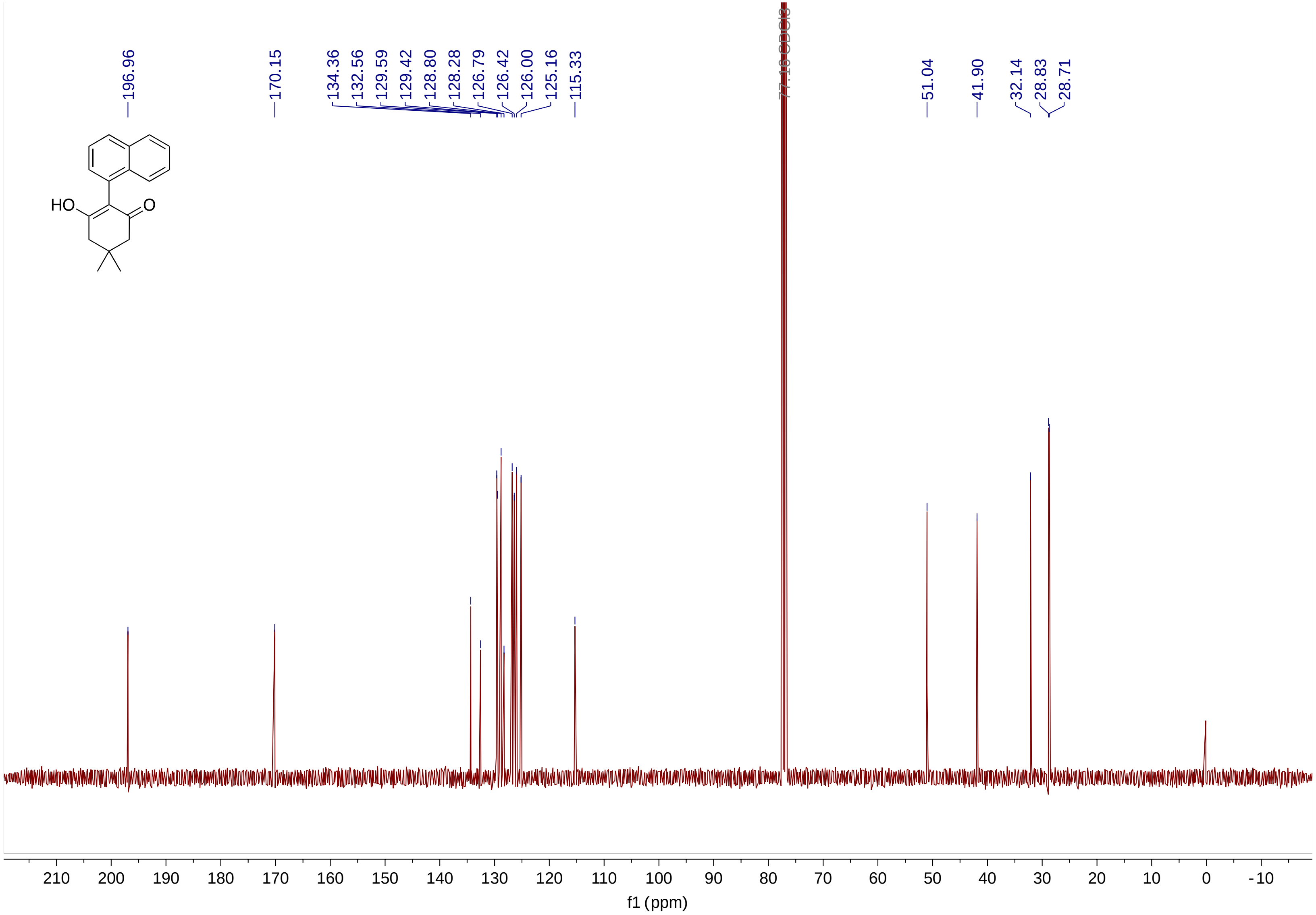

32 - ^{1}H NMR (400 MHz, CD_3OD):

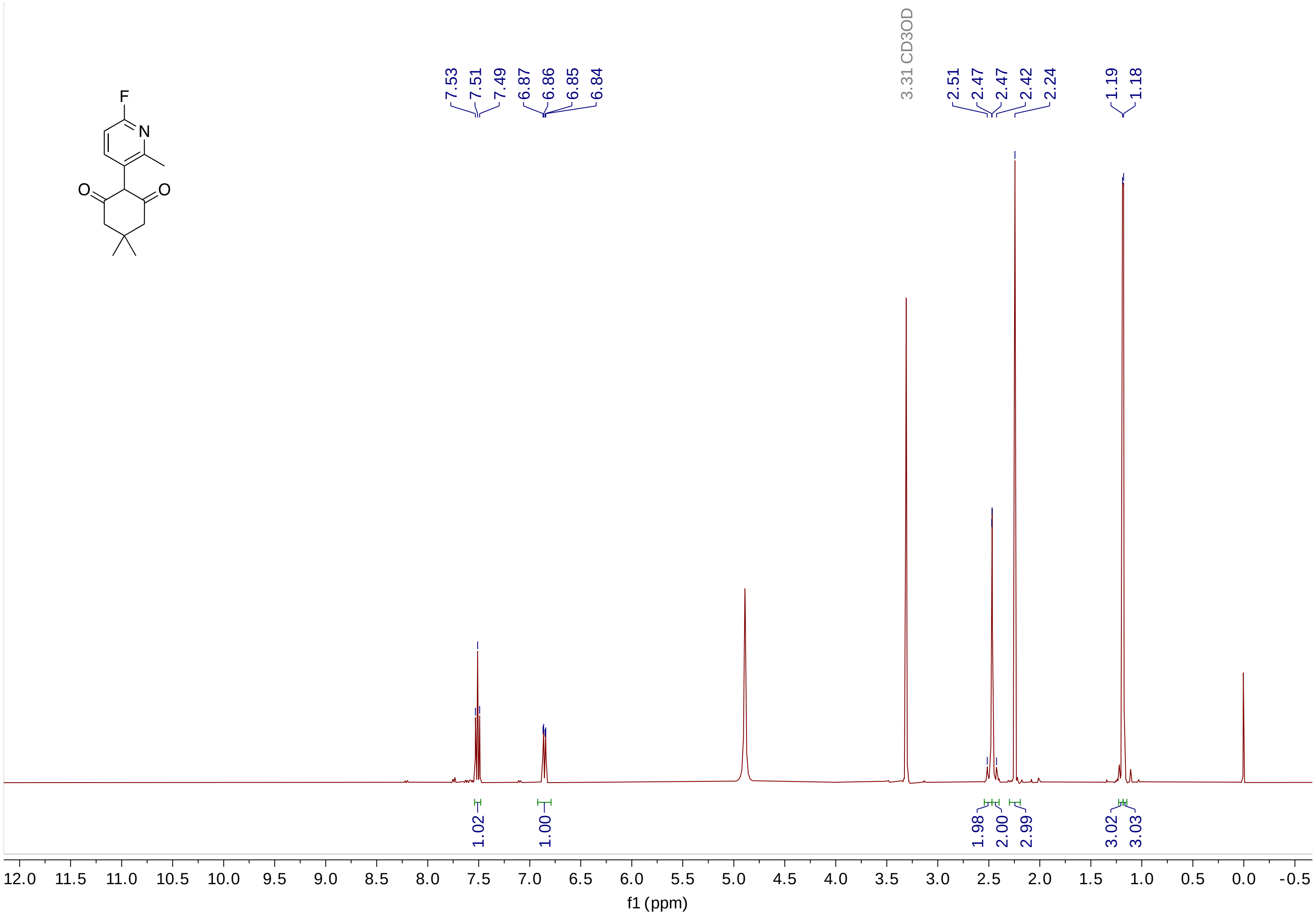

32 - $^{13}C\{^{1}H\}$ NMR (101 MHz, CD_3OD):

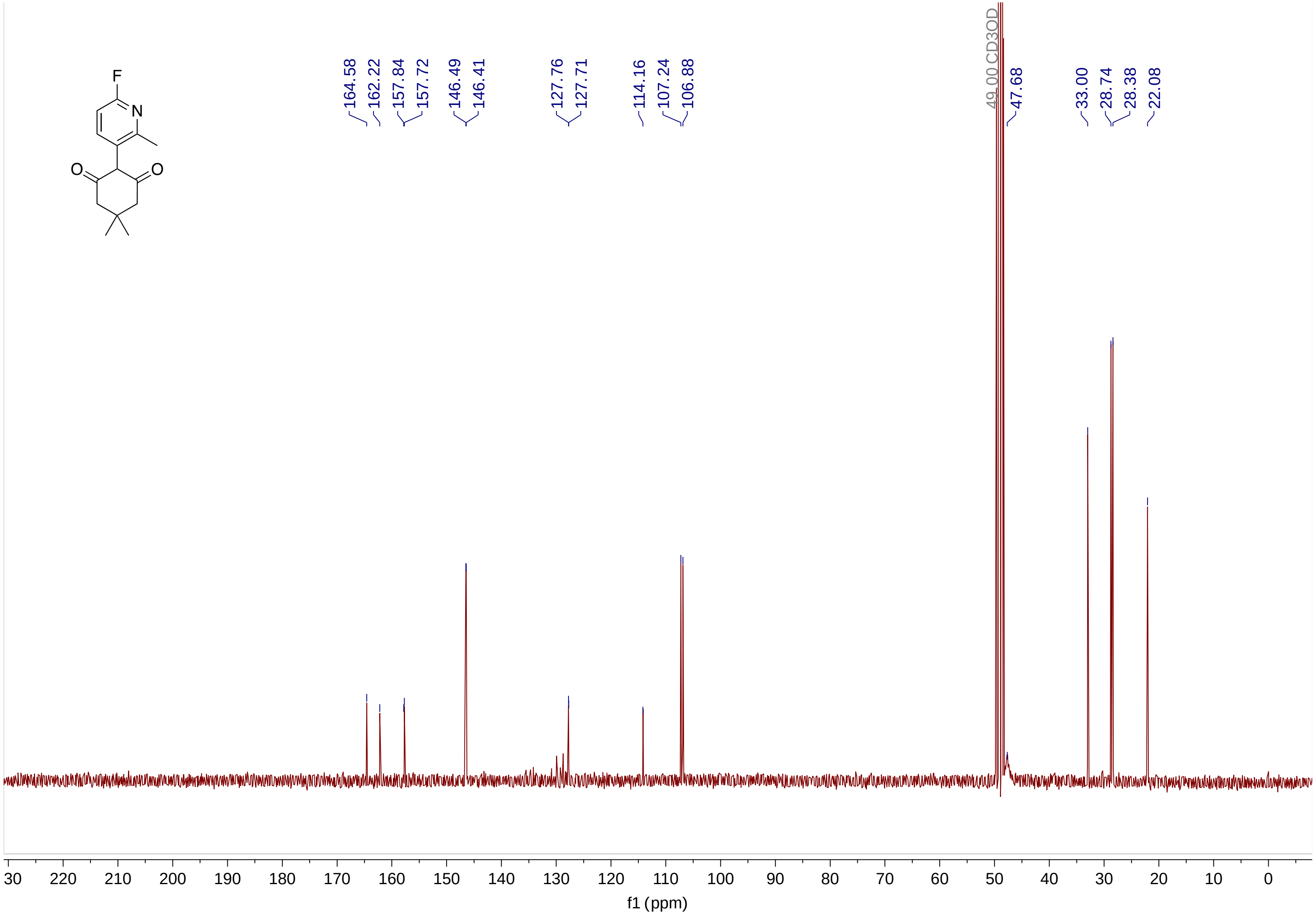

32 - ^{19}F NMR (376 MHz, CD_3OD):

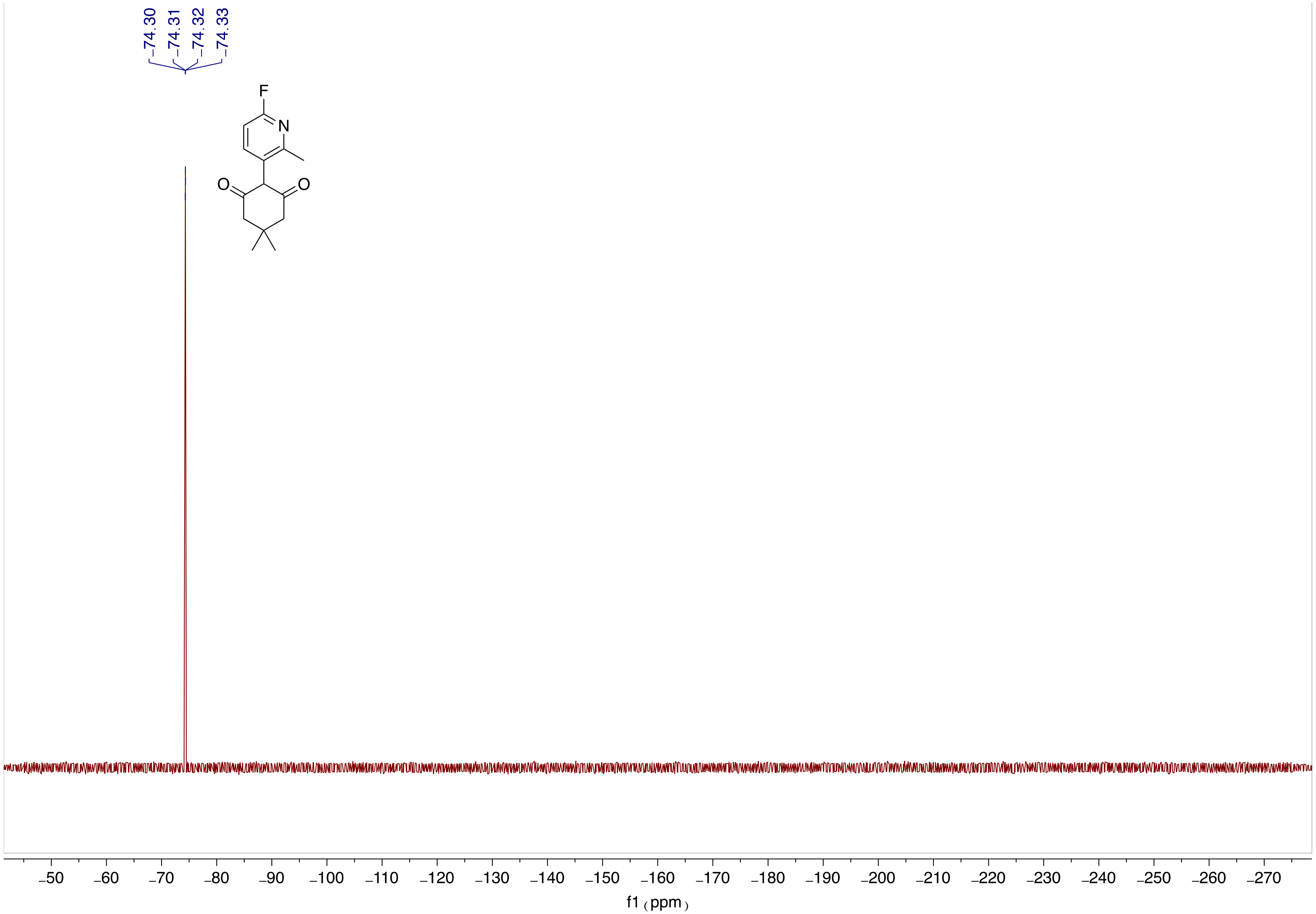

33 - ^{1}H NMR (400 MHz, $CDCl_3$):

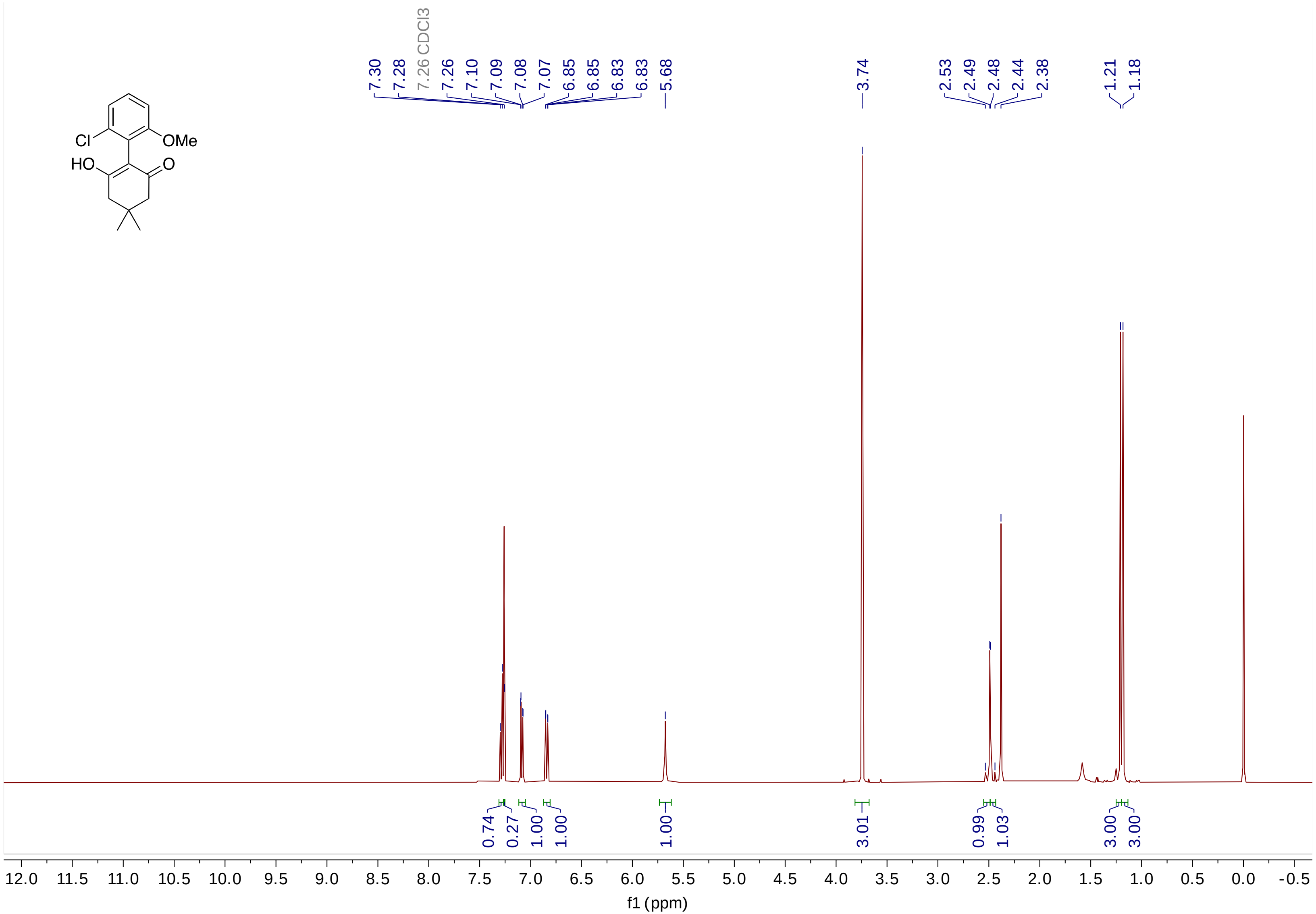

33 - $^{13}C\{^1H\}$ NMR (101 MHz, $CDCl_3$):

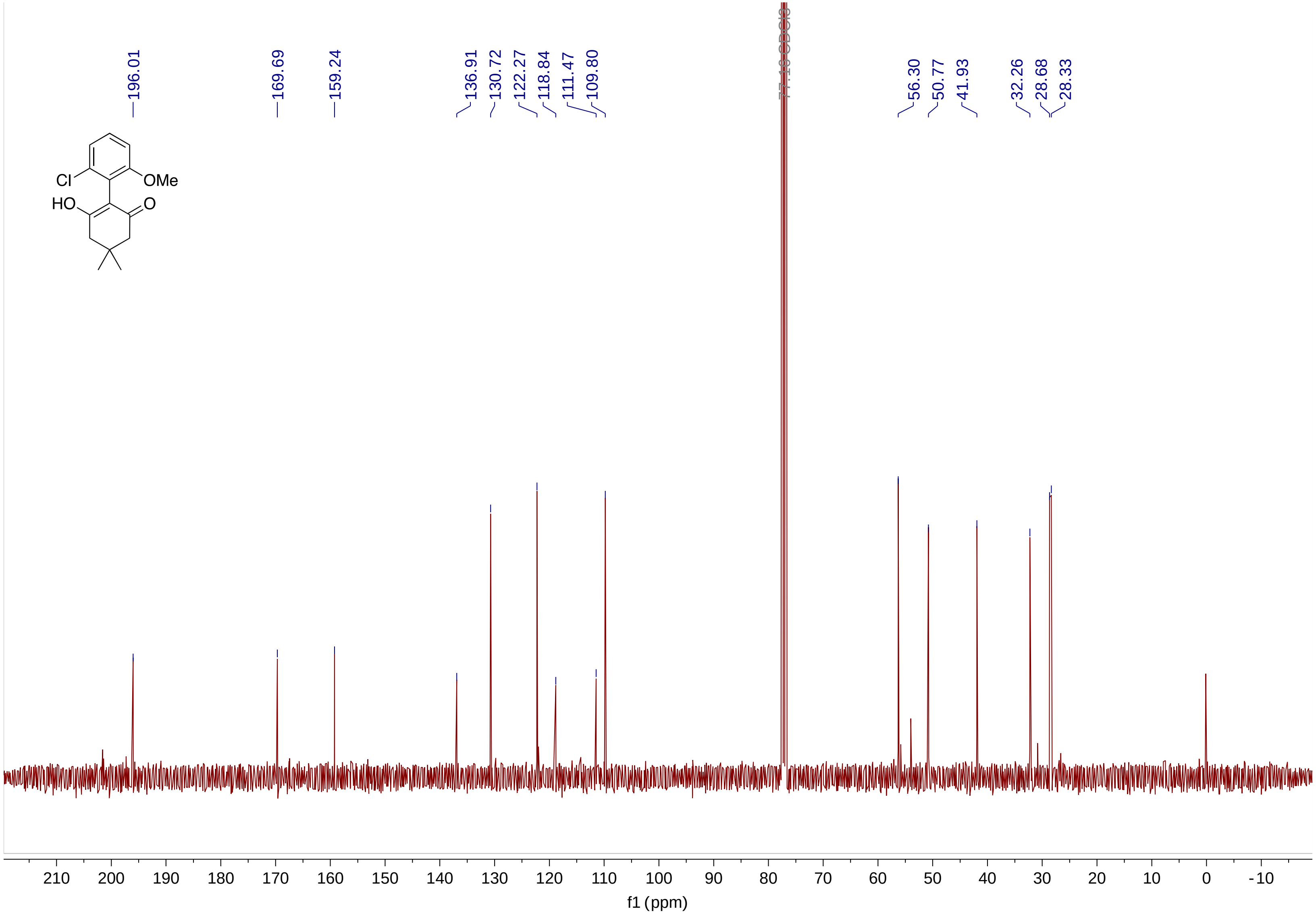

34 - ^{1}H NMR (400 MHz, $CDCl_3$):

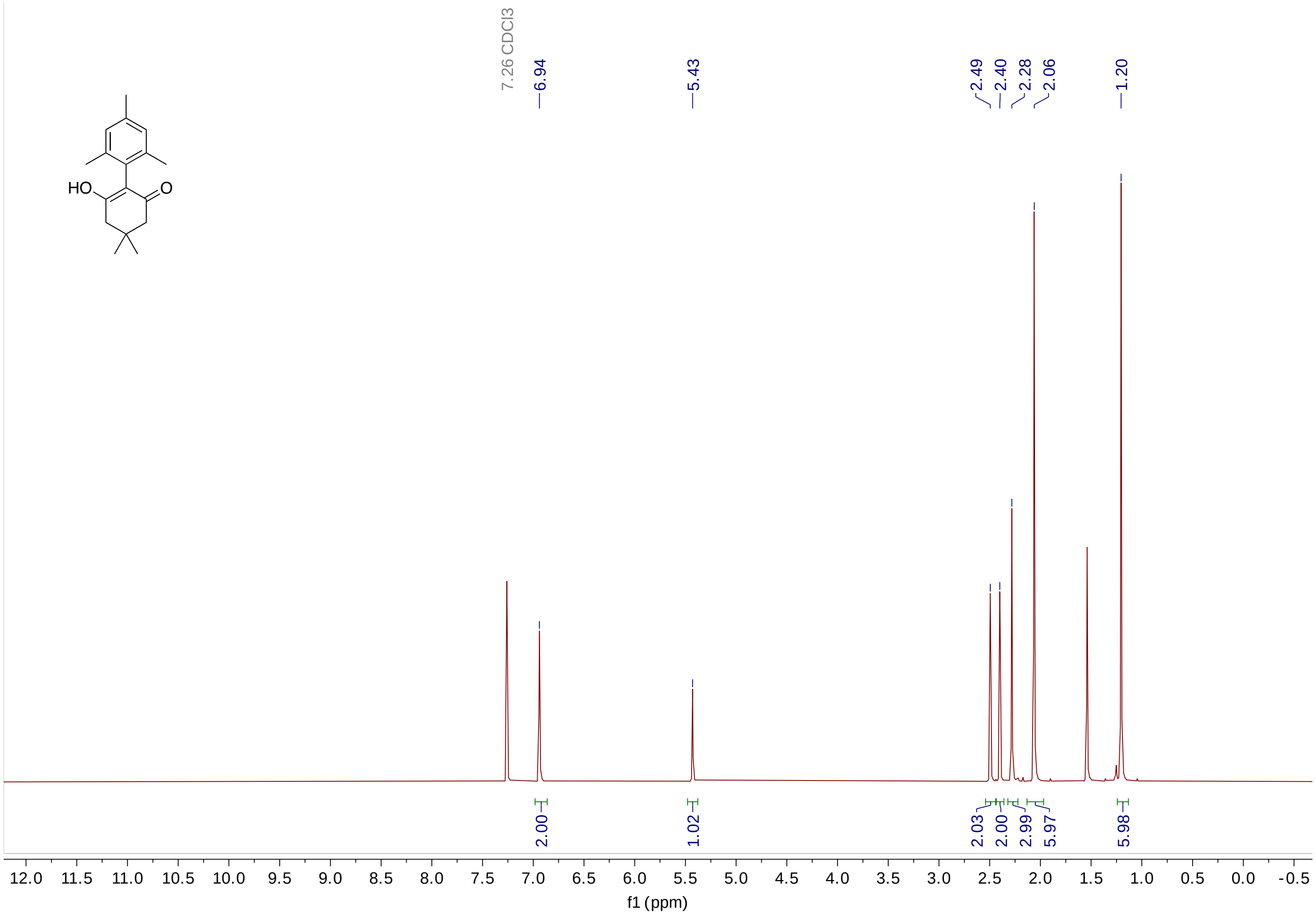

34 - $^{13}C\{^{1}H\}$ NMR (126 MHz, $CDCl_3$):

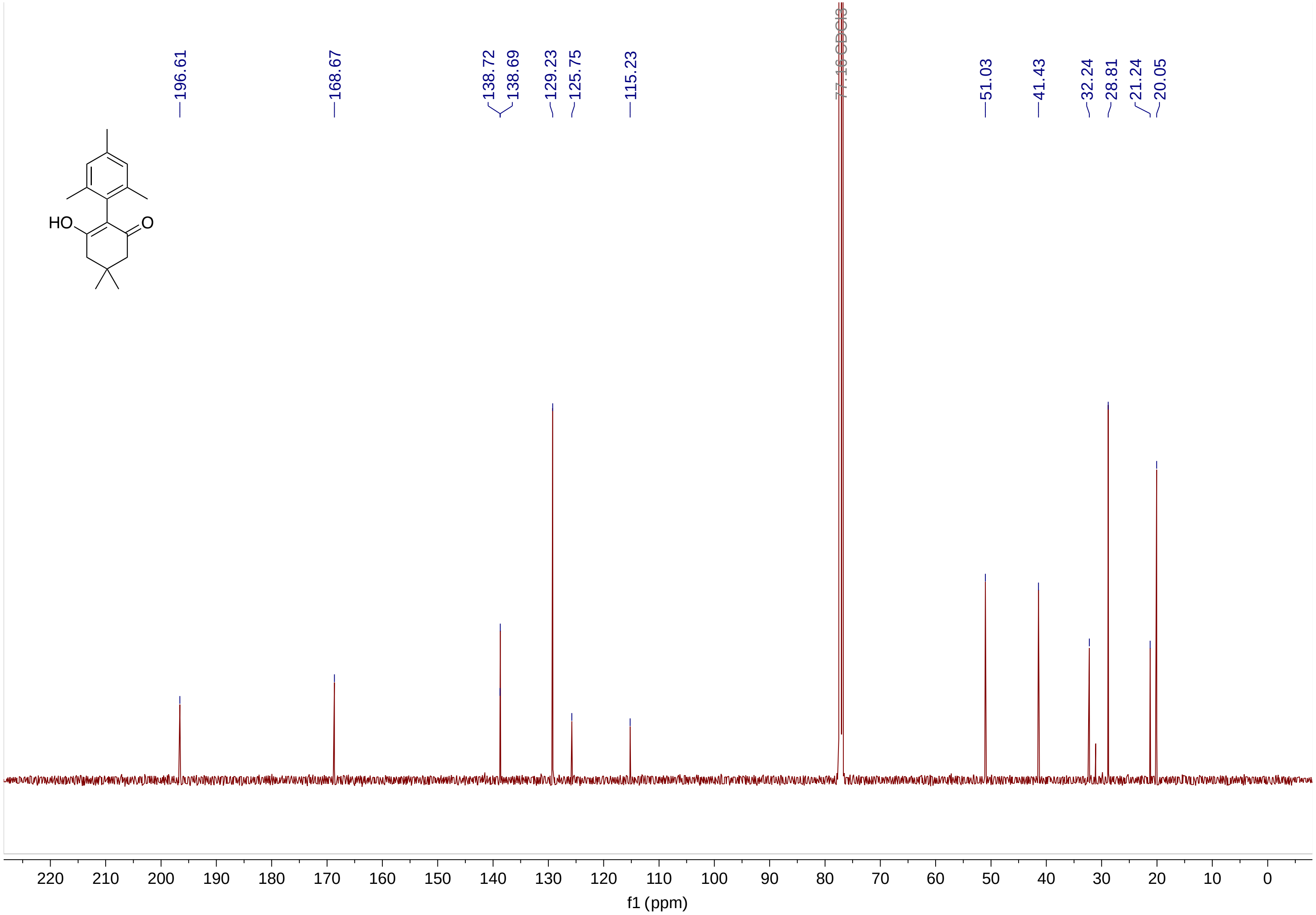

35 - ^{1}H NMR (400 MHz, CDCl$_3$):

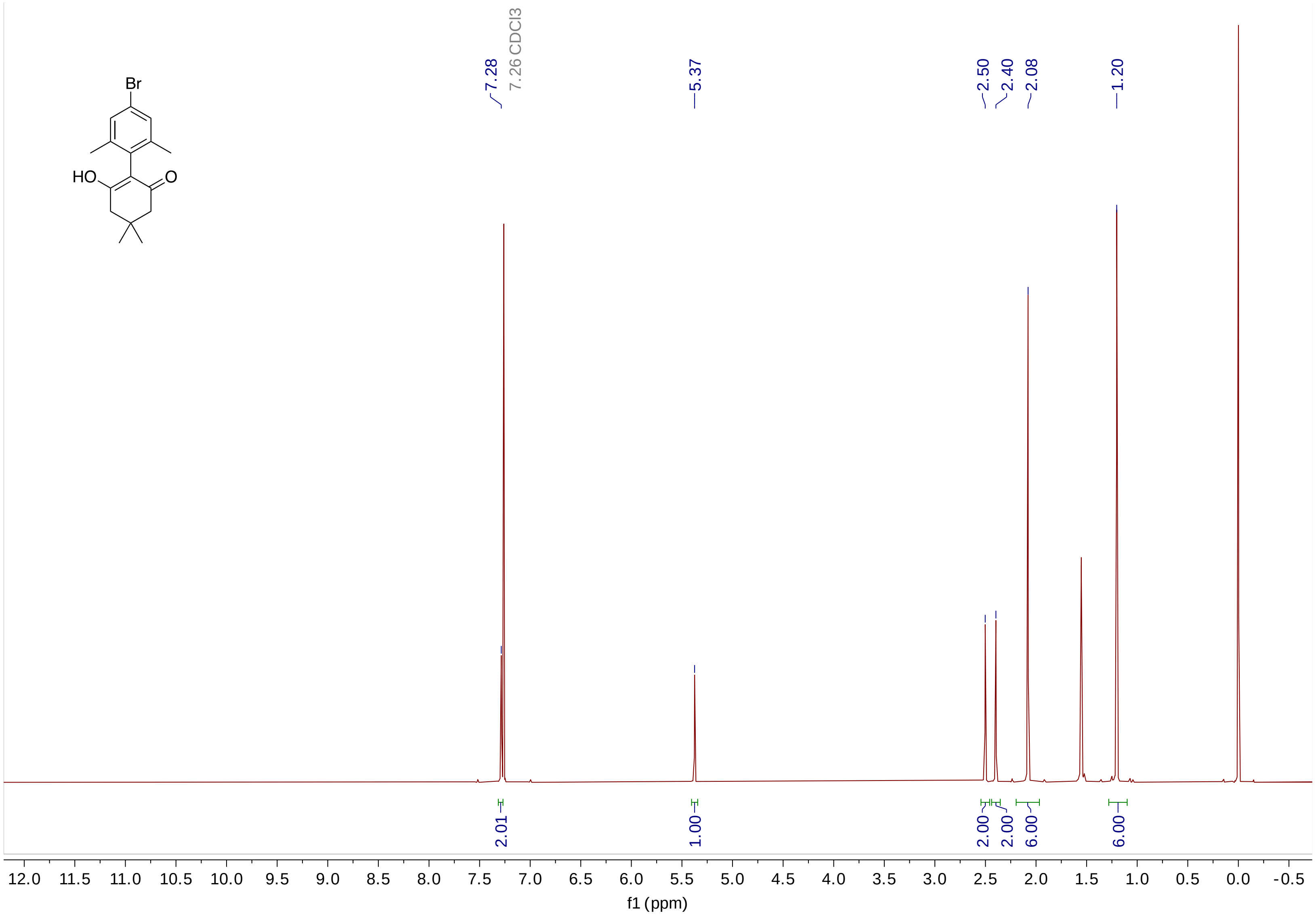

35 - $^{13}C\{^{1}H\}$ NMR (126 MHz, $CDCl_3$):

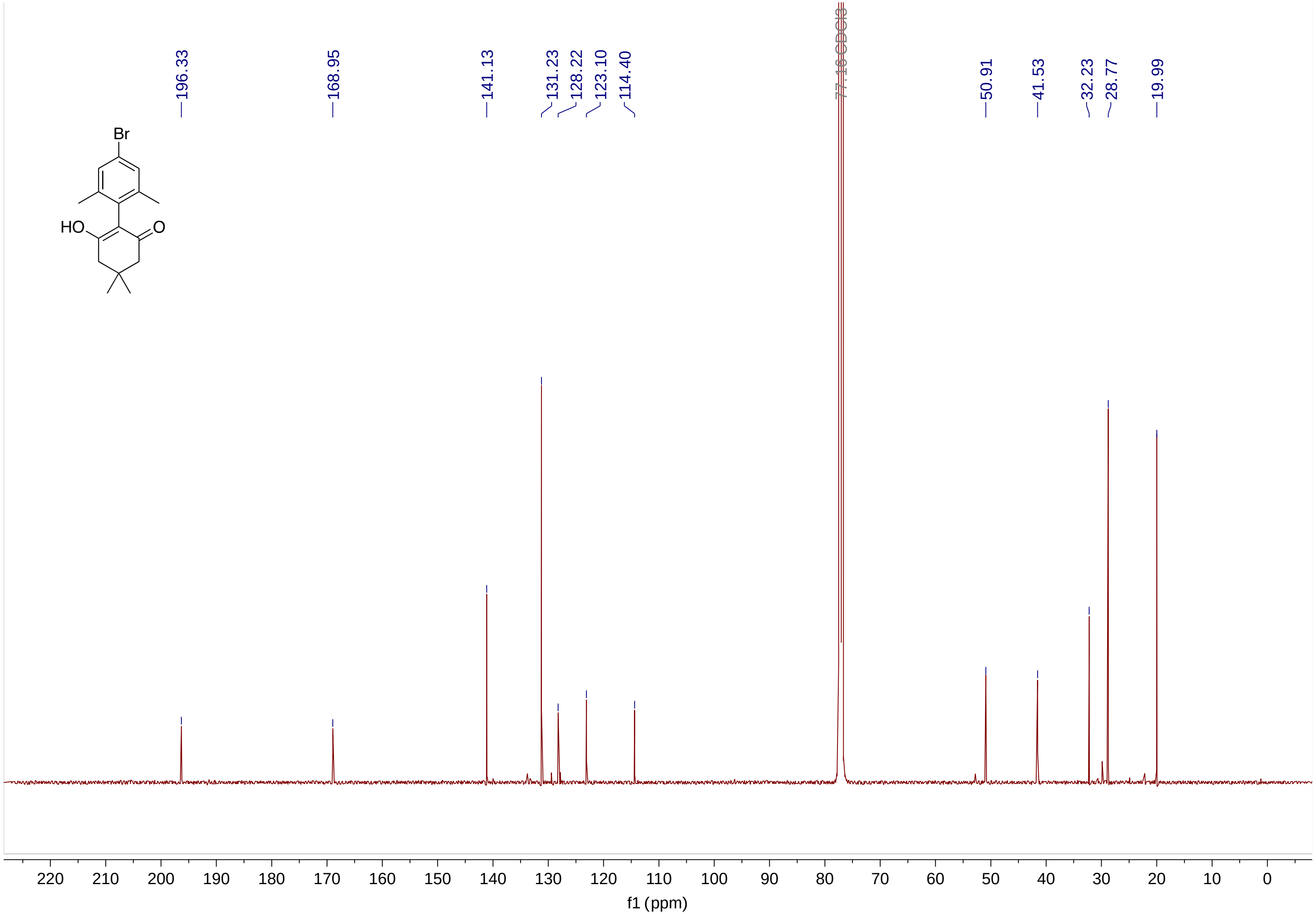

36 - ^{1}H NMR (400 MHz, $CDCl_3$):

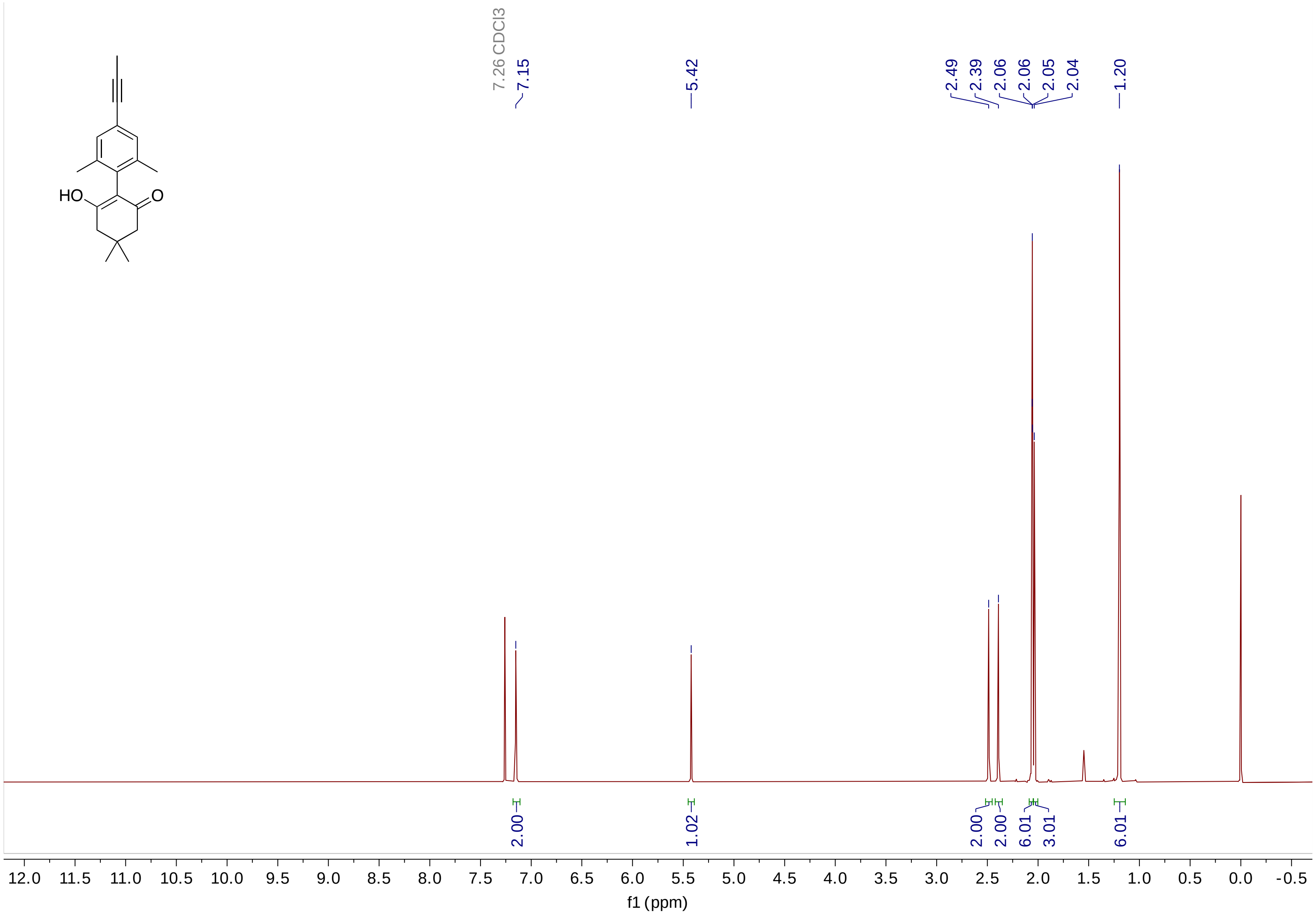

36 - $^{13}C\{^1H\}$ NMR (101 MHz, $CDCl_3$):

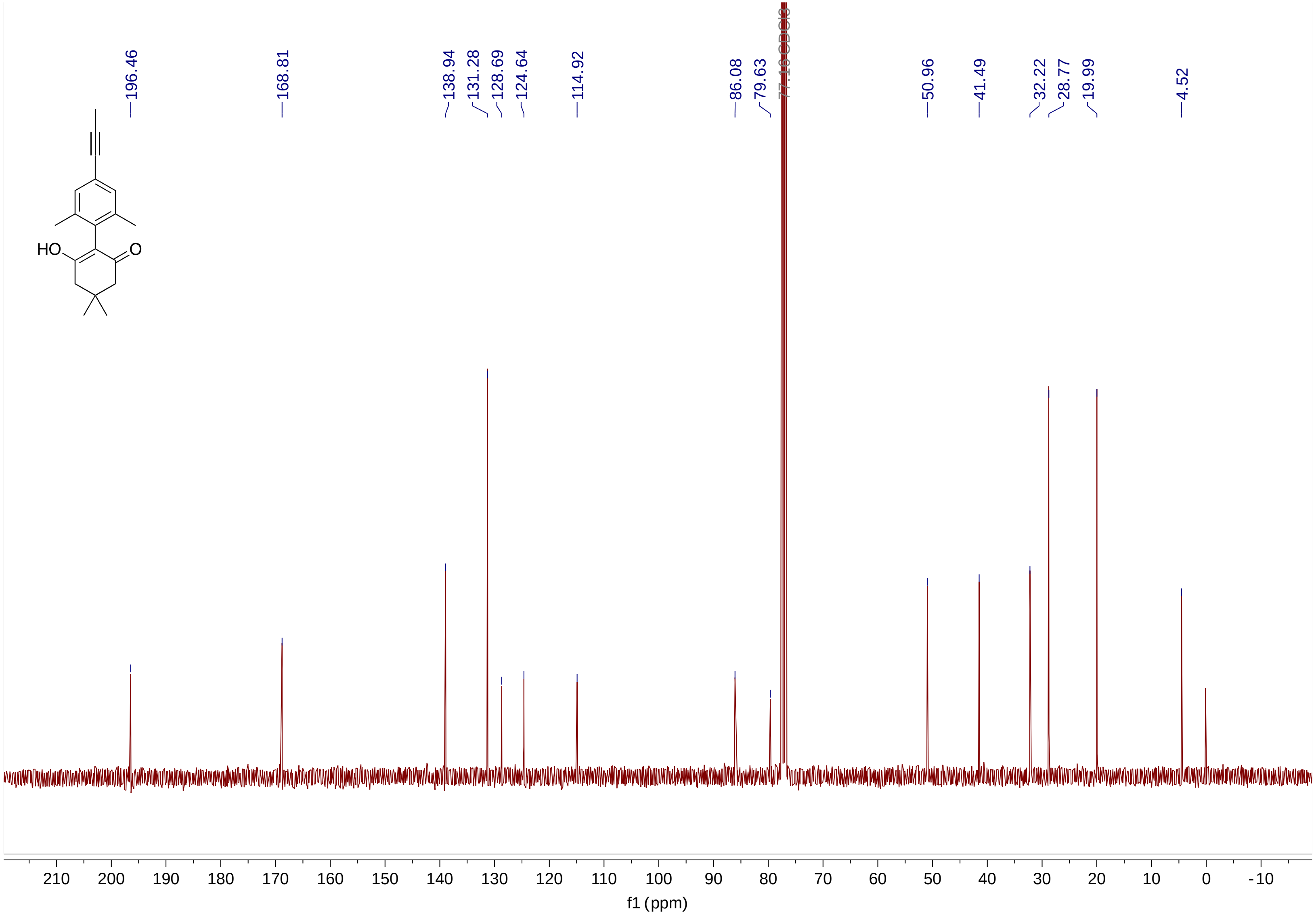

37 - ^{1}H NMR (400 MHz, $CDCl_3$):

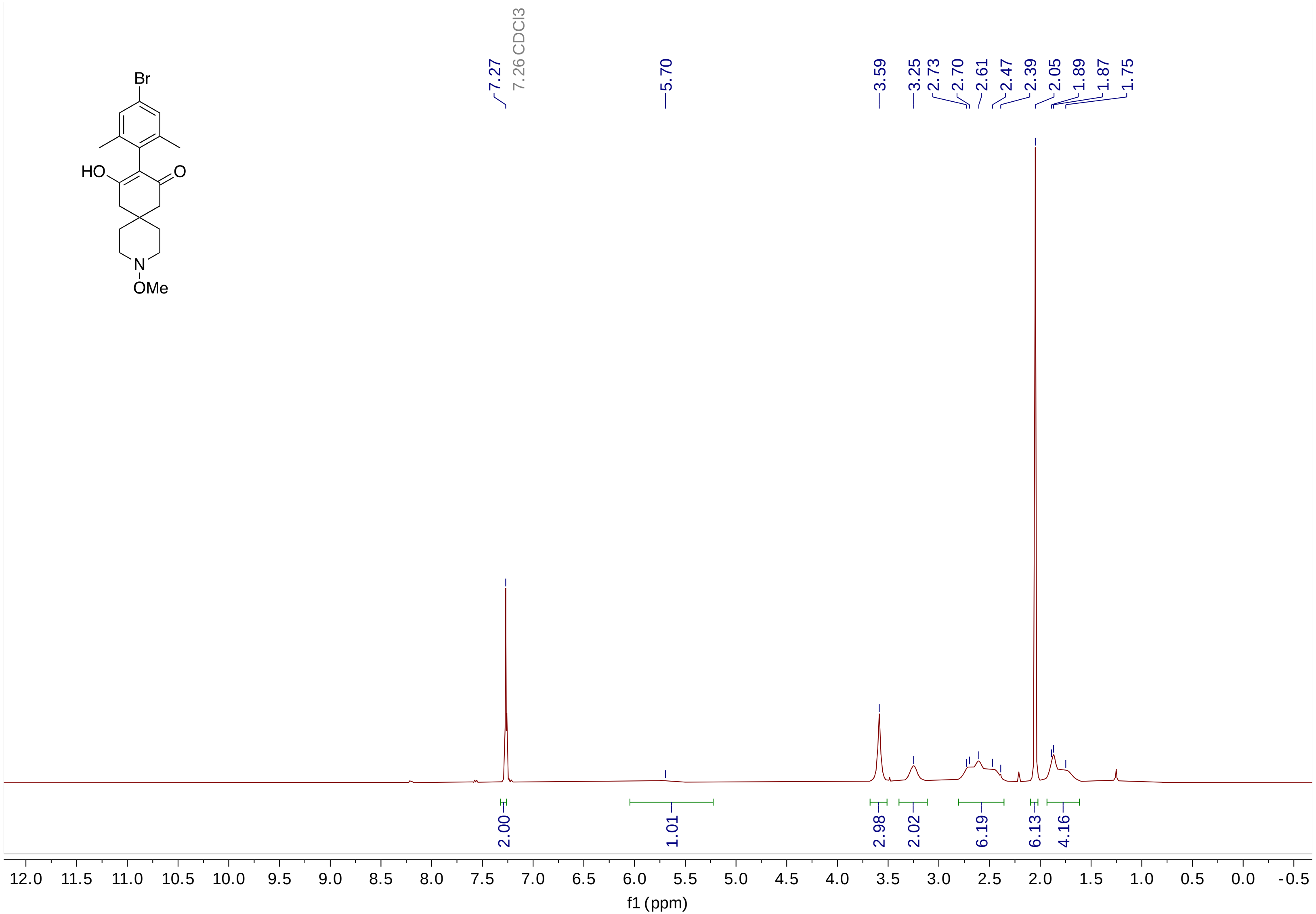

37 - $^{13}C\{^1H\}$ NMR (126 MHz, $CDCl_3$):

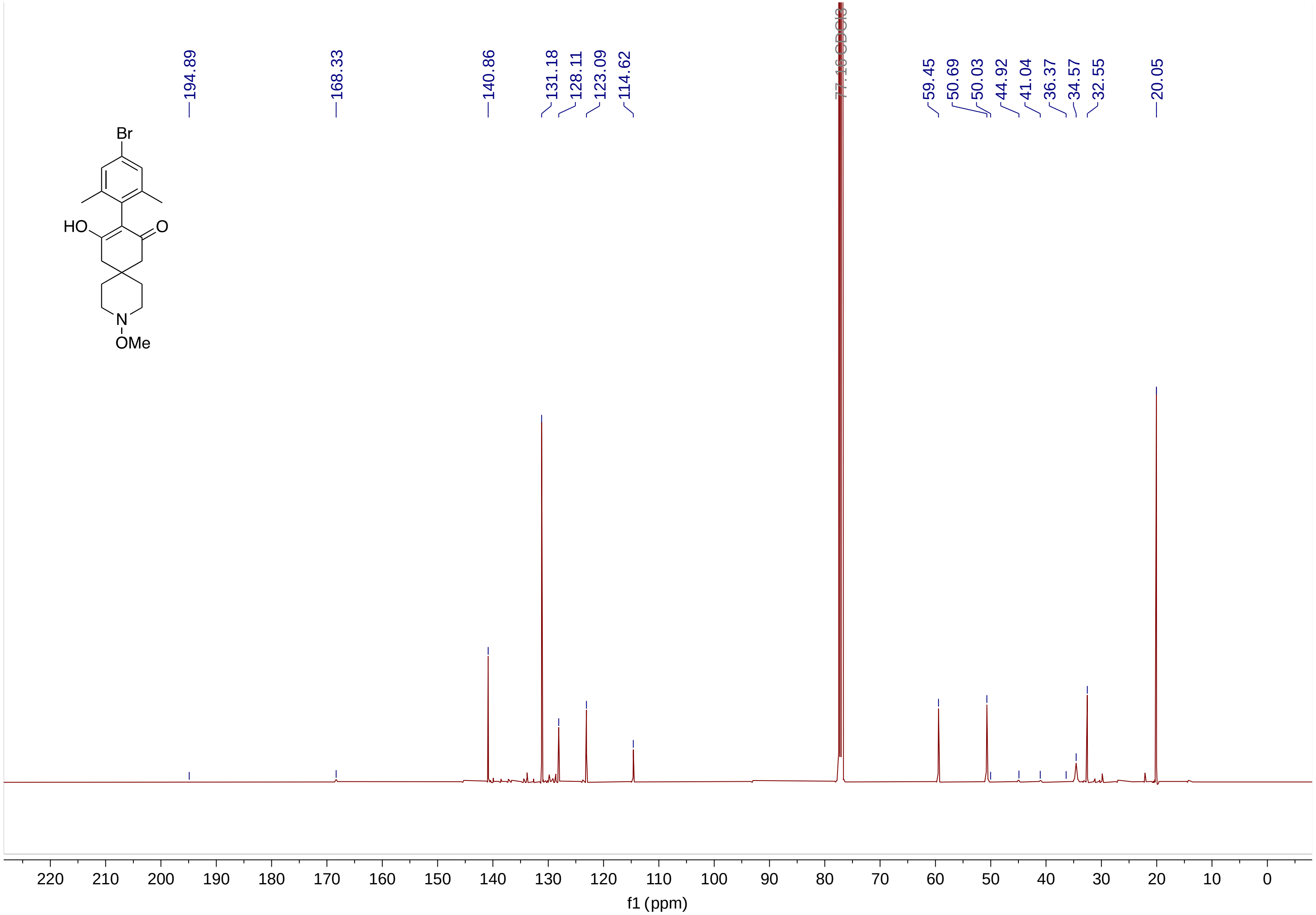

38 - ^{1}H NMR (500 MHz, CDCl$_3$):

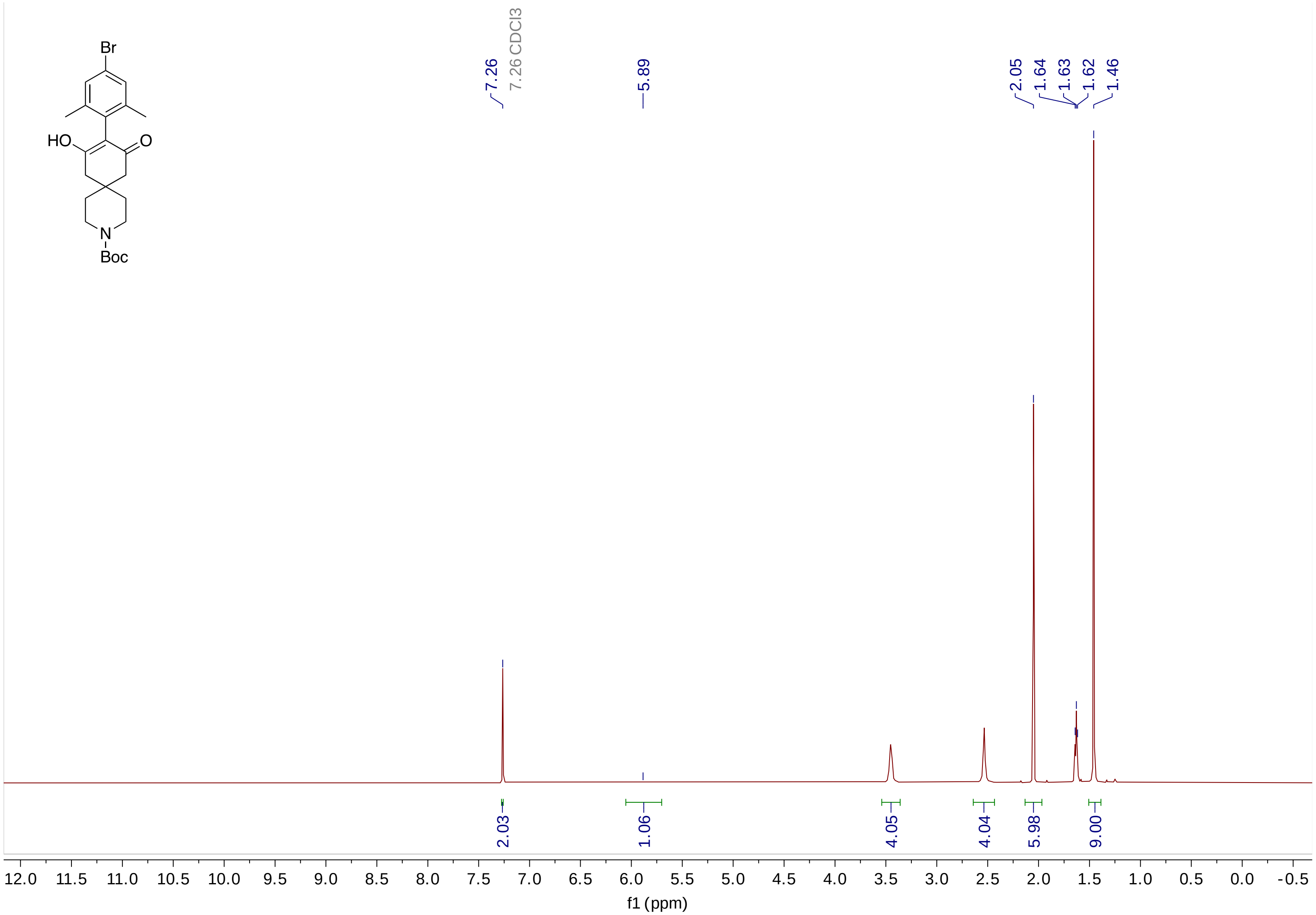

38 - $^{13}C\{^{1}H\}$ NMR (126 MHz, $CDCl_3$):

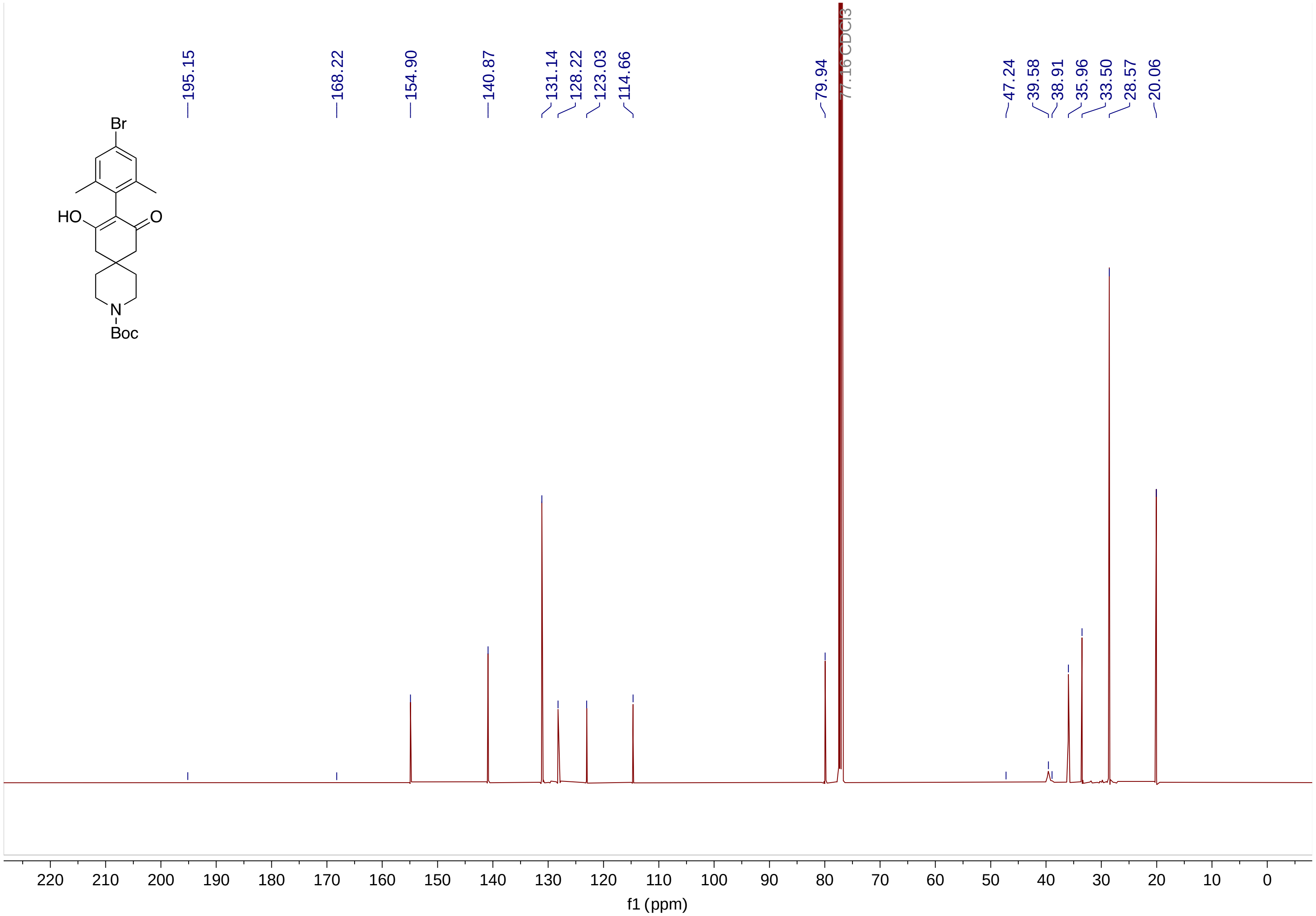

39 - ^{1}H NMR (400 MHz, CDCl₃):

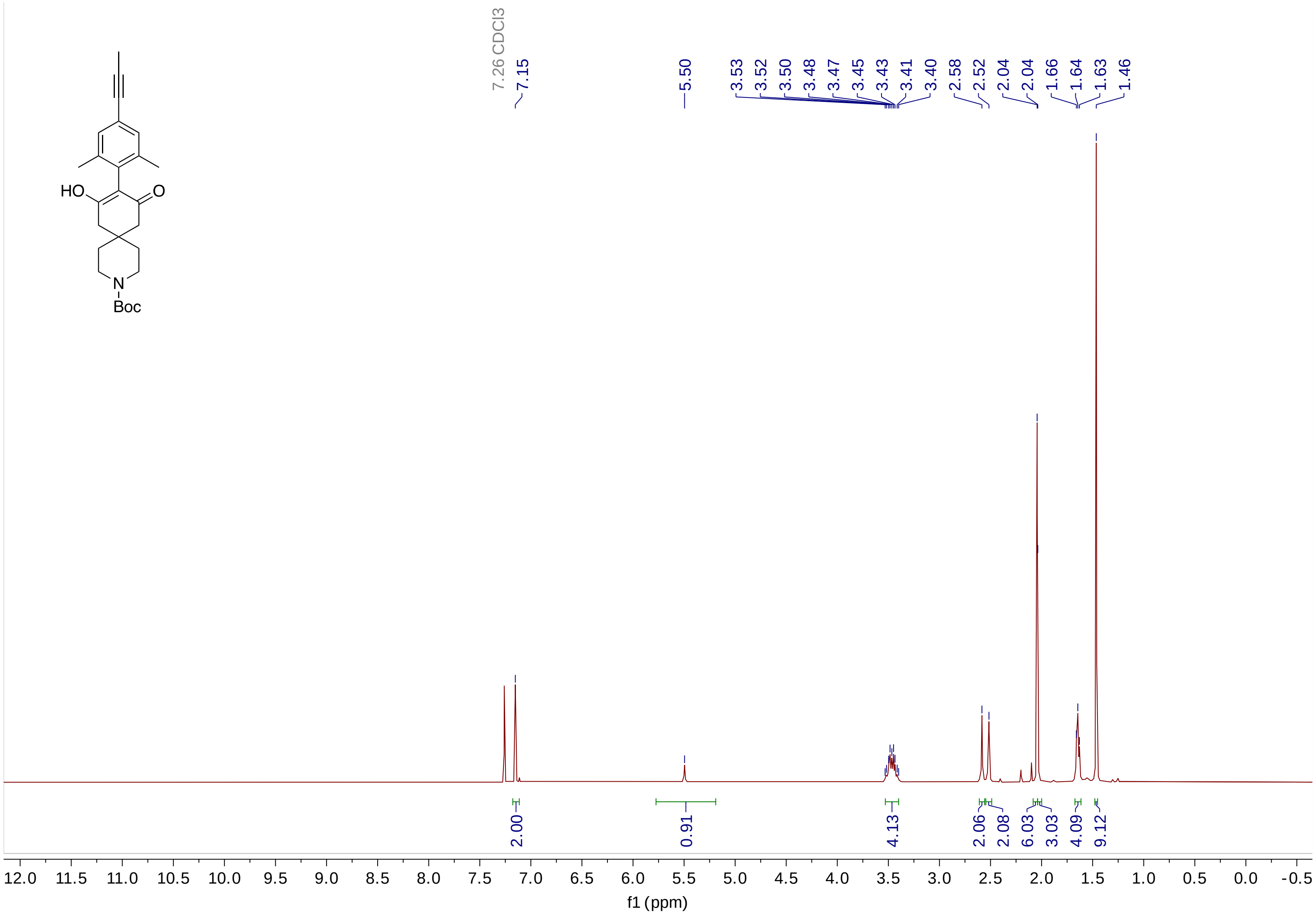

39 - $^{13}C\{^1H\}$ NMR (126 MHz, $CDCl_3$):

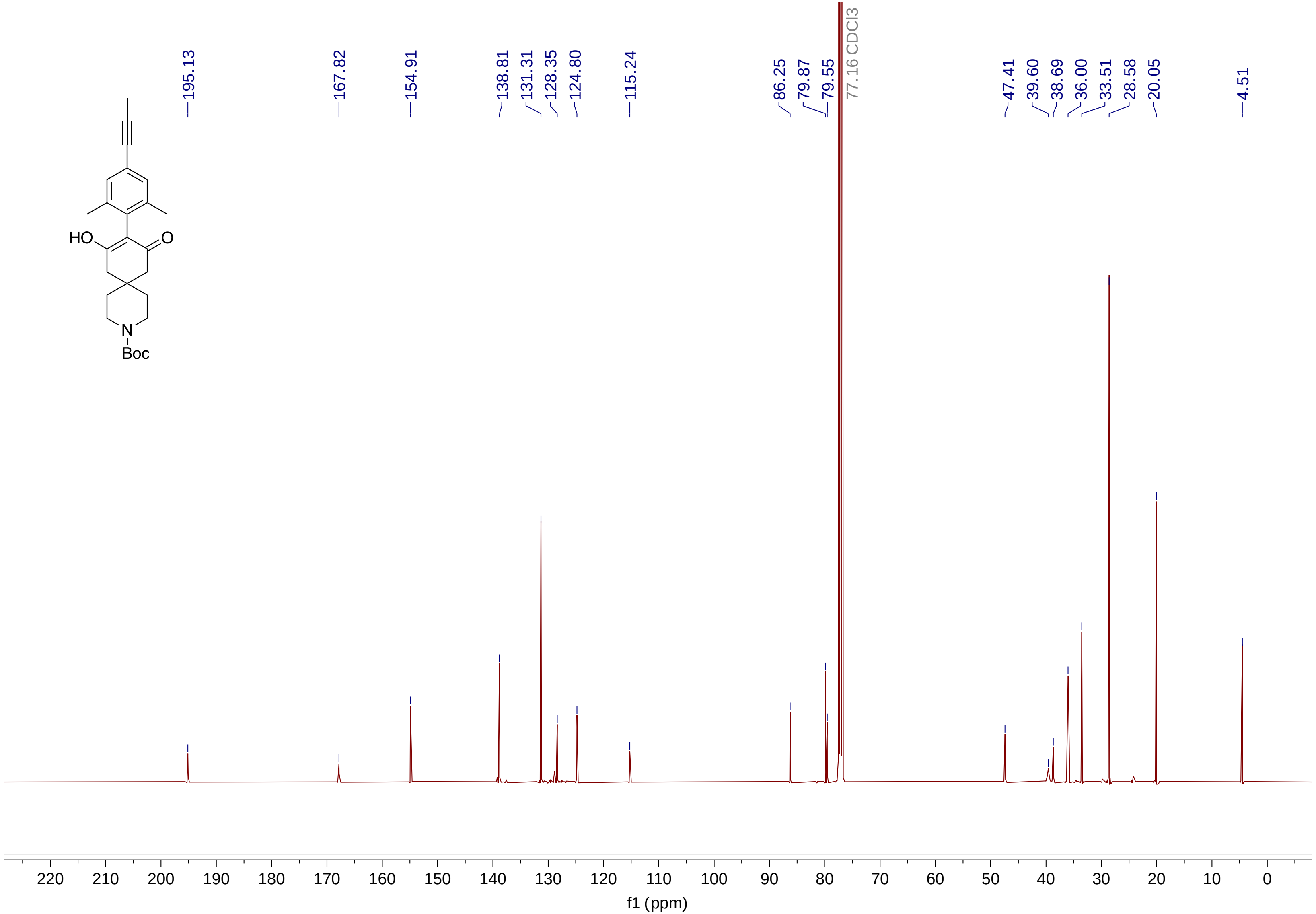

40 - ^{1}H NMR (400 MHz, $CDCl_3$):

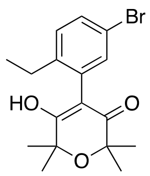

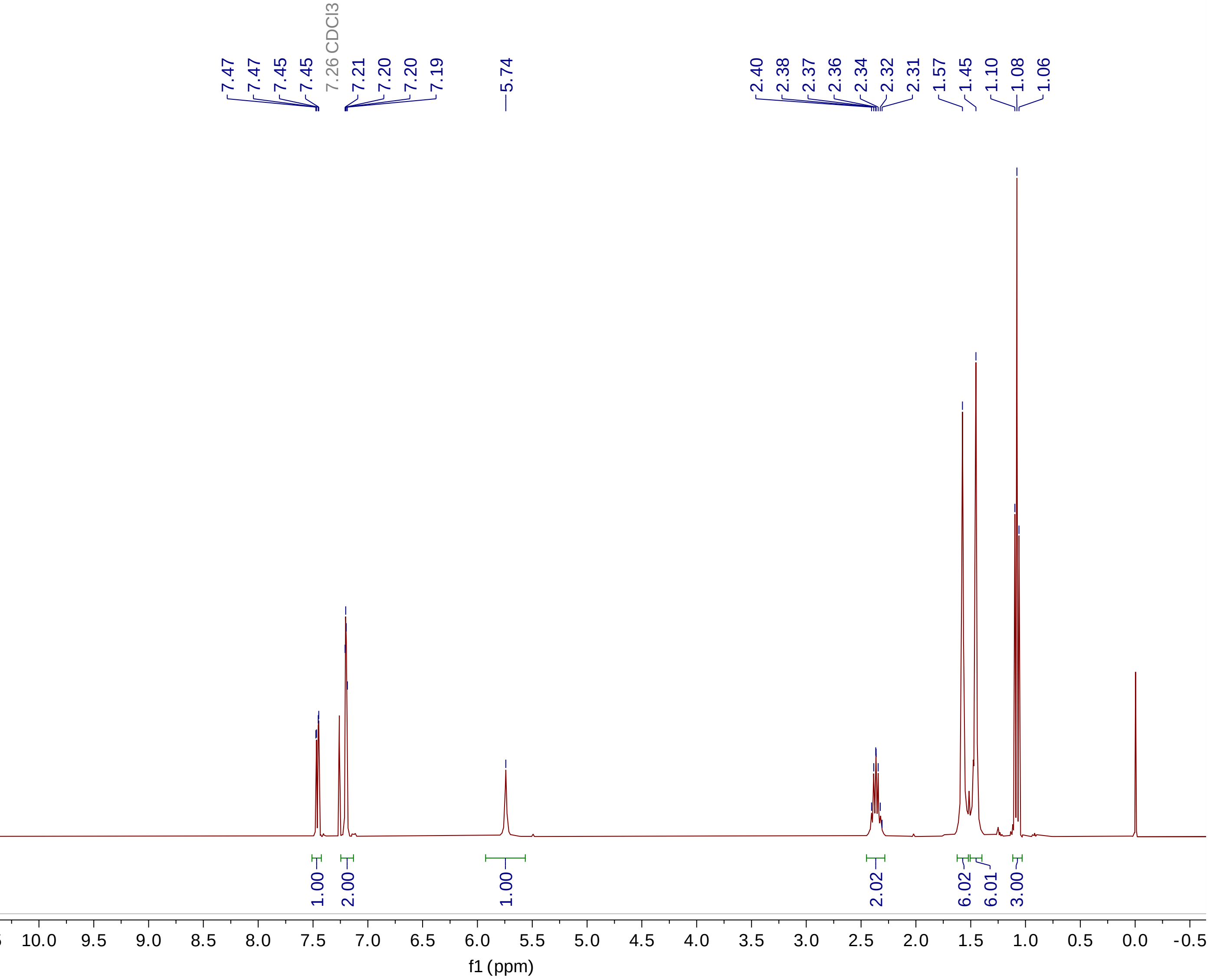

40 - $^{13}C\{^1H\}$ NMR (101 MHz, $CDCl_3$):

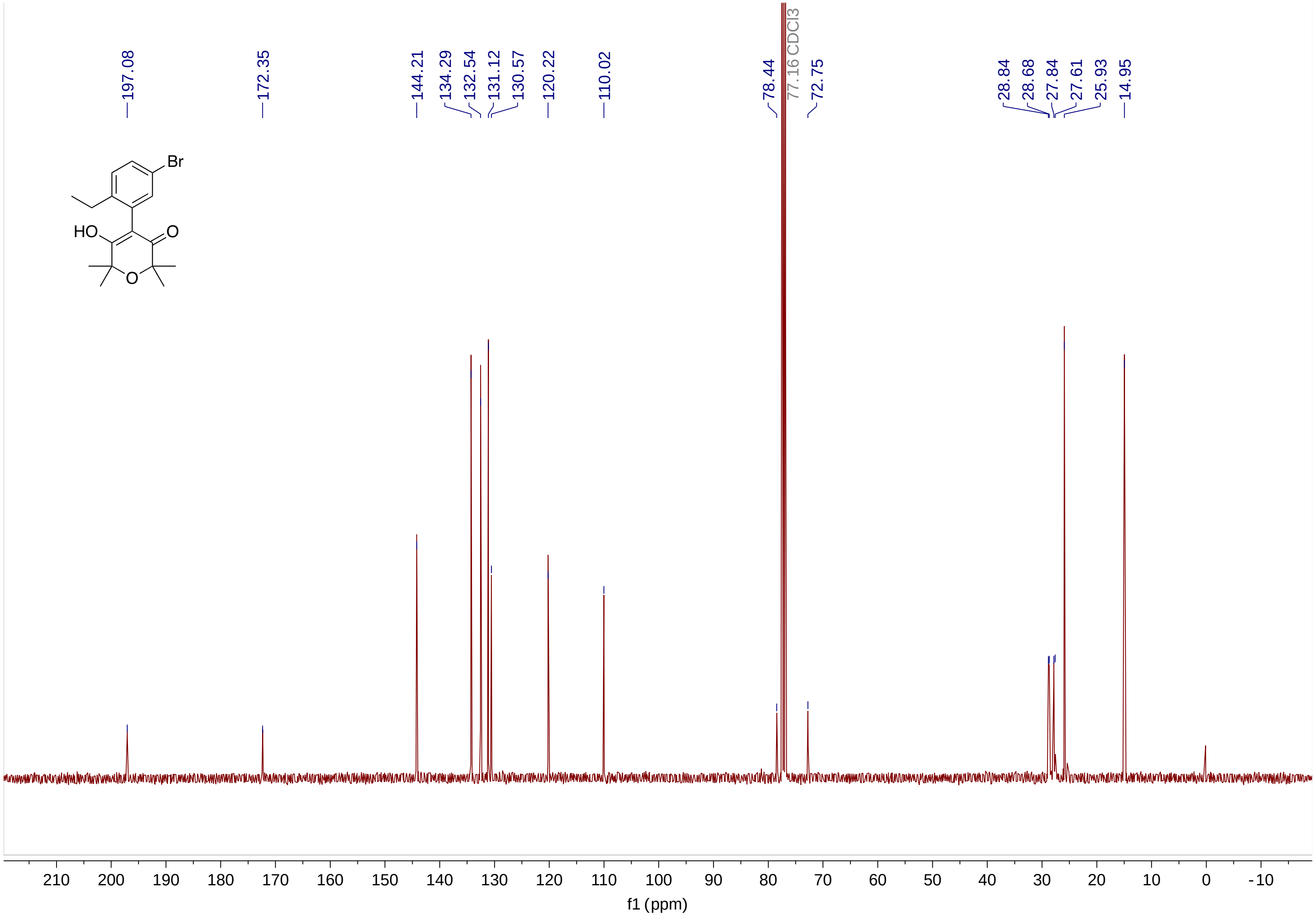

41 - ^{1}H NMR (400 MHz, CDCl$_3$):

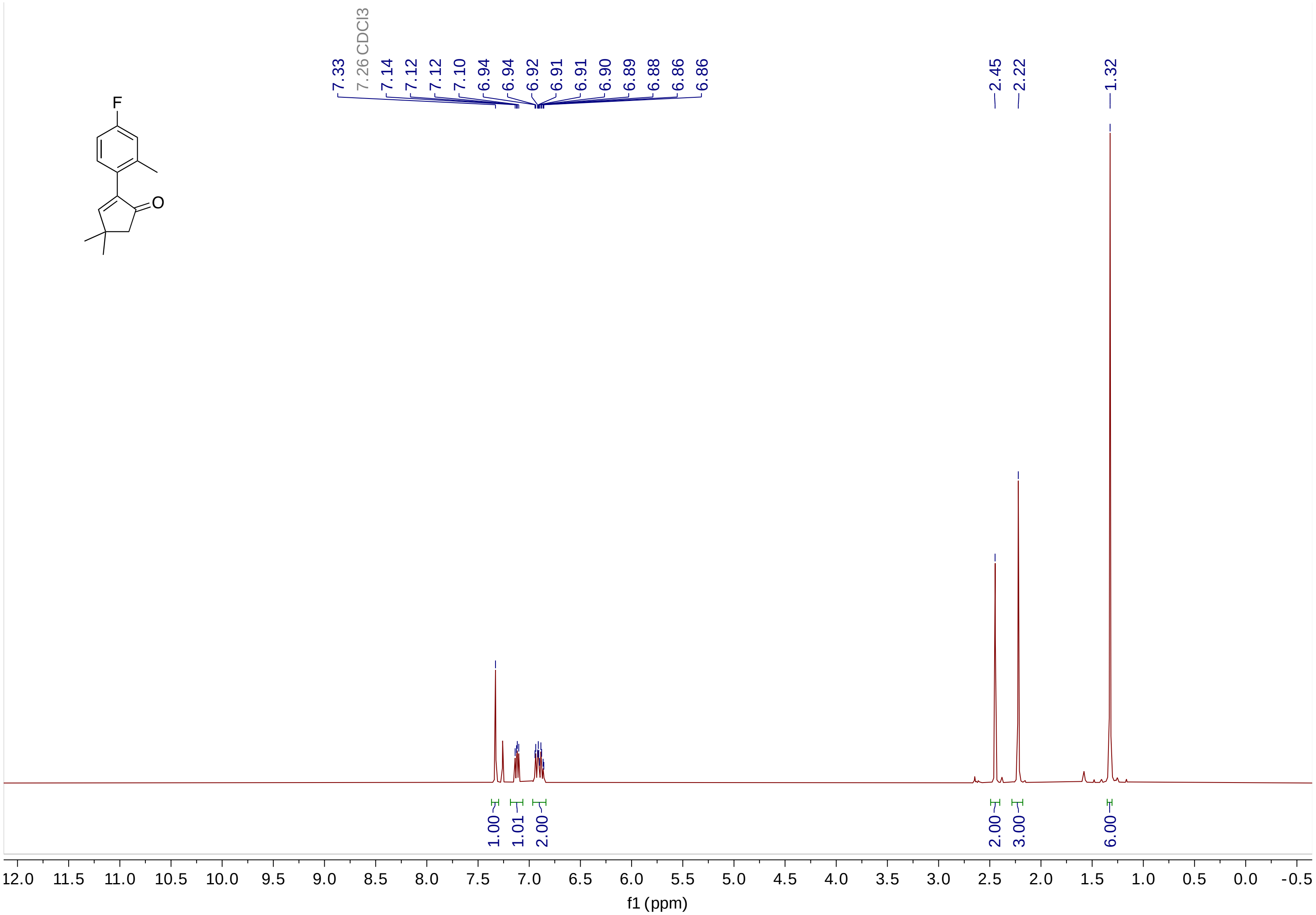

41 - $^{13}C\{^{1}H\}$ NMR (101 MHz, $CDCl_3$):

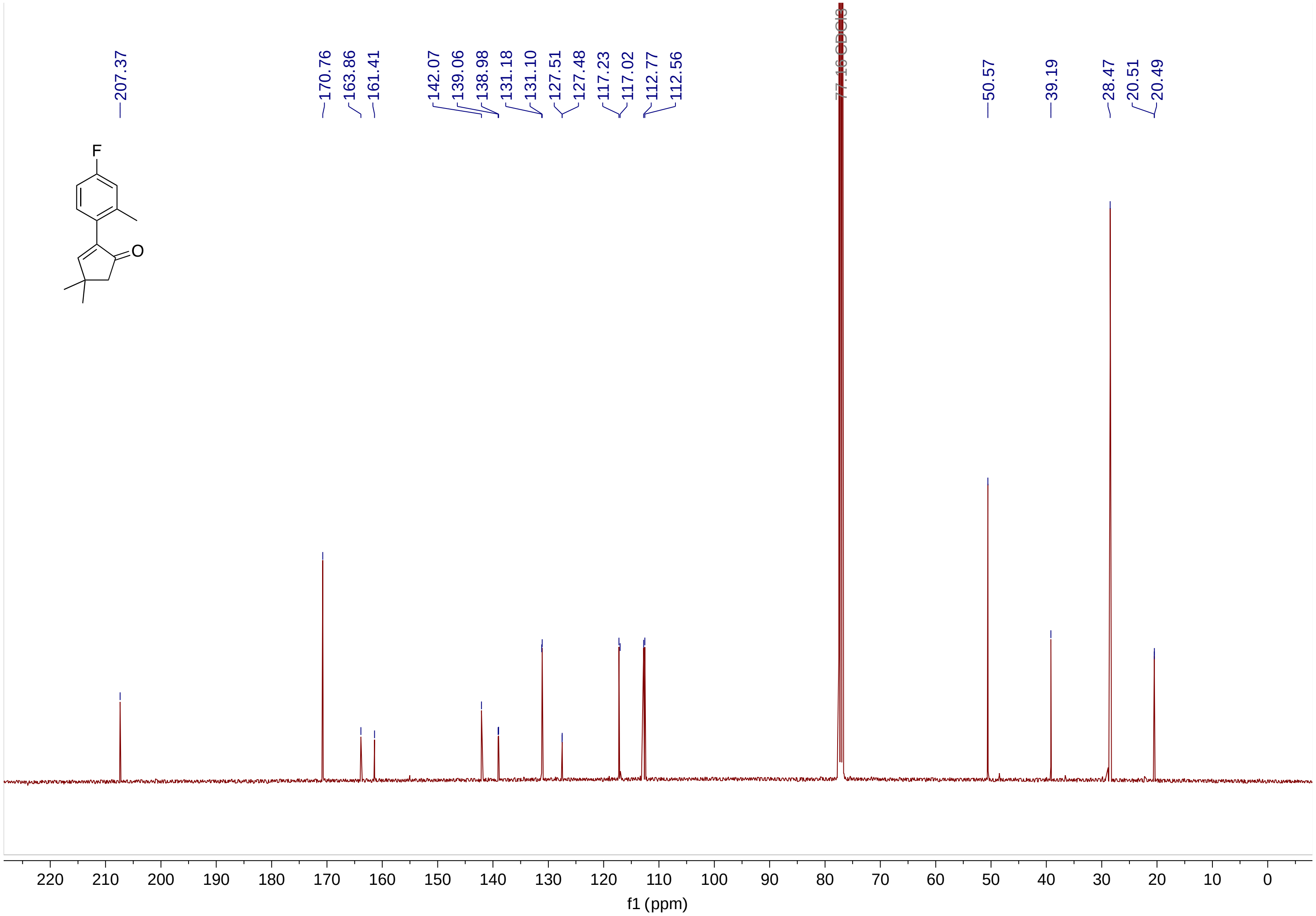

41 - ^{19}F NMR (377 MHz, $CDCl_3$):

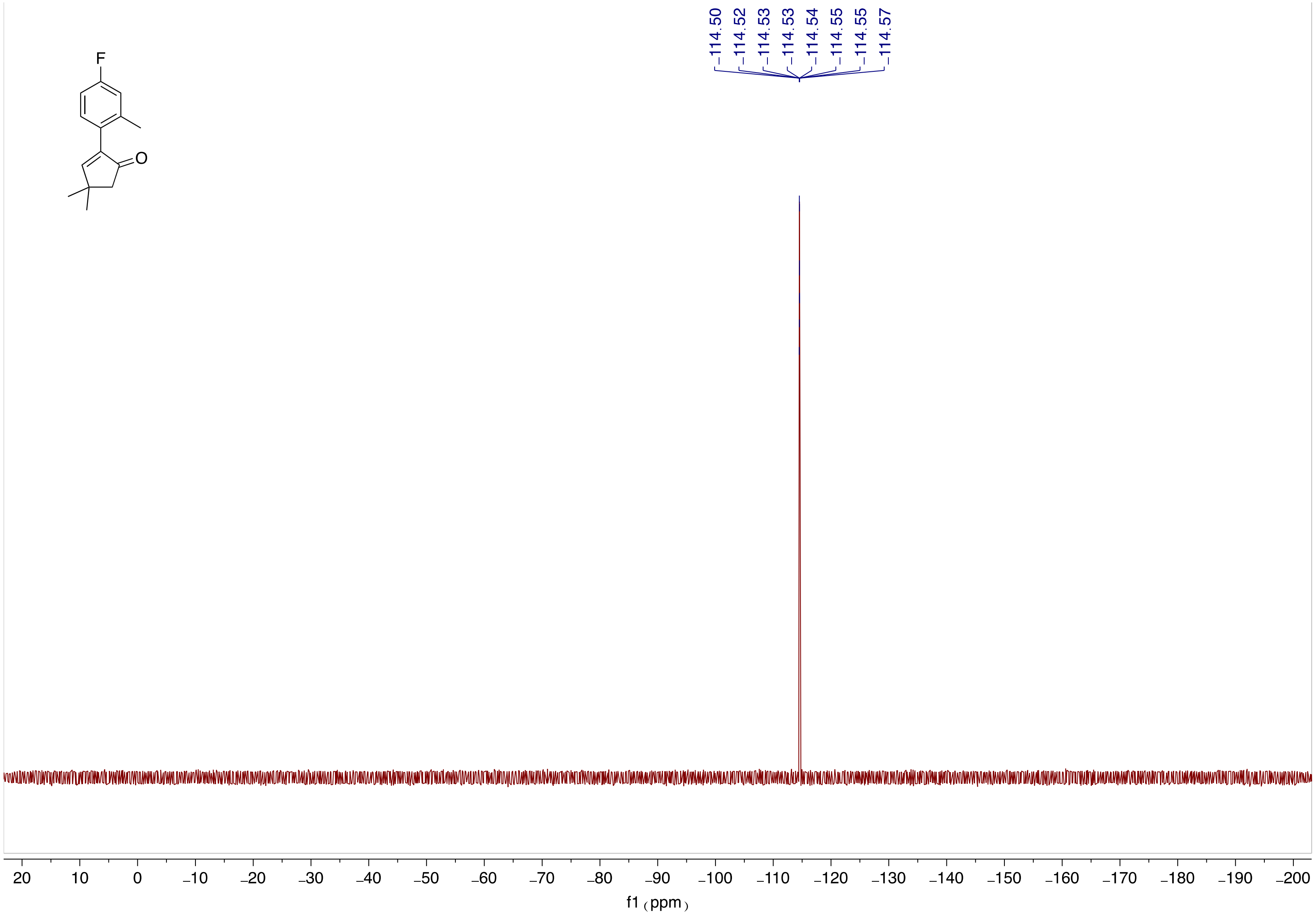

42 - ^{1}H NMR (400 MHz, $CDCl_3$):

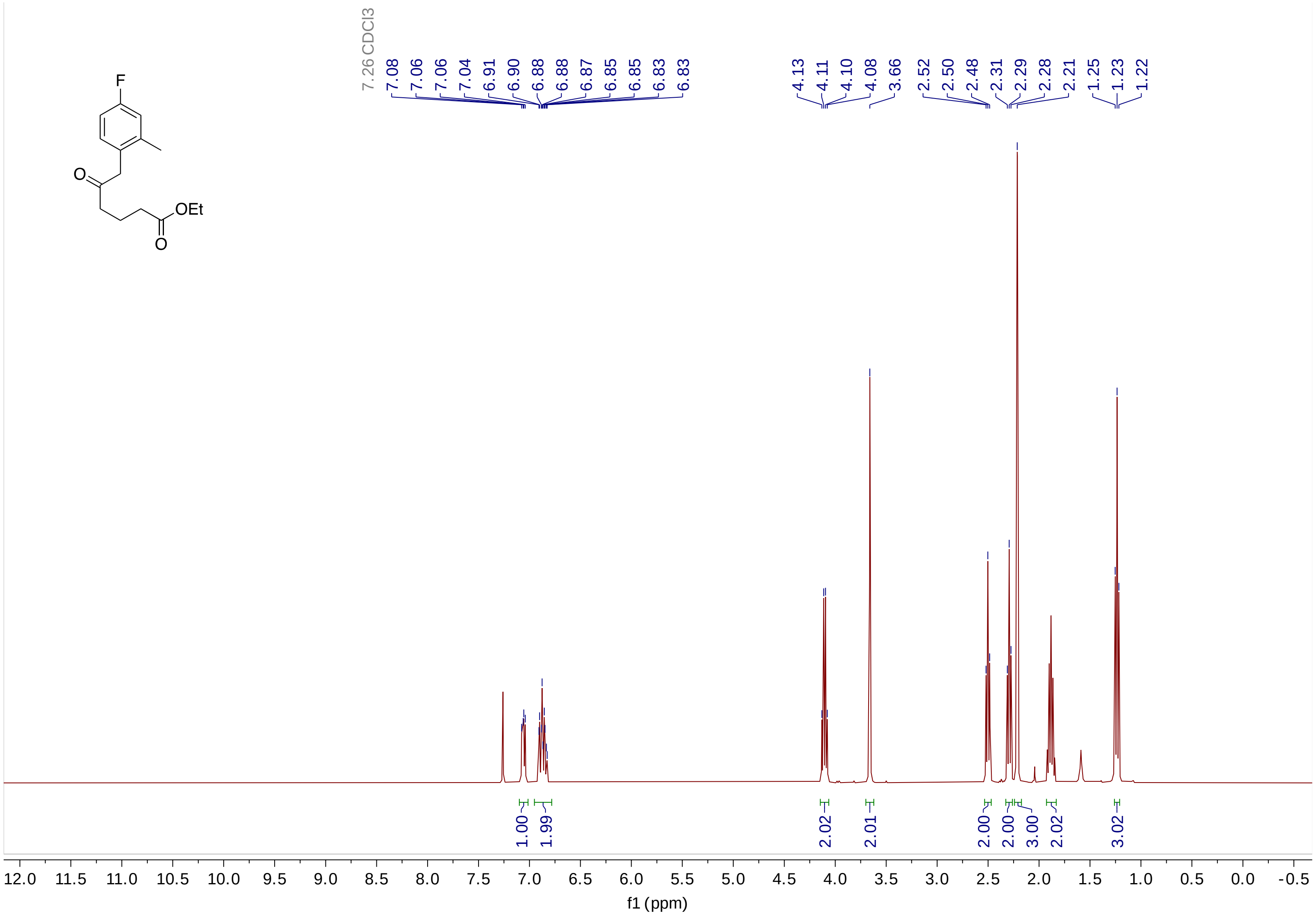

42 - $^{13}C\{^{1}H\}$ NMR (101 MHz, $CDCl_3$):

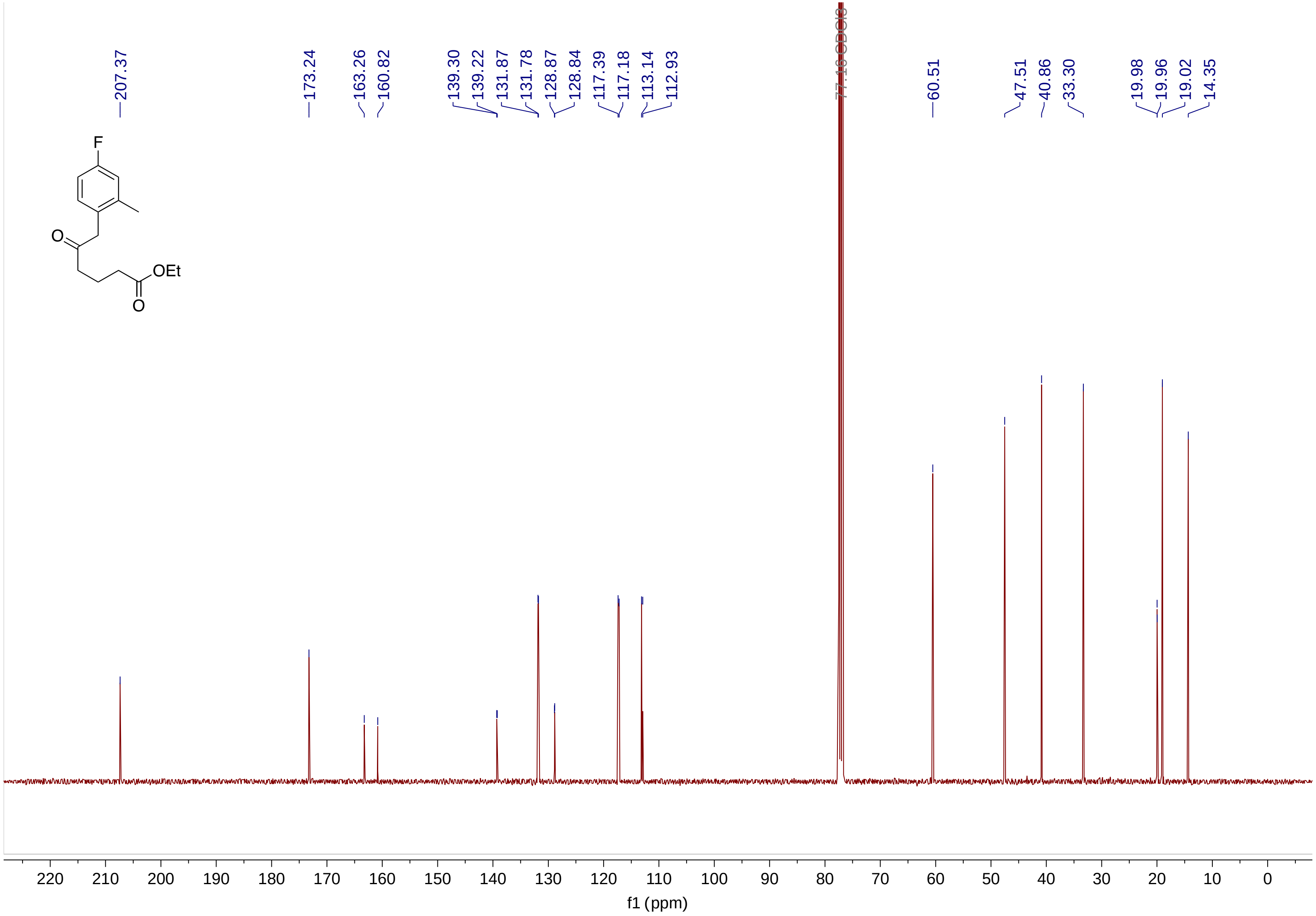

<![CDATA[**42 - ^{19}F NMR (377 MHz, $CDCl_3$):**

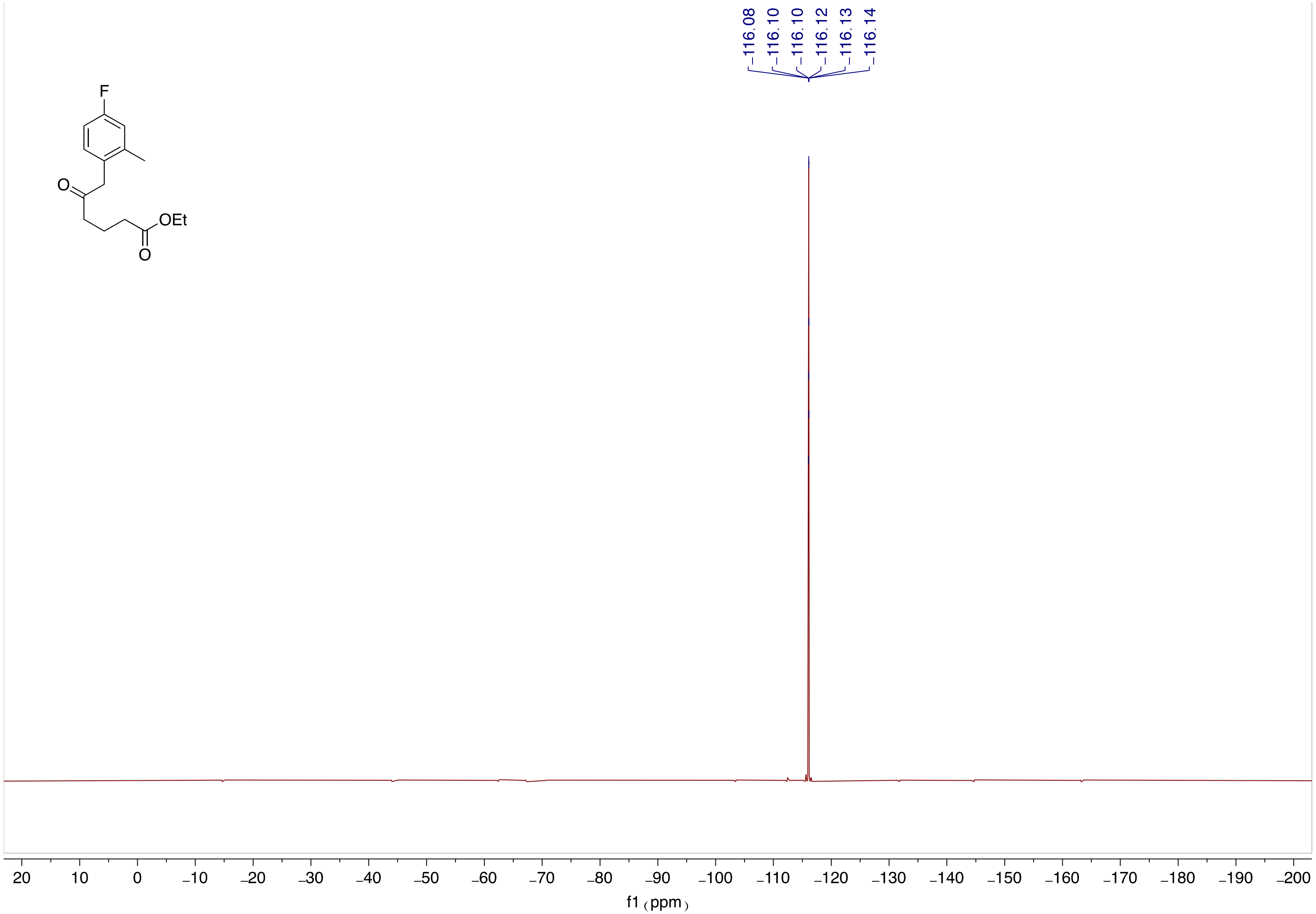

]]>

43 - ^{1}H NMR (400 MHz, $CDCl_3$):

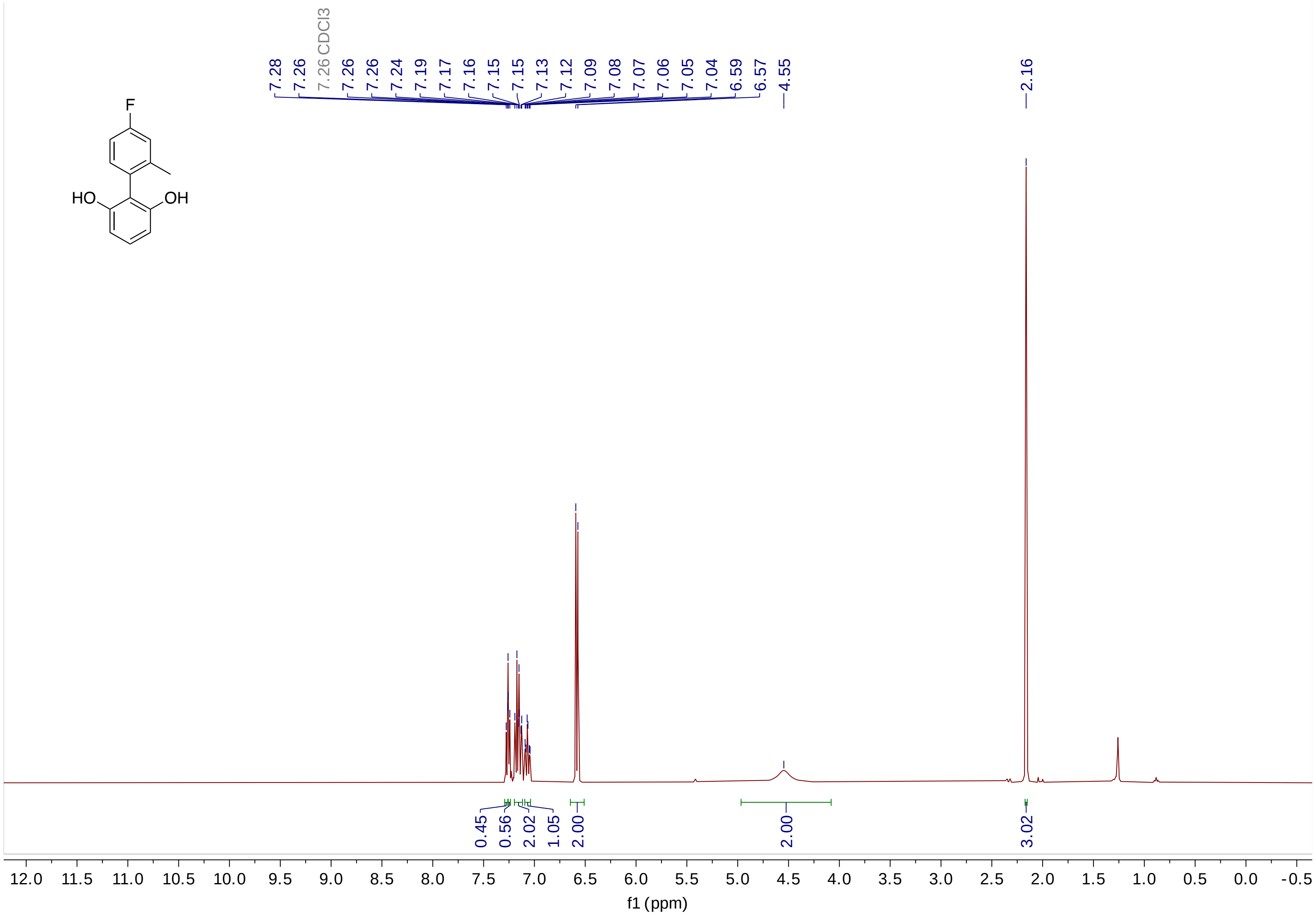

43 - $^{13}C\{^1H\}$ NMR (101 MHz, CDCl$_3$):

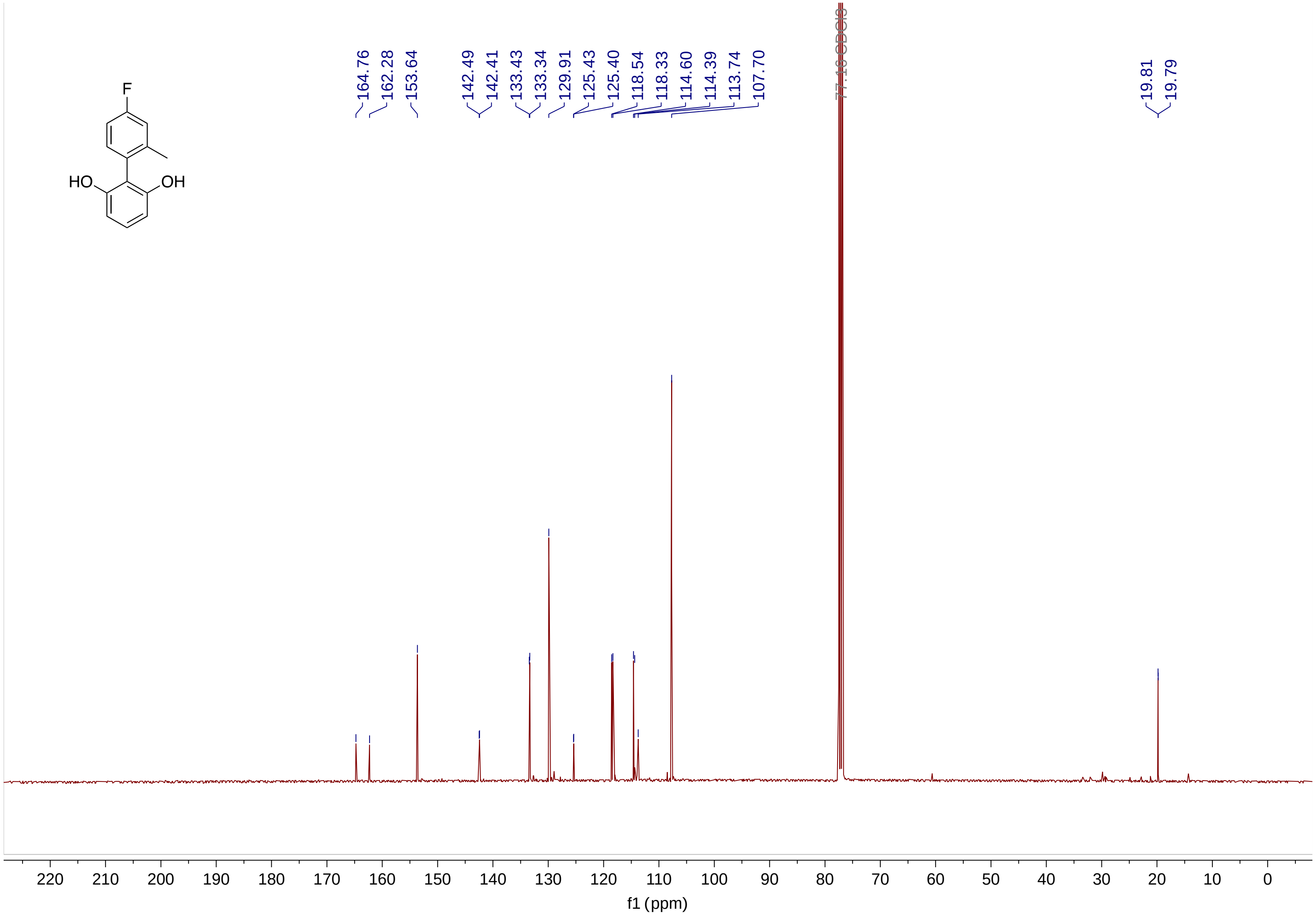

43 - ^{19}F NMR (377 MHz, $CDCl_3$):

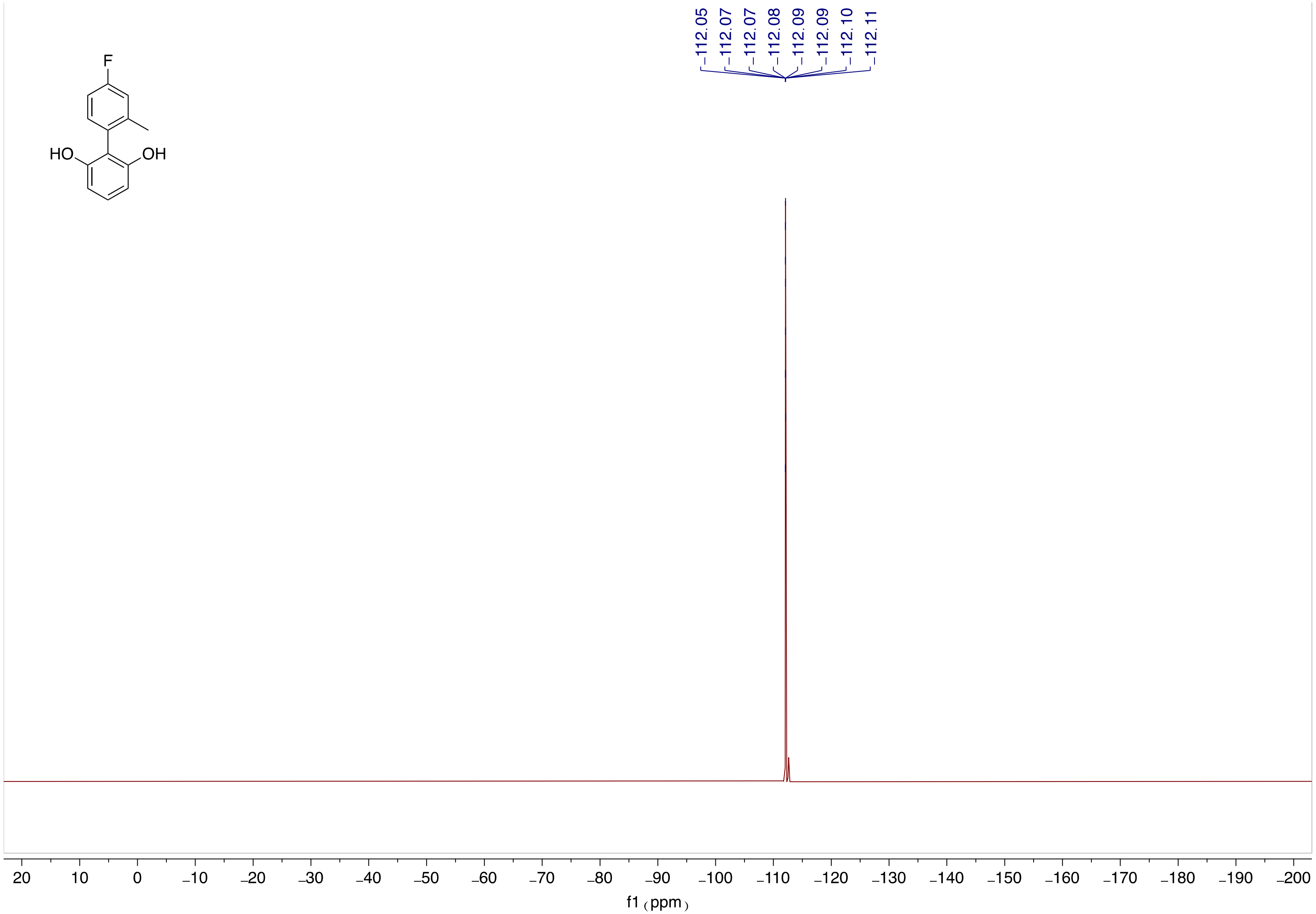

45 - ^{1}H NMR (400 MHz, CDCl$_3$):

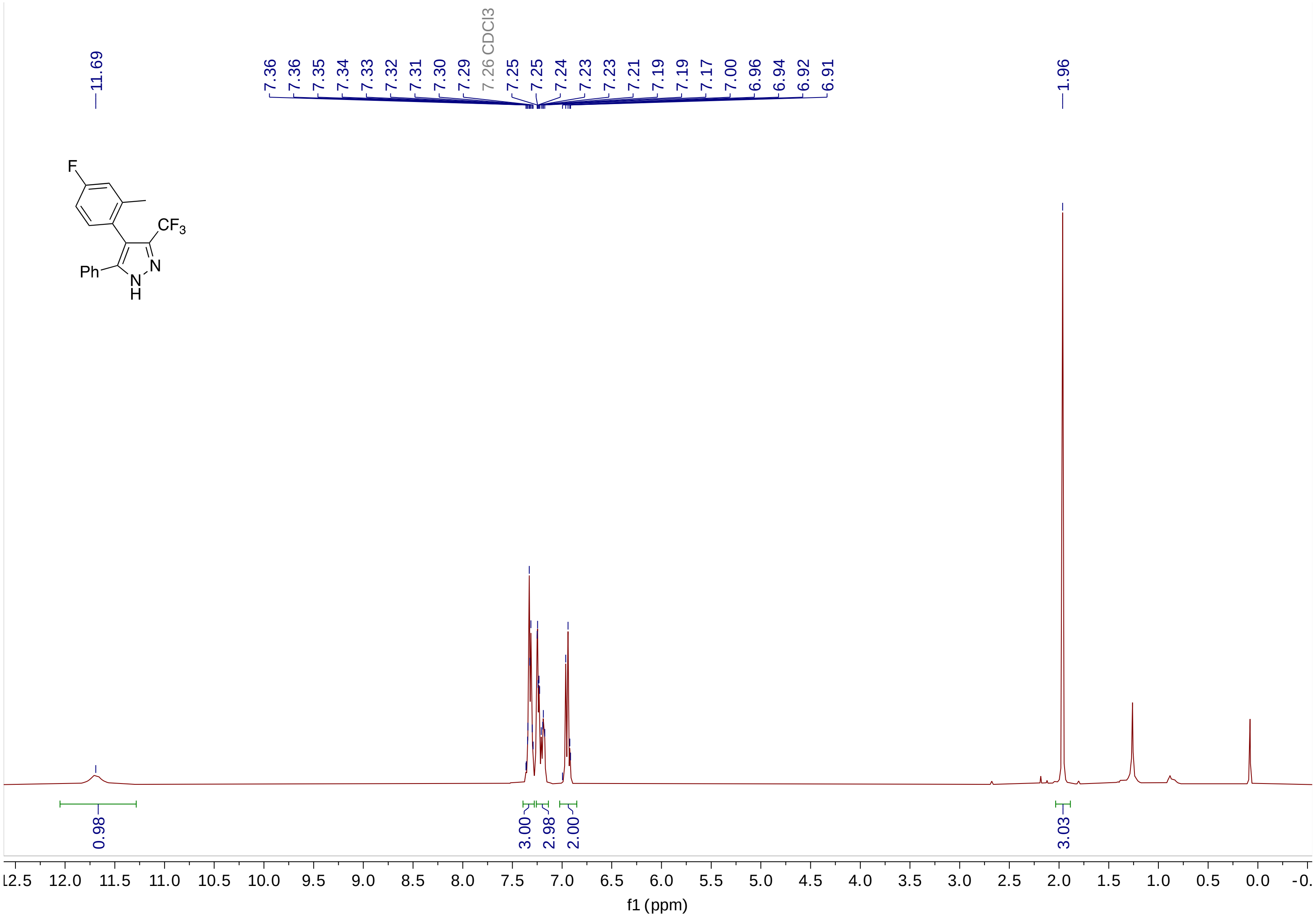

45 - $^{13}C\{^{1}H\}$ NMR (101 MHz, $CDCl_3$):

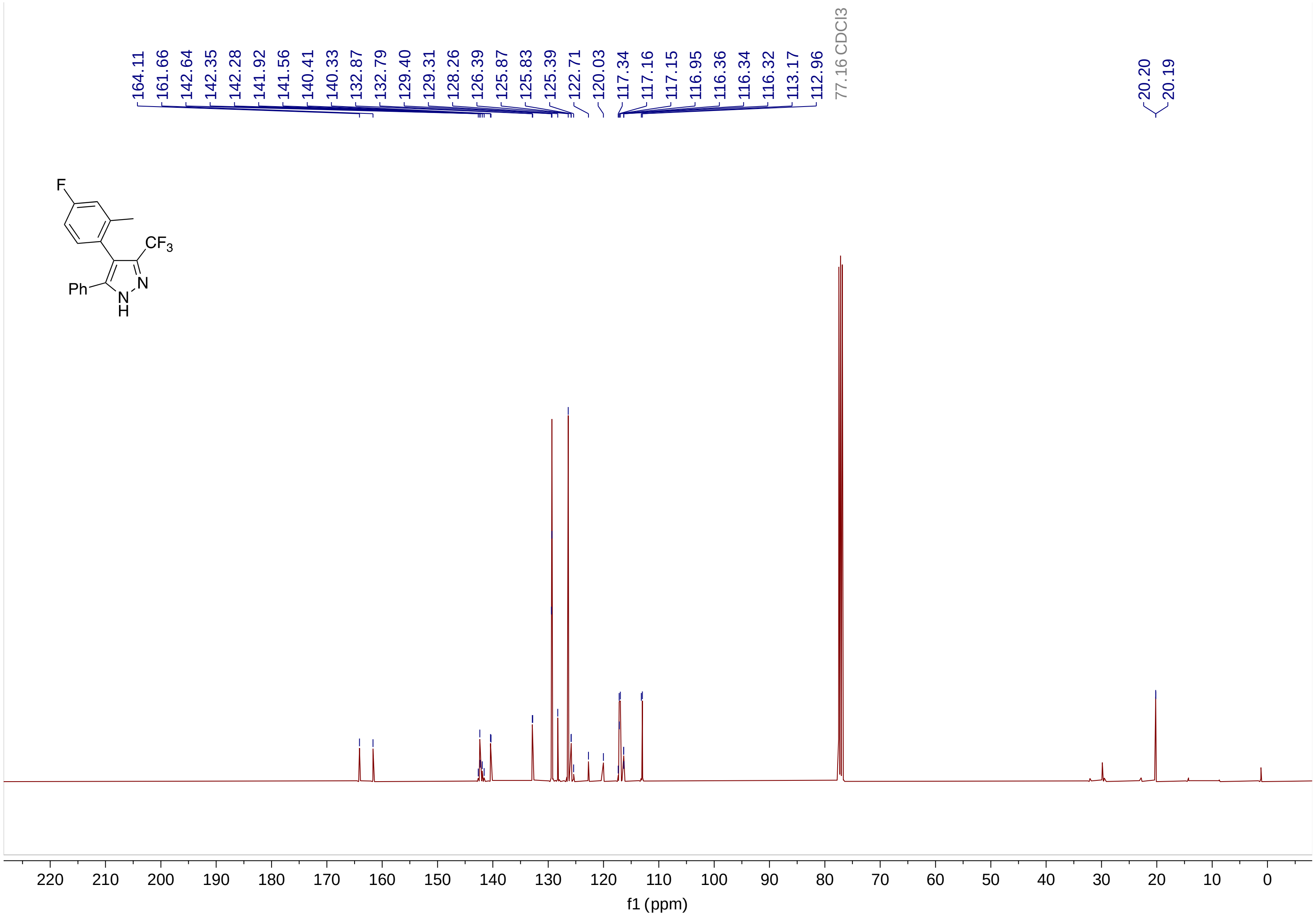

45 - ^{19}F NMR (377 MHz, CDCl$_3$):

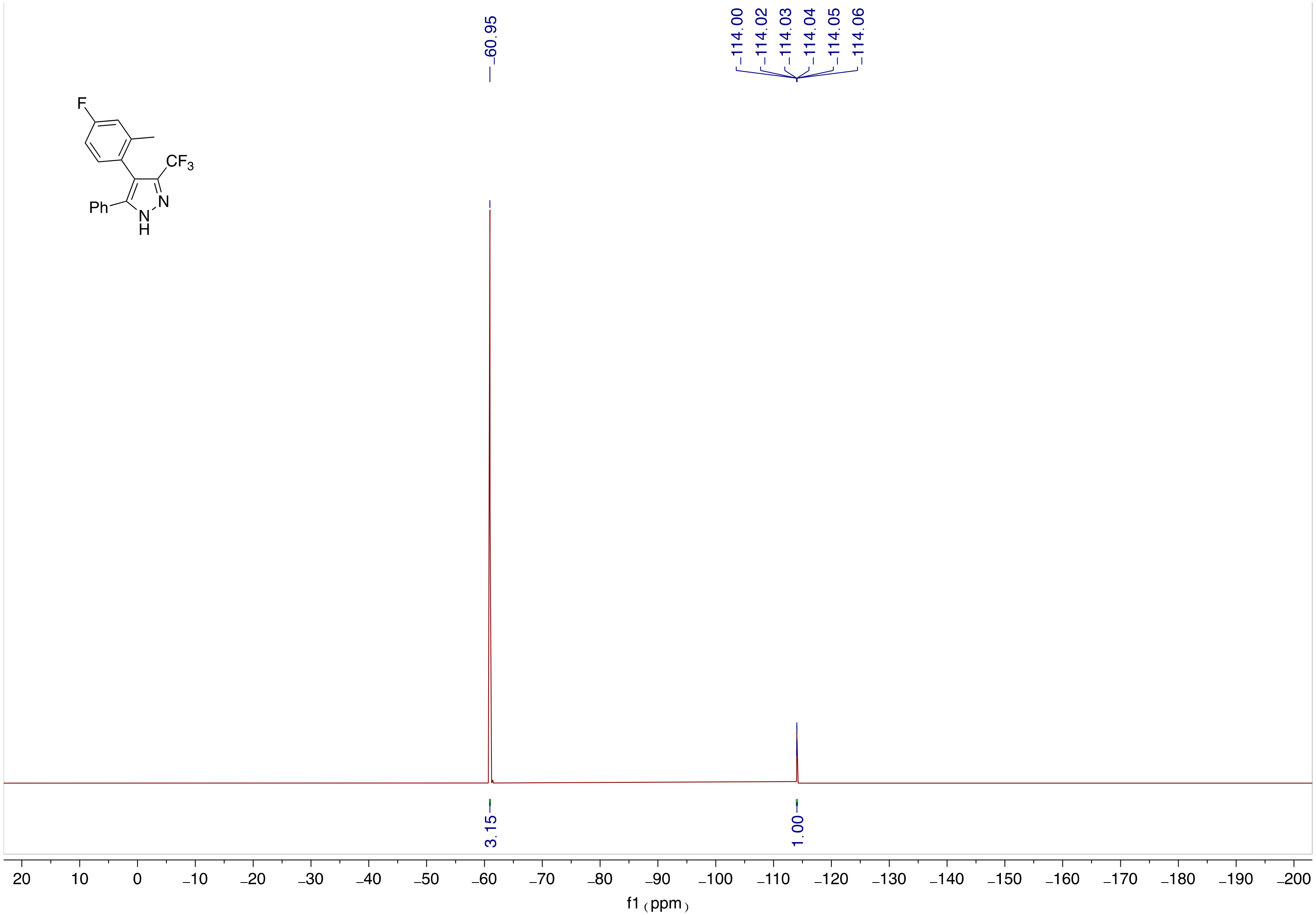

46 - ^{1}H NMR (400 MHz, $CDCl_3$):

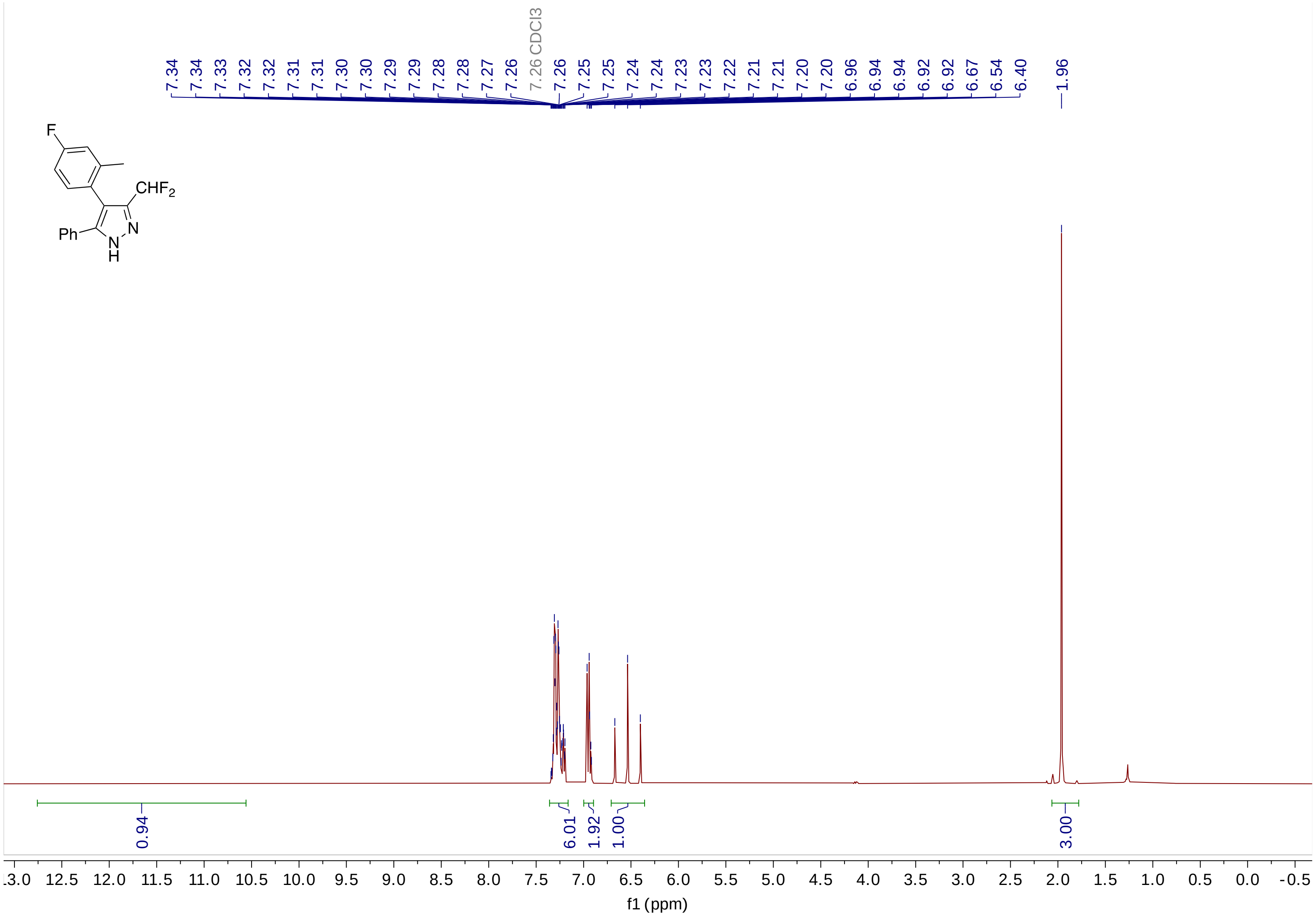

46 - $^{13}C\{^{1}H\}$ NMR (101 MHz, $CDCl_3$):

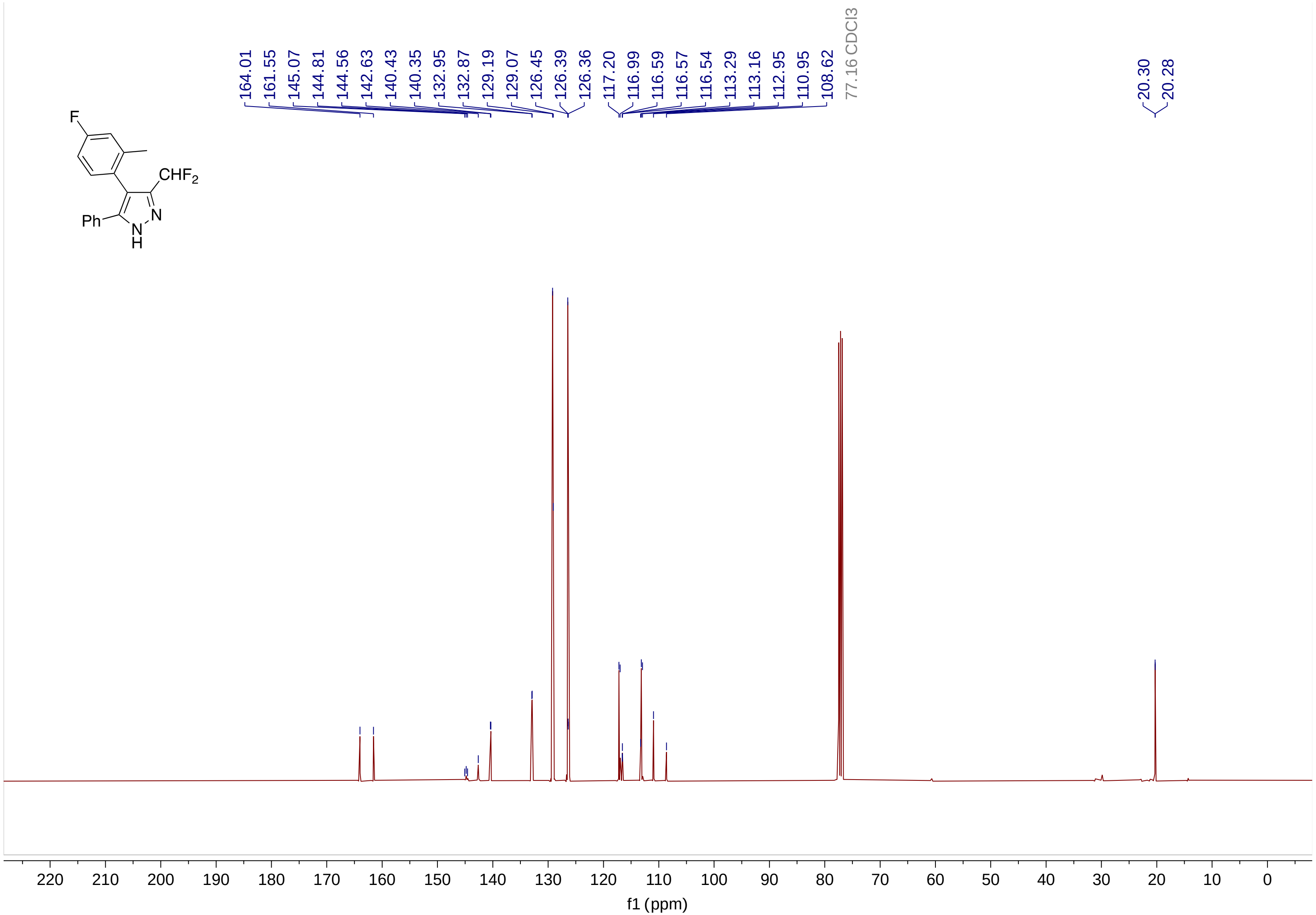

46 - ^{19}F NMR (377 MHz, CDCl$_3$):

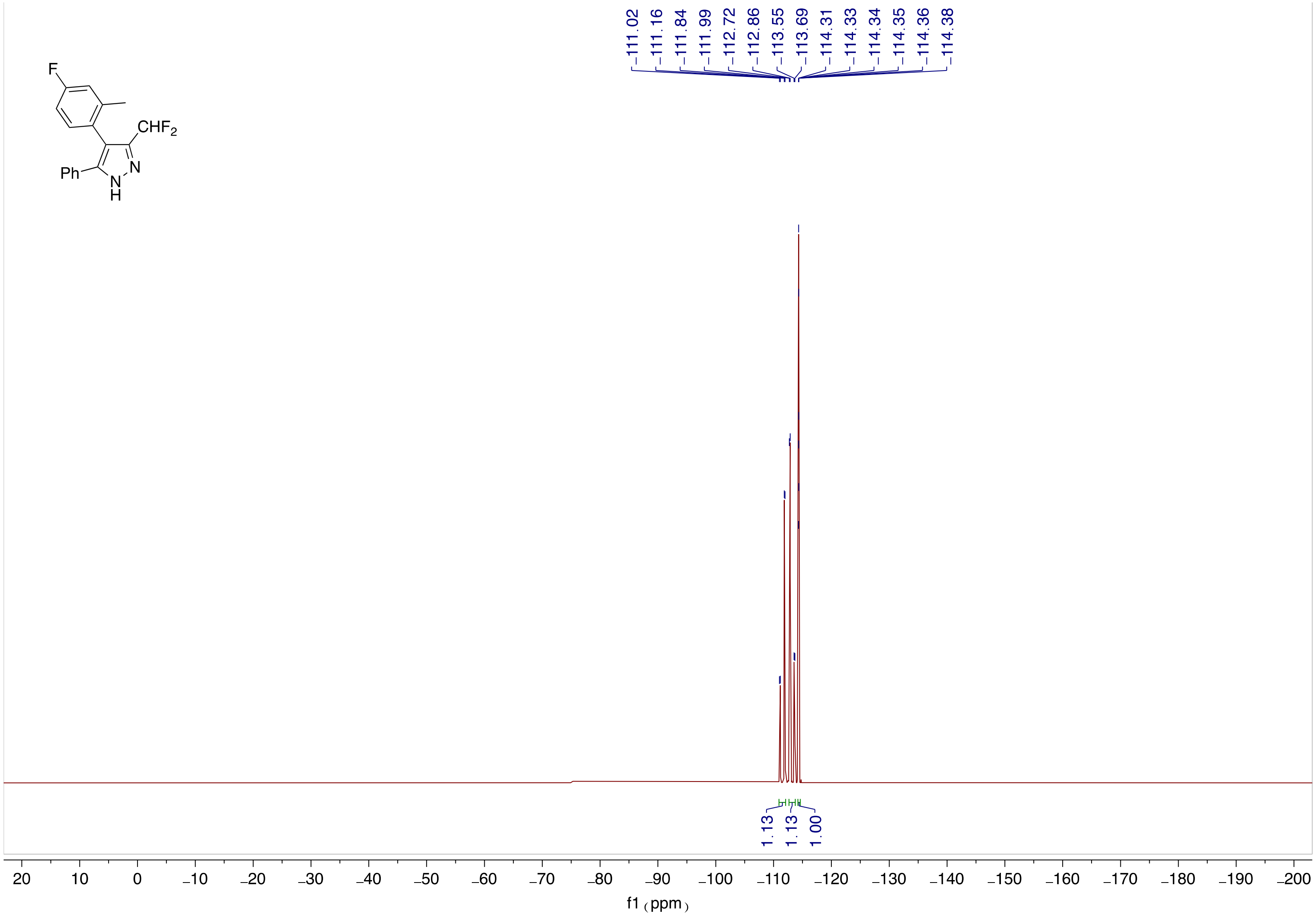

47 - ^{1}H NMR (400 MHz, $CDCl_3$):

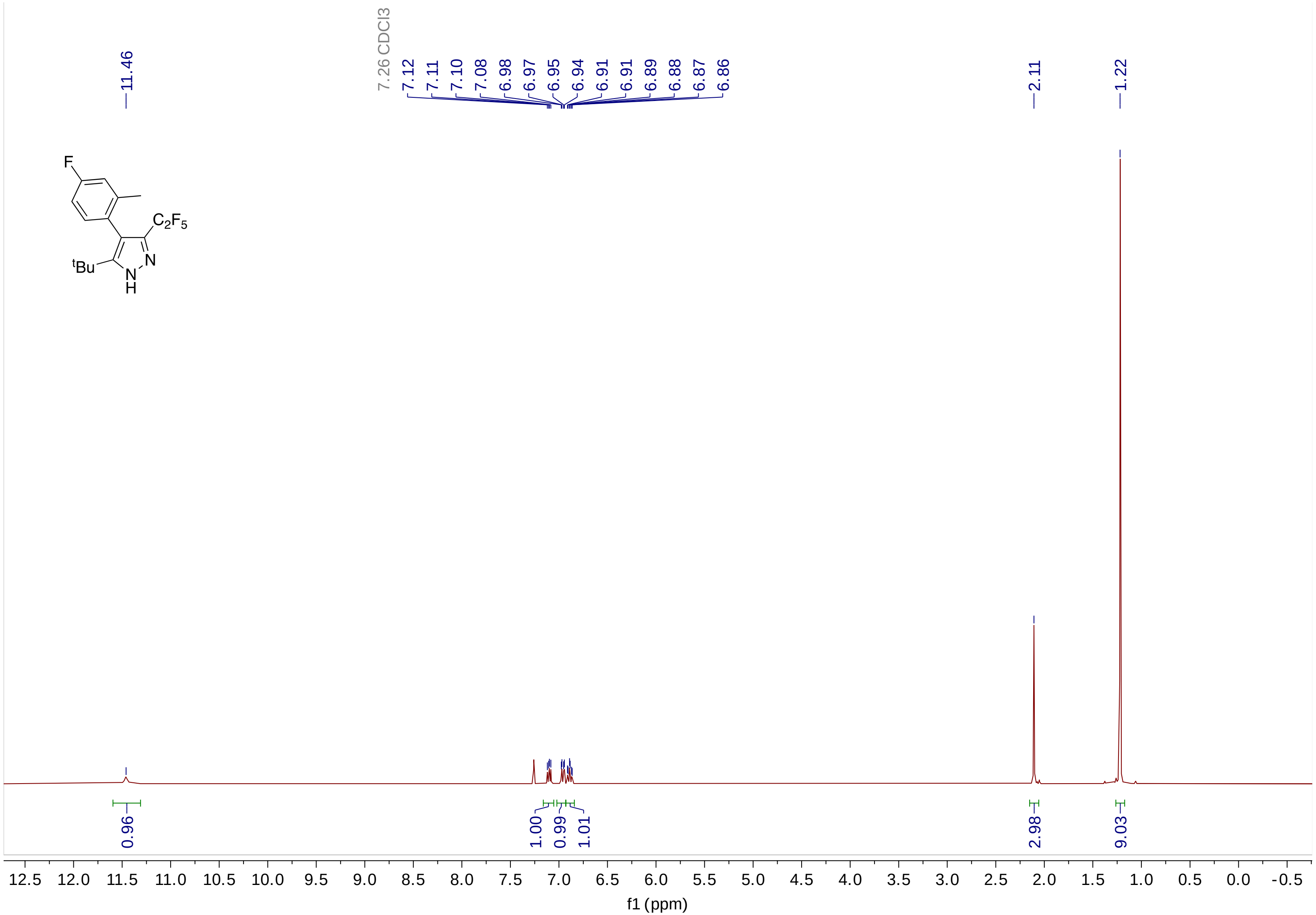

47 - $^{13}C\{^{1}H\}$ NMR (101 MHz, $CDCl_3$):

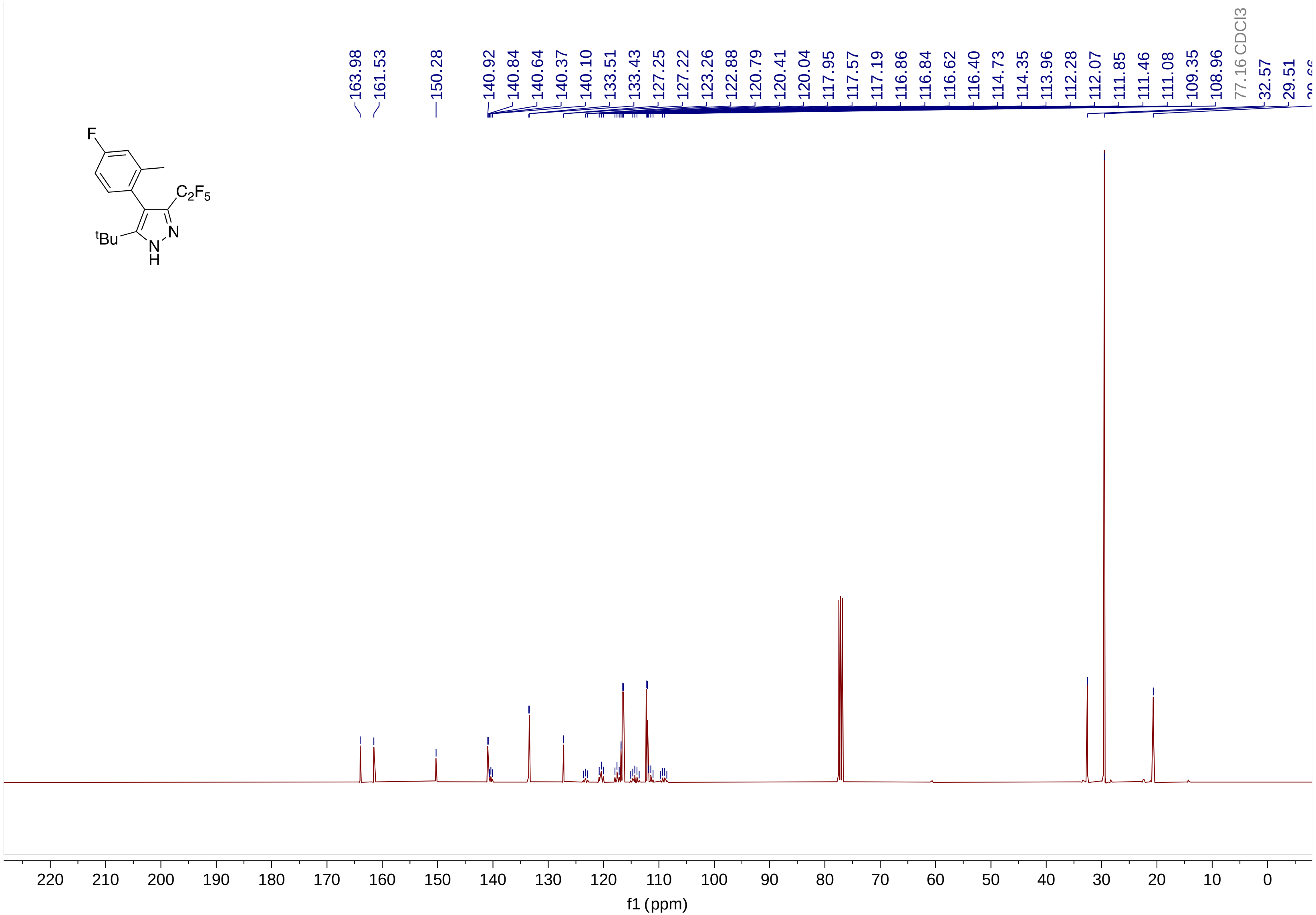

47 - ^{19}F NMR (377 MHz, $CDCl_3$):

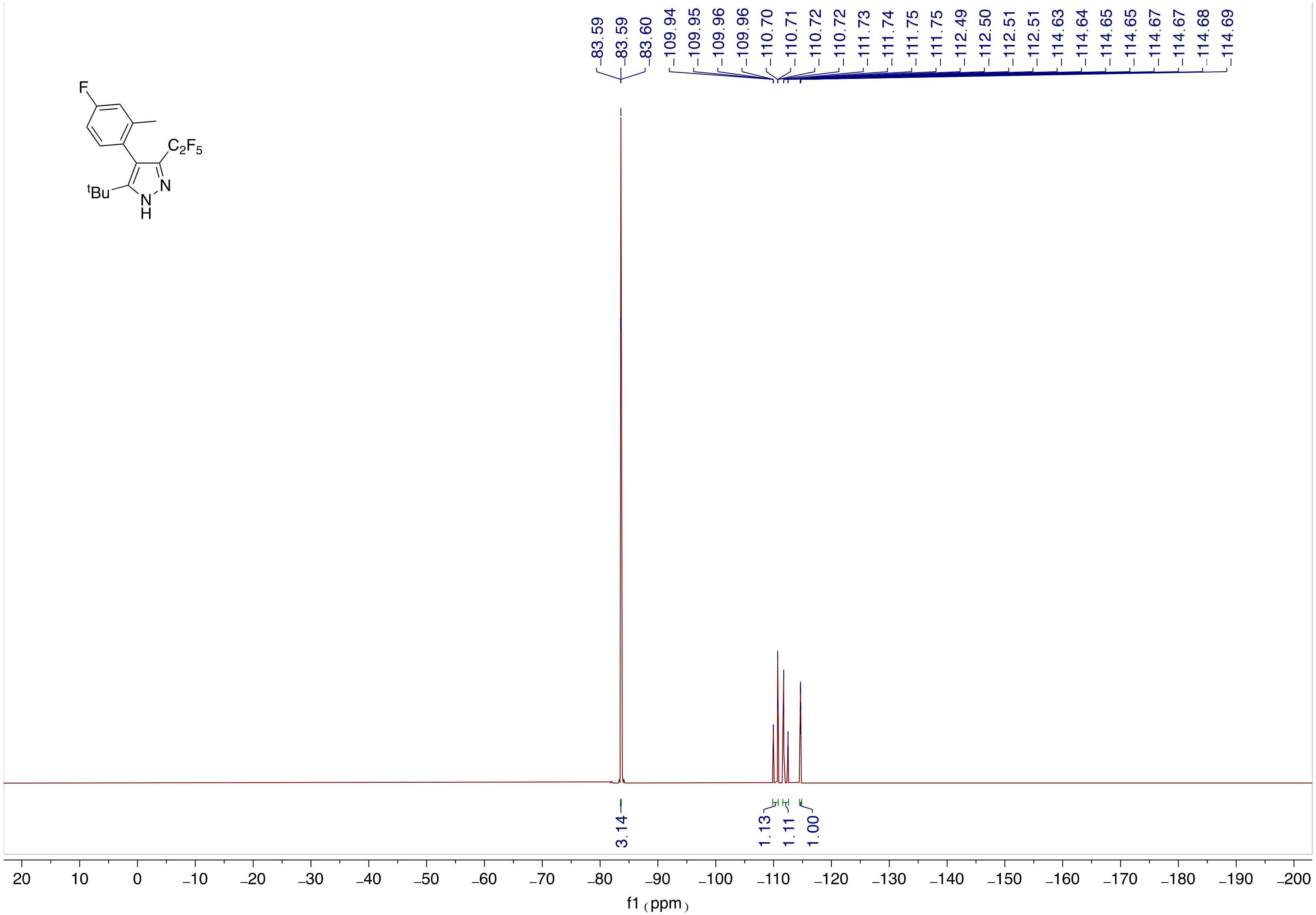

48 - ^{1}H NMR (400 MHz, $CDCl_3$):

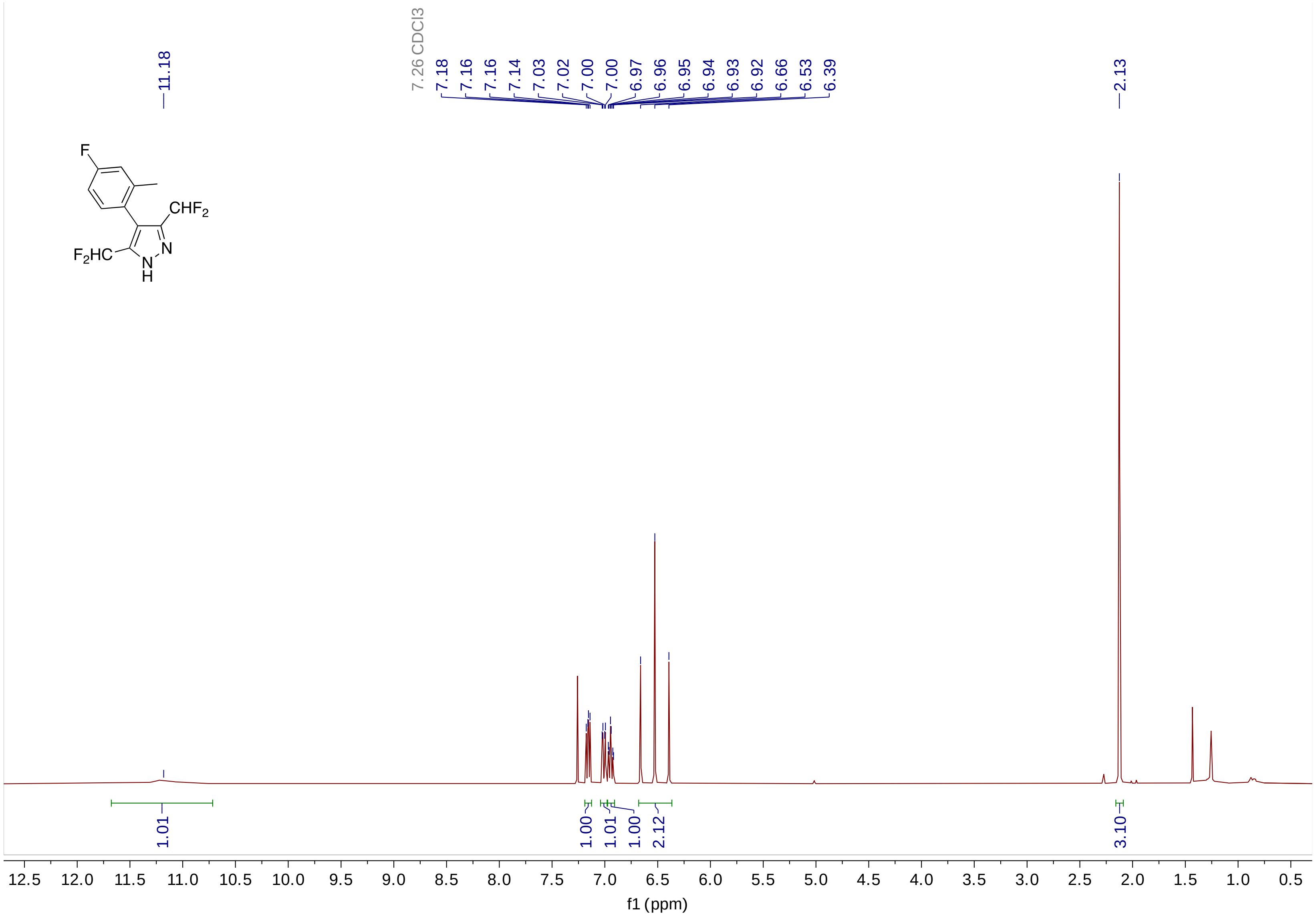

48 - $^{13}C\{^{1}H\}$ NMR (101 MHz, $CDCl_3$):

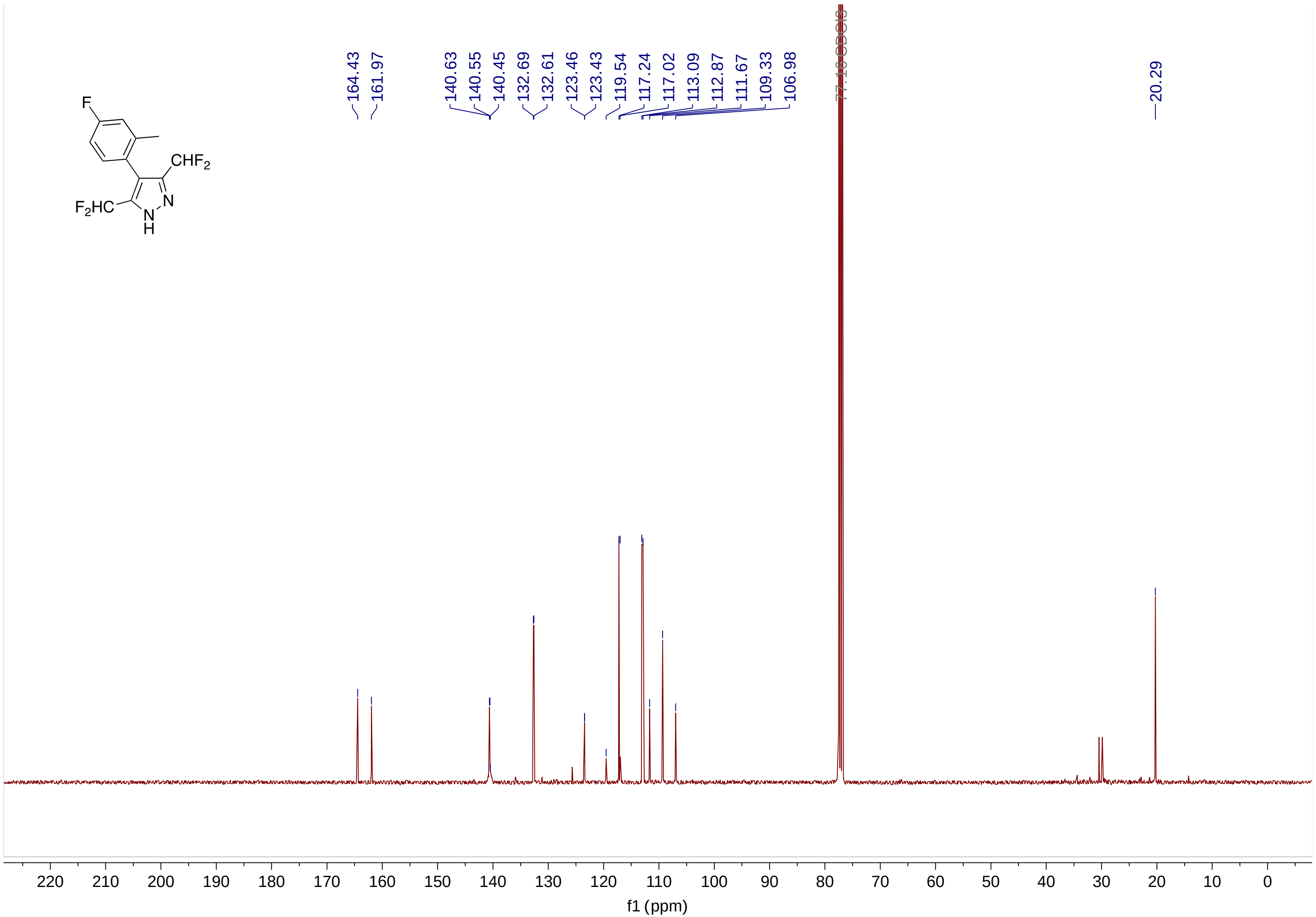

48 - ^{19}F NMR (377 MHz, $CDCl_3$):

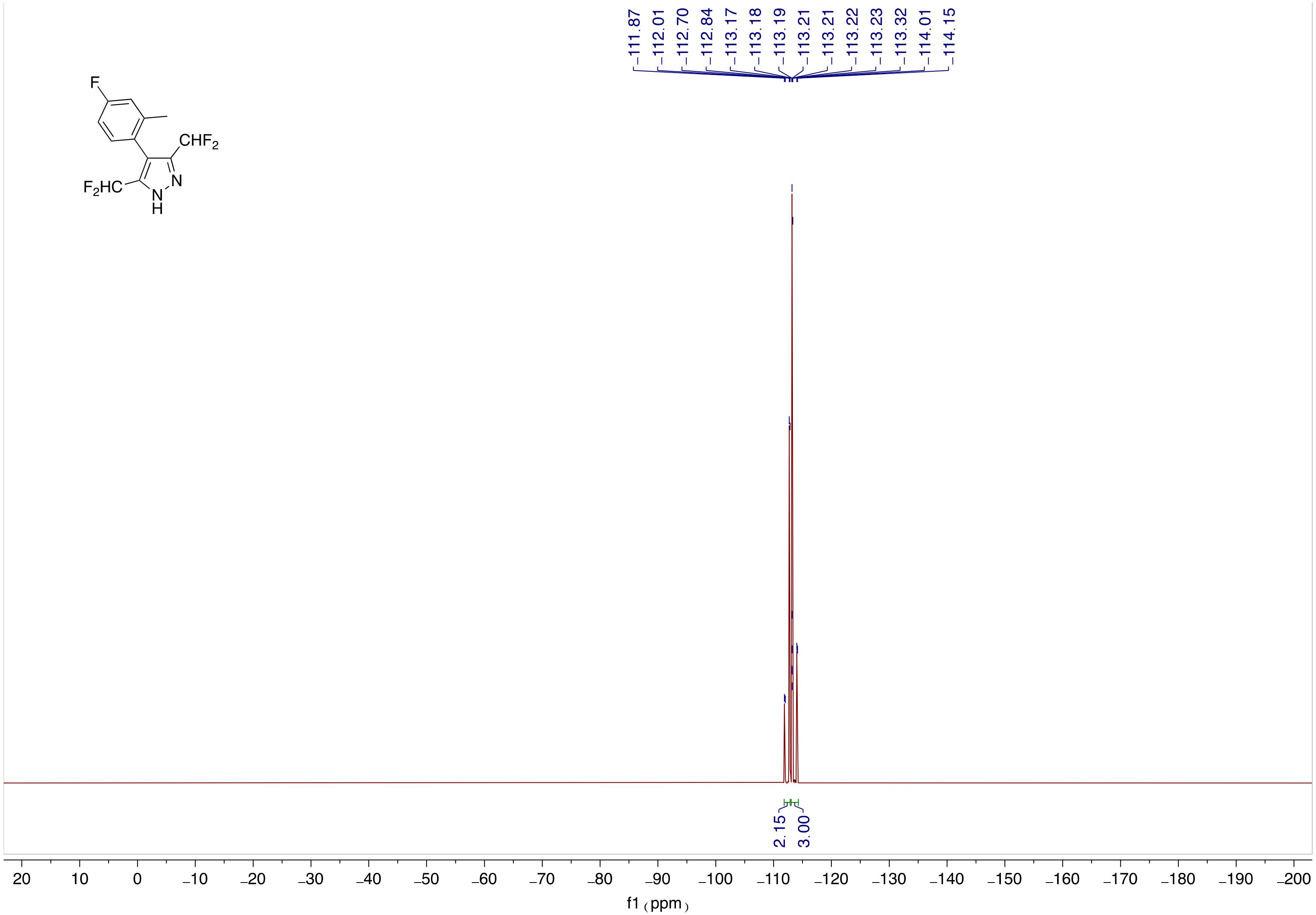

49 - ^{1}H NMR (400 MHz, $CDCl_3$):

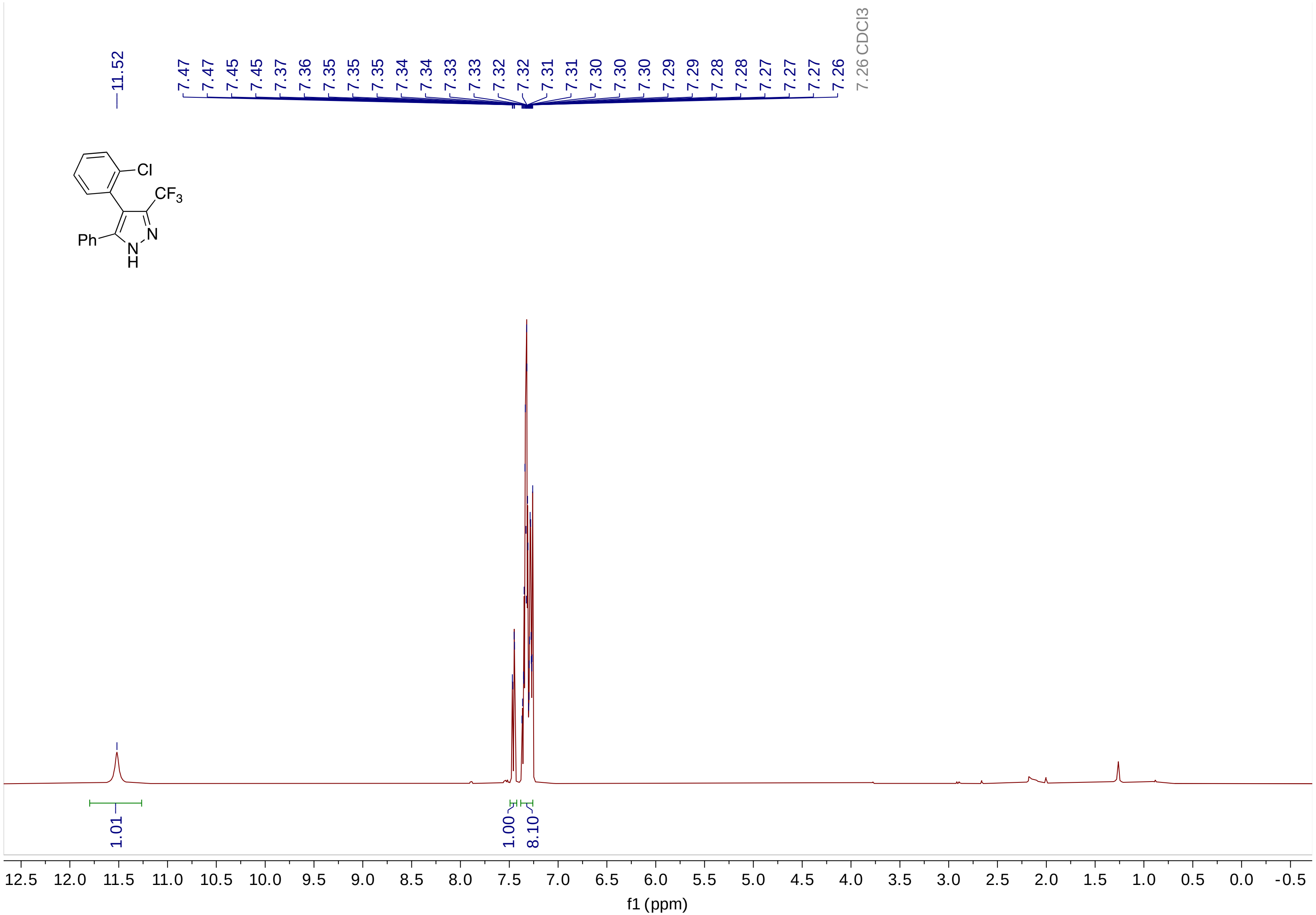

49 - $^{13}C\{^1H\}$ NMR (101 MHz, $CDCl_3$):

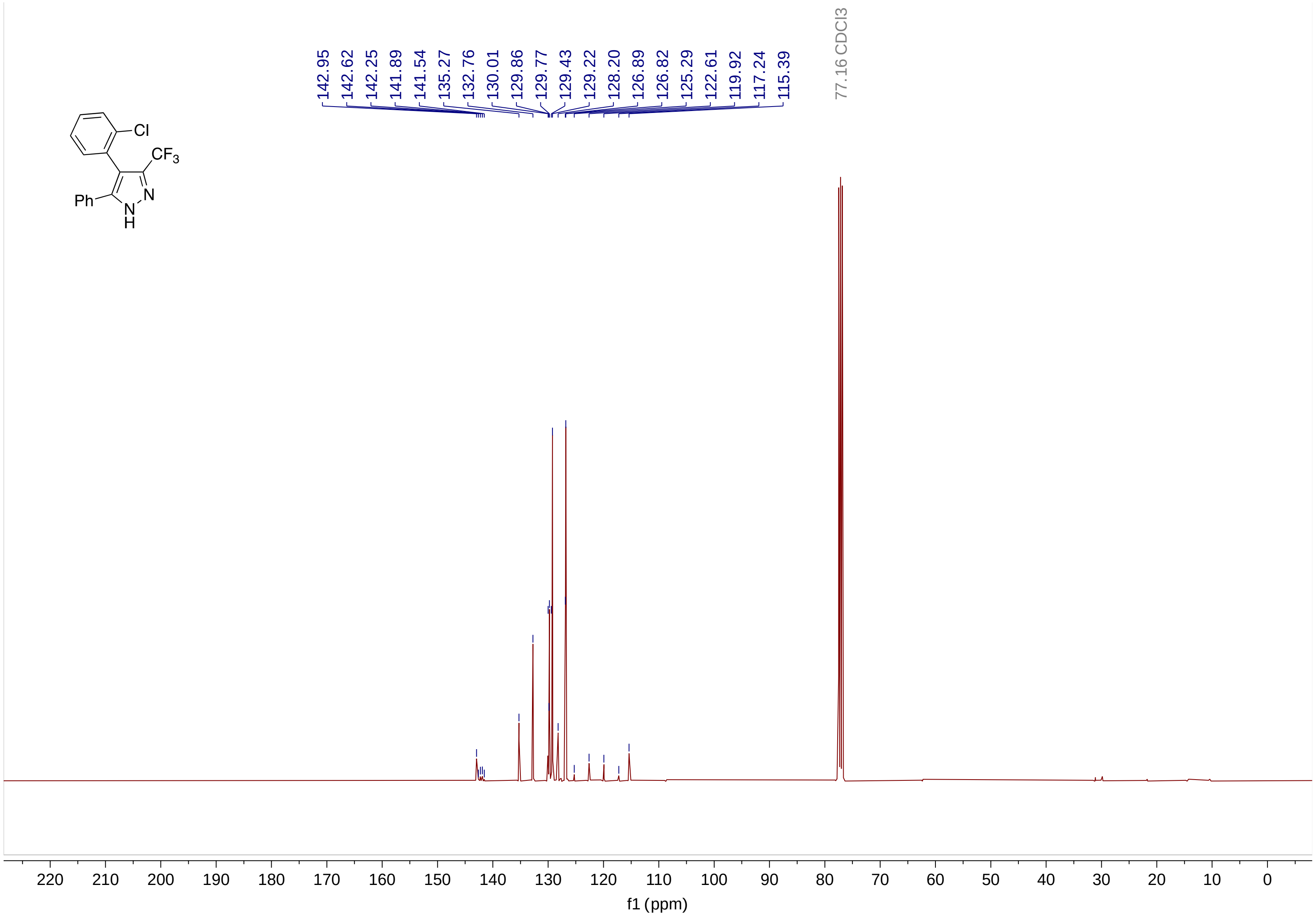

49 - ^{19}F NMR (377 MHz, $CDCl_3$):

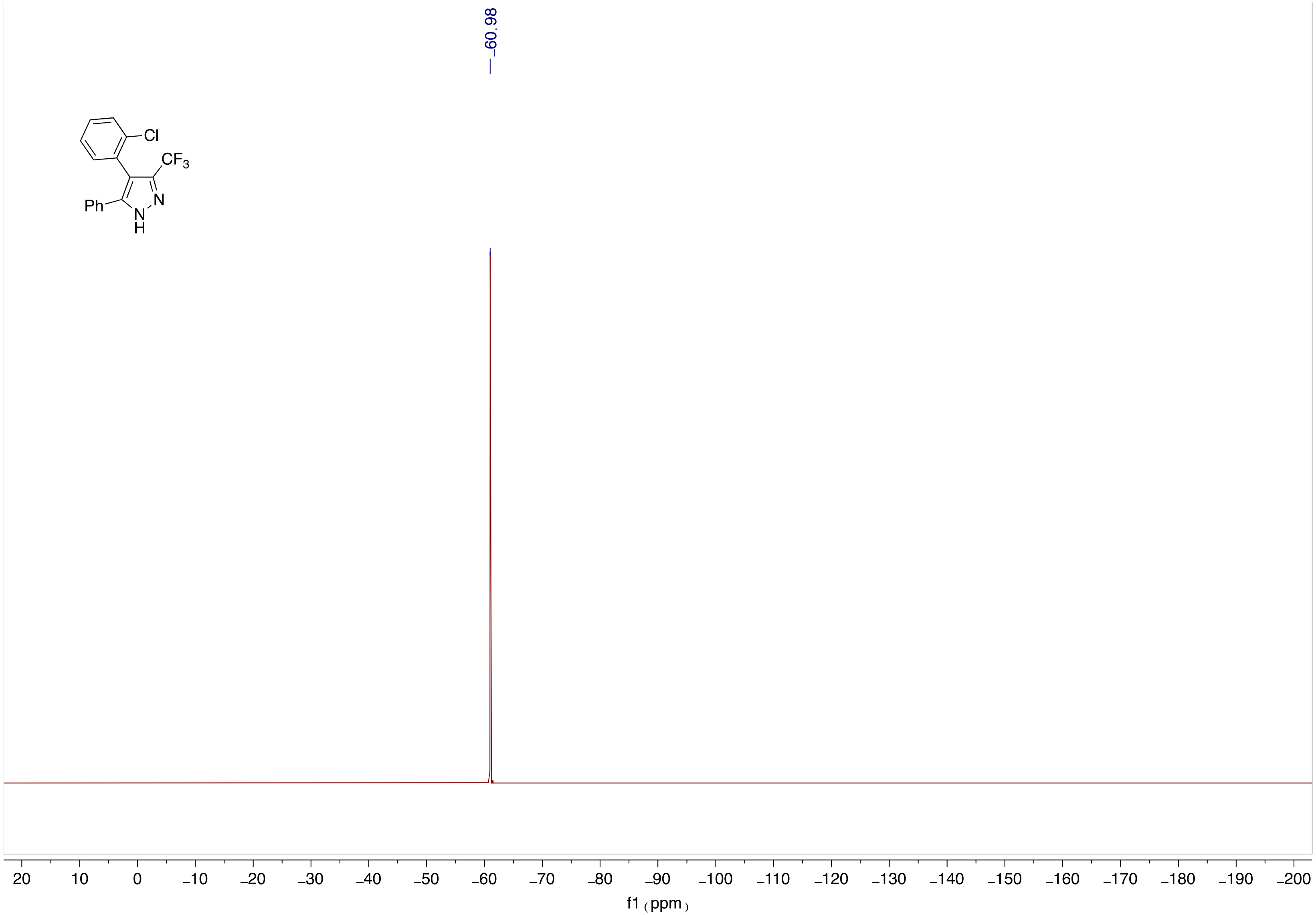

50 - ^{1}H NMR (400 MHz, $CDCl_3$):

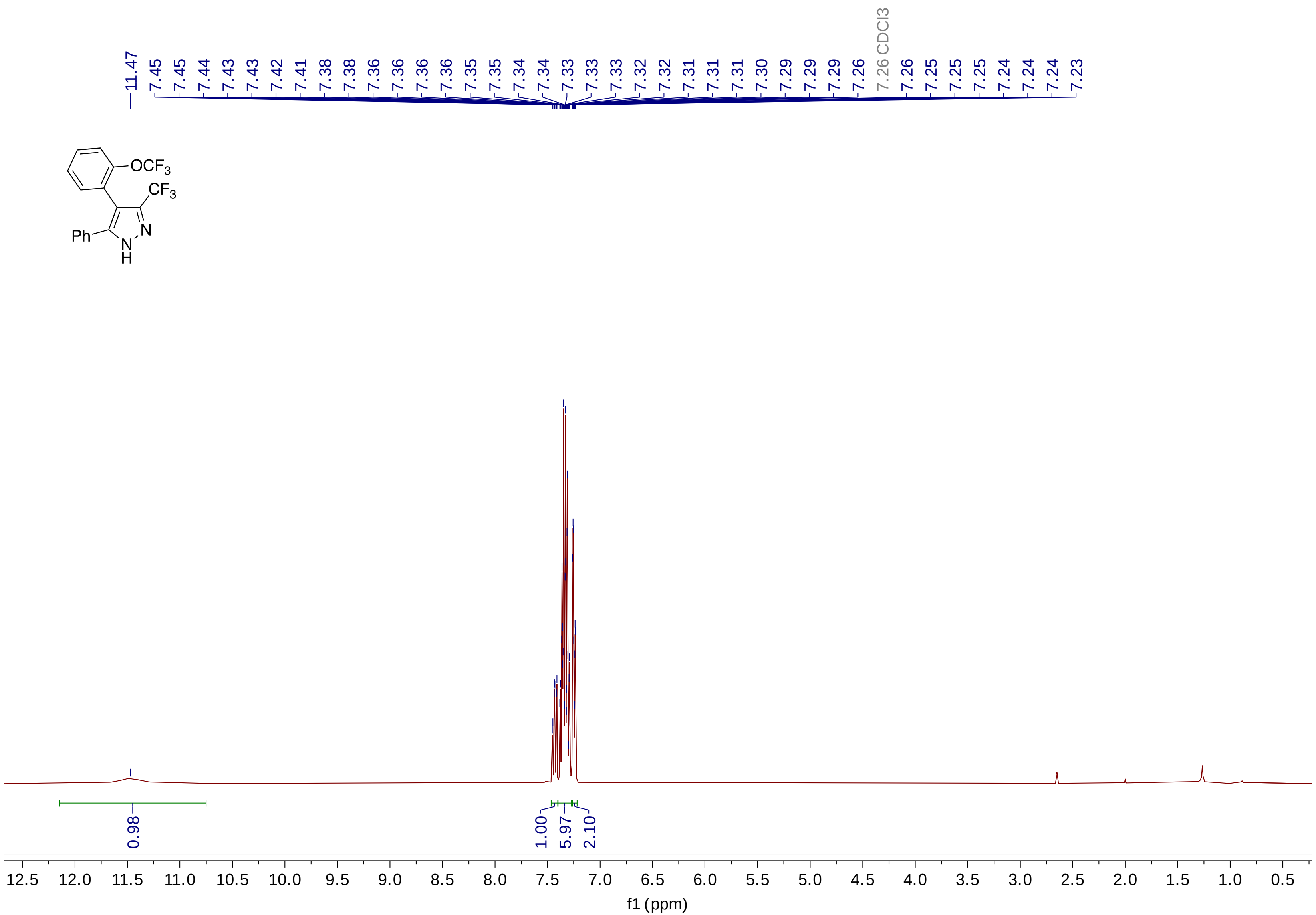

50 - $^{13}C\{^{1}H\}$ NMR (101 MHz, $CDCl_3$):

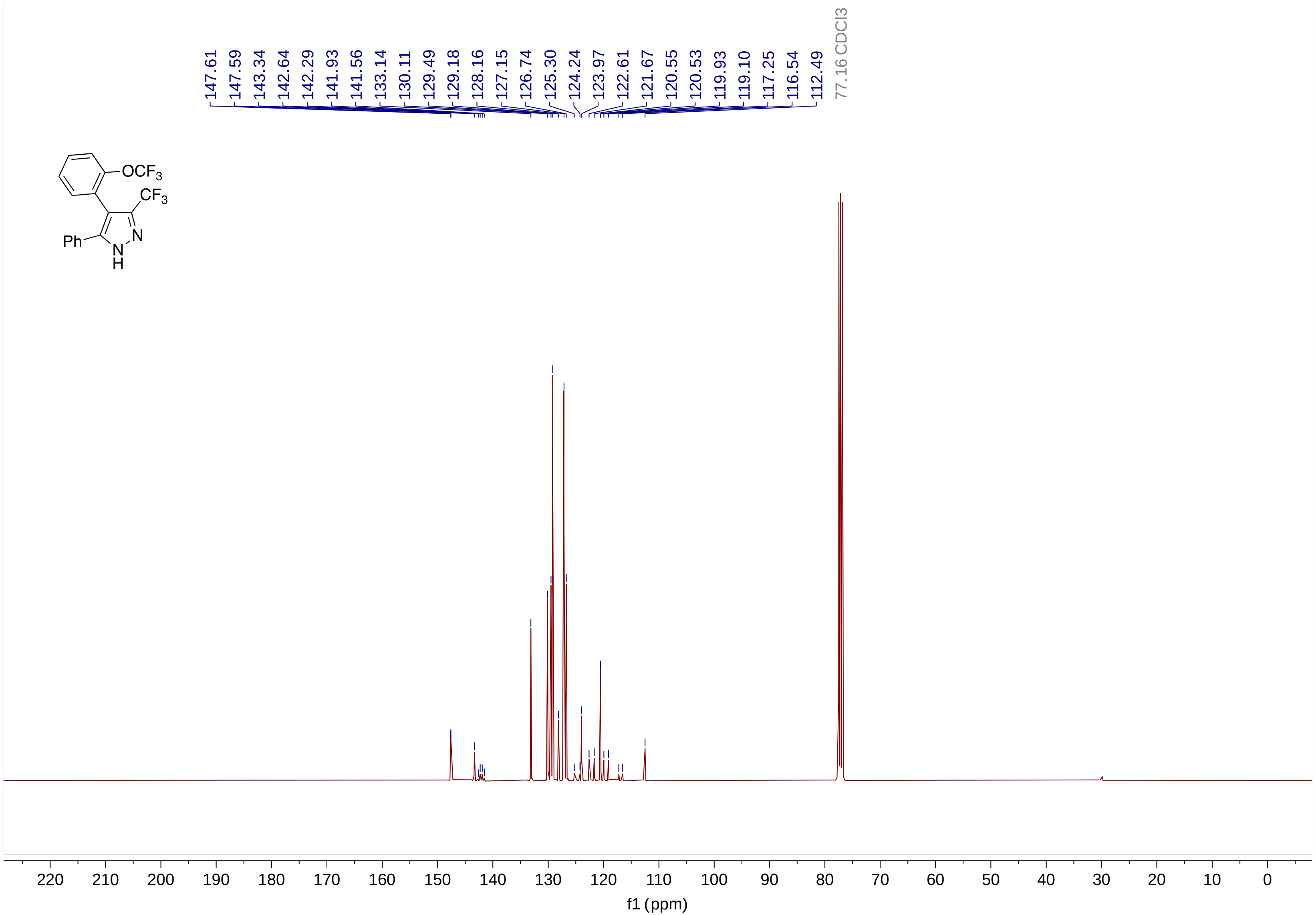

50 - ^{19}F NMR (377 MHz, CDCl$_3$):

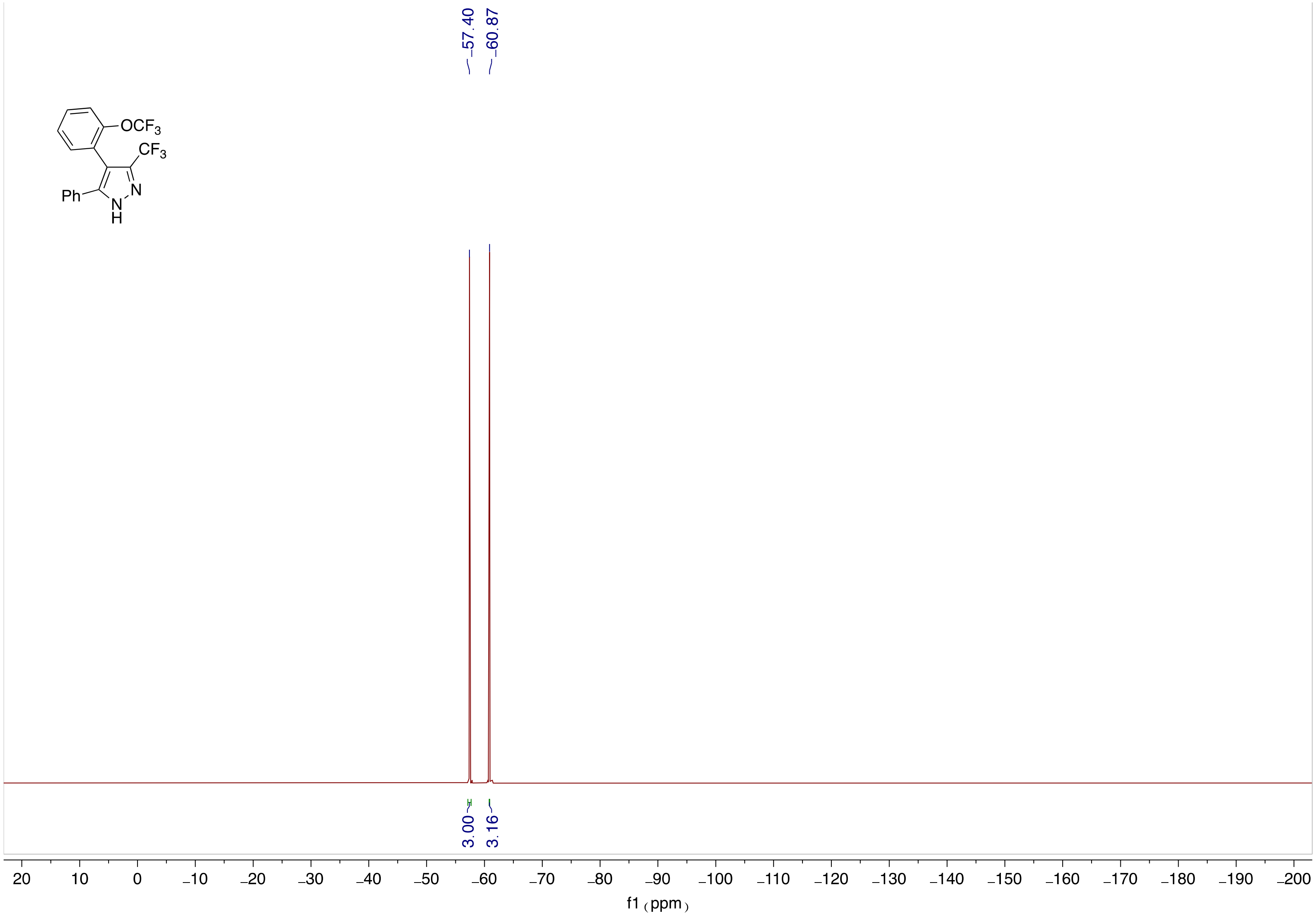

51 - ^{1}H NMR (400 MHz, CDCl₃):

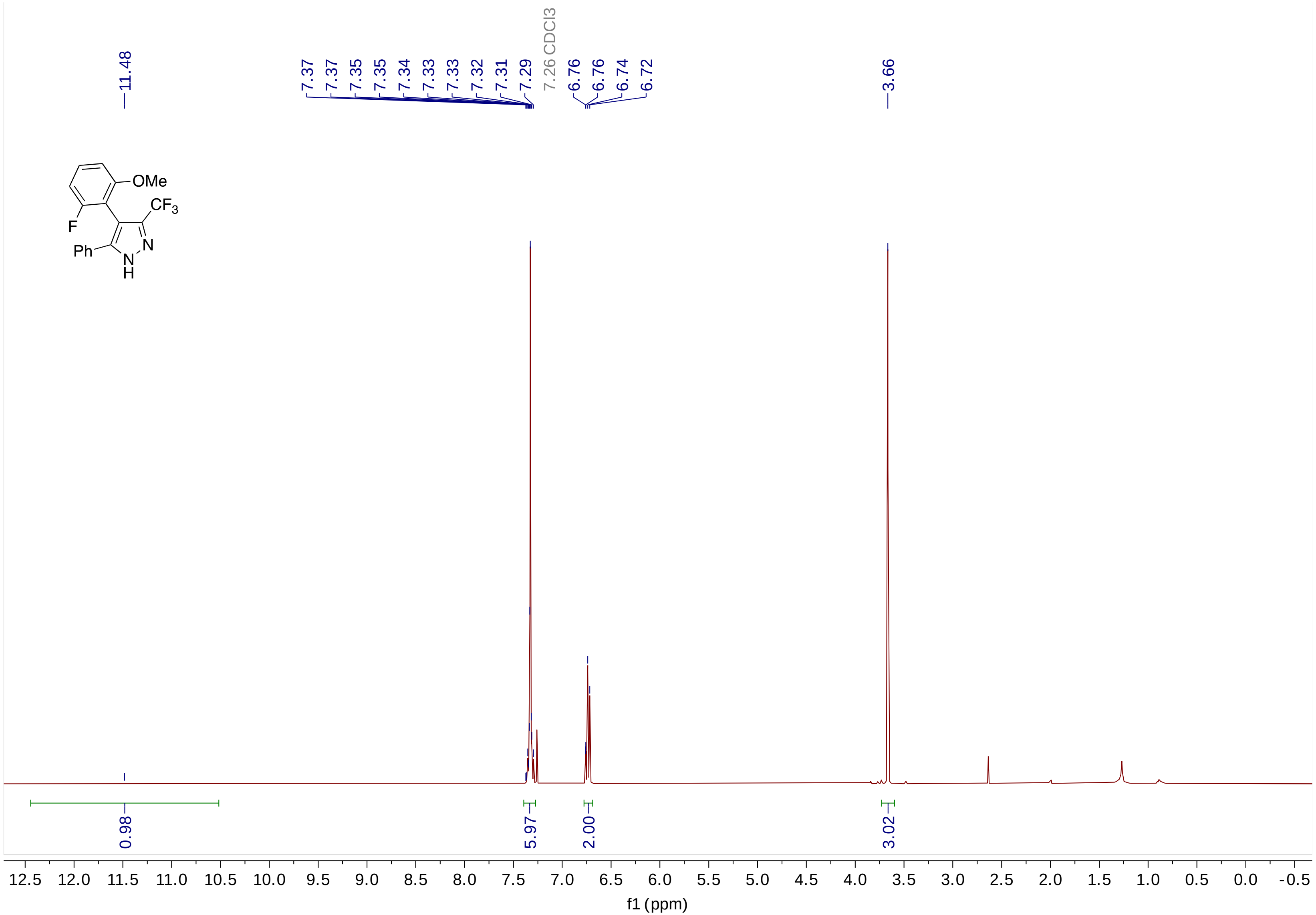

51 - $^{13}C\{^{1}H\}$ NMR (101 MHz, $CDCl_3$):

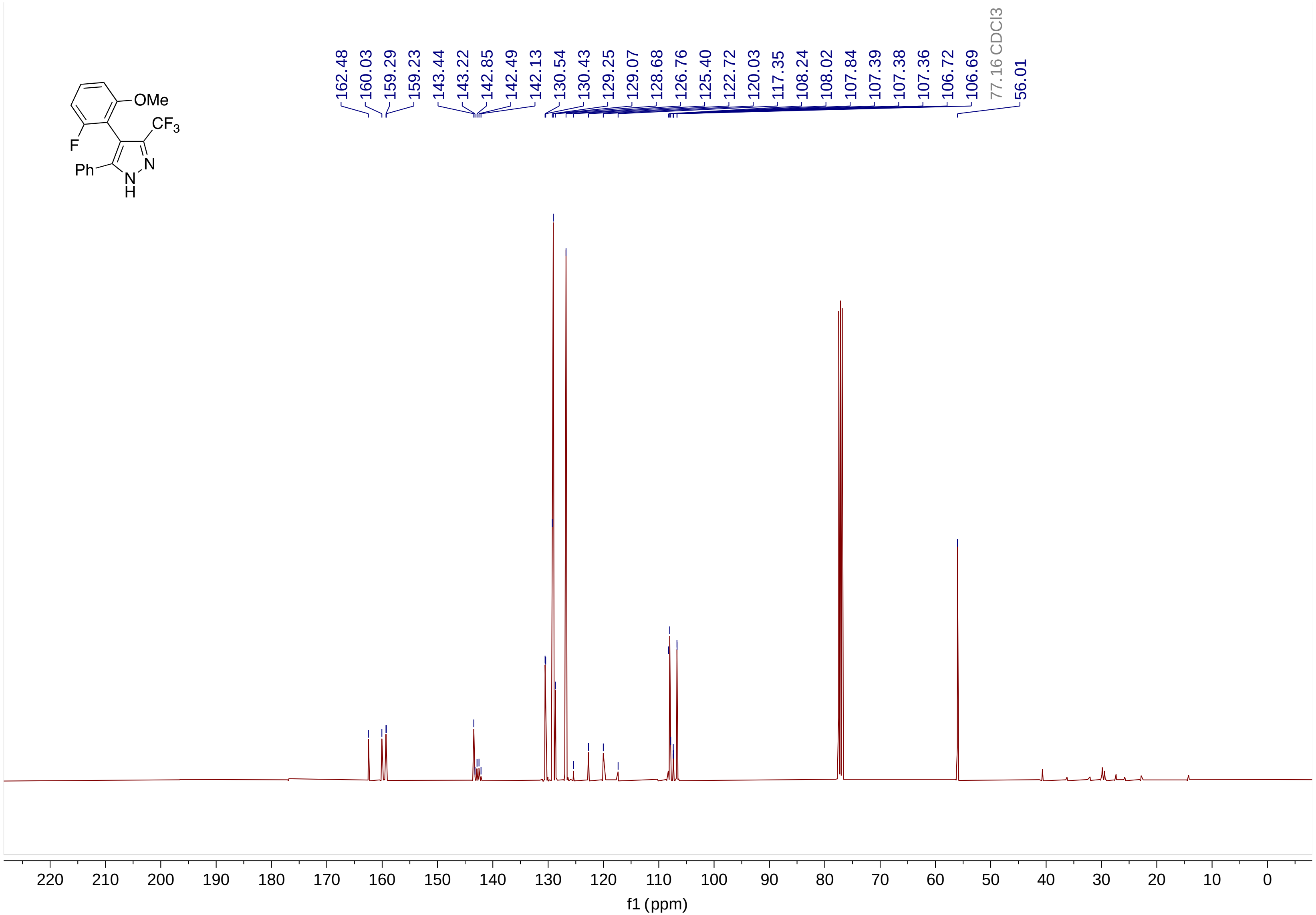

51 - ^{19}F NMR (377 MHz, CDCl$_3$):

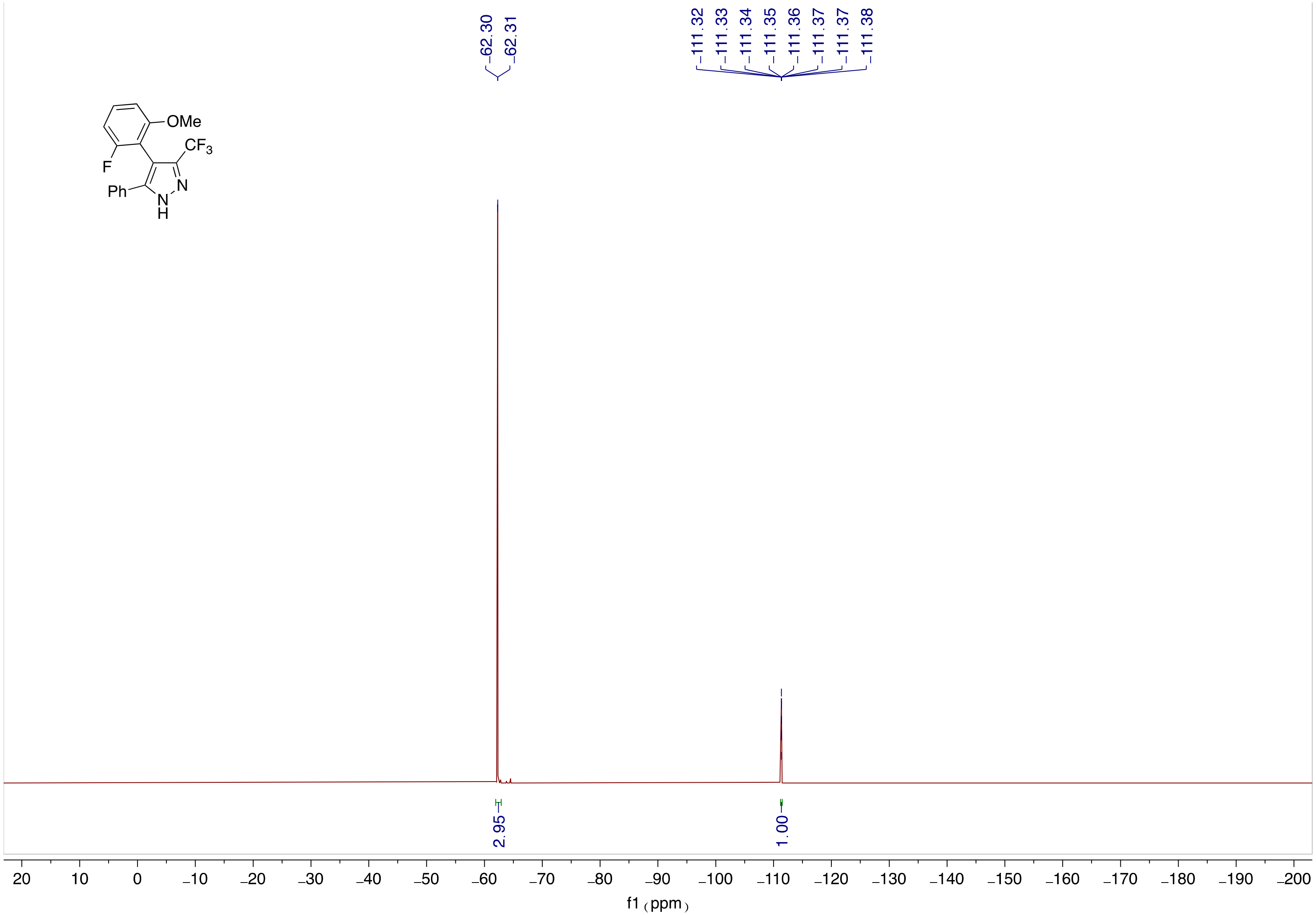

52 - ^{1}H NMR (400 MHz, $CDCl_3$):

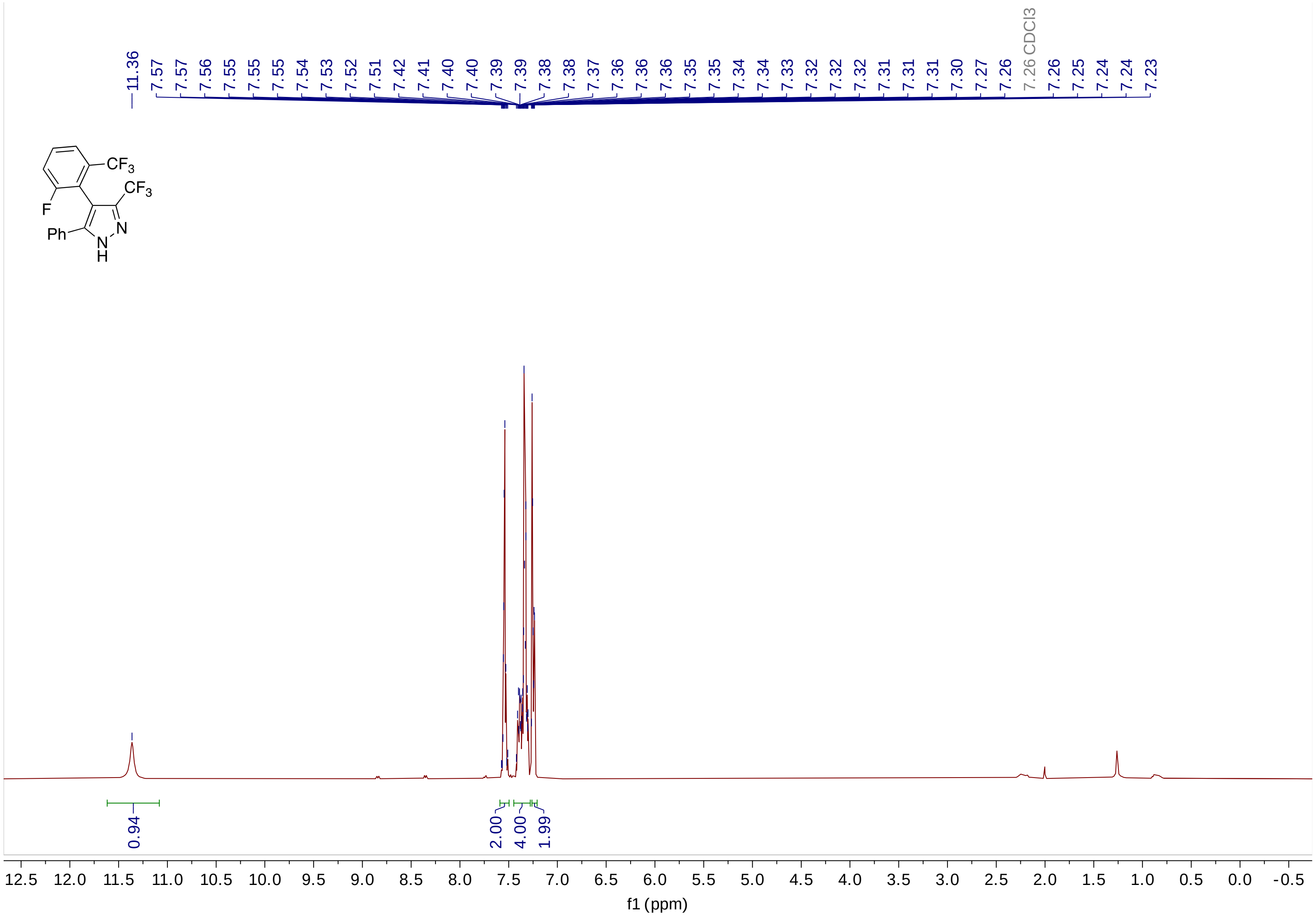

52 - $^{13}C\{^1H\}$ NMR (101 MHz, $CDCl_3$):

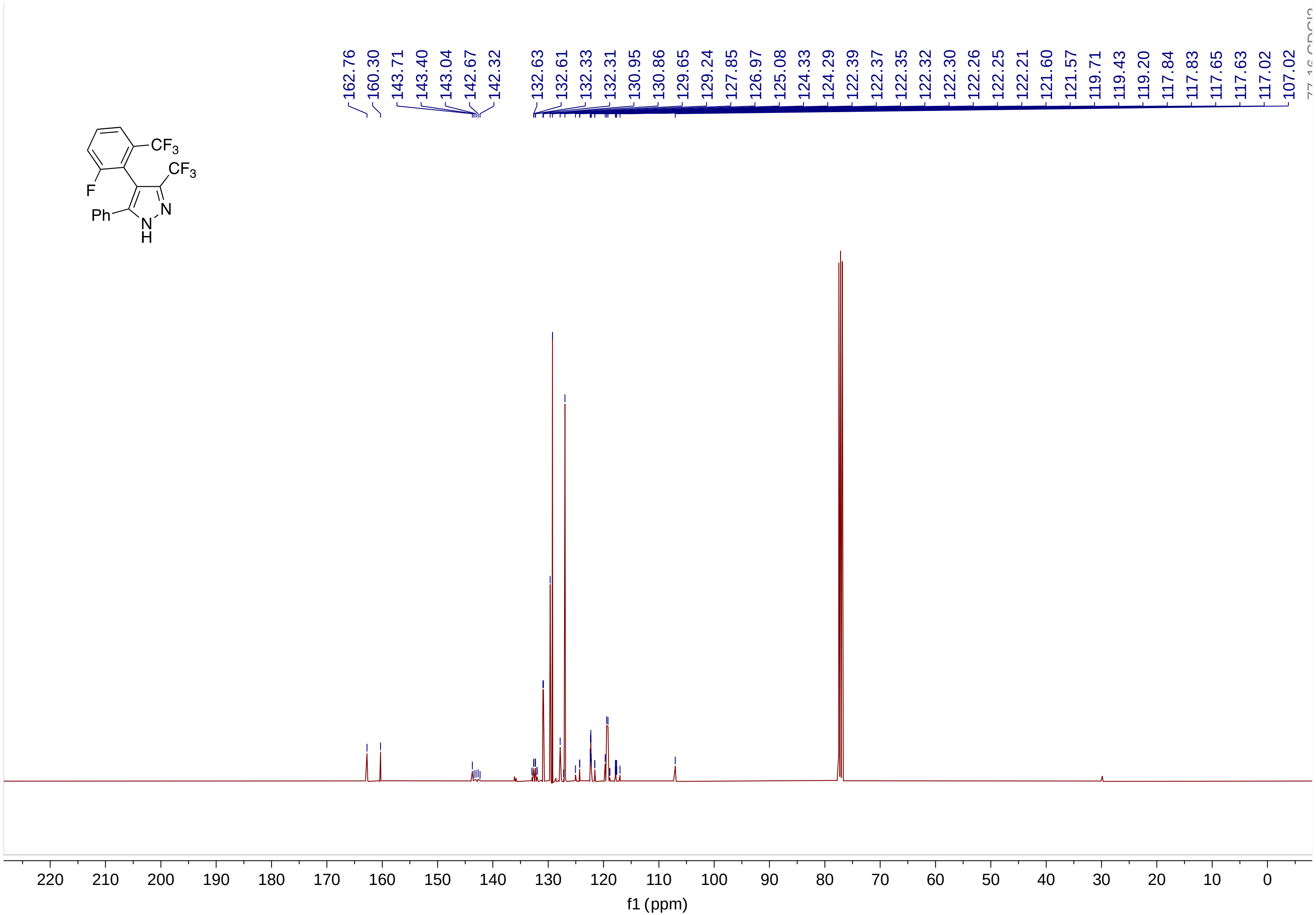

52 - ^{19}F NMR (377 MHz, CDCl$_3$):

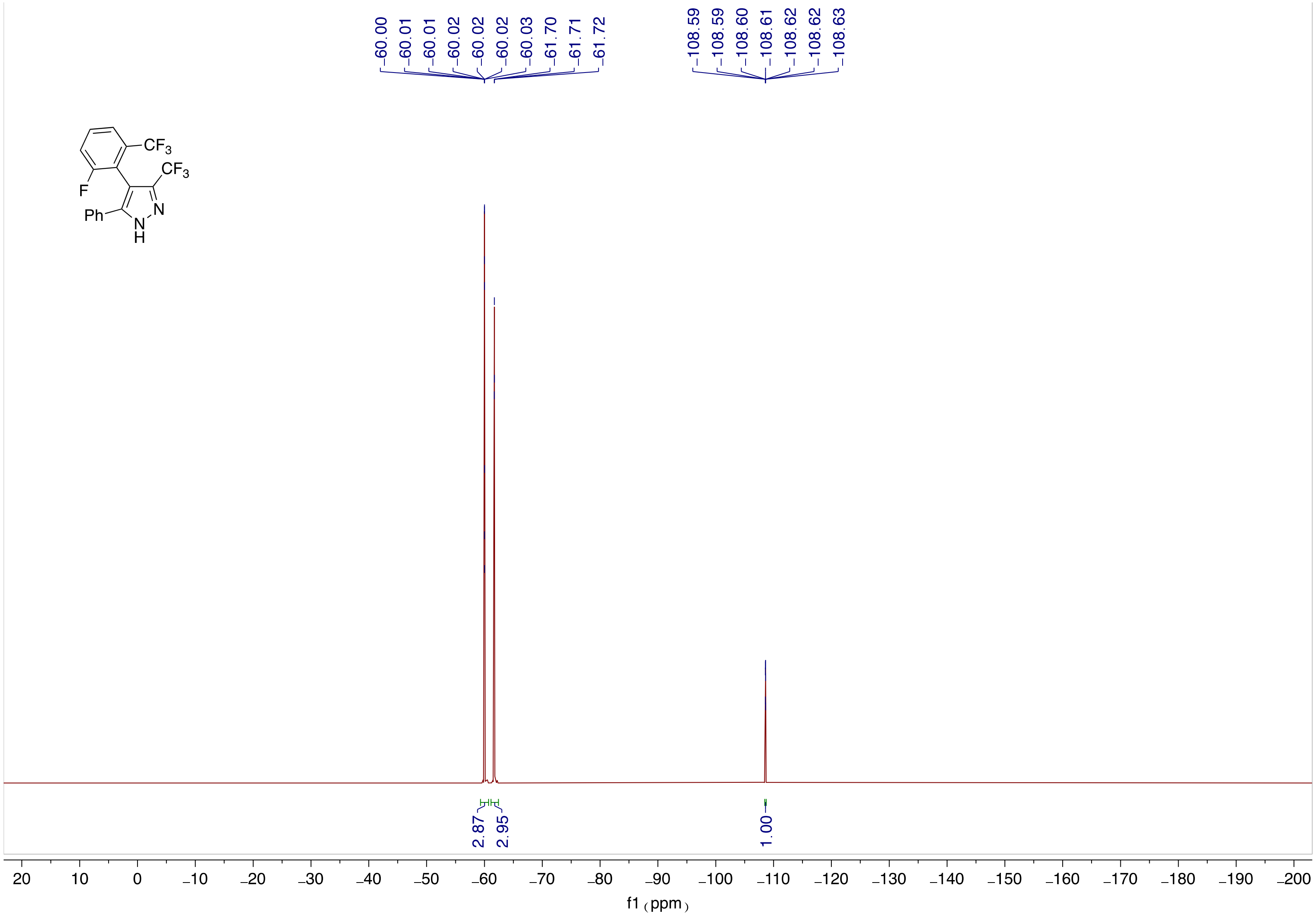

53 - ^{1}H NMR (400 MHz, $CDCl_3$):

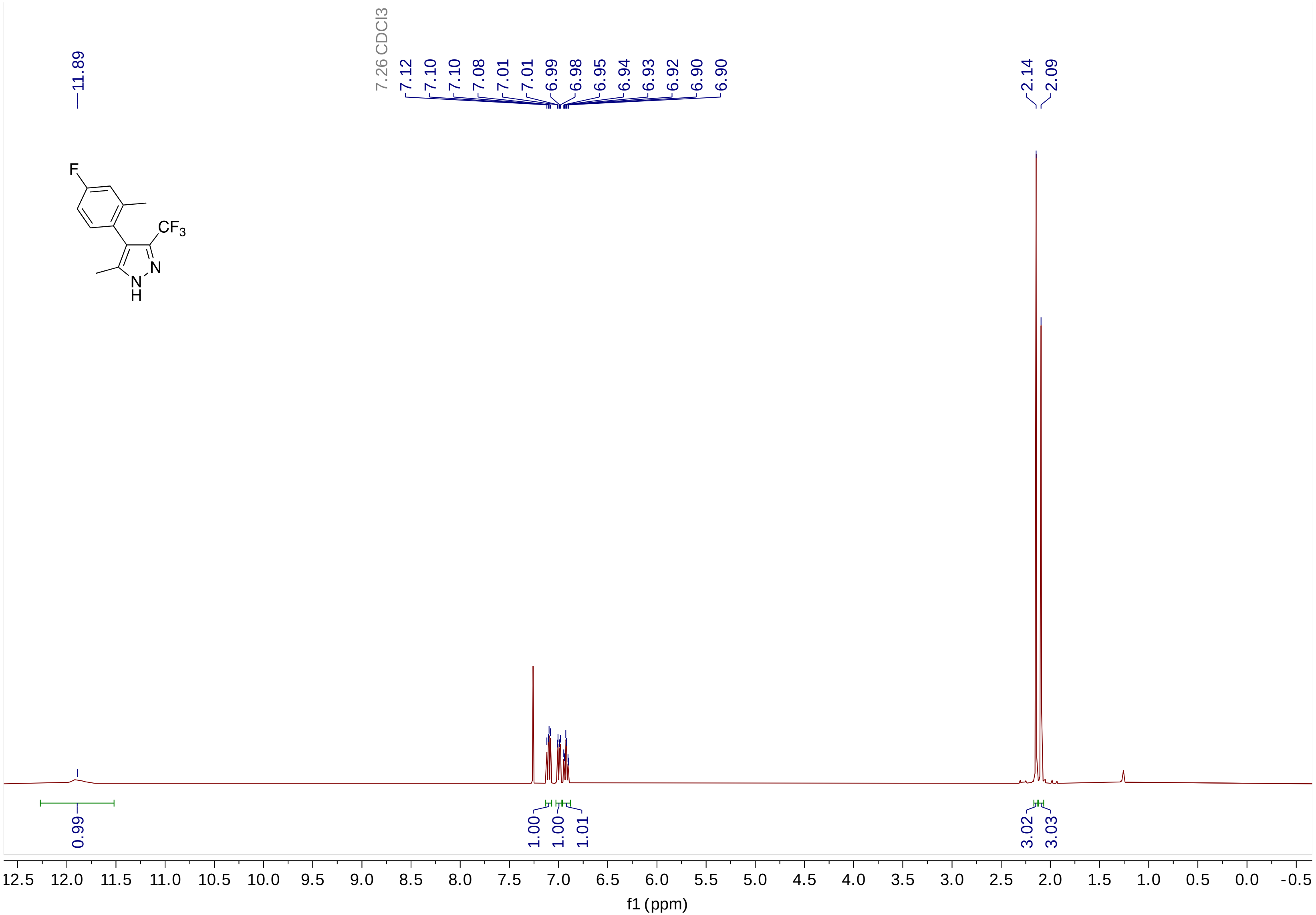

53 - $^{13}C\{^1H\}$ NMR (101 MHz, $CDCl_3$):

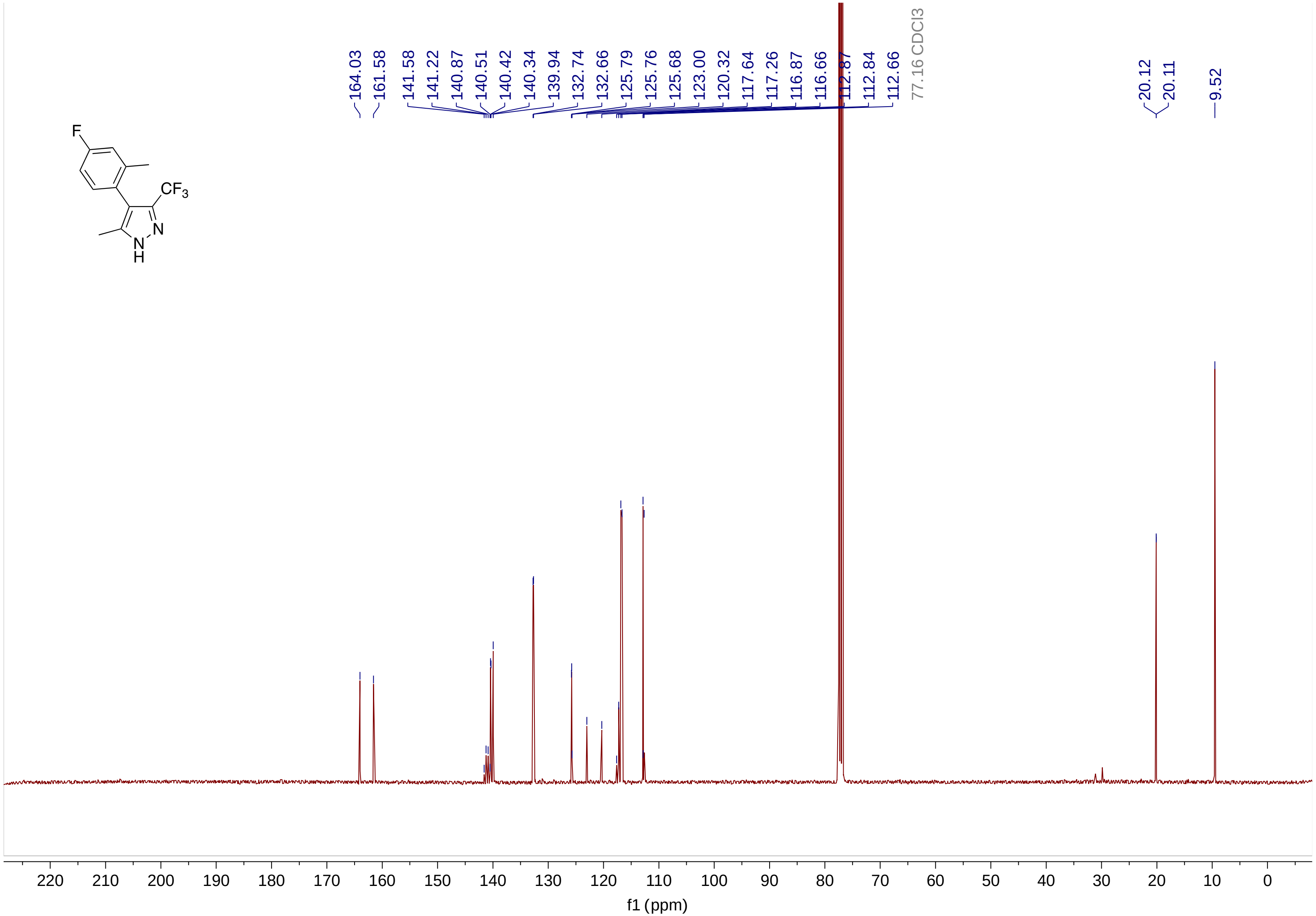

53 - ^{19}F NMR (377 MHz, CDCl$_3$):

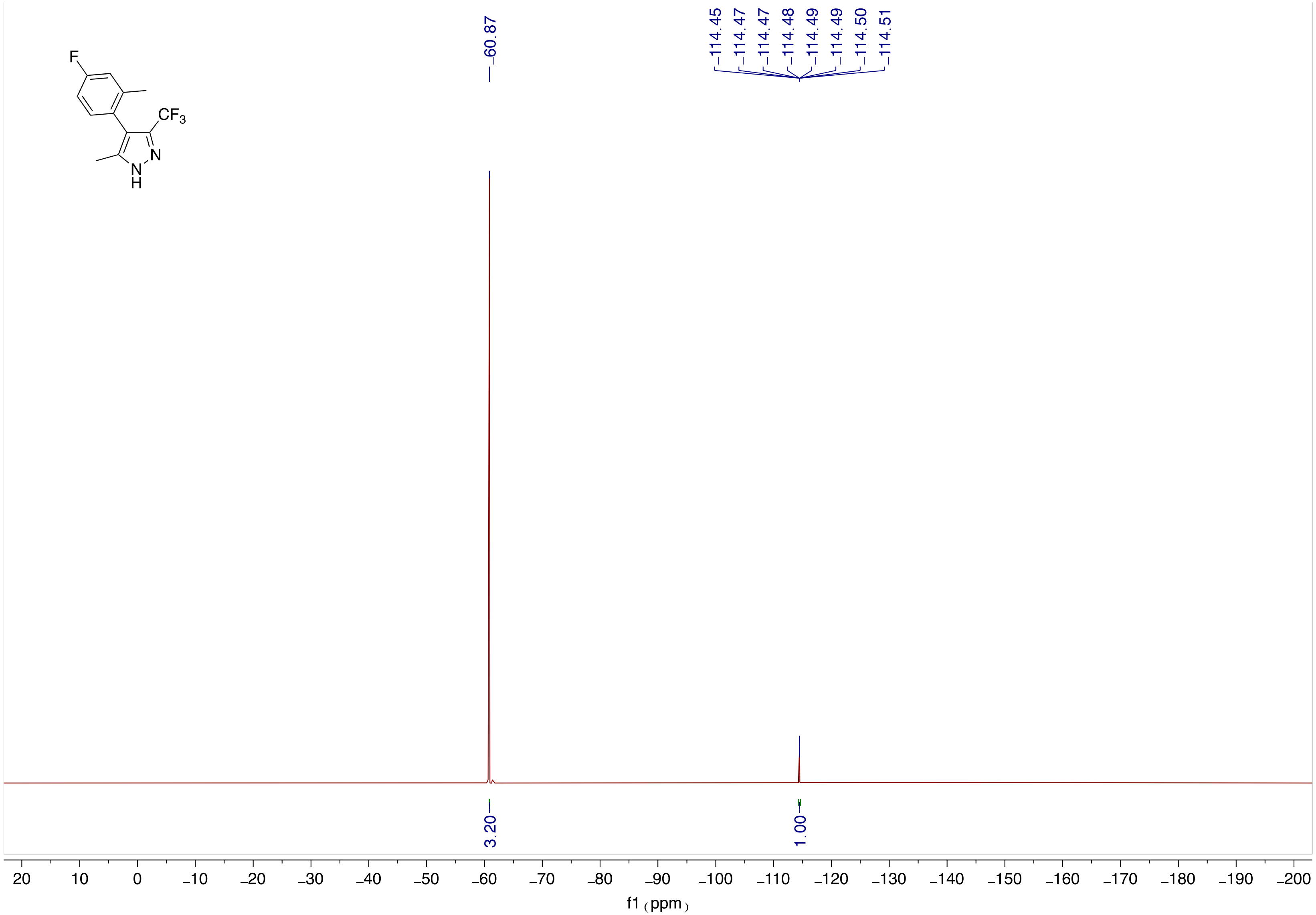

54 - ^{1}H NMR (400 MHz, CDCl$_3$):

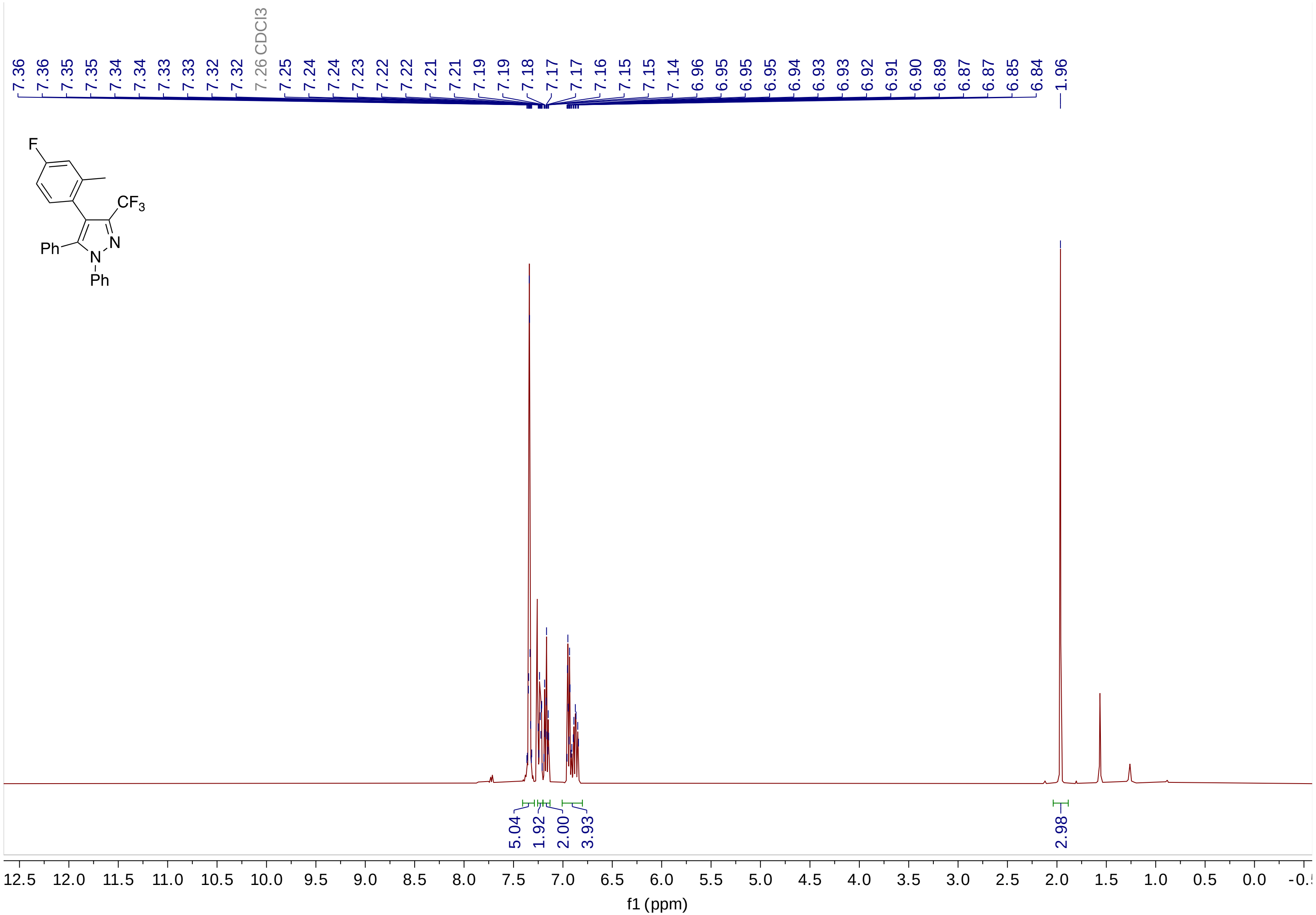

54 - $^{13}C\{^{1}H\}$ NMR (101 MHz, $CDCl_3$):

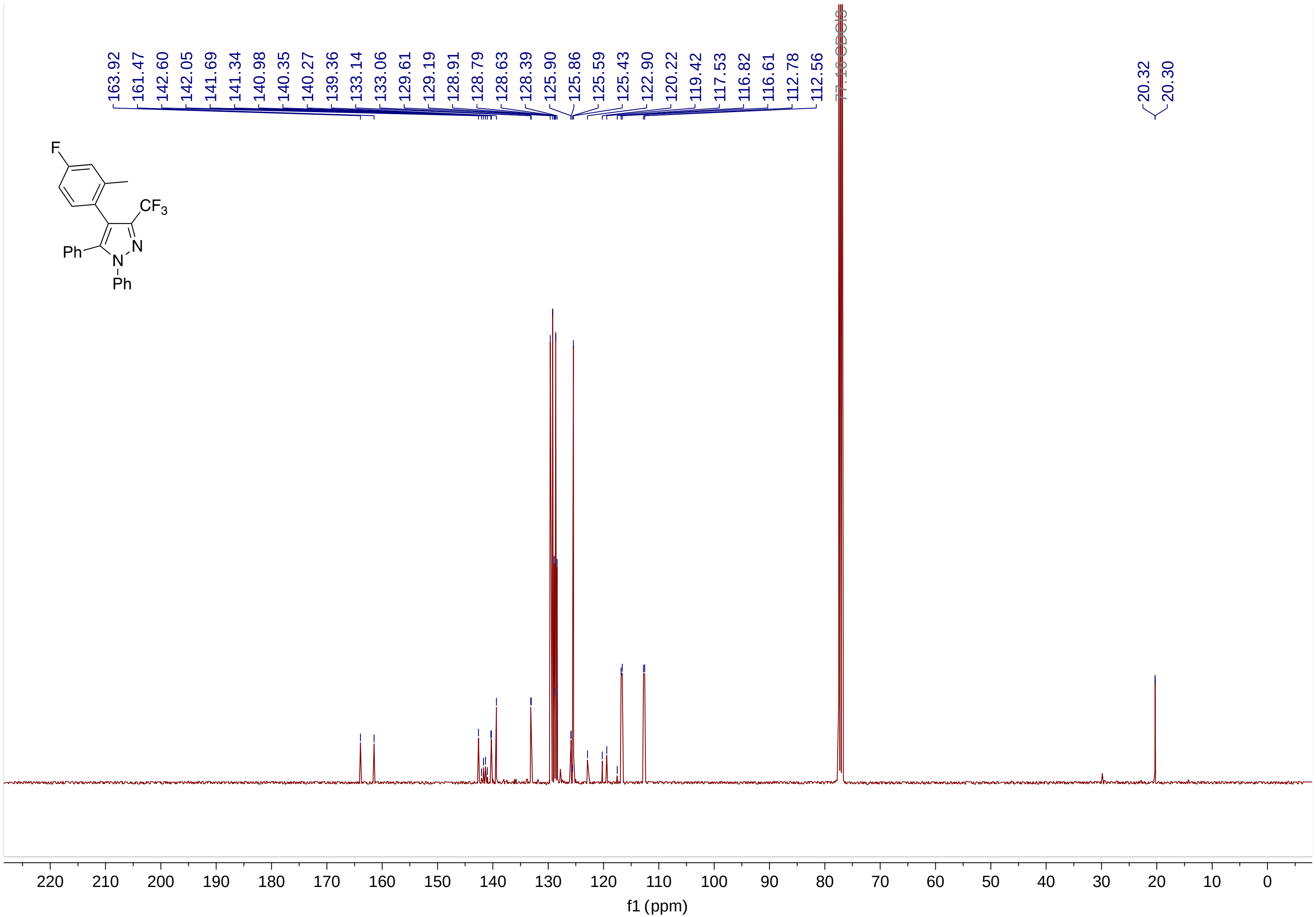

54 - ^{19}F NMR (377 MHz, $CDCl_3$):

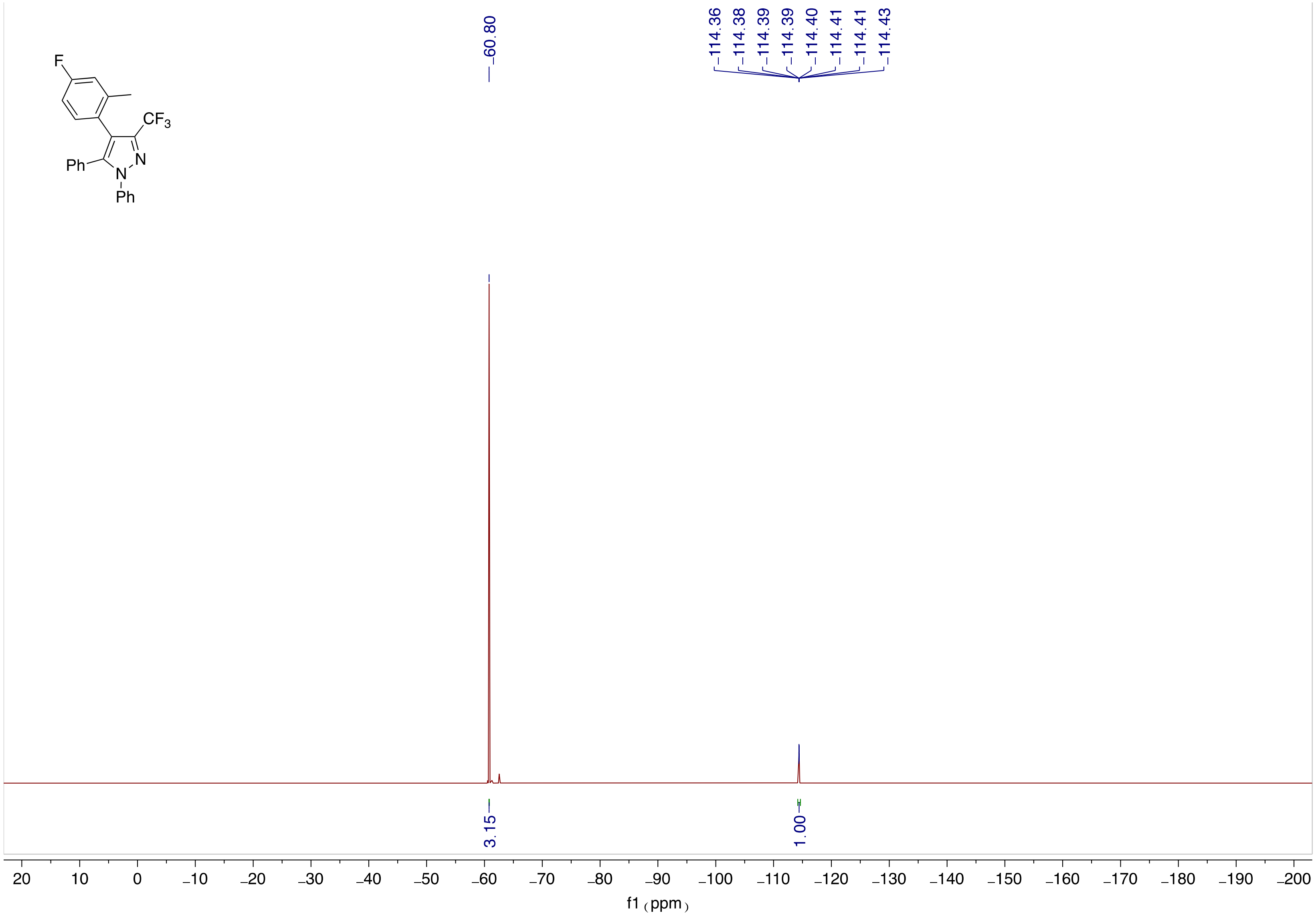

55 - ^{1}H NMR (500 MHz, CDCl$_3$):

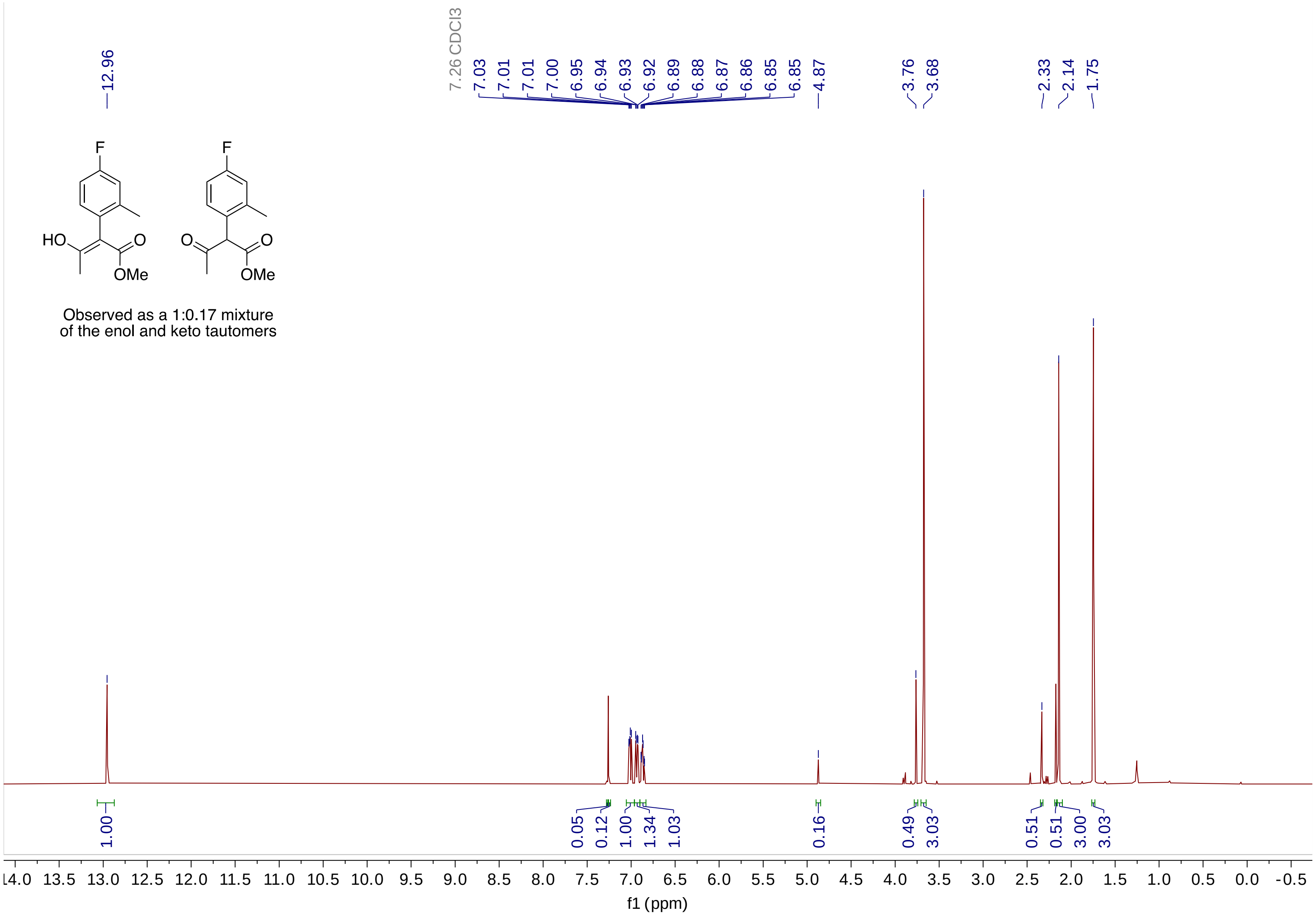

55 - $^{13}C\{^1H\}$ NMR (126 MHz, $CDCl_3$):

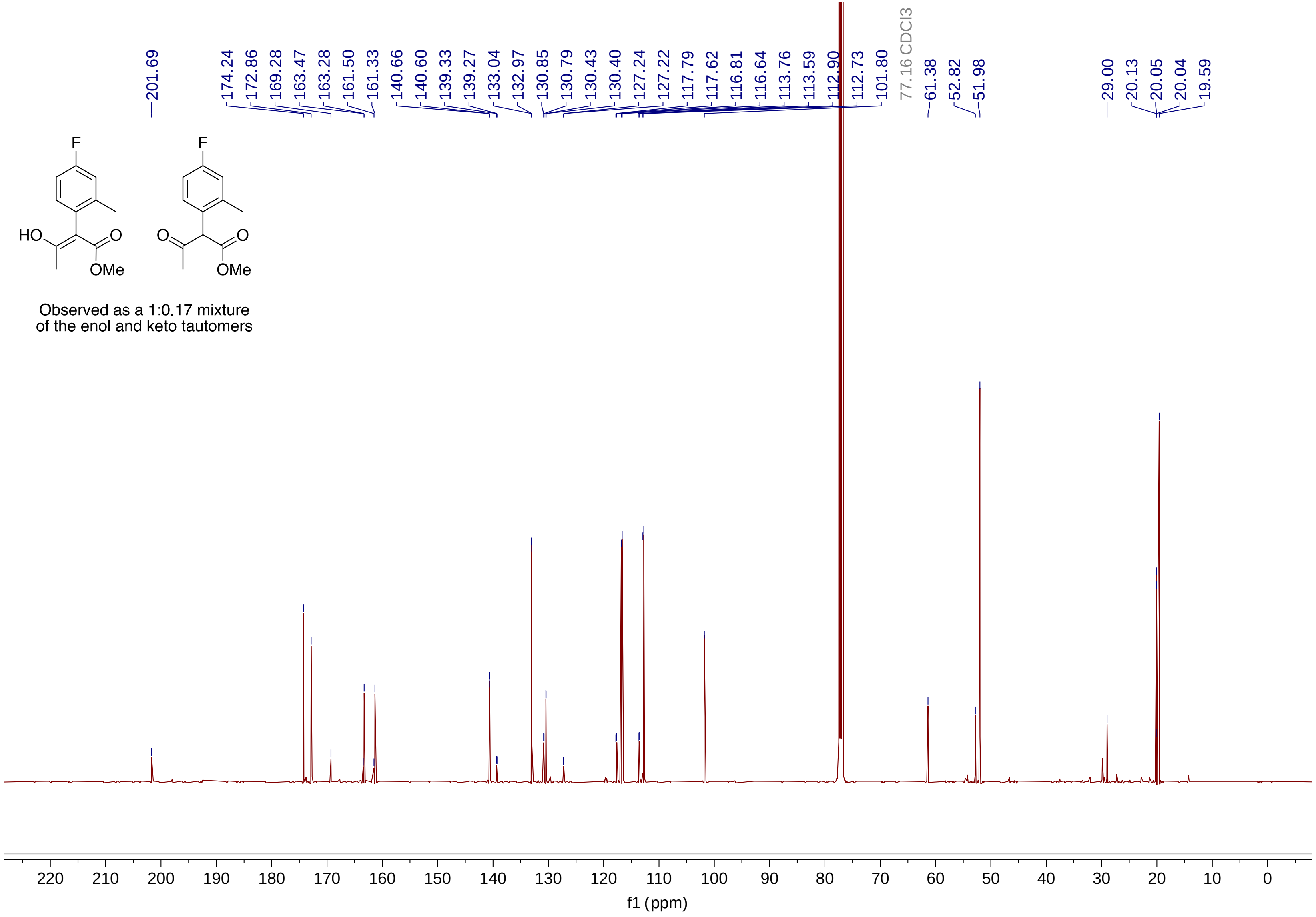

55 - ^{19}F NMR (377 MHz, $CDCl_3$):

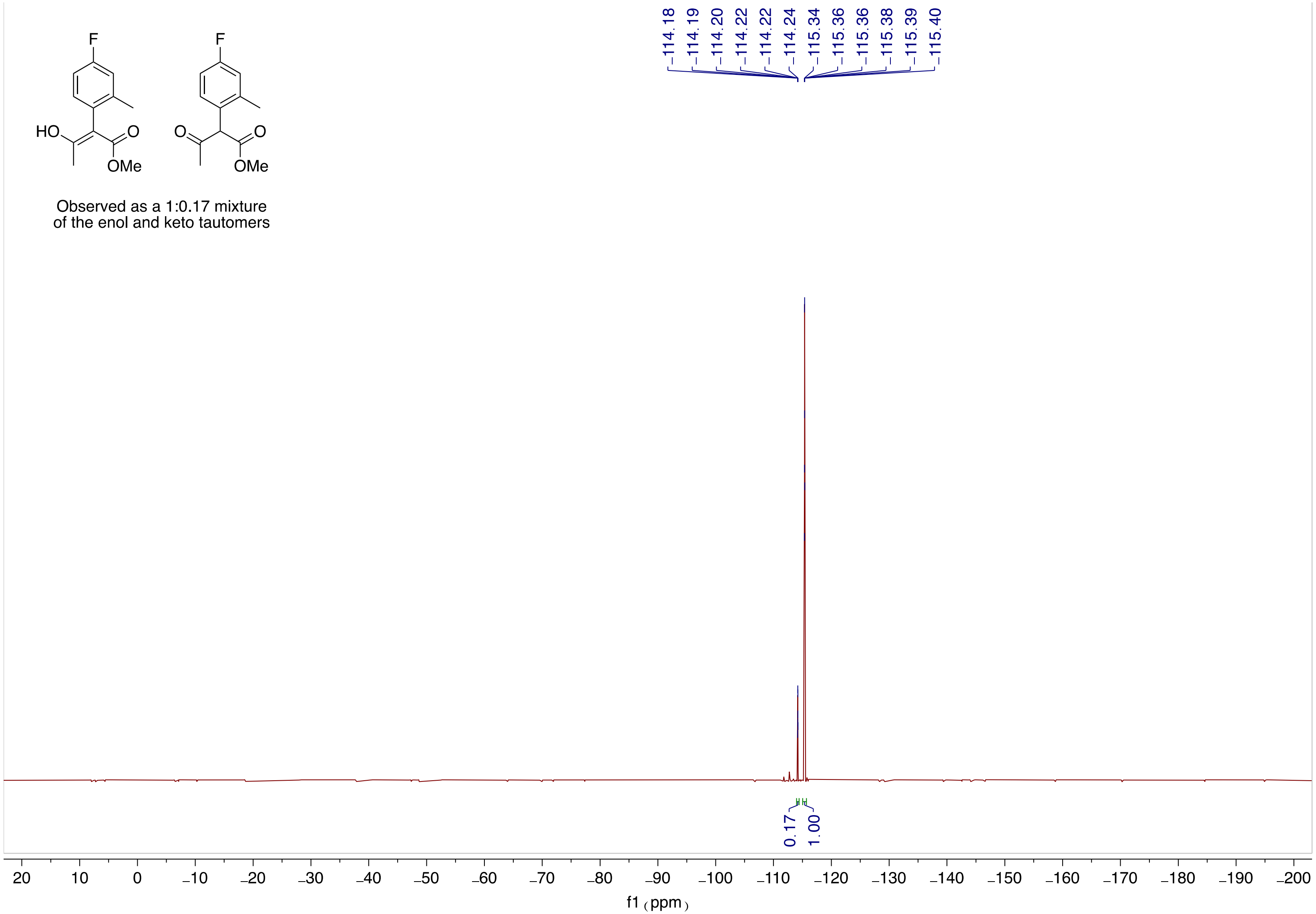

56 - ^{1}H NMR (400 MHz, $CDCl_3$):

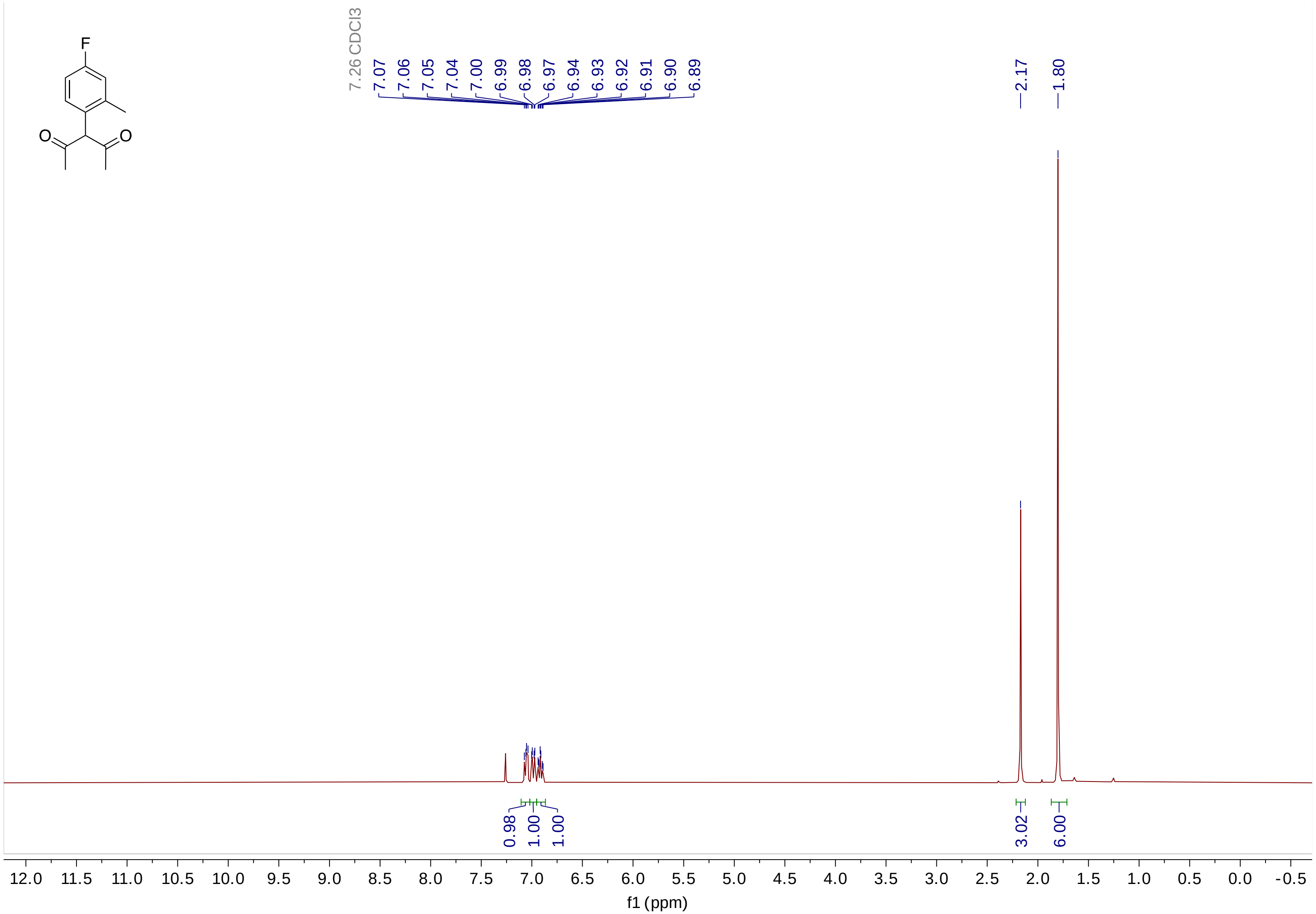

56 - $^{13}C\{^{1}H\}$ NMR (101 MHz, $CDCl_3$):

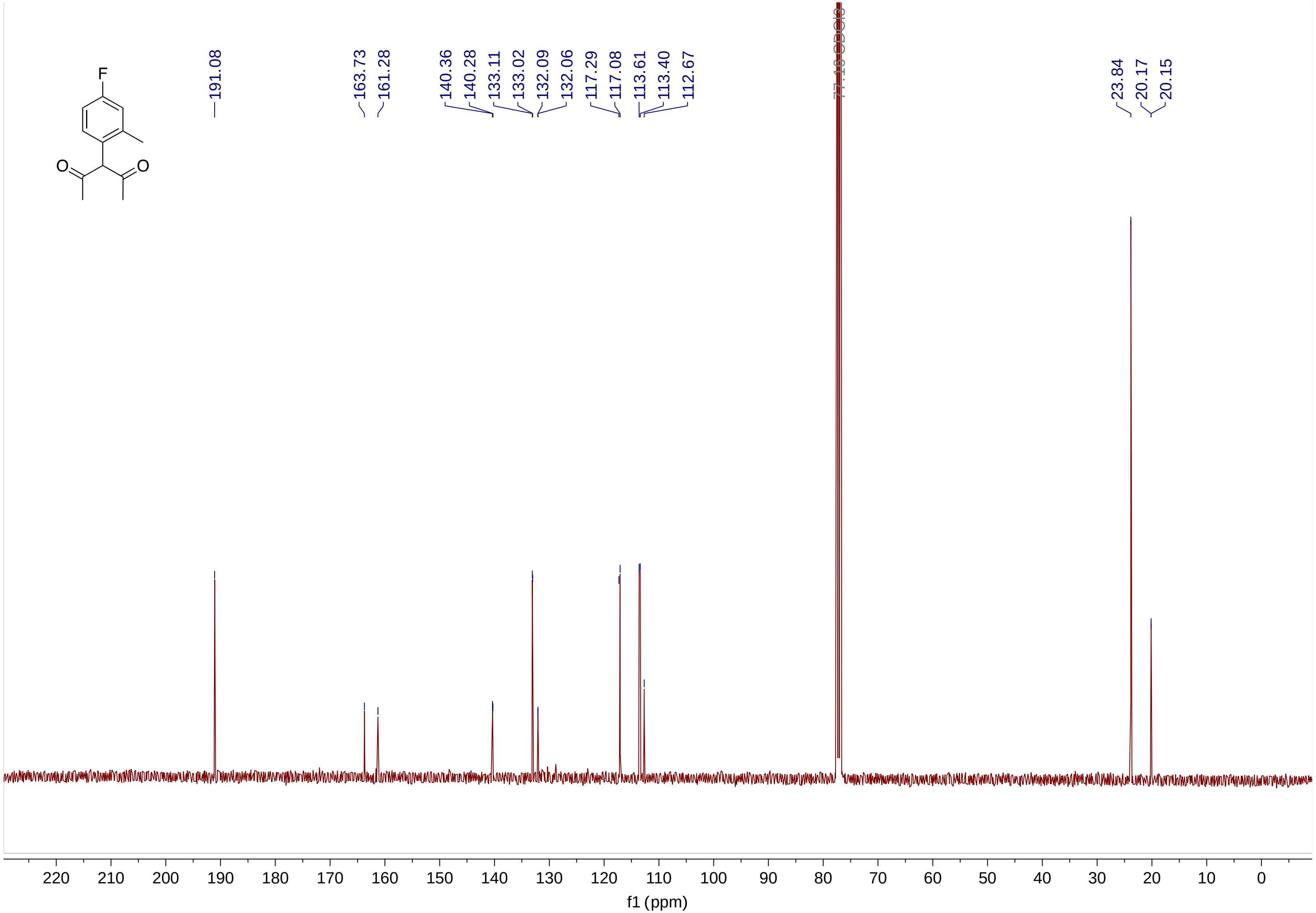

56 - ^{19}F NMR (377 MHz, $CDCl_3$):

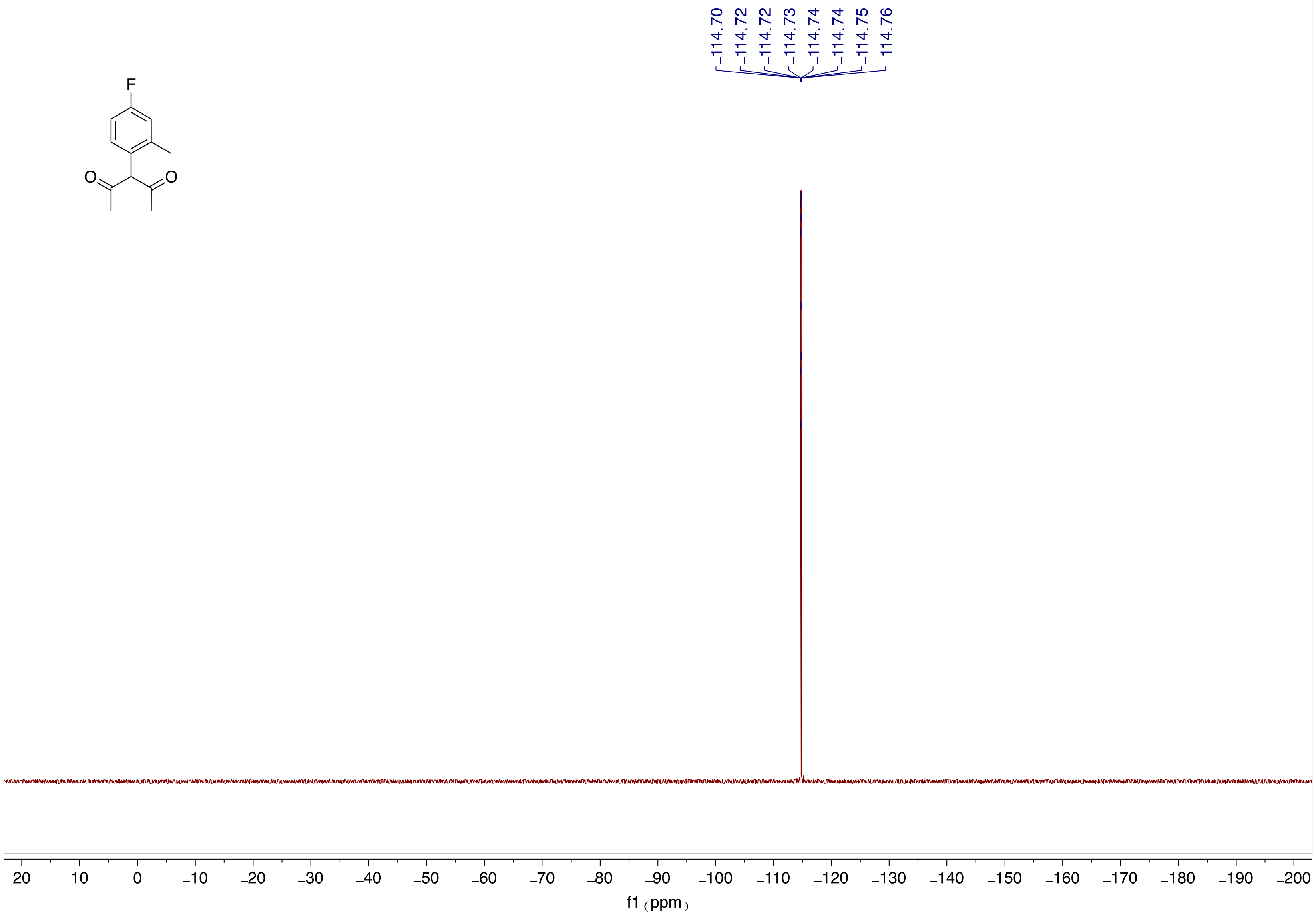

S6 - ^{1}H NMR (400 MHz, $CDCl_3$):

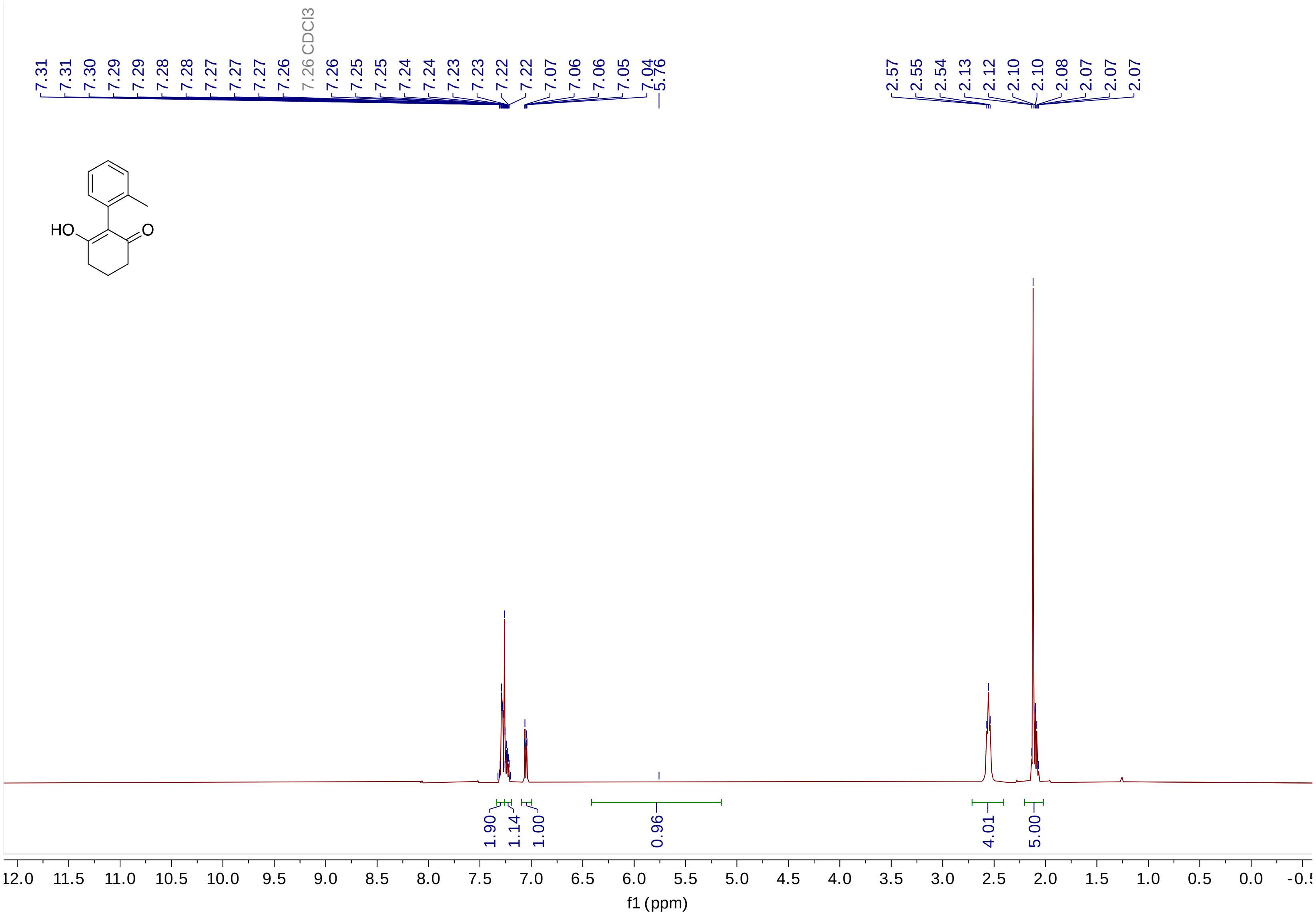

S6 - $^{13}C\{^{1}H\}$ NMR (101 MHz, $CDCl_3$):

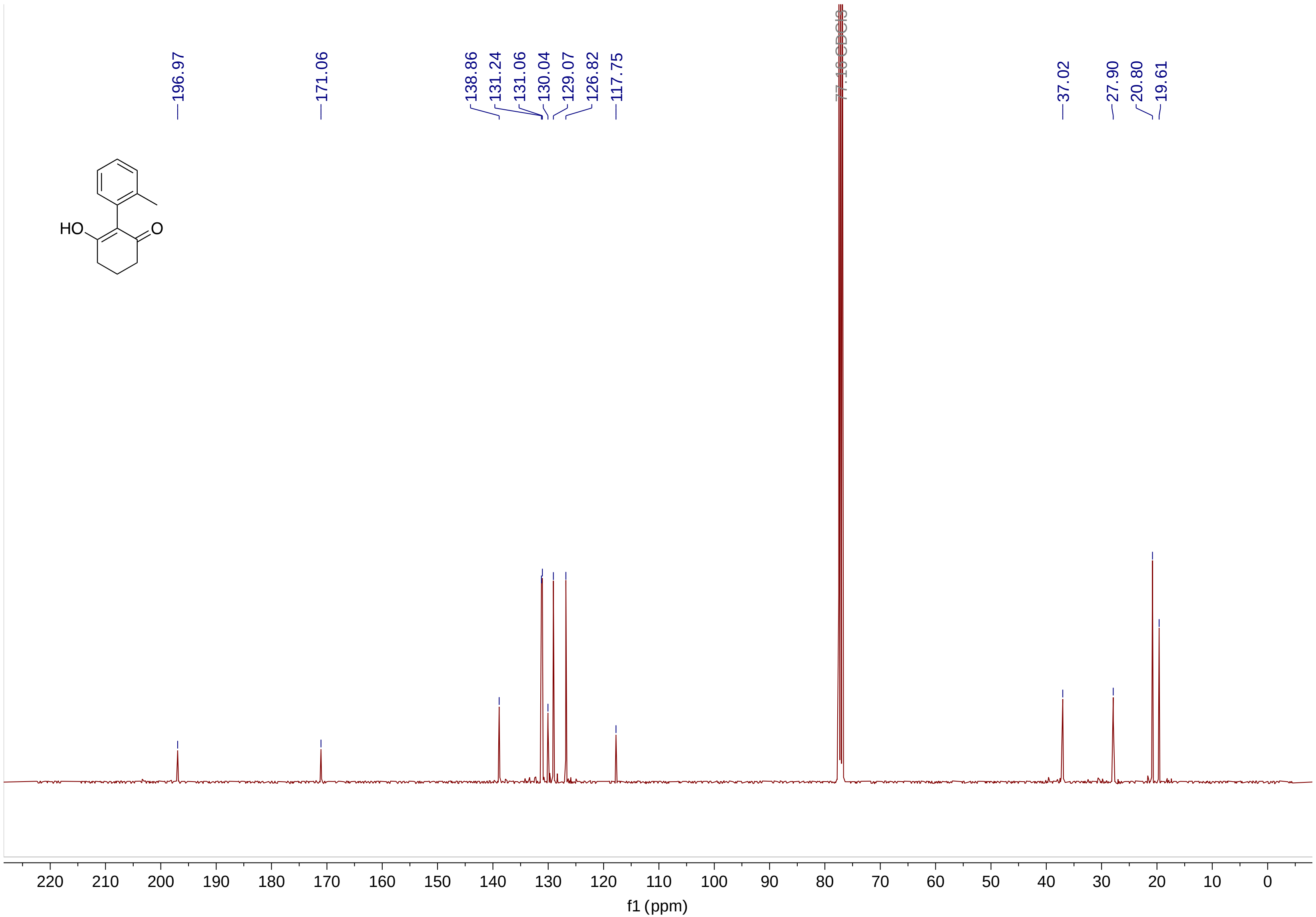

Supplement: Supplementary file 2 — Supporting Information [file ANIE-61-0-s002.pdf]
